# Highly selective synthesis of all-carbon tetrasubstituted alkenes by deoxygenative alkenylation of carboxylic acids

**DOI:** 10.1038/s41467-021-27507-x

**Published:** 2022-02-04

**Authors:** Yantao Li, Qianzhen Shao, Hengchi He, Chengjian Zhu, Xiao-Song Xue, Jin Xie

**Affiliations:** 1 State Key Laboratory of Coordination Chemistry, Jiangsu Key Laboratory of Advanced Organic Materials, Chemistry and Biomedicine Innovation Center (ChemBIC), School of Chemistry and Chemical Engineering, Nanjing University, Nanjing, 210023 China; 2 Key Laboratory of Organofluorine Chemistry, Shanghai Institute of Organic Chemistry, University of Chinese Academy of Sciences, Chinese Academy of Sciences, Shanghai, 200032 China; 3 State Key Laboratory and Institute of Elemento-Organic Chemistry, College of Chemistry, Nankai University, Tianjin, 300071 China; 4 State Key Laboratory of Organometallic Chemistry, Shanghai Institute of Organic Chemistry, Shanghai, 200032 China; 5 Advanced Catalytic Engineering Research Center of the Ministry of Education, Hunan University, Changsha, 410082 China

**Keywords:** Homogeneous catalysis, Synthetic chemistry methodology

## Abstract

The synthesis of all-carbon tetrasubstituted olefins under mild reaction conditions is challenging because of the inevitable issues including significant steric hindrance and the uncontrolled *Z/E* stereoselectivity. In this paper, we report the synthesis of all-carbon tetrasubstituted alkenes from readily available carboxylic acids and alkenyl triflates with the synergistic catalysis of cyclo-octa-1,5-diene(tetramethyl-1,4-benzoquinone)nickel and visible light under an air atmosphere, thus avoiding the need for a glovebox or a Schlenk line. A wide range of aromatic carboxylic acids and cyclic and acyclic alkenyl triflates undergo the C-C coupling process smoothly, forming structurally diverse alkenes stereospecifically in moderate to good yields. The practicality of the method is further illustrated by the late-stage modification of complex molecules, the one pot synthesis and gram-scale applications. This is an important step towards the valuable utilization of carboxylic acids, and it also simplifies the experimental operation of metallophotoredox catalysis with moisture sensitive nickel(0) catalysis.

## Abstract (web summary)

Tetrasubstituted olefins have been explored as chemical synthons and can sometime have useful photophysical properties, but are sometimes difficult to synthesize with high selectivity in mild conditions. Here the authors present a method to make tetrasubstituted olefins via dual photo- and nickel catalysis, without the need for an inert atmosphere.

## Introduction

**Fig. 1:**
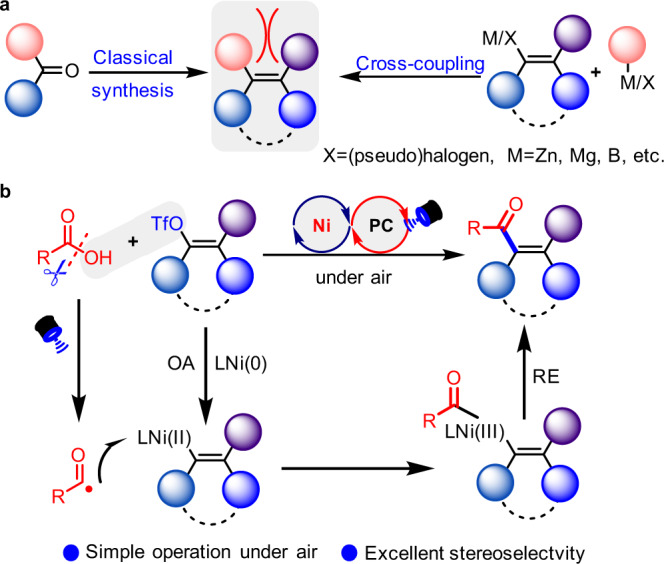
General strategies to access bulky alkenes. OA oxidative addition, RE reductive elimination. **a** Traditional methods to construct all-carbon tetrasubstituted olefins. **b** This work: Deoxygenative alkenylation of carboxylic acids.

The construction of all-carbon tetrasubstituted alkenes has long been a challenge in organic chemistry due to the significant steric hindrance and the uncontrolled *Z/E* stereoselectivity in the synthesis of such molecules^1^. The classical synthetic methods^2–5^, including Wittig olefination, Peterson olefination, Horner–Wadsworth–Emmons reaction, olefin-substitution and elimination of water from alcohols, do not proceed well and can result in poor selectivity and efficiency when used in an attempt to construct linear tetrasubstituted alkenes. Transition-metal-catalyzed C-C coupling reactions or carbometalation of internal alkynes have recently been identified as powerful tools with which to realize such stereospecific transformations (Fig. 1a)^6–17^. However, in these reactions, organometallic reagents derived from Zn, Al, and Mg are required to improve the reaction efficiency, and in turn decrease the compatibility of the reaction system, and require that moisture must be scrupulously avoided. Thus, development of a robust and practical synthetic methodology to address the existing limitations to access all-carbon tetrasubstituted alkenes is highly desirable.

Thanks to the pioneering works of MacMillan, Doyle, and Molander^18,19^, photoredox-nickel dual catalysis has proved to be successful as an efficient and mild strategy for these reactions^20–38^. Very recently, we have achieved cross-electrophile C-C coupling of aromatic acids and aromatic halides enabled by photoredox catalysis and nickel catalysis^39^. To the best of our knowledge, almost all the metallophotoredox catalyzed reactions require an inert reaction atmosphere, such as nitrogen or argon, and this requires use of a glovebox or the Schlenk technique. In 2020, Cornella and Engle independently reported two kinds of air-stable nickel(0)–olefin catalysts for Ni-catalyzed coupling^40,41^. Based on this point, we wondered if the synergistic photoredox and Ni(0) catalysis could be achieved under an air atmosphere. This technique has not been used to date but would greatly simplify the operation of metallophotoredox catalysis with nickel catalyst.

Carboxylic acids are commercially abundant, structurally diverse and generally stable feedstock chemicals commonly used in synthesis. With our continuing efforts in selective C-O bond functionalization of carboxylic acid^39,42–44^, we attempted to develop a practical strategy for the construction of all-carbon tetrasubstituted alkenes with a wide array of readily available cyclic and acyclic alkenyl triflates under air atmosphere (Fig. 1b). This reaction would be enabled by metallophotoredox catalysis using a photocatalyst and Ni(COD)(DQ) at room temperature without the involvement of organometallic reagents, in which the resultant acyl radical by photoredox catalysis would potentially add to Ni(II)-species to initiate the unprecedented C-C coupling for the synthesis of highly sterically congested ketones.

Here, we show a highly efficient metallophotoredox deoxygenative alkenylation of aromatic acids by means of Ni(COD)(DQ) under air conditions, constructing all-carbon tetrasubstituted alkenes from readily available starting materials.

## Results

### Reaction optimization

To initiate the study of cross-coupling used for the synthesis of all-carbon tetrasubstituted alkenes, 4-fluorobenzoic acid (**1a**) with ethyl 2-(((trifluoromethyl)sulfonyl)oxy)cyclohex-1-ene-1-carboxylate (**2a**) were selected as a model reaction. The optimized reaction conditions (Table 1) were identified as 1 mol% [Ir{dF(CF_3_)ppy}_3_{dtbbpy}]PF_6_, 3 mol% Ni(COD)(DQ), 5 mol% 4,4′-di-methyl-2,2′-bipyridine (**L1**), 1.5 equiv Ph_3_P, and 2.0 equiv Na_2_CO_3_ under blue LEDs in DMF. The reaction afforded **3a** in 90% isolated yield under an air atmosphere (entry 1). When **L2**, or **L3** was employed instead of **L1**, the yield was not improved (entries 2–3). The photocatalyst **PC-2** proved to be ineffective for this transformation (entry 4). When the solvent was changed from DMF to MeCN, DCM or THF, the yield of target product (**3a**) significantly decreased (entries 5-7), probably as the inorganic base can dissolve better in DMF to promote the reaction process. The use of Cs_2_CO_3_ as the base also compromised the reaction efficiency (entry 8). Control experiments demonstrated that photocatalyst, nickel catalyst, Ph_3_P and light were all necessary for the reaction to occur (entry 9).

**Table 1:** Optimization of the reaction conditions^a^.


Entry	Variation of standard conditions	Yield(%)^b^
1	none	90
2	**L2** instead of **L1**	70
3	**L3** instead of **L1**	60
4	**PC-2** instead of **PC-1**	Trace
5	MeCN as solvent	Trace
6	DCM as the solvent	ND
7	THF as the solvent	10
8	Cs_2_CO_3_ as base	32
9	No **PC-1** or [Ni] or Ph_3_P or light	ND


DMF *N*,*N*-dimethylformamide, ND not detected, Ni(COD)(DQ) cyclo-octa-1,5-diene(tetramethyl-1,4-benzoquinone)nickel.

^a^Standard conditions: **PC-1** (1 mol%), Ni(COD)(DQ) (3 mol%), **L1** (5 mol%), **1a** (0.2 mmol), **2a** (0.3 mmol), Ph_3_P (0.3 mmol), Na_2_CO_3_ (0.4 mmol), DMF (3.5 mL), blue LEDs, under air atmosphere at ambient temperature, 24 h.

^b^Isolated yields.

### Substrate scope

**Fig. 2:**
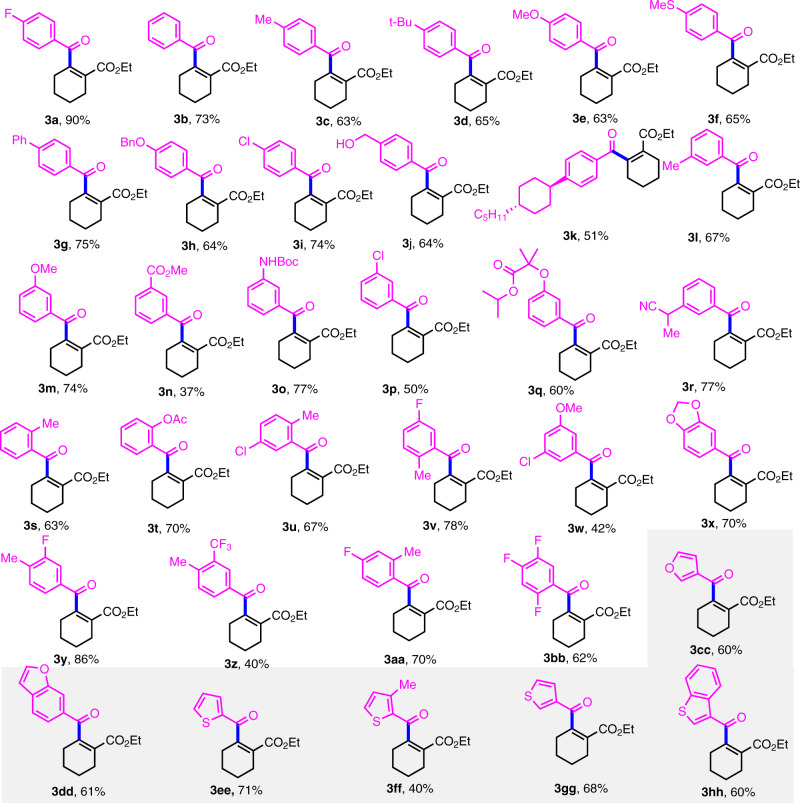
Scope of aromatic acids. Standard conditions: **PC-1** (1 mol%), Ni(COD)(DQ) (3 mol%), **L1** (5 mol%), **1a** (0.2 mmol), **2a** (0.3 mmol), Ph_3_P (0.3 mmol), Na_2_CO_3_ (0.4 mmol), DMF (3.5 mL), blue LEDs, under air, ambient temperature, 24 h. Isolated yields.

With the standard conditions in hand and using ethyl 2-(((trifluoromethyl)sulfonyl)-oxy)cyclohex-1-ene-1-carboxylate (**2a**) as a coupling partner, a wide range of aromatic carboxylic acids were investigated, and the results shown in Fig. 2 were obtained. Aromatic acids with one *para*-substituent on the phenyl group were found to produce the desired product (**3a**–**3k**) with satisfactory yields. A series of versatile functional group substituents, such as -methylthio, -benzyloxy, and - hydroxymethyl are tolerated well under the optimized reaction conditions, and aromatic acids bearing electron-withdrawing or electron-donating groups at the *meta*- or *ortho*-position delivered the desired products (**3l**–**3t**) in moderate to good yields. A series of useful functional groups, such as reactive carbonyl (**3n**, **3o**, **3q**) and cyano groups (**3r**) are also compatible. Significantly, multi-substituted carboxylic acids do not detract from the reaction efficiency, giving rise to the desired products (**3u**-**3bb**) in synthetically useful yields. Heteroaromatic carboxylic acids engage in this C-C coupling process successfully, producing desired the tetrasubstituted alkenes (**3cc-3hh**) in 40–71% yields.

**Fig. 3:**
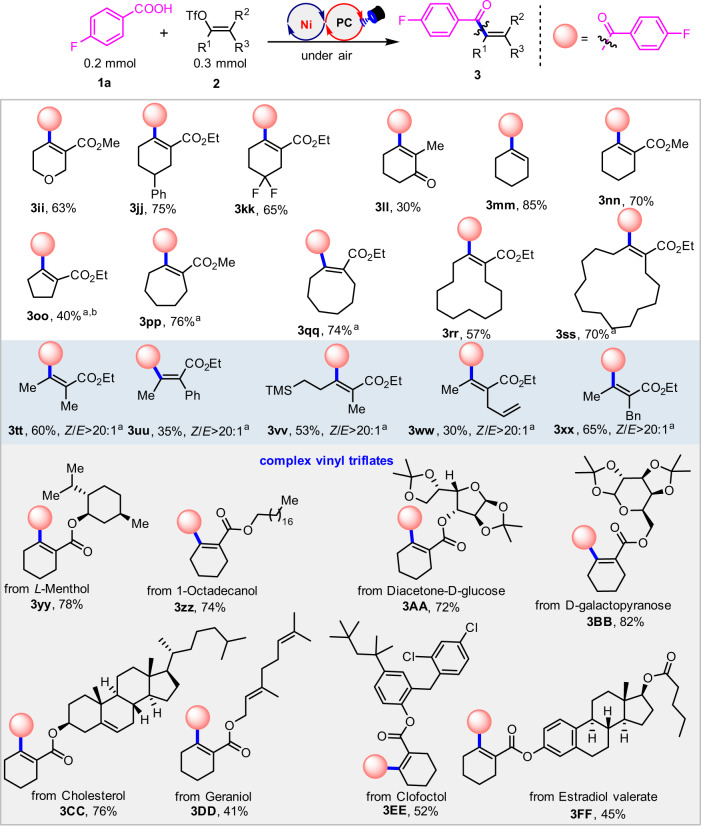
Scope of alkenyl triflates. Standard conditions: **PC-1** (1 mol%), Ni(COD)(DQ) (3 mol%), **L1** (5 mol%), **1a** (0.2 mmol), **2** (0.3 mmol), Ph_3_P (0.3 mmol), Na_2_CO_3_ (0.4 mmol), DMF (3.5 mL), blue LEDs, under air, ambient temperature, 24 h. Isolated yields. ^a^Modified standard conditions: **PC-1** (2 mol%), Ni(COD)(DQ) (6 mol%), **L1** (10 mol%), **1a** (0.2 mmol), **2** (0.3 mmol), Ph_3_P (0.3 mmol), Cs_2_CO_3_ (0.3 mmol), DMF (3.5 mL), blue LEDs, under air, ambient temperature, 24 h, isolated yields. ^b^0.4 mmol K_2_CO_3_ instead of Cs_2_CO_3_.

Subsequently we explored the scope of the enol triflates (Fig. 3). Generally, the ester substituents on the six membered rings may have negligible influence of the transformation and deliver the products (**3ii**–**3kk**). When there is a methyl substituent on the side chain, as in **3ll**, the yield declines to 30%. It was envisioned that the ester group could play a role in the stabilization of the transition-state with coordination to the nickel center. The reaction is also suitable for the synthesis of trisubstituted alkenes in good yield (**3mm**). We envisioned that the trisubstituted alkene is likely to be less congested than tetrasubstituted alkene, where the chelation stabilization of ester group is not vital. The replacement of the carboethoxy group in the ester unit with a carbomethoxy group delivered the product (**3nn**) in 70% yield. In addition to six membered rings, other kinds of rings bearing alkenyl triflates react well. For example, the five-membered alkenyl triflate can undergo this C-C cross-coupling in moderate yield (**3oo**). Alkenyl triflates derived from 7-, 8-, 12-, or 15-membered cyclic ketones, can uniformly undergo this transformation to afford the desired products (**3pp–3ss**) in 57-76% yields. When acyclic alkenyl triflates were subjected to this protocol, the desired linear all-carbon tetrasubstituted alkenes (**3tt–3xx**) were obtained in moderate yields and with excellent *Z/E* stereoselectivity. The stereochemistry of the alkenyl triflate starting materials is reliably translated into the products. Interestingly, it was found that the tetramethylsilyl group and terminal alkenes remain intact during the coupling (**3vv**, **3ww**). To demonstrate the inherent value of the methodology, the strategy was applied to the construction of a series of complex all-carbon tetrasubstituted alkenes (**3yy**–**3FF**). Many complex alkenyl triflates bearing different functional groups tolerate the conditions well, affording a series of structurally diverse all-carbon substituted alkenes in satisfying yields of 41–82% under air atmosphere conditions.

### Mechanistic studies

**Fig. 4:**
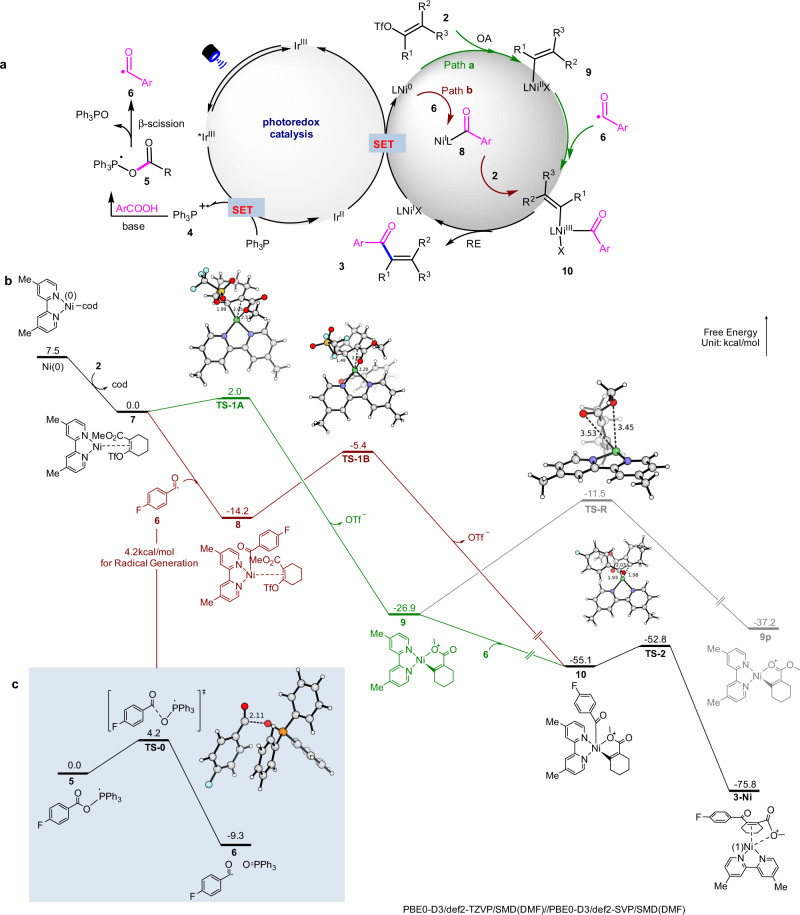
Mechanistic studies. **a** Proposed mechanism. **b** DFT calculation results. PES of the reaction with **7** as the reference point (radical coupling is barrierless confirmed by scan study, see details in SI.). **c** PES of the reaction with **5** as the reference point.

Two possible dual catalytic mechanisms are depicted in Fig. 4a^20–38^. Firstly, under blue light irradiation, photo-excited *[Ir(dF(CF_3_)ppy)_2_(dtbbpy)]PF_6_ [^1/2^*E*_red_ (*Ir^III^/Ir^II^) = +1.21 V vs SCE = 2.3 τ = 2.3 μs]^45^ can be generated, and is able to undergo a single electron oxidation with PPh_3_ to generate a phosphoryl radical (**4**)^42^. In the presence of base, the resultant carboxylate anion would recombine with the phosphoryl radical (**4**) to produce the key intermediate (**5**), which can generate an acyl radical (**6**) via C-O homolysis^46^. Meanwhile, the alkenyl triflate (**2**) would undergo oxidative addition with L_n_Ni(0) to produce the (alkenyl)Ni(II) complex (**9**), which can be intercepted by the resulting acyl radical (**6**), to produce a Ni(III) species (path **a**). In an alternative mechanistic pathway (path **b**), the acyl radical (**6**) is captured by Ni(0), forming an acylnickel(I) intermediate (**8**) prior to oxidative addition to **2**, forming the Ni(III)-intermediate (**10**). The reductive elimination of this Ni(III)-intermediate (**10**) would deliver the desired product (**3**). Concurrently, single-electron transfer from Ir(II) to the Ni(I) regenerated both **PC-1** and Ni(0), completing both catalytic cycles.

The main difference between path **a** and path **b** is that either Ni(0) or Ni(I) is acting as the active species and DFT calculations were conducted to shed light on this question (Fig. 4b). Based on the energy profile calculated at the level of PBE0-D3/def2-TZVP/SMD(DMF)//PBE0-D3/def2-SVP/SMD (DMF) in Fig. 4b, once the acyl radical (**6**) is formed, it could be captured rapidly in a barrierless manner to give Ni(I) (Path **b**). But the generation of the acyl radical (**6**) should be slower than the oxidative addition (OA) (path **a**) according to the computed barriers (ΔG^≠^ = 4.2 kcal/mol vs 2.0 kcal/mol). Accordingly, the Ni(0) will be converted to Ni(II) (**9**) before any radical is formed. As a result, the reaction goes through path **a** as the radical generation loses the competition with the Ni(0) oxidative addition.

The shape of the potential energy surface (PES) in Fig. 4b is commonly observed in this type of catalysis that consists of Ni(0) and a radical^47–50^. The oxidative addition barrier of Ni(I) is consistently significantly lower than the oxidative addition barrier of Ni(0), and is always limited by the concentration of the radical. This renders the determination of the preferred pathway difficult^47–50^. Our DFT results provide a strategy for this type of reaction by comparing the Ni(0) oxidative addition barrier with the overall barrier of radical generation. In our case, the Ni(0) oxidative addition barrier is low enough that the higher barrier is associated with the radical generation. This small barrier for a crowded transition state is a result of the neighboring ester group stabilization (d_O…Ni,TS-A_ = 2.57 Å), which is sterically infeasible in the reactant.

In those TSs (**TS-1A** and **TS-1B**) of oxidative addition (OA), an S_N_2′-like process is observed. The O-atom in ester moiety attacks the Ni center and pushes the electron to the middle carbon which then kicks off the -OTf as a leaving group, resulting in the formation of a chelated product **9** or **10**. With **9** formed by the favorable path **a**, a competition exists between isomerization into a more stable **9p** and recombination with free acyl radical **6**. The energy barrier of the former is 15.4 kcal mol^−1^, which is higher than the estimated barrier of the radical generation (4.2 kcal mol^−1^). Therefore, before the isomerization of **9**, there will be sufficient concentration of acyl radicals to complete the radical addition and irreversibly produces the pentacoordinate **10** (See Supplementary Information for detailed discussion of the active species for radical addition among Ni(II) isomers, including a more comprehensive PES with the potential OTf coordination). The final reductive elimination **TS-2** has a low barrier of 2.3 kcal mol^−1^ that gives the final product swiftly. The mechanistic control experiments (Supplementary Fig. 13) also suggest that the radical addition to Ni(0) is less likely (Path **b**).

### Synthetic application

**Fig. 5:**
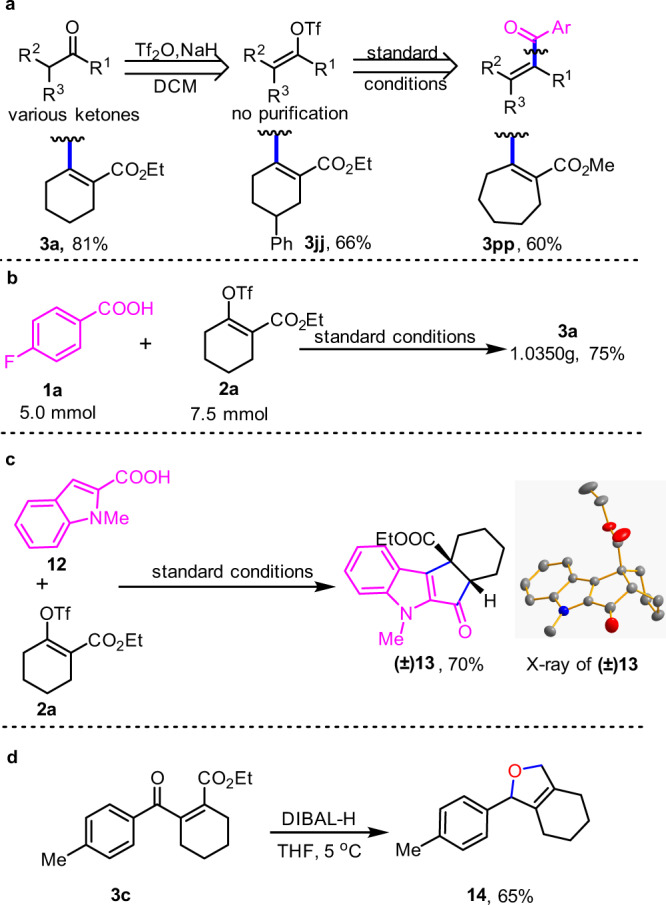
Synthetic application. **a** one-pot two-steps synthesis of **3**. **b** Gram-scale experiment. **c** Tandem C-C coupling/Nazarov cyclization. **d** Reduction of **3c**.

With this possible mechanistic understanding of this coupling reactions, we explored its synthetic applications (Fig. 5). As shown in Fig. 5a, we developed a one-pot, two-step strategy for the concise synthesis of all-carbon tetrasubstituted alkenes directly from ketones. In addition, this protocol was found to be easily used at the gram scale (Fig. 5b). Interestingly, when indole-based aromatic acid (**12**) was subjected to this protocol, it can undergo a tandem C-C coupling and subsequent Nazarov cyclization to afford product (**13**) in 70% yield. The structure of **13** was confirmed by X-ray single crystal analysis (Fig. 5c). Interestingly, reduction of product **3c** with diisobutylaluminium hydride (DIBAL-H) can directly lead to all-carbon tetrasubstituted alkenes, hexahydroisobenzofuran product **14** (Fig. 5d).

## Discussion

We have developed a strategy for the synthesis of all-carbon tetrasubstituted alkenes via synergistic photoredox/nickel catalysis under an air atmosphere. A large number of readily available aromatic acids and alkenyl triflates are efficient coupling partners in this C-C coupling, affording a rich library of structurally diverse acyclic and cyclic all-carbon tetrasubstituted alkenes in moderate to good yields and with excellent *Z*/*E* stereoselectivity. DFT calculations indicate that this transformation may proceed via a Ni(0)/Ni(II)/Ni(III) pathway. This protocol can avoid the utilization of moisture-sensitive organometallic reagents and the need for an inert gas atmosphere, greatly simplifying the operation of metallophotoredox reactions with Ni(0) catalysis. This is also an important advance in the synthesis of all-carbon tetrasubstituted alkenes from readily available feedstock chemicals.

## Methods

### General procedure for the synthesis of all-carbon tetrasubstituted alkenes

Under air atmosphere, a stirring bar, Ni(COD)(DQ) (3.0 mol%), 4,4′-di-methyl-butyl-2,2′-bipyridine (5.0 mol%), and DMF (2.0 mL) were successively added to a vial (2.0 mL). The vial was stirred until the resulting mixture become homogeneous (about 20 min). Subsequently, photocatalyst Ir[dF(CF_3_)ppy]_2_(dtbbpy)PF_6_ (1 mol%), aromatic carboxylic acid (0.2 mmol, 1.0 equiv), triflates (0.3 mmol, 1.5 equiv), Ph_3_P (0.3 mmol, 1.5 equiv), and Na_2_CO_3_ (0.4 mmol, 2.0 equiv) were added to an 3.5 mL screw-cap vial equipped with a magnetic stirring bar. The resulting homogenous solution was syringed into the vial. Then DMF (1.5 mL) was added into the vial. The vial was sealed and placed ~5 cm blue LEDs. The reaction mixture was stirred for 24 h at room temperature (air conditions were used to keep the temperature is 25 °C or so). After reaction completion, the reaction mixture was removed from the light. The solvent was removed and the residue was purified by flash chromatography on silica gel to afford the corresponding products.

## Supplementary information

Supplementary Information

## Data Availability

The authors declare that all other data supporting the findings of this study are available within the article and Supplementary Information files, and also are available from the corresponding author. The X-ray crystallographic data of product **13** in this study has been deposited in the Cambridge Crystallographic Data Centre under accession code CCDC 2089722.
