# Supplementary material for: Highly selective synthesis of all-carbon tetrasubstituted alkenes by deoxygenative alkenylation of carboxylic acids
Source: Nat Commun. 2022 Feb 4;13:10. doi: 10.1038/s41467-021-27507-x (PMC8816943; doi:10.1038/s41467-021-27507-x)
Supplement: Supplementary file 1 — Supplementary Information [file 41467_2021_27507_MOESM1_ESM.pdf]

SUPPLEMENTARY INFORMATION

Highly Selective Synthesis of All-Carbon Tetrasubstituted Alkenes by Deoxygenative Alkenylation of Carboxylic Acids

Li et al.

Table of Contents

1. Supplementary Note 2
2. Supplementary Methods, Tables and Figures 2
    2.1 Preparation of substrates 2
    2.2 General procedure 11
    2.3 X-Ray Crystal Data 13
    2.4 Characterization of products 15
    2.5 Investigation of the reaction mechanism 39
    2.6 Synthetic application 54
    2.7 DFT calculations 58
    2.8 NMR spectra 95
3. Supplementary References 207

1. Supplementary Note

All the reactions were conducted in oven-dried Schlenk tubes under air unless otherwise noted. All solvents were purified by Solvent Purification System. Reagents were purchased from Energy Chemical, TCI and etc. Flash column chromatographic purification of products was accomplished using forced-flow chromatography on Silica Gel (300-400 mesh). ^{1}H NMR, ^{13}C NMR and ^{19}F NMR spectra were recorded on a 400 MHz or 500 MHz spectrometer in $CDCl_3$ (δ H = 7.26 ppm, δ C = 77.0 ppm as standard). Data for ^{1}H NMR are reported as follows: chemical shift (ppm, scale), multiplicity, coupling constant (Hz), and integration. Data for ^{13}C NMR are reported in terms of chemical shift (ppm, scale), multiplicity, and coupling constant (Hz). Abbreviations for signal couplings are: s, singlet; d, doublet; t, triplet; m, multiple. GC-MS analyses were performed on a GC-MS with an EI mode. High resolution mass spectra were obtained using an Agilent 6210 Series TOF LC-MS equipped with electrospray ionization (ESI) probe operating in positive ion mode. Melting points (mp) were determined with a digital electrothermal apparatus without further correction. IR spectra were recorded on a Thermo Scientific Nicolet 380 FT-IR spectrometer. The 45 W blue LED lamps (λmax = 455 nm) were purchased from Kessil (A360NE/WE).

2. Supplementary Methods, Tables and Figures

2.1 Preparation of substrates

General Procedures:

Method A: Reaction was conducted under Argon atmosphere. A dry round-bottom flask equipped with a magnetic stirbar was charged with dry dichloromethane (12.0 mL) and the flask was cooled to 0 °C in an ice bath. Next, NaH 60% dispersion in mineral oil (144 mg, 3.60 mmol, 1.2 equiv) was added in one portion and the suspension was stirred for 5 minutes. Then, a solution of the β-keto ester (3.00 mmol, 1.0 equiv) in dry dichloromethane (3.0 mL) was added dropwise to the suspension at 0 °C. Once the addition was finished, the mixture was stirred for 30 min at 0 °C. After that time, Tf_2O 1M in CH_2Cl_2 (3.6 mL, 3.60 mmol, 1.2equiv) was added dropwise to the content at 0 °C and the reaction mixture was warmed to room temperature and stirred overnight. Then it was quenched with the addition of H_2O (10 mL) and the resulting layers were separated, and the aqueous phase was extracted with CH_2Cl_2 (2 x 20 mL). The combined organic phase was washed with brine, dried over $MgSO_4$ and filtered. The solvent was removed and the residue was purified by flash chromatography on silica gel to afford the corresponding products.[1]

Method B: To an oven dried 50 mL round bottom flask was added 168 mg of NaH (60% dispersion in mineral oil, 5.0 mmol, 1.0 equiv). The flask was purged with N_2 and 15 mL of

THF was added. Then 1.6 g of β-keto ester (5.0 mmol, 1.0 equiv), dissolved in 5 mL of THF, was added dropwise. Caution: H_2 is evolved during the addition. The reaction mixture was stirred for 15 min followed by addition of 2.0 g of $PhNTf_2$ (5.6 mmol, 1.1 equiv) in one portion. The reaction mixture was stirred for 6 h followed by quenching with 5 mL of water. The reaction mixture was extracted with 1x50 mL of Et_2O, organic layer washed with 1X10 mL of brine, dried over $MgSO_4$, and solvents evaporated under reduced pressure. The product was then purified by flash column chromatography (20→40% Et_2O:hexanes) to afford the corresponding products.[2]

Method C: To a solution of β-keto ester (1.0 equiv) in THF (0.2 M) was slowly added a solution of KHMDS (1.2 equiv) in THF at – 78 °C. After stirring for 1h, a solution of Comins' reagent (1.2 equiv) in THF was added dropwise to the reaction. The resulting mixture was then gradually warmed to room temperature. After stirring for overnight, the reaction was quenched by saturated aqueous NH_4Cl solution. The mixture was then extracted with EtOAc. The combined organic phases were washed by brine, dried over Na_2SO_4, filtered and concentrated in *vacuo*. Purification by MPLC (2:98 to 5:95 EtOAc: hexane) afforded the product.[3]

Method D: **General Procedure for the Synthesis of (*Z*)-Enol Triflates**

The starting acetoacetate derivative (4 mmol) was added to a round-bottom flask and dissolved in either hexanes or toluene (20 mL, 0.2M). The solution was cooled with an ice bath to 5-10°C (internal temperature) followed by addition of a saturated aqueous solution of LiOH (6 mL, ~30 mmol) in one portion. The resulting biphasic mixture was ***vigorously stirred*** at 5-10°C for ~5 minutes followed by the addition of triflic anhydride (10 mmol) dropwise at a rate to maintain the internal temperature between 5-15 ºC. Upon completion of the reaction (as judged by TLC, typically <10 min), the biphasic solution was diluted with H_2O (5mL) and the layers were separated. The aqueous layer was extracted with EtOAc (1 x 10mL). The combined organic layers were washed with H_2O (1 x 5mL), brine (1 x 5mL), and dried over $MgSO_4$. The organic layer was filtered and concentrated under reduced pressure to yield the corresponding crude (Z)-enol triflate.[4]

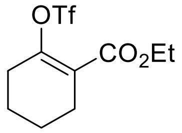

ethyl 2-(((trifluoromethyl)sulfonyl)oxy)cyclohex-1-ene-1-carboxylate (**2a**).[1] Method **A**. On 1.0 mmol scale; purified by flash column chromatography on SiO_2 (eluent: petroleum ether/ethyl acetate) to afford **2a**, 271.8 mg, 90%, light yellow oil; Rf = 0.7 (petroleum ether/ethyl acetate 10:1).^{1}H NMR (400 MHz, Chloroform-*d*) δ 4.28 - 4.21 (m, 2H), 2.47 - 2.42 (m, 2H), 2.39 - 2.35 (m, 2H), 1.79 - 1.73 (m, 2H), 1.69 - 1.62 (m, 2H), 1.32 - 1.27 (m, 3H). ^{13}C NMR (101 MHz, Chloroform-*d*) δ 164.68 , 151.27 , 123.19 , 118.26 (q, J = 319.8 Hz), 61.48 (d, J = 2.4 Hz), 28.40 , 26.07 , 22.17 , 20.93 , 13.84 . ^{19}F NMR (376 MHz, Chloroform-*d*) δ -74.95 .

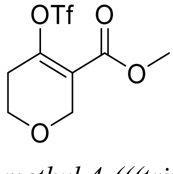

methyl 4-(((trifluoromethyl)sulfonyl)oxy)-5,6-dihydro-2H-pyran-3-carboxylate (**2b**).[3] Method **C**. On 1.0 mmol scale; purified by flash column chromatography on SiO_2 (eluent: petroleum

ether/ethyl acetate) to afford **2b**, 272.6 mg, 94%, light yellow oil; Rf = 0.4 (petroleum ether/ethyl acetate 5:1). ^{1}H NMR (400 MHz, Chloroform-*d*) δ 4.37 (t, *J* = 2.6 Hz, 2H), 3.82 (t, *J* = 5.5 Hz,2H), 3.75 (s, 3H), 2.48 (tt, *J* = 5.5, 2.7 Hz, 2H). ^{13}C NMR (101 MHz, Chloroform-*d*) δ 162.19 , 149.60 , 121.38 , 118.13 (q, *J* = 319.7 Hz), 64.67 , 63.74 , 51.93 , 28.61 . ^{19}F NMR (376 MHz, Chloroform-*d*) δ -75.01 .

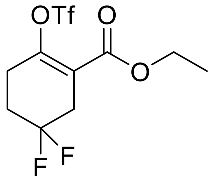

ethyl 5,5-difluoro-2-(((trifluoromethyl)sulfonyl)oxy)cyclohex-1-ene-1-carboxylate (**2c**). Method **C**. On 1.0 mmol scale; purified by flash column chromatography on SiO_2 (eluent: petroleum ether/ethyl acetate) to afford **2c**, 287.3 mg, 85%, light yellow oil; Rf = 0.3 (petroleum ether/ethyl acetate 10:1).^{1}H NMR (400 MHz, Chloroform-*d*) δ 4.27 (q, *J* = 7.1 Hz, 2H), 2.96 m, (3.00-2.92, 2H), 2.66 (m, 2.68-2.64, 2H), 2.24 – 2.14 (m, 2H), 1.31 (t, *J* = 7.1 Hz, 3H). ^{13}C NMR (101 MHz, Chloroform-*d*) δ 162.77 , 150.02 , 120.10 (t, *J* = 240.7 Hz), 118.92 (t, *J* = 6.1 Hz), 118.25 (q, *J* = 319.9 Hz), 62.11 , 34.74 (t, *J* = 29.8 Hz), 29.67 (t, *J* = 25.7 Hz), 26.84 – 26.07 (m), 13.79 . ^{19}F NMR (376 MHz, Chloroform-*d*) δ -74.61 , -98.78 . HRMS (ESI) Calculated for $C_{10}H_{11}F_5O_5S$ $[M+H]^+$: 339.0320, found: 339.0313. IR ν (neat, cm^{-1}): 2990.6, 2918.3, 1725.2, 1426.3, 1377.1, 1298.6, 1209.3, 1137.4, 1078.2, 1037.5, 877.2, 843.5, 623.4.

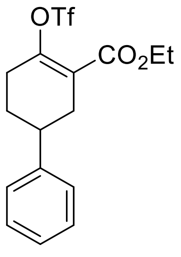

ethyl 4-(((trifluoromethyl)sulfonyl)oxy)-1,2,5,6-tetrahydro-[1,1'-biphenyl]-3-carboxylate (**2d**).[5] Method **A**. On 1.0 mmol scale; purified by flash column chromatography on SiO_2 (eluent: petroleum ether/ethyl acetate) to afford **2d**, 302.4 mg, 80%, light yellow oil; Rf = 0.5 (petroleum ether/ethyl acetate 10:1). ^{1}H NMR (500 MHz, Chloroform-*d*) δ 7.33 – 7.30 (m, 2H), 7.23 – 7.21 (m, 2H), 4.27 (q, *J* = 7.1 Hz, 2H), 2.90 – 2.81 (m, 2H), 2.64 – 2.45 (m, 3H), 2.09 – 2.04 (m, 1H), 1.98 – 1.89 (m, 1H), 1.31 (t, *J* = 7.2 Hz, 3H). ^{13}C NMR (126 MHz, Chloroform-*d*) δ 164.24 , 151.06 , 143.63 , 128.60 , 126.74 , 126.62 , 122.80 , 118.26 (q, *J* = 319.8 Hz), 61.57 , 38.46 , 33.46 , 28.94 , 28.70 , 13.84 . ^{19}F NMR (471 MHz, Chloroform-*d*) δ -74.73

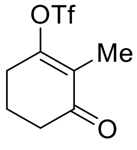

2-methyl-3-oxocyclohex-1-en-1-yl trifluoromethanesulfonate (**2e**).[6] Method **A**. On 1.0 mmol scale; purified by flash column chromatography on SiO_2 (eluent: petroleum ether/ethyl acetate) to afford **2e**, 252.8 mg, 98%, light yellow oil; Rf = 0.5 (petroleum ether/ethyl acetate 10:1). ^{1}H NMR (400 MHz, Chloroform-*d*) δ 2.64 (q, *J* = 6.6, 6.0 Hz, 2H), 2.34 – 2.42 (m, 2H), 2.02 – 1.94 (m, 2H), 1.76 – 1.72 (m, 3H). ^{13}C NMR (101 MHz, Chloroform-*d*) δ 197.22 , 161.92 ,

127.70 , 118.09 (q, J = 319.7 Hz), 36.27 , 28.45 , 20.37 , 8.72 . ^{19}F NMR (376 MHz, Chloroform-*d*) δ -74.98 .

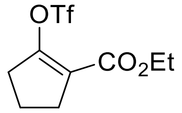

ethyl 2-(((trifluoromethyl)sulfonyl)oxy)cyclopent-1-ene-1-carboxylate (**2f**).[7] Method **A**. On 1.0 mmol scale; purified by flash column chromatography on SiO_2 (eluent: petroleum ether/ethyl acetate) to afford **2f**, 253.4 mg, 88%, light yellow oil; Rf = 0.4 (petroleum ether/ethyl acetate 8:1). ^{1}H NMR (400 MHz, Chloroform-*d*) δ 4.25 – 4.17 (m, 2H), 2.68 (m, 2.62-2.74, 4H), 1.97 (m, 1.92-2.02, 2H), 1.30 – 1.23 (m, 3H). ^{13}C NMR (101 MHz, Chloroform-*d*) δ 162.16 (d, J = 8.6 Hz), 153.39 , 123.32 (d, J = 2.9 Hz), 118.25 (q, J = 320.0, 319.3 Hz), 60.97 (d, J = 9.0 Hz), 32.55 (d, J = 5.8 Hz), 29.13 (d, J = 5.0 Hz), 18.64 (d, J = 6.2 Hz), 13.80 (d, J = 10.0 Hz). ^{19}F NMR (376 MHz, Chloroform-*d*) δ -74.94 .

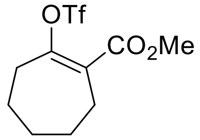

methyl 2-(((trifluoromethyl)sulfonyl)oxy)cyclohept-1-ene-1-carboxylate (**2g**).[8] Method **A**. On 1.0 mmol scale; purified by flash column chromatography on SiO_2 (eluent: petroleum ether/ethyl acetate) to afford **2g**, 253.7 mg, 84%, light yellow oil; Rf = 0.5 (petroleum ether/ethyl acetate 8:1). ^{1}H NMR (400 MHz, Chloroform-*d*) δ 3.76 – 3.73 (m, 3H), 2.58 – 2.49(m, 4H), 1.75 – 1.61 (m, 6H). ^{13}C NMR (101 MHz, Chloroform-*d*) δ 166.00 , 154.96 , 127.73 , 118.26 (q, J = 319.9 Hz), 52.12 , 33.86 , 30.56 , 27.87 , 25.14 , 23.64 . ^{19}F NMR (376 MHz, Chloroform-*d*) δ -75.00 .

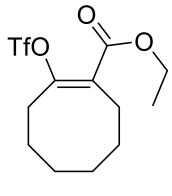

ethyl (Z)-2-(((trifluoromethyl)sulfonyl)oxy)cyclooct-1-ene-1-carboxylate (**2h**).[9] Method **B**. On 1.0 mmol scale; purified by flash column chromatography on SiO_2 (eluent: petroleum ether/ethyl acetate) to afford **2h**, 290.4 mg, 88%, light yellow oil; Rf = 0.5 (petroleum ether/ethyl acetate 8:1).^{1}H NMR (400 MHz, Chloroform-*d*) δ 4.28 – 4.22 (m, 2H), 2.53 – 2.43 (m, 4H), 1.79 – 1.66 (m, 4H), 1.59 – 1.51 (m, 4H), 1.32 – 1.28 (m, 3H). ^{13}C NMR (101 MHz, Chloroform-*d*) δ 165.10 , 152.05 , 125.58 , 118.33 (q, J = 320.1 Hz), 61.51 , 30.93 , 29.46 , 28.37 , 27.82 , 26.03 , 25.51 , 13.88 . ^{19}F NMR (376 MHz, Chloroform-*d*) δ -74.88 .

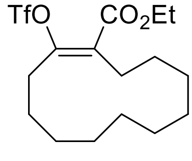

ethyl (Z)-2-(((trifluoromethyl)sulfonyl)oxy)cyclododec-1-ene-1-carboxylate (**2i**). Method **B**. On 1.0 mmol scale; purified by flash column chromatography on SiO_2 (eluent: petroleum

ether/ethyl acetate) to afford **2i**, 308.8 mg, 80%, light yellow oil; Rf = 0.4 (petroleum ether/ethyl acetate 10:1). ^{1}H NMR (400 MHz, Chloroform-*d*) δ 4.21 (q, *J* = 7.2 Hz, 2H), 2.47 – 2.37 (m, 4H), 1.70 – 1.67 (m, 2H), 1.52 – 1.46 (m, 2H), 1.34 (s, 10H), 1.27 (t, *J* = 7.1 Hz, 5H). ^{13}C NMR (101 MHz, Chloroform-*d*) δ 165.45 , 149.96 , 127.70 , 118.17 (q, *J* = 319.8 Hz), 61.45 , 27.40 , 26.62 , 25.18 , 24.28 , 24.23 , 24.10 , 23.60 , 23.53 , 22.24 , 21.92 , 13.70 . ^{19}F NMR (376 MHz, Chloroform-*d*) δ -75.22 . HRMS (ESI) Calculated for $C_{16}H_{25}F_3O_5S$ $[M+H]^+$: 387.1448, found: 387.1436. IR ν (neat, cm^{-1}): 2988.9, 2934.2, 1727.6, 1416.0, 1208.9, 1135.5, 917.5, 844.5, 601.1.

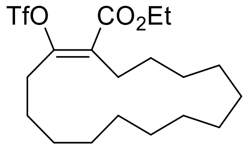

ethyl (Z)-2-(((trifluoromethyl)sulfonyl)oxy)cyclopentadec-1-ene-1-carboxylate (**2j**). Method **B**. On 1.0 mmol scale; purified by flash column chromatography on SiO_2 (eluent: petroleum ether/ethyl acetate) to afford **2j**, 342.4 mg, 80%, light yellow oil; Rf = 0.6 (petroleum ether/ethyl acetate 10:1).^{1}H NMR (400 MHz, Chloroform-*d*) δ 4.24 (q, *J* = 7.1 Hz, 2H), 2.41 – 2.37 (m, 2H), 2.33 – 2.29 (m, 2H), 1.66 – 1.59 (m, 2H), 1.49 – 1.24 (m, 23H). ^{13}C NMR (101 MHz, Chloroform-*d*) δ 165.63 , 150.91 , 126.87 , 118.26 (q, *J* = 319.8 Hz), 61.59 , 30.69 , 29.35 , 27.13 , 27.01(2C) , 26.80 , 26.70 , 26.58 , 26.03 , 25.91 , 25.79 , 25.61 , 25.15 , 13.83 . ^{19}F NMR (376 MHz, Chloroform-*d*) δ -75.07 . HRMS (ESI) Calculated for $C_{19}H_{31}F_3O_5S$ $[M+H]^+$: 429.1917, found: 429.1907. IR ν (neat, cm^{-1}): 2931.4, 2858.4, 1727.8, 1420.5, 1308.8, 1210.4, 926.4, 863.2.

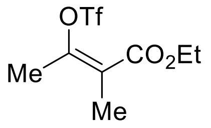

ethyl (Z)-2-methyl-3-(((trifluoromethyl)sulfonyl)oxy)but-2-enoate (**2k**).[4] Method **D**. On 1.0 mmol scale; purified by flash column chromatography on SiO_2 (eluent: petroleum ether/ethyl acetate) to afford **2k**, 248.4 mg, 90%, light yellow oil; Rf = 0.6 (petroleum ether/ethyl acetate 10:1).^{1}H NMR (400 MHz, Chloroform-*d*) δ 4.23 – 4.18 (m, 2H), 2.08 (d, *J* = 1.5 Hz, 3H), 1.92 (t, *J* = 1.4 Hz, 3H), 1.26 (t, *J* = 7.1 Hz, 3H). ^{13}C NMR (101 MHz, Chloroform-*d*) δ 165.21 , 147.54 , 121.75 , 118.23 (q, *J* = 319.7 Hz), 61.54 , 17.41 , 14.98 , 13.67 . ^{19}F NMR (376 MHz, Chloroform-*d*) δ -75.08 .

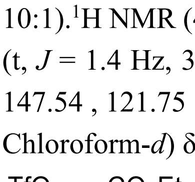

ethyl (Z)-2-phenyl-3-(((trifluoromethyl)sulfonyl)oxy)but-2-enoate (**2l**).[4] Method **D**. On 1.0 mmol scale; purified by flash column chromatography on SiO_2 (eluent: petroleum ether/ethyl acetate) to afford **2l**, 283.9 mg, 84%, light yellow oil; Rf = 0.5 (petroleum ether/ethyl acetate 10:1). ^{1}H NMR (400 MHz, Chloroform-*d*) δ 7.42 – 7.37 (m, 3H), 7.32 – 7.30 (m, 2H), 4.27 (q, *J* = 7.2 Hz, 2H), 2.07 (s, 3H), 1.29 (t, *J* = 7.1 Hz, 3H). ^{13}C NMR (101 MHz, Chloroform-*d*) δ

164.56 , 148.78 , 132.58 , 128.98 , 128.85 , 128.68 , 118.30 (q, *J* = 319.9 Hz), 62.04 , 18.08 , 13.80 . ^{19}F NMR (376 MHz, Chloroform-*d*) δ -74.59 .

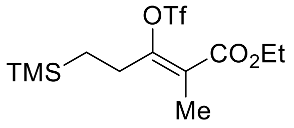

ethyl (Z)-2-methyl-3-(((trifluoromethyl)sulfonyl)oxy)-5-(trimethylsilyl)pent-2-enoate (**2m**).[4] Method **B**. On 1.0 mmol scale; purified by flash column chromatography on SiO_2 (eluent: petroleum ether/ethyl acetate) to afford **2m**, 325.8 mg, 90%, light yellow oil; Rf = 0.7 (petroleum ether/ethyl acetate 10:1). ^{1}H NMR (400 MHz, Chloroform-*d*) δ 4.26 (q, *J* = 7.1 Hz, 2H), 2.39 – 2.35 (m, 2H), 1.96 (s, 3H), 1.32 (t, *J* = 7.1 Hz, 3H), 0.81 – 0.77 (m, 2H), 0.03 (s, 9H). ^{13}C NMR (101 MHz, Chloroform-*d*) δ 165.59 , 153.47 , 120.21 , 118.31 (q, *J* = 320.0 Hz), 61.64 , 25.81 , 14.87 , 13.84 , 13.39 , -2.22 . ^{19}F NMR (376 MHz, Chloroform-*d*) δ -74.95.

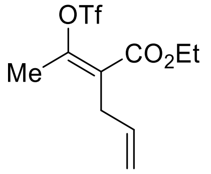

ethyl (Z)-2-(1-(((trifluoromethyl)sulfonyl)oxy)ethylidene)pent-4-enoate (**2n**).[4] Method **D**. On 1.0 mmol scale; purified by flash column chromatography on SiO_2 (eluent: petroleum ether/ethyl acetate) to afford **2n**, 286.9 mg, 95%, light yellow oil; Rf = 0.5 (petroleum ether/ethyl acetate 10:1).^{1}H NMR (400 MHz, Chloroform-*d*) δ 5.83 – 5.73(m, 1H), 5.15 – 5.09 (m, 2H), 4.27 (q, *J* = 7.2 Hz, 2H), 3.11 – 3.08(m, 2H), 2.13 (s, 3H), 1.31 (t, *J* = 7.1 Hz, 3H). ^{13}C NMR (101 MHz, Chloroform-*d*) δ 164.85 , 149.00 , 132.46 , 123.99 , 118.30 (q, *J* = 319.7 Hz), 116.95 , 61.81 , 33.25 , 17.56 , 13.90 . ^{19}F NMR (376 MHz, Chloroform-*d*) δ -74.67 .

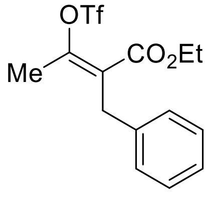

ethyl (Z)-2-benzyl-3-(((trifluoromethyl)sulfonyl)oxy)but-2-enoate (**2o**).[4] Method **D**. On 1.0 mmol scale; purified by flash column chromatography on SiO_2 (eluent: petroleum ether/ethyl acetate) to afford **2o**, 278.1 mg, 79%, light yellow oil; Rf = 0.5 (petroleum ether/ethyl acetate 10:1). ^{1}H NMR (400 MHz, Chloroform-*d*) δ 7.31 – 7.26 (m, 2H), 7.23 – 7.16 (m, 3H), 4.18 (q, *J* = 7.1 Hz, 2H), 3.72 (s, 2H), 2.18 (s, 3H), 1.19 (t, *J* = 7.1 Hz, 3H). ^{13}C NMR (101 MHz, Chloroform-*d*) δ 164.88 , 149.14 , 136.77 , 128.72 , 128.05 , 126.86 , 125.41 , 118.37 (q, *J* = 319.9 Hz), 61.78 , 35.02 , 17.80 , 13.75 . ^{19}F NMR (376 MHz, Chloroform-*d*) δ -74.73 .

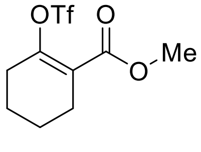

methyl 2-(((trifluoromethyl)sulfonyl)oxy)cyclohex-1-ene-1-carboxylate (**2p**).[3] Method **A**. On 1.0 mmol scale; purified by flash column chromatography on SiO_2 (eluent: petroleum ether/ethyl acetate) to afford **2p**, 267.8 mg, 93%, light yellow oil; Rf = 0.6 (petroleum

ether/ethyl acetate 10:1). ^{1}H NMR (400 MHz, Chloroform-*d*) δ 3.76 (s, 1H), 2.56 – 2.27 (m, 2H), 1.84 – 1.56 (m, 2H). ^{13}C NMR (101 MHz, Chloroform-*d*) δ 164.99 , 151.73 , 122.76 , 118.23 (q, *J* = 319.7 Hz), 51.95 , 28.48 , 25.98 , 22.13 , 20.88 . ^{19}F NMR (376 MHz, Chloroform-*d*) δ -75.01 .

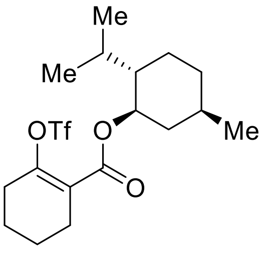

(1R,2S,5R)-2-isopropyl-5-methylcyclohexyl 2-(((trifluoromethyl)sulfonyl)oxy)cyclohex-1-ene-1-carboxylate (**2q**).[3] Method **C**. On 1.0 mmol scale; purified by flash column chromatography on SiO_2 (eluent: petroleum ether/ethyl acetate) to afford **2q**, 329.6 mg, 80%, light yellow oil; Rf = 0.5 (petroleum ether/ethyl acetate 5:1). ^{1}H NMR (500 MHz, Chloroform-*d*) δ 4.82 (td, *J* = 10.9, 4.4 Hz, 1H), 2.51 – 2.37 (m, 4H), 2.06 – 2.01 (m, 1H), 1.91 – 1.85 (m, 1H), 1.80 – 1.74 (m, 2H), 1.71 – 1.63 (m, 4H), 1.54 – 1.46 (m, 2H), 1.11 – 1.01 (m, 2H), 0.90 (t, *J* = 6.8 Hz, 7H), 0.75 (d, *J* = 7.0 Hz, 3H). ^{13}C NMR (126 MHz, Chloroform-*d*) δ 164.11 , 151.26 , 123.47 , 118.31 (q, *J* = 319.8 Hz), 75.78 , 46.52 , 40.44 , 34.16 , 31.44 , 28.48 , 26.23 , 26.03 , 23.26 , 22.21 , 21.97 , 21.03 , 20.75 , 16.09 . ^{19}F NMR (471 MHz, Chloroform-*d*) δ -74.68 .

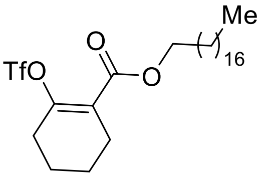

octadecyl 2-(((trifluoromethyl)sulfonyl)oxy)cyclohex-1-ene-1-carboxylate (**2r**). Method **C**. On 1.0 mmol scale; purified by flash column chromatography on SiO_2 (eluent: petroleum ether/ethyl acetate) to afford **2r**, 411.1 mg, 78%, light yellow oil; Rf = 0.5 (petroleum ether/ethyl acetate 10:1). ^{1}H NMR (500 MHz, Chloroform-*d*) δ 4.19 (t, *J* = 6.9 Hz, 2H), 2.49 – 2.45 (m, 2H), 2.41 – 2.37 (m, 2H), 1.80 – 1.75 (m, 2H), 1.71 – 1.64 (m, 4H), 1.37 – 1.23 (s, 32H), 0.87 (t, *J* = 6.9 Hz, 3H). ^{13}C NMR (126 MHz, Chloroform-*d*) δ 164.76 , 151.33 , 123.25 , 118.30 (q, *J* = 319.7 Hz), 65.69 , 31.92 , 29.69 , 29.66 , 29.65 , 29.63 , 29.55 , 29.48 , 29.36 , 29.20 , 28.47 , 28.31 , 26.13 , 25.81 , 22.68 , 22.22 , 21.00 , 14.10 . ^{19}F NMR (471 MHz, Chloroform-*d*) δ -74.76 . HRMS (ESI) Calculated for $C_{26}H_{45}F_3O_5S$ $[M+H]^+$: 527.3013, found: 527.3004. IR ν (neat, cm^{-1}): 2923.7, 2852.7, 1723.9, 1423.2, 1285.2, 1207.2, 1140.9, 1044.6, 905.0, 664.3.

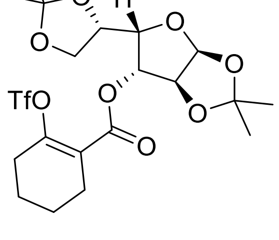

(3aS,5S,6R,6aS)-5-((S)-2,2-dimethyl-1,3-dioxolan-4-yl)-2,2-dimethyltetrahydrofuro[2,3-

d][1,3]dioxol-6-yl 2-(((trifluoromethyl)sulfonyl)oxy)cyclohex-1-ene-1-carboxylate (**2s**). Method **C**. On 1.0 mmol scale; purified by flash column chromatography on SiO_2 (eluent: petroleum ether/ethyl acetate) to afford **2s**, 433.4 mg, 84%, light yellow oil; Rf = 0.3 (petroleum ether/ethyl acetate 4:1). ^{1}H NMR (500 MHz, Chloroform-*d*) δ 5.90 (d, *J* = 3.7 Hz, 1H), 5.37 (d, *J* = 3.1 Hz, 1H), 4.58 (d, *J* = 3.7 Hz, 1H), 4.26 – 4.14 (m, 2H), 4.15 – 4.03 (m, 1H), 3.99 (dd, *J* = 8.6, 4.8 Hz, 1H), 2.56 – 2.36 (m, 4H), 1.85 – 1.60 (m, 4H), 1.50 (s, 3H), 1.39 (s, 3H), 1.30 (s, 3H), 1.29 (s, 3H). ^{13}C NMR (126 MHz, Chloroform-*d*) δ 163.20 , 152.13 , 122.73 , 118.25 (q, *J* = 319.9 Hz), 112.19 , 109.32 , 105.19 , 83.09 , 79.87 , 77.10 , 72.50 , 67.34 , 28.42 , 26.77 , 26.68 , 26.13 , 26.06 , 25.23 , 22.02 , 20.88 . ^{19}F NMR (471 MHz, Chloroform-*d*) δ -74.42 . HRMS (ESI) Calculated for $C_{20}H_{27}F_3O_{10}S$ $[M+Na]^+$: 539.1169, found: 539.1158. IR ν (neat, cm^{-1}): 2990.0, 2940.2, 1734.8, 1423.0, 1375.5, 1248.7, 1140.5, 1043.3, 1019.2, 915.7, 831.5, 612.9.

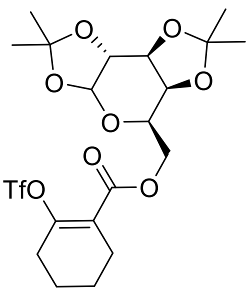

((5R,5aS,8aS,8bR)-2,2,7,7-tetramethyltetrahydro-5H-bis([1,3]dioxolo)[4,5-b:4',5'-d]pyran-5-yl)methyl 2-(((trifluoromethyl)sulfonyl)oxy)cyclohex-1-ene-1-carboxylate (**2t**). Method **C**. On 1.0 mmol scale; purified by flash column chromatography on SiO_2 (eluent: petroleum ether/ethyl acetate) to afford **2t**, 464.4 mg, 90%, light yellow oil; Rf = 0.3 (petroleum ether/ethyl acetate 4:1). ^{1}H NMR (500 MHz, Chloroform-*d*) δ 5.51 (d, *J* = 5.0 Hz, 1H), 4.60 (dd, *J* = 7.9, 2.4 Hz, 1H), 4.32 (ddd, *J* = 12.5, 5.1, 2.7 Hz, 3H), 4.24 (dd, *J* = 7.8, 1.8 Hz, 1H), 4.18 – 4.07 (m, 1H), 2.47 (tt, *J* = 6.0, 3.0 Hz, 2H), 2.37 (td, *J* = 6.1, 2.9 Hz, 2H), 1.75 (qd, *J* = 7.9, 6.3, 4.1 Hz, 2H), 1.69 – 1.59 (m, 2H), 1.49 (s, 3H), 1.43 (s, 3H), 1.31 (d, *J* = 3.8 Hz, 6H). ^{13}C NMR (126 MHz, Chloroform-*d*) δ 164.44 , 151.66 , 122.96 , 118.24 (q, *J* = 319.9 Hz), 109.55 , 108.78 , 96.21 , 70.94 , 70.66 , 70.36 , 65.65 , 64.37 , 28.43 , 26.00 , 25.89 , 25.73 , 24.90 , 24.40 , 22.10 , 20.91 . ^{19}F NMR (471 MHz, Chloroform-*d*) δ -74.79 . HRMS (ESI) Calculated for $C_{20}H_{27}F_3O_{10}S$ $[M+H]^+$: 517.1350, found: 517.1340. IR ν (neat, cm^{-1}): 2989.9, 2940.7, 1726.2, 1666.1, 1460.9, 1421.2, 1250.2, 1206.5, 1138.9, 1068.5, 894.8, 822.4, 656.5.

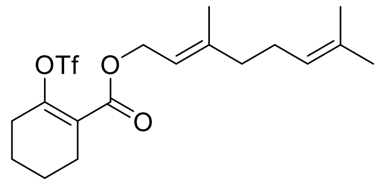

(E)-3,7-dimethylocta-2,6-dien-1-yl 2-(((trifluoromethyl)sulfonyl)oxy)cyclohex-1-ene-1-carboxylate (**2u**). Method **C**. On 1.0 mmol scale; purified by flash column chromatography on SiO_2 (eluent: petroleum ether/ethyl acetate) to afford **2u**, 328.0 mg, 80%, light yellow oil; Rf = 0.6 (petroleum ether/ethyl acetate 10:1). ^{1}H NMR (400 MHz, Chloroform-*d*) δ 5.41 – 5.36 (m, 1H), 5.09 – 5.05 (m, 1H), 4.72 (d, *J* = 7.2 Hz, 2H), 2.49 – 2.44 (m, 2H), 2.41 – 2.36 m, 2H), 2.12 – 2.01 (m, 4H), 1.79 – 1.73 (m, 2H), 1.70 (s, 3H), 1.68 – 1.62 m, 5H), 1.59 (s, 3H). ^{13}C NMR (126 MHz, Chloroform-*d*) δ 164.63 , 151.27 , 142.69 , 131.68 , 123.69 , 123.19 , 118.26

(q, J = 319.7 Hz), 117.72 , 62.26 , 39.47 , 28.44 , 26.16 , 26.09 , 25.55 , 22.18 , 20.95 , 17.55 , 16.36 . ^{19}F NMR (376 MHz, Chloroform-d) δ -74.82 . HRMS (ESI) Calculated for $C_{18}H_{25}F_3O_5S$ [M+Na]$^+$: 433.1267, found: 433.1258. IR ν (neat, cm^{-1}): 2927.3, 2858.1, 1721.9, 1423.0, 1284.4, 1206.0, 1141.4, 1044.1, 973.3, 822.2, 614.5.

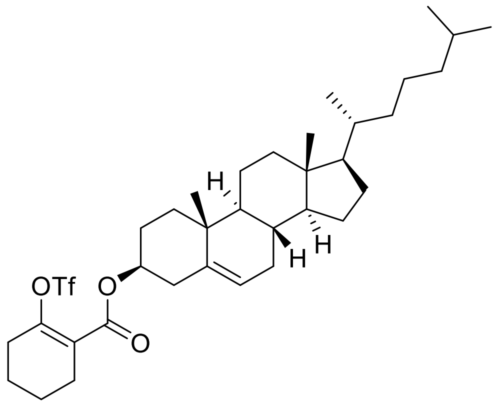

(3S,8S,9S,10R,13R,14S,17R)-10,13-dimethyl-17-((R)-6-methylheptan-2-yl)-2,3,4,7,8,9,10,11,12,13,14,15,16,17-tetradecahydro-1H-cyclopenta[a]phenanthren-3-yl 2-(((trifluoromethyl)sulfonyl)oxy)cyclohex-1-ene-1-carboxylate (**2v**). Method **C**. On 1.0 mmol scale; purified by flash column chromatography on SiO_2 (eluent: petroleum ether/ethyl acetate) to afford **2v**, 455.8 mg, 71%, light yellow oil; Rf = 0.4 (petroleum ether/ethyl acetate 10:1). ^{1}H NMR (500 MHz, Chloroform-d) δ 5.37 (dd, J = 5.1, 2.2 Hz, 1H), 4.79 – 4.72 (m, 1H), 2.48 – 2.45 (m, 2H), 2.40 – 2.37 (m, 2H), 2.02 – 0.94 (m, 35H), 0.91 (d, J = 6.4 Hz, 3H), 0.86 (d, J = 6.8 Hz, 6H), 0.67 (s, 3H). ^{13}C NMR (126 MHz, Chloroform-d) δ 164.12 , 150.96 , 139.53 , 123.61 , 122.70 , 118.29 (q, J = 319.7 Hz), 75.62 , 56.66 , 56.11 , 49.99 , 42.28 , 39.71 , 39.49 , 37.67 , 36.97 , 36.57 , 36.16 , 35.78 , 31.88 , 31.83 , 28.37 , 28.21 , 27.99 , 27.33 , 26.25 , 24.26 , 23.82 , 22.79 , 22.53 , 22.24 , 21.01 , 19.23 , 18.68 , 11.83 . ^{19}F NMR (471 MHz, Chloroform-d) δ -74.63 . HRMS (ESI) Calculated for $C_{35}H_{53}F_3O_5S$ [M+Na]$^+$: 665.3458, found: 665.3454. IR ν (neat, cm^{-1}): 2939.8, 2859.7, 1717.1, 1424.4, 1287.5, 1208.7, 1142.2, 1046.0, 910.5, 613.9.

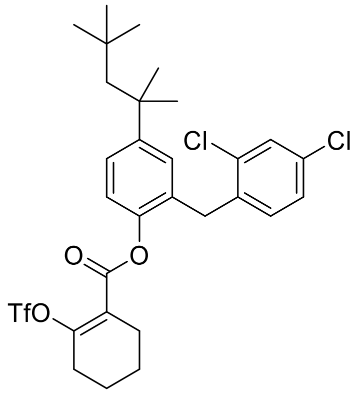

2-(2,4-dichlorobenzyl)-4-(2,4,4-trimethylpentan-2-yl)phenyl 2-(((trifluoromethyl)sulfonyl)oxy)cyclohex-1-ene-1-carboxylate (**2w**). Method **C**. On 1.0 mmol scale; purified by flash column chromatography on SiO_2 (eluent: petroleum ether/ethyl acetate) to afford **2w**, 543.1 mg, 86%, light yellow oil; Rf = 0.5 (petroleum ether/ethyl acetate 5:1). ^{1}H NMR (500 MHz, Chloroform-d) δ 7.38 (d, J = 2.1 Hz, 1H), 7.31 (dd, J = 8.5, 2.4 Hz, 1H), 7.14 – 7.07 (m, 3H), 6.90 (d, J = 8.3 Hz, 1H), 3.97 (s, 2H), 2.46 – 2.41 (m, 4H), 1.83 – 1.78 (m, 2H), 1.74 – 1.61 (m, 4H), 1.33 (s, 6H), 0.71 (s, 9H). ^{13}C NMR (126 MHz, Chloroform-d) δ 162.50 ,

153.01 , 148.29 , 146.12 , 136.30 , 134.50 , 132.46 , 131.29 , 129.34 , 128.88 , 128.76 , 127.05 , 125.75 , 122.26 , 121.67 , 118.26 (q, J = 319.9 Hz), 56.87 , 38.31 , 33.50 , 32.26 , 31.73 , 31.45 , 28.75 , 25.98 , 22.06 , 20.94 . ^{19}F NMR (471 MHz, Chloroform-d) δ -74.50 . HRMS (ESI) Calculated for $C_{29}H_{33}Cl_2F_3O_5S$ $[M+H]^+$: 621.1451, found: 621.1442. IR ν (neat, cm^{-1}): 2956.8, 2921.7, 1744.4, 1665.2, 1501.3, 1212.9, 1140.9, 1044.8, 909.2, 813.8, 613.2.

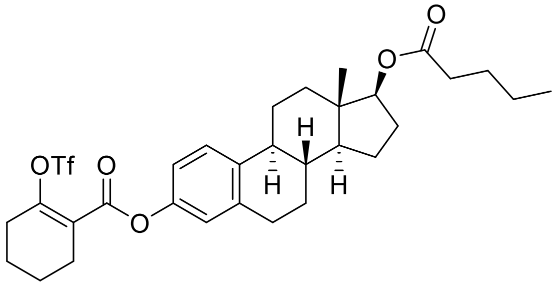

(8R,9S,13S,14S,17S)-13-methyl-17-(pentanoyloxy)-7,8,9,11,12,13,14,15,16,17-decahydro-6H-cyclopenta[a]phenanthren-3-yl 2-(((trifluoromethyl)sulfonyl)oxy)cyclohex-1-ene-1-carboxylate (**2x**). Method **C**. On 1.0 mmol scale; purified by flash column chromatography on SiO_2 (eluent: petroleum ether/ethyl acetate) to afford **2x**, 459.0 mg, 75%, light yellow oil; Rf = 0.3 (petroleum ether/ethyl acetate 10:1). ^{1}H NMR (400 MHz, Chloroform-d) δ 7.28 (d, J = 8.5 Hz, 1H), 6.89 (dd, J = 8.5, 2.6 Hz, 1H), 6.85 (d, J = 2.5 Hz, 1H), 4.70 (dd, J = 9.2, 7.7 Hz, 1H), 2.86 (dd, J = 7.9, 3.4 Hz, 2H), 2.63 – 2.58 (m, 2H), 2.50 – 2.45 (m, 2H), 2.34 – 2.17 (m, 5H), 1.91 – 1.82 (m, 4H), 1.78 – 1.24 (m, 14H), 0.93 (t, J = 7.3 Hz, 3H), 0.83 (s, 3H). ^{13}C NMR (126 MHz, Chloroform-d) δ 173.22, 162.84 , 152.27 , 147.68 , 137.70 , 125.99 , 122.28 , 121.03 , 118.19 , 118.02 (q, J = 320.2 Hz), 81.92 , 49.44 , 43.60 , 42.50 , 37.86 , 36.54 , 33.82 , 29.13 , 28.36 , 27.24 , 26.83 , 26.65 , 25.93 , 25.69 , 22.87 , 21.88 , 21.80 , 20.64 , 13.32 , 11.63 . ^{19}F NMR (376 MHz, Chloroform-d) δ -74.49. HRMS (ESI) Calculated for $C_{31}H_{39}F_3O_7S$ $[M+H]^+$: 613.2441, found: 613.2433. IR ν (neat, cm^{-1}): 2963.0, 2868.0, 1732.0, 1494.1, 1422.9, 1209.2, 1140.0, 1037.3, 920.0, 831.2, 612.6.

2.2 General procedure

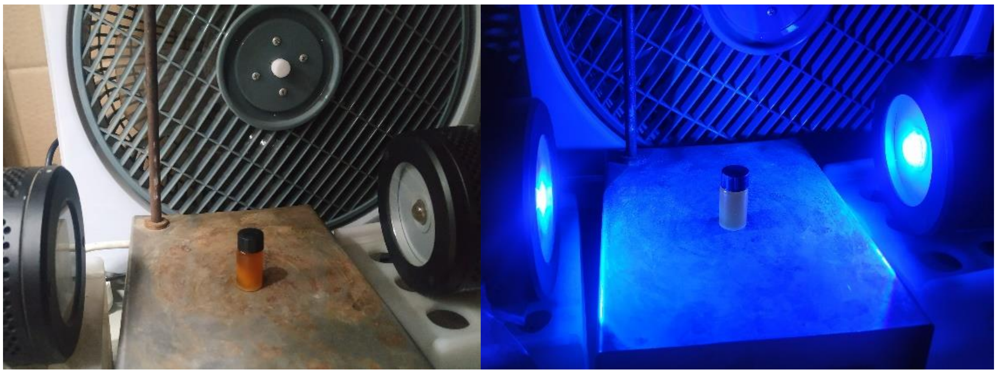

Supplementary Figure 1. Reaction device

General procedure A (Standard conditions)

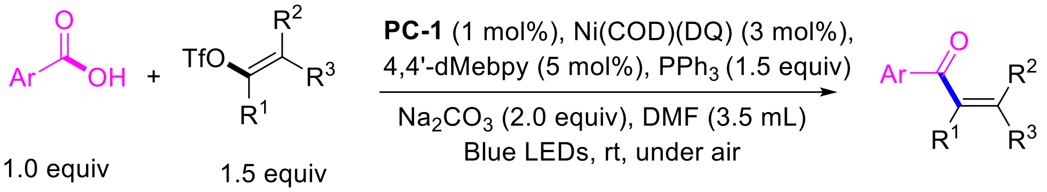

All operations are under air.

A stirring bar, Ni(COD)(DQ) (3.0 mol%), 4,4′-di-methyl-butyl-2,2′-bipyridine (5.0 mol%) and DMF (2.0 mL) were successively added to a vial (2.0 mL). The vial was stirred until the resulting mixture become homogeneous (about 20 min). Photocatalyst Ir[dF(CF_3)ppy]$_2$(dtbbpy)PF_6 (1 mol%), aromatic carboxylic acid (0.2 mmol, 1.0 equiv), triflates (0.3 mmol, 1.5 equiv), Ph_3P (0.3 mmol, 1.5 equiv), and Na_2CO_3 (0.4 mmol, 2.0 equiv) were added to an 3.5 mL screw-cap vial equipped with a magnetic stirring bar. Subsequently, the homogenous solution was syringed into the vial. Then add DMF (1.5 mL) to the vial. The vial was then sealed and placed ~5 cm from 2 × 45 W blue LEDs. The reaction mixture was stirred for 24 h at room temperature (air-condition was used to keep the temperature is 25 °C or so). After completion, the reaction mixture was removed from the light. The solvent was removed and the residue was purified by flash chromatography on silica gel to afford the corresponding products.

General procedure B (Modified standard conditions)

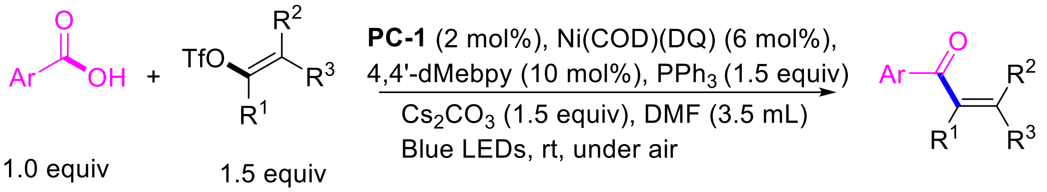

All operations are under air.

A stirring bar, Ni(COD)(DQ) (6.0 mol%), 4,4′-di-methyl-butyl-2,2′-bipyridine (10.0 mol%) and DMF (2.0 mL) were successively added to a vial (2.0 mL). The vial was stirred until the resulting mixture become homogeneous (about 20 min). Photocatalyst Ir[dF(CF_3)ppy]$_2$(dtbbpy)PF_6 (2 mol%), aromatic carboxylic acid (0.2 mmol, 1.0 equiv), triflates (0.3 mmol, 1.5 equiv), Ph_3P (0.3 mmol, 1.5 equiv), and Cs_2CO_3 (0.3 mmol, 1.5 equiv) were added to an 3.5 mL screw-cap vial equipped with a magnetic stirring bar. Subsequently, the homogenous solution was syringed into the vial. Then add DMF (1.5 mL) to the vial. The vial was then sealed and placed ~5 cm from 2 × 45 W blue LEDs. The reaction mixture was stirred for 24 h at room temperature (air-condition was used to keep the temperature is 25 °C or so). After completion, the reaction mixture was removed from the light. The solvent was removed and the residue was purified by flash chromatography on silica gel to afford the corresponding products.

2.3 X-Ray Crystal Data

Compound **13** was crystallized as a colorless crystal via vaporization of a hexane/ethyl acetate solution, and its relative configuration was determined by x-ray structure analaysis. CCDC 2089722 contains the supplementary crystallographic data that can be obtained free of charge from The Cambridge Crystallographic Data Centre via www.ccdc.cam.ac.uk/data_request/cif.

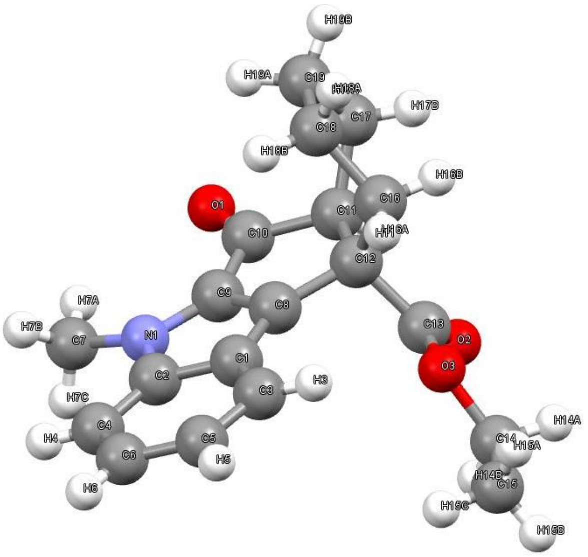

Supplementary Figure 2. X-ray structure of compound **13**

Identification code	CCDC 2089722
Empirical formula	$C_{19}H_{21}NO_3$
Formula weight	311.15
Temperature	193 K
Wavelength	1.34139 Å
Space group	P 2_1/n
Unit cell dimensions	**a** 7.4836(7) **b** 11.4528(12) **c** 18.3368(18); α 90 β 90.591(4) γ 90
Volume	1571.53 Å
Z, Calculated density	4, 1.316 g/cm^3
Absorption coefficient	0.455 mm^{-1}
F(000)	664.0
h,k,l max	9, 14, 23
Data completeness	0.977
Theta(max)	60.561
R(reflections)	0.0532(3038)
wR2(reflections)	0.1436(3524)

Bonds Length:

C1 C2 1.424(2)

C1 C3 1.405(2)

C2	C4	1.400(2)
C12	C16	1.541(2)
C12	C13	1.529(2)
C12	C11	1.561(2)
C9	C10	1.449(2)
C3	H3	0.950
C3	C5	1.382(2)
C16	H16A	0.990
C16	H16B	0.990
C16	C18	1.531(2)
C4	H4	0.950
C4	C6	1.380(2)
C5	H5	0.950
C5	C6	1.404(3)
C10	C11	1.525(2)
C7	H7A	0.980
C7	H7B	0.980
C7	H7C	0.980
C11	H11	1.000
C11	C17	1.521(2)
C6	H6	0.950
C17	H17A	0.990
C17	H17B	0.990
C17	C19	1.524(3)
C18	H18A	0.990
C18	H18B	0.990
C18	C19	1.539(3)
C19	H19A	0.990
C19	H19B	0.990
C14	H14A	0.990
C14	H14B	0.990
C14	C15	1.468(4)
C15	H15A	0.980
C15	H15B	0.980
C15	H15C	0.980

2.4 Characterization of products

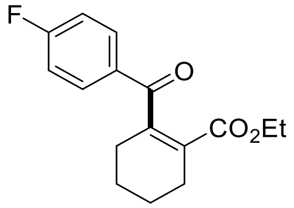

ethyl 2-(4-fluorobenzoyl)cyclohex-1-ene-1-carboxylate (**3a**). The reaction was carried out according to the general procedure **A** on 0.2 mmol scale (24 h); purified by flash column chromatography on SiO_2 (eluent: petroleum ether/ethyl acetate) to afford **3a**, 49.7 mg, 90%, light yellow oil; Rf = 0.5 (petroleum ether/ethyl acetate 10:1). ^{1}H NMR (400 MHz, Chloroform-*d*) δ 7.92 – 7.87 (m, 2H), 7.16 – 7.10 (m, 2H), 3.94 (q, *J* = 7.1 Hz, 2H), 2.47 – 2.44 (m, 2H), 2.36 – 2.33 (m, 2H), 1.81 – 1.72 (m, 4H), 0.98 (t, *J* = 7.1 Hz, 3H). ^{13}C NMR (101 MHz, Chloroform-*d*) δ 197.36 , δ 166.09 , 165.70 (d, *J* = 254.7 Hz), 149.12 , 131.83 (d, *J* = 2.9 Hz), 131.15 (d, *J* = 9.3 Hz), 127.89 , 115.79 (d, *J* = 21.9 Hz), 60.83 , 28.77 , 24.54 , 21.62 , 21.32 , 13.59 . ^{19}F NMR (376 MHz, Chloroform-*d*) δ -105.19. HRMS (ESI) Calculated for $C_{16}H_{17}FO_3$ $[M+Na]^+$: 299.1054, found: 299.1550. IR ν (neat, cm^{-1}): 2938.9, 17118, 1672.0, 1597.4, 1504.3, 1262.5, 1152.6, 1047.9, 847.3, 750.7.

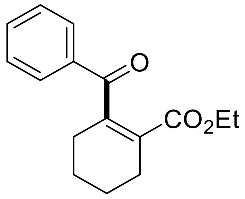

ethyl 2-benzoylcyclohex-1-ene-1-carboxylate (**3b**). The reaction was carried out according to the general procedure **A** on 0.2 mmol scale (24 h); purified by flash column chromatography on SiO_2 (eluent: petroleum ether/ethyl acetate) to afford **3b**, 37.7 mg, 73%, light yellow oil; Rf = 0.5 (petroleum ether/ethyl acetate 10:1). ^{1}H NMR (400 MHz, Chloroform-*d*) δ 7.89 – 7.87 (m, 2H), 7.55 (dd, *J* = 8.6, 6.4 Hz, 1H), 7.45 (t, *J* = 7.8 Hz, 2H), 3.91 (q, *J* = 7.1 Hz, 2H), 2.49 – 2.44 (m, 2H), 2.38 – 2.35 (m, 2H), 1.81 – 1.72 (m, 4H), 0.93 (t, *J* = 7.2 Hz, 3H).^{13}C NMR (101 MHz, Chloroform-*d*) δ 198.80 , 166.14 , 149.31 , 135.31 , 133.05 , 128.60 , 128.57 , 127.73 , 60.74 , 28.79 , 24.54 , 21.63 , 21.34 , 13.48 . HRMS (ESI) Calculated for $C_{16}H_{18}O_3$ $[M+Na]^+$: 281.1148, found: 281.1143. IR ν (neat, cm^{-1}): 2928.5, 1710.4, 1669.7, 1596.3, 1504.0, 1245.0, 1151.8, 1064.1, 849.3, 747.8, 700.8, 603.2.

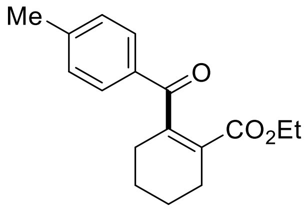

ethyl 2-(4-methylbenzoyl)cyclohex-1-ene-1-carboxylate (**3c**). The reaction was carried out according to the general procedure **A** on 0.2 mmol scale (24 h); purified by flash column chromatography on SiO_2 (eluent: petroleum ether/ethyl acetate) to afford **3c**, 34.3 mg, 63%,

light yellow oil; Rf = 0.5 (petroleum ether/ethyl acetate 10:1). ^{1}H NMR (400 MHz, Chloroform-*d*) δ 7.79 – 7.75 (m, 2H), 7.26 – 7.24 (m, 2H), 3.91 (q, *J* = 7.1 Hz, 2H), 2.47 – 2.43 (m, 2H), 2.41 (s, 3H), 2.37 – 2.33 (m, 2H), 1.82 – 1.70 (m, 4H), 0.95 (t, *J* = 7.1 Hz, 3H). ^{13}C NMR (101 MHz, Chloroform-*d*) δ 198.59 , 166.21 , 149.50 , 143.87 , 132.89 , 129.32 , 128.72 , 127.44 , 60.70 , 28.86 , 24.56 , 21.67 , 21.37 , 13.53 . HRMS (ESI) Calculated for $C_{17}H_{20}O_3$ $[M+Na]^+$: 295.1305, found: ,295.1300. IR ν (neat, cm^{-1}): 2936.6, 1711.4, 1668.2, 1605.7, 1263.1, 1177.9, 1047.7, 926.3, 829.8, 739.0.

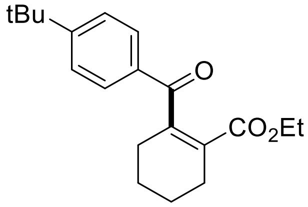

ethyl 2-(4-(tert-butyl)benzoyl)cyclohex-1-ene-1-carboxylate (**3d**). The reaction was carried out according to the general procedure **A** on 0.2 mmol scale (24 h); purified by flash column chromatography on SiO_2 (eluent: petroleum ether/ethyl acetate) to afford **3d**, 40.9 mg, 65%, light yellow oil; Rf = 0.5 (petroleum ether/ethyl acetate 10:1). ^{1}H NMR (400 MHz, Chloroform-*d*) δ 7.82 – 7.79 (m, 2H), 7.48 – 7.45 (m, 2H), 3.92 (q, *J* = 7.1 Hz, 2H), 2.48 – 2.43 (m, 2H), 2.38 – 2.33 (m, 2H), 1.81 – 1.71 (m, 4H), 1.33 (s, 9H), 0.93 (t, *J* = 7.1 Hz, 3H). ^{13}C NMR (101 MHz, Chloroform-*d*) δ 198.64 , 166.27 , 156.80 , 149.63 , 132.77 , 128.56 , 127.44 , 125.58 , 60.71 , 35.11 , 31.07 , 28.88 , 24.58 , 21.70 , 21.39 , 13.49 . HRMS (ESI) Calculated for $C_{20}H_{26}O_3$ $[M+Na]^+$: 377.1774, found: 377.1770. IR ν (neat, cm^{-1}): 2938.5, 1712.2, 1670.8, 1605.4, 1367.3, 1265.8, 1243.0, 1144.8, 1048.0, 927.7, 846.3, 697.2.

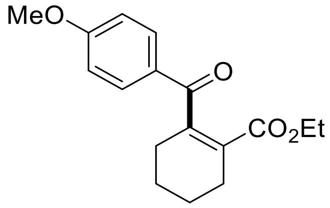

ethyl 2-(4-methoxybenzoyl)cyclohex-1-ene-1-carboxylate (**3e**). The reaction was carried out according to the general procedure **A** on 0.2 mmol scale (24 h); purified by flash column chromatography on SiO_2 (eluent: petroleum ether/ethyl acetate) to afford **3e**, 36.3 mg, 63%, light yellow oil; Rf = 0.2 (petroleum ether/ethyl acetate 10:1). ^{1}H NMR (400 MHz, Chloroform-*d*) δ 7.88 – 7.82 (m, 1H), 6.99 – 6.89 (m, 1H), 3.92 (q, *J* = 7.1 Hz, 1H), 3.87 (s, 2H), 2.50 – 2.40 (m, 1H), 2.37 – 2.33 (m, 1H), 1.85 – 1.69 (m, 3H), 0.95 (t, *J* = 7.1 Hz, 2H). ^{13}C NMR (101 MHz, Chloroform-*d*) δ 197.70 , 166.28 , 163.51 , 149.46 , 130.88 , 128.50 , 127.29 , 113.86 , 60.70 , 55.44 , 28.93 , 24.61 , 21.70 , 21.41 , 13.57 . HRMS (ESI) Calculated for $C_{17}H_{20}O_4$ $[M+Na]^+$: 311.1254, found: 311.1251. IR ν (neat, cm^{-1}): 2936.8, 1711.3, 1663.7, 1599.7, 1508.7, 1252.8, 1169.2, 1047.9, 842.6.

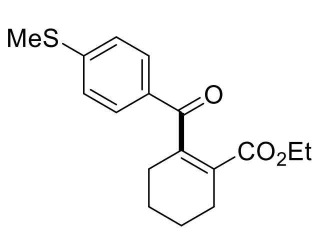

ethyl 2-(4-(methylthio)benzoyl)cyclohex-1-ene-1-carboxylate (**3f**). The reaction was carried out according to the general procedure **A** on 0.2 mmol scale (24 h); purified by flash column chromatography on SiO_2 (eluent: petroleum ether/ethyl acetate) to afford **3f**, 39.5 mg, 65%, light yellow oil; Rf = 0.3 (petroleum ether/ethyl acetate 10:1). ^{1}H NMR (400 MHz, Chloroform-*d*) δ 7.80 – 7.76 (m, 2H), 7.27 – 7.24 (m, 2H), 3.93 (q, *J* = 7.1 Hz, 2H), 2.51 (s, 3H), 2.47 – 2.43 (m, 2H), 2.36 – 2.32 (m, 2H), 1.81 – 1.70 (m, 3H), 0.97 (t, *J* = 7.1 Hz, 3H). ^{13}C NMR (101 MHz, Chloroform-*d*) δ 198.01 , 166.15 , 149.29 , 145.85 , 131.72 , 128.94 , 127.57 , 125.03 , 60.74 , 28.83 , 24.54 , 21.63 , 21.34 , 14.71 , 13.57 . HRMS (ESI) Calculated for $C_{17}H_{20}O_3S$ $[M+Na]^+$: 327.1025, found: 327.1021. IR ν (neat, cm^{-1}): 2935.9, 1710.1, 1666.3, 1588.5, 1263.0, 1244.4, 1181.2, 1089.9, 1047.6, 926.0, 833.9, 743.4.

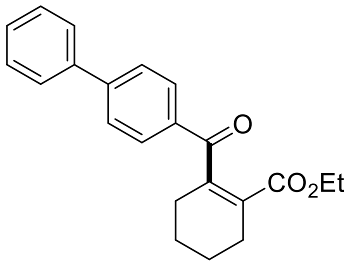

ethyl 2-([1,1'-biphenyl]-4-carbonyl)cyclohex-1-ene-1-carboxylate (**3g**). The reaction was carried out according to the general procedure **A** on 0.2 mmol scale (24 h); purified by flash column chromatography on SiO_2 (eluent: petroleum ether/ethyl acetate) to afford **3g**, 50.2 mg, 75%, colorless oil; Rf = 0.4 (petroleum ether/ethyl acetate 10:1). ^{1}H NMR (400 MHz, Chloroform-*d*) δ 7.97 – 7.94 (m, 2H), 7.69 – 7.66 (m, 2H), 7.63 – 7.60 (m, 2H), 7.49 – 7.44 (m, 2H), 7.41 – 7.37 (m, 1H), 3.95 (q, *J* = 7.1 Hz, 2H), 2.50 – 2.46 (m, 2H), 2.41 – 2.38 (m, 2H), 1.83 – 1.74 (m, 4H), 0.98 (t, *J* = 7.1 Hz, 3H). ^{13}C NMR (101 MHz, Chloroform-*d*) δ 198.52, 166.19, 149.49, 145.72, 139.89, 134.07, 129.14, 128.88, 128.14, 127.69, 127.29, 127.21, 60.78, 28.84, 24.55, 21.64, 21.35, 13.56. HRMS (ESI) Calculated for $C_{22}H_{22}O_3$ $[M+Na]^+$: 357.1461, found: 357.1458. IR ν (neat, cm^{-1}): 2936.2, 17.9.3, 1669.7, 1602.7, 1261.4, 1243.0, 1177.8, 1047.7, 926.9, 741.8, 696.6.

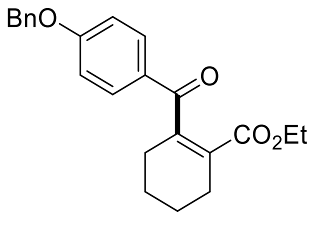

ethyl 2-(4-(benzyloxy)benzoyl)cyclohex-1-ene-1-carboxylate (**3h**). The reaction was carried out according to the general procedure **A** on 0.2 mmol scale (24 h); purified by flash column chromatography on SiO_2 (eluent: petroleum ether/ethyl acetate) to afford **3h**, 46.6 mg, 64%, light yellow solid; mp = 77-79 °C, Rf = 0.2 (petroleum ether/ethyl acetate 10:1). ^{1}H NMR (400 MHz, Chloroform-*d*) δ 7.82 – 7.80 (m, 2H), 7.44 – 7.31 (m, 5H), 7.01 – 6.98 (m, 2H), 5.12 (s, 2H), 3.92 (q, *J* = 7.1 Hz, 2H), 2.46 – 2.43 (m, 2H), 2.37 – 2.33 (m, 2H), 1.80 – 1.70 (m, 4H), 0.95 (t, *J* = 7.2 Hz, 3H). ^{13}C NMR (101 MHz, Chloroform-*d*) δ 197.61 , 166.20 , 162.60 , 149.42 , 136.10 , 130.82 , 128.60 , 128.15 , 127.40 , 127.26 , 114.67 , 70.07 , 60.64 , 28.87 , 24.55 , 21.64 , 21.35 , 13.53 . HRMS (ESI) Calculated for $C_{23}H_{24}O_4$ $[M+H]^+$: 365.1747, found: 365.1744. IR ν (neat, cm^{-1}): 2935.7, 1708.9, 1662.3, 1598.1, 1241.2, 1168.1, 1047.4, 841.1,

752.4, 735.0, 696.7.

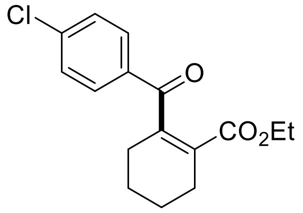

ethyl 2-(4-chlorobenzoyl)cyclohex-1-ene-1-carboxylate (**3i**). The reaction was carried out according to the general procedure **A** on 0.2 mmol scale (24 h); purified by flash column chromatography on SiO_2 (eluent: petroleum ether/ethyl acetate) to afford **3i**, 43.4 mg, 74%, colorless oil; Rf = 0.3 (petroleum ether/ethyl acetate 10:1). ^{1}H NMR (400 MHz, Chloroform-*d*) δ 7.81 (dd, *J* = 8.6, 1.8 Hz, 2H), 7.42 (dd, *J* = 8.5, 1.8 Hz, 2H), 3.94 (q, *J* = 7.1 Hz, 2H), 2.47 – 2.43 (m, 2H), 2.35 – 2.32 (m, 2H), 1.81 – 1.72 (m, 4H), 0.99 (t, *J* = 7.1, 3H). ^{13}C NMR (101 MHz, Chloroform-*d*) δ 197.63 , 166.01 , 149.00 , 139.40 , 133.75 , 129.90 , 128.96 , 128.05 , 60.85 , 28.71 , 24.48 , 21.57 , 21.27 , 13.60 . HRMS (ESI) Calculated for $C_{16}H_{17}ClO_3$ $[M+H]^+$: 293.0939, found: 293.0936. IR ν (neat, cm^{-1}): 2937.2, 1710.8, 1674.2, 1587.0, 1279.1, 1259.5, 1090.3, 1047.8, 1012.8, 925.8, 840.9, 742.3.

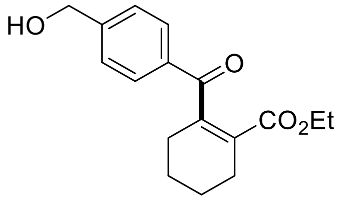

ethyl 2-(4-(hydroxymethyl)benzoyl)cyclohex-1-ene-1-carboxylate (**3j**). The reaction was carried out according to the general procedure **A** on 0.2 mmol scale (24 h); purified by flash column chromatography on SiO_2 (eluent: petroleum ether/ethyl acetate) to afford **3j**, 36.9 mg, 64%, colorless oil; Rf = 0.3 (petroleum ether/ethyl acetate 2:1). ^{1}H NMR (400 MHz, Chloroform-*d*) δ 7.87 – 7.83 (m, 2H), 7.44 – 7.42 (m, 2H), 4.75 (s, 2H), 3.90 (q, *J* = 7.1 Hz, 2H), 2.46 – 2.43 (m, 2H), 2.36 – 2.31 (m, 2H), 2.12 (s, 1H), 1.80 – 1.71 (m, 4H), 0.95 (t, *J* = 7.1 Hz, 3H). ^{13}C NMR (101 MHz, Chloroform-*d*) δ 198.63 , 166.18 , 149.47 , 146.30 , 134.49 , 128.85 , 127.66 , 126.68 , 64.55 , 60.78 , 28.81 , 24.51 , 21.61 , 21.32 , 13.56 . HRMS (ESI) Calculated for $C_{17}H_{20}O_4$ $[M+Na]^+$: 311.1254, found: 311.1251. IR ν (neat, cm^{-1}): 3436.4, 2934.2, 1710.0, 1667.8, 1607.2, 1265.0, 1244.7, 1047.8, 927.4, 737.5.

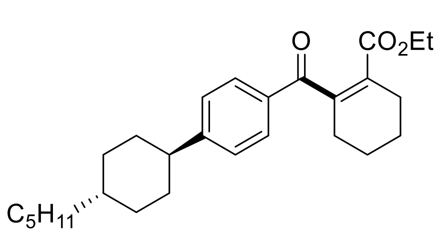

ethyl 2-(4-((1s,4r)-4-pentylcyclohexyl)benzoyl)cyclohex-1-ene-1-carboxylate (**3k**). The reaction was carried out according to the general procedure **A** on 0.2 mmol scale (24 h); purified by flash column chromatography on SiO_2 (eluent: petroleum ether/ethyl acetate) to afford **3k**, 41.9 mg, 51%, light yellow oil; Rf = 0.4 (petroleum ether/ethyl acetate 10:1). ^{1}H NMR (400 MHz, Chloroform-*d*) δ 7.80 – 7.78 (m, 2H), 7.31 – 7.24 (m, 2H), 3.91 (q, *J* = 7.2, 2H), 2.55 – 2.43 (m, 3H), 2.37 – 2.34 (m, 2H), 1.88 (d, *J* = 11.8 Hz, 4H), 1.75 (t, *J* = 6.7 Hz, 4H), 1.50 –

1.39 (m, 2H), 1.36 – 1.20 (m, 10H), 1.10 – 1.00 (m, 2H), 0.94 – 0.88 (m, 5H). ^{13}C NMR (101 MHz, Chloroform-*d*) δ 198.61 , 166.23 , 153.59 , 149.60 , 133.18 , 128.78 , 127.38 , 127.11 , 60.67 , 44.75 , 37.27 , 37.19 , 33.97 , 33.38 , 32.15 , 28.86 , 26.58 , 24.56 , 22.67 , 21.67 , 21.37 , 14.07 , 13.47 . HRMS (ESI) Calculated for $C_{27}H_{38}O_3$ $[M+H]^+$: 411.2894, found: 411.2889. IR ν (neat, cm^{-1}): 2920.9, 2851.6, 1712.8,, 1670.5, 1605.1, 1447.6, 1277.8, 1263.6, 1242.3, 1178.8, 1143.4, 1048.1, 926.9, 697.9.

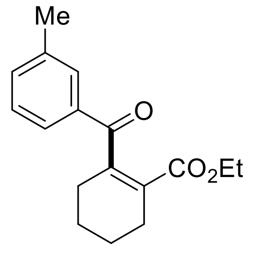

ethyl 2-(3-methylbenzoyl)cyclohex-1-ene-1-carboxylate (**3l**). The reaction was carried out according to the general procedure **A** on 0.2 mmol scale (24 h); purified by flash column chromatography on SiO_2 (eluent: petroleum ether/ethyl acetate) to afford **3l**, 37.1 mg, 67%, light yellow oil; Rf = 0.3 (petroleum ether/ethyl acetate 10:1). ^{1}H NMR (400 MHz, Chloroform-*d*) δ 7.71 (d, *J* = 1.9 Hz, 1H), 7.64 – 7.61 (m, 1H), 7.37 – 7.30 (m, 2H), 3.91 (q, *J* = 7.1 Hz, 2H), 2.47 – 2.43 (m, 2H), 2.39 (s, 3H), 2.37 – 2.32 (m, 2H), 1.81 – 1.71 (m, 4H), 0.94 (t, *J* = 7.2 Hz, 3H). ^{13}C NMR (101 MHz, Chloroform-*d*) δ 199.04 , 166.18 , 149.55 , 138.42 , 135.29 , 133.88 , 128.81 , 128.45 , 127.54 , 126.02 , 60.72 , 28.85 , 24.53 , 21.64 , 21.34 , 13.52 . HRMS (ESI) Calculated for $C_{17}H_{20}O_3$ $[M+Na]^+$: 295.1305, found: 295.1302. IR ν (neat, cm^{-1}): 2936.6, 1712.2, 1670.7, 1270.1, 1245.3, 1195.6, 1048.6, 727.8.

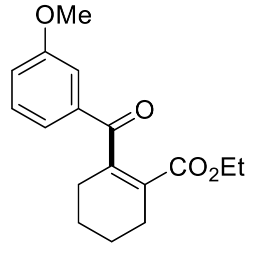

ethyl 2-(3-methoxybenzoyl)cyclohex-1-ene-1-carboxylate (**3m**). The reaction was carried out according to the general procedure **A** on 0.2 mmol scale (24 h); purified by flash column chromatography on SiO_2 (eluent: petroleum ether/ethyl acetate) to afford **3m**, 42.7 mg, 74%, light yellow oil; Rf = 0.2 (petroleum ether/ethyl acetate 10:1). ^{1}H NMR (400 MHz, Chloroform-*d*) δ 7.47 (dd, *J* = 2.7, 1.5 Hz, 1H), 7.40 – 7.31 (m, 2H), 7.10 – 7.07 m, 1H), 3.92 (q, *J* = 7.1 Hz, 2H), 3.84 (s, 3H), 2.47 – 2.42 (m, 2H), 2.37 – 2.33 (m, 2H), 1.80 – 1.71 (m, 4H), 0.96 (t, *J* = 7.1 Hz, 3H). ^{13}C NMR (101 MHz, Chloroform-*d*) δ 198.59 , 166.12 , 159.87 , 149.41 , 136.68 , 129.59 , 127.72 , 121.52 , 119.59 , 112.34 , 60.75 , 55.38 , 28.85 , 24.51 , 21.62 , 21.33 , 13.57 . HRMS (ESI) Calculated for $C_{17}H_{20}O_4$ $[M+Na]^+$: 311.1254, found: 311.1250. IR ν (neat, cm^{-1}): 2937.4, 1711.5, 1671.6, 1484.8, 1432.3, 1271.6, 1247.3, 1047.4, 736.2.

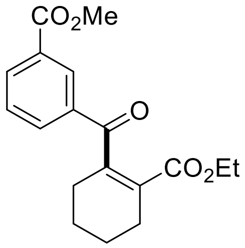

methyl 3-(2-(ethoxycarbonyl)cyclohex-1-ene-1-carbonyl)benzoate (**3n**). The reaction was carried out according to the general procedure **A** on 0.2 mmol scale (24 h); purified by flash column chromatography on SiO_2 (eluent: petroleum ether/ethyl acetate) to afford **3n**, 23.4 mg, 37%, light yellow oil; Rf = 0.2 (petroleum ether/ethyl acetate 10:1). ^{1}H NMR (400 MHz, Chloroform-*d*) δ 8.49 – 8.48 (m, 1H), 8.23 – 8.20 (m, 1H), 8.08 – 8.06 (m, 1H), 7.57 – 7.53 (m, 1H), 3.96-3.90 (m, 6H), 2.49 – 2.45 (m, 2H), 2.37 – 2.34 (m, 2H), 1.82 – 1.73 (m, 4H), 0.96 (t, *J* = 7.2 Hz, 3H). ^{13}C NMR (101 MHz, Chloroform-*d*) δ 198.03 , 166.27 , 166.04 , 149.15 , 135.75 , 133.80 , 132.66 , 130.81 , 129.61 , 128.88 , 128.27 , 60.88 , 52.36, 28.76 , 24.51 , 21.60 , 21.30 , 13.62 . HRMS (ESI) Calculated for $C_{18}H_{20}O_5$ $[M+Na]^+$: 339.1203, found: 339.1199. IR ν (neat, cm^{-1}): 2922.4, 2851.2, 1711.5, 1675.1, 1433.1, 1255.8, 1047.8, 719.4.

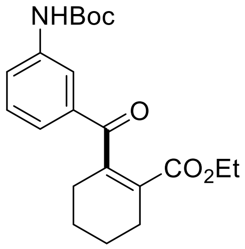

ethyl 2-(3-((tert-butoxycarbonyl)amino)benzoyl)cyclohex-1-ene-1-carboxylate (**3o**). The reaction was carried out according to the general procedure **A** on 0.2 mmol scale (24 h); purified by flash column chromatography on SiO_2 (eluent: petroleum ether/ethyl acetate) to afford **3o**, 57.4 mg, 77%, light yellow solid; mp = 123-125 °C, Rf = 0.2 (petroleum ether/ethyl acetate 10:1). ^{1}H NMR (400 MHz, Chloroform-*d*) δ 7.78 (d, *J* = 7.9 Hz, 1H), 7.70 (t, *J* = 1.9 Hz, 1H), 7.47 (dt, *J* = 7.7, 1.3 Hz, 1H), 7.35 (t, *J* = 7.9 Hz, 1H), 6.74 (s, 1H), 3.91 (q, *J* = 7.1 Hz, 2H), 2.45 – 2.41 (m, 2H), 2.35 – 2.32 (m, 2H), 1.79 – 1.70 (m, 4H), 1.50 (s, 9H), 0.95 (t, *J* = 7.1 Hz, 3H). ^{13}C NMR (101 MHz, Chloroform-*d*) δ 198.47 , 166.20 , 152.61 , 149.40 , 139.07 , 136.04 , 129.37 , 127.81 , 123.29 , 123.18 , 118.09 , 60.85 , 28.84 , 28.31 , 24.56 , 21.65 , 21.35 , 13.61 . HRMS (ESI) Calculated for $C_{21}H_{27}NO_5$ $[M+Na]^+$: 396.1781, found: 396.1777. IR ν (neat, cm^{-1}): 3342.7, 2978.6, 2931.2, 1711.8, 1537.5, 1279.7, 1240.4, 1158.2, 1049.6, 734.2.

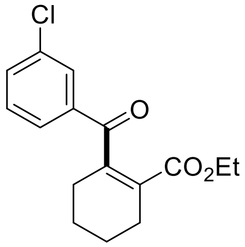

ethyl 2-(3-chlorobenzoyl)cyclohex-1-ene-1-carboxylate (**3p**). The reaction was carried out according to the general procedure **A** on 0.2 mmol scale (24 h); purified by flash column

chromatography on SiO_2 (eluent: petroleum ether/ethyl acetate) to afford **3p**, 29.3 mg, 50%, colorless oil; Rf = 0.3 (petroleum ether/ethyl acetate 10:1). ^{1}H NMR (400 MHz, Chloroform-*d*) δ 7.85 – 7.82 (m, 1H), 7.74 – 7.72 (m, 1H), 7.53 – 7.48 (m, 1H), 7.39 (t, *J* = 7.8 Hz, 1H), 3.95 (q, *J* = 7.1, 2H), 2.47 – 2.44 (m, 2H), 2.35 – 2.32 (m, 2H), 1.81 – 1.72 (m, 4H), 0.99 (t, *J* = 7.1, 3H). ^{13}C NMR (101 MHz, Chloroform-*d*) δ 197.52 , 166.01 , 148.94 , 137.04 , 134.97 , 132.97 , 129.99 , 128.42 , 128.32 , 126.69 , 60.93 , 28.72 , 24.50 , 21.58 , 21.28 , 13.63 . HRMS (ESI) Calculated for $C_{16}H_{17}ClO_3$ $[M+Na]^+$: 315.0758, found: 315.0756. IR ν (neat, cm^{-1}): 2936.5, 1709.9, 1675.3, 1278.4, 1254.9, 1239.0, 1048.6, 731.0.

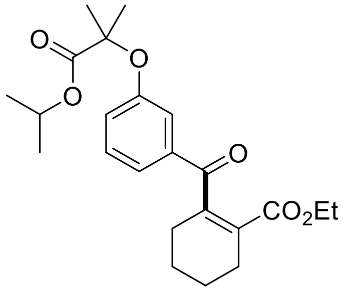

ethyl 2-(3-((1-isopropoxy-2-methyl-1-oxopropan-2-yl)oxy)benzoyl)cyclohex-1-ene-1-carboxylate (**3q**). The reaction was carried out according to the general procedure **A** on 0.2 mmol scale (24 h); purified by flash column chromatography on SiO_2 (eluent: petroleum ether/ethyl acetate) to afford **3q**, 48.2 mg, 60%, colorless oil; Rf = 0.2 (petroleum ether/ethyl acetate 10:1).

^{1}H NMR (400 MHz, Chloroform-*d*) δ 7.78 – 7.74 (m, 2H), 6.83 – 6.78 (m, 2H), 5.10 – 5.03 (m, 1H), 3.89 (q, *J* = 7.1 Hz, 2H), 2.44 – 2.41 (m, 2H), 2.35 – 2.30 (m, 2H), 1.78 – 1.69 (m, 4H), 1.62 (s, 6H), 1.18 (d, *J* = 6.3 Hz, 6H), 0.91 (t, *J* = 7.1 Hz, 3H). ^{13}C NMR (101 MHz, Chloroform-*d*) δ 197.62 , 173.07 , 166.20 , 159.82 , 149.29 , 130.37 , 128.87 , 127.33 , 117.41 , 79.29 , 69.22 , 60.65 , 28.87 , 25.30 , 24.59 , 21.66 , 21.46 , 21.38 , 13.49 . HRMS (ESI) Calculated for $C_{23}H_{30}O_6$ $[M+H]^+$: 403.2115, found: 403.2115. IR ν (neat, cm^{-1}): 2982.7, 2936.8, 1713.3, 1666.9, 1598.5, 1279.6, 1244.7, 1147.2, 1102.8, 927.8.

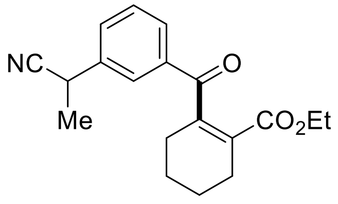

ethyl 2-(3-(1-cyanoethyl)benzoyl)cyclohex-1-ene-1-carboxylate (**3r**). The reaction was carried out according to the general procedure **A** on 0.2 mmol scale (24 h); purified by flash column chromatography on SiO_2 (eluent: petroleum ether/ethyl acetate) to afford **3r**, 47.9 mg, 77%, colorless oil; Rf = 0.3 (petroleum ether/ethyl acetate 4:1). ^{1}H NMR (400 MHz, Chloroform-*d*) δ 7.91 – 7.90 (m, 1H), 7.76 (d, *J* = 7.8 Hz, 1H), 7.60 – 7.58 (m, 1H), 7.48 (t, *J* = 7.7 Hz, 1H), 4.00 – 3.90 (m, 3H), 2.48 – 2.44 (m, 2H), 2.36 – 2.33 (m, 2H), 1.80 – 1.73 (m, 4H), 1.66 (d, *J* = 7.3 Hz, 3H), 0.97 (t, *J* = 7.1 Hz, 3H). ^{13}C NMR (101 MHz, Chloroform-*d*) δ 198.08 , 166.05 , 149.04 , 137.79 , 136.11 , 131.15 , 129.54 , 128.54 , 128.21 , 126.43 , 120.98 , 60.81 , 31.06 , 28.70 , 24.47 , 21.55 , 21.26 , 21.19 , 13.56 . HRMS (ESI) Calculated for $C_{19}H_{21}NO_3$ $[M+H]^+$: 312.1594, found: 312.1591. IR ν (neat, cm^{-1}): 2983.6, 2938.0, 1709.4, 1672.1, 1277.8, 1245.6, 1048.4, 732.4, 695.5.

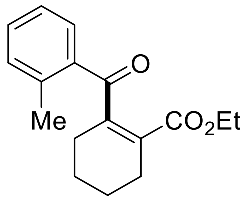

ethyl 2-(2-methylbenzoyl)cyclohex-1-ene-1-carboxylate (**3s**). The reaction was carried out according to the general procedure **A** on 0.2 mmol scale (24 h); purified by flash column chromatography on SiO_2 (eluent: petroleum ether/ethyl acetate) to afford **3s**, 34.3 mg, 63%, light yellow oil; Rf = 0.5 (petroleum ether/ethyl acetate 10:1). ^{1}H NMR (400 MHz, Chloroform-*d*) δ 7.58 (dd, *J* = 7.7, 1.4 Hz, 1H), 7.37 (td, *J* = 7.5, 1.5 Hz, 1H), 7.27 – 7.19 (m, 2H), 3.91 (q, *J* = 7.1 Hz, 2H), 2.66 (s, 3H), 2.46-2.36 (s, 4H), 1.80 – 1.63 (m, 4H), 0.97 (t, *J* = 7.1 Hz, 3H). ^{13}C NMR (101 MHz, Chloroform-*d*) δ 200.52, 166.68, 149.97, 140.39, 134.50, 132.21, 131.92, 131.08, 128.26, 125.53, 60.68, 28.89, 24.97, 21.97, 21.68, 21.49, 13.64. HRMS (ESI) Calculated for $C_{17}H_{20}O_3$ $[M+Na]^+$: 295.1305, found: 295.1300. IR ν (neat, cm^{-1}): 2936.6, 1712.2, 1670.7, 1270.1, 1245.3, 1048.6, 727.8.

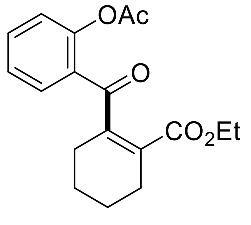

ethyl 2-(2-acetoxybenzoyl)cyclohex-1-ene-1-carboxylate (**3t**). The reaction was carried out according to the general procedure **A** on 0.2 mmol scale (24 h); purified by flash column chromatography on SiO_2 (eluent: petroleum ether/ethyl acetate) to afford **3t**, 44.2 mg, 70%, light yellow oil; Rf = 0.3 (petroleum ether/ethyl acetate 4:1). ^{1}H NMR (400 MHz, Chloroform-*d*) δ 7.75 (dd, *J* = 7.8, 1.7 Hz, 1H), 7.55 (td, *J* = 7.8, 1.7 Hz, 1H), 7.30 (td, *J* = 7.6, 1.2 Hz, 1H), 7.12 (dd, *J* = 8.1, 1.2 Hz, 1H), 3.97 (q, *J* = 7.1 Hz, 2H), 2.45 – 2.41 (m, 3H), 2.35 (s, 3H), 2.34 – 2.31 (m, 2H), 1.78 – 1.69 (m, 4H), 1.00 (t, *J* = 7.1 Hz, 3H). ^{13}C NMR (101 MHz, Chloroform-*d*) δ 196.56 , 169.44 , 166.32 , 150.06 , 149.54 , 133.77 , 131.63 , 127.91 , 127.79 , 125.91 , 124.19 , 60.88 , 28.28 , 24.75 , 21.51 , 21.34 , 21.06 , 13.52 . HRMS (ESI) Calculated for $C_{18}H_{20}O_5$ $[M+Na]^+$: 339.1203, found: 339.1200. IR ν (neat, cm^{-1}): 2937.0, 1768.1, 1710.3, 1671.1, 1602.4, 1448.7, 1367.8, 1279.9, 1241.3, 1187.1, 1046.1, 910.8, 696.7.

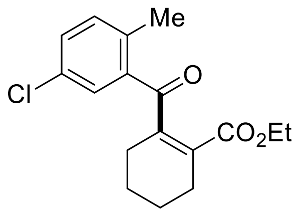

ethyl 2-(5-chloro-2-methylbenzoyl)cyclohex-1-ene-1-carboxylate (**3u**). The reaction was carried out according to the general procedure **A** on 0.2 mmol scale (24 h); purified by flash column chromatography on SiO_2 (eluent: petroleum ether/ethyl acetate) to afford **3u**, 41.1 mg, 67%, colorless oil; Rf = 0.5 (petroleum ether/ethyl acetate 10:1). ^{1}H NMR (400 MHz, Chloroform-*d*) δ 7.48 – 7.45 (m, 1H), 7.34 – 7.30 (m, 1H), 7.20 – 7.18 (m, 1H), 3.95 (q, *J* =

7.1, 2H), 2.60 (m, 3H), 2.44 – 2.41 (m, 2H), 2.38 – 2.32 (m, 2H), 1.76 – 1.73 (m, 4H), 1.03 (t, *J* = 7.1, 3H). ^{13}C NMR (101 MHz, Chloroform-*d*) δ 199.29 , 166.47 , 149.29 , 138.59 , 136.07 , 133.44 , 131.60 , 131.09 , 130.30 , 129.14 , 60.80 , 28.71 , 24.89 , 21.54 , 21.36 , 21.21 , 13.70 . HRMS (ESI) Calculated for $C_{17}H_{19}ClO_3$ $[M+Na]^+$: 329.0915, found: 329.0914. IR ν (neat, cm^{-1}): 2934.9, 2861.3, 1710.5, 1674.5, 1278.7, 1250.3, 1046.1, 932.2, 819.0, 717.3.

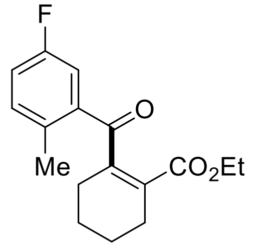

ethyl 2-(5-fluoro-2-methylbenzoyl)cyclohex-1-ene-1-carboxylate (**3v**). The reaction was carried out according to the general procedure **A** on 0.2 mmol scale (24 h); purified by flash column chromatography on SiO_2 (eluent: petroleum ether/ethyl acetate) to afford **3v**, 45.2 mg, 78%, colorless oil; Rf = 0.5 (petroleum ether/ethyl acetate 10:1). ^{1}H NMR (400 MHz, Chloroform-*d*) δ 7.29 – 7.21 (m, 2H), 7.12 – 7.06 m, 1H), 3.96 (qd, *J* = 7.1, 1.5 Hz, 2H), 2.61 (s, 3H), 2.45 – 2.42 (m, 2H), 2.39 – 2.35 (m, 2H), 1.79 – 1.71 (m, 4H), 1.03 (td, *J* = 7.2, 1.5 Hz, 3H). ^{13}C NMR (101 MHz, Chloroform-*d*) δ 199.30 , 166.41 , 160.40 (d, *J* = 244.6 Hz), 149.50 , 135.82 (d, *J* = 3.3 Hz), 135.68 (d, *J* = 5.4 Hz), 133.49 (d, *J* = 7.0 Hz), 128.76 , 118.62 (d, *J* = 20.7 Hz), 117.20 (d, *J* = 22.1 Hz), 60.77 , 28.71 , 24.82 , 21.55 , 21.35 , 21.05 , 13.66 . ^{19}F NMR (376 MHz, Chloroform-*d*) δ -117.15 (d, *J* = 7.2 Hz). HRMS (ESI) Calculated for $C_{17}H_{19}FO_3$ $[M+Na]^+$: 313.1210, found: 313.1204. IR ν (neat, cm^{-1}): 2935.8, 1711.6, 1674.7, 1491.9, 1298.6, 1278.7, 1245.5, 1179.3, 1045.8, 817.7.

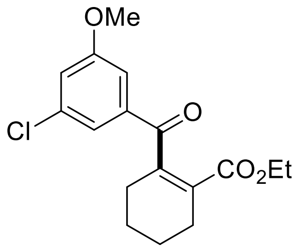

ethyl 2-(3-chloro-5-methoxybenzoyl)cyclohex-1-ene-1-carboxylate (**3w**). The reaction was carried out according to the general procedure **A** on 0.2 mmol scale (24 h); purified by flash column chromatography on SiO_2 (eluent: petroleum ether/ethyl acetate) to afford **3w**, 27.1 mg, 42%, light yellow solid; mp = 45-46 °C, Rf = 0.2 (petroleum ether/ethyl acetate 10:1). ^{1}H NMR (400 MHz, Chloroform-*d*) δ 7.36 – 7.34 (m, 2H), 7.08 – 7.06 (m, 1H), 3.98 (q, *J* = 7.1 Hz, 2H), 3.85 (s, 3H), 2.48 – 2.43 (m, 2H), 2.35 – 2.31 (m, 2H), 1.81 – 1.72 (m, 4H), 1.04 (t, *J* = 7.1 Hz, 3H). ^{13}C NMR (101 MHz, Chloroform-*d*) δ 197.41 , 166.01 , 160.53 , 149.03 , 137.68 , 135.34 , 128.29 , 121.17 , 119.12 , 111.71 , 60.95 , 55.74 , 28.77 , 24.45 , 21.56 , 21.26 , 13.70 . HRMS (ESI) Calculated for $C_{17}H_{19}ClO_4$ $[M+Na]^+$: 345.0864, found: 345.0861. IR ν (neat, cm^{-1}): 2938.8, 1711.9, 1677.0, 1574.9, 1459.1, 1419.1, 1279.3, 1250.7, 1048.0, 728.5.

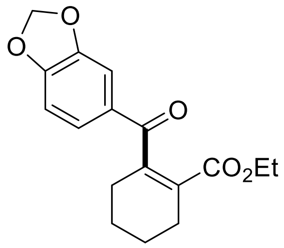

ethyl 2-(benzo[d][1,3]dioxole-5-carbonyl)cyclohex-1-ene-1-carboxylate (**3x**). The reaction was carried out according to the general procedure **A** on 0.2 mmol scale (24 h); purified by flash column chromatography on SiO_2 (eluent: petroleum ether/ethyl acetate) to afford **3x**, 42.3 mg, 70%, colorless oil; Rf = 0.3 (petroleum ether/ethyl acetate 10:1). ^{1}H NMR (400 MHz, Chloroform-*d*) δ 7.42 – 7.39 (m, 2H), 6.83 (dd, *J* = 7.9, 0.6 Hz, 2H), 6.04 (s, 2H), 3.96 (q, *J* = 7.1 Hz, 2H), 2.46 – 2.42 (m, 2H), 2.36 – 2.32 (m, 2H), 1.80 – 1.71 (m, 4H), 1.01 (t, *J* = 7.1 Hz, 3H). ^{13}C NMR (101 MHz, Chloroform-*d*) δ 197.26 , 166.19 , 151.82 , 149.54 , 148.28 , 130.33 , 127.45 , 125.20 , 108.03 , 101.84 , 60.76 , 29.02 , 24.58 , 21.68 , 21.39 , 13.70 . HRMS (ESI) Calculated for $C_{17}H_{18}O_5$ $[M+Na]^+$: 325.1046, found: 325.1044. IR ν (neat, cm^{-1}): 2936.8, 1710.4, 1661.8, 1486.6, 1440.0, 1359.4, 1256.7, 1037.7, 932.4, 744.2.

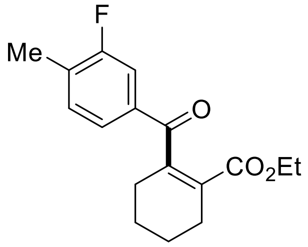

ethyl 2-(3-fluoro-4-methylbenzoyl)cyclohex-1-ene-1-carboxylate (**3y**). The reaction was carried out according to the general procedure **A** on 0.2 mmol scale (24 h); purified by flash column chromatography on SiO_2 (eluent: petroleum ether/ethyl acetate) to afford **3y**, 50.0 mg, 86%, light yellow oil; Rf = 0.5 (petroleum ether/ethyl acetate 10:1). ^{1}H NMR (400 MHz, Chloroform-*d*) δ 7.55 – 7.50 (m, 2H), 7.28 – 7.24 (m, 1H), 3.95 (q, *J* = 7.1 Hz, 2H), 2.49 – 2.43 (m, 2H), 2.38 – 2.31 (m, 5H), 1.81 – 1.72 (m, 4H), 1.00 (t, *J* = 7.1 Hz, 3H). ^{13}C NMR (101 MHz, Chloroform-*d*) δ 197.52 (d, *J* = 2.2 Hz), 166.04 , 161.30 (d, *J* = 246.5 Hz), 149.16 , 135.22 (d, *J* = 6.3 Hz), 131.65 (d, *J* = 4.9 Hz), 130.78 (d, *J* = 17.6 Hz), 127.89 , 124.15 (d, *J* = 3.2 Hz), 114.67 (d, *J* = 23.2 Hz), 60.81 , 28.77 , 24.49 , 21.60 , 21.30 , 14.85 (d, *J* = 3.6 Hz), 13.61 . ^{19}F NMR (376 MHz, Chloroform-*d*) δ -116.26 . HRMS (ESI) Calculated for $C_{17}H_{19}FO_3$ $[M+Na]^+$: 313.1210, found: 313.1207. IR ν (neat, cm^{-1}): 2937.6, 1712.4, 1673.3, 1416.0, 1278.0, 1265.0, 1246.9, 1048.0, 738.0.

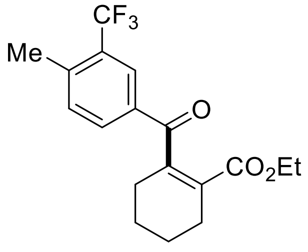

ethyl 2-(4-methyl-3-(trifluoromethyl)benzoyl)cyclohex-1-ene-1-carboxylate (**3z**). The reaction

was carried out according to the general procedure **A** on 0.2 mmol scale (24 h); purified by flash column chromatography on SiO_2 (eluent: petroleum ether/ethyl acetate) to afford **3z**, 27.2 mg, 40%, light yellow solid; mp = 48-50 °C, Rf = 0.5 (petroleum ether/ethyl acetate 10:1). ^{1}H NMR (400 MHz, Chloroform-*d*) δ 8.12 (s, 1H), 7.87 (d, *J* = 7.9 Hz, 1H), 7.37 (d, *J* = 7.9 Hz, 1H), 3.95 (q, *J* = 7.1 Hz, 2H), 2.53 (s, 3H), 2.46 – 2.44 (m, 2H), 2.34 – 2.31 (m, 2H), 1.80 – 1.76 (m, 4H), 0.99 (t, *J* = 7.1 Hz, 3H). ^{13}C NMR (126 MHz, Chloroform-*d*) δ 197.47 , 166.03 , 149.02 , 142.13 , 133.33 , 132.38 , 131.61 , 129.50 (q, *J* = 30.5 Hz), 128.28 , 125.87 (q, *J* = 5.6 Hz), 124.00 (q, *J* = 273.9 Hz), 60.90 , 28.73 , 24.48 , 21.58 , 21.27 , 19.56 (d, *J* = 2.3 Hz), 13.62 . ^{19}F NMR (376 MHz, Chloroform-*d*) δ -62.01 . HRMS (ESI) Calculated for $C_{18}H_{19}F_3O_3$ $[M+Na]^+$: 363.1179, found: 363.1173. IR ν (neat, cm^{-1}): 2939.6, 1712.2, 1675.3, 1612.5, 1320.1, 1279.4, 1255.4, 1233.5, 1169.5, 1124.7, 1051.4, 737.4, 669.4.

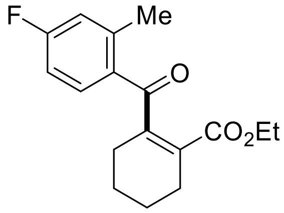

ethyl 2-(4-fluoro-2-methylbenzoyl)cyclohex-1-ene-1-carboxylate (**3aa**). The reaction was carried out according to the general procedure **A** on 0.2 mmol scale (24 h); purified by flash column chromatography on SiO_2 (eluent: petroleum ether/ethyl acetate) to afford **3aa**, 40.6 mg, 70%, light yellow oil; Rf = 0.5 (petroleum ether/ethyl acetate 10:1). ^{1}H NMR (400 MHz, Chloroform-*d*) δ 7.60 – 7.56 (m, 1H), 6.95 (dd, *J* = 9.6, 2.6 Hz, 1H), 6.88 (td, *J* = 8.3, 2.6 Hz, 1H), 3.93 (q, *J* = 7.1, 2H), 2.66 (s, 3H), 2.43 – 3.40 (m, 2H), 2.37 – 2.34 (m, 2H), 1.77 – 1.71 (m, 4H), 1.00 (t, *J* = 7.1, 3H). ^{13}C NMR (101 MHz, Chloroform-*d*) δ 199.16 , 166.47 , 164.24 (d, J = 253.8 Hz), 149.98 , 144.13 (d, *J* = 8.8 Hz), 133.55 (d, *J* = 9.5 Hz), 130.86 , 128.10 , 119.03 (d, *J* = 21.3 Hz), 112.35 (d, *J* = 21.4 Hz), 60.70 , 28.87 , 24.83 , 22.11 , 21.61 , 21.41 , 13.67 . ^{19}F NMR (376 MHz, Chloroform-*d*) δ -107.23 . HRMS (ESI) Calculated for $C_{17}H_{19}FO_3$ $[M+Na]^+$: 313.1210, found: 313.1207. IR ν (neat, cm^{-1}): 2936.2, 1711.0, 1669.2, 1582.1, 1448.2, 1303.7, 1232.3, 1046.3, 963.5, 864.7.

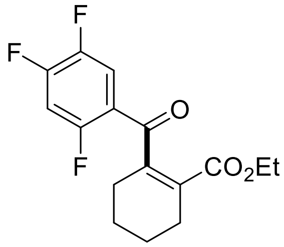

ethyl 2-(2,4,5-trifluorobenzoyl)cyclohex-1-ene-1-carboxylate (**3bb**). The reaction was carried out according to the general procedure **A** on 0.2 mmol scale (24 h); purified by flash column chromatography on SiO_2 (eluent: petroleum ether/ethyl acetate) to afford **3bb**, 38.7 mg, 62%, colorless oil; Rf = 0.6 (petroleum ether/ethyl acetate 10:1). ^{1}H NMR (400 MHz, Chloroform-*d*) δ 7.87 – 7.81 (m, 1H), 6.97 – 6.91 (m, 1H), 4.02 (q, *J* = 7.1 Hz, 2H), 2.40 – 2.36 (m, 2H), 2.32 – 2.28 (m, 2H), 1.76 – 1.70 (m, 4H), 1.09 (t, *J* = 7.1 Hz, 3H). ^{13}C NMR (101 MHz, Chloroform-*d*) δ 192.96 , 166.30 , 159.05 – 155.85 (m), 154.81 – 151.48 (m), 151.14 , 147.16 (ddd, *J* = 247.8, 12.7, 3.4 Hz), 126.59 (d, *J* = 3.6 Hz), 121.82 – 120.40 (m), 118.50 (dt, *J* = 20.0, 2.7 Hz),

106.54 (dd, $J = 29.0, 21.0$ Hz), 60.91 , 27.73 (d, $J = 2.6$ Hz), 24.38 , 21.37 , 21.33 , 13.81 . ^{19}F NMR (376 MHz, Chloroform-*d*) δ -111.96 (dd, $J = 15.7, 8.2$ Hz), -124.92 (dd, $J = 21.7, 8.2$ Hz), -140.87 (dd, $J = 21.5, 15.9$ Hz). HRMS (ESI) Calculated for $C_{16}H_{15}F_3O_3$ $[M+H]^+$: 313.1046, found: 313.1043. IR ν (neat, cm^{-1}): 2936.9, 1708.3, 1674.7, 1623.9, 1510.2, 1426.7, 1279.5, 1253.0, 1225.9, 1142.3, 1064.3, 1044.8, 900.4, 790.6, 751.4, 621.6.

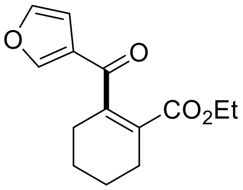

ethyl 2-(furan-3-carbonyl)cyclohex-1-ene-1-carboxylate (**3cc**). The reaction was carried out according to the general procedure **A** on 0.2 mmol scale (24 h); purified by flash column chromatography on SiO_2 (eluent: petroleum ether/ethyl acetate) to afford **3cc**, 29.8 mg, 60%, colorless oil; Rf = 0.2 (petroleum ether/ethyl acetate 10:1). ^{1}H NMR (400 MHz, Chloroform-*d*) δ 7.80 – 7.79 (m, 1H), 7.45 – 7.42 (m, 1H), 6.76 (d, $J = 1.9$ Hz, 1H), 3.97 (q, $J = 7.1$ Hz, 2H), 2.43 – 2.32 (m, 4H), 1.75 – 1.67 (m, 4H), 1.04 (t, $J = 7.1$ Hz, 3H). ^{13}C NMR (101 MHz, Chloroform-*d*) δ 192.94 , 166.38 , 148.99 , 147.72 , 144.42 , 127.98 , 126.79 , 108.59 , 60.84 , 28.44 , 24.66 , 21.59 , 21.34 , 13.60. HRMS (ESI) Calculated for $C_{14}H_{16}O_4$ $[M+Na]^+$: 271.0941, found: 271.0938. IR ν (neat, cm^{-1}): 3131.1, 2937.8, 1711.6, 1664.3, 1562.5, 1508.7, 1303.9, 1279.2, 1247.0, 1166.6, 1154.1, 1049.1, 872.4, 741.6, 723.8, 600.9.

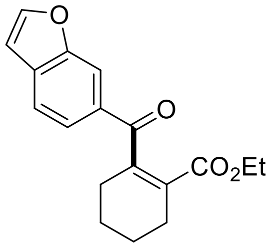

ethyl 2-(benzofuran-6-carbonyl)cyclohex-1-ene-1-carboxylate (**3dd**). The reaction was carried out according to the general procedure **A** on 0.2 mmol scale (24 h); purified by flash column chromatography on SiO_2 (eluent: petroleum ether/ethyl acetate) to afford **3dd**, 36.4 mg, 61%, colorless oil; Rf = 0.2 (petroleum ether/ethyl acetate 10:1). ^{1}H NMR (400 MHz, Chloroform-*d*) δ 8.13 (d, $J = 1.7$ Hz, 1H), 7.90 (dd, $J = 8.7, 1.6$ Hz, 1H), 7.68 (d, $J = 2.2$ Hz, 1H), 7.54 (d, $J = 8.6$ Hz, 1H), 6.85 – 6.84 (m, 1H), 3.92 – 3.87 (m, 2H), 2.49 – 2.44 (m, 2H), 2.41 – 2.38 (m, 2H), 1.83 – 1.74 (m,, 4H), 0.91 (t, $J = 7.2$, 3H). ^{13}C NMR (101 MHz, Chloroform-*d*) δ 198.50 , 166.20 , 157.51 , 149.72 , 146.30 , 130.87 , 127.59 , 127.47 , 125.22 , 122.90 , 111.63 , 107.28 , 60.71 , 28.99 , 24.59 , 21.68 , 21.39 , 13.55 . HRMS (ESI) Calculated for $C_{18}H_{18}O_4$ $[M+Na]^+$: 321.1097, found: 321.1094. IR ν (neat, cm^{-1}): 3117.6, 2936.6, 1710.3, 1666.5, 1439.2, 1278.2, 1248.4, 1046.9, 750.0, 658.0.

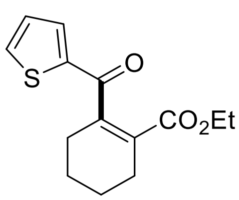

ethyl 2-(thiophene-2-carbonyl)cyclohex-1-ene-1-carboxylate (**3ee**). The reaction was carried

out according to the general procedure **A** on 0.2 mmol scale (24 h); purified by flash column chromatography on SiO_2 (eluent: petroleum ether/ethyl acetate) to afford **3ee**, 35.9 mg, 68%, light yellow oil; Rf = 0.4 (petroleum ether/ethyl acetate 10:1). ^{1}H NMR (400 MHz, Chloroform-*d*) δ 7.65 (dd, *J* = 4.9, 1.2 Hz, 1H), 7.52 (dd, *J* = 3.8, 1.3 Hz, 1H), 7.10 (dd, *J* = 5.1, 3.9 Hz, 1H), 3.95 (q, *J* = 7.3, 2H), 2.47 – 2.41 (m, 4H), 1.79 – 1.71 (m, 4H), 1.00 (t, *J* = 7.2, 3H). ^{13}C NMR (101 MHz, Chloroform-*d*) δ 191.29 , 166.17 , 148.16 , 143.22 , 133.81 , 132.67 , 128.37 , 128.02 , 60.81 , 28.77 , 24.69 , 21.56 , 21.35 , 13.45 . HRMS (ESI) Calculated for $C_{14}H_{16}O_3S$ $[M+Na]^+$: 287.0712, found: 287.0707. IR ν (neat, cm^{-1}): 3100.6, 1710.0, 1649.8, 1516.3, 1411.6, 1269.9, 1244.1, 1142.6, 1048.6, 857.8, 719.7.

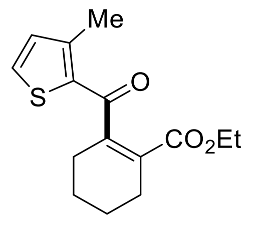

ethyl 2-(3-methylthiophene-2-carbonyl)cyclohex-1-ene-1-carboxylate (**3ff**). The reaction was carried out according to the general procedure **A** on 0.2 mmol scale (24 h); purified by flash column chromatography on SiO_2 (eluent: petroleum ether/ethyl acetate) to afford **3ff**, 22.2 mg, 40%, light yellow oil; Rf = 0.3 (petroleum ether/ethyl acetate 10:1). ^{1}H NMR (400 MHz, Chloroform-*d*) δ 7.42 (d, *J* = 4.9 Hz, 1H), 6.93 (d, *J* = 4.9 Hz, 1H), 3.99 (q, *J* = 7.2 Hz, 2H), 2.55 (s, 3H), 2.44 – 2.39 (m, 4H), 1.77 – 1.72 (m, 4H), 1.01 (t, *J* = 7.1 Hz, 3H). ^{13}C NMR (101 MHz, Chloroform-*d*) δ 191.83 , 166.14 , 150.13 , 144.75 , 135.40 , 132.61 , 130.94 , 127.49 , 60.75 , 28.71 , 24.64 , 21.53 , 21.45 , 16.31 , 13.51 . HRMS (ESI) Calculated for $C_{15}H_{18}O_3S$ $[M+Na]^+$: 301.0869, found: 301.0866. IR ν (neat, cm^{-1}): 2935.2, 1712.5, 1651.1, 1636.0, 1401.6, 1279.1, 1244.4, 1143.1, 1046.4, 732.5.

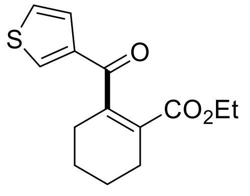

ethyl 2-(thiophene-3-carbonyl)cyclohex-1-ene-1-carboxylate (**3gg**). The reaction was carried out according to the general procedure **A** on 0.2 mmol scale (24 h); purified by flash column chromatography on SiO_2 (eluent: petroleum ether/ethyl acetate) to afford **3gg**, 35.9 mg, 68%, colorless oil; Rf = 0.2 (petroleum ether/ethyl acetate 10:1). ^{1}H NMR (400 MHz, Chloroform-*d*) δ 7.89 – 7.88 (m, 1H), 7.52 – 7.50 (m, 1H), 7.34 – 7.29 (m, 1H), 3.93 (q, *J* = 7.1 Hz, 3H), 2.46 – 2.42 (m, 2H), 2.40 – 2.37 (m, 2H), 1.75 (q, *J* = 3.0 Hz, 4H), 0.97 (t, *J* = 7.2, 3H). ^{13}C NMR (101 MHz, Chloroform-*d*) δ 192.72 , 166.32 , 149.26 , 141.13 , 132.57 , 127.70 , 126.90 , 126.62 , 60.78 , 28.60 , 24.63 , 21.64 , 21.39 , 13.48 . HRMS (ESI) Calculated for $C_{14}H_{16}O_3S$ $[M+Na]^+$: 287.0712, found: 287.0709. IR ν (neat, cm^{-1}): 3103.9, 2936.3, 1710.1, 1659.8, 1510.4, 1394.1, 1369.4, 1279.2, 1244.9, 1141.1, 1047.6, 731.5, 718.1.

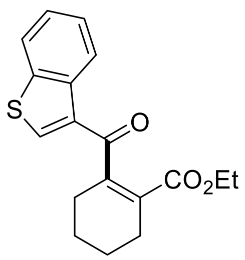

ethyl 2-(benzo[b]thiophene-3-carbonyl)cyclohex-1-ene-1-carboxylate (**3hh**). The reaction was carried out according to the general procedure **A** on 0.2 mmol scale (24 h); purified by flash column chromatography on SiO_2 (eluent: petroleum ether/ethyl acetate) to afford **3hh**, 37.7 mg, 60%, light yellow solid; mp = 89-90 °C, Rf = 0.3 (petroleum ether/ethyl acetate 10:1). ^{1}H NMR (400 MHz, Chloroform-*d*) δ 8.82 (dt, *J* = 8.2, 1.0 Hz, 1H), 8.03 (s, 1H), 7.85 (d, *J* = 8.2 Hz, 1H), 7.53 – 7.48 (m, 1H), 7.44 – 7.40 (m, 1H), 3.85 (q, *J* = 7.1 Hz, 2H), 2.49 – 2.46 (m, 4H), 1.81 – 1.73 m, 4H), 0.82 (t, *J* = 7.1 Hz, 3H). ^{13}C NMR (101 MHz, Chloroform-*d*) δ 193.50 , 166.41 , 149.10 , 140.14 , 137.78 , 136.42 , 134.12 , 128.24 , 125.81 , 125.51 (d, *J* = 2.9 Hz), 122.19 , 60.74 , 28.93 , 24.78 , 21.68 , 21.47 , 13.46 . HRMS (ESI) Calculated for $C_{18}H_{18}O_3S$ $[M+Na]^+$: 337.0869, found: 337.0866. IR ν (neat, cm^{-1}): 3092.1, 2936.4, 1710.0, 1653.2, 1459.8, 1367.0, 1278.5, 1248.0, 1208.5, 1051.5, 859.0, 767.5, 708.1.

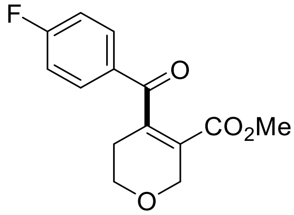

methyl 4-(4-fluorobenzoyl)-5,6-dihydro-2H-pyran-3-carboxylate (**3ii**). The reaction was carried out according to the general procedure **A** on 0.2 mmol scale (24 h); purified by flash column chromatography on SiO_2 (eluent: petroleum ether/ethyl acetate) to afford **4a**, 33.3 mg, 63%, colorless oil; Rf = 0.2 (petroleum ether/ethyl acetate 10:1). ^{1}H NMR (400 MHz, Chloroform-*d*) δ 7.94 – 7.89 (m, 2H), 7.17 – 7.12 (m, 2H), 4.45 (t, *J* = 2.8 Hz, 2H), 3.89 (t, *J* = 5.5 Hz, 2H), 3.53 (s, 3H), 2.50 – 2.46 (m, 2H). ^{13}C NMR (101 MHz, Chloroform-*d*) δ 195.77 , 165.95 (d, J = 255.8 Hz), 164.06 , 147.37 , 131.44 , 131.19 (d, *J* = 9.5 Hz), 126.75 , 116.04 (d, *J* = 22.1 Hz), 64.06 , 63.15 , 51.80 , 28.25 . ^{19}F NMR (376 MHz, Chloroform-*d*) δ -104.19 . HRMS (ESI) Calculated for $C_{14}H_{13}FO_4$ $[M+H]^+$: 265.0871, found: 265.0868. IR ν (neat, cm^{-1}): 2922.4, 2850.8, 1719.3, 1673.2, 1597.2, 1504.6, 1270.6, 1233.6, 1152.8, 1078.4, 1045.1, 848.7, 750.0, 570.4.

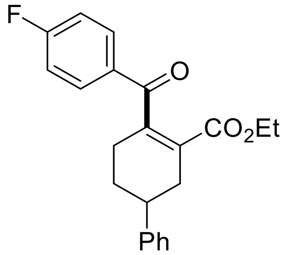

ethyl 4-(4-fluorobenzoyl)-1,2,5,6-tetrahydro-[1,1'-biphenyl]-3-carboxylate (**3jj**). The reaction was carried out according to the general procedure **A** on 0.2 mmol scale (24 h); purified by

flash column chromatography on SiO_2 (eluent: petroleum ether/ethyl acetate) to afford **4b**, 52.8 mg, 75%, light yellow solid; mp = 77-79 °C, Rf = 0.5 (petroleum ether/ethyl acetate 10:1). ^{1}H NMR (400 MHz, Chloroform-*d*) δ 7.94 – 7.90 (m, 2H), 7.38 – 7.34 (m, 2H), 7.30 – 7.24 (m, 3H), 7.14 (t, *J* = 8.6 Hz, 2H), 3.99 – 3.92 (m, 2H), 2.99 – 2.86 (m, 2H), 2.56 – 2.45 (m, 3H), 2.13 – 2.07 (m, 2H), 1.95 – 1.85 (m, 1H), 0.99 (t, *J* = 7.1 Hz, 3H). ^{13}C NMR (101 MHz, Chloroform-*d*) δ 197.21 , 165.75 , 165.75 (d, J = 255.1 Hz) , 148.95 , 145.07 , 131.78 (d, *J* = 2.9 Hz), 131.16 (d, *J* = 9.3 Hz), 128.60 , 127.72 , 126.80 , 126.57 , 115.85 (d, *J* = 22.0 Hz), 60.98 , 39.24 , 32.48 , 29.52 , 28.35 , 13.61 . ^{19}F NMR (376 MHz, Chloroform-*d*) δ -104.91 . HRMS (ESI) Calculated for $C_{22}H_{21}FO_3$ $[M+Na]^+$: 375.1367, found: 375.1365. IR ν (neat, cm^{-1}): 2928.5, 1710.4, 1669.7, 1504.0, 1245.0, 1151.8, 849.3, 747.8, 770.8.

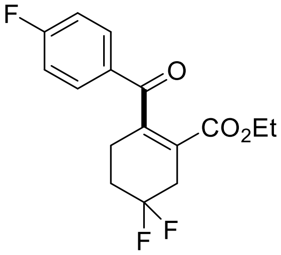

ethyl 5,5-difluoro-2-(4-fluorobenzoyl)cyclohex-1-ene-1-carboxylate (**3kk**). The reaction was carried out according to the general procedure **A** on 0.2 mmol scale (24 h); purified by flash column chromatography on SiO_2 (eluent: petroleum ether/ethyl acetate) to afford **4c**, 40.6 mg, 65%, light yellow solid; mp = 86-88 °C, Rf = 0.3 (petroleum ether/ethyl acetate 10:1). ^{1}H NMR (400 MHz, Chloroform-*d*) δ 7.89 – 7.84 (m, 2H), 7.17 – 7.11 (m, 2H), 3.98 (q, *J* = 7.1 Hz, 2H), 3.00 – 2.92 (m, 2H), 2.67 – 2.62 (m, 2H), 2.22 – 2.12 (m, 2H), 1.00 (t, *J* = 7.1 Hz, 3H). ^{13}C NMR (101 MHz, Chloroform-*d*) δ 195.79 , 165.97 (d, J = 255.8 Hz), 164.33 , 147.83 , 131.46 , 131.14 (d, *J* = 9.5 Hz), 123.75 , 121.82 (t, *J* = 240.0 Hz), 116.08 (d, *J* = 22.3 Hz), 61.51 , 34.10 (t, *J* = 28.4 Hz), 29.19 (t, *J* = 24.8 Hz), 27.43 (t, *J* = 5.5 Hz), 13.55 . ^{19}F NMR (376 MHz, Chloroform-*d*) δ -97.27 , -104.06 . HRMS (ESI) Calculated for $C_{16}H_{15}F_3O_3$ $[M+H]^+$: 313.1046, found: 3113.1043. IR ν (neat, cm^{-1}): 2983.6, 1714.3, 1672.6, 1596.8, 1504.9, 1374.1, 1291.9, 1255.8, 1152.0, 1120.2, 1039.2, 850.4, 751.7, 580.6.

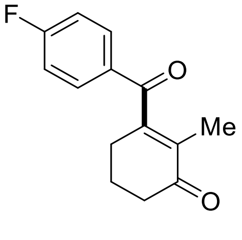

3-(4-fluorobenzoyl)-2-methylcyclohex-2-en-1-one (**3ll**). The reaction was carried out according to the general procedure **A** on 0.2 mmol scale (24 h); purified by flash column chromatography on SiO_2 (eluent: petroleum ether/ethyl acetate) to afford **4d**, 13.9 mg, 30%, light yellow oil; Rf = 0.3 (petroleum ether/ethyl acetate 4:1). ^{1}H NMR (400 MHz, Chloroform-*d*) δ 7.86 – 7.92 (m, 2H), 7.26 – 7.13 (m, 2H), 2.56 – 2.49 (m, 4H), 2.17 – 2.11 (m, 2H), 1.60 (t, *J* = 1.9 Hz, 3H). ^{13}C NMR (101 MHz, Chloroform-*d*) δ 198.70 , 196.17 , 166.38 (d, *J* = 257.1 Hz), 152.78 , 131.89 , 131.89 (d, *J* = 9.8 Hz), 130.84 , 116.37 (d, *J* = 22.1 Hz), 37.73 , 27.98 , 22.72 , 12.67 . ^{19}F NMR (376 MHz, Chloroform-*d*) δ -102.52 . HRMS (ESI) Calculated for $C_{14}H_{13}FO_2$ $[M+H]^+$: 233.0972, found: 233.0970. IR ν (neat, cm^{-1}): 2927.6, 1671.1, 1594.9, 1504.2, 1411.2, 1236.3,

1153.0, 1039.2, 925.8, 849.1.

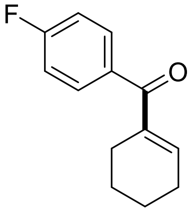

cyclohex-1-en-1-yl(4-fluorophenyl)methanone (**3mm**). The reaction was carried out according to the general procedure **A** on 0.2 mmol scale (24 h); purified by flash column chromatography on SiO_2 (eluent: petroleum ether/ethyl acetate) to afford **4e**, 34.7 mg, 85%, light yellow oil; Rf = 0.7 (petroleum ether/ethyl acetate 10:1). ^{1}H NMR (400 MHz, Chloroform-*d*) δ 7.62 – 7.57 (m, 2H), 7.04 – 6.98 (m, 2H), 6.47 – 6.45 (m, 1H), 2.35 – 2.31 (m, 2H), 2.22 – 2.17 (m, 2H), 1.69 – 1.56 (m, 4H). ^{13}C NMR (101 MHz, Chloroform-*d*) δ 196.68 , 164.65 (d, *J* = 252.0 Hz), 143.47 , 138.60 , 134.67 (d, *J* = 3.0 Hz), 131.56 (d, *J* = 8.9 Hz), 115.04 (d, *J* = 21.7 Hz), 26.02 , 24.00 , 21.92 , 21.57 . ^{19}F NMR (376 MHz, Chloroform-*d*) δ -108.07 . HRMS (ESI) Calculated for $C_{13}H_{13}OF$ $[M+H]^+$: 205.102, found: 205.1023. IR ν (neat, cm^{-1}): 2932.9, 1642.7, 1597.1, 1503.6, 1275.5, 1255.4, 1224.6, 1154.6, 975.2, 889.7, 842.8, 753.5, 688.0, 616.1, 559.0.

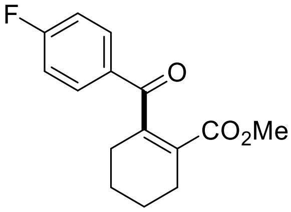

methyl 2-(4-fluorobenzoyl)cyclohex-1-ene-1-carboxylate (**3nn**). The reaction was carried out according to the general procedure **A** on 0.2 mmol scale (24 h); purified by flash column chromatography on SiO_2 (eluent: petroleum ether/ethyl acetate) to afford **4f**, 36.7 mg, 70%, colorless oil; Rf = 0.3 (petroleum ether/ethyl acetate 10:1). ^{1}H NMR (400 MHz, Chloroform-*d*) δ 7.87 (ddd, *J* = 10.2, 5.3, 2.5 Hz, 2H), 7.15 – 7.09 (m, 2H), 3.49 (s, 3H), 2.47 – 2.42 (m, 2H), 2.36 – 2.33 (m, 2H), 1.81 – 1.69 (m, 4H). ^{13}C NMR (101 MHz, Chloroform-*d*) δ 197.47 , 166.58 , 165.69 (d, J = 254.7 Hz) , 149.78 , 131.76 , 131.03 (d, *J* = 9.4 Hz), 127.71 , 115.84 (d, *J* = 22.2 Hz), 51.64 , 28.80 , 24.52 , 21.60 , 21.28 . ^{19}F NMR (376 MHz, Chloroform-*d*) δ -105.20 . HRMS (ESI) Calculated for $C_{15}H_{15}FO_3$ $[M+Na]^+$: 285.0897, found: 285.0892. IR ν (neat, cm^{-1}): 2938.7, 2860.8, 1713.6, 1668.7, 1596.0, 1503.9, 1434.2, 1279.1, 1239.6, 1151.0, 1047.3, 846.1, 749.7, 682.9, 607.9, 503.7.

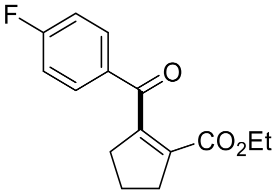

ethyl 2-(4-fluorobenzoyl)cyclopent-1-ene-1-carboxylate (**3oo**). The reaction was carried out according to the general procedure **B**(0.4 mmol K_2CO_3 instead of Cs_2CO_3.) on 0.2 mmol scale (24 h); purified by flash column chromatography on SiO_2 (eluent: petroleum ether/ethyl acetate) to afford **4g**, 21.0 mg, 40%, light yellow oil; Rf = 0.4 (petroleum ether/ethyl acetate 10:1). ^{1}H NMR (400 MHz, Chloroform-*d*) δ 7.93 – 7.88 (m, 2H), 7.16 – 7.11 (m, 2H), 3.94 (q, *J* = 7.1

Hz, 2H), 2.88 – 2.81 (m, 2H), 2.16 – 2.07 (m, 2H), 0.91 (t, *J* = 7.1 Hz, 3H). ^{13}C NMR (101 MHz, Chloroform-*d*) δ 195.10 , 165.99 (d, J = 255.5 Hz), 163.87 , 152.53 , 134.31 , 132.54 (d, *J* = 61.3 Hz), 131.38 (d, *J* = 9.4 Hz), 115.85 (d, *J* = 21.9 Hz), 60.65 , 37.63 , 33.11 , 22.51 , 13.56 . ^{19}F NMR (376 MHz, Chloroform-*d*) δ -104.31 . HRMS (ESI) Calculated for $C_{15}H_{15}FO_3$ $[M+H]^+$: 263.1078, found: 263.1076. IR ν (neat, cm^{-1}): 2920.5, 2851.0, 1713.0, 1669.4, 1596.5, 1504.4, 1268.8, 1225.4, 1152.0, 1105.0, 1020.7, 849.2, 751.5.

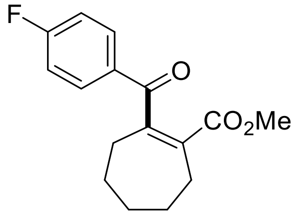

methyl 2-(4-fluorobenzoyl)cyclohept-1-ene-1-carboxylate (**3pp**). The reaction was carried out according to the general procedure **B** on 0.2 mmol scale (24 h); purified by flash column chromatography on SiO_2 (eluent: petroleum ether/ethyl acetate) to afford **4h**, 42.0 mg, 76%, light yellow oil; Rf = 0.4 (petroleum ether/ethyl acetate 10:1). ^{1}H NMR (400 MHz, Chloroform-*d*) δ 7.91 – 7.86 (m, 2H), 7.16 – 7.10 (m, 2H), 3.46 (s, 3H), 2.70 – 2.68 (m, 2H), 2.45 – 2.43 (m, 2H), 1.91 – 1.85 (m, 2H), 1.72 – 1.64 (m, 4 H). ^{13}C NMR (101 MHz, Chloroform-*d*) δ 197.32 , 167.39 , 165.66 (d, J = 254.6 Hz), 154.01 , 134.38 , 131.80 , 131.20 (d, *J* = 9.1 Hz), 115.81 (d, *J* = 22.0 Hz), 51.80 , 32.36 , 32.11 , 28.52 , 25.67 , 25.55 . ^{19}F NMR (376 MHz, Chloroform-*d*) δ -105.31 . HRMS (ESI) Calculated for $C_{16}H_{17}FO_3$ $[M+H]^+$: 277.1234, found: 277.1232. IR ν (neat, cm^{-1}): 2923.2, 2851.5, 1711.8, 1669.1, 1595.9, 1503.9, 1435.0, 1289.2, 1257.8, 1222.8, 1150.1, 1098.4, 892.2, 847.6, 748.4, 612.7, 505.7.

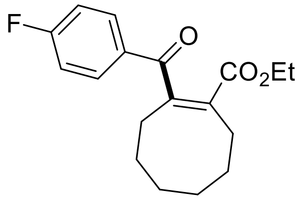

ethyl (Z)-2-(4-fluorobenzoyl)cyclooct-1-ene-1-carboxylate (**3qq**). The reaction was carried out according to the general procedure **B** on 0.2 mmol scale (24 h); purified by flash column chromatography on SiO_2 (eluent: petroleum ether/ethyl acetate) to afford **4i**, 45.0 mg, 74%, light yellow oil; Rf = 0.5 (petroleum ether/ethyl acetate 10:1). ^{1}H NMR (400 MHz, Chloroform-*d*) δ 7.91 – 7.85 (m, 2H), 7.14 – 7.02 (m, 2H), 3.94 (q, *J* = 7.1 Hz, 2H), 2.60 – 2.54 (m, 2H), 2.41 – 2.38 (m, 2H), 1.76 – 1.71 (m, 4H), 1.63 – 1.56 (m, 4H), 0.95 (t, *J* = 7.1 Hz, 3H). ^{13}C NMR (101 MHz, Chloroform-*d*) δ 196.88 , 166.38 , 165.55 (d, J = 254.4 Hz) , 151.51 , 131.88 , 131.25 (d, *J* = 9.2 Hz), 131.07 , 115.62 (d, *J* = 22.0 Hz), 60.85 , 30.50 , 29.49 , 29.03 , 26.46 , 25.98 , 13.50 . ^{19}F NMR (376 MHz, Chloroform-*d*) δ -105.62 . HRMS (ESI) Calculated for $C_{18}H_{21}FO_3$ $[M+H]^+$: 305.1547, found: 305.1545. IR ν (neat, cm^{-1}): 2928.0, 2855.5, 1709.3, 1669.3, 1597.4, 1504.3, 1298.8, 1268.8, 1252.8, 1203.2, 1150.1, 1094.3, 969.9, 848.8, 752.1, 610.9, 505.6.

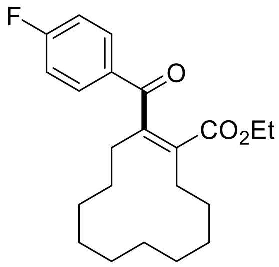

ethyl (Z)-2-(4-fluorobenzoyl)cyclododec-1-ene-1-carboxylate (**3rr**). The reaction was carried out according to the general procedure **A** on 0.2 mmol scale (24 h); purified by flash column chromatography on SiO_2 (eluent: petroleum ether/ethyl acetate) to afford **4j**, 41.1 mg, 57%, light yellow solid; mp = 46-48 °C, Rf = 0.5 (petroleum ether/ethyl acetate 10:1). ^{1}H NMR (400 MHz, Chloroform-*d*) δ 7.91 – 7.85 (m, 2H), 7.10 (t, J = 8.6 Hz, 2H), 3.90 (q, J = 7.1 Hz, 2H), 2.50 – 2.42 m, 4H), 1.76 – 1.70 (m, 2H), 1.54 – 1.31 (m, 12H), 0.97 (t, J = 7.1 Hz, 3H). ^{13}C NMR (101 MHz, Chloroform-*d*) δ 197.05 , 166.89 , 165.54 (d, J = 254.4 Hz) , 150.70 , 132.65 , 131.48 , 131.06 (d, J = 9.3 Hz), 115.65 (d, J = 22.0 Hz), 60.88 , 29.07 , 26.99 , 25.57 , 25.45 , 25.25 , 25.04 , 24.41 , 22.61 , 21.90 , 13.55 . ^{19}F NMR (376 MHz, Chloroform-*d*) δ -105.63 . HRMS (ESI) Calculated for $C_{22}H_{29}FO_3$ $[M+H]^+$: 361.2173, found: 361.2170. IR ν (neat, cm^{-1}): 2929.9, 2860.6, 1710.4, 1672.0, 1620.9, 1597.1, 1504.0, 1297.2, 1255.8, 1185.1, 1150.4, 1095.0, 851.7, 759.3.

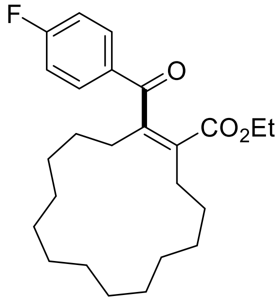

ethyl (Z)-2-(4-fluorobenzoyl)cyclopentadec-1-ene-1-carboxylate (**3ss**). The reaction was carried out according to the general procedure **B** on 0.2 mmol scale (24 h); purified by flash column chromatography on SiO_2 (eluent: petroleum ether/ethyl acetate) to afford **4k**, 56.4 mg, 70%, light yellow oil; Rf = 0.5 (petroleum ether/ethyl acetate 10:1). ^{1}H NMR (400 MHz, Chloroform-*d*) δ 7.93 – 7.85 (m, 2H), 7.15 – 7.09 (m, 2H), 3.90 (q, J = 7.1 Hz, 2H), 2.44 – 2.40 (m, 2H), 2.36 – 2.31 (m, 2H), 1.68 – 1.32 (m, 22H), 0.96 (t, J = 7.1 Hz, 3H). ^{13}C NMR (101 MHz, Chloroform-*d*) δ 196.96 , 166.85 , 165.57 (d, J = 256.1 Hz) , 150.26 , 132.50 (d, J = 3.0 Hz), 131.69 , 131.15 (d, J = 9.2 Hz), 115.67 (d, J = 21.8 Hz), 60.91 , 31.59 , 28.13 , 27.74 , 27.40 , 27.28 , 26.70 , 26.54 , 26.18 , 26.09 , 25.28 , 25.20 , 13.51 . ^{19}F NMR (376 MHz, Chloroform-*d*) δ -105.53 . HRMS (ESI) Calculated for $C_{25}H_{35}FO_3$ $[M+H]^+$: 403.2643, found: 403.2638. IR ν (neat, cm^{-1}): 2927.3, 2856.7, 1711.4, 1671.9, 1597.3, 1504.0, 1460.8, 1257.4, 1187.6, 1150.4, 850.5, 766.2.

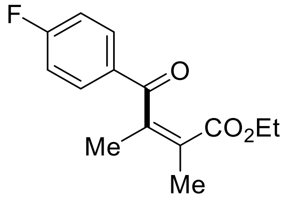

ethyl (Z)-4-(4-fluorophenyl)-2,3-dimethyl-4-oxobut-2-enoate (**3tt**). The reaction was carried out according to the general procedure **B** on 0.2 mmol scale (24 h); purified by flash column chromatography on SiO_2 (eluent: petroleum ether/ethyl acetate) to afford **4l**, 30.0 mg, 60%, Z/E>20:1, light yellow oil; Rf = 0.4 (petroleum ether/ethyl acetate 10:1). ^{1}H NMR (400 MHz, Chloroform-*d*) δ 7.89– 7.84 (m, 2H), 7.13 – 7.08 (m, 2H), 3.93 (q, *J* = 7.1 Hz, 2H), 2.03 – 2.01 (m, 6H), 0.98 (t, *J* = 7.1 Hz, 3H). ^{13}C NMR (101 MHz, Chloroform-*d*) δ 197.40 , 166.53 , 165.66 (d, J = 254.8 Hz) , 146.69 , 131.68 , 131.17 (d, *J* = 9.3 Hz), 126.59 , 115.78 (d, *J* = 22.0 Hz), 61.02 , 18.11 , 13.89 , 13.56 . ^{19}F NMR (376 MHz, Chloroform-*d*) δ -105.25 . HRMS (ESI) Calculated for $C_{14}H_{15}FO_3$ $[M+H]^+$: 251.1078, found: 251.1075. IR ν (neat, cm^{-1}): 2983.6, 1709.5, 1672.4, 1597.1, 1504.3, 1287.0, 1261.9, 1228.2, 1190.9, 1151.5, 1087.1, 850.7, 759.1, 687.6, 606.5, 504.4.

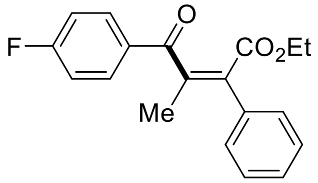

ethyl (Z)-4-(4-fluorophenyl)-3-methyl-4-oxo-2-phenylbut-2-enoate (**3uu**). The reaction was carried out according to the general procedure **B** on 0.2 mmol scale (24 h); purified by flash column chromatography on SiO_2 (eluent: petroleum ether/ethyl acetate) to afford **4m**, 21.8 mg, 35%, Z/E>20:1, light yellow oil; Rf = 0.4 (petroleum ether/ethyl acetate 10:1). ^{1}H NMR (400 MHz, Chloroform-*d*) δ 8.03 – 7.98 (m, 2H), 7.46 – 7.31 (m, 5H), 7.21 – 7.15 (m, 2H), 3.96 (q, *J* = 7.1 Hz, 2H), 1.94 (s, 3H), 0.97 (t, *J* = 7.1 Hz, 3H). ^{13}C NMR (101 MHz, Chloroform-*d*) δ 196.83 , 165.83 , 165.79 (d, J = 255.1 Hz), 148.99 , 134.81 , 132.52 , 131.58 , 131.22 (d, *J* = 9.4 Hz), 129.52 , 128.29 , 127.99 , 115.96 (d, *J* = 21.8 Hz), 61.34 , 19.39 , 13.59 . ^{19}F NMR (376 MHz, Chloroform-*d*) δ -104.75 . HRMS (ESI) Calculated for $C_{19}H_{17}FO_3$ $[M+Na]^+$: 335.1054, found: 335.1051. IR ν (neat, cm^{-1}): 2983.1, 1711.2, 1672.4, 1597.2, 1503.8, 1295.8, 1266.3, 1226.2, 1149.2, 1046.6, 851.7, 721.0, 699.3, 606.0, 505.6.

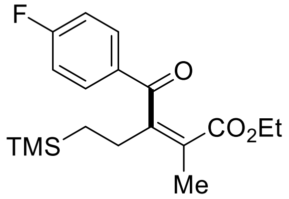

ethyl (Z)-3-(4-fluorobenzoyl)-2-methyl-5-(trimethylsilyl)pent-2-enoate (**3vv**). The reaction was carried out according to the general procedure **B** on 0.2 mmol scale (24 h); purified by flash column chromatography on SiO_2 (eluent: petroleum ether/ethyl acetate) to afford **4n**, 35.7 mg, 53%, Z/E>20:1, light yellow oil; Rf = 0.5 (petroleum ether/ethyl acetate 10:1). ^{1}H NMR (400 MHz, Chloroform-*d*) δ 7.91 – 7.86 (m, 2H), 7.11 (t, *J* = 8.6 Hz, 2H), 3.90 (q, *J* = 7.1 Hz, 2H), 2.34 – 2.29 (m, 2H), 2.02 (s, 3H), 0.97 (t, *J* = 7.1 Hz, 3H), 0.68 – 0.63 (m, 2H), -0.03 (s, 9H). ^{13}C NMR (101 MHz, Chloroform-*d*) δ 197.01 , 166.93 , 165.61 (d, J = 254.6 Hz) , 152.97 , 132.66 , 131.20 (d, *J* = 9.3 Hz), 125.44 , 115.69 (d, *J* = 22.0 Hz), 61.00 , 26.46 , 14.61 , 13.55 , 13.43 , -2.12 . ^{19}F NMR (376 MHz, Chloroform-*d*) δ -105.52 . HRMS (ESI) Calculated for $C_{18}H_{25}FO_3Si$ $[M+H]^+$: 337.1630, found: 337.1627. IR ν (neat, cm^{-1}): 2953.3, 1711.1, 1670.3, 1597.2, 1504.3, 1249.9, 1188.0, 1150.0, 1085.9, 852.2, 836.1, 753.9, 689.3, 611.5, 505.1.

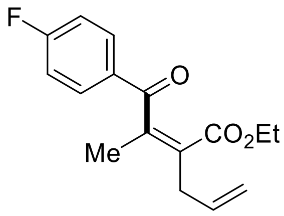

ethyl (Z)-2-(1-(4-fluorophenyl)-1-oxopropan-2-ylidene)pent-4-enoate (**3ww**). The reaction was carried out according to the general procedure **B** on 0.2 mmol scale (24 h); purified by flash column chromatography on SiO_2 (eluent: petroleum ether/ethyl acetate) to afford **4o**, 16.6 mg, 30%, Z/E>20:1, light yellow oil; Rf = 0.5 (petroleum ether/ethyl acetate 10:1). ^{1}H NMR (400 MHz, Chloroform-*d*) δ 7.91 – 7.85 (m, 2H), 7.13 (t, *J* = 8.6 Hz, 2H), 5.94 – 5.84 (m, 1H), 5.19 – 5.09 (m, 2H), 3.95 (q, *J* = 7.1 Hz, 2H), 3.24 – 3.22 (m, 2H), 2.03 (s, 3H), 0.98 (t, *J* = 7.1 Hz, 3H). ^{13}C NMR (101 MHz, Chloroform-*d*) δ 196.96 , 166.05 , 165.70 (d, J = 254.8 Hz) , 148.10 , 134.03 , 131.66 , 131.19 (d, *J* = 9.3 Hz), 128.68 , 116.16 , 115.85 (d, *J* = 21.8 Hz), 61.11 , 32.22 , 17.80 , 13.54 . ^{19}F NMR (376 MHz, Chloroform-*d*) δ -105.11 . HRMS (ESI) Calculated for $C_{16}H_{17}FO_3$ $[M+Na]^+$: 299.1054, found: 299.1051. IR ν (neat, cm^{-1}): 3079.0, 2982.3, 1710.6, 1673.3, 1597.6, 1504.5, 1293.5, 1260.1, 1227.6, 1151.5, 1111.2, 851.1, 761.9, 605.7.

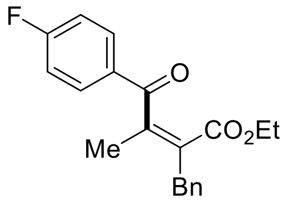

ethyl (Z)-2-benzyl-4-(4-fluorophenyl)-3-methyl-4-oxobut-2-enoate (**3xx**). The reaction was carried out according to the general procedure **B** on 0.2 mmol scale (24 h); purified by flash column chromatography on SiO_2 (eluent: petroleum ether/ethyl acetate) to afford **4p**, 42.4 mg, 65%, Z/E>20:1, light yellow oil; Rf = 0.4 (petroleum ether/ethyl acetate 10:1). ^{1}H NMR (400 MHz, Chloroform-*d*) δ 7.91 – 7.86 (m, 2H), 7.35 – 7.21 (m, 4H), 7.15 – 7.09 (m, 2H), 3.91 (q, *J* = 7.1 Hz, 2H), 3.86 (s, 2H), 2.11 (s, 3H), 0.93 (t, *J* = 7.1 Hz, 3H). ^{13}C NMR (101 MHz, Chloroform-*d*) δ 196.88 , 166.17 , 165.65 (d, J = 254.9 Hz) , 148.30 , 138.63 , 131.65 (d, *J* = 2.9 Hz), 131.15 (d, *J* = 9.5 Hz), 129.90 , 128.56 , 128.18 , 126.35 , 115.84 (d, *J* = 22.0 Hz), 61.14 , 33.68 , 18.30 , 13.44 . ^{19}F NMR (376 MHz, Chloroform-*d*) δ -105.00 . HRMS (ESI) Calculated for $C_{20}H_{19}FO_3$ $[M+H]^+$: 327.1391, found: 327.1388. IR ν (neat, cm^{-1}): 3028.6, 2982.4, 1709.4, 1672.5, 1597.3, 1504.1, 1298.1, 1260.4, 1228.4, 1191.2, 1150.8, 1048.5, 851.7, 762.0, 700.1, 606.6, 504.7.

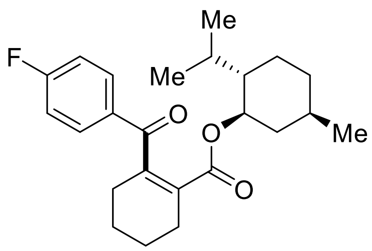

-

(1R,2S,5R)-2-isopropyl-5-methylcyclohexyl 2-(4-fluorobenzoyl)cyclohex-1-ene-1-carboxylate (**3yy**). The reaction was carried out according to the general procedure **A** on 0.2 mmol scale (24 h); purified by flash column chromatography on SiO_2 (eluent: petroleum ether/ethyl acetate) to

afford **5a**, 60.3 mg, 78%, colorless oil; Rf = 0.5 (petroleum ether/ethyl acetate 10:1). ^{1}H NMR (400 MHz, Chloroform-*d*) δ 7.92 – 7.85 (m, 2H), 7.14 – 7.08 (m, 2H), 4.57 – 4.50 (m, 1H), 2.49 – 2.26 (m, 4H), 1.80 – 1.65 (m, 5H), 1.59 – 1.50 (m, 3H), 1.33 – 1.24 (m, 1H), 1.04 – 0.97 (m, 1H), 0.94 – 0.83 (m, 1H), 0.77 – 0.65 (m, 7H), 0.59 (d, *J* = 6.9 Hz, 3H), 0.54 – 0.45 (m, 1H). ^{13}C NMR (101 MHz, Chloroform-*d*) δ 197.20 , 165.71 (d, J = 254.7 Hz), 165.46 , 148.82 , 131.92 , 131.23 (d, *J* = 9.3 Hz), 128.00 , 115.70 (d, *J* = 22.0 Hz), 75.10 , 46.66 , 40.16 , 34.02 , 31.15 , 28.83 , 25.86 , 24.60 , 22.92 , 21.83 , 21.64 , 21.31 , 20.76 , 15.84 . ^{19}F NMR (376 MHz, Chloroform-*d*) δ -105.44 . HRMS (ESI) Calculated for $C_{24}H_{31}FO_3$ [M+Na]$^+$: 409.2149, found: 409.2145. IR ν (neat, cm^{-1}): 2931.9, 2868.2, 1705.8, 1673.2, 1597.5, 1504.2, 1277.8, 1261.5, 1241.6, 1152.0, 1038.7, 845.9, 609.0, 502.7.

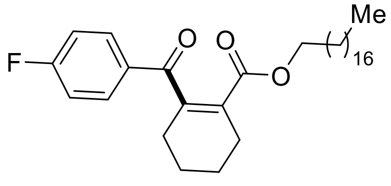

octadecyl 2-(4-fluorobenzoyl)cyclohex-1-ene-1-carboxylate (**3zz**). The reaction was carried out according to the general procedure **A** on 0.2 mmol scale (24 h); purified by flash column chromatography on SiO_2 (eluent: petroleum ether/ethyl acetate) to afford **5b**, 74.1 mg, 74%, light yellow solid; mp = 42-43 °C, Rf = 0.5 (petroleum ether/ethyl acetate 10:1). ^{1}H NMR (400 MHz, Chloroform-*d*) δ 7.91 – 7.86 (m, 2H), 7.14 – 7.08 (m, 2H), 3.87 (t, *J* = 6.6 Hz, 2H), 2.47 – 2.43 (m, 2H), 2.35 – 2.31 (m, 2H), 1.80 – 1.71 (m, 4H), 1.36 – 1.07 (m, 32H), 0.89 – 0.85 (m, 3H). ^{13}C NMR (101 MHz, Chloroform-*d*) δ 197.29 , 166.17 , 165.70 (d, J = 254.8 Hz), 149.10 , 131.75 (d, *J* = 2.9 Hz), 131.19 (d, *J* = 9.4 Hz), 127.85 , 115.77 (d, *J* = 21.9 Hz), 65.15 , 31.89 , 29.67 , 29.63 , 29.60 , 29.54 , 29.42 , 29.33 , 29.16 , 28.79 , 28.16 , 25.81 , 24.57 , 22.66 , 21.63 , 21.32 , 14.08 . ^{19}F NMR (376 MHz, Chloroform-*d*) δ -105.14 . HRMS (ESI) Calculated for $C_{32}H_{49}FO_3$ [M+H]$^+$: 501.3739, found: 507.3737. IR ν (neat, cm^{-1}): 2922.9, 2852.9, 1713.3, 1673.9, 1597.9, 1278.8, 1261.7, 1240.6, 1151.8, 845.7750.7, 608.0.

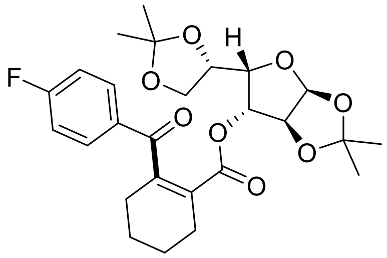

(3aS,5S,6R,6aS)-5-((S)-2,2-dimethyl-1,3-dioxolan-4-yl)-2,2-dimethyltetrahydrofuro[2,3-d][1,3]dioxol-6-yl 2-(4-fluorobenzoyl)cyclohex-1-ene-1-carboxylate (**3AA**). The reaction was carried out according to the general procedure **A** on 0.2 mmol scale (24 h); purified by flash column chromatography on SiO_2 (eluent: petroleum ether/ethyl acetate) to afford **5c**, 70.6 mg, 72%, colorless oil; Rf = 0.3 (petroleum ether/ethyl acetate 4:1). ^{1}H NMR (400 MHz, Chloroform-*d*) δ 7.92 – 7.87 (m, 2H), 7.14 (t, *J* = 8.5 Hz, 2H), 5.43 (d, *J* = 3.6 Hz, 1H), 5.13 (d, *J* = 2.9 Hz, 1H), 4.13 (d, *J* = 3.6 Hz, 1H), 3.99 (dd, *J* = 8.6, 3.0 Hz, 1H), 3.86 – 3.84 (m, 2H), 3.67 – 3.62 (m, 1H), 2.54 – 2.27 (m, 4H), 1.80 – 1.72 (m, 4H), 1.42 (s, 3H), 1.33 (s, 3H), 1.27 (s, 3H), 1.20 (s, 3H). ^{13}C NMR (101 MHz, Chloroform-*d*) δ 196.77 , 165.89 (d, J = 256.0 Hz), 164.68 , 150.66 , 131.67 (d, *J* = 9.2 Hz), 131.26 (d, *J* = 2.8 Hz), 127.14 , 115.83 (d, *J* = 22.1

Hz), 112.08 , 109.27 , 104.78 , 82.84 , 79.47 , 76.78 , 71.92 , 67.28 , 29.03 , 26.83 , 26.57 , 26.02 , 25.18 , 24.63 , 21.50 , 21.16 . ^{19}F NMR (376 MHz, Chloroform-*d*) δ -104.21 . HRMS (ESI) Calculated for $C_{26}H_{31}FO_8$ $[M+Na]^+$: 513.1895, found: 513.1892. IR ν (neat, cm^{-1}): 2987.4, 2936.8, 1718.3, 1672.5, 1597.1, 1372.6, 1257.7, 1240.3, 1153.9, 1075.2, 1020.8, 847.0, 752.9, 608.6,, 509.5.

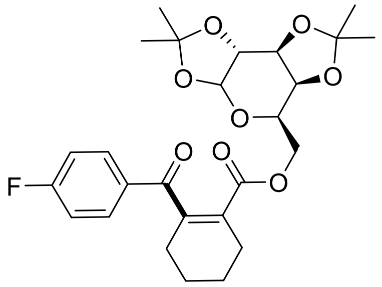

((5R,5aS,8aS,8bR)-2,2,7,7-tetramethyltetrahydro-5H-bis([1,3]dioxolo)[4,5-b:4',5'-d]pyran-5-yl)methyl 2-(4-fluorobenzoyl)cyclohex-1-ene-1-carboxylate (**3BB**) The reaction was carried out according to the general procedure **A** on 0.2 mmol scale (24 h); purified by flash column chromatography on SiO_2 (eluent: petroleum ether/ethyl acetate) to afford **5d**, 80.4 mg, 82%, light yellow oil; Rf = 0.3 (petroleum ether/ethyl acetate 4:1). ^{1}H NMR (400 MHz, Chloroform-*d*) δ 7.92 – 7.86 (m, 2H), 7.22 – 6.97 (m, 2H), 5.43 (d, *J* = 4.9 Hz, 1H), 4.49 (dd, *J* = 7.9, 2.5 Hz, 1H), 4.25 (dd, *J* = 5.0, 2.6 Hz, 1H), 4.09 (dd, *J* = 11.3, 6.0 Hz, 1H), 4.02 (dd, *J* = 11.3, 6.8 Hz, 1H), 3.89 (dd, *J* = 7.9, 1.9 Hz, 1H), 3.81 – 3.77 (m, 1H), 2.47 – 2.44 (m, 2H), 2.36 – 2.30 (m, 2H), 1.79 – 1.72 (m, 4H), 1.44 (s, 3H), 1.36 (s, 3H), 1.30 (s, 3H), 1.27 (s, 3H). ^{13}C NMR (101 MHz, Chloroform-*d*) δ 197.22 , 165.68 , 165.64 (d, J = 254.8 Hz), 150.01 , 131.56 , 131.18 (d, *J* = 9.5 Hz), 127.40 , 115.79 (d, *J* = 22.1 Hz), 109.34 , 108.59 , 96.07 , 70.57 , 70.44 , 70.38 , 65.42 , 63.51 , 28.85 , 25.91 , 25.80 , 24.86 , 24.44 , 24.30 , 21.54 , 21.19 . ^{19}F NMR (376 MHz, Chloroform-*d*) δ -105.16 . HRMS (ESI) Calculated for $C_{26}H_{31}FO_8$ $[M+Na]^+$: 513.1895, found: 513.1891. IR ν (neat, cm^{-1}): 2934.7, 2859.8, 1710.4, 1672.2, 1597.5, 1504.9, 1449.2, 1239.1, 1152.3, 1040.2, 846.4, 750.5.

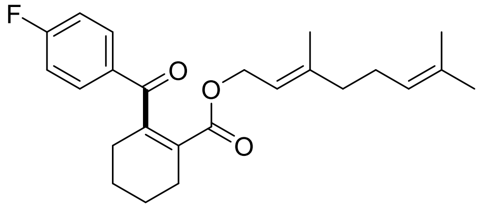

(E)-3,7-dimethylocta-2,6-dien-1-yl 2-(4-fluorobenzoyl)cyclohex-1-ene-1-carboxylate (**3DD**). The reaction was carried out according to the general procedure **A** on 0.2 mmol scale (24 h); purified by flash column chromatography on SiO_2 (eluent: petroleum ether/ethyl acetate) to afford **5e**, 31.5 mg, 41%, colorless oil; Rf = 0.3 (petroleum ether/ethyl acetate 10:1). ^{1}H NMR (400 MHz, Chloroform-*d*) δ 7.92 – 7.85 (m, 2H), 7.14 – 7.08 (m, 2H), 5.06 – 4.98 m, 2H), 4.40 (d, *J* = 7.1 Hz, 2H), 2.47 – 2.43 (m, 2H), 2.35 – 2.30 (m, 2H), 2.03 – 1.97 (m, 2H), 1.92 – 1.88 (m, 2H), 1.80 – 1.72 (m, 4H), 1.67 (s, 3H), 1.58 (s, 3H), 1.51 (s, 3H). ^{13}C NMR (101 MHz, Chloroform-*d*) δ 197.35 , 166.11 , 165.67 (d, J = 254.8 Hz), 149.13 , 142.25 , 131.82 (d, *J* = 10.5 Hz), 131.37 , 131.19 (d, *J* = 9.5 Hz), 127.94 , 123.66 , 117.47 , 115.72 (d, *J* = 22.1 Hz), 61.71 , 39.39 , 31.05 , 28.80 , 26.18 , 25.64 , 24.58 , 21.63 , 21.33 , 17.64 , 16.26 . ^{19}F NMR (376 MHz, Chloroform-*d*) δ -105.33 . HRMS (ESI) Calculated for $C_{24}H_{29}FO_3$ $[M+Na]^+$:

407.1993, found: 407.1989. IR ν (neat, cm^{-1}): 2934.6, 2859.7, 2360.7, 2341.3, 1711.1, 1672.3, 11597.7, 1505.2, 1278.5, 1260.7, 1239.6, 1152.3, 1040.7, 846.5.

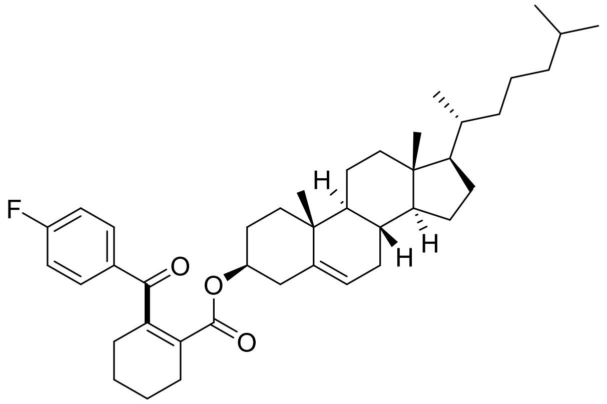

(3S,8S,9S,10R,13R,14S,17R)-10,13-dimethyl-17-((R)-6-methylheptan-2-yl)-2,3,4,7,8,9,10,11,12,13,14,15,16,17-tetradecahydro-1H-cyclopenta[a]phenanthren-3-yl 2-(4-fluorobenzoyl)cyclohex-1-ene-1-carboxylate (**3CC**). The reaction was carried out according to the general procedure **A** on 0.2 mmol scale (24 h); purified by flash column chromatography on SiO_2 (eluent: petroleum ether/ethyl acetate) to afford **5f**, 93.8 mg, 76%, light yellow oil; Rf = 0.8 (petroleum ether/ethyl acetate 10:1). ^{1}H NMR (400 MHz, Chloroform-*d*) δ 7.93 – 7.88 (m, 2H), 7.15 – 7.09 (m, 2H), 5.23 – 5.21 (m, 1H), 4.43 (tt, *J* = 11.4, 4.7 Hz, 1H), 2.45 – 2.42 (m, 2H), 2.35 – 2.31 (m, 2H), 2.04 – 1.67 (m, 10H), 1.58 – 0.80 (m, 34H), 0.63 (s, 3H). ^{13}C NMR (101 MHz, Chloroform-*d*) δ 197.15 , 165.71 (d, J = 255.1 Hz), 165.25 , 148.64 , 139.26 , 131.91 (d, *J* = 2.9 Hz), 131.26 (d, *J* = 9.3 Hz), 127.97 , 122.57 , 115.75 (d, *J* = 21.9 Hz), 74.82 , 56.55 , 56.05 , 49.84 , 42.21 , 39.62 , 39.45 , 37.31 , 36.71 , 36.41 , 36.12 , 35.72 , 31.76 , 31.72 , 28.76 , 28.15 , 27.95 , 27.00 , 24.54 , 24.20 , 23.77 , 22.77 , 22.51 , 21.62 , 21.33 , 20.90 , 19.07 , 18.65 , 11.76 . ^{19}F NMR (376 MHz, Chloroform-*d*) δ -105.01 . HRMS (ESI) Calculated for $C_{41}H_{57}FO_3$ $[M+Na]^+$: 639.4184, found: 639.4180. IR ν (neat, cm^{-1}): 2940.7, 2867.0, 1710.5, 1673.7, 1597.9, 1504.2, 1264.6, 1241.9, 1152.2, 1042.0, 924.2, 846.3, 751.4.

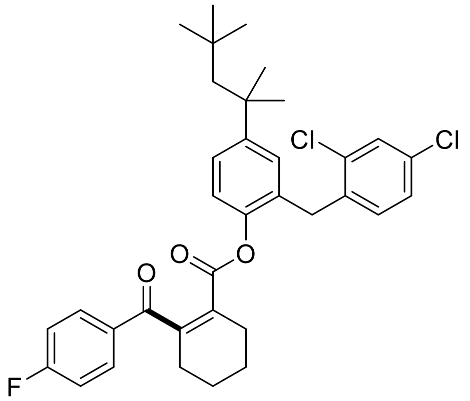

2-(2,4-dichlorobenzyl)-4-(2,4,4-trimethylpentan-2-yl)phenyl 2-(4-fluorobenzoyl)cyclohex-1-ene-1-carboxylate (**3EE**). The reaction was carried out according to the general procedure **A** on 0.2 mmol scale (24 h); purified by flash column chromatography on SiO_2 (eluent: petroleum ether/ethyl acetate) to afford **5g**, 61.9 mg, 52%, light yellow oil; Rf = 0.3 (petroleum ether/ethyl acetate 10:1). ^{1}H NMR (400 MHz, Chloroform-*d*) δ 7.84 – 7.81 (m, 2H), 7.39 (d, *J* = 2.2 Hz, 1H), 7.16 (dd, *J* = 8.5, 2.4 Hz, 1H), 7.09 – 7.02 (m, 3H), 6.98 (d, *J* = 2.4 Hz, 1H), 6.72 (dd, *J* = 8.4, 3.5 Hz, 2H), 3.77 (s, 2H), 2.46 – 2.37 (m, 4H), 1.83 – 1.75 (m, 4H), 1.61 (s, 2H), 1.24 (s, 6H), 0.64 (s, 9H). ^{13}C NMR (101 MHz, Chloroform-*d*) δ 196.92 , 165.71 (d, J = 255.2 Hz),

164.31 , 152.04 , 148.07 , 146.26 , 136.31 , 134.50 , 132.40 , 131.48 , 131.16 , 131.07 , 129.22 , 128.72 (d, J = 15.4 Hz), 126.95 , 125.56 , 121.20 , 115.80 (d, J = 22.1 Hz), 56.77 , 38.23 , 33.18 , 32.21 , 31.70 , 31.41 , 29.15 , 24.55 , 21.57 , 21.17 . ^{19}F NMR (376 MHz, Chloroform-*d*) δ -104.68. HRMS (ESI) Calculated for $C_{35}H_{37}Cl_2FO_3$ $[M+H]^+$: 595.2177, found: 595.2175. IR ν (neat, cm^{-1}): 2949.9, 1729.9, 1671.8, 1597.4, 1503.3, 1472.4, 1364.7, 1277.7, 1255.3, 1233.7, 1189.7, 1151.0, 1019.6, 926.0, 846.6, 749.5, 733.4.

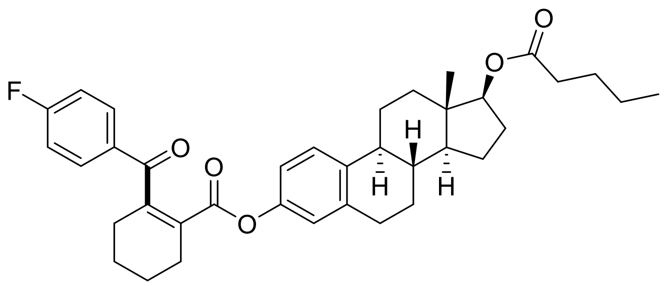

(8R,9S,13S,14S,17S)-13-methyl-17-(pentanoyloxy)-7,8,9,11,12,13,14,15,16,17-decahydro-6H-cyclopenta[a]phenanthren-3-yl 2-(4-fluorobenzoyl)cyclohex-1-ene-1-carboxylate (**3FF**). The reaction was carried out according to the general procedure **A** on 0.2 mmol scale (24 h); purified by flash column chromatography on SiO_2 (eluent: petroleum ether/ethyl acetate) to afford **5h**, 52.8 mg, 45%, light yellow oil; Rf = 0.2 (petroleum ether/ethyl acetate 10:1). ^{1}H NMR (400 MHz, Chloroform-*d*) δ 7.91 (dd, J = 8.5, 5.5 Hz, 2H), 7.12 (t, J = 8.7 Hz, 3H), 6.48 (d, J = 8.5 Hz,1H), 6.42 (s, 1H), 4.67 (t, J = 8.4 Hz, 1H), 2.76 – 2.71 (m, 2H), 2.60 – 2.57 (m, 2H), 2.45 – 2.41 (m, 2H), 2.30 (t, J = 7.5 Hz, 2H), 2.24 – 2.12 (m, 3H), 1.87 – 1.80 (m, 6H), 1.75 – 1.46 (m, 5H), 1.43 – 1.19 (m, 7H), 0.92 (t, J = 7.3 Hz, 3H), 0.79 (s, 3H). ^{13}C NMR (101 MHz, Chloroform-*d*) δ 196.86 , 173.84 , 165.75 (d, J = 255.1 Hz), 164.76 , 150.96 , 147.80 , 137.93 , 137.80 , 131.74 , 131.28 (d, J = 9.5 Hz), 127.41 , 126.18 , 121.02 , 118.06 , 115.81 (d, J = 21.9 Hz), 82.27 , 49.70 , 43.83 , 42.80 , 38.07 , 36.77 , 34.25 , 29.33 , 29.05 , 27.51 , 27.13 , 26.87 , 25.92 , 24.72 , 23.18 , 22.20 , 21.59 , 21.25 , 13.68 , 11.97 . ^{19}F NMR (376 MHz, Chloroform-*d*) δ -104.92 . HRMS (ESI) Calculated for $C_{37}H_{43}FO_5$ $[M+H]^+$: 587.3167, found: 587.3167. IR ν (neat, cm^{-1}): 2931.9, 2870.6, 1728.2, 1671.8, 1597.2, 1493.8, 1255.4, 1226.8, 1176.2, 1151.3, 1022.0, 915.5, 847.0, 732.9.

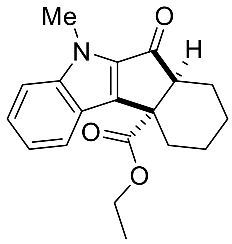

ethyl 5-methyl-6-oxo-6,6a,7,8,9,10-hexahydroindeno[2,1-b]indole-10a(5H)-carboxylate (**13**). The reaction was carried out according to the general procedure **A** on 0.2 mmol scale (24 h); purified by flash column chromatography on SiO_2 (eluent: petroleum ether/ethyl acetate) to afford **3ii**, 36.7 mg, 70%, light yellow solid; mp = 140-142 °C; Rf = 0.5 (petroleum ether/ethyl acetate 10:1).^{1}H NMR (400 MHz, Chloroform-*d*) δ 7.90 (d, J = 8.1 Hz, 1H), 7.43 – 7.36 (m, 2H), 7.21 – 7.17 (m, 1H), 4.31 – 4.19 (m, 2H), 3.93 (s, 3H), 3.61 (dd, J = 6.5, 4.7 Hz, 1H), 2.58 – 2.52 (m, 1H), 2.18 – 2.11 (m, 1H), 1.98 – 1.89 (m, 1H), 1.82 – 1.76 (m, 1H), 1.63 – 1.55 (m, 1H), 1.54 – 1.43 (m, 1H), 1.41 – 1.34 (m, 2H), 1.31 (t, J = 7.1 Hz, 3H). ^{13}C NMR (101 MHz, Chloroform-*d*) δ 195.01 , 174.22 , 144.56 , 144.12 , 137.13 , 126.59 , 122.75 , 122.22 , 120.48 , 110.97 , 61.35 , 55.15 , 49.09 , 32.72 , 30.20 , 22.05 ,

19.86 , 19.16 , 14.25 . HRMS (ESI) Calculated for $C_{19}H_{21}NO_3$ $[M+H]^+$: 312.1594, found: 312.1592. IR ν (neat, cm^{-1}): 2935.0, 2863.6, 1729.9, 1688.4, 1496.8, 1452.5, 1203.8, 1090.8, 1019.3, 806.3, 739.1.

2.5 Investigation of the reaction mechanism

2.5.1 Radical inhibition experiments with TEMPO

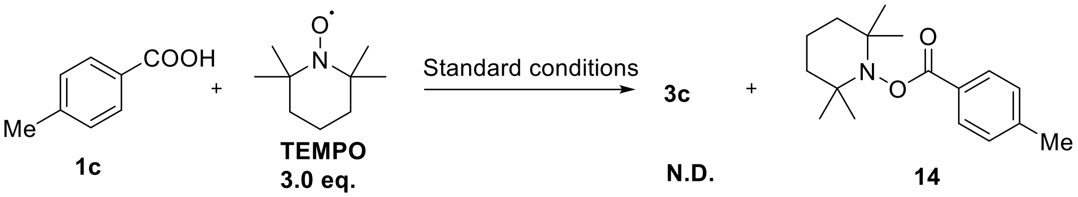

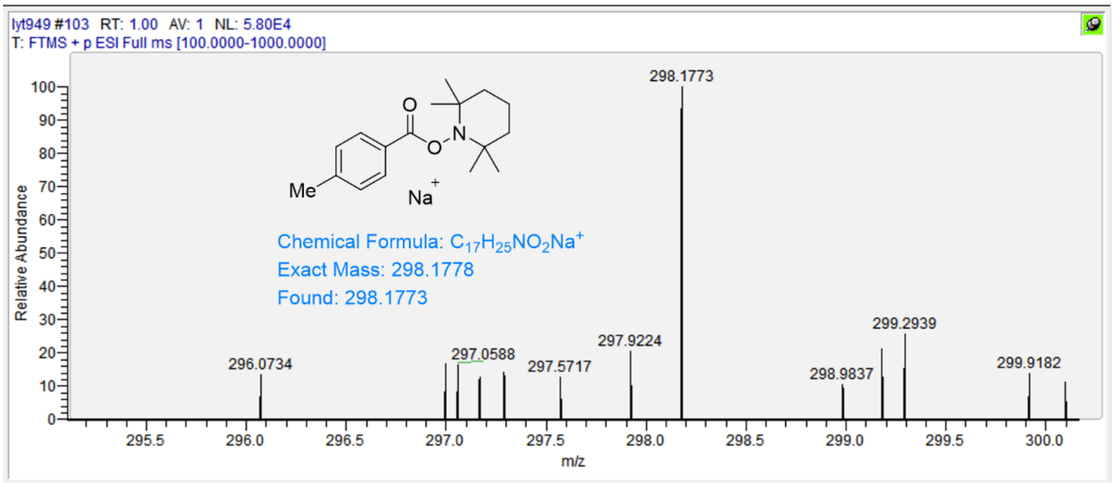

Supplementary Figure 3. HRMS data of the reaction mixture.

The reaction was completely inhibited by TEMPO. The compound **14** detected by HRMS indicated that the reaction probably proceeded via a free acyl radical process.

2.5.2 Luminescence quenching experiment

The luminescence quenching experiment was taken using a F-7000 FL Spectrophotometer (Hitachi, Japan). The experiments were carried out in 1 x 10^{-6} mol/L of $[Ir\{dF(CF_3)ppy\}_2\{dtbbpy\}]PF_6$ in CH_3CN at 25 °C. The excitation wavelength was 315 nm and the emission intensity was collected at 475 nm. The concentrations of quenchers (**1a** and Ph_3P) in MeCN were 0.3, 0.6, 1.0, 1.5, 2.1 mM.

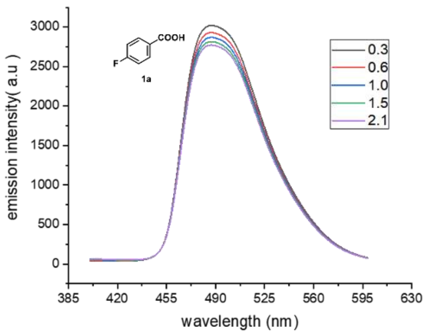

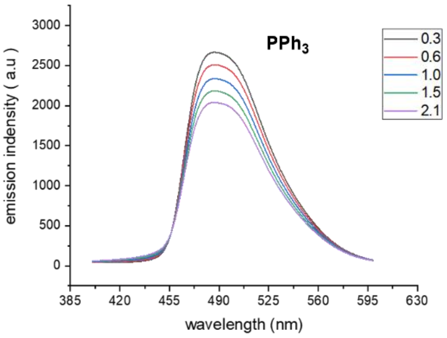

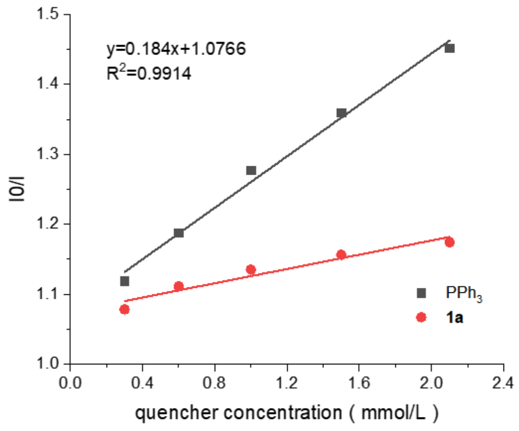

Supplementary Figure 4. The data of fluorescence quenching of $[Ir\{dF(CF_3)ppy\}_2\{dtbbpy\}]PF_6$* by **1a** and PPh_3.

To determine whether a reductive or oxidative quenching cycle is operative in the reaction, fluorescence quenching studies were conducted. Based on the above data, photoexcited $[Ir\{dF(CF_3)ppy\}_2\{dtbbpy\}]PF_6$* can be quenched by Ph_3P, involving a reductive quenching cycle.

2.5.3 Quantum yield measurement

The quantum yield (Φ) was determined by the known ferrioxalate actinometry method. A ferrioxalate actinometry solution was prepared by following the Hammond variation of the Hatchard and Parker procedure outlined in Handbook of Photochemistry. The actinometrysolutions (1mL) were irradiated with two 45 W blue LEDs for specified time intervals (30 s, 60 s, 90 s, 120 s, and 150 s). The UV-Vis spectra is shown in Fig.1a. Based on the data, we got the graph (Fig.1b) between the number of moles of products (y axis) and time (x axis). Then, the irradiated light intensity was estimated to 1.48 x 10^{-8} einstein S^{-1} by using $K_3[Fe(C_2O_4)_3]$ as an actinometer. For five clean tubes, according to the general procedure, the 0.2 mmol scale model reaction solution was irradiated with two 45 W blue LEDs for specified time intervals (30 min, 60 min, 90 min, 120 min and 150 min). The moles of products formed were determined by GC yield with acetophenone as reference standard. The number of moles of products (y axis) per unit time is related to the number of photons (x axis, calculated from the light intensity) (Fig.1c). The slope gives the quantum yield (Φ) of the photoreaction, 0.2928 (29%).

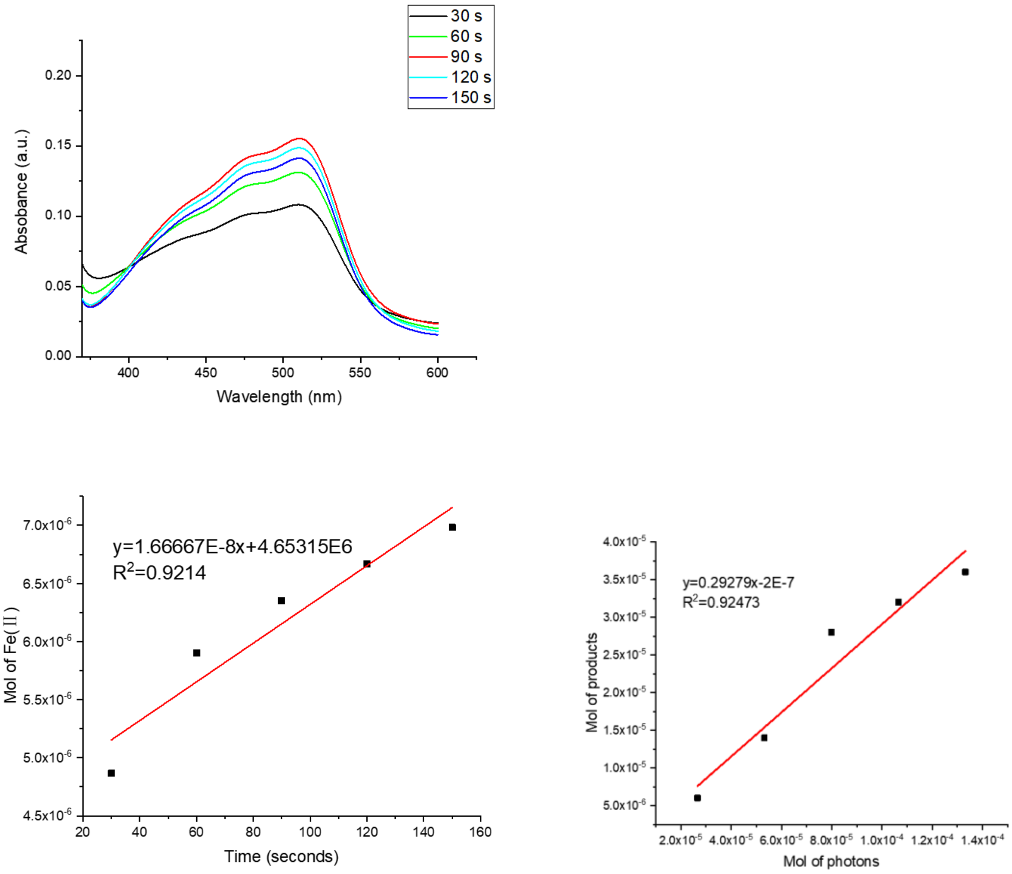

Supplementary Figure 5. The UV-Vis spectra and data of quantum yield measurement.

In order to determine whether a radical-chain reaction is involved, the quantum yield measurement was conducted, and gives the quantum yield (Φ) of the photoreaction, 29%. Thus, a radical chain pathway is less likely.

2.5.4 Kinetic experiments

2.5.4.1 The total reaction profile

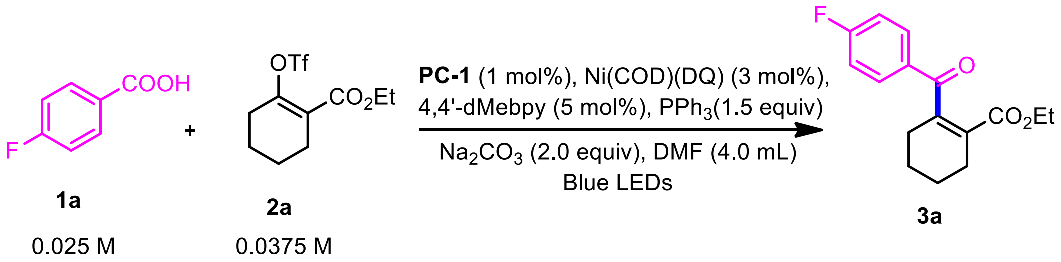

A stirring bar, Ni(COD)(DQ) (3.0 mol%), 4,4′-di-methyl-butyl-2,2′-bipyridine (5.0 mol%) and DMF (2.0 mL) were successively added to a vial (2.0 mL). The vial was stirred until the resulting mixture become homogeneous (about 20 min). Photocatalyst Ir[dF(CF_3)ppy]$_2$(dtbbpy)PF_6 (1 mol%), aromatic carboxylic acid (0.1 mmol, 1.0 equiv), triflates (0.15 mmol, 1.5 equiv), Ph_3P (0.15 mmol, 1.5 equiv), and Na_2CO_3 (0.2 mmol, 2.0 equiv) were added to an 4.0 mL screw-cap vial equipped with a magnetic stirring bar. Subsequently, the homogenous solution was syringed into the vial. Then add DMF (2.0 mL) to the vial. The vial was then sealed and placed ~5 cm from 2 × 45 W blue LEDs. The reaction mixture was stirred

at room temperature (air-condition was used to keep the temperature is 25 °C or so). At 1, 2, 3, 4, 5, 6, 7, 8, 9, 10, 11, 12, 13, 14, 15, 16, 18, 20, 22, 24, 26, 28, 30, 32, 34, 36, 38, 40, 45, 50, 55, 60, 70, 80, 90, 100, 110, 120, 180, 300, 420, 720 min, 20 μL the reaction mixture was carefully taken out by micro-syringe into 2.0 mL vial. Then 1.0 ml EA was added into the vial. The reaction mixture was analyzed by GC-MS after filtration.

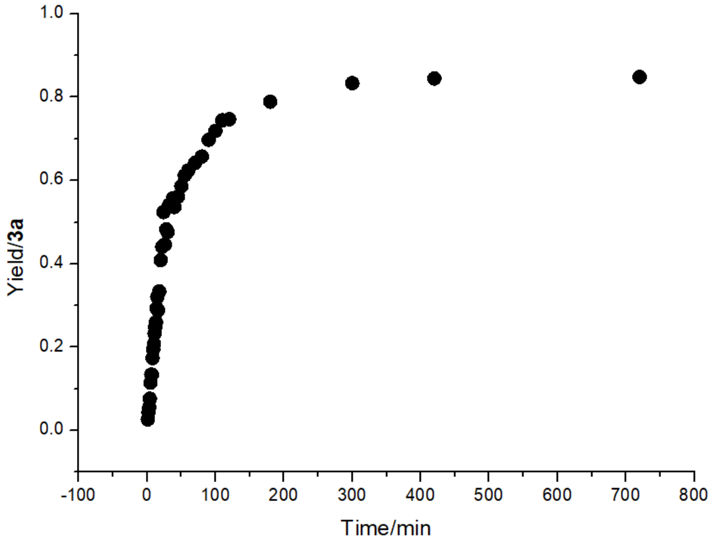

Supplementary Figure 6. The total reaction profile

2.5.4.2 Dependence of the reaction rate on concentration of Ni(COD)(DQ)

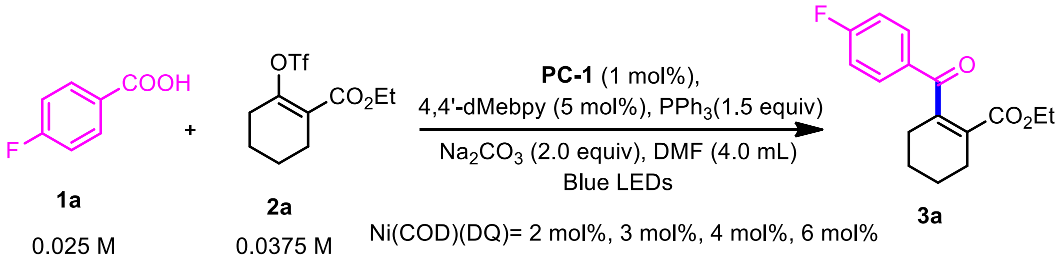

A stirring bar, Ni(COD)(DQ) (2 mol%, 3 mol%, 4 mol% or 6 mol%), 4,4′-di-methyl-butyl-2,2′-bipyridine [1.67 times of Ni(COD)(DQ)] and DMF (2.0 mL) were successively added to a vial (2.0 mL). The vial was stirred until the resulting mixture become homogeneous (about 20 min). Photocatalyst Ir[dF(CF_3)ppy]$_2$(dtbbpy)PF_6 (1 mol%), aromatic carboxylic acid (0.1 mmol, 1.0 equiv), triflates (0.15 mmol, 1.5 equiv), Ph_3P (0.15 mmol, 1.5 equiv), and Na_2CO_3 (0.2 mmol, 2.0 equiv) were added to an 4.0 mL screw-cap vial equipped with a magnetic stirring bar. Subsequently, the homogenous solution was syringed into the vial. Then add DMF (2.0 mL) to the vial. The vial was then sealed and placed ~5 cm from 2 × 45 W blue LEDs. The reaction mixture was stirred at room temperature (air-condition was used to keep the temperature is 25 °C or so). At 1, 2, 3, 4, 5, 6, 7, 8, 9, 10, 11, 12, 13, 14, 15 min, 20 μL the reaction mixture was carefully taken out by micro-syringe into 2.0 mL vial. Then 1.0 ml EA was added into the vial. The reaction mixture was analyzed by GC-MS after filtration.

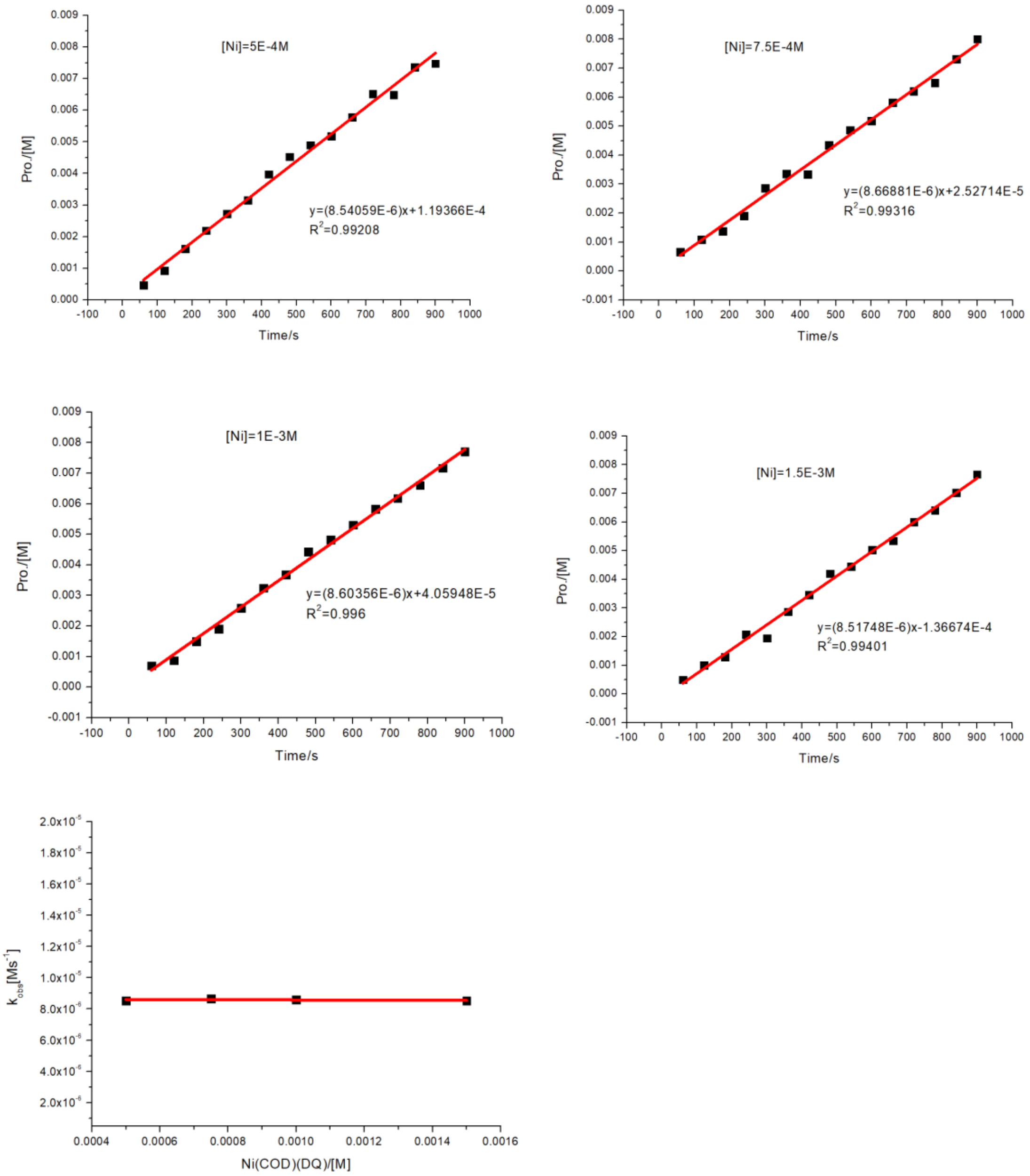

Supplementary Figure 7. Dependence of the reaction rate on loading of [Ni]

The plot of *Kobs* vs [Ni] suggests a zero-order kinetic dependence in [Ni].

2.5.4.3 Dependence of the reaction rate on concentration of **PC-1**

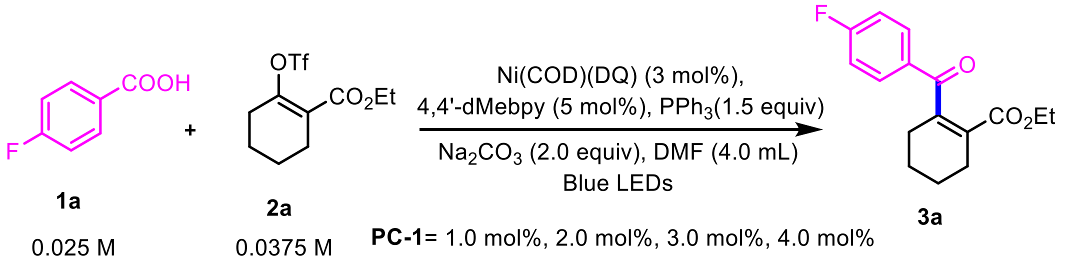

A stirring bar, Ni(COD)(DQ) (3.0 mol%), 4,4′-di-methyl-butyl-2,2′-bipyridine (5.0 mol%) and DMF (2.0 mL) were successively added to a vial (2.0 mL). The vial was stirred until the

resulting mixture become homogeneous (about 20 min). Photocatalyst Ir[dF(CF_3)ppy]$_2$(dtbbpy)PF_6 (1.0 mol%, 2.0 mol%, 3.0 mol% or 4.0 mol%), aromatic carboxylic acid (0.1 mmol, 1.0 equiv), triflates (0.15 mmol, 1.5 equiv), Ph_3P (0.15 mmol, 1.5 equiv), and Na_2CO_3 (0.2 mmol, 2.0 equiv) were added to an 4.0 mL screw-cap vial equipped with a magnetic stirring bar. Subsequently, the homogenous solution was syringed into the vial. Then add DMF (2.0 mL) to the vial. The vial was then sealed and placed ~5 cm from 2 × 45 W blue LEDs. The reaction mixture was stirred at room temperature (air-condition was used to keep the temperature is 25 °C or so). At 1, 2, 3, 4, 5, 6, 7, 8, 9, 10, 11, 12, 13, 14, 15 min, 20 μL the reaction mixture was carefully taken out by micro-syringe into 2.0 mL vial. Then 1.0 ml EA was added into the vial. The reaction mixture was analyzed by GC-MS after filtration.

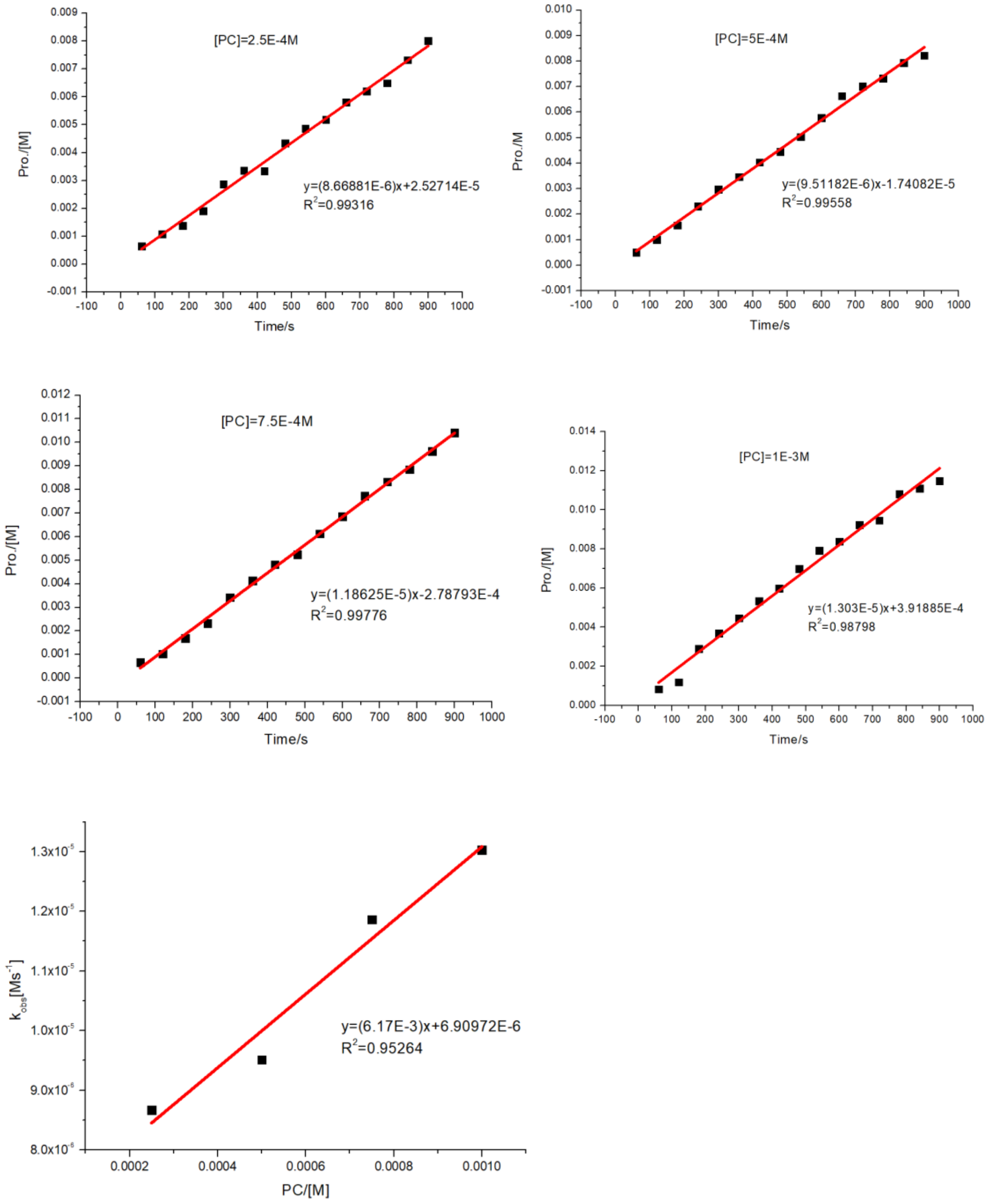

Supplementary Figure 8. Dependence of the reaction rate on **[PC-1]**

The plot of *Kobs* vs [**PC**] displayed a linear relationship in [**PC**], which should suggest a first-order kinetic dependence in [**PC**].

2.5.4.4 Dependence of the reaction rate on concentration of **1a**

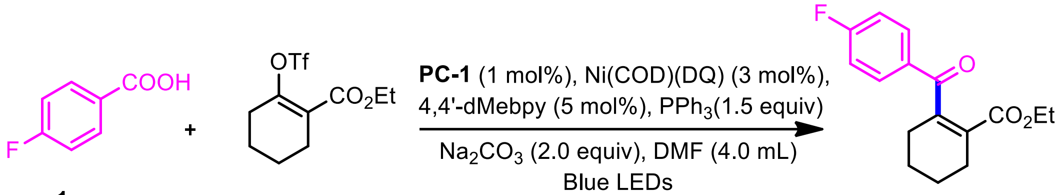

1a **2a** **3a**

1a	2a
0.0125 M	0.0375 M
0.01875 M	
0.025 M	
0.03125 M	

A stirring bar, Ni(COD)(DQ) (3.0 mol%), 4,4′-di-methyl-butyl-2,2′-bipyridine (5.0 mol%) and DMF (2.0 mL) were successively added to a vial (2.0 mL). The vial was stirred until the resulting mixture become homogeneous (about 20 min). Photocatalyst Ir[dF(CF_3)ppy]$_2$(dtbbpy)PF_6 (1 mol%), aromatic carboxylic acid (0.05 mmol, 0.075 mmol, 0.1 mmol, or 0.125 mmol), triflates (0.15 mmol, 1.5 equiv), Ph_3P (0.15 mmol, 1.5 equiv), and Na_2CO_3 (0.2 mmol, 2.0 equiv) were added to an 4.0 mL screw-cap vial equipped with a magnetic stirring bar. Subsequently, the homogenous solution was syringed into the vial. Then add DMF (2.0 mL) to the vial. The vial was then sealed and placed ~5 cm from 2 × 45 W blue LEDs. The reaction mixture was stirred at room temperature (air-condition was used to keep the temperature is 25 °C or so). At 1, 2, 3, 4, 5, 6, 7, 8, 9, 10, 11, 12, 13, 14, 15 min, 20 μL the reaction mixture was carefully taken out by micro-syringe into 2.0 mL vial. Then 1.0 ml EA was added into the vial. The reaction mixture was analyzed by GC-MS after filtration.

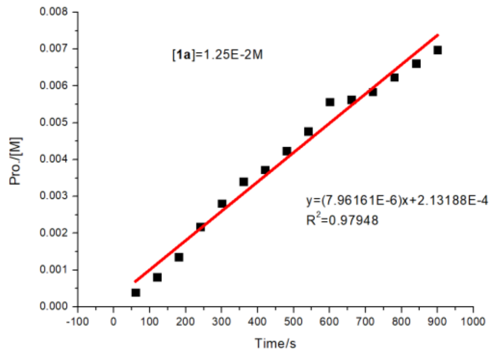

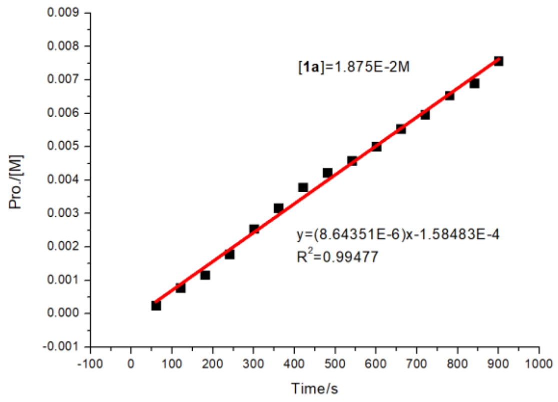

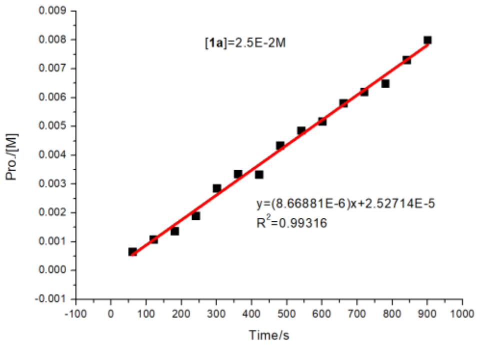

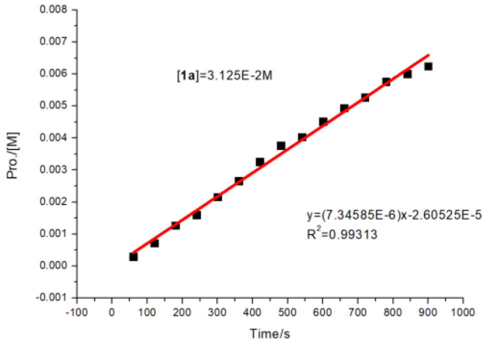

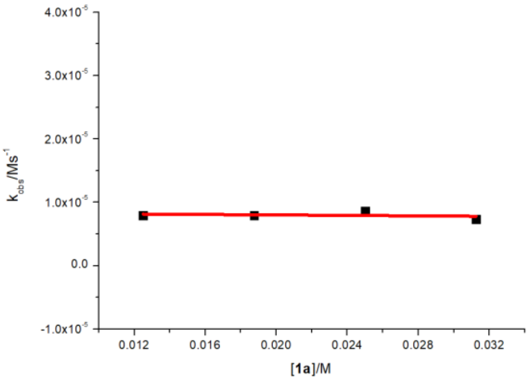

Supplementary Figure 9. Dependence of the reaction rate on [**1a**]

The plot of *Kobs* vs [**1a**] shows the same reaction rate regardless of the initial concentrations of **1a**, which would suggest a zero-order kinetic dependence in [**1a**].

2.5.4.5 Dependence of the reaction rate on concentration of **2a**

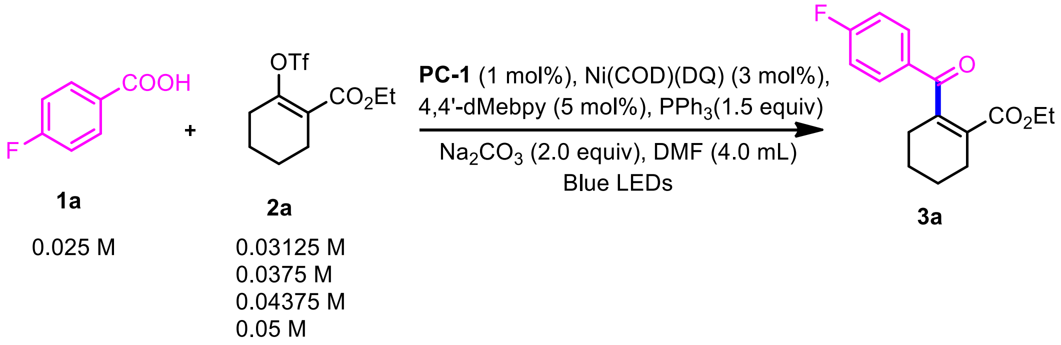

A stirring bar, Ni(COD)(DQ) (3.0 mol%), 4,4′-di-methyl-butyl-2,2′-bipyridine (5.0 mol%) and DMF (2.0 mL) were successively added to a vial (2.0 mL). The vial was stirred until the resulting mixture become homogeneous (about 20 min). Photocatalyst Ir[dF(CF_3)ppy]$_2$(dtbbpy)PF_6 (1 mol%), aromatic carboxylic acid (0.1 mmol, 1.0 equiv), triflates (0.125 mmol, 0.15 mmol, 0.175 mmol, or 0.2 mmol), Ph_3P (0.15 mmol, 1.5 equiv), and Na_2CO_3 (0.2 mmol, 2.0 equiv) were added to an 4.0 mL screw-cap vial equipped with a magnetic stirring bar. Subsequently, the homogenous solution was syringed into the vial. Then add DMF (2.0 mL) to the vial. The vial was then sealed and placed ~5 cm from 2 × 45 W blue LEDs. The reaction mixture was stirred at room temperature (air-condition was used to keep the temperature is 25 °C or so). At 1, 2, 3, 4, 5, 6, 7, 8, 9, 10, 11, 12, 13, 14, 15 min, 20 μL the reaction mixture was carefully taken out by micro-syringe into 2.0 mL vial. Then 1.0 ml EA was added into the vial. The reaction mixture was analyzed by GC-MS after filtration.

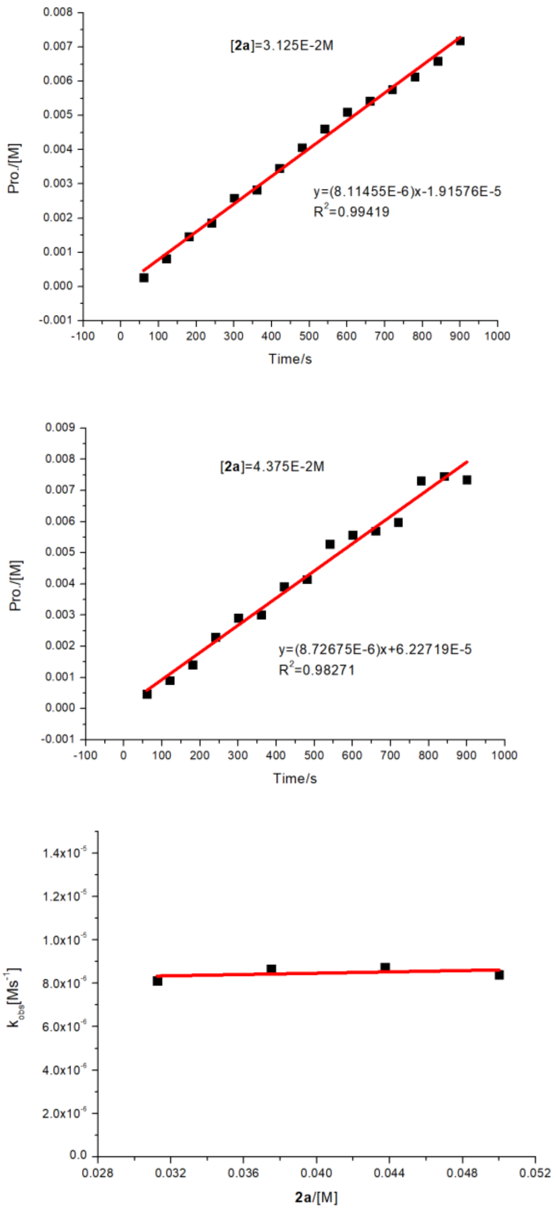

Supplementary Figure 10. Dependence of the reaction rate on [**2a**]

The plot of *Kobs* vs [**2a**] shows the same reaction rate regardless of the initial concentrations of **2a**, which would suggest a zero-order kinetic dependence in [**2a**].

2.5.4.6 Dependence of the reaction rate on concentration of PPh_3

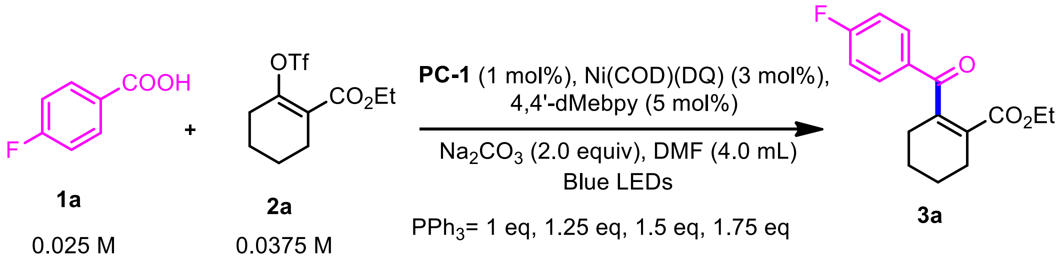

A stirring bar, Ni(COD)(DQ) (3.0 mol%), 4,4′-di-methyl-butyl-2,2′-bipyridine (5.0 mol%) and DMF (2.0 mL) were successively added to a vial (2.0 mL). The vial was stirred until the resulting mixture become homogeneous (about 20 min). Photocatalyst

Ir[dF(CF_3)ppy]$_2$(dtbbpy)PF_6 (1 mol%), aromatic carboxylic acid (0.1 mmol, 1.0 equiv), triflates (0.15 mmol, 1.5 equiv), Ph_3P (0.1 mmol, 0.125 mmol, 0.15 mmol, or 0.175 mmol), and Na_2CO_3 (0.2 mmol, 2.0 equiv) were added to an 4.0 mL screw-cap vial equipped with a magnetic stirring bar. Subsequently, the homogenous solution was syringed into the vial. Then add DMF (2.0 mL) to the vial. The vial was then sealed and placed ~5 cm from 2 × 45 W blue LEDs. The reaction mixture was stirred at room temperature (air-condition was used to keep the temperature is 25 °C or so). At 1, 2, 3, 4, 5, 6, 7, 8, 9, 10, 11, 12, 13, 14, 15 min, 20 μL the reaction mixture was carefully taken out by micro-syringe into 2.0 mL vial. Then 1.0 ml EA was added into the vial. The reaction mixture was analyzed by GC-MS after filtration.

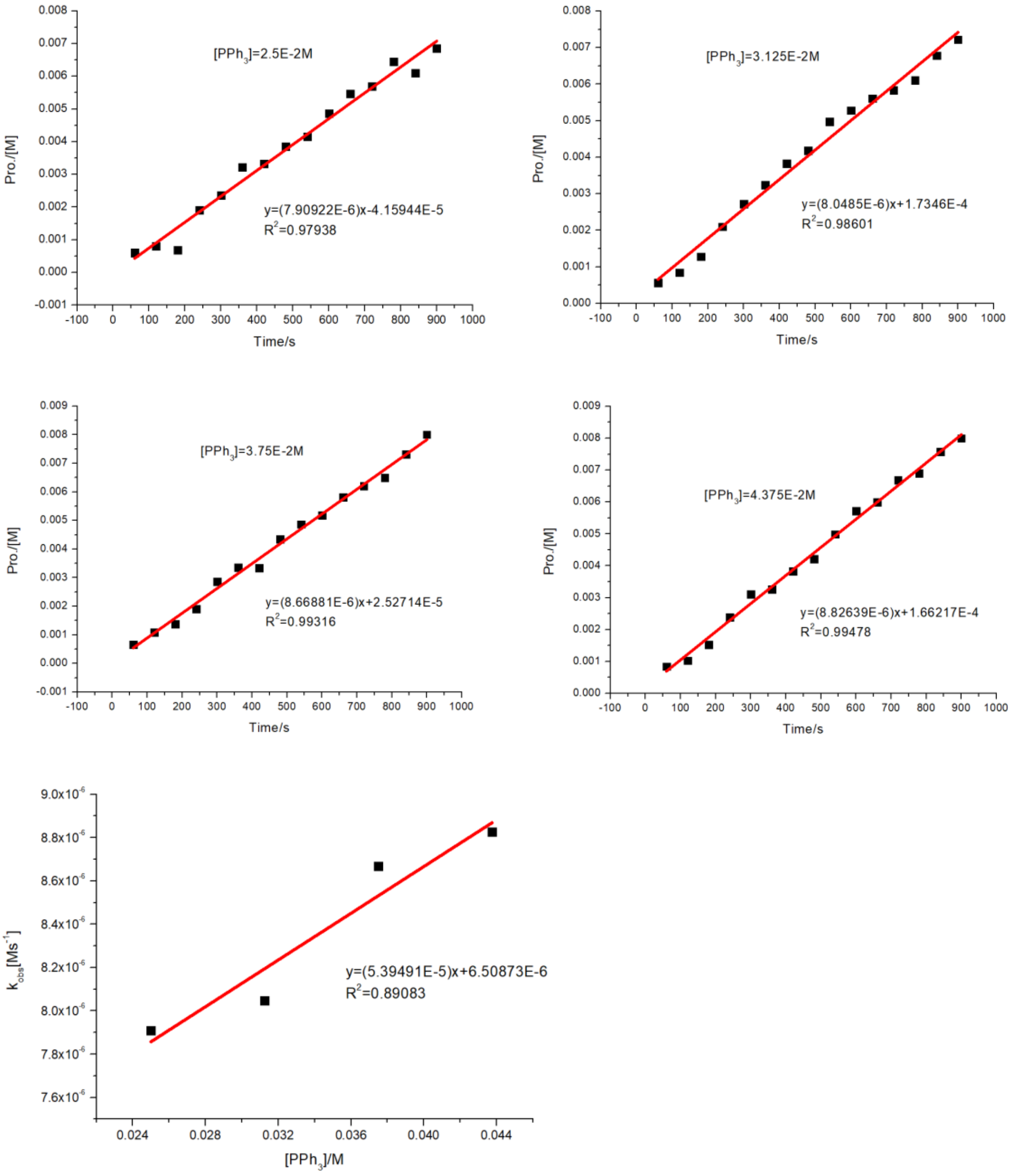

Supplementary Figure 11. Dependence of the reaction rate on [PPh_3]

The plot of *K*obs vs [PPh_3] displayed a linear relationship in [PPh_3], which should suggest a first-order kinetic dependence in [PPh_3].

The plots of *Kobs* suggest a zero-order kinetic dependence in [Ni], [**2a**], [**1a**](**1a** probably exists

in the form of salt), and suggest a first-order kinetic dependence in [PC-1], and [PPh_3]. Generating acyl radical probably is the rate determining step of the reaction.

2.5.5 The reactivity of acyl radical from 1a and 2a towards Ni(0)

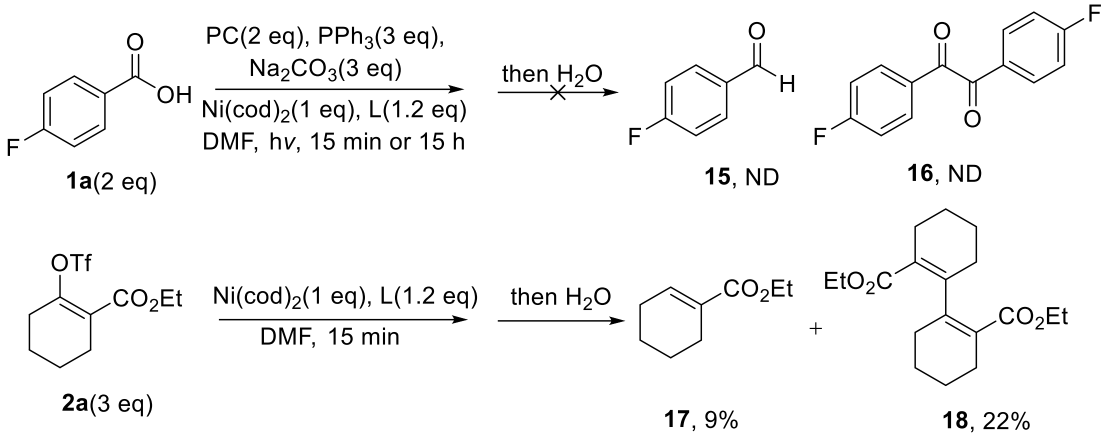

Supplementary Figure 12. The reactivity towards Ni(0)

A stirring bar, $Ni(cod)_2$ (1.0 equiv, 0.05 mmol), 4,4′-di-methyl-butyl-2,2′-bipyridine (2.0 equiv) and DMF (2.0 mL) were successively added to a vial (2.0 mL) in glovebox and was stirred for 2 h. Photocatalyst Ir[dF(CF_3)ppy]$_2$(dtbbpy)PF_6 (1.0 equiv), aromatic carboxylic acid (2.0 equiv), Ph_3P (3.0 eq), and Na_2CO_3 (3.0 equiv) were added to an 4.0 mL screw-cap vial equipped with a magnetic stirring bar. Subsequently, the homogenous solution was syringed into the vial. The vial was then sealed and placed ~5 cm from 2 × 45 W blue LEDs. The reaction mixture was stirred at room temperature (air-condition was used to keep the temperature is 25 °C or so). After 15 min or 15 h, 2.0 mL water and 2.0 mL EA were added. Then the organic phase was analyzed by GC-MS after filtration. Neither **15** nor **16** was detected.

A stirring bar, $Ni(cod)_2$ (1.0 equiv, 0.05 mmol), 4,4′-di-methyl-butyl-2,2′-bipyridine (2.0 equiv) and DMF (2.0 mL) were successively added to a vial (2.0 mL) in glovebox and was stirred for 2 h. Triflates **2a** (3.0 eq) was syringed into the vial. After 15 min, 2.0 mL water, 2.0 mL EA, and mesitylene (1.0 eq) were added. Then the organic phase was analyzed by GC-MS after filtration. 9% yield of protonation product **17** and 20% yield of self-coupling product **18** were formed in 15 min.

The stoichiometric reaction of Ni(0) with **1a** showed neither protonation product **15** nor self-coupling product **16** was formed in 15 min or 15 h. However, stoichiometric reaction of Ni(0) with **2a** showed that 9% yield of protonation product **17** and 22% yield of self-coupling product **18** were formed in 15 min. This result suggests a reaction pathway involving **2a** firstly reacts with nickel to form Ni(II) and then undergoes radical addition with acyl radical from **1a**(Path **a**).

2.5.6 Sequential addition experiments

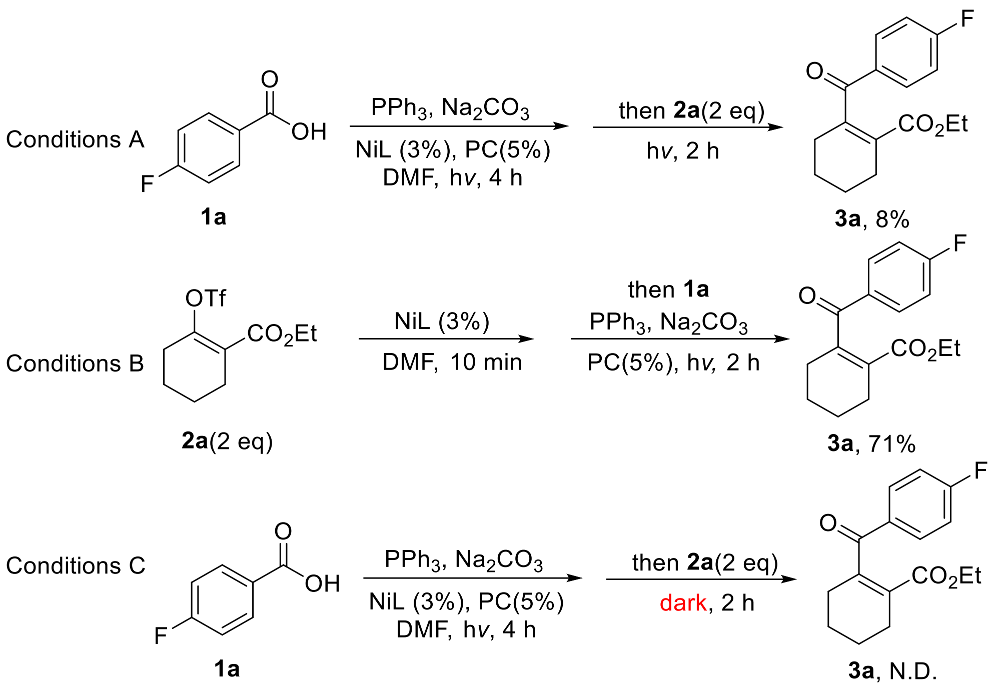

Supplementary Figure 13. Sequential addition experiments

Conditions A:

A stirring bar, Ni(COD)(DQ) (3.0 mol%), 4,4′-di-methyl-butyl-2,2′-bipyridine (6.0 mol%) and DMF (2.0 mL) were successively added to a vial (2.0 mL). The vial was stirred until the resulting mixture become homogeneous (about 20 min). Photocatalyst Ir[dF(CF_3)ppy]$_2$(dtbbpy)PF_6 (5 mol%), aromatic carboxylic acid (0.1 mmol, 1.0 equiv), Ph_3P (0.15 mmol, 1.5 equiv), and Na_2CO_3 (0.2 mmol, 2.0 equiv) were added to an 4.0 mL screw-cap vial equipped with a magnetic stirring bar. Subsequently, the homogenous solution was syringed into the vial. Then add DMF (2.0 mL) to the vial. The vial was then sealed and placed ~5 cm from 2 × 45 W blue LEDs. The reaction mixture was stirred at room temperature (air-condition was used to keep the temperature is 25 °C or so) for 4 hours. Then triflates (0.15 mmol, 1.5 equiv) was added. After 2 hours, 2.0 mL water, 2.0 mL EA, and biphenyl (1.0 equiv) were added. Then the organic phase was analyzed by GC after filtration.

Conditions B:

A stirring bar, Ni(COD)(DQ) (3.0 mol%), 4,4′-di-methyl-butyl-2,2′-bipyridine (6.0 mol%) and DMF (2.0 mL) were successively added to a vial (2.0 mL). The vial was stirred until the resulting mixture become homogeneous (about 20 min). Then triflates (0.15 mmol, 1.5 equiv) was added and the reaction mixture was stirred for 10 minutes.

Photocatalyst Ir[dF(CF_3)ppy]$_2$(dtbbpy)PF_6 (5 mol%), aromatic carboxylic acid (0.1 mmol, 1.0 equiv), Ph_3P (0.15 mmol, 1.5 equiv), and Na_2CO_3 (0.2 mmol, 2.0 equiv) were added to an 4.0 mL screw-cap vial equipped with a magnetic stirring bar.

Subsequently, the reaction mixture containing **2a** was syringed into the vial. Then add DMF (2.0 mL) to the vial. The vial was then sealed and placed ~5 cm from 2 × 45 W blue LEDs. The reaction mixture was stirred at room temperature (air-condition was used to keep the temperature is 25 °C or so). After 2 hours, 2.0 mL water, 2.0 mL EA, and biphenyl (1.0 equiv) were added. Then the organic phase was analyzed by GC after filtration.

Conditions C:

A stirring bar, Ni(COD)(DQ) (3.0 mol%), 4,4′-di-methyl-butyl-2,2′-bipyridine (6.0 mol%) and DMF (2.0 mL) were successively added to a vial (2.0 mL). The vial was stirred until the resulting mixture become homogeneous (about 20 min). Photocatalyst Ir[dF(CF$_3$)ppy]$_2$(dtbbpy)PF$_6$ (5 mol%), aromatic carboxylic acid (0.1 mmol, 1.0 equiv), Ph$_3$P (0.15 mmol, 1.5 equiv), and Na$_2$CO$_3$ (0.2 mmol, 2.0 equiv) were added to an 4.0 mL screw-cap vial equipped with a magnetic stirring bar. Subsequently, the homogenous solution was syringed into the vial. Then add DMF (2.0 mL) to the vial. The vial was then sealed and placed ~5 cm from 2 × 45 W blue LEDs. The reaction mixture was stirred at room temperature (air-condition was used to keep the temperature is 25 °C or so) for 4 hours. Then triflates (0.15 mmol, 1.5 equiv) was added, and put in dark. After 2 hours, 2.0 mL water, 2.0 mL EA, and biphenyl (1.0 equiv) were added. Then the organic phase was analyzed by GC after filtration.

We wished to experimentally control the sequence of the two processes, oxidative addition and radical addition, which can be achieved by varying the order of how the reaction components are added. By delayed addition of the **2a** after pre-stirring of **1a** and the catalysts (Conditions A), the acyl radical species could be pregenerated, driving the reaction to the radical addition first (path **b**), whereas addition of the **2a** first, then radical addition with **1a**(Conditions B) will enforce the mechanism where the oxidative addition occurs first (path **a**). The resulting 8% yield of the product **3a** under Condition A demonstrates radical addition firstly is unfavorable. The higher yield (71%) of **3a** under Condition B indicates than oxidation addition with **2b** firstly is more reasonable (Path **a**). Moreover, delayed addition of the **2a** and then reacting in dark for 2 hours (Conditions C) would lead to no product, which might suggest that radical addition to Ni(0) is less likely (Path **b**).

2.5.7 Other PCs and substrates

2.5.7.1 Other PCs

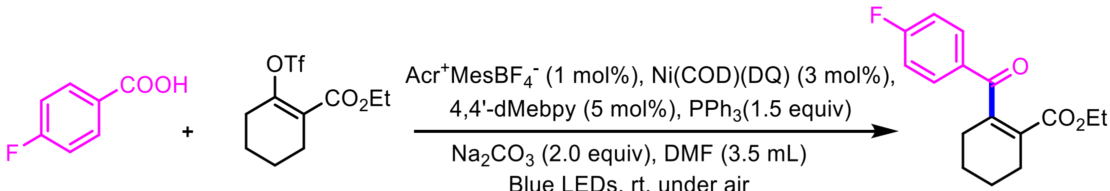

1a **2a** **3a,** N.D.

Operations are under air.

A stirring bar, Ni(COD)(DQ) (3.0 mol%), 4,4′-di-methyl -2,2′-bipyridine (5.0 mol%) and DMF (2.0 mL) were successively added to a vial (2.0 mL). The vial was stirred until the resulting mixture become homogeneous (about 20 min). Photocatalyst Acr$^+$MesBF4$^-$ (1.0 mol%), aromatic carboxylic acid **1a** (0.2 mmol, 1.0 equiv), **2a** (0.3 mmol, 1.5 equiv), Ph$_3$P (0.3 mmol, 1.5 equiv), and Na$_2$CO$_3$ (0.4 mmol, 2.0 equiv) were added to an 3.5 mL screw-cap vial equipped with a magnetic stirring bar. Subsequently, the homogenous solution was syringed into the vial. Then add DMF (1.5 mL) to the vial. The vial was then sealed and placed ~5 cm from 2 × 45 W blue LEDs. The reaction mixture was stirred for 24 h at room temperature (air-condition was used to keep the temperature is 25 °C or so). After completion, the reaction mixture was

removed from the light. 4.0 mL water and 4.0 mL EA were added. Then the organic phase was analyzed by GC-MS after filtration. **3a** was not detected.

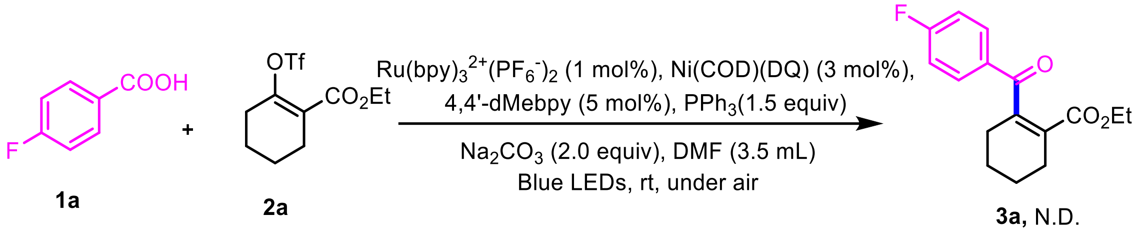

Operations are under air.

A stirring bar, Ni(COD)(DQ) (3.0 mol%), 4,4′-di-methyl -2,2′-bipyridine (5.0 mol%) and DMF (2.0 mL) were successively added to a vial (2.0 mL). The vial was stirred until the resulting mixture become homogeneous (about 20 min). Photocatalyst $Ru(bpy)_3^{2+}(PF_6^-)_2$ (1.0 mol%), aromatic carboxylic acid **1a** (0.2 mmol, 1.0 equiv), **2a** (0.3 mmol, 1.5 equiv), Ph_3P (0.3 mmol, 1.5 equiv), and Na_2CO_3 (0.4 mmol, 2.0 equiv) were added to an 3.5 mL screw-cap vial equipped with a magnetic stirring bar. Subsequently, the homogenous solution was syringed into the vial. Then add DMF (1.5 mL) to the vial. The vial was then sealed and placed ~5 cm from 2 × 45 W blue LEDs. The reaction mixture was stirred for 24 h at room temperature (air-condition was used to keep the temperature is 25 °C or so). After completion, the reaction mixture was removed from the light. 4.0 mL water and 4.0 mL EA were added. Then the organic phase was analyzed by GC-MS after filtration. **3a** was not detected.

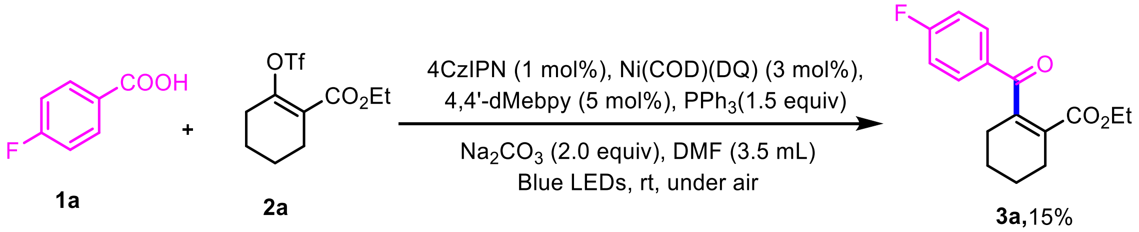

All operations are under air.

A stirring bar, Ni(COD)(DQ) (3.0 mol%), 4,4′-di-methyl-butyl-2,2′-bipyridine (5.0 mol%) and DMF (2.0 mL) were successively added to a vial (2.0 mL). The vial was stirred until the resulting mixture become homogeneous (about 20 min). Photocatalyst, (1 mol%), aromatic carboxylic acid (0.2 mmol, 1.0 equiv), triflates (0.3 mmol, 1.5 equiv), Ph_3P (0.3 mmol, 1.5 equiv), and Na_2CO_3 (0.4 mmol, 2.0 equiv) were added to an 3.5 mL screw-cap vial equipped with a magnetic stirring bar. Subsequently, the homogenous solution was syringed into the vial. Then add DMF (1.5 mL) to the vial. The vial was then sealed and placed ~5 cm from 2 × 45 W blue LEDs. The reaction mixture was stirred for 24 h at room temperature (air-condition was used to keep the temperature is 25 °C or so). After completion, the reaction mixture was removed from the light. The solvent was removed and the residue was purified by flash chromatography on silica gel to afford the corresponding products **3a** in 15% yield.

2.5.7.2 Other substrates

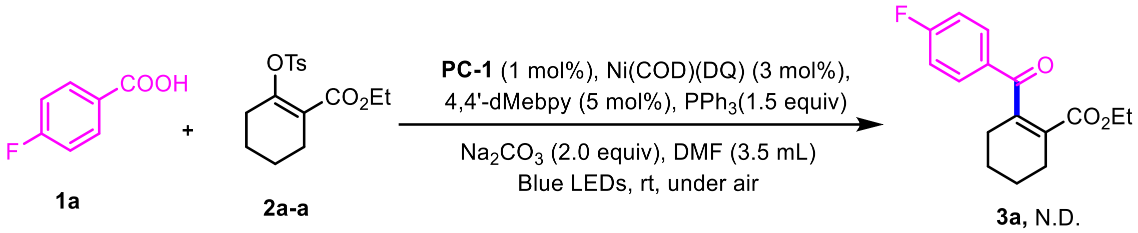

Operations are under air.

A stirring bar, Ni(COD)(DQ) (3.0 mol%), 4,4′-di-methyl -2,2′-bipyridine (5.0 mol%) and DMF (2.0 mL) were successively added to a vial (2.0 mL). The vial was stirred until the resulting mixture become homogeneous (about 20 min). Photocatalyst **PC-1** (1.0 mol%), aromatic carboxylic acid **1a** (0.2 mmol, 1.0 equiv), **2a-a** (0.3 mmol, 1.5 equiv), Ph_3P (0.3 mmol, 1.5 equiv), and Na_2CO_3 (0.4 mmol, 2.0 equiv) were added to an 3.5 mL screw-cap vial equipped with a magnetic stirring bar. Subsequently, the homogenous solution was syringed into the vial. Then add DMF (1.5 mL) to the vial. The vial was then sealed and placed ~5 cm from 2 × 45 W blue LEDs. The reaction mixture was stirred for 24 h at room temperature (air-condition was used to keep the temperature is 25 °C or so). After completion, the reaction mixture was removed from the light. 4.0 mL water and 4.0 mL EA were added. Then the organic phase was analyzed by GC-MS after filtration. **3a** was not detected.

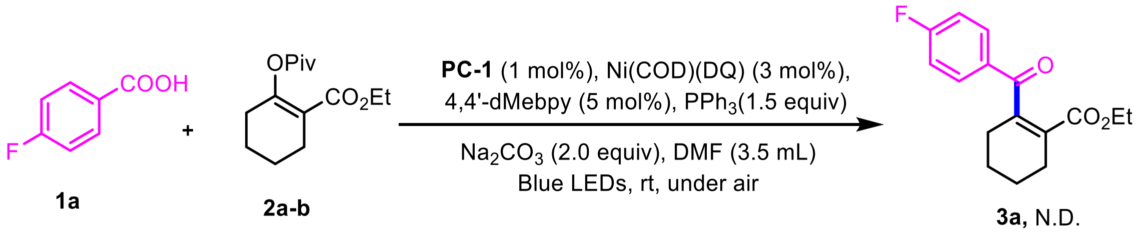

Operations are under air.

A stirring bar, Ni(COD)(DQ) (3.0 mol%), 4,4′-di-methyl -2,2′-bipyridine (5.0 mol%) and DMF (2.0 mL) were successively added to a vial (2.0 mL). The vial was stirred until the resulting mixture become homogeneous (about 20 min). Photocatalyst **PC-1** (1.0 mol%), aromatic carboxylic acid **1a** (0.2 mmol, 1.0 equiv), **2a-b** (0.3 mmol, 1.5 equiv), Ph_3P (0.3 mmol, 1.5 equiv), and Na_2CO_3 (0.4 mmol, 2.0 equiv) were added to an 3.5 mL screw-cap vial equipped with a magnetic stirring bar. Subsequently, the homogenous solution was syringed into the vial. Then add DMF (1.5 mL) to the vial. The vial was then sealed and placed ~5 cm from 2 × 45 W blue LEDs. The reaction mixture was stirred for 24 h at room temperature (air-condition was used to keep the temperature is 25 °C or so). After completion, the reaction mixture was removed from the light. 4.0 mL water and 4.0 mL EA were added. Then the organic phase was analyzed by GC-MS after filtration. **3a** was not detected.

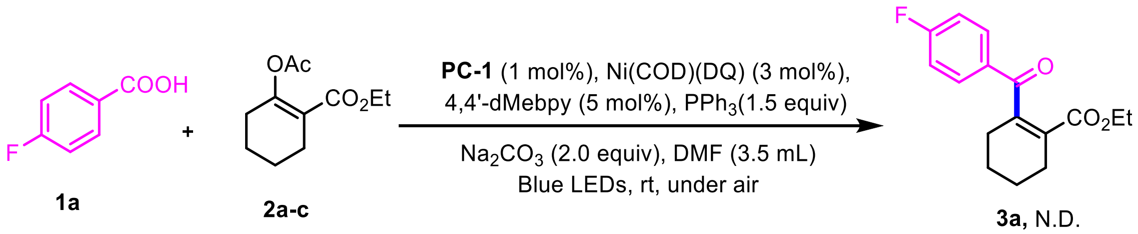

Operations are under air.

A stirring bar, Ni(COD)(DQ) (3.0 mol%), 4,4′-di-methyl -2,2′-bipyridine (5.0 mol%) and DMF (2.0 mL) were successively added to a vial (2.0 mL). The vial was stirred until the resulting mixture become homogeneous (about 20 min). Photocatalyst **PC-1** (1.0 mol%), aromatic carboxylic acid **1a** (0.2 mmol, 1.0 equiv), **2a-c** (0.3 mmol, 1.5 equiv), Ph_3P (0.3 mmol, 1.5 equiv), and Na_2CO_3 (0.4 mmol, 2.0 equiv) were added to an 3.5 mL screw-cap vial equipped with a magnetic stirring bar. Subsequently, the homogenous solution was syringed into the vial. Then add DMF (1.5 mL) to the vial. The vial was then sealed and placed ~5 cm from 2 × 45 W blue LEDs. The reaction mixture was stirred for 24 h at room temperature (air-condition was used to keep the temperature is 25 °C or so). After completion, the reaction mixture was removed from the light. 4.0 mL water and 4.0 mL EA were added. Then the organic phase was analyzed by GC-MS after filtration. **3a** was not detected.

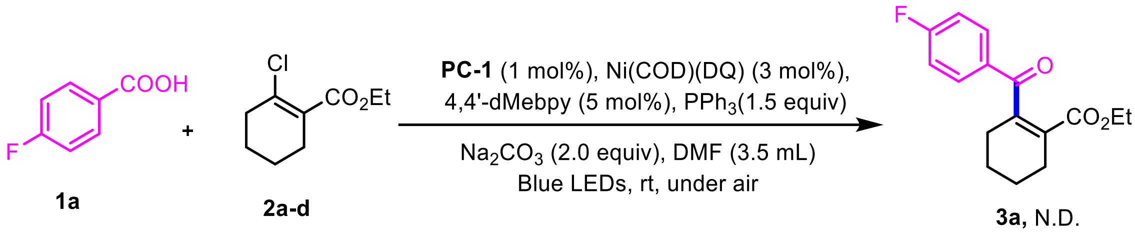

Operations are under air.

A stirring bar, Ni(COD)(DQ) (3.0 mol%), 4,4′-di-methyl -2,2′-bipyridine (5.0 mol%) and DMF (2.0 mL) were successively added to a vial (2.0 mL). The vial was stirred until the resulting mixture become homogeneous (about 20 min). Photocatalyst **PC-1** (1.0 mol%), aromatic carboxylic acid **1a** (0.2 mmol, 1.0 equiv), **2a-d** (0.3 mmol, 1.5 equiv), Ph_3P (0.3 mmol, 1.5 equiv), and Na_2CO_3 (0.4 mmol, 2.0 equiv) were added to an 3.5 mL screw-cap vial equipped with a magnetic stirring bar. Subsequently, the homogenous solution was syringed into the vial. Then add DMF (1.5 mL) to the vial. The vial was then sealed and placed ~5 cm from 2 × 45 W blue LEDs. The reaction mixture was stirred for 24 h at room temperature (air-condition was used to keep the temperature is 25 °C or so). After completion, the reaction mixture was removed from the light. 4.0 mL water and 4.0 mL EA were added. Then the organic phase was analyzed by GC-MS after filtration. **3a** was not detected.

2.6 Synthetic application

2.6.1 Gram-scale reaction

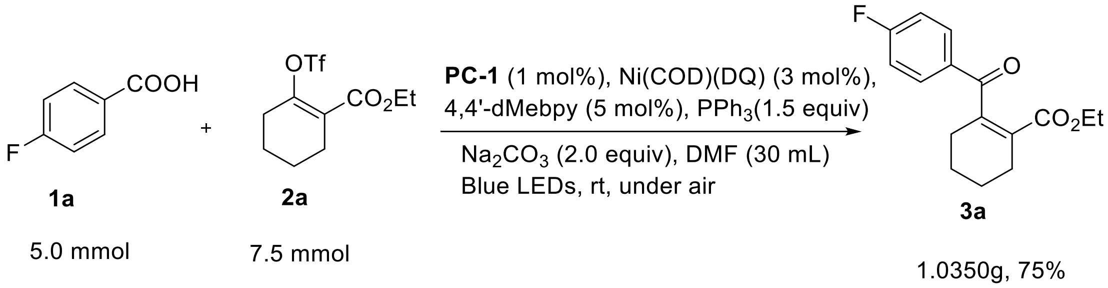

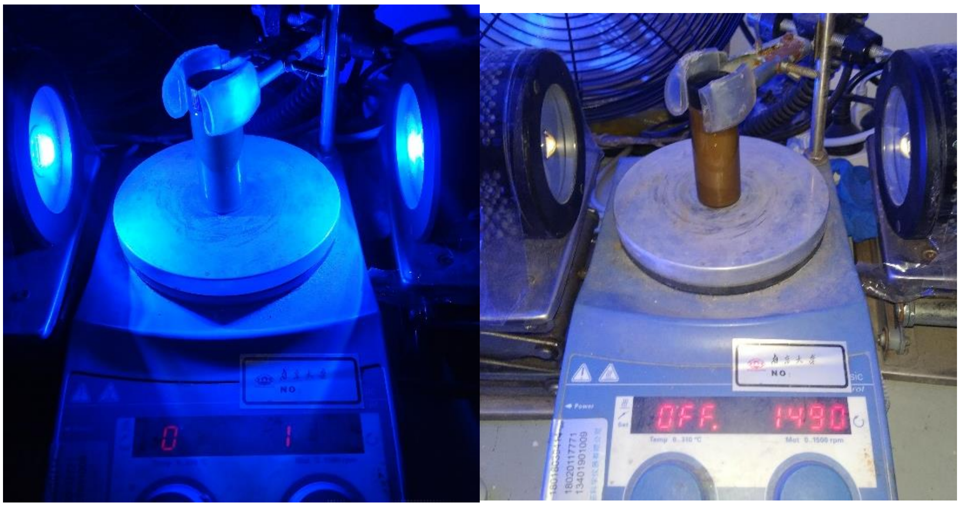

Supplementary Figure 14. (left) Reaction going on; (right) Reaction having finished.

Procedure for the gram-scale reaction

All operations are under air.

An 30 mL glass vial was added Ni(COD)(DQ) (50.0 mg, 3.0 mol%) and 4,4'-dimethyl-2,2'-bipyridine (46.0 mg, 5.0 mol%) and dissolved in DMF (30.0 mL). The glass vial was stirred until the resulting mixture became homogenous solution (about 30 min). An 30 mL screw-cap vial equipped with a magnetic with stir bar was added aromatic 4-fluorobenzoic acid (700 mg, 5.0 mmol), triflate (2.27 g, 7.5 mmol), Ph_3P (1.97 g, 7.5 mmol), anhydrous Na_2CO_3 (1.06 g, 10.0 mmol) and $Ir[dF(CF_3)ppy]_2(dtbbpy)PF_6$ (56.1 mg, 1.0 mol%). Subsequently, the homogenous solution was syringed into the vial. The vial was then sealed and was placed at a distance (app. 5 cm) from 2 x 45 W blue LEDs, and the mixture was stirred for 48 h at room temperature (air condition was used to keep the temperature is 25 °C or so). After completion, the reaction mixture was removed from the light. The solvent was removed and the residue was purified by flash chromatography on silica gel to afford the corresponding products **3a** in 1.035 g with 75% yield.

2.6.2. Two steps "one pot" for synthesis of products

One-Pot Two-Steps Synthesis of **3a**

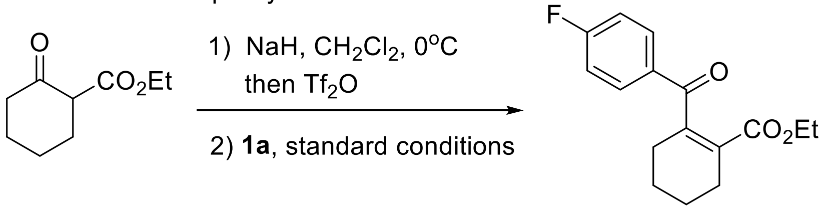

S2a **3a,** 81%

One-Pot Two-Steps Synthesis of *ethyl 2-(4-fluorobenzoyl)cyclohex-1-ene-1-carboxylate* ***3a***

Reaction was conducted under Argon atmosphere. A dry round-bottom flask equipped with a magnetic stirbar was charged with dry dichloromethane (30 mL) and the flask was cooled to 0 °C in an ice bath. Next, NaH 60% dispersion in mineral oil (960 mg, 24 mmol) was added in one portion and the suspension was stirred for 30 minutes. Then, a solution of the ethyl 2-oxocyclohexane-1-carboxylate (3.2 mL, 20 mmol) in dry dichloromethane (30 mL) was added

dropwise to the suspension at 0 °C. After stirring for 30 min, trifluoromethanesulfonic anhydride (4.0 mL, 24 mmol) in dry dichloromethane (30 mL) was added dropwise to the reaction. The reaction mixture was warmed to room temperature and stirred overnight. Then it was quenched with the addition of H_2O (20 mL) and the resulting layers were separated, and the aqueous phase was extracted with CH_2Cl_2 (3 x 30 mL). The combined organic phase was washed with brine (3 x 30 mL), dried over Na_2SO_4 and filtered. After concentration in vacuo, the residue was used in the next step without further purification. (crude product **2a**, 6.0 g, 99%).

Operations are under air. A stirring bar, Ni(COD)(DQ) (3.0 mol%), 4,4′-di-methyl -2,2′-bipyridine (5.0 mol%) and DMF (2.0 mL) were successively added to a vial (2.0 mL). The vial was stirred until the resulting mixture become homogeneous (about 20 min). Photocatalyst Ir[dF(CF_3)ppy]$_2$(dtbbpy)PF_6 (1.0 mol%), aromatic carboxylic acid **1a** (0.2 mmol, 1.0 equiv), crude product **2a** (0.3 mmol, 1.5 equiv), Ph_3P (0.3 mmol, 1.5 equiv), and Na_2CO_3 (0.4 mmol, 2.0 equiv) were added to an 3.5 mL screw-cap vial equipped with a magnetic stirring bar. Subsequently, the homogenous solution was syringed into the vial. Then add DMF (1.5 mL) to the vial. The vial was then sealed and placed ~5 cm from 2 × 45 W blue LEDs. The reaction mixture was stirred for 24 h at room temperature (air-condition was used to keep the temperature is 25 °C or so). After completion, the reaction mixture was removed from the light. The solvent was removed and the residue was purified by flash chromatography on silica gel to afford the corresponding products. (**3a**, 45.3 mg, 82%).

One-Pot Two-Steps Synthesis of **3jj**

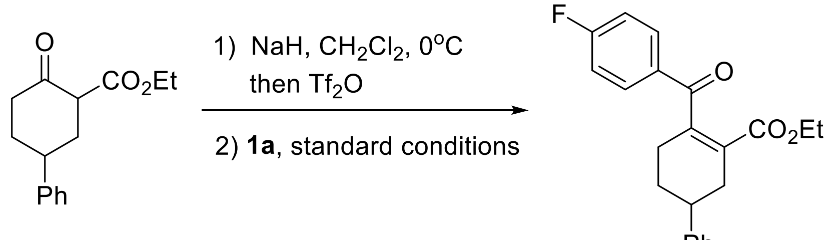

S2d

3jj, 66%

One-Pot Two-Steps Synthesis of *ethyl 4-(4-fluorobenzoyl)-1,2,5,6-tetrahydro-[1,1'-biphenyl]-3-carboxylate* **3jj**

Reaction was conducted under Argon atmosphere. A dry round-bottom flask equipped with a magnetic stirbar was charged with dry dichloromethane (30 mL) and the flask was cooled to 0 °C in an ice bath. Next, NaH 60% dispersion in mineral oil (170.3 mg, 4.3 mmol) was added in one portion and the suspension was stirred for 30 minutes. Then, a solution of the ethyl 2-oxo-5-phenylcyclohexane-1-carboxylate(**S2d**) (872.8 mg, 3.5 mmol) in dry dichloromethane (30 mL) was added dropwise to the suspension at 0 °C. After stirring for 30 min, trifluoromethanesulfonic anhydride (0.72 mL, 4.3 mmol) in dry dichloromethane (30 mL) was added dropwise to the reaction. The reaction mixture was warmed to room temperature and stirred overnight. Then it was quenched with the addition of H_2O (20 mL) and the resulting layers were separated, and the aqueous phase was extracted with CH_2Cl_2 (3 x 30 mL). The combined organic phase was washed with brine (3 x 30 mL), dried over Na_2SO_4 and filtered.

After concentration in vacuo, the residue was used in the next step without further purification. (crude product **2d**, 1.3320g, 99%).

Operations are under air. A stirring bar, Ni(COD)(DQ) (3.0 mol%), 4,4′-di-methyl -2,2′-bipyridine (5.0 mol%) and DMF (2.0 mL) were successively added to a vial (2.0 mL). The vial was stirred until the resulting mixture become homogeneous (about 20 min). Photocatalyst Ir[dF(CF_3)ppy]$_2$(dtbbpy)PF_6 (1.0 mol%), aromatic carboxylic acid **1a** (0.2 mmol, 1.0 equiv), crude product **2d** (0.3 mmol, 1.5 equiv), Ph_3P (0.3 mmol, 1.5 equiv), and Na_2CO_3 (0.4 mmol, 2.0 equiv) were added to an 3.5 mL screw-cap vial equipped with a magnetic stirring bar. Subsequently, the homogenous solution was syringed into the vial. Then add DMF (1.5 mL) to the vial. The vial was then sealed and placed ~5 cm from 2 × 45 W blue LEDs. The reaction mixture was stirred for 24 h at room temperature (air-condition was used to keep the temperature is 25 °C or so). After completion, the reaction mixture was removed from the light. The solvent was removed and the residue was purified by flash chromatography on silica gel to afford the corresponding products. (**3jj**, 46.5 mg, 66%).

One-Pot Two-Steps Synthesis of **3pp**

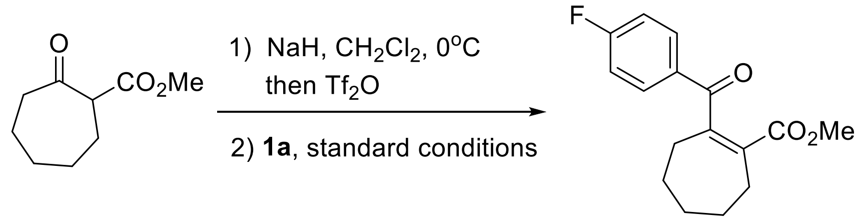

S2g **3pp**, 60%

One-Pot Two-Steps Synthesis of *methyl 2-(4-fluorobenzoyl)cyclohept-1-ene-1-carboxylate* **3pp**

Reaction was conducted under Argon atmosphere. A dry round-bottom flask equipped with a magnetic stirbar was charged with dry dichloromethane (30 mL) and the flask was cooled to 0 °C in an ice bath. Next, NaH 60% dispersion in mineral oil (190 mg, 4.8 mmol) was added in one portion and the suspension was stirred for 30 minutes. Then, a solution of the 2-oxocycloheptane-1-carboxylate (**S2g**) (674.5 mg, 4.0 mmol) in dry dichloromethane (30 mL) was added dropwise to the suspension at 0 °C. After stirring for 30 min, trifluoromethanesulfonic anhydride (0.8 mL, 4.8 mmol) in dry dichloromethane (30 mL) was added dropwise to the reaction. The reaction mixture was warmed to room temperature and stirred overnight. Then it was quenched with the addition of H_2O (20 mL) and the resulting layers were separated, and the aqueous phase was extracted with CH_2Cl_2 (3 x 30 mL). The combined organic phase was washed with brine (3 x 30 mL), dried over Na_2SO4 and filtered. After concentration in vacuo, the residue was used in the next step without further purification. (crude product **2g**, 1.2100 g, 99%).

Operations are under air. A stirring bar, Ni(COD)(DQ) (3.0 mol%), 4,4′-di-methyl -2,2′-bipyridine (5.0 mol%) and DMF (2.0 mL) were successively added to a vial (2.0 mL). The vial was stirred until the resulting mixture become homogeneous (about 20 min). Photocatalyst Ir[dF(CF_3)ppy]$_2$(dtbbpy)PF_6 (1.0 mol%), aromatic carboxylic acid **1a** (0.2 mmol, 1.0 equiv), crude product **2g** (0.3 mmol, 1.5 equiv), Ph_3P (0.3 mmol, 1.5 equiv), and Na_2CO_3 (0.4 mmol, 2.0 equiv) were added to an 3.5 mL screw-cap vial equipped with a magnetic stirring bar. Subsequently, the homogenous solution was syringed into the vial. Then add DMF (1.5 mL) to

the vial. The vial was then sealed and placed ~5 cm from 2 × 45 W blue LEDs. The reaction mixture was stirred for 24 h at room temperature (air-condition was used to keep the temperature is 25 °C or so). After completion, the reaction mixture was removed from the light. The solvent was removed and the residue was purified by flash chromatography on silica gel to afford the corresponding products. (**3pp**, 33.2 mg, 60%).

2.6.3. Derivation of product

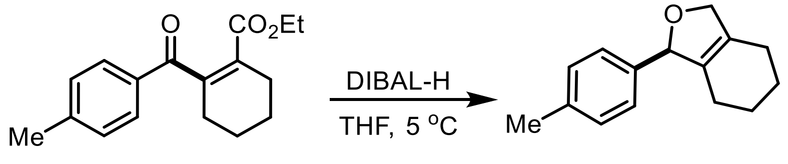

A solution of **3c** (272.3 mg, 1 mmol) in dry tetrahydrofuran (1 ml) is added dropwise to a stirred solution of diisobutylaluminium hydride (1.2 M in toluene, 0.4 mmol) at 5 ºC, with ice cooling. The excess reagent is decomposed by careful of methanol, the mixture is poured ton to ice. The aqueous layer is separated, saturated with sodium chloride, and extracted with ethyl acetate. The combined organic layers are dried with magnesium sulphate The solvent was removed and the residue was purified by flash chromatography on silica gel to afford the corresponding products.

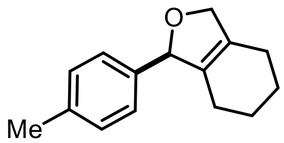

1-(p-tolyl)-1,3,4,5,6,7-hexahydroisobenzofuran (**14**). Affording **14** with 139.1 mg, 65%. ^{1}H NMR (400 MHz, Chloroform-*d*) δ 7.22 (d, *J* = 7.9 Hz, 2H), 7.13 (d, *J* = 7.8 Hz, 2H), 5.73 (s, 1H), 4.35 (d, *J* = 11.7 Hz, 1H), 4.09 (d, *J* = 11.7 Hz, 1H), 2.33 (s, 3H), 2.18 – 2.06 (m, 2H), 1.70 – 1.60 (m, 2H), 1.56 – 1.49 (m, 4H). ^{13}C NMR (101 MHz, Chloroform-*d*) δ 139.48 , 136.99 , 136.56 , 133.67 , 128.89 , 125.72 , 72.01 , 62.73 , 28.96 , 24.46 , 22.63 , 22.48 , 21.05 . MS(EI): m/z (%) = 91.1 (30), 119.0 (100), 214.2 (20) [M$^+$].

2.7 DFT calculations

Computational Methods:

Conformational searches were performed using the CREST conformer-rotamer ensemble sampling tool,[10] version 2.7.1 with xtb version 6.2 RC2 (SAW190805).[11] Conformers were then optimized in Gaussian 16 Rev. A.03 (sse4)[12] at the PBE0[13]-D3(BJ)[14]/def2-SVP[15], SMD[16](DMF) level of theory. All geometries were verified as stationary points on the potential energy surface and characterized as transition states or minima by frequency calculations. Single point energies were computed at the PBE0-D3(BJ)/def2-TZVP, SMD(DMF) level of theory. The hybrid functional PBE0-D3(BJ) was chosen as it is recommended for

thermochemistry calculations of transition-metal-containing systems.[17] Standard enthalpies and Gibbs free energies were calculated using GoodVibes version 3.0.1[18] with quasi-harmonic corrections applied to entropies[19] and enthalpies[20].

Scan Study of Radical Coupling

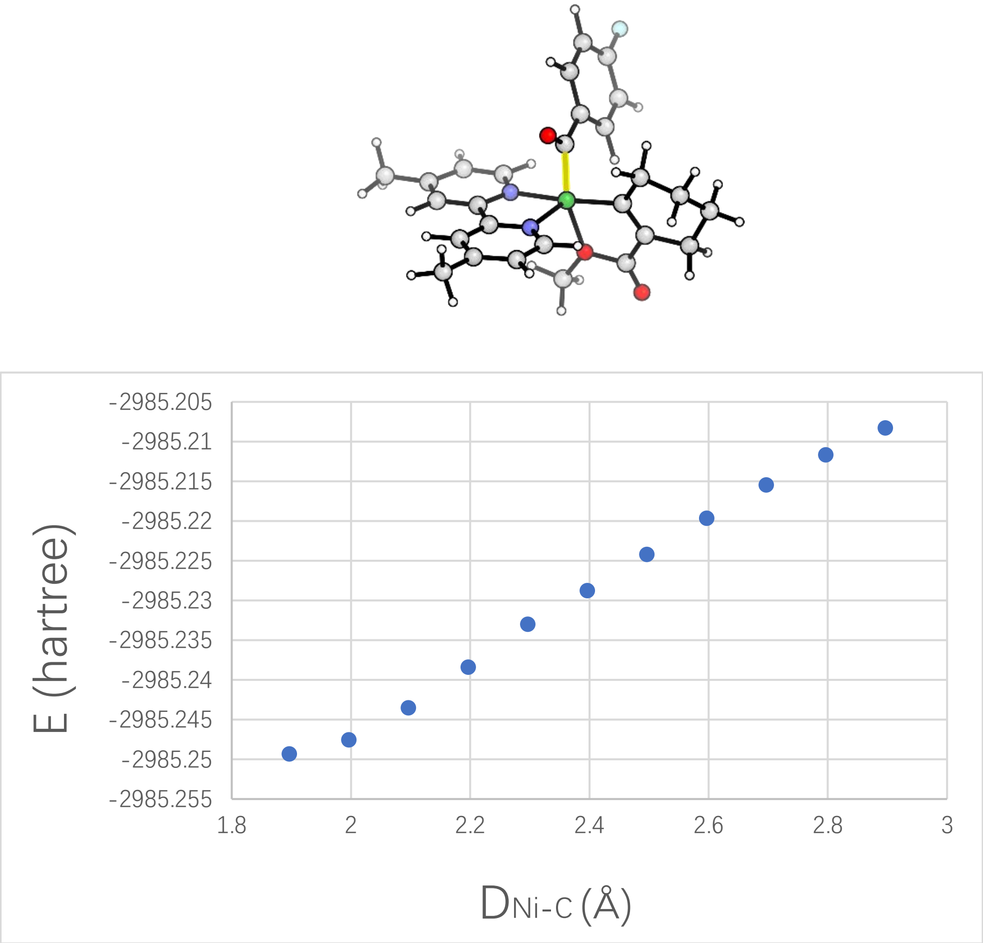

Supplementary Figure 15. Scanned electronic energy of the Ni-C bond. The corresponding bond is highlighted in the structure.

Potential Energy Surface of Isomerization and Reductive Elimination

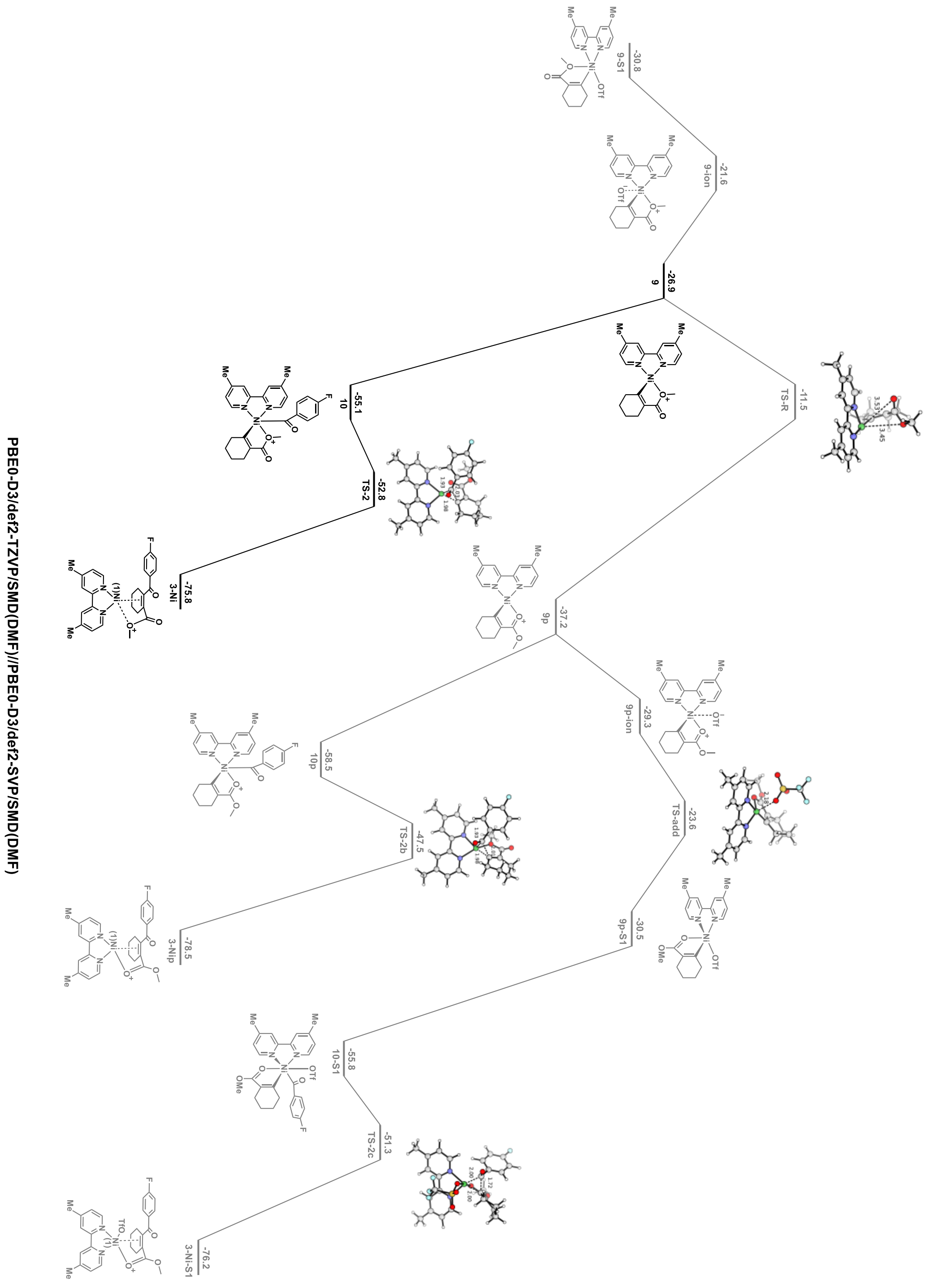

Supplementary Figure 16. Potential Energy Surface after **TS-1A**. Free energies are relative to **7**. Unlikely pathways are noted as gray.

Cartesian Coordinates of Optimized Geometries

Ni0-cod

E -311.780231

C	-1.15324900	-1.27241500	-0.50148300
H	-1.72308700	-1.90607500	-1.19356700
C	-1.68414300	-0.07125500	-0.22892700
H	-2.62203100	0.16963700	-0.74549100
C	-1.15324900	1.01617500	0.66563200
H	-0.75047900	0.60424400	1.60061700
H	-1.99561300	1.65915600	0.96506000
C	-0.11008400	1.91206600	-0.00849500
H	-0.58133700	2.41934600	-0.86952400
H	0.15894400	2.73355400	0.68253900
C	1.15324900	1.27241500	-0.50148300
H	1.72308700	1.90607500	-1.19356700
C	1.68414300	0.07125500	-0.22892700
H	2.62203100	-0.16963700	-0.74549100
C	1.15324900	-1.01617500	0.66563200
H	0.75047900	-0.60424400	1.60061700
H	1.99561300	-1.65915600	0.96506000
C	0.11008400	-1.91206600	-0.00849500
H	0.58133700	-2.41934600	-0.86952400
H	-0.15894400	-2.73355400	0.68253900

5

E -1554.74389080

C	5.26118600	0.30333500	-0.73000000
C	4.98117200	-1.06387000	-0.81709900
C	3.66688800	-1.48525800	-0.90901300
C	2.58525000	-0.54667300	-0.91650600
C	2.91900600	0.84277300	-0.83144400
C	4.23819800	1.25465400	-0.73642800
H	5.80623600	-1.78119700	-0.80918700
H	3.43444000	-2.55032300	-0.97251800
H	2.12172500	1.58797800	-0.84028800
H	4.49297600	2.31553900	-0.66757000
F	6.54446000	0.71144200	-0.64092300
C	1.25724200	-1.03783300	-1.00558700
O	0.83299500	-2.19642400	-1.01816000
O	0.28577700	0.03075800	-1.07035000
P	-0.99328100	0.06994900	-0.08962600
C	-0.57838200	-0.71091100	1.45678300
C	-1.38706900	-1.70786300	2.01571900

C	0.62111200	-0.32481400	2.07842000
C	-0.99977400	-2.31186800	3.20812700
H	-2.31326700	-2.00855300	1.52027300
C	0.99894900	-0.94209100	3.26814800
H	1.24867800	0.45809900	1.64410000
C	0.19187500	-1.93154700	3.83063300
H	-1.62958800	-3.08563800	3.65310200
H	1.93031100	-0.64834200	3.75796600
H	0.49400300	-2.41247500	4.76440600
C	-2.40356400	-0.71515900	-0.86424600
C	-3.67375900	-0.13567100	-0.74531300
C	-2.22695400	-1.92673600	-1.54741400
C	-4.77231200	-0.77745000	-1.31191900
H	-3.80545600	0.81011600	-0.21403400
C	-3.33399100	-2.55492900	-2.11152900
H	-1.22131000	-2.35362600	-1.62358000
C	-4.60212600	-1.98326100	-1.99317900
H	-5.76546100	-0.33105600	-1.22323100
H	-3.20569800	-3.49771400	-2.64884800
H	-5.46683800	-2.48118500	-2.43930300
C	-1.31483900	1.81326100	0.13679000
C	-1.63502200	2.32388000	1.40099700
C	-1.27051000	2.65769000	-0.98344600
C	-1.90376700	3.68330600	1.54285700
H	-1.67260100	1.66353500	2.27055100
C	-1.53936200	4.01372900	-0.82784300
H	-1.02312400	2.25795600	-1.96986400
C	-1.85551400	4.52611500	0.43226000
H	-2.15138600	4.08528100	2.52805700
H	-1.50179200	4.67574200	-1.69618000
H	-2.06654000	5.59212400	0.54831600

TS-0

E -1554.73566549

C	4.66146100	0.43902100	-0.43638000
C	4.60559500	-0.95624600	-0.38724900
C	3.46298000	-1.60895900	-0.80871800
C	2.32665400	-0.86512900	-1.27869400
C	2.43047400	0.55904800	-1.32758900
C	3.59084100	1.19197700	-0.91673600
H	5.46881300	-1.51811900	-0.02228800
H	3.41585100	-2.69928100	-0.76844400
H	1.58737100	1.13548300	-1.70250200

H	3.67769500	2.28037700	-0.96120500
F	5.77766500	1.06578700	-0.02567800
C	1.17308000	-1.61206800	-1.50918900
O	0.67001100	-2.60519000	-1.89932100
O	-0.32460100	-0.13758800	-1.66482700
P	-1.01425000	0.07953400	-0.31126800
C	0.07402300	-0.24575400	1.09573200
C	0.18557600	-1.54826700	1.60140000
C	0.93425200	0.75885700	1.55602000
C	1.15630200	-1.84182800	2.55565800
H	-0.47838300	-2.33763000	1.23988700
C	1.90327900	0.45915800	2.51303500
H	0.85642000	1.77493800	1.16212000
C	2.01701800	-0.83927400	3.00809900
H	1.24435300	-2.85879800	2.94540200
H	2.57546000	1.24391600	2.86827900
H	2.78123300	-1.07302000	3.75375800
C	-2.44548100	-1.01525200	-0.15136700
C	-3.20041900	-1.08682000	1.02658000
C	-2.79412000	-1.79240500	-1.26031500
C	-4.30296400	-1.93474100	1.08792200
H	-2.92515900	-0.48782000	1.89862800
C	-3.89914100	-2.64020600	-1.19130100
H	-2.18815900	-1.73058000	-2.16693700
C	-4.65274500	-2.71007200	-0.02018700
H	-4.89194000	-1.99373100	2.00647300
H	-4.17095400	-3.24999100	-2.05656000
H	-5.51829400	-3.37554300	0.03270700
C	-1.58469600	1.79026000	-0.18274300
C	-2.04875400	2.33246600	1.02269800
C	-1.57361700	2.56756500	-1.34591100
C	-2.50138100	3.64896300	1.05773600
H	-2.04907200	1.73203700	1.93604600
C	-2.02746000	3.88501700	-1.30315200
H	-1.20505400	2.13233000	-2.27795100
C	-2.49163400	4.42429700	-0.10368000
H	-2.86044000	4.07418500	1.99811500
H	-2.01691500	4.49299300	-2.21117600
H	-2.84605900	5.45774300	-0.07106300

6

E -443.857814484

C	1.71429300	-0.08852400	0.00000000
C	0.90783100	-1.22508400	0.00000000

C	-0.46890200	-1.05929600	-0.00000100
C	-1.02274400	0.23357600	-0.00000100
C	-0.18575400	1.35772600	0.00000000
C	1.19476000	1.20169800	0.00000000
H	1.36978200	-2.21459800	0.00000000
H	-1.12914300	-1.92976600	-0.00000100
H	-0.62843500	2.35670000	0.00000000
H	1.87230600	2.05772200	0.00000000
F	3.03475500	-0.24575900	0.00000000
C	-2.47562100	0.41919400	-0.00000100
O	-3.34756100	-0.38674700	0.00000100

$OPPh_3$

E -1110.88206220

O	-0.01389100	-0.16052600	2.40794300
P	-0.00260200	-0.03348600	0.90483200
C	1.40879500	-0.91132500	0.16641500
C	1.79284400	-0.76561100	-1.17281000
C	2.11016100	-1.78672700	1.00181500
C	2.86722000	-1.50001100	-1.67003900
H	1.26113800	-0.07120500	-1.82871800
C	3.18512900	-2.51971000	0.50000700
H	1.80393900	-1.87962000	2.04699300
C	3.56223000	-2.37785600	-0.83525000
H	3.16754100	-1.38365800	-2.71444700
H	3.73213100	-3.20244300	1.15534300
H	4.40578100	-2.95107900	-1.22872300
C	0.09690200	1.67421700	0.29281500
C	-1.06765100	2.40262800	0.01892700
C	1.34404300	2.30850000	0.20989900
C	-0.98388500	3.74529100	-0.34634500
H	-2.04624100	1.92038700	0.08783700
C	1.42400200	3.65058900	-0.15535900
H	2.25915400	1.75117300	0.42826500
C	0.26076200	4.36890100	-0.43603300
H	-1.89612100	4.30658600	-0.56365000
H	2.39988200	4.13792600	-0.22308100
H	0.32519100	5.42087900	-0.72590800
C	-1.50081900	-0.74950600	0.16259600
C	-1.77464400	-0.70603700	-1.21047400
C	-2.39273900	-1.38417800	1.03228700
C	-2.93488600	-1.29695700	-1.70488200
H	-1.08691600	-0.20503200	-1.89670500
C	-3.55347500	-1.97500900	0.53294800

H	-2.16293700	-1.40493600	2.10069400
C	-3.82396600	-1.93189800	-0.83428000
H	-3.14905300	-1.26065400	-2.77601300
H	-4.24974400	-2.46964300	1.21509000
H	-4.73372700	-2.39398500	-1.22640800

7

E -3504.42056754

Ni	0.67911900	-0.17052300	0.23342500
N	1.60858400	1.48635300	-0.03542900
N	2.43051600	-0.95133100	0.03610100
C	2.95280000	1.35664400	-0.10732900
C	1.07479300	2.70922700	-0.08332400
C	3.42375100	-0.04414600	-0.07040800
C	2.73239300	-2.25199700	0.04726800
C	3.78749500	2.46297900	-0.21959300
C	1.84859000	3.85733800	-0.19944300
H	-0.01459400	2.76604800	-0.02522800
C	4.75784000	-0.43171300	-0.15060800
C	4.04112000	-2.70907500	-0.03427300
H	1.88736400	-2.94494500	0.11031900
C	3.24125500	3.75145700	-0.26866100
H	4.86959000	2.33059400	-0.27070400
H	1.36047900	4.83357400	-0.23315000
C	5.09276000	-1.79036300	-0.13265900
H	5.54537000	0.31956700	-0.22935100
H	4.23848200	-3.78314600	-0.01853900
C	4.11866000	4.95198300	-0.41059100
C	6.51312700	-2.23804900	-0.24899800
H	4.50847600	5.01732300	-1.44011800
H	4.98916000	4.88754100	0.25925400
H	3.57079300	5.87958300	-0.19636900
H	6.76888000	-2.40445600	-1.30927500
H	6.67859300	-3.18730600	0.27996400
H	7.20721400	-1.48102500	0.14226600
C	-1.12357000	0.03617000	0.66707800
C	-0.76013000	-1.37789300	0.65982200
C	-0.72551500	-2.19041100	-0.54760800
O	-1.03122100	-1.52906100	-1.68973200
O	-0.42471600	-3.37413600	-0.57438500
C	-1.02162300	-2.28452800	-2.88028200
H	-1.73950400	-3.11808200	-2.83216500
H	-0.02168900	-2.69558000	-3.08948200
H	-1.31313100	-1.59653600	-3.68363300

C	-0.78456300	-2.19609800	1.93589600
C	-0.82330700	-1.35629600	3.20330600
H	0.07535800	-2.88422000	1.95446100
H	-1.68252800	-2.84331200	1.91235400
C	-1.85771300	-0.24791300	3.08225400
H	-1.03976400	-2.00355000	4.06868000
H	0.16842000	-0.90329400	3.38704500
C	-1.44903300	0.72264100	1.98401700
H	-1.97263700	0.29166100	4.03662000
H	-2.84118600	-0.68823400	2.83909000
H	-0.56254300	1.28511200	2.32698900
H	-2.23049000	1.47809400	1.81617900
O	-1.97453100	0.57601200	-0.40276300
S	-3.33892200	-0.08700500	-0.85467700
O	-3.61470800	-1.35805800	-0.21301800
O	-3.49143800	0.10206900	-2.28231400
C	-4.54917400	1.10612800	-0.10598700
F	-4.58896400	0.96367200	1.20940400
F	-4.21211400	2.35033600	-0.39726100
F	-5.75039000	0.85432700	-0.60020500

TS-1A

E -3504.41491070

Ni	0.78942700	-0.36995900	0.38457400
N	1.52315600	1.37348600	0.28046500
N	2.60957000	-0.93879200	0.01830200
C	2.86341300	1.41480500	0.12756500
C	0.83325900	2.51532100	0.36281900
C	3.48965800	0.08513800	-0.01921000
C	3.05238300	-2.18672200	-0.13556100
C	3.55042400	2.62375600	0.08682500
C	1.45651400	3.75405800	0.32176600
H	-0.25127900	2.41582400	0.44935900
C	4.84964800	-0.13264900	-0.20265600
C	4.39716400	-2.47738200	-0.32912000
H	2.29950400	-2.97870100	-0.10962800
C	2.84896700	3.82960700	0.19222100
H	4.63460300	2.63516900	-0.03477500
H	0.85230300	4.66120500	0.38916700
C	5.33265200	-1.43847300	-0.35971500
H	5.54296300	0.70970500	-0.22858100
H	4.71104300	-3.51549000	-0.45515600
C	3.55673300	5.14430600	0.18136900
C	6.79234400	-1.69701500	-0.53838200

H	4.57274000	5.05365900	-0.22630800
H	3.63898300	5.53483300	1.20965600
H	2.99849200	5.89018900	-0.40315200
H	7.22646300	-1.00917900	-1.27945100
H	6.98395800	-2.73128700	-0.85397700
H	7.32545000	-1.52451300	0.41130500
C	-0.87523700	-0.37686500	1.02096700
C	-0.75425200	-1.70043400	0.56271600
C	-0.62786500	-2.06658000	-0.85500500
O	-0.57991000	-1.00903900	-1.69344900
O	-0.56213200	-3.20897900	-1.25981900
C	-0.53083000	-1.29863700	-3.07536900
H	-1.42527500	-1.85585000	-3.39106500
H	0.36473300	-1.88577700	-3.33039000
H	-0.50233400	-0.33114500	-3.59028200
C	-1.09947700	-2.86410800	1.46962700
C	-1.34705600	-2.44660200	2.91362400
H	-0.31180500	-3.63240900	1.41264000
H	-2.01136800	-3.33529400	1.06108500
C	-2.18968000	-1.17947700	2.98306900
H	-1.83464900	-3.27298600	3.45469000
H	-0.38401900	-2.26157700	3.42322600
C	-1.42055600	-0.01884400	2.36182400
H	-2.45934600	-0.93807200	4.02356100
H	-3.13109400	-1.32681500	2.42588400
H	-0.57123400	0.26235800	3.00966200
H	-2.04346000	0.88152100	2.27223200
O	-2.06923200	0.77255900	-0.08258200
S	-3.17072400	0.22405000	-0.94948100
O	-3.45749300	-1.19873700	-0.76203400
O	-3.15355300	0.73721100	-2.31743000
C	-4.63449200	1.05998500	-0.18411000
F	-4.73592200	0.74530300	1.10567000
F	-4.53249600	2.38137300	-0.28043600
F	-5.75497700	0.68250000	-0.79151600

8

E -3948.32177664

C	-0.54612000	4.35943500	-0.13079100
C	-0.42367800	3.00702600	0.19135700
C	-2.19085600	2.47178400	-1.19442100
C	-2.37182600	3.79651600	-1.55863400
C	-1.53246500	4.78283800	-1.02214500
H	0.13483500	5.08959400	0.30865600

H	-2.83256800	1.68936200	-1.60770800
H	-3.16500200	4.05821900	-2.26235800
C	0.58968700	2.47813000	1.12367000
C	1.46629000	3.27485100	1.86115800
C	1.46077000	0.55313700	2.06084900
C	2.38089200	2.67803000	2.73253400
H	1.43790400	4.36126800	1.76701100
C	2.35835800	1.28078500	2.82884000
H	1.40480100	-0.53301100	2.10997900
H	3.04514400	0.75734600	3.49800300
N	-1.24194700	2.07842000	-0.33844100
N	0.60668000	1.13967800	1.22341300
C	-1.69398500	6.22124400	-1.39206000
H	-2.68781200	6.58809800	-1.08897100
H	-0.93107900	6.85100500	-0.91475700
H	-1.62515700	6.35193400	-2.48354000
C	3.34455600	3.49280300	3.53364200
H	4.38222600	3.23422400	3.26819000
H	3.20198500	4.56915700	3.36713900
H	3.22963000	3.28586000	4.60933600
Ni	-0.71488600	0.16290300	0.04806800
C	-1.59884200	-0.79041000	1.51878600
O	-1.03710700	-1.09182200	2.56316500
C	-3.03755500	-1.23448100	1.35808000
C	-3.63205300	-2.08668700	2.29846800
C	-3.79225700	-0.80579900	0.26239100
C	-4.94837300	-2.50736400	2.15010700
H	-3.03104100	-2.41082400	3.15233000
C	-5.11493800	-1.20901100	0.09512500
H	-3.33277400	-0.14032500	-0.47352400
C	-5.66791500	-2.05786800	1.04586200
H	-5.42960000	-3.17447400	2.86942400
H	-5.71769800	-0.88049600	-0.75436200
F	-6.93441700	-2.45822200	0.89623000
C	-1.23181300	-2.26780100	-2.79895900
C	-0.80157300	-2.47133500	-1.35303300
C	0.13307900	-1.38529700	-0.84554300
C	0.43176900	-0.22403900	-1.62399600
C	-0.33528700	0.05521500	-2.90193100
C	-1.57330200	-0.81113700	-3.06187100
H	-1.68847400	-2.53570700	-0.70289500
H	-0.29725700	-3.44482900	-1.24655200
H	-0.40951800	-2.56795100	-3.47152500
H	-2.08657800	-2.92482100	-3.02284200

H	-0.59487300	1.12306200	-2.95610700
H	0.34104700	-0.13121900	-3.75757200
H	-2.35724200	-0.48572200	-2.35536800
H	-1.98655200	-0.67893300	-4.07396400
O	1.14052900	-1.90900100	0.02443500
S	2.24420900	-2.88826200	-0.62142800
O	2.01555100	-4.22674600	-0.10879100
O	2.40584700	-2.65793900	-2.04281500
C	3.80971300	-2.30974900	0.25352800
F	4.59576200	-1.68653000	-0.59114600
F	4.42330400	-3.40603600	0.67376000
F	3.54262900	-1.55037900	1.29131900
C	1.67189900	0.53077700	-1.41953200
O	1.61585400	1.73237200	-2.02919700
O	2.65710100	0.18841300	-0.79612600
C	2.74788400	2.56505400	-1.87329800
H	2.94106100	2.78352900	-0.81177200
H	3.64895700	2.10118200	-2.30325400
H	2.52188800	3.49708400	-2.40679900

TS-1B

E -3948.30987266

C	2.86274200	2.86699500	1.42420900
C	1.88788300	2.14330600	0.73554200
C	2.35606500	0.25292200	1.97604400
C	3.33040500	0.91207000	2.70999100
C	3.61378000	2.25459600	2.42950200
H	3.03018900	3.92001300	1.19412700
H	2.11966200	-0.79326500	2.18032900
H	3.86555600	0.37838900	3.49832600
C	1.01552300	2.74324600	-0.30024400
C	1.21920100	4.02104600	-0.82403300
C	-0.84450000	2.43656700	-1.62655700
C	0.34566900	4.52526700	-1.79016900
H	2.06195200	4.62783200	-0.49039100
C	-0.71292000	3.69931500	-2.18768600
H	-1.66163200	1.77148800	-1.91206100
H	-1.43373000	4.03678800	-2.93598900
N	1.66144100	0.84624000	1.00325700
N	-0.00340300	1.97148100	-0.70487900
C	4.67544900	2.99478100	3.17494800
H	5.67216500	2.63315500	2.87241100
H	4.63075200	4.07468200	2.97920100
H	4.58772800	2.82334500	4.25841500

C	0.52692100	5.88793200	-2.37596300
H	1.42051500	6.38551700	-1.97567300
H	0.61795800	5.83050600	-3.47209200
H	-0.34993700	6.51945800	-2.16098200
Ni	0.16787900	-0.01769900	-0.07378400
C	1.33808300	-0.43835300	-1.56274100
O	1.24161400	0.09295900	-2.65155000
C	2.53489100	-1.31200100	-1.29206700
C	2.57167800	-2.16977600	-0.18788500
C	3.63302800	-1.27261800	-2.16193100
C	3.67781700	-2.97861600	0.05417900
H	1.70910800	-2.20969300	0.48155700
C	4.75394000	-2.06109200	-1.92945700
H	3.59014800	-0.60631900	-3.02697800
C	4.75410400	-2.90387600	-0.82193900
H	3.71864200	-3.66014800	0.90652700
H	5.62415600	-2.03884800	-2.58958000
F	5.82596700	-3.66763800	-0.59458900
O	-2.33323200	-0.10478400	-0.79555200
S	-3.87403300	-0.46151000	-0.70863600
O	-4.56616000	0.31878800	-1.71529100
O	-4.12551600	-1.88135900	-0.55047300
C	-4.33951200	0.33535700	0.90633700
F	-3.96309400	-0.41563800	1.92284300
F	-5.65996300	0.44370700	0.92311400
F	-3.80619000	1.53469600	1.00486900
C	-0.55577600	-3.23985100	-1.89676600
C	-1.26021700	-1.89780100	-2.01853000
C	-1.24177800	-1.12011000	-0.72685400
C	-1.09863700	-1.83232200	0.53236700
C	-0.90557900	-3.32802000	0.59806300
C	-1.15141800	-4.02451500	-0.73608800
H	-0.84110400	-1.27939600	-2.82645100
H	-2.31169300	-2.09538800	-2.28673900
H	-0.66819900	-3.79321200	-2.84258300
H	0.52664000	-3.09967100	-1.74248600
H	0.11656700	-3.56678800	0.94719700
H	-1.57634300	-3.74340900	1.36916800
H	-0.73939300	-5.04557300	-0.70056100
H	-2.23748200	-4.12460200	-0.90413300
C	-1.15893900	-1.04232900	1.71240400
O	-1.24158300	-1.71605800	2.86793900
O	-1.11465500	0.20957200	1.70287200
C	-1.24550200	-0.94167800	4.05338600

H	-2.12878400	-0.28720800	4.10012000
H	-1.27321400	-1.65492300	4.88709300
H	-0.34119300	-0.31876900	4.12964100

9

E -2543.200879

Ni	0.17503000	-0.43327800	0.14420300
N	1.29305700	1.02184800	0.54382800
N	1.84366400	-1.12101700	-0.79238800
C	2.59753600	0.87039600	0.23159700
C	0.85127000	2.22277500	0.93090500
C	2.92426500	-0.41820800	-0.39701300
C	2.04031700	-2.29636100	-1.39625400
C	3.51080800	1.90449300	0.41294100
C	1.70641200	3.29892300	1.11019800
H	-0.22831600	2.32408800	1.05259400
C	4.22372600	-0.87608800	-0.60286700
C	3.30327500	-2.81367600	-1.64263800
H	1.15388700	-2.85252300	-1.70282200
C	3.07886500	3.14802900	0.88259800
H	4.55965400	1.75442600	0.15337800
H	1.29351400	4.25943200	1.42522400
C	4.43883900	-2.10009600	-1.24011400
H	5.07336400	-0.28594900	-0.25592800
H	3.39807400	-3.77771100	-2.14699700
C	4.03591000	4.26878300	1.11677600
C	5.81348900	-2.62804600	-1.48589300
H	4.27536700	4.33618800	2.19153700
H	3.59814200	5.23349100	0.82201100
H	4.97799900	4.11675600	0.57266500
H	5.91909300	-3.64416400	-1.07518500
H	6.58157800	-1.98354800	-1.03797100
H	6.00684800	-2.69942600	-2.56847400
C	-0.74399700	-0.52799900	1.79398800
C	-1.62459100	-1.56583400	1.86906500
C	-1.87945400	-2.30769500	0.64942700
O	-1.02483200	-1.87083000	-0.35673300
O	-2.67280400	-3.18400200	0.43007200
C	-1.31091700	-2.30321000	-1.68973000
H	-0.75181500	-1.64012200	-2.35669700
H	-2.38515300	-2.21119100	-1.88659600
H	-1.00676500	-3.35061400	-1.82071000
C	-2.36634300	-2.00390700	3.10532600
C	-1.71898300	-1.44478900	4.36278000

H	-2.40714600	-3.10527700	3.13533600
H	-3.41890800	-1.67012700	3.04118700
C	-1.41620200	0.03398100	4.18220300
H	-2.37306700	-1.60919300	5.23369100
H	-0.77776500	-1.98697900	4.56490200
C	-0.44524300	0.26569500	3.02650600
H	-1.00241900	0.47070800	5.10536500
H	-2.35881200	0.57157800	3.97536200
H	0.58877900	0.01421600	3.33333100
H	-0.40667900	1.34002100	2.79399400
O	-2.05867000	2.29241300	-0.12396100
S	-1.86022500	1.75884400	-1.48008400
O	-0.75601100	0.77373600	-1.61119600
O	-1.93128500	2.72718800	-2.57487300
C	-3.37547300	0.72519900	-1.72502300
F	-3.51568700	-0.16589700	-0.74305500
F	-4.46348400	1.48940800	-1.73921300
F	-3.32260500	0.06073000	-2.87781500

10

E -2987.124836

Ni	-0.03199800	-0.35130600	0.22985800
N	1.79282000	-0.82596100	-0.51699900
N	0.91107800	1.34981700	0.62101900
C	2.68545000	0.17594300	-0.45453400
C	2.13313900	-1.98064800	-1.08424500
C	2.19314300	1.40064900	0.21279000
C	0.37093700	2.39429200	1.25287600
C	3.96744700	0.03691300	-0.97947200
C	3.39328200	-2.19269000	-1.62621800
H	1.36886100	-2.76208200	-1.10340300
C	2.97094300	2.53330400	0.43679400
C	1.09134600	3.54881800	1.51307900
H	-0.67416800	2.29632400	1.55739900
C	4.34441500	-1.16646500	-1.58571000
H	4.68004500	0.86086700	-0.92228000
H	3.63052600	-3.15728500	-2.07964100
C	2.42608800	3.63884600	1.09718400
H	4.00900800	2.56021300	0.10355000
H	0.61070200	4.37670600	2.03792400
C	5.69913000	-1.33925500	-2.18855600
C	3.22774400	4.87374200	1.33951900
H	5.65072300	-1.14904200	-3.27410400
H	6.06623800	-2.36687100	-2.05499800

H	6.42545400	-0.63595200	-1.75886500
H	2.86530800	5.68998700	0.69299100
H	4.29296500	4.71416000	1.12606700
H	3.11567700	5.21524600	2.37951800
C	-1.10382800	-1.92509900	-0.09361600
C	-1.24733600	-2.68468800	1.02269100
C	-0.56961600	-2.30750600	2.27079400
O	0.26179800	-1.22776600	2.09411400
O	-0.66100200	-2.84816200	3.34082700
C	0.97795300	-0.74606800	3.23216400
H	1.71787300	-0.03164900	2.85566200
H	1.48261800	-1.57974000	3.73662000
H	0.29206000	-0.24836100	3.93243300
C	-2.05703400	-3.95533200	1.08462000
C	-2.95628300	-4.11351500	-0.13149900
H	-2.64360300	-3.95745200	2.01780000
H	-1.36897000	-4.81682000	1.16732400
C	-2.18489700	-3.80193100	-1.40470700
H	-3.37386400	-5.13193900	-0.16102400
H	-3.81282700	-3.42085100	-0.04876700
C	-1.69596200	-2.35729700	-1.39517700
H	-2.80290300	-3.97985100	-2.29881700
H	-1.31748900	-4.48169300	-1.47901700
H	-2.52961800	-1.67064800	-1.63711400
H	-0.95751500	-2.18903800	-2.19717800
C	-0.79785800	0.42697100	-1.32082200
O	-0.20891400	0.38779200	-2.36229500
C	-2.04679900	1.19604800	-1.09451500
C	-2.83117300	1.02325600	0.05286400
C	-2.43322700	2.13269200	-2.06624400
C	-3.98768000	1.77236700	0.23413900
H	-2.55480900	0.28680500	0.81224600
C	-3.58066400	2.89289900	-1.89264800
H	-1.81723000	2.25965100	-2.95900000
C	-4.33930700	2.69809000	-0.74169700
H	-4.61803300	1.64910200	1.11676600
H	-3.89918400	3.63110600	-2.63149600
F	-5.44127200	3.42360600	-0.57071800

TS-2

E -2987.121076

Ni	0.28813000	-0.51561200	0.57946600
N	2.20301900	-1.16261100	0.20739400
N	1.25808600	1.22630700	0.72358100

C	3.08608500	-0.15540900	0.07553500
C	2.61823300	-2.41340300	0.00370500
C	2.55545700	1.19338400	0.37612400
C	0.69426900	2.38909400	1.06090300
C	4.40958300	-0.38878600	-0.29265500
C	3.92158000	-2.72295300	-0.35560900
H	1.87880500	-3.20383400	0.13434400
C	3.32159600	2.35662300	0.33514700
C	1.39829600	3.58288700	1.05100800
H	-0.35938800	2.34981300	1.34450200
C	4.85430500	-1.69340200	-0.52302400
H	5.10788100	0.44210200	-0.39853600
H	4.20515600	-3.76673500	-0.50608100
C	2.74648600	3.58561300	0.67098400
H	4.37219600	2.31648800	0.04618500
H	0.89407200	4.50902000	1.33484800
C	6.26027100	-1.97989900	-0.93416700
C	3.53323100	4.85327500	0.62685300
H	6.28124900	-2.37650200	-1.96234200
H	6.70680500	-2.74943000	-0.28591200
H	6.88543000	-1.07778900	-0.89914100
H	3.47851600	5.37712600	1.59376300
H	3.11352100	5.53576300	-0.12968800
H	4.58770100	4.66868400	0.38246700
C	-0.96597400	-1.97804500	0.10757900
C	-2.10514600	-1.86335300	0.84625400
C	-2.28668500	-0.57352700	1.51990600
O	-3.49307400	-0.31840700	1.95782500
O	-1.34963800	0.21934600	1.65802600
C	-3.71716900	0.95270000	2.56744900
H	-3.54003400	1.75873700	1.84086000
H	-3.05684700	1.08715400	3.43548700
H	-4.76586300	0.95500300	2.88435700
C	-3.17905900	-2.90992600	0.93788400
C	-2.76203100	-4.24052400	0.32205400
H	-4.06997800	-2.50657000	0.42076100
H	-3.48867700	-3.04022100	1.98732400
C	-1.92162600	-4.04216900	-0.93090500
H	-2.17087100	-4.81426600	1.05645900
H	-3.65860500	-4.84107900	0.10403100
C	-0.65595500	-3.25960100	-0.59085900
H	-2.50040000	-3.49005600	-1.69247300
H	-1.65271600	-5.01301700	-1.37529500
H	-0.03922200	-3.06635000	-1.48107800

H	-0.03774500	-3.87211000	0.09064600
C	-0.51300700	-0.46822600	-1.17389700
O	0.15073400	-0.85774500	-2.09780500
C	-1.58696300	0.56792300	-1.33175000
C	-1.17731100	1.88414000	-1.58188700
C	-2.95269500	0.26760700	-1.29784000
C	-2.11473200	2.89889900	-1.74145300
H	-0.11371200	2.12287800	-1.64543100
C	-3.89998300	1.27532900	-1.46434100
H	-3.28932400	-0.76140700	-1.16465200
C	-3.46475300	2.57785700	-1.66840700
H	-1.81063200	3.93197000	-1.92216300
H	-4.96957200	1.05684700	-1.44295100
F	-4.36945800	3.54853600	-1.81378400

3-Ni

E -2987.15763

Ni	0.56923400	-0.07462400	0.44930900
N	1.93029600	1.39449300	0.06766100
N	2.15682200	-1.23524500	-0.09379400
C	3.15326500	0.93612800	-0.26284100
C	1.72352400	2.70785500	0.17724300
C	3.27270500	-0.53938300	-0.38215000
C	2.16626800	-2.56567300	-0.18625000
C	4.21339700	1.81089300	-0.48295000
C	2.73625100	3.63508300	-0.02782600
H	0.70038100	3.01043300	0.41486400
C	4.44134300	-1.18626200	-0.77480300
C	3.29408800	-3.27467600	-0.57433400
H	1.23297300	-3.08040300	0.05691700
C	4.02038900	3.19209100	-0.36155800
H	5.19897700	1.42859000	-0.75196200
H	2.51941500	4.70096600	0.06872300
C	4.47117200	-2.58160300	-0.87785000
H	5.33713700	-0.61180600	-1.01232500
H	3.25107200	-4.36357000	-0.64206700
C	5.15017700	4.14738400	-0.55987200
C	5.71820300	-3.29955200	-1.27469500
H	5.90018000	3.74429700	-1.25464800
H	5.65636800	4.33033600	0.40321900
H	4.79370000	5.11776800	-0.93265900
H	6.36903500	-2.66501600	-1.89205300
H	5.48946200	-4.22565100	-1.82065700
H	6.28801900	-3.58208200	-0.37321700

C	-1.23236300	0.38081200	1.21103800
C	-1.11109500	-1.02379800	1.00470400
C	-1.51344600	-1.60756400	-0.29920500
O	-1.15840700	-0.77755000	-1.30596300
O	-2.02798900	-2.68081000	-0.47818200
C	-1.55983600	-1.13282300	-2.62323200
H	-2.65158900	-1.25369600	-2.67147300
H	-1.07426200	-2.06830400	-2.93644800
H	-1.24381400	-0.30912100	-3.27352200
C	-0.97750200	-1.99587200	2.14774100
C	-0.43058600	-1.32619900	3.39743300
H	-0.34610400	-2.84444900	1.84092700
H	-1.97681200	-2.42020200	2.35611600
C	-1.22683300	-0.07084700	3.71466700
H	-0.46110300	-2.03192100	4.24163200
H	0.63261900	-1.06311800	3.24145100
C	-1.09484400	0.97205800	2.61303800
H	-0.90526600	0.36971900	4.67114100
H	-2.28986600	-0.34483400	3.83377900
H	-0.11838700	1.48176800	2.69426600
H	-1.85083800	1.76200900	2.74560800
C	-1.95377300	1.29685100	0.25870700
O	-1.47634700	2.37370300	-0.05338800
C	-3.31346800	0.90995100	-0.21396800
C	-4.09489900	-0.04605300	0.44911500
C	-3.83108500	1.55545100	-1.34637000
C	-5.37034300	-0.35879000	-0.01113800
H	-3.71747400	-0.54256800	1.34543900
C	-5.09412500	1.24041100	-1.82760500
H	-3.21944900	2.30648200	-1.85057000
C	-5.84372900	0.28453100	-1.14763300
H	-5.99868200	-1.09300800	0.49687100
H	-5.50891600	1.72068400	-2.71623000
F	-7.05892600	-0.02041700	-1.60189200

TS-R

E -2543.174258

Ni	0.30611700	-0.69173100	-0.73227500
N	-0.31869400	1.02257400	-0.37111100
N	-1.55673800	-1.21748700	-0.57851400
C	-1.65436900	1.09540400	-0.14129900
C	0.41227700	2.14126000	-0.30692100
C	-2.36478800	-0.18963500	-0.26293200
C	-2.06246200	-2.44275900	-0.72076200

C	-2.26971200	2.29912400	0.17272100
C	-0.14885200	3.37458000	-0.00918700
H	1.48068100	2.03745800	-0.49147000
C	-3.73167000	-0.37275900	-0.08444500
C	-3.41546800	-2.69983500	-0.55418400
H	-1.35590700	-3.23778300	-0.97441300
C	-1.51886000	3.47855100	0.24417300
H	-3.34327900	2.32451700	0.36470800
H	0.49696800	4.25387600	0.03054500
C	-4.28494700	-1.65018100	-0.23063600
H	-4.37413700	0.47191400	0.16824600
H	-3.79031000	-3.71804600	-0.67614400
C	-2.16669100	4.78680200	0.55019800
C	-5.75060000	-1.88204800	-0.07070700
H	-2.60447700	5.20936900	-0.37009600
H	-1.44113500	5.51401600	0.93911700
H	-2.98544600	4.66644800	1.27393800
H	-5.94666400	-2.82666500	0.45718000
H	-6.22669700	-1.96080700	-1.06254700
H	-6.23383200	-1.05776300	0.47082500
C	2.11487000	-0.30973700	-0.79693600
C	2.81887400	-0.39565500	0.34698800
C	2.09676000	-0.39628300	1.65693400
O	1.94419800	-1.62773500	2.15020500
O	1.71598900	0.59454500	2.23440900
C	1.28006000	-1.72610000	3.40361100
H	0.25366600	-1.33527800	3.33384000
H	1.82092400	-1.16814300	4.18223400
H	1.25540000	-2.79269000	3.65749800
C	4.32702900	-0.42670700	0.41688100
C	4.94670300	-0.65030500	-0.95601300
H	4.64554600	-1.21242600	1.12327200
H	4.68108400	0.52699600	0.85272300
C	4.26299300	0.20982100	-2.00999300
H	6.02720000	-0.43947600	-0.91845200
H	4.84137800	-1.71404600	-1.23417200
C	2.78343200	-0.15196600	-2.13317500
H	4.76172200	0.10397700	-2.98676200
H	4.35201900	1.27253400	-1.72149000
H	2.66376400	-1.08680800	-2.71231700
H	2.24931300	0.61778000	-2.71799000

9p

E -2543.217601

Ni	-0.39981700	-0.12886000	-0.22852200
N	0.90055700	1.25812500	-0.27034000
N	1.16524700	-1.28071600	-0.13379400
C	2.16983600	0.84273600	-0.03255700
C	0.70373400	2.54201700	-0.58199500
C	2.30857600	-0.61369700	0.11362200
C	1.16172100	-2.61437200	-0.09050100
C	3.23945900	1.72721000	-0.02100500
C	1.73116300	3.47473300	-0.60772600
H	-0.31284400	2.83845900	-0.82635300
C	3.48860900	-1.27810800	0.42666000
C	2.30546700	-3.34349600	0.20222600
H	0.20633100	-3.10147300	-0.29411300
C	3.03566200	3.08452100	-0.29630800
H	4.24636600	1.35701700	0.17731700
H	1.50248300	4.50875800	-0.87258100
C	3.50580100	-2.67692100	0.47443500
H	4.39836300	-0.71490000	0.63949800
H	2.25539900	-4.43401700	0.22378100
C	4.16555700	4.05711500	-0.25917300
C	4.76152800	-3.42493700	0.77564400
H	4.38590000	4.33064000	0.78649800
H	3.92247500	4.97909100	-0.80421700
H	5.08228800	3.61753800	-0.67828500
H	5.27897600	-3.67670800	-0.16572200
H	4.54936700	-4.36991500	1.29518700
H	5.45297900	-2.82370800	1.38200600
C	-2.02235100	0.83828800	0.10771200
C	-3.11628600	0.02031000	0.04139200
C	-2.74375100	-1.33550500	-0.27908400
O	-3.67496900	-2.24085100	-0.39314800
O	-1.53523700	-1.63190200	-0.42587400
C	-3.27759500	-3.58443600	-0.67606700
H	-2.64109200	-3.97356000	0.13104400
H	-2.73607700	-3.63307900	-1.63094900
H	-4.20434200	-4.16535800	-0.73714700
C	-4.54467700	0.39360300	0.32583700
C	-4.70401600	1.90205900	0.45933800
H	-4.87129000	-0.10803300	1.25526200
H	-5.19795700	-0.00271100	-0.46928100
C	-3.55487800	2.48861200	1.26528200
H	-4.71077400	2.36364500	-0.54392800
H	-5.67411500	2.13969600	0.92278100
C	-2.22337400	2.25205700	0.55574100

H	-3.52680000	2.01573800	2.26310300
H	-3.69911700	3.56809100	1.43035400
H	-1.38203300	2.56644600	1.19576800
H	-2.17483300	2.91122000	-0.33237900

9-ion

E -3504.454234

Ni	0.17503000	-0.43327800	0.14420300
N	1.29305700	1.02184800	0.54382800
N	1.84366400	-1.12101700	-0.79238800
C	2.59753600	0.87039600	0.23159700
C	0.85127000	2.22277500	0.93090500
C	2.92426500	-0.41820800	-0.39701300
C	2.04031700	-2.29636100	-1.39625400
C	3.51080800	1.90449300	0.41294100
C	1.70641200	3.29892300	1.11019800
H	-0.22831600	2.32408800	1.05259400
C	4.22372600	-0.87608800	-0.60286700
C	3.30327500	-2.81367600	-1.64263800
H	1.15388700	-2.85252300	-1.70282200
C	3.07886500	3.14802900	0.88259800
H	4.55965400	1.75442600	0.15337800
H	1.29351400	4.25943200	1.42522400
C	4.43883900	-2.10009600	-1.24011400
H	5.07336400	-0.28594900	-0.25592800
H	3.39807400	-3.77771100	-2.14699700
C	4.03591000	4.26878300	1.11677600
C	5.81348900	-2.62804600	-1.48589300
H	4.27536700	4.33618800	2.19153700
H	3.59814200	5.23349100	0.82201100
H	4.97799900	4.11675600	0.57266500
H	5.91909300	-3.64416400	-1.07518500
H	6.58157800	-1.98354800	-1.03797100
H	6.00684800	-2.69942600	-2.56847400
C	-0.74399700	-0.52799900	1.79398800
C	-1.62459100	-1.56583400	1.86906500
C	-1.87945400	-2.30769500	0.64942700
O	-1.02483200	-1.87083000	-0.35673300
O	-2.67280400	-3.18400200	0.43007200
C	-1.31091700	-2.30321000	-1.68973000
H	-0.75181500	-1.64012200	-2.35669700
H	-2.38515300	-2.21119100	-1.88659600
H	-1.00676500	-3.35061400	-1.82071000
C	-2.36634300	-2.00390700	3.10532600

C	-1.71898300	-1.44478900	4.36278000
H	-2.40714600	-3.10527700	3.13533600
H	-3.41890800	-1.67012700	3.04118700
C	-1.41620200	0.03398100	4.18220300
H	-2.37306700	-1.60919300	5.23369100
H	-0.77776500	-1.98697900	4.56490200
C	-0.44524300	0.26569500	3.02650600
H	-1.00241900	0.47070800	5.10536500
H	-2.35881200	0.57157800	3.97536200
H	0.58877900	0.01421600	3.33333100
H	-0.40667900	1.34002100	2.79399400
O	-2.05867000	2.29241300	-0.12396100
S	-1.86022500	1.75884400	-1.48008400
O	-0.75601100	0.77373600	-1.61119600
O	-1.93128500	2.72718800	-2.57487300
C	-3.37547300	0.72519900	-1.72502300
F	-3.51568700	-0.16589700	-0.74305500
F	-4.46348400	1.48940800	-1.73921300
F	-3.32260500	0.06073000	-2.87781500

9-S1

E -3504.468468

Ni	0.25341300	-0.14018800	-0.35346300
N	1.97502600	-0.95134700	-0.36590700
N	1.28718000	1.51677500	-0.20456800
C	3.01327100	-0.08643300	-0.28568000
C	2.24349000	-2.26127100	-0.42537200
C	2.61874700	1.32821000	-0.18981000
C	0.79996800	2.75288900	-0.08250500
C	4.33393900	-0.52431100	-0.27591300
C	3.53546200	-2.76358800	-0.41729900
H	1.38425200	-2.93139100	-0.47063500
C	3.50617600	2.39454100	-0.07056400
C	1.62340700	3.86171400	0.04532600
H	-0.28785500	2.83878500	-0.08701500
C	4.62501800	-1.88891600	-0.34605200
H	5.14600800	0.20080800	-0.20783800
H	3.68621100	-3.84403400	-0.46529900
C	3.01450500	3.69792700	0.04688300
H	4.58248200	2.21773500	-0.06194600
H	1.17793300	4.85383300	0.14486700
C	6.03070400	-2.39005100	-0.36139500
C	3.93134600	4.87097700	0.15974500
H	6.31338000	-2.68535600	-1.38574200

H	6.13853100	-3.28267200	0.27224000
H	6.73832900	-1.62080400	-0.02372300
H	3.90860000	5.45985000	-0.77209300
H	4.96858500	4.55935200	0.34183900
H	3.61127100	5.54237100	0.97071800
C	-0.67860400	-1.76087300	-0.46151700
C	-1.17377300	-2.36812000	0.64409400
C	-0.94232300	-1.81424700	1.98988800
O	-0.12054100	-0.74572000	1.99479200
O	-1.41474100	-2.25690100	3.01487000
C	0.00334600	-0.03757300	3.21062400
H	0.71248000	0.77863300	3.02041800
H	0.38802300	-0.68282200	4.01447000
H	-0.96726500	0.38018300	3.51684800
C	-2.03708300	-3.60646200	0.59867000
C	-2.61280200	-3.84390500	-0.78958300
H	-2.84141500	-3.50316700	1.34476100
H	-1.44870200	-4.48588100	0.92397100
C	-1.53057800	-3.70954700	-1.85053000
H	-3.09342800	-4.83441100	-0.83906000
H	-3.39552600	-3.09023600	-0.98253000
C	-0.94580300	-2.30014400	-1.83268000
H	-1.92597900	-3.94292300	-2.85284300
H	-0.72969600	-4.44455900	-1.64978000
H	-1.64551700	-1.59026700	-2.31112700
H	-0.02294300	-2.24983000	-2.43788400
O	-1.35571900	0.85558200	-0.62754700
S	-2.75523900	0.59392800	-0.11493700
O	-2.85193400	0.47287100	1.33666700
O	-3.53433000	-0.32596400	-0.93843900
C	-3.48684100	2.25195200	-0.48489400
F	-2.83506000	3.21282700	0.16384600
F	-3.43791900	2.51468500	-1.78352600
F	-4.75549200	2.27311500	-0.09639700

9p-ion

E -3504.467388

Ni	-0.43943800	-0.70397000	-0.87810600
N	-2.06093800	-1.36800500	-0.12186000
N	-1.47735000	0.92565900	-1.08694800
C	-3.01146200	-0.42258000	0.07870500
C	-2.36449200	-2.63873000	0.15992800
C	-2.64110100	0.91473900	-0.40945500
C	-1.02869300	2.06607600	-1.60507700

C	-4.24708300	-0.73098900	0.63491800
C	-3.58496300	-3.01809100	0.70007600
H	-1.60446400	-3.38471600	-0.06109300
C	-3.39664300	2.06742400	-0.23975600
C	-1.73860800	3.25524000	-1.48708300
H	-0.06833500	2.01617600	-2.11938900
C	-4.55671500	-2.05187400	0.97521800
H	-4.98654300	0.05694400	0.78561900
H	-3.77047600	-4.07450400	0.90402400
C	-2.94853500	3.27657200	-0.78862000
H	-4.33127700	2.03679000	0.32228300
H	-1.33684500	4.16699700	-1.93362600
C	-5.86311200	-2.40299700	1.60473800
C	-3.74383700	4.52957000	-0.62127800
H	-5.80561500	-2.24250800	2.69476800
H	-6.12057800	-3.45782300	1.43714900
H	-6.67459700	-1.76602500	1.22474000
H	-4.74823600	4.41036700	-1.05773000
H	-3.25339900	5.38722900	-1.10039200
H	-3.88490300	4.75701300	0.44722100
C	0.79258900	-2.09905000	-0.47429500
C	1.97402800	-1.94771000	-1.14159100
C	1.96028500	-0.77110200	-1.97478300
O	3.04932700	-0.43483400	-2.61396700
O	0.92505400	-0.07342200	-2.06094900
C	3.08466000	0.86604700	-3.19499200
H	2.91469400	1.61908100	-2.41268700
H	2.32627700	0.96610900	-3.98444500
H	4.08730900	0.98144500	-3.62282200
C	3.25801500	-2.68111000	-0.87587800
C	3.06098000	-3.81426200	0.12353000
H	3.98427300	-1.94727100	-0.48247500
H	3.69124300	-3.05854600	-1.81785400
C	2.11716600	-3.38415800	1.23706000
H	2.63152600	-4.69466900	-0.38692900
H	4.03472300	-4.13128300	0.52937000
C	0.73613900	-3.06568200	0.66935300
H	2.51496200	-2.47885400	1.73019400
H	2.03671400	-4.16409000	2.01127500
H	0.07899600	-2.66274100	1.45776600
H	0.25632600	-4.00655700	0.33833600
O	0.66874800	0.57163800	0.96668000
S	1.86637000	1.40573200	0.73013600
O	1.60067900	2.74149200	0.17910800

O	3.02847400	0.70791100	0.15650400
C	2.42409100	1.75254000	2.45905500
F	1.47992500	2.38822500	3.15081400
F	2.70924900	0.61982000	3.10172400
F	3.51794500	2.51259500	2.46584800

TS-add

E -3504.458704

Ni	0.47612600	-0.43333600	0.29474500
N	2.17759600	-1.17273300	-0.15457100
N	1.50016500	1.20634500	0.48648700
C	3.19141600	-0.27330200	-0.19936000
C	2.47832500	-2.46532000	-0.33818500
C	2.79163200	1.10182100	0.13180100
C	1.00181000	2.39522400	0.83269000
C	4.49990200	-0.65076500	-0.48622800
C	3.76092300	-2.90863800	-0.61926000
H	1.65885400	-3.17529900	-0.24576400
C	3.63597300	2.21003600	0.11532200
C	1.78765900	3.53776600	0.84954400
H	-0.06341600	2.42599500	1.07466400
C	4.81356600	-1.99158300	-0.71750000
H	5.28651100	0.10482700	-0.51398100
H	3.93390900	-3.97802400	-0.75751600
C	3.13845000	3.46297500	0.48242900
H	4.67943000	2.10414600	-0.18487500
H	1.34247900	4.49138600	1.14189100
C	6.20130200	-2.42771100	-1.05159900
C	4.00400100	4.68008600	0.47909500
H	6.26738900	-2.68911200	-2.12094500
H	6.47968500	-3.32778900	-0.48337700
H	6.93418500	-1.63481100	-0.85023600
H	5.03529800	4.44395900	0.18476700
H	4.02025300	5.14478000	1.47758900
H	3.60441500	5.43441500	-0.21732000
C	-0.60371300	-1.94603300	0.01964300
C	-1.54832100	-2.13470700	0.98012900
C	-1.52522500	-1.10382300	2.00401300
O	-2.47421900	-1.15349900	2.91680300
O	-0.68544700	-0.19177900	1.99867400
C	-2.60634100	-0.01568200	3.75880300
H	-2.76489600	0.88509900	3.14847800
H	-1.71254700	0.12075500	4.38475900
H	-3.48112600	-0.20224600	4.39308800

C	-2.70283100	-3.09409900	0.89244800
C	-2.58631700	-4.00654700	-0.32311500
H	-3.63022000	-2.49667800	0.83288100
H	-2.78619800	-3.68640300	1.81965800
C	-2.09921800	-3.22838000	-1.53778500
H	-1.87043800	-4.82128800	-0.11265000
H	-3.55673800	-4.48729800	-0.52503400
C	-0.69717800	-2.67968500	-1.28389700
H	-2.78282200	-2.38336900	-1.73439500
H	-2.10223300	-3.86105900	-2.44002900
H	-0.38803600	-1.99909300	-2.09584200
H	0.02795100	-3.51291400	-1.31562500
O	-1.06480900	0.70905800	-0.74916600
S	-2.36342600	1.27189500	-0.28335200
O	-2.24717300	2.55200000	0.42776500
O	-3.31498400	0.31021300	0.28167600
C	-3.13180200	1.72707200	-1.90122200
F	-2.37807900	2.60162600	-2.55956300
F	-3.28375600	0.64970700	-2.66760700
F	-4.32969300	2.27173900	-1.71010000

9p-S1

E -3504.466817

Ni	-0.30139900	-0.11258600	-0.35338500
N	-1.35334600	1.52400200	-0.08702000
N	-2.00908500	-0.94428200	-0.37007200
C	-2.68157400	1.31210000	-0.05068900
C	-0.88699900	2.76655000	0.04932000
C	-3.05686000	-0.10350700	-0.19659300
C	-2.25947500	-2.25225400	-0.49935000
C	-3.58530900	2.35737300	0.11768200
C	-1.72864900	3.85611400	0.22278700
H	0.19718700	2.87863900	0.02579700
C	-4.36804200	-0.56528500	-0.15772900
C	-3.54282100	-2.77753500	-0.46978900
H	-1.39212200	-2.90145200	-0.62613700
C	-3.11530000	3.66703000	0.25597300
H	-4.65776000	2.15985600	0.14434600
H	-1.29867300	4.85384100	0.33395100
C	-4.64052900	-1.92960200	-0.29423400
H	-5.18798300	0.14056700	-0.01825600
H	-3.67933400	-3.85458200	-0.58649600
C	-4.05415400	4.81806800	0.40955900
C	-6.03647800	-2.45310400	-0.22475300

H	-3.65480700	5.56535200	1.11051000
H	-5.04318700	4.48954600	0.75719100
H	-4.19006500	5.32384500	-0.56126300
H	-6.14223700	-3.38976900	-0.78954400
H	-6.76047500	-1.71779000	-0.60312400
H	-6.30293000	-2.66760900	0.82416600
C	0.64160700	-1.68964400	-0.70384700
C	1.22279100	-2.37953600	0.30584200
C	1.01059600	-1.85659400	1.66298100
O	1.64950200	-2.55495500	2.60757300
O	0.32829100	-0.88445900	1.94527700
C	1.55323300	-2.05373300	3.92835800
H	0.50976300	-2.04496100	4.27812000
H	1.95257000	-1.02980600	3.98786300
H	2.15027200	-2.72363600	4.55982000
O	1.32514000	0.87022500	-0.63074600
S	2.43970500	1.15431500	0.34891200
O	3.26769900	0.00835800	0.70818100
O	2.05881600	2.07680400	1.41702700
C	3.51009700	2.16094900	-0.77149000
F	2.86714100	3.24461100	-1.19201200
F	4.60056800	2.54701400	-0.11996600
F	3.88113100	1.45353000	-1.83095400
C	2.12692000	-3.57084100	0.09528400
C	2.61960400	-3.65038400	-1.34402000
H	1.59738100	-4.50380200	0.36683500
H	2.97995400	-3.50516200	0.78847600
C	1.47493400	-3.45094100	-2.32619200
H	3.37423700	-2.86012700	-1.50934900
H	3.12591300	-4.61321400	-1.51996700
C	0.84400300	-2.07428700	-2.13496800
H	0.71094500	-4.23122800	-2.15623900
H	1.82001900	-3.57076400	-3.36623200
H	-0.11333100	-2.00156500	-2.68157000
H	1.48484200	-1.29066100	-2.58399500

10p

E -2987.129895

Ni	0.02382000	0.27088100	0.22061200
N	-1.80842800	0.21977400	-0.66527800
N	-0.49132100	-1.58902800	0.68456800
C	-2.43289300	-0.96341900	-0.54921500
C	-2.38404500	1.20574600	-1.34776700
C	-1.69951200	-1.97725400	0.23896300

C	0.24923700	-2.42440200	1.41559200
C	-3.67629100	-1.18645100	-1.13519800
C	-3.62299500	1.05783700	-1.95658800
H	-1.83230900	2.14755000	-1.40648700
C	-2.19397000	-3.24757800	0.52554900
C	-0.18387200	-3.69996300	1.73986800
H	1.22319200	-2.05442300	1.74600800
C	-4.29745800	-0.16476000	-1.86140400
H	-4.16971900	-2.15352800	-1.03250900
H	-4.05925200	1.89588800	-2.50403100
C	-1.43478700	-4.14004800	1.28754600
H	-3.17653500	-3.54949800	0.16087600
H	0.45494700	-4.34954700	2.34162000
C	-5.61462700	-0.38125000	-2.53024000
C	-1.93052700	-5.51101600	1.60702800
H	-5.45524000	-0.67216000	-3.58236800
H	-6.21615600	0.53881500	-2.53437200
H	-6.18553600	-1.18540300	-2.04603200
H	-1.94327700	-5.67286500	2.69624200
H	-1.25567100	-6.26985000	1.17969200
H	-2.94115500	-5.67937900	1.21232100
C	0.64670700	2.06454200	-0.18847600
C	0.22504400	2.93763700	0.76700200
C	-0.48930000	2.32821200	1.88705300
O	-0.89292100	3.14460700	2.83042400
O	-0.71028500	1.10953500	1.94821500
C	-1.60230200	2.58381000	3.93388200
H	-0.97046900	1.86455100	4.47402200
H	-2.51666400	2.08037300	3.58953400
H	-1.85827300	3.42531700	4.58747300
C	0.42199000	4.43066300	0.71319400
C	0.76808100	4.89013700	-0.69624700
H	1.22249000	4.72164100	1.41785400
H	-0.48962600	4.93158700	1.07578300
C	1.84387700	4.00167100	-1.30118600
H	-0.13740400	4.84616000	-1.32764000
H	1.09165100	5.94253300	-0.68048500
C	1.37296100	2.55276900	-1.39742700
H	2.74733500	4.04627600	-0.66757100
H	2.14100000	4.36264800	-2.29845000
H	2.22780100	1.89093300	-1.61452700
H	0.69450300	2.41958400	-2.26213400
C	1.02592900	-0.38626100	-1.25284800
O	0.50543500	-0.56758700	-2.31624900

C	2.42379300	-0.77896200	-0.93411400
C	3.10864400	-1.60336200	-1.83998500
C	3.06144300	-0.35952400	0.23982000
C	4.40885000	-2.00989400	-1.57592700
H	2.60414800	-1.92432000	-2.75387600
C	4.36607300	-0.75290500	0.51292200
H	2.54707200	0.29743200	0.94767500
C	5.01552900	-1.57550700	-0.40069300
H	4.96091700	-2.65474300	-2.26283600
H	4.88528100	-0.43208600	1.41803300
F	6.26299300	-1.96095900	-0.14352700

TS-2b

E -2987.111755

Ni	0.23822800	-0.48879700	0.45094300
N	2.16006600	-1.08270500	0.12623200
N	1.13977400	1.30653800	0.56270700
C	3.02767700	-0.05844700	0.02892200
C	2.62084200	-2.33073500	0.03091000
C	2.44413700	1.28616900	0.22910700
C	0.54529000	2.48487800	0.77447400
C	4.38274600	-0.27229800	-0.21084000
C	3.95798700	-2.62047400	-0.19759300
H	1.89104000	-3.13328700	0.14578800
C	3.17870700	2.46276100	0.09733200
C	1.21575400	3.69285400	0.66939600
H	-0.51597200	2.46693900	1.02639200
C	4.87601300	-1.57441900	-0.33922500
H	5.06818400	0.57238500	-0.28695100
H	4.27944000	-3.66183200	-0.26403700
C	2.57040700	3.70127400	0.31673800
H	4.23173900	2.42310300	-0.18247500
H	0.67698700	4.62428400	0.85516500
C	6.31660800	-1.83626300	-0.62742600
C	3.32575400	4.98066300	0.17888100
H	6.45038800	-2.03642200	-1.70395300
H	6.67507800	-2.72375500	-0.08629300
H	6.94485400	-0.97381900	-0.36643600
H	3.29377100	5.54915200	1.12173100
H	2.86360300	5.61694100	-0.59257100
H	4.37538200	4.80599100	-0.09212300
C	-0.92859200	-1.96683900	-0.17440100
C	-1.94808300	-2.18496500	0.70062600
C	-2.28943600	-1.18948600	1.73459000

O	-1.31220900	-0.24808000	1.88043400
O	-3.27696900	-1.18634700	2.41985300
C	-1.53301500	0.76359600	2.86779800
H	-1.91856700	0.30490000	3.78678900
H	-2.25420900	1.50488600	2.49487500
H	-0.56102100	1.22910100	3.06008200
C	-2.84472400	-3.39319000	0.67651200
C	-2.38112100	-4.48320700	-0.28087300
H	-3.85104500	-3.03144900	0.39224000
H	-2.96374000	-3.78554900	1.69911800
C	-1.75382900	-3.89822800	-1.53662200
H	-1.63266900	-5.11927400	0.22213800
H	-3.23085400	-5.13753800	-0.52997100
C	-0.55579200	-3.03693000	-1.15264600
H	-2.49150000	-3.28301400	-2.08172900
H	-1.43529800	-4.69993200	-2.22087800
H	-0.06262400	-2.59475800	-2.02964900
H	0.19555900	-3.68835500	-0.67372800
C	-0.74065200	-0.24268100	-1.18868000
O	-0.08551700	-0.37856000	-2.19023500
C	-1.89046800	0.71051400	-1.11766000
C	-1.61885300	2.02215100	-1.54073500
C	-3.18882000	0.38248100	-0.71213900
C	-2.60419400	3.00039300	-1.50544400
H	-0.61665100	2.28000500	-1.88911700
C	-4.18846900	1.35089100	-0.68609800
H	-3.44761100	-0.64489500	-0.46010800
C	-3.87600600	2.64864000	-1.06795400
H	-2.40027000	4.02756000	-1.81469800
H	-5.20788400	1.10389500	-0.38323000
F	-4.82744800	3.58149900	-1.03082700

3-Nip

E -2987.157288

Ni	0.54726000	-0.05377800	0.49058200
N	1.92658900	1.40761200	0.05681800
N	2.11778800	-1.22966600	-0.12543000
C	3.12270400	0.93588300	-0.34347200
C	1.74111200	2.72205800	0.18735100
C	3.22232500	-0.54140700	-0.46846700
C	2.11043100	-2.55950200	-0.22136900
C	4.17738400	1.80084500	-0.62321300
C	2.74940100	3.63933700	-0.07539200
H	0.74081600	3.03482100	0.50271200

C	4.36555300	-1.19746600	-0.91803200
C	3.21259900	-3.27715400	-0.66377000
H	1.18417700	-3.06659100	0.06368600
C	4.00600200	3.18345800	-0.48822300
H	5.14192500	1.41008300	-0.94971000
H	2.55137600	4.70703400	0.04019800
C	4.37963900	-2.59279800	-1.02184000
H	5.25340300	-0.63024500	-1.19899400
H	3.15806600	-4.36561400	-0.73075100
C	5.13053300	4.12925100	-0.75158000
C	5.60124300	-3.32073300	-1.47587700
H	5.86118200	3.70181100	-1.45208800
H	5.66150000	4.34827900	0.19038000
H	4.76312200	5.08594300	-1.14892800
H	6.24147400	-2.68280400	-2.10065900
H	5.33955000	-4.23067000	-2.03425500
H	6.19537500	-3.63326600	-0.60041700
C	-1.21412000	0.39418600	1.41740200
C	-1.16973600	-0.98697300	1.07111900
C	-1.51155300	-1.35291700	-0.32805200
O	-2.28659200	-2.40467400	-0.46245100
O	-1.03942200	-0.72744600	-1.27130800
C	-2.61953300	-2.79687100	-1.79342200
H	-1.71089500	-3.02616000	-2.36788000
H	-3.17953500	-1.99679400	-2.29832500
H	-3.24438600	-3.69195200	-1.69890600
C	-1.08624600	-2.08145000	2.10252200
C	-0.45154600	-1.59006600	3.39334600
H	-0.53337400	-2.94013100	1.69087200
H	-2.11148700	-2.44409900	2.30222500
C	-1.11721700	-0.30412800	3.85689800
H	-0.52834100	-2.37085600	4.16546700
H	0.62657700	-1.41020100	3.22644000
C	-0.91776100	0.82272400	2.85243500
H	-0.72498500	0.00965700	4.83678000
H	-2.19773300	-0.48826800	3.99181100
H	0.12135200	1.19270400	2.91734300
H	-1.55312500	1.68464800	3.10532300
C	-1.88853900	1.44380800	0.58918400
O	-1.45859000	2.58714900	0.56302200
C	-3.14854100	1.10920400	-0.13540200
C	-4.03580800	0.12875000	0.32806100
C	-3.47797300	1.84775700	-1.28039700
C	-5.22573800	-0.12539900	-0.34795700

H	-3.81056200	-0.43217600	1.23737900
C	-4.65152100	1.59206600	-1.97613900
H	-2.78993500	2.62144100	-1.62742800
C	-5.50689200	0.60438000	-1.49572400
H	-5.93447000	-0.87764800	0.00380800
H	-4.91635900	2.14266900	-2.88125800
F	-6.63766500	0.35636400	-2.15720800

10-S1

E -3948.389348

Ni	-0.25634500	-0.04651400	-0.14418200
N	1.08656500	-0.32097700	1.34870100
N	0.58203700	1.78431100	-0.15376100
C	1.84606700	0.75581200	1.59726100
C	1.23113200	-1.41473300	2.09244400
C	1.60928400	1.91282900	0.70627900
C	0.33660800	2.76530200	-1.02498100
C	2.79224500	0.75393900	2.62159100
C	2.14743300	-1.48873300	3.13082600
H	0.58468100	-2.26009800	1.84724500
C	2.40720700	3.05593300	0.71479900
C	1.08874300	3.92874000	-1.07218700
H	-0.49393100	2.61450200	-1.71801700
C	2.95965600	-0.38413100	3.41403900
H	3.39960200	1.63869700	2.81509700
H	2.22927200	-2.40769000	3.71529600
C	2.15638400	4.09636400	-0.18241300
H	3.24115800	3.13752200	1.41262100
H	0.84346400	4.69889900	-1.80635900
C	3.96434100	-0.42679300	4.51849900
C	2.99022800	5.33465400	-0.19963500
H	4.72453500	-1.19809500	4.31538500
H	3.48247900	-0.69669700	5.47120000
H	4.47306600	0.53862800	4.64186700
H	2.37654700	6.21215600	0.06041400
H	3.39446400	5.51523500	-1.20780800
H	3.82554800	5.27137000	0.51052200
C	-1.18747200	-1.72390900	-0.18434800
C	-2.10562600	-1.89698200	0.80090700
C	-2.30814700	-0.73239300	1.65821400
O	-3.27004100	-0.85117400	2.55684300
O	-1.62932400	0.29114400	1.56450800
C	-3.49563000	0.26149000	3.41446300
H	-3.79269100	1.14671700	2.83352000

H	-2.59177100	0.49704900	3.99499800
H	-4.30825100	-0.03252400	4.08908800
C	-2.88129100	-3.16935200	1.01892000
C	-2.28285400	-4.33284800	0.23589800
H	-3.93014300	-3.00069300	0.71115600
H	-2.92429500	-3.40454800	2.09450500
C	-1.89015700	-3.91319100	-1.17476100
H	-1.38192800	-4.69889400	0.76002200
H	-2.99528800	-5.17251200	0.21239900
C	-0.83407900	-2.81317500	-1.13125000
H	-2.78226100	-3.54120600	-1.71008900
H	-1.51354000	-4.77568900	-1.74759100
H	-0.63959400	-2.38029100	-2.12531900
H	0.13973400	-3.22179900	-0.80472300
C	-1.61802600	0.12571600	-1.50989400
O	-1.40079400	-0.21408300	-2.64506300
C	-2.93140100	0.76360100	-1.16256000
C	-3.05469600	1.84429200	-0.28341300
C	-4.07744300	0.25801500	-1.79317700
C	-4.29971200	2.41525400	-0.03445600
H	-2.17309600	2.24921200	0.21243200
C	-5.33145500	0.79729800	-1.52977700
H	-3.97839200	-0.57819300	-2.48915900
C	-5.41897000	1.87285200	-0.65296100
H	-4.41273600	3.26806400	0.63809900
H	-6.23676100	0.40159800	-1.99510100
F	-6.61814000	2.40312200	-0.39969600
O	1.30291700	-0.48840900	-1.55478000
S	2.34480900	-1.54538100	-1.67738900
O	2.48540800	-2.42495700	-0.51183900
O	2.38482700	-2.18759700	-2.99088200
C	3.89096000	-0.53023900	-1.64145800
F	3.90273600	0.35299600	-2.63488500
F	4.96124400	-1.30966500	-1.76539200
F	4.00045800	0.13925600	-0.49474700

TS-2c

E -3948.38059

Ni	-0.16587200	-0.05109000	-0.21312800
N	1.15469300	-0.16733300	1.36868400
N	0.76889900	1.74000100	-0.42973000
C	1.85265500	0.96010200	1.56058800
C	1.23661100	-1.15048800	2.26055800
C	1.71605000	1.98077900	0.49258700

C	0.64446800	2.57027000	-1.46603100
C	2.66844900	1.12718100	2.68022800
C	2.02670300	-1.05989400	3.39774300
H	0.64629300	-2.04671400	2.05349100
C	2.54549000	3.09934200	0.40477400
C	1.43576000	3.69861300	-1.62163100
H	-0.11950700	2.31782200	-2.20557700
C	2.76831700	0.10562700	3.62810900
H	3.22355300	2.05417600	2.82864900
H	2.06305500	-1.89395600	4.10218000
C	2.41433400	3.98960000	-0.66326700
H	3.31424700	3.27430900	1.15832100
H	1.29120200	4.34534500	-2.48972000
C	3.63725900	0.24298100	4.83573300
C	3.28709400	5.19496800	-0.79117400
H	4.48542000	-0.45859200	4.77690600
H	3.07877600	-0.00690400	5.75087100
H	4.03909200	1.26065800	4.93228400
H	2.67774400	6.11279200	-0.78930100
H	3.83347100	5.17675000	-1.74733500
H	4.01575300	5.25801700	0.02829600
C	-1.40889500	-1.61548100	-0.29779800
C	-2.14160300	-1.64716800	0.85784500
C	-2.31469000	-0.35632800	1.54013600
O	-3.30945200	-0.30197400	2.39868400
O	-1.56602100	0.59392000	1.32629900
C	-3.51169100	0.93560400	3.07685500
H	-3.76062500	1.73002300	2.35867500
H	-2.61092900	1.22106000	3.63895500
H	-4.35001000	0.77273000	3.76352000
C	-2.78287400	-2.87635800	1.42501100
C	-2.28033300	-4.16297800	0.78114100
H	-3.87377200	-2.76651200	1.27252700
H	-2.64793300	-2.89922600	2.51825200
C	-2.06182000	-3.99550600	-0.71559400
H	-1.32236300	-4.45166200	1.24740900
H	-2.98939500	-4.97898400	0.99013500
C	-1.03653000	-2.89883100	-0.97556000
H	-3.01599500	-3.73737300	-1.20924300
H	-1.71935700	-4.94232900	-1.16211600
H	-0.88643900	-2.70612700	-2.04619000
H	-0.05668000	-3.21041300	-0.57629200
C	-1.73866000	-0.34235700	-1.40557300
O	-1.52824500	-0.61787500	-2.58198000

C	-2.98932300	0.47101900	-1.11543800
C	-2.94832100	1.85700600	-0.95003200
C	-4.23026400	-0.17517200	-1.11420600
C	-4.12006700	2.59166000	-0.78170600
H	-1.98830400	2.37351000	-0.92821000
C	-5.41197100	0.54223900	-0.93791900
H	-4.27644200	-1.25938700	-1.24647900
C	-5.33498600	1.91885900	-0.77399100
H	-4.09955400	3.67511700	-0.64574100
H	-6.38552000	0.04737100	-0.92566200
F	-6.46379900	2.61746500	-0.60189400
O	1.23270000	-0.78590200	-1.59345500
S	2.25841700	-1.86538000	-1.53323300
O	2.28105000	-2.63815800	-0.28635500
O	2.38109200	-2.62191400	-2.77822600
C	3.82756100	-0.88723400	-1.46705300
F	3.91479700	-0.06225200	-2.50567600
F	4.87982300	-1.70006600	-1.49145500
F	3.89778900	-0.15811500	-0.35516600

3-Ni-S1

E -3948.421075

C	2.45523700	0.27325700	-1.51976500
O	2.14705000	-0.14255300	-2.62615500
C	3.83358600	0.01271100	-1.00022100
C	4.52339300	-1.10617400	-1.48558700
C	4.47489100	0.87290000	-0.09922800
C	5.81409500	-1.38855900	-1.05795500
H	4.02309600	-1.76308600	-2.20015700
C	5.77539200	0.61369400	0.32514000
H	3.96591700	1.76688300	0.26624500
C	6.41854800	-0.52014400	-0.15412400
H	6.35937600	-2.26653900	-1.41088400
H	6.29399400	1.27819700	1.01917300
F	7.65952100	-0.78274600	0.26029000
O	-1.58053900	1.24027100	-0.64646500
S	-2.73231200	1.53831500	0.26338100
O	-3.72004800	0.46431400	0.36850900
O	-2.35367300	2.21735900	1.50623300
C	-3.61214700	2.83683800	-0.71652700
F	-2.84258100	3.90252100	-0.91382300
F	-4.70271500	3.22800700	-0.06586200
F	-3.97882000	2.36275100	-1.90142200
Ni	-0.00170400	-0.03031000	-0.10981900

C	0.59988100	3.53551800	-0.66835500
C	1.17534200	2.41160400	-1.51911800
C	1.52560100	1.15347200	-0.74320100
C	1.45229000	1.15431700	0.68182800
C	1.16110400	2.40245700	1.48091600
C	1.34825600	3.66196800	0.64801900
H	0.48917700	2.14861900	-2.33968700
H	2.10125800	2.76815100	-2.00733400
H	0.64355700	4.47597700	-1.24042900
H	-0.45756700	3.33192900	-0.45535500
H	0.12525800	2.36757700	1.85963200
H	1.82077600	2.41450000	2.36191500
H	0.99299100	4.53655300	1.21548400
H	2.42387900	3.82480100	0.45233400
C	1.93228300	-0.06177400	1.35694200
O	2.36647900	0.09950600	2.59429300
O	1.86704700	-1.16434700	0.81325200
C	2.74759500	-1.07812800	3.29955600
H	3.59394600	-1.57140800	2.80032600
H	3.04359600	-0.74936500	4.30238900
H	1.90555400	-1.78215700	3.36619300
C	-2.63363800	-2.83119900	1.59192200
C	-1.83937500	-2.02946100	0.77406800
C	-1.20985700	-0.73217100	2.58099600
C	-1.98273100	-1.48266900	3.45429000
C	-2.72075200	-2.56538500	2.96090500
H	-3.18899600	-3.67088900	1.17279700
H	-0.62872500	0.12116100	2.93870600
H	-2.00898900	-1.22329500	4.51477000
C	-1.71096000	-2.23023900	-0.68956000
C	-2.47249700	-3.15410000	-1.40365400
C	-0.63623000	-1.55312000	-2.62432900
C	-2.30838500	-3.27904500	-2.78642600
H	-3.20929600	-3.77581900	-0.89356500
C	-1.36099900	-2.44991100	-3.39725800
H	0.12760800	-0.90159100	-3.05765600
H	-1.18649000	-2.49856700	-4.47434100
N	-1.13589000	-1.00376600	1.27839300
N	-0.80870200	-1.44724900	-1.30686100
C	-3.58038400	-3.39019700	3.86157100
H	-4.47151700	-2.81505700	4.16305600
H	-3.92011800	-4.31023800	3.36708800
H	-3.04315800	-3.65648500	4.78421400
C	-3.09796100	-4.26983900	-3.57759200

H	-3.96623200	-4.63851000	-3.01459600
H	-3.44407900	-3.83251000	-4.52578800
H	-2.46663500	-5.13700500	-3.83393500

2.8 NMR spectra

2.8.1. NMR spectra of substrates

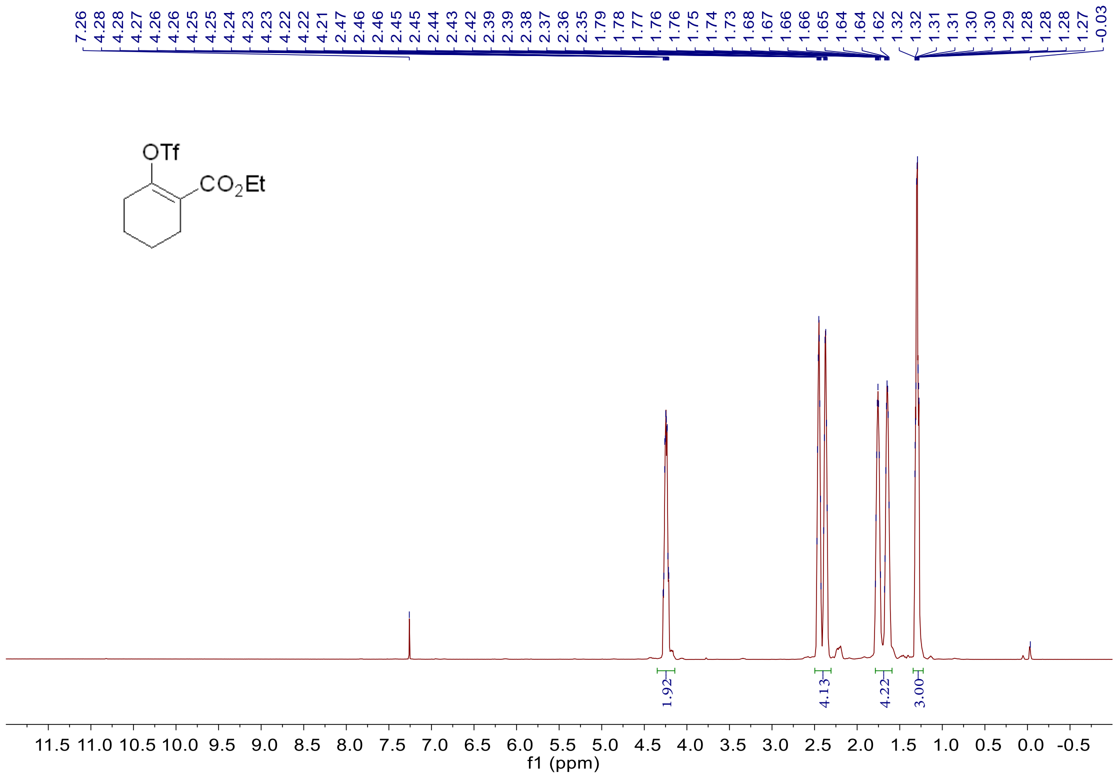

Supplementary Figure 17. ^{1}H NMR spectrum for compound **2a**

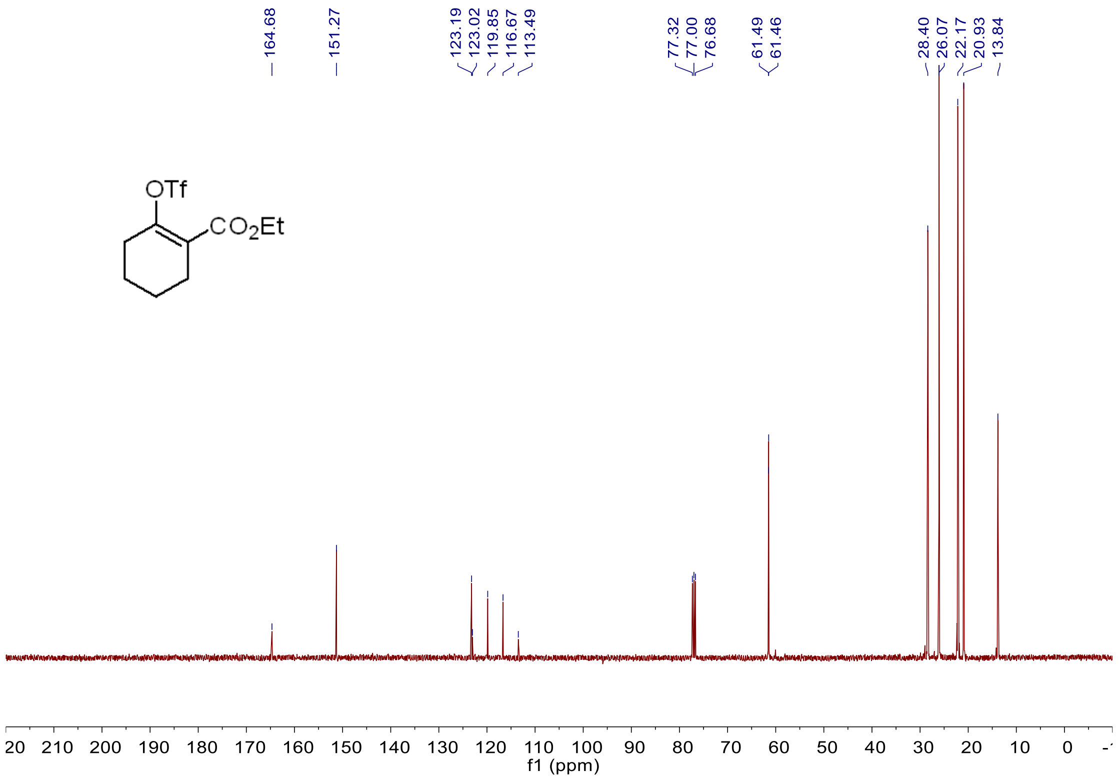

Supplementary Figure 18. ^{13}C NMR spectrum for compound **2a**

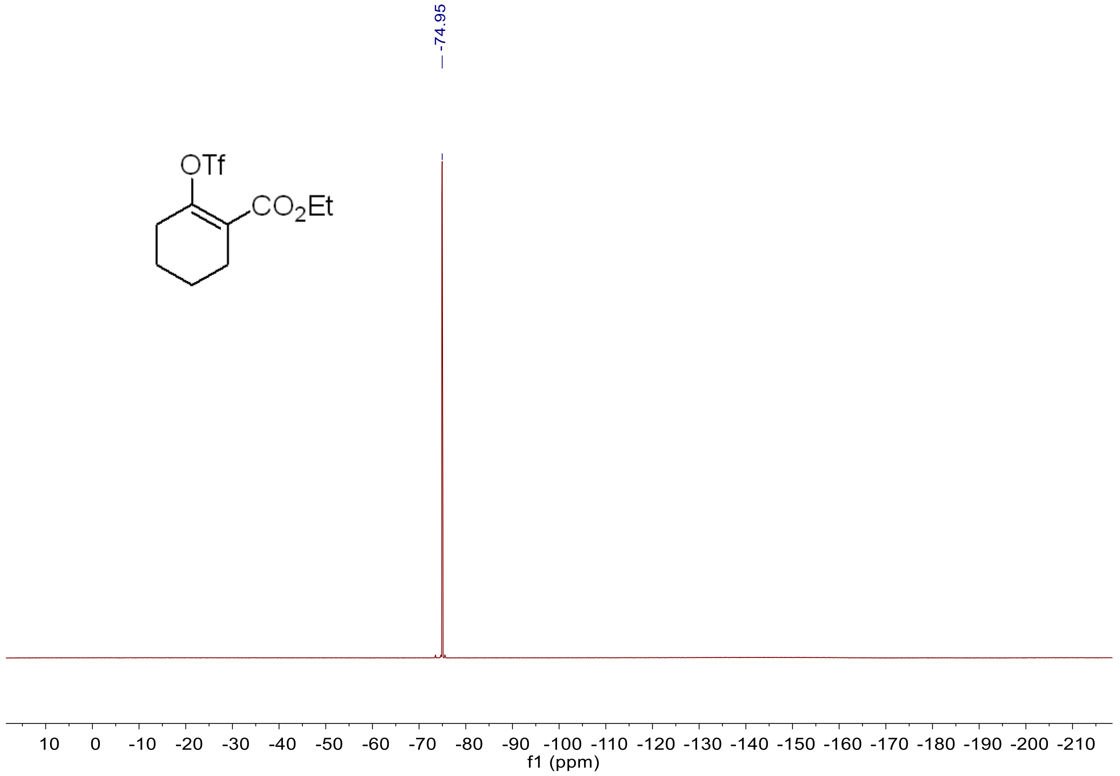

Supplementary Figure 19. ^{19}F NMR spectrum for compound **2a**

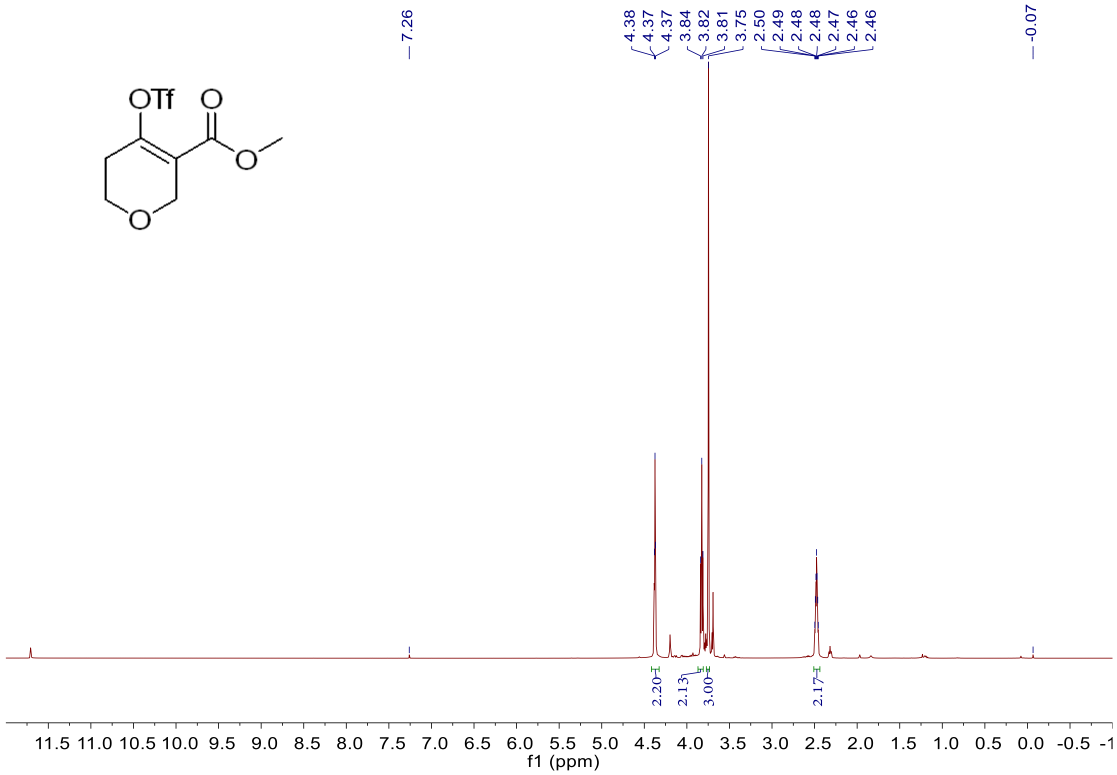

Supplementary Figure 20. ^{1}H NMR spectrum for compound **2b**

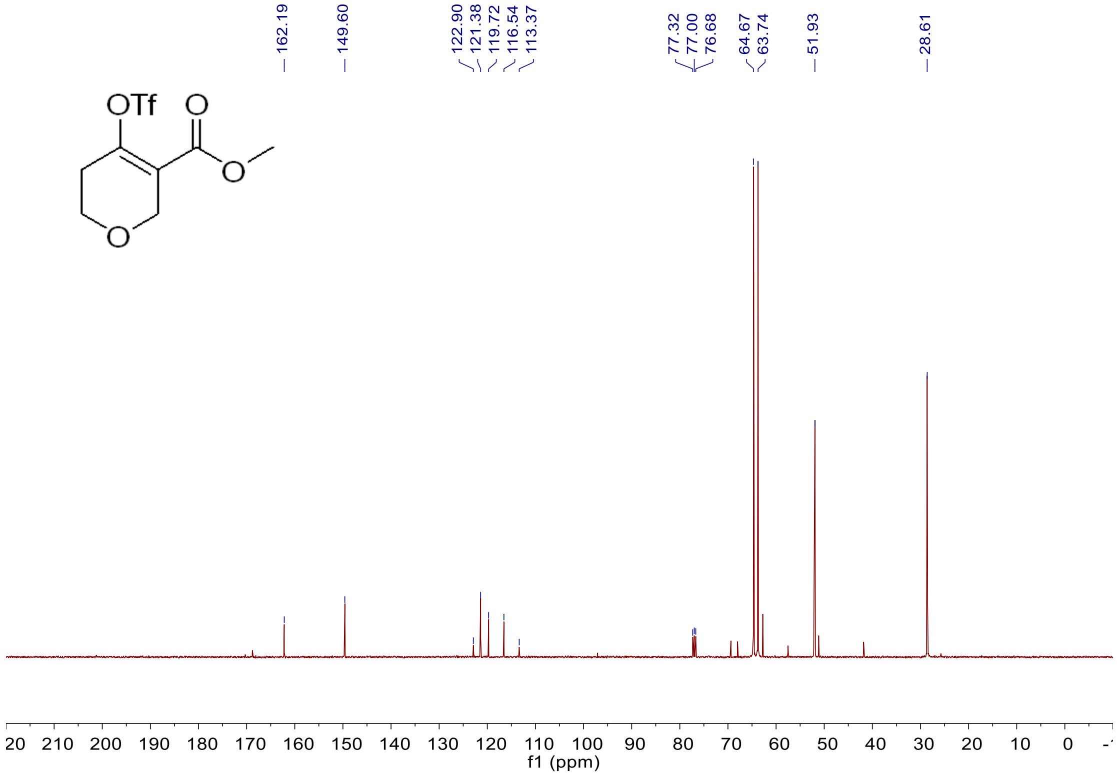

Supplementary Figure 21. ^{13}C NMR spectrum for compound **2b**

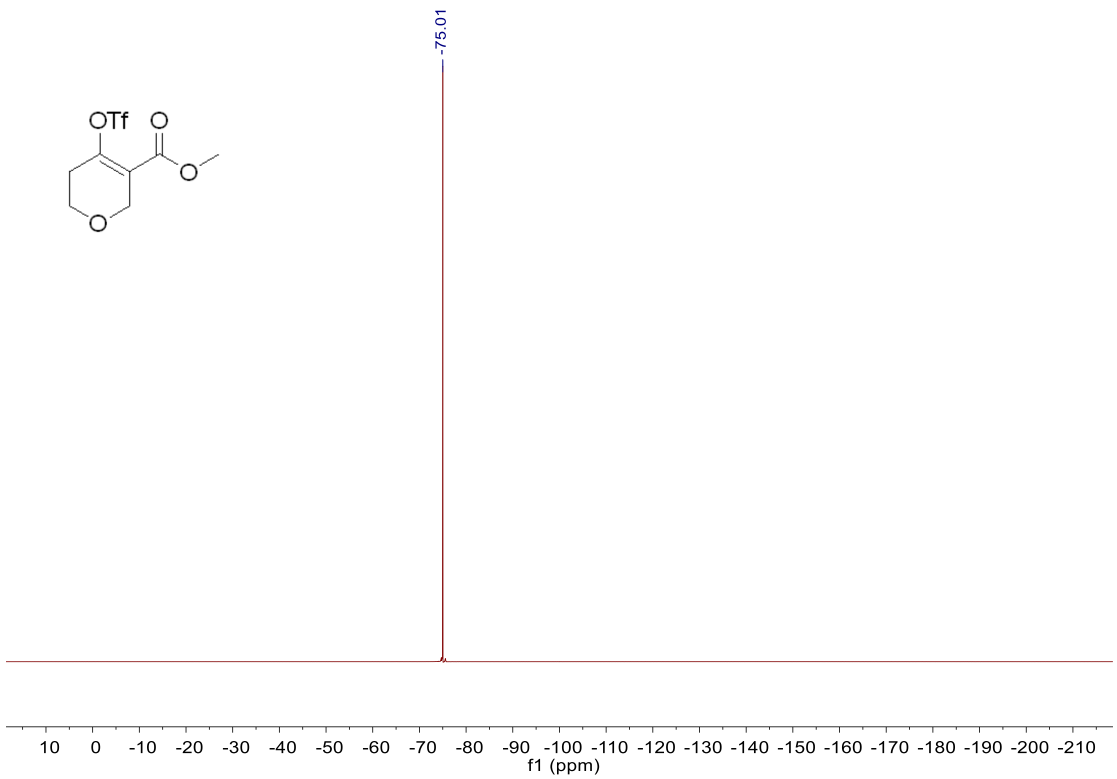

Supplementary Figure 22. ^{19}F NMR spectrum for compound **2b**

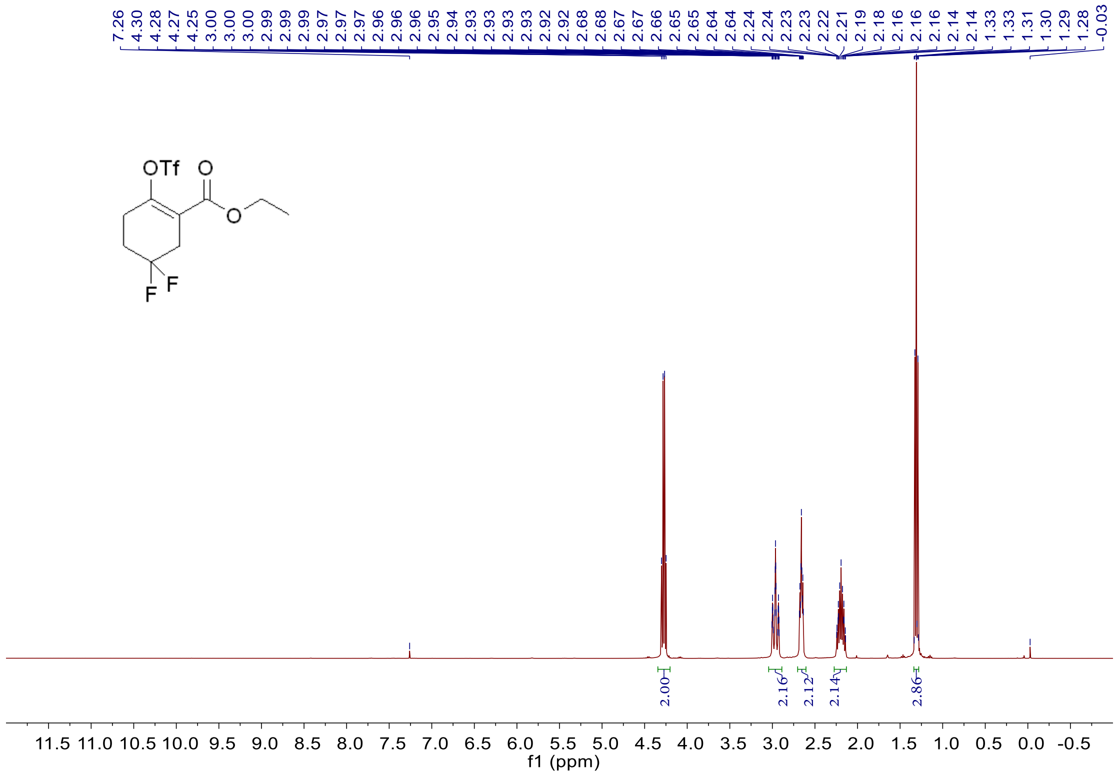

Supplementary Figure 23. ^{1}H NMR spectrum for compound **2c**

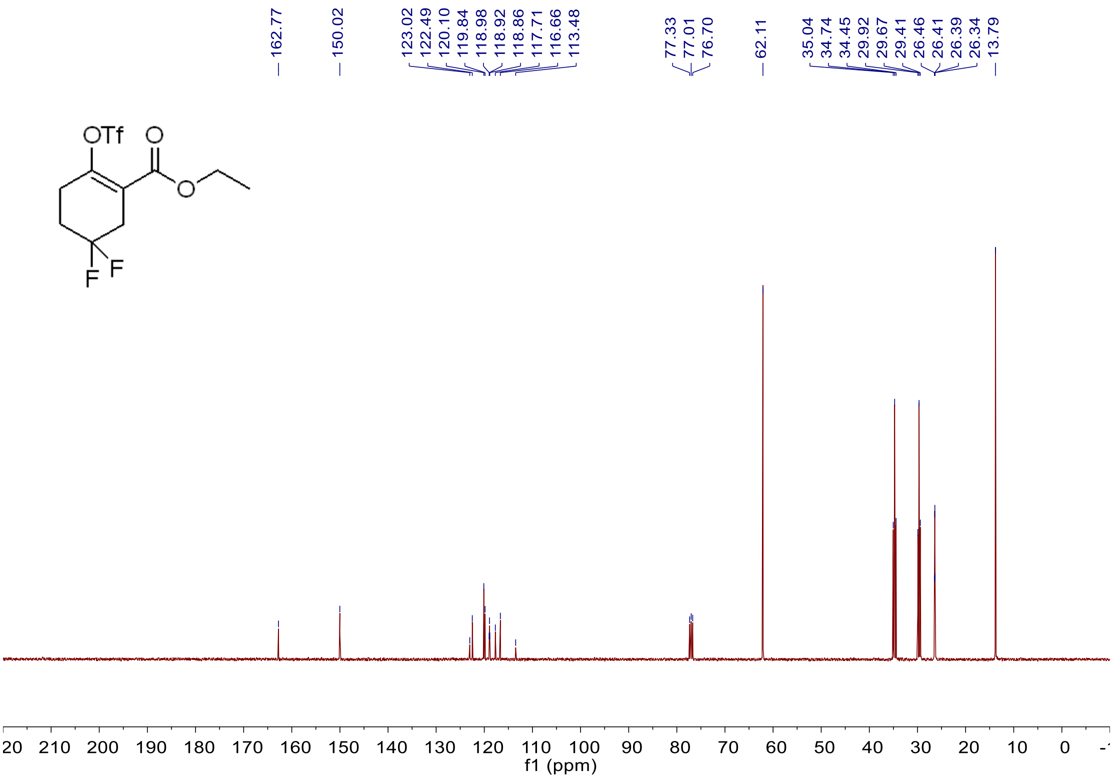

Supplementary Figure 24. ^{13}C NMR spectrum for compound **2c**

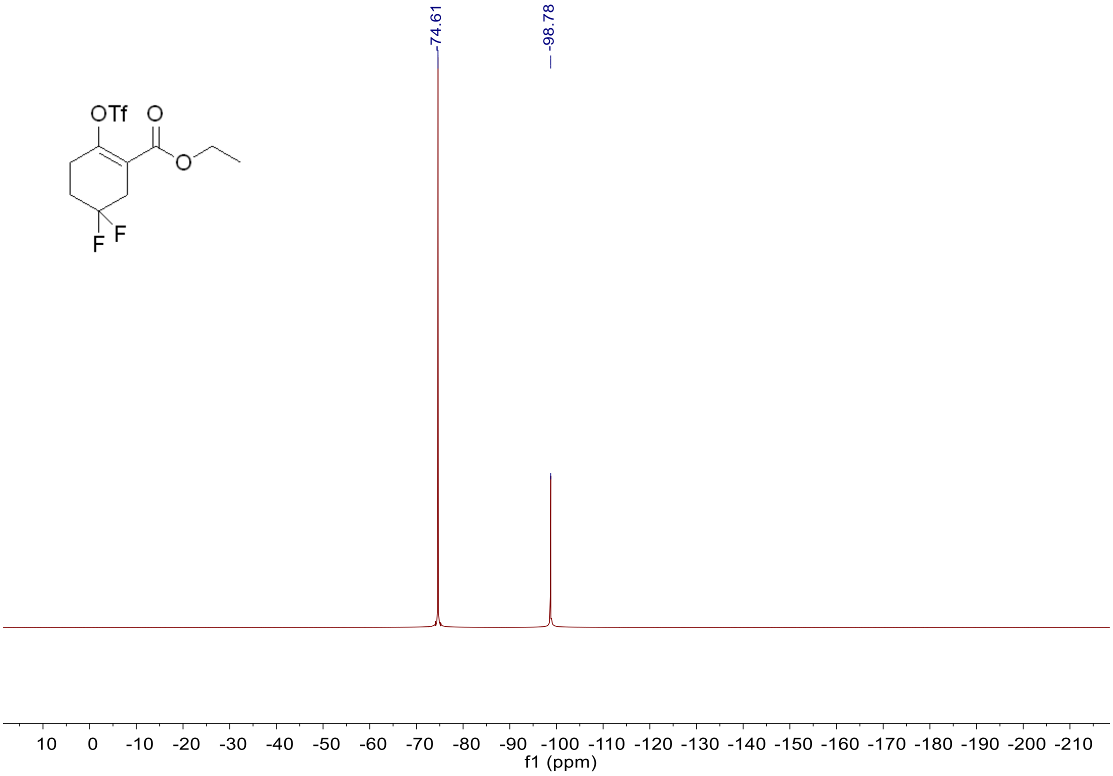

Supplementary Figure 25. ^{19}F NMR spectrum for compound **2c**

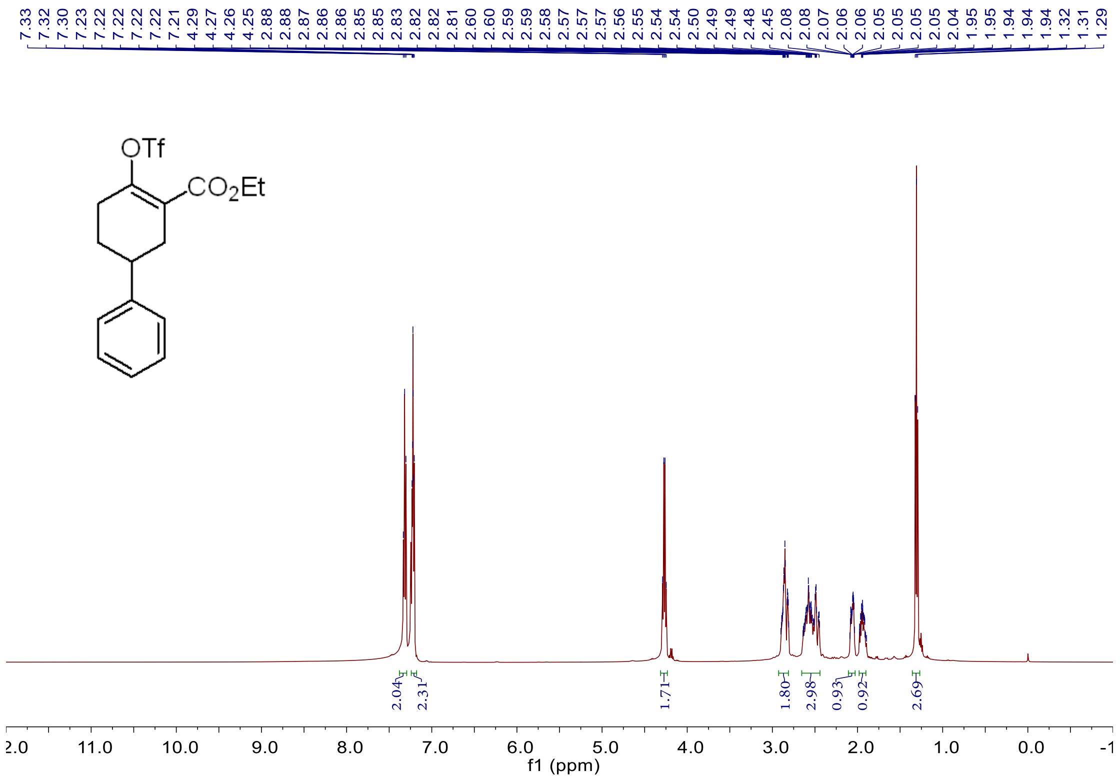

Supplementary Figure 26. ^{1}H NMR spectrum for compound **2d**

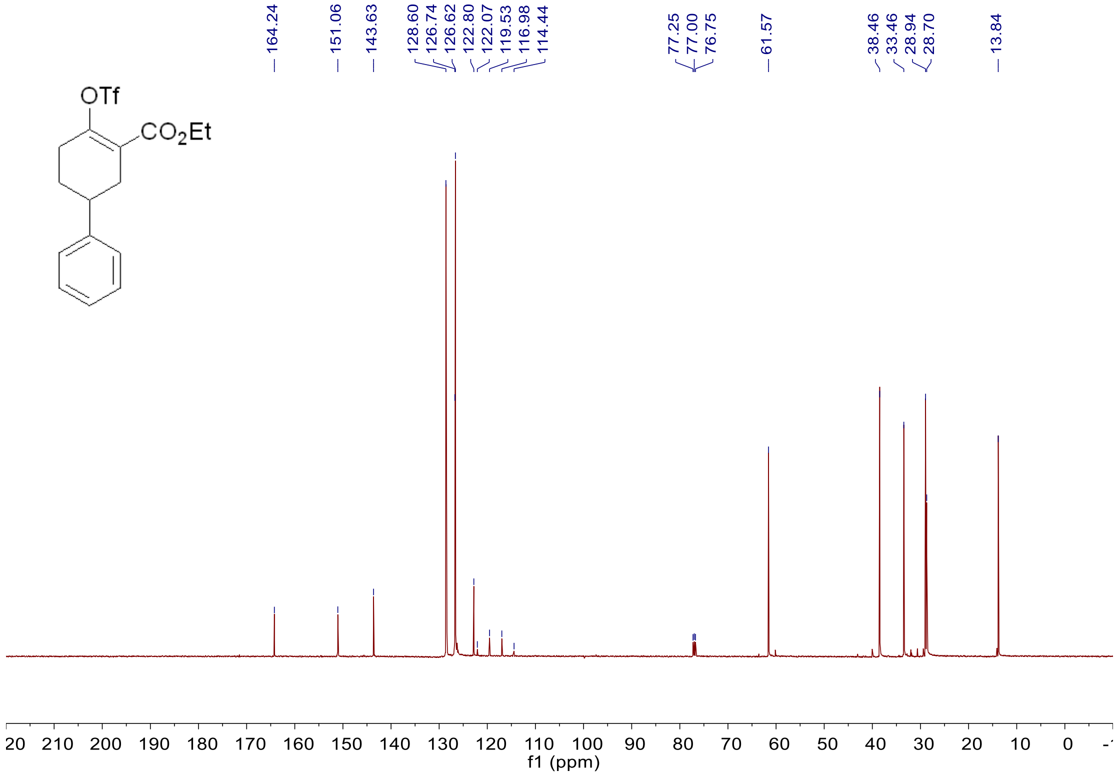

Supplementary Figure 27. ^{13}C NMR spectrum for compound **2d**

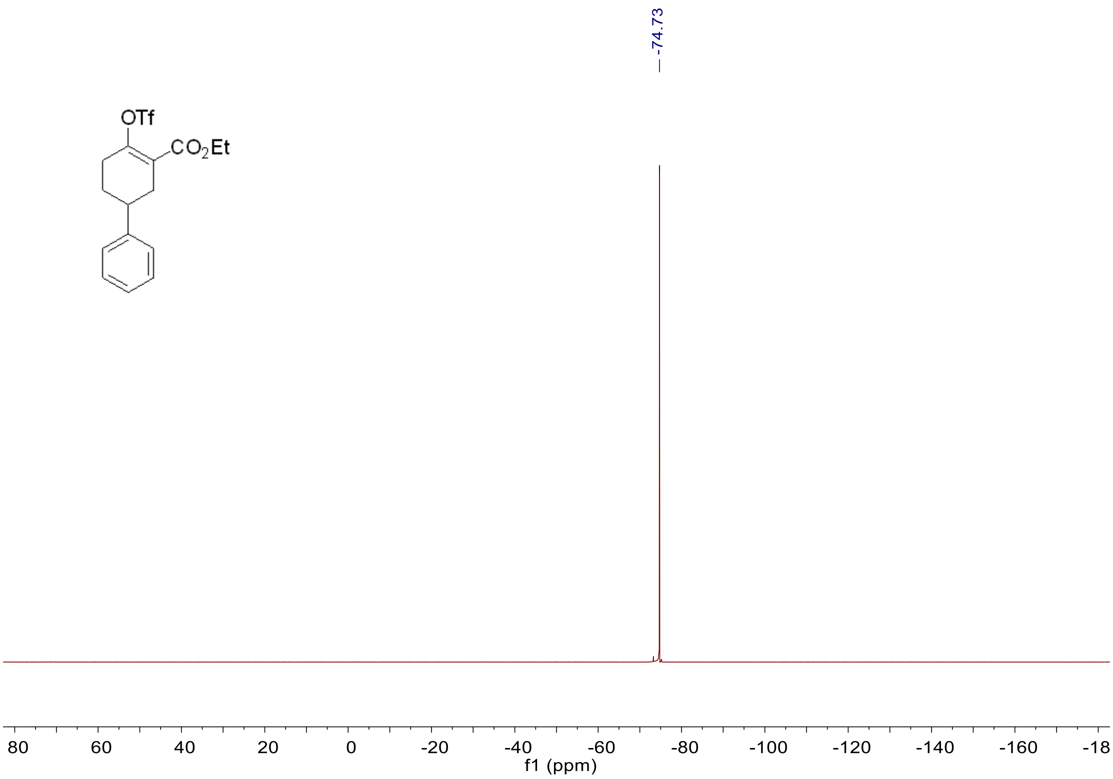

Supplementary Figure 28. ^{19}F NMR spectrum for compound **2d**

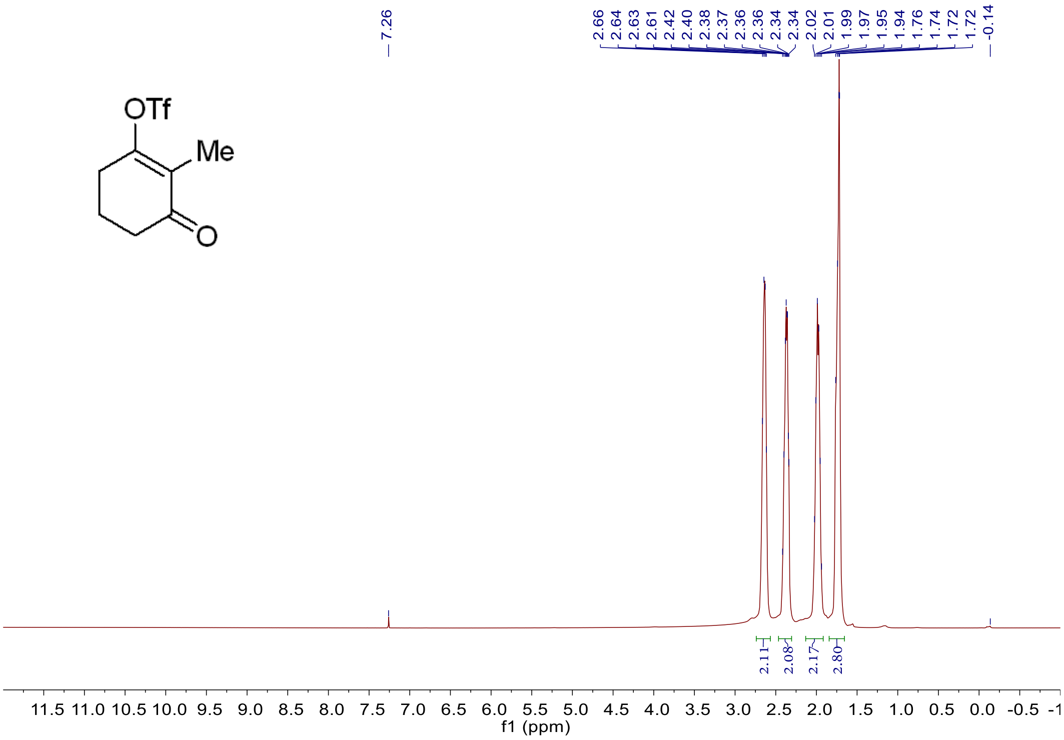

Supplementary Figure 29. ^{1}H NMR spectrum for compound **2e**

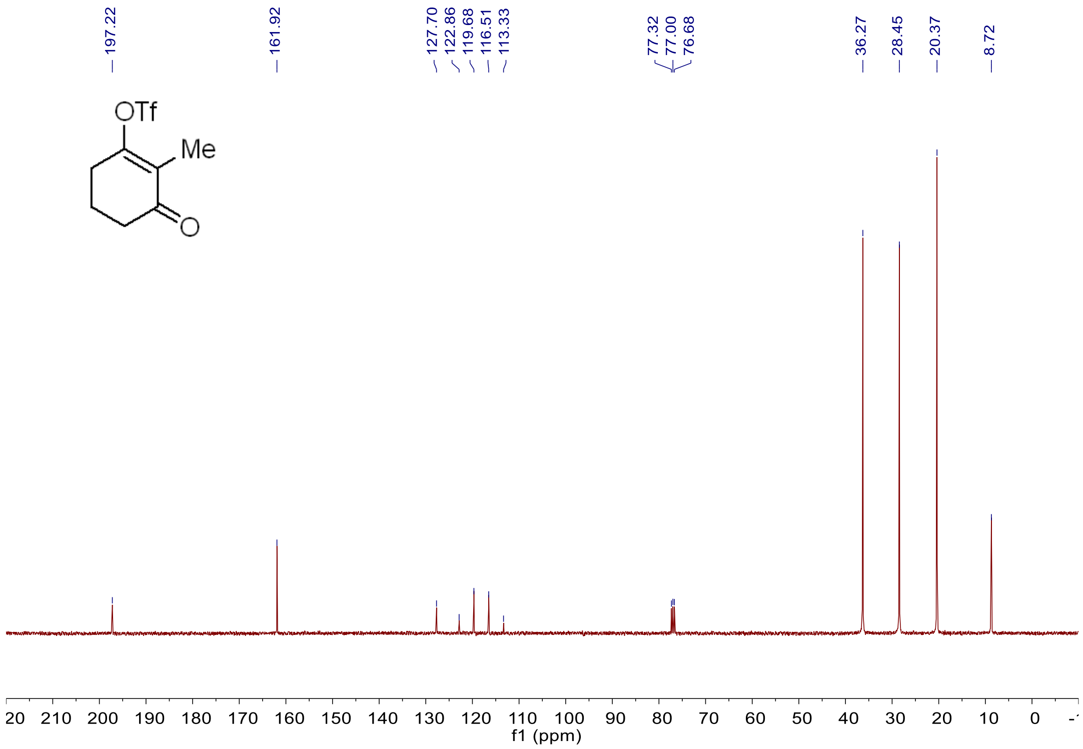

Supplementary Figure 30. ^{13}C NMR spectrum for compound **2e**

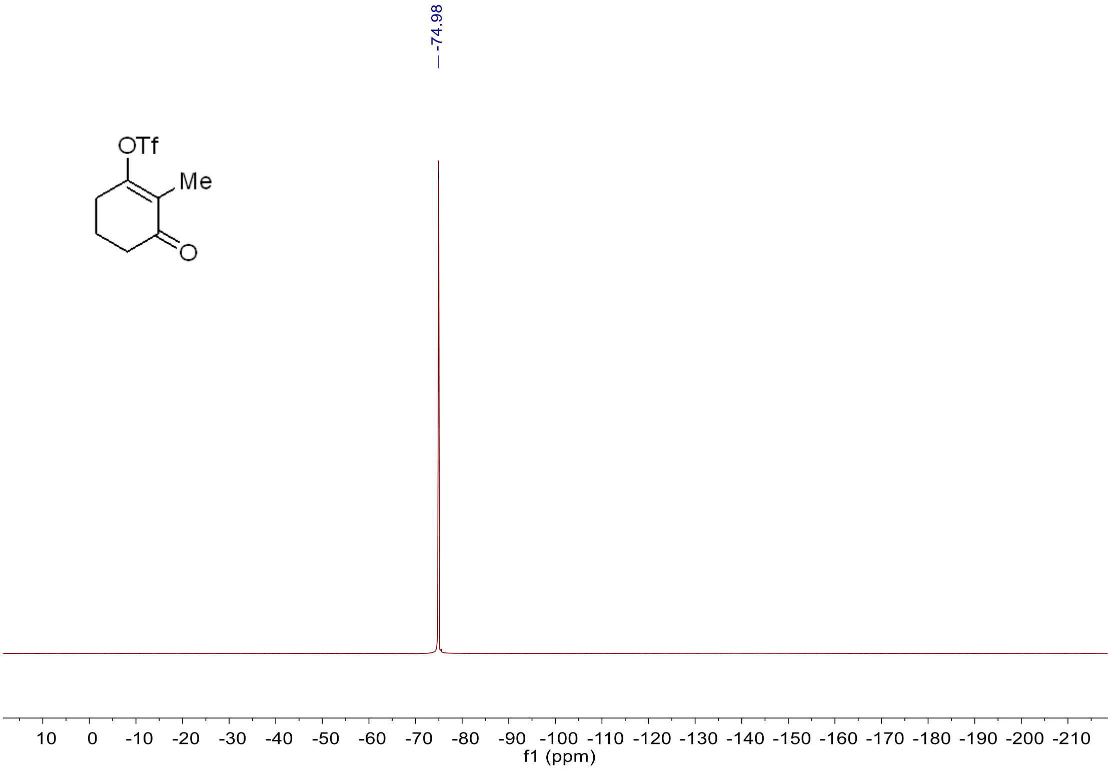

Supplementary Figure 31. ^{19}F NMR spectrum for compound **2e**

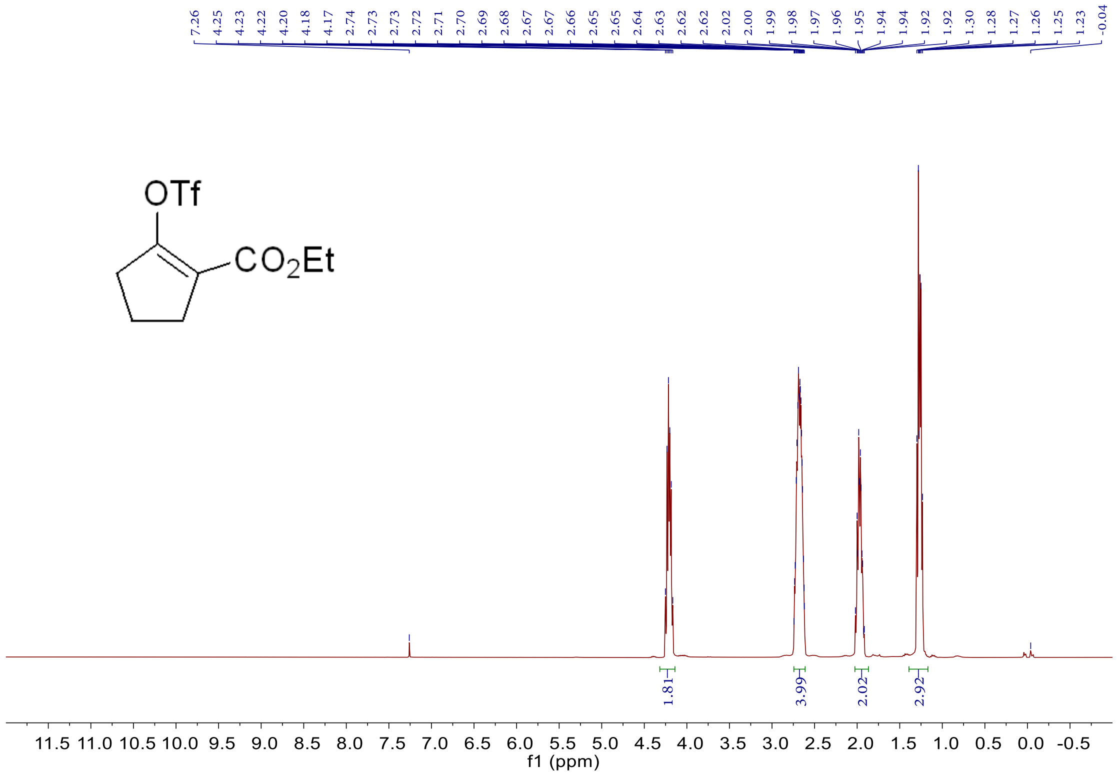

Supplementary Figure 32. ^{1}H NMR spectrum for compound **2f**

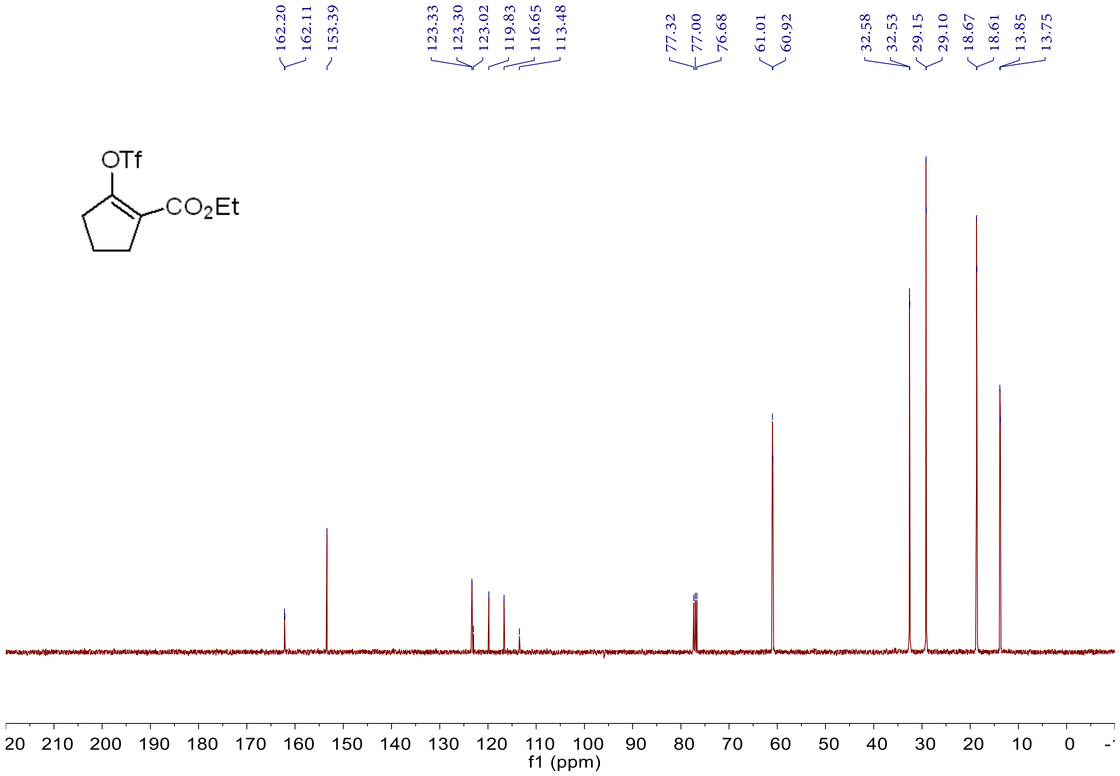

Supplementary Figure 33. ^{13}C NMR spectrum for compound **2f**

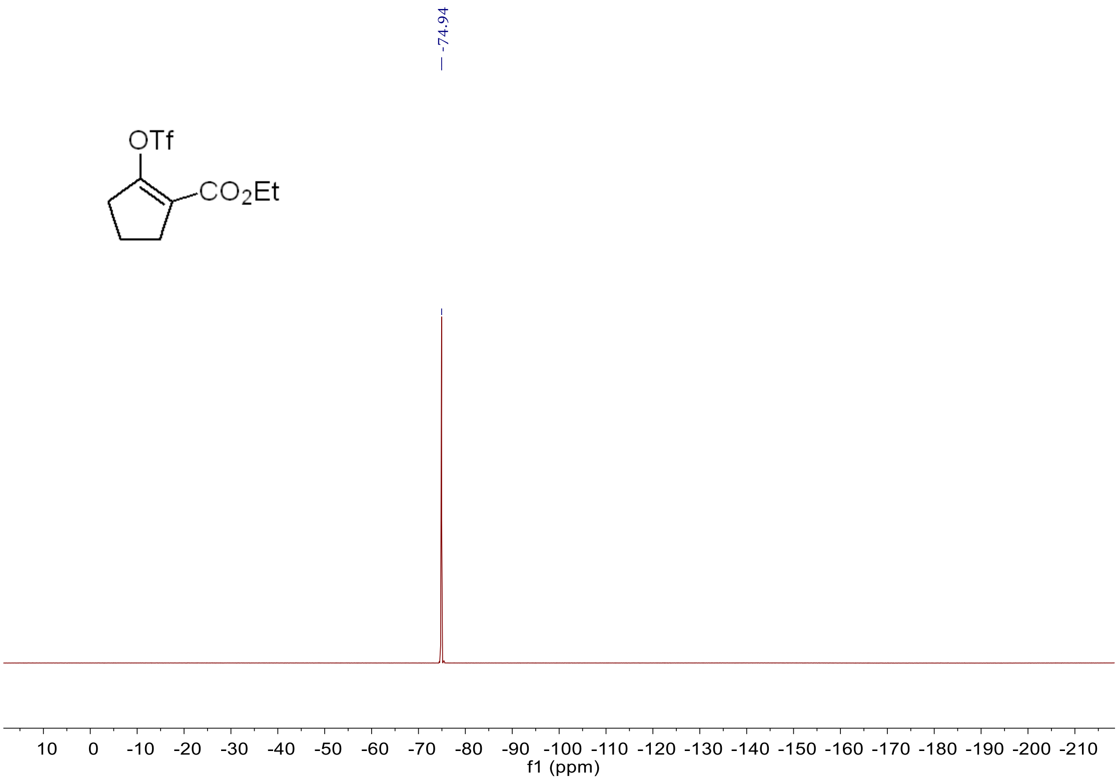

Supplementary Figure 34. ^{19}F NMR spectrum for compound **2f**

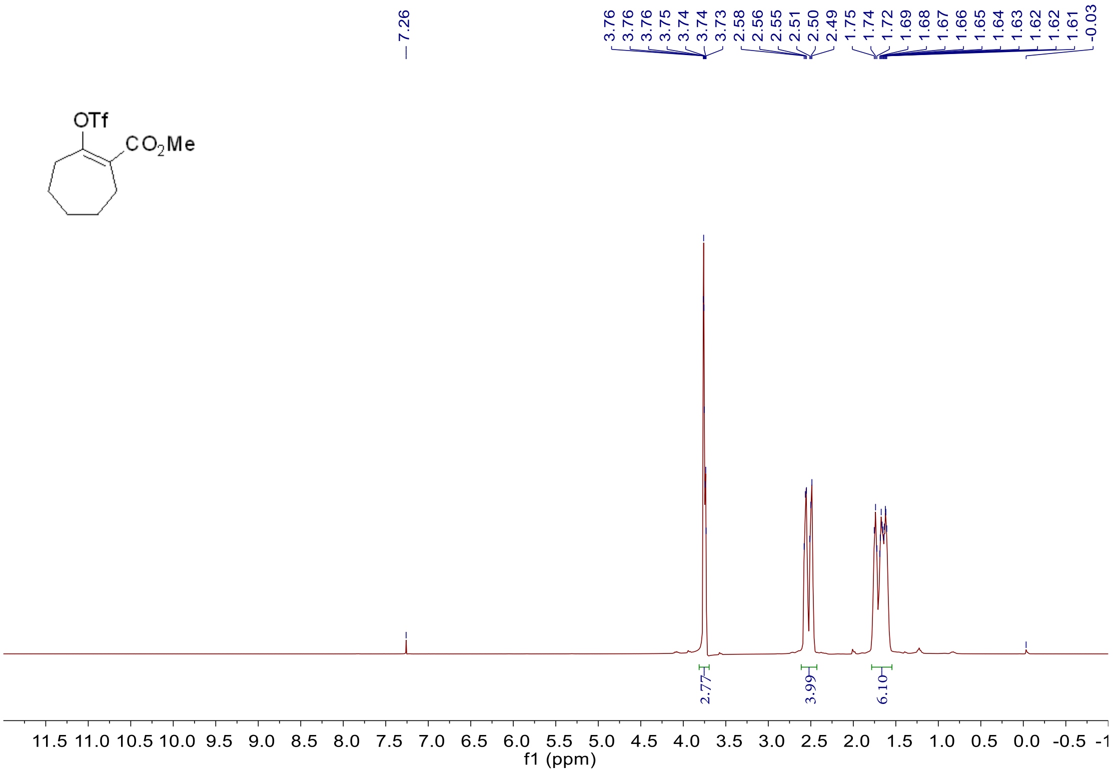

Supplementary Figure 35. ^{1}H NMR spectrum for compound **2g**

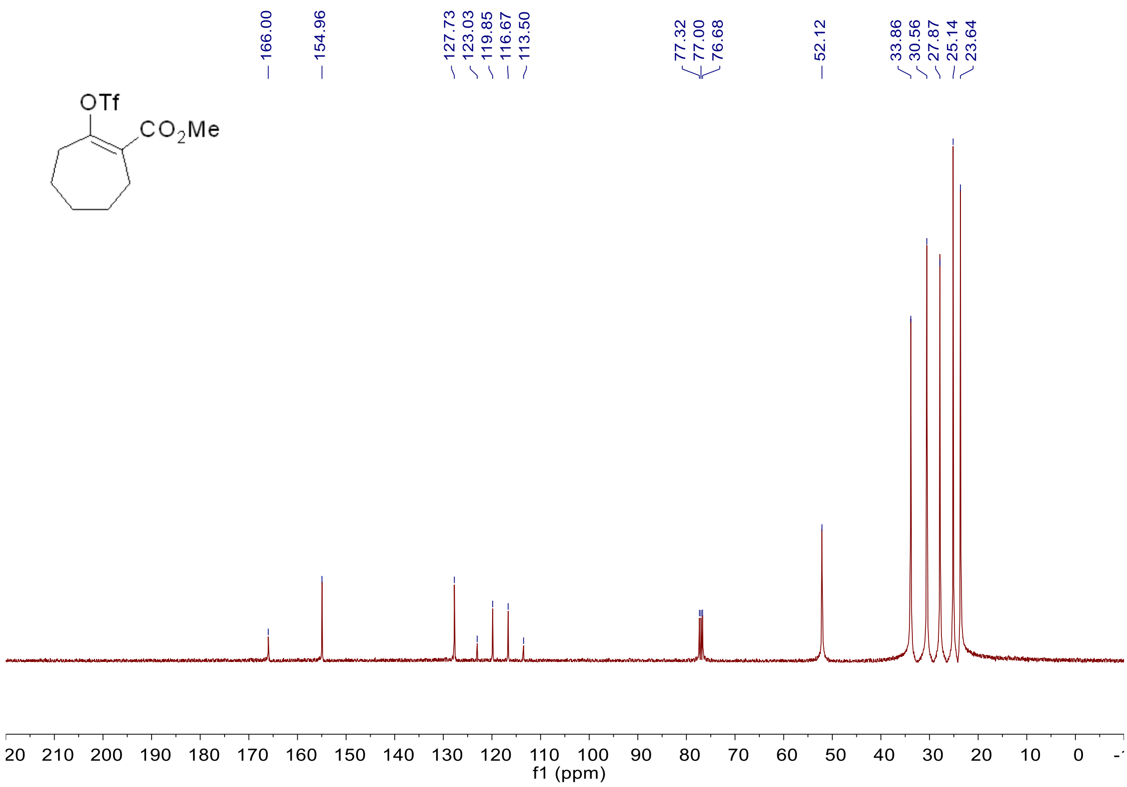

Supplementary Figure 36. ^{13}C NMR spectrum for compound **2g**

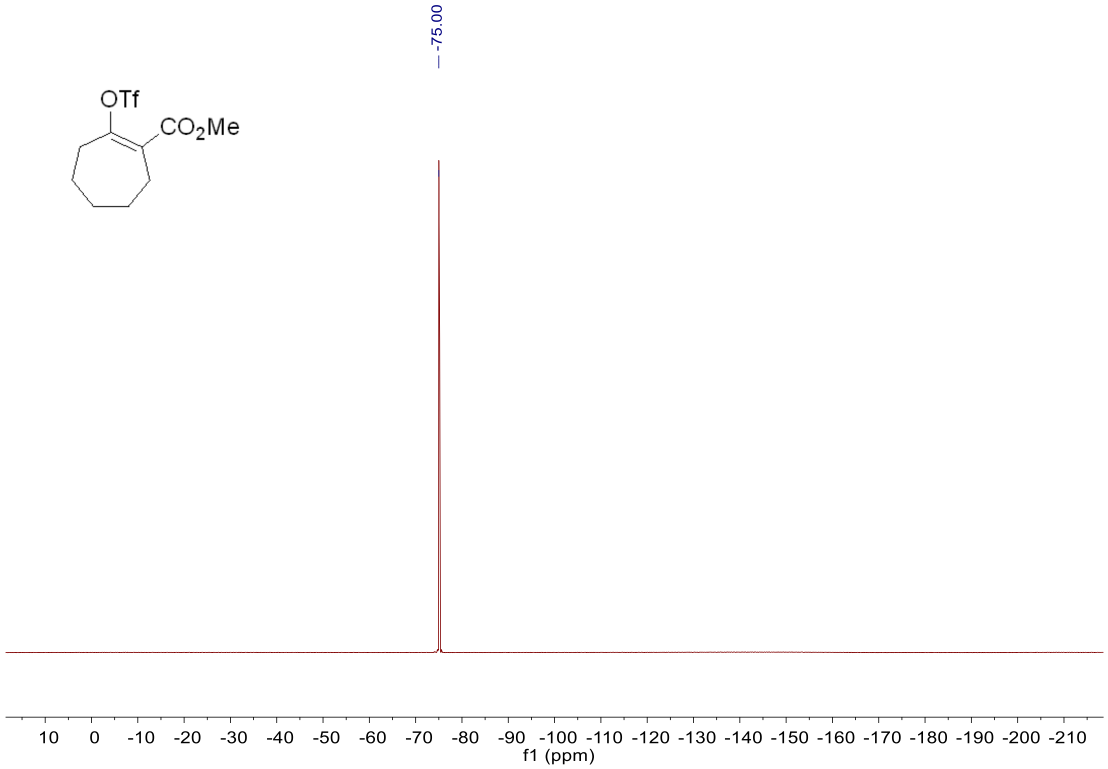

Supplementary Figure 37. ^{19}F NMR spectrum for compound **2g**

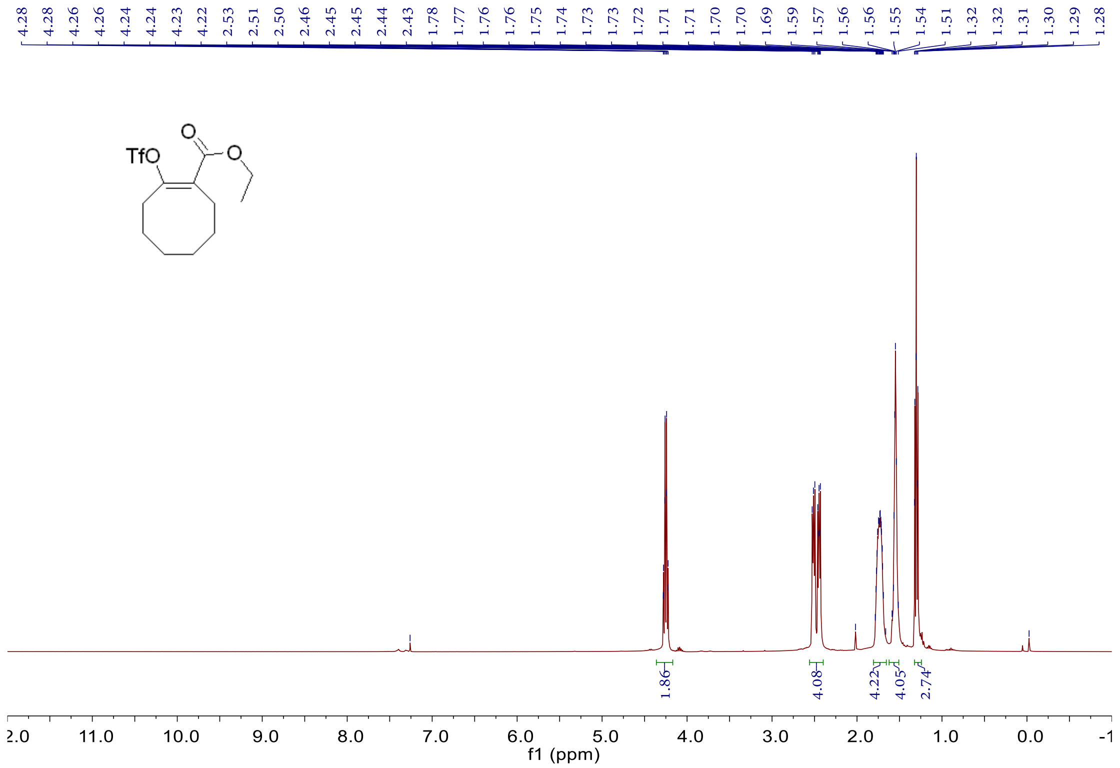

Supplementary Figure 38. ^{1}H NMR spectrum for compound **2h**

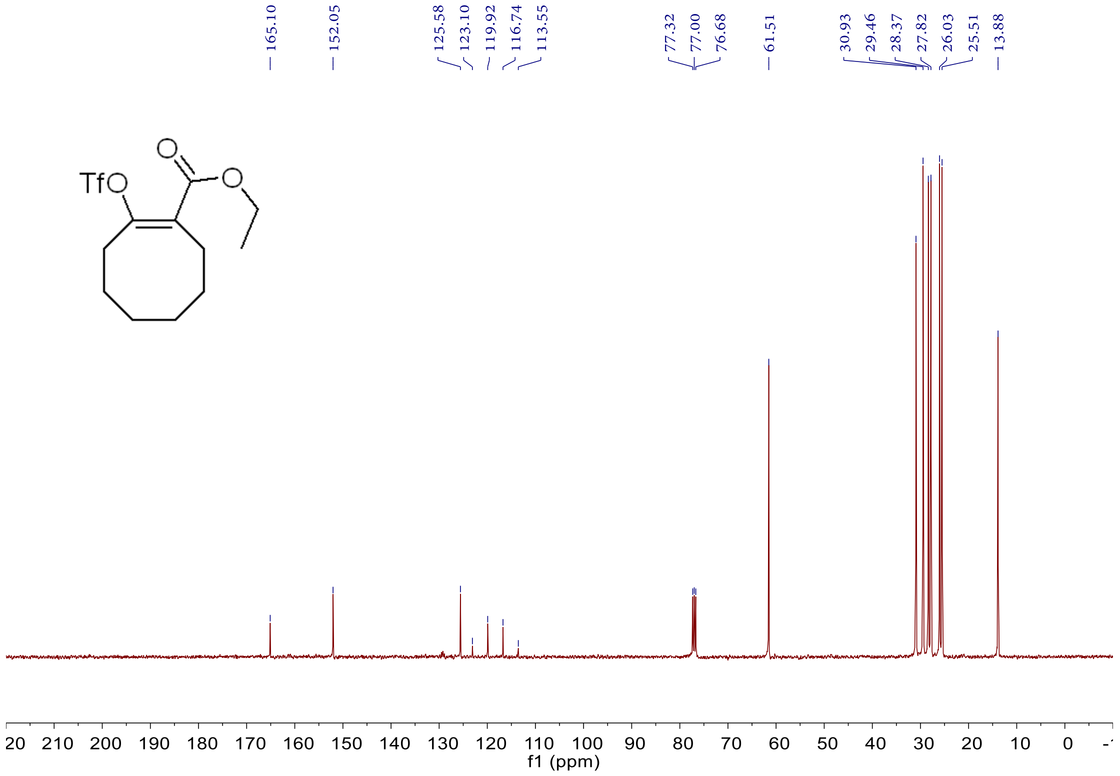

Supplementary Figure 39. ^{13}C NMR spectrum for compound **2h**

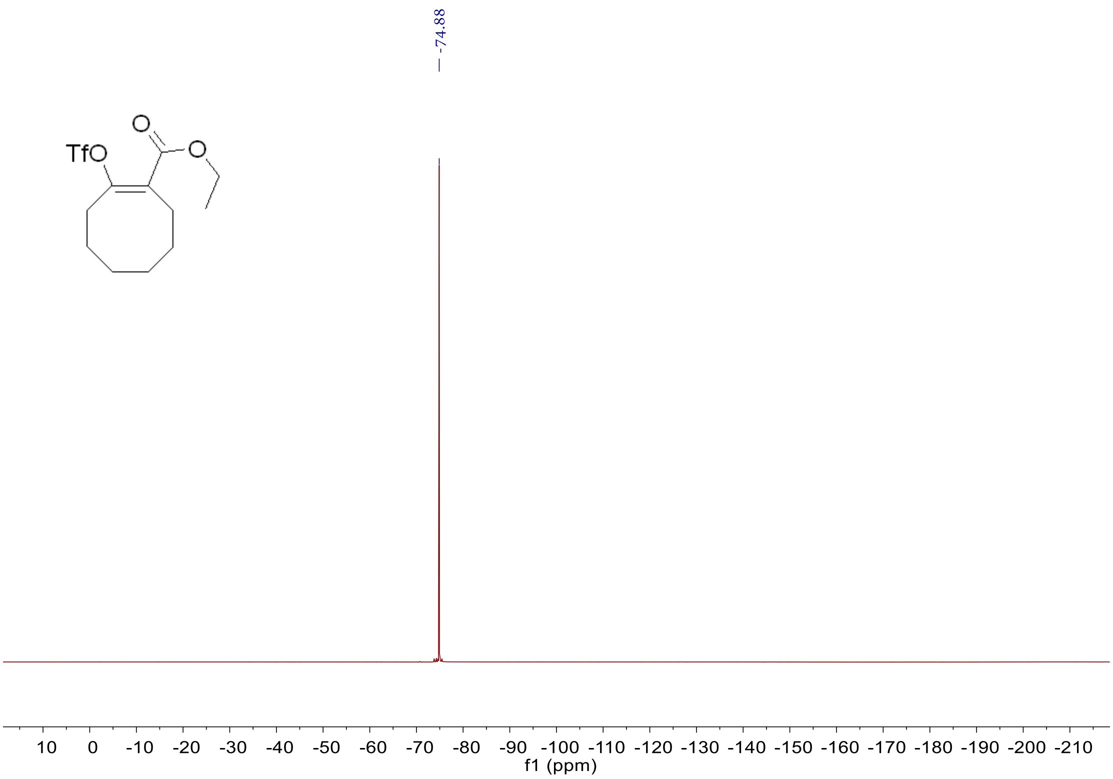

Supplementary Figure 40. ^{19}F NMR spectrum for compound **2h**

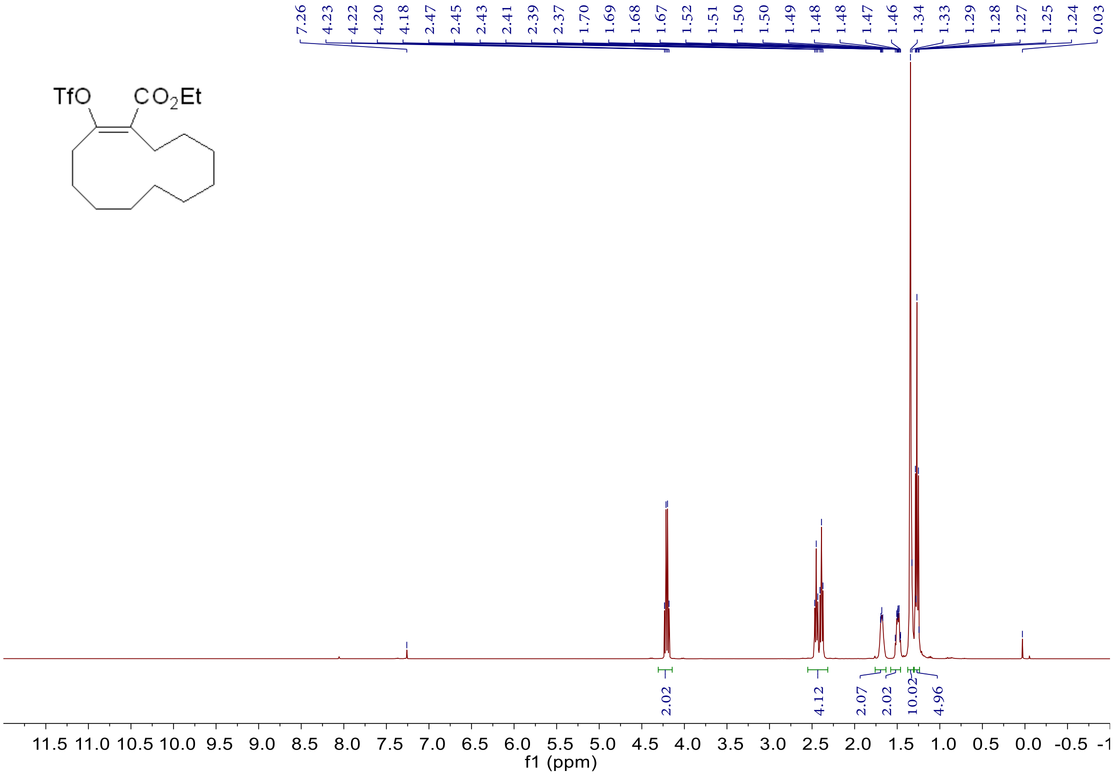

Supplementary Figure 41. ^{1}H NMR spectrum for compound **2i**

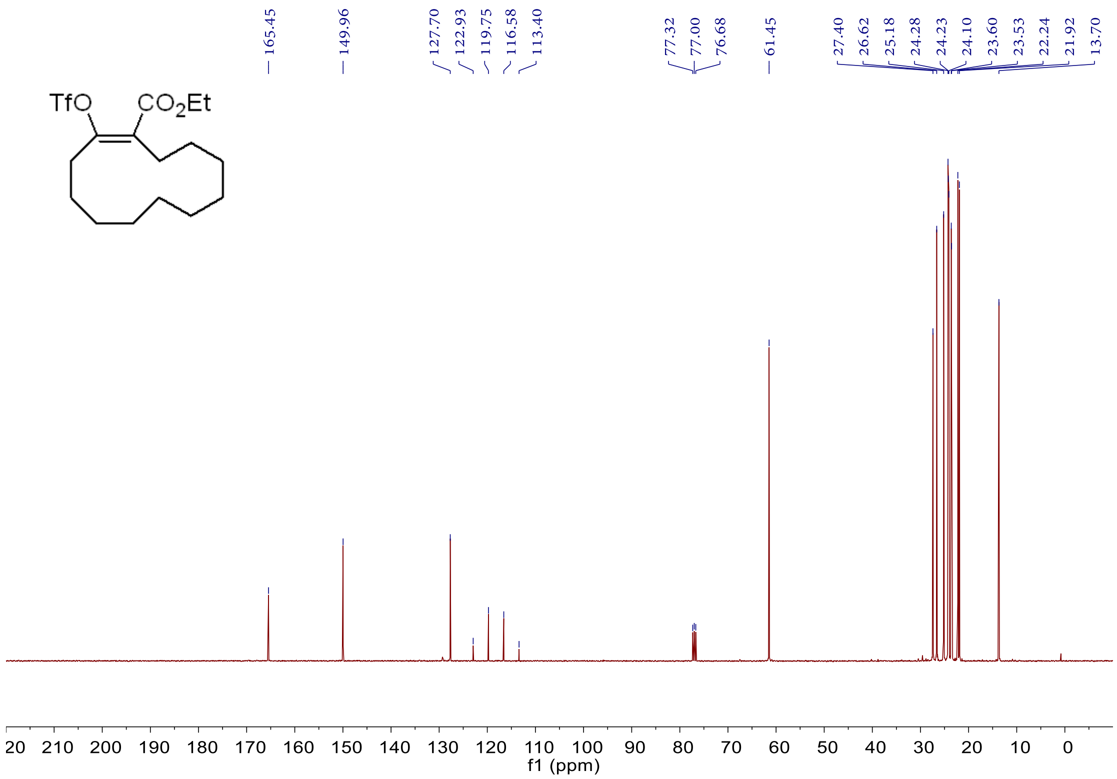

Supplementary Figure 42. ^{13}C NMR spectrum for compound **2i**

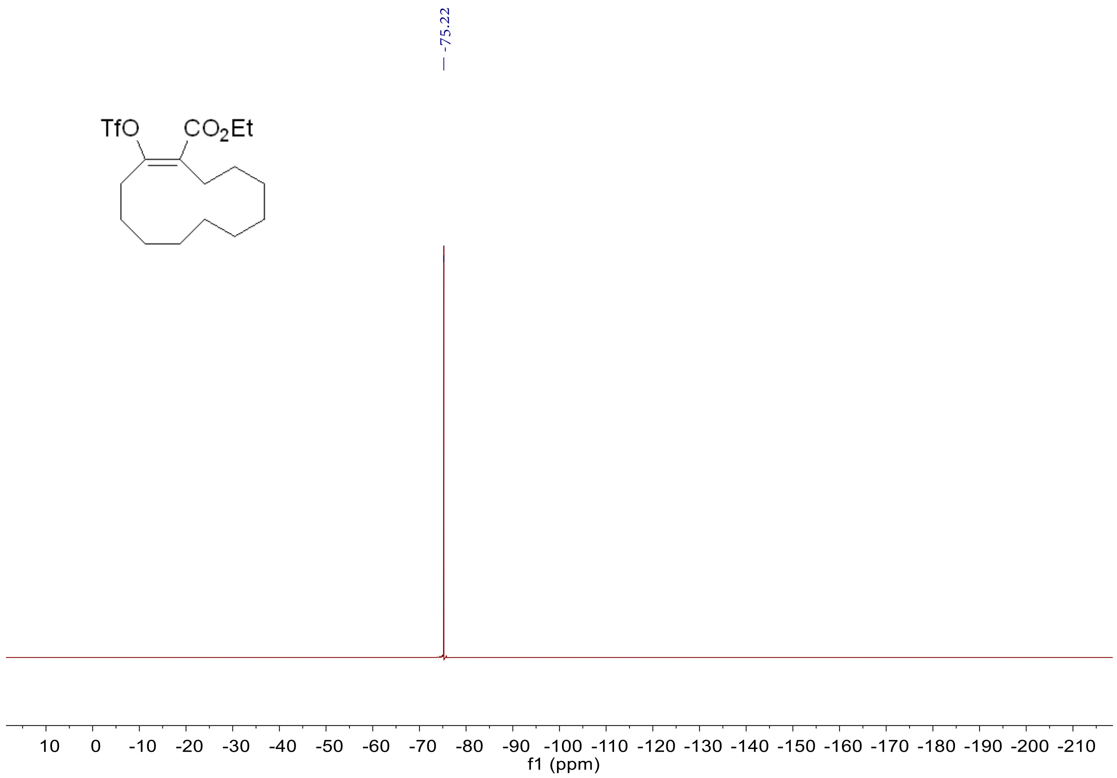

Supplementary Figure 43. ^{19}F NMR spectrum for compound **2i**

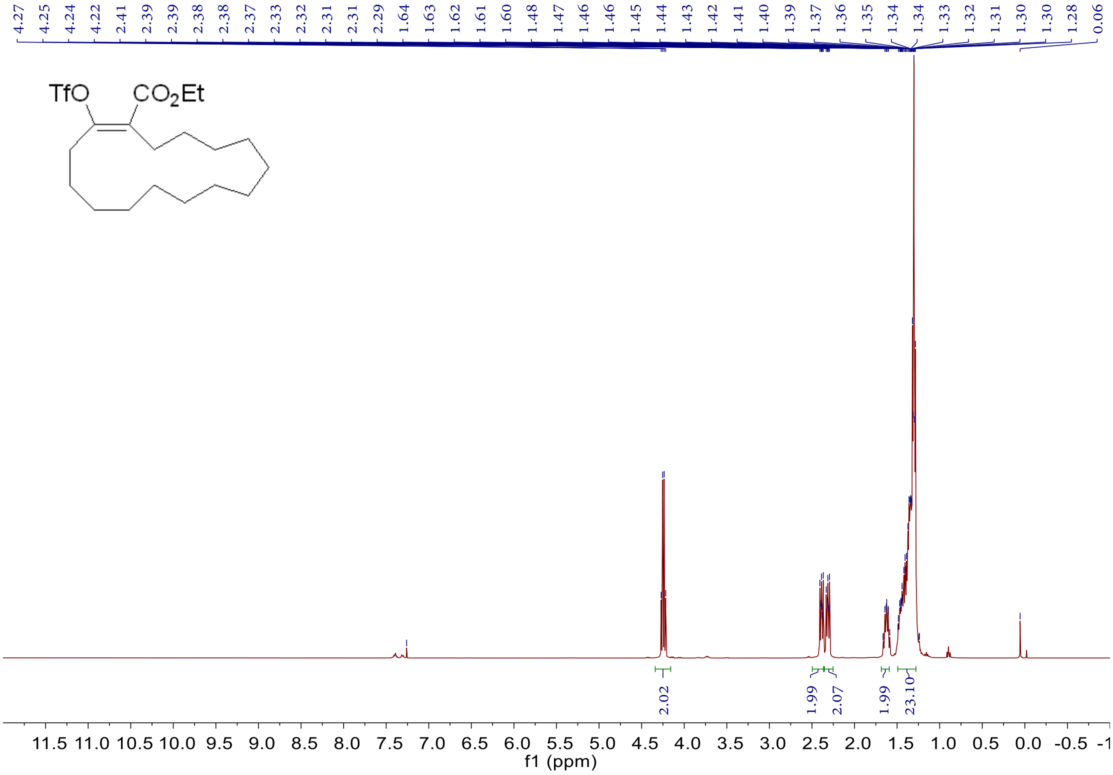

Supplementary Figure 44. ^{1}H NMR spectrum for compound **2j**

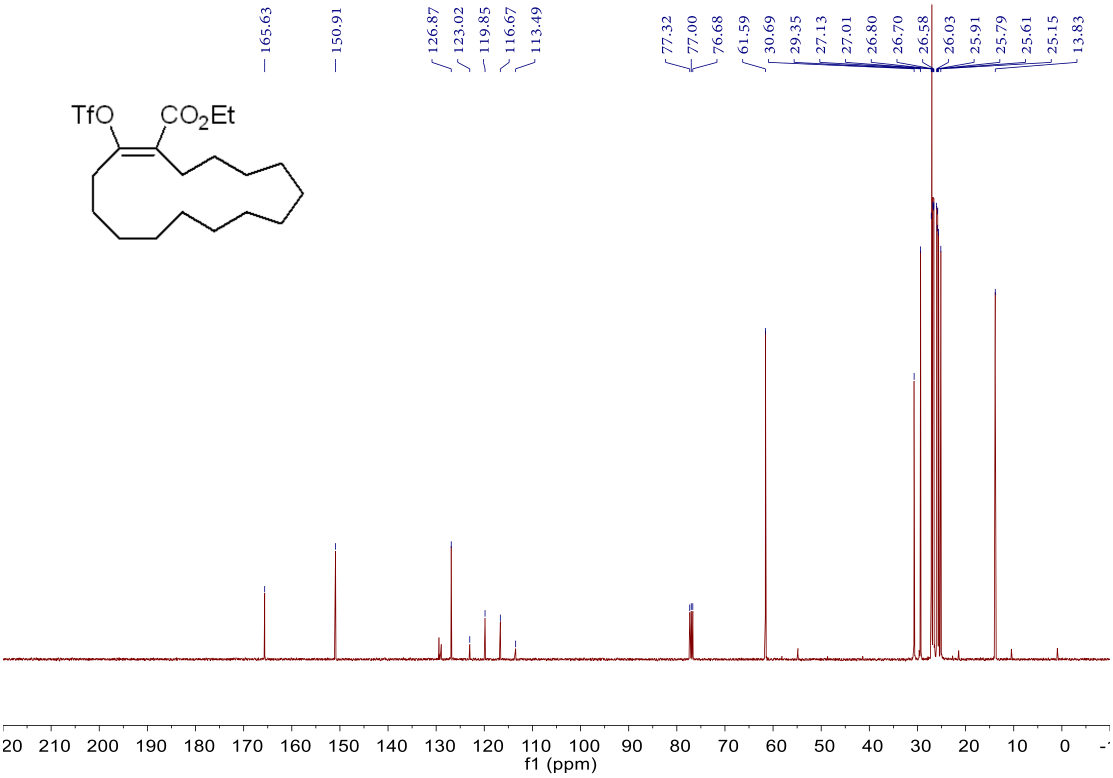

Supplementary Figure 45. ^{13}C NMR spectrum for compound **2j**

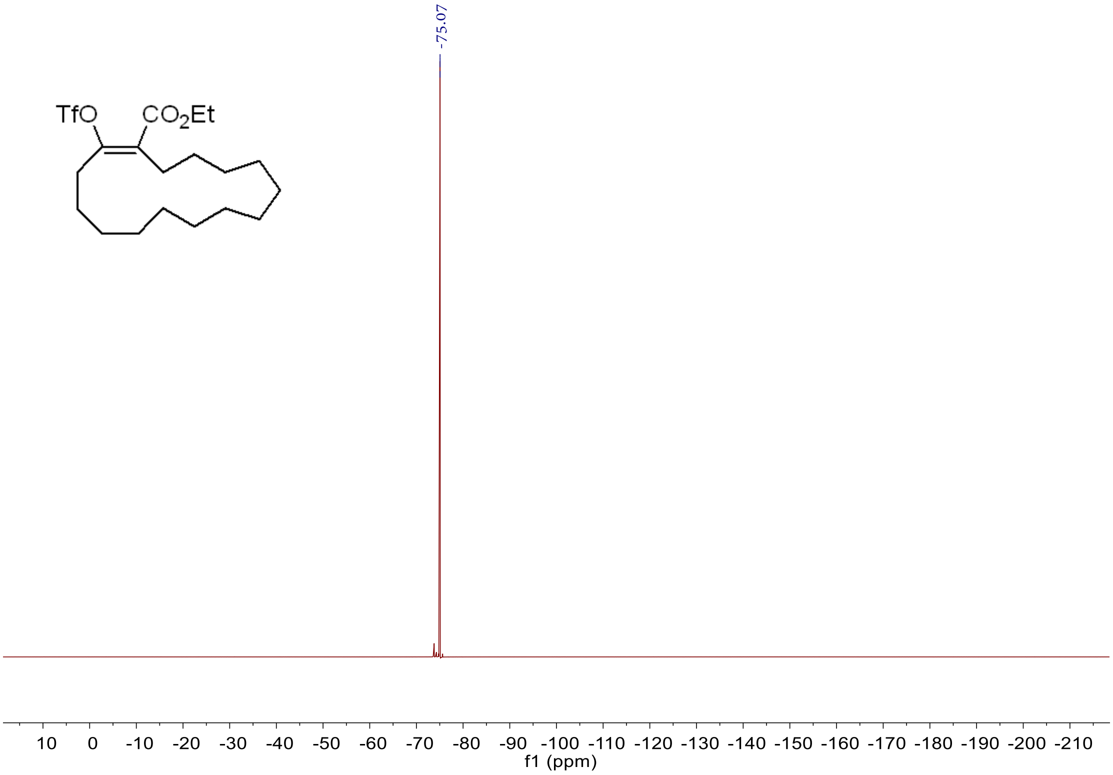

Supplementary Figure 46. ^{19}F NMR spectrum for compound **2j**

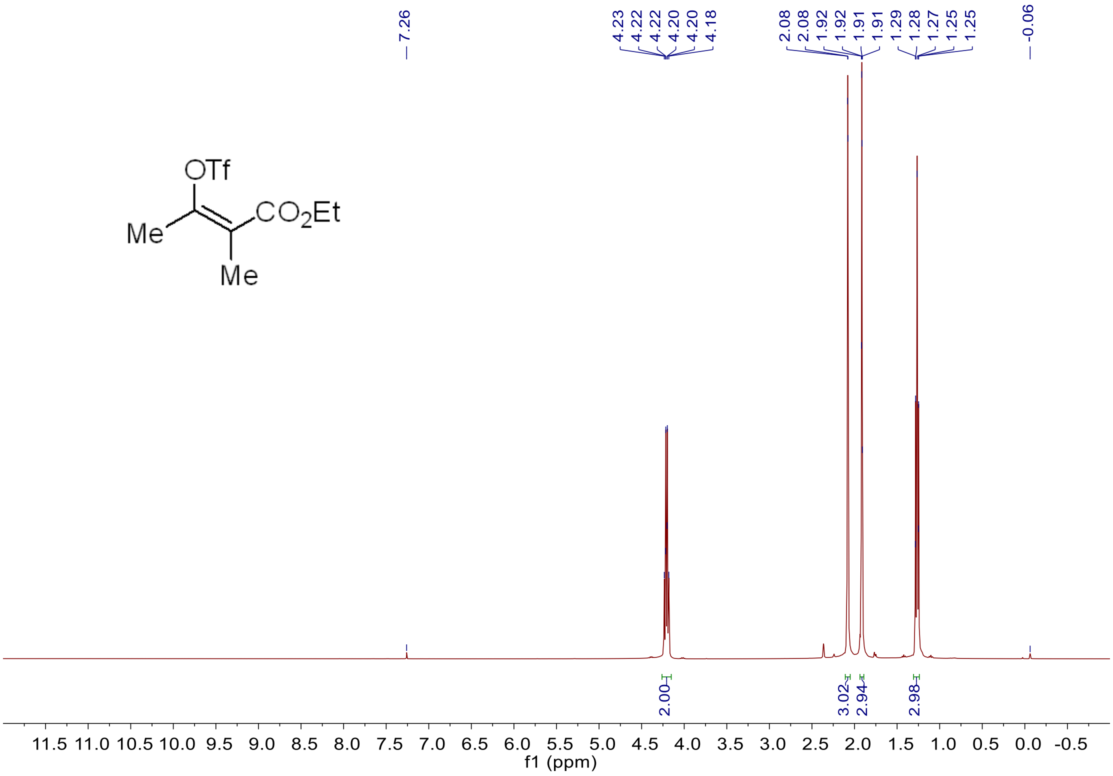

Supplementary Figure 47. ^{1}H NMR spectrum for compound **2k**

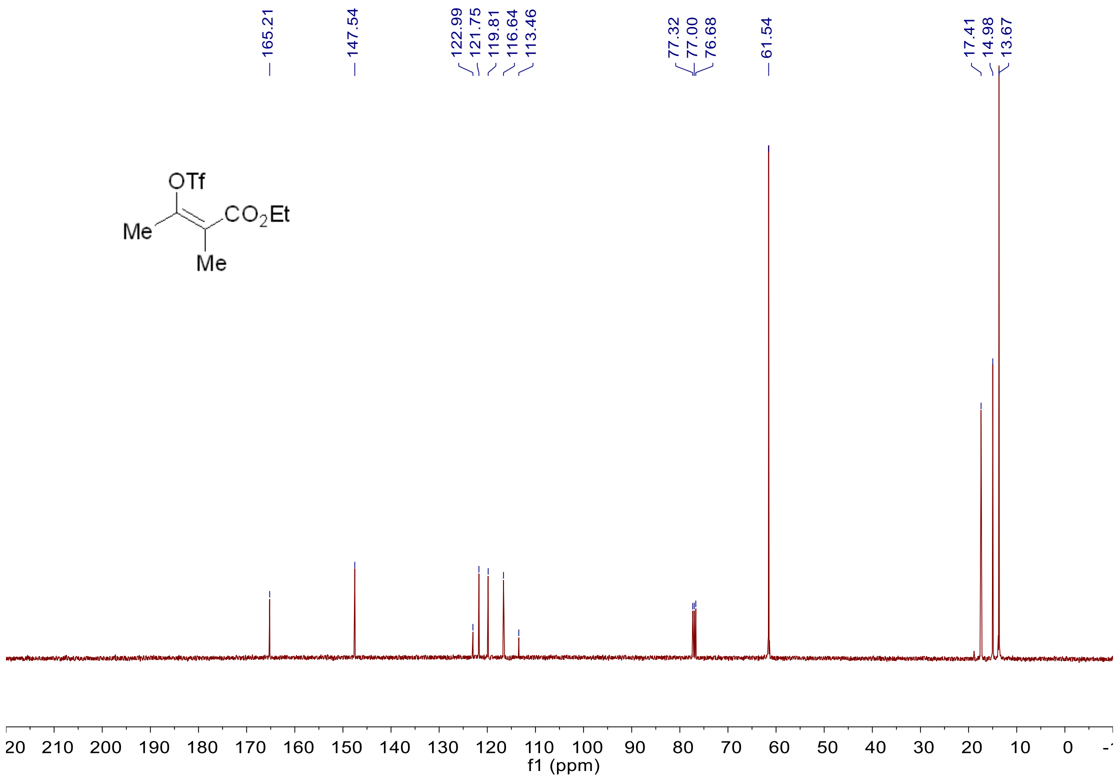

Supplementary Figure 48. ^{13}C NMR spectrum for compound **2k**

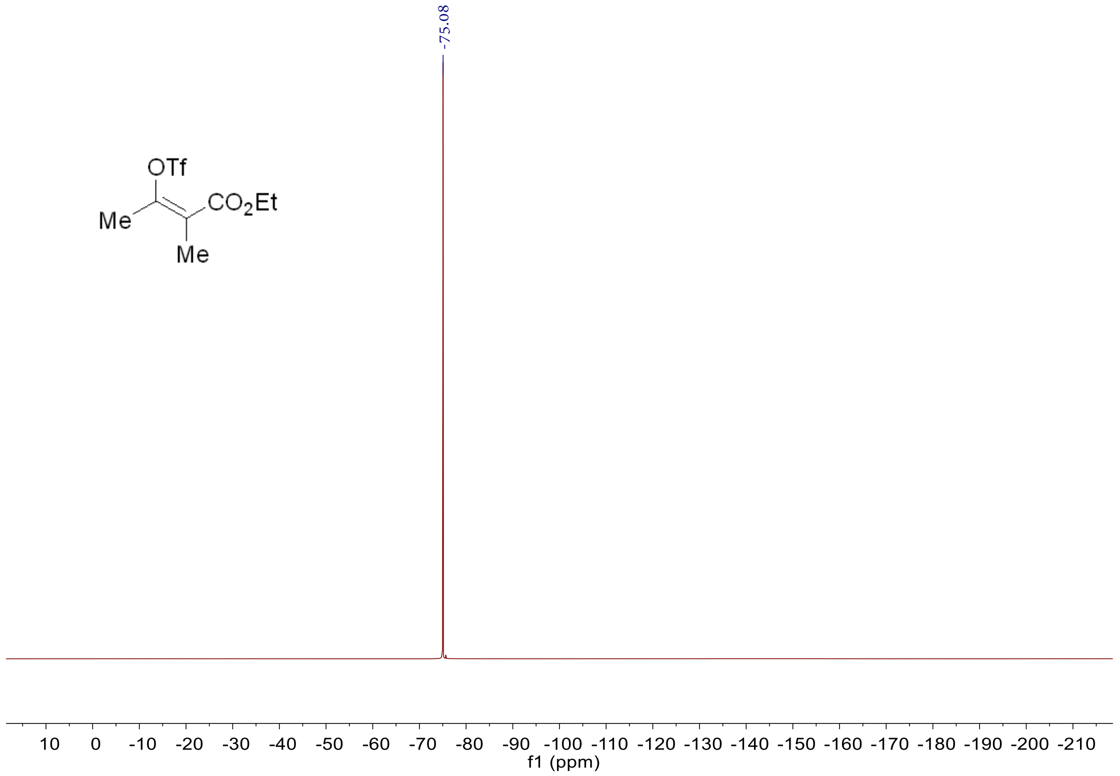

Supplementary Figure 49. ^{19}F NMR spectrum for compound **2k**

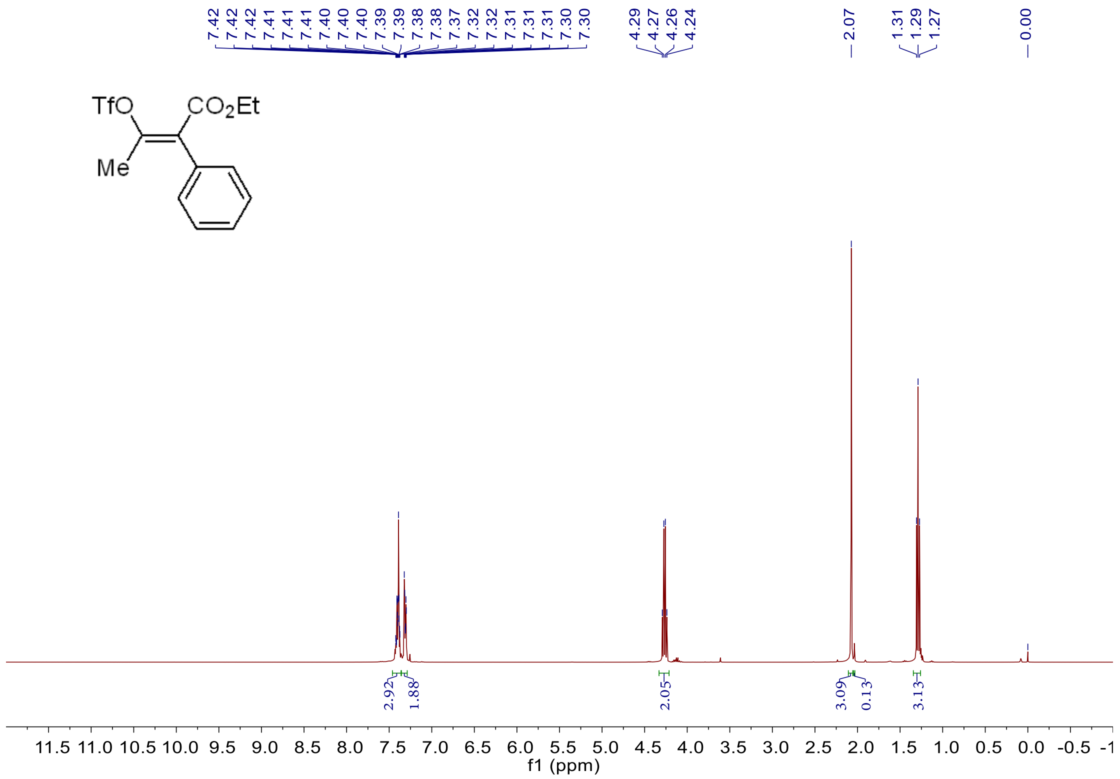

Supplementary Figure 50. ^{1}H NMR spectrum for compound **2l**

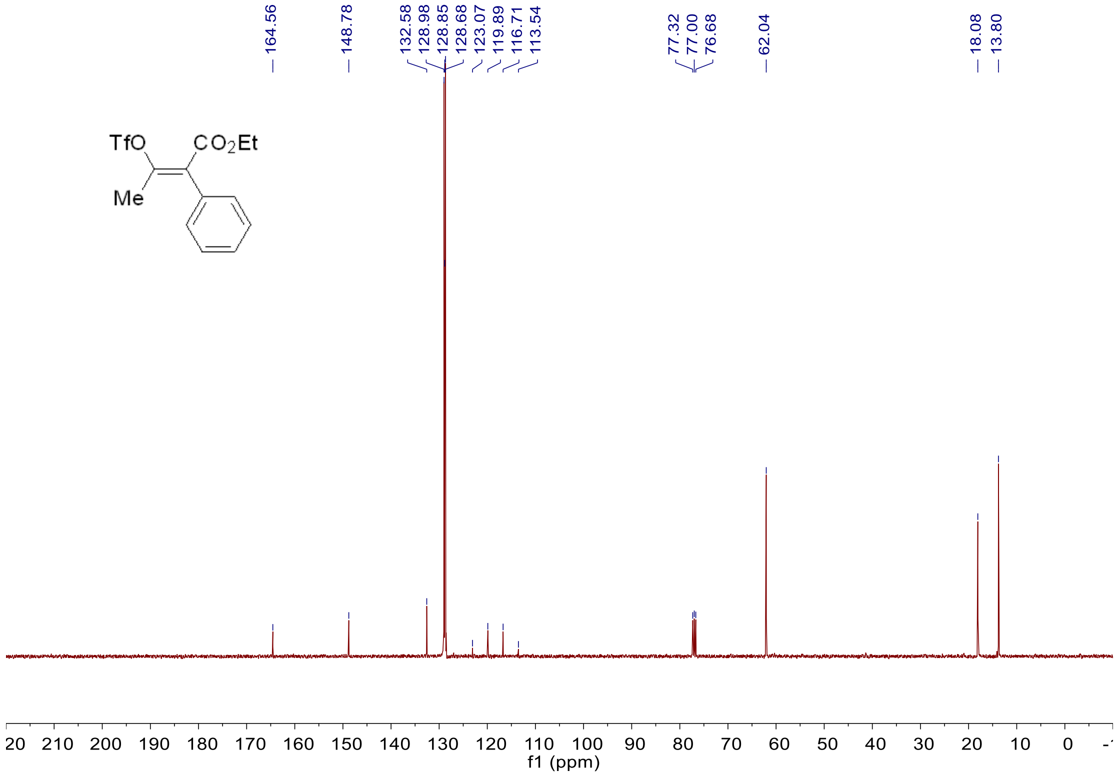

Supplementary Figure 51. ^{13}C NMR spectrum for compound **2l**

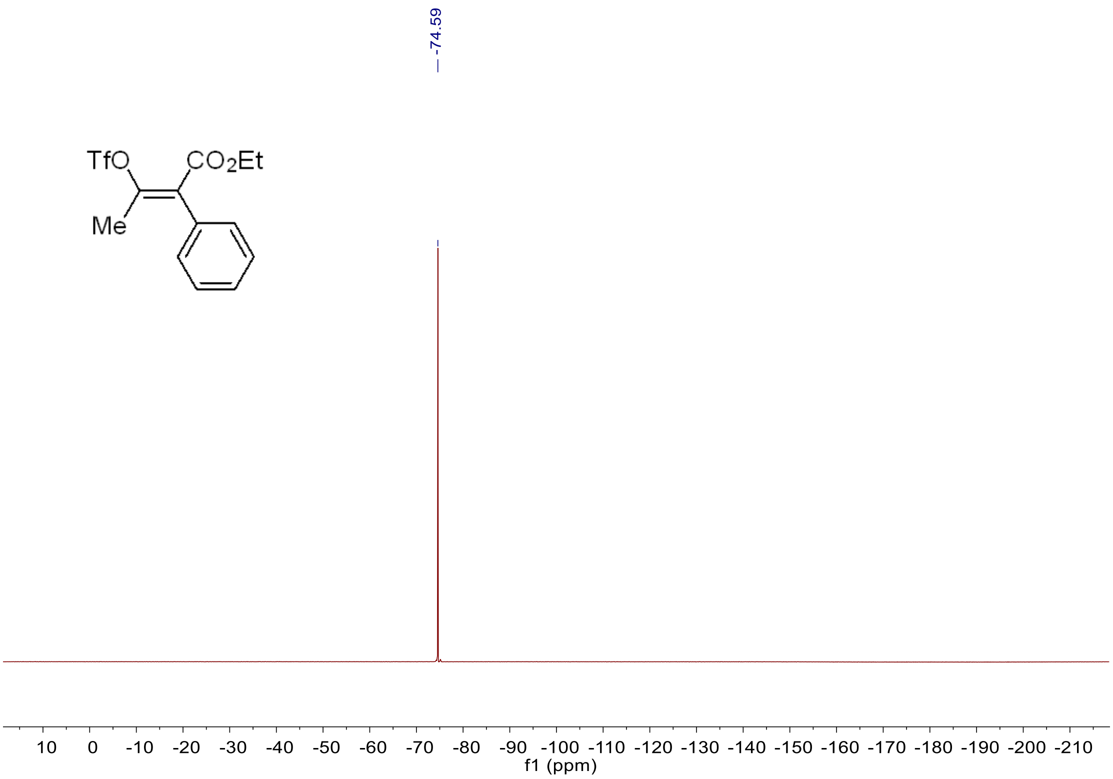

Supplementary Figure 52. ^{19}F NMR spectrum for compound **2l**

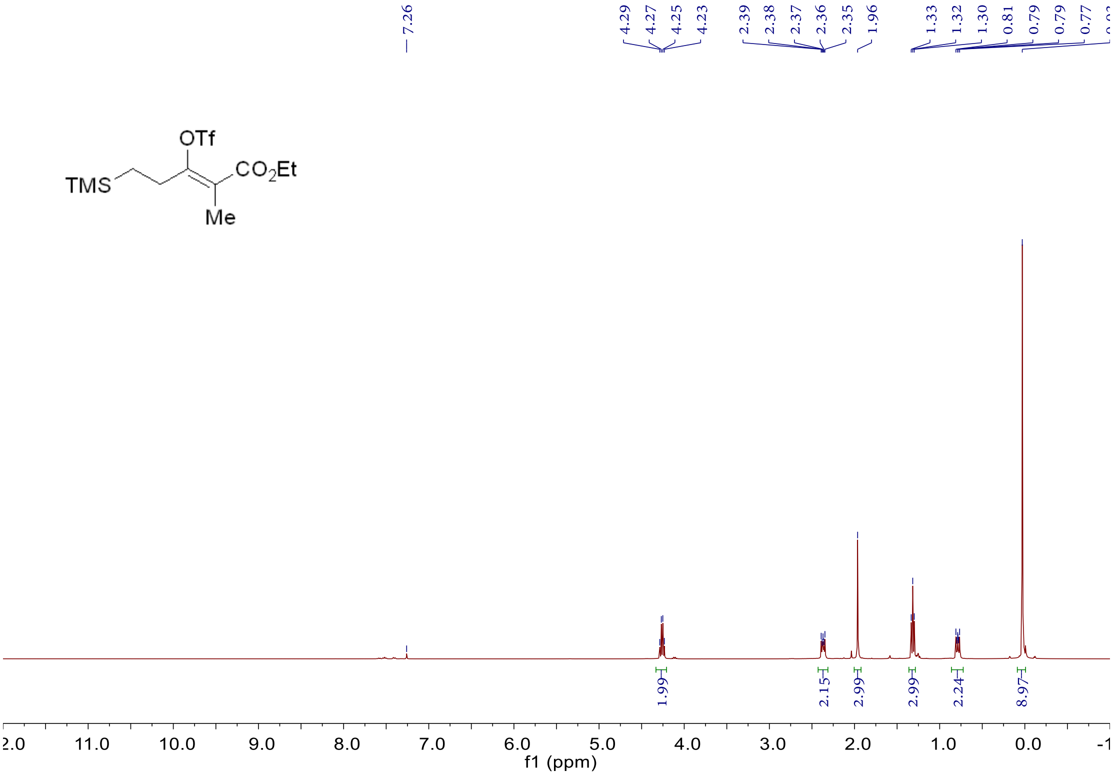

Supplementary Figure 53. ^{1}H NMR spectrum for compound **2m**

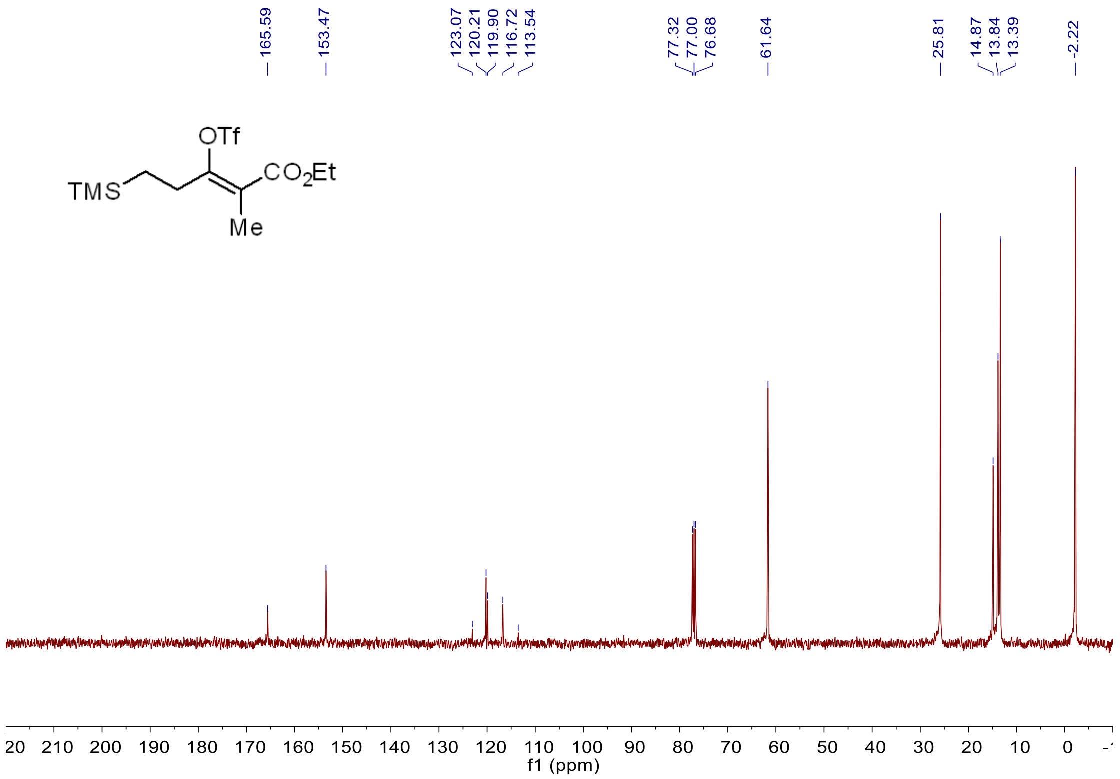

Supplementary Figure 54. ^{13}C NMR spectrum for compound **2m**

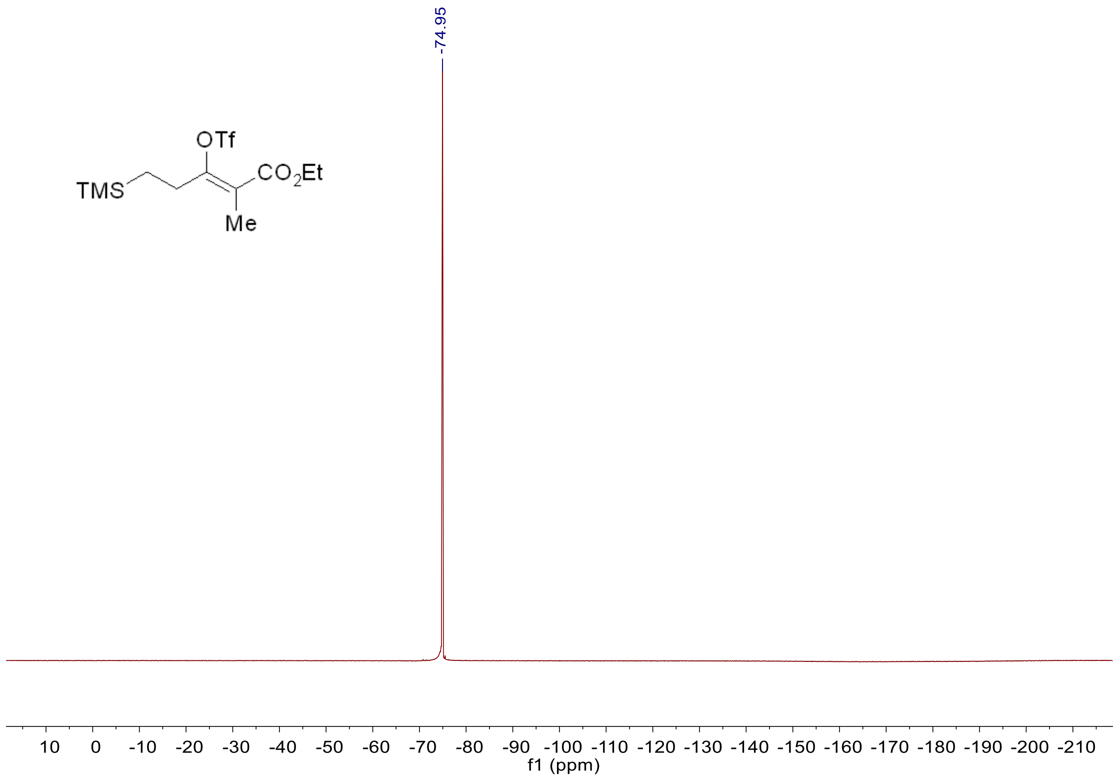

Supplementary Figure 55. ^{19}F NMR spectrum for compound **2m**

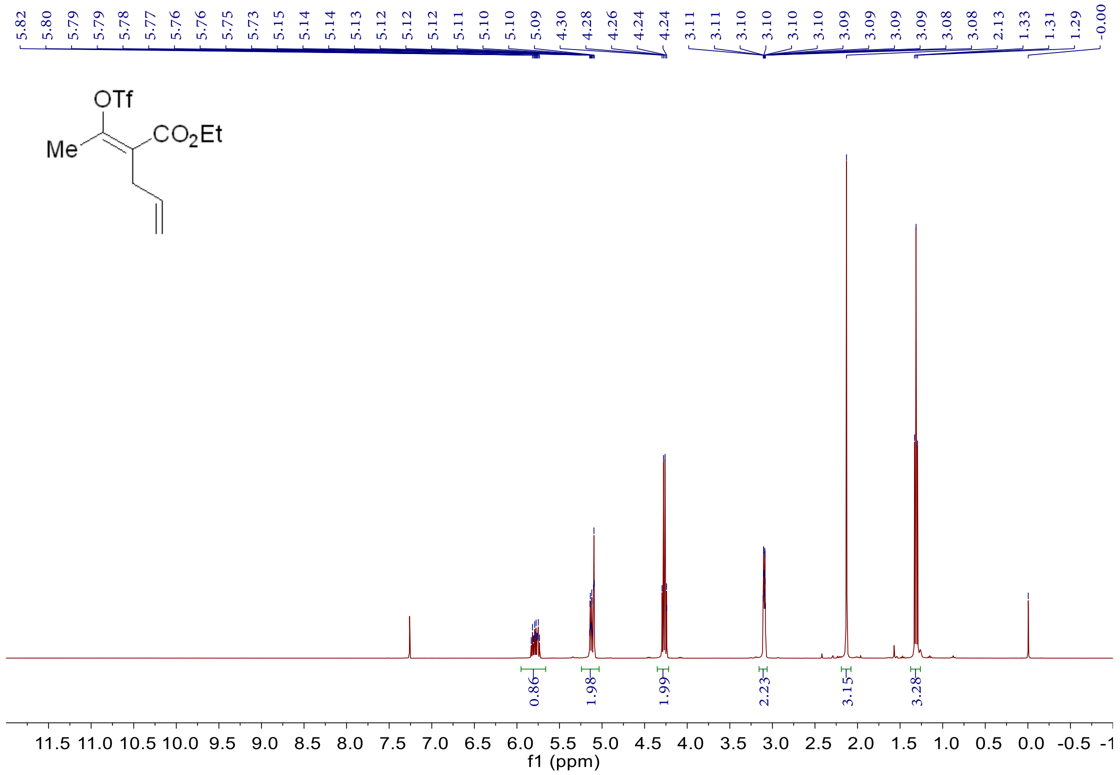

Supplementary Figure 56. ^{1}H NMR spectrum for compound **2n**

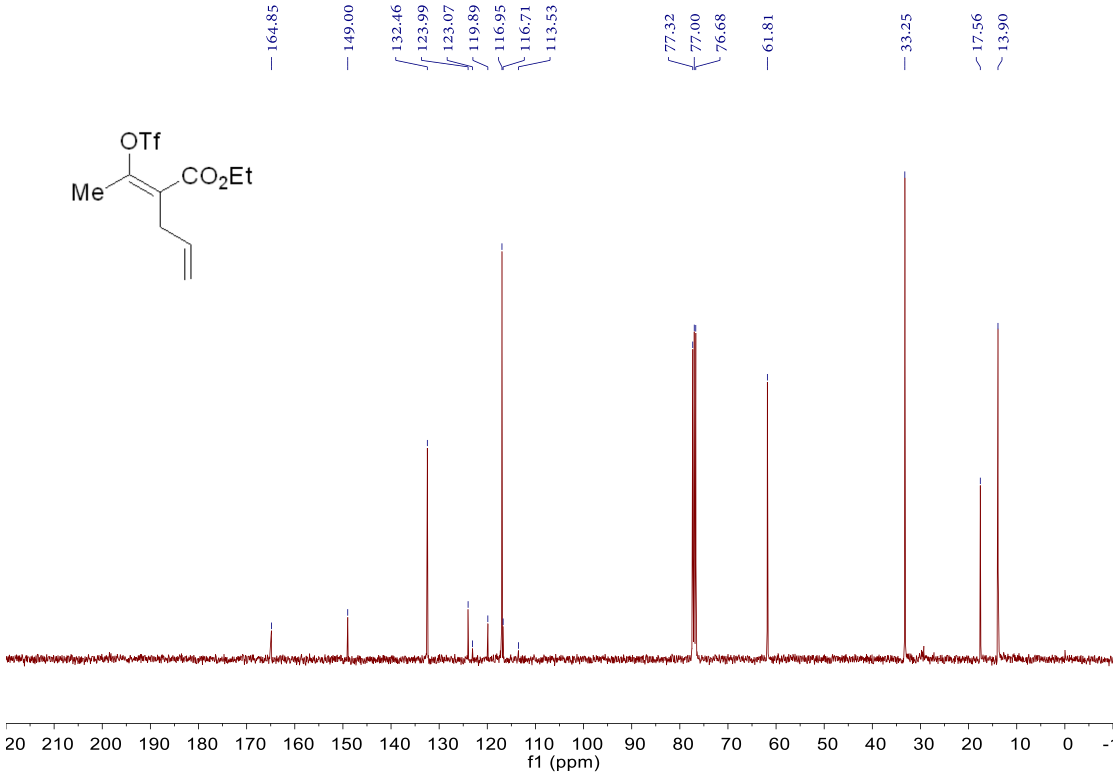

Supplementary Figure 57. ^{13}C NMR spectrum for compound **2n**

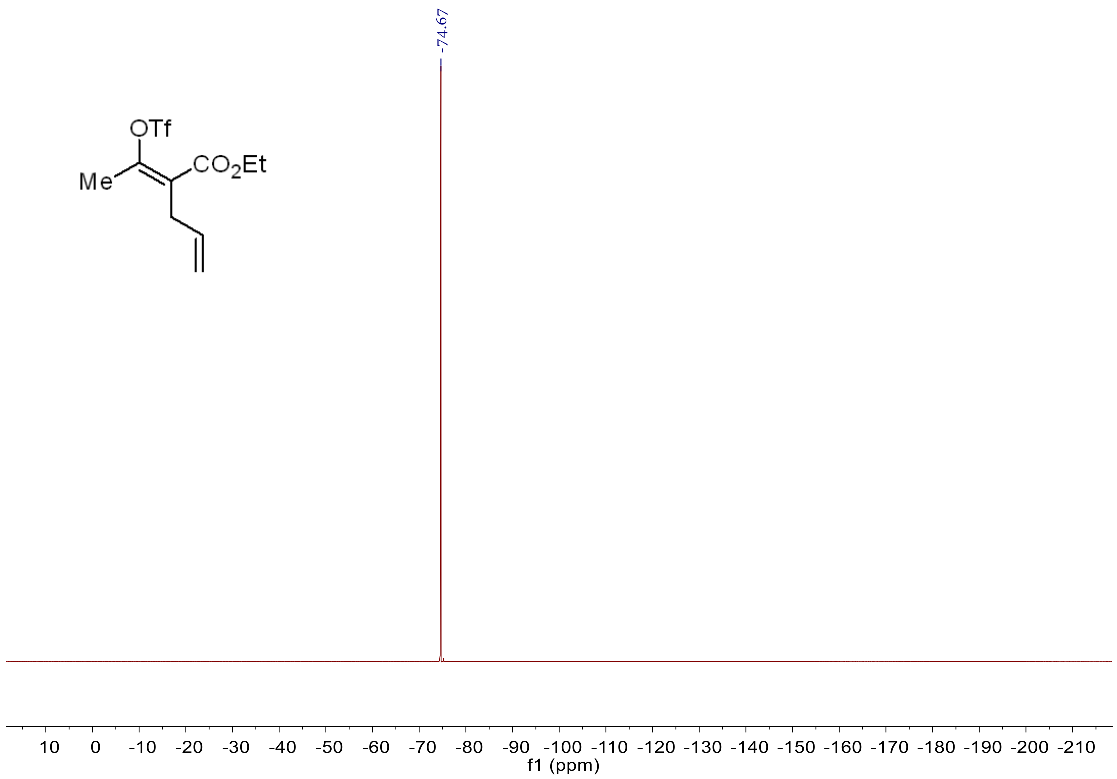

Supplementary Figure 58. ^{19}F NMR spectrum for compound **2n**

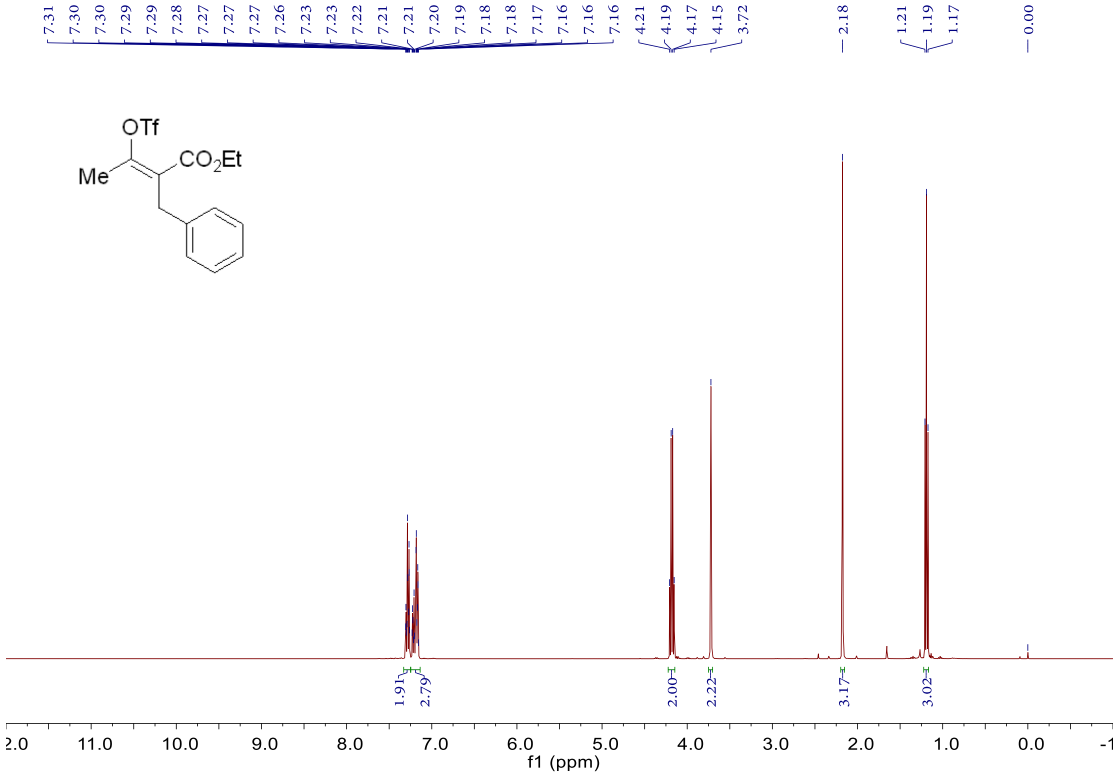

Supplementary Figure 59. ^{1}H NMR spectrum for compound **2o**

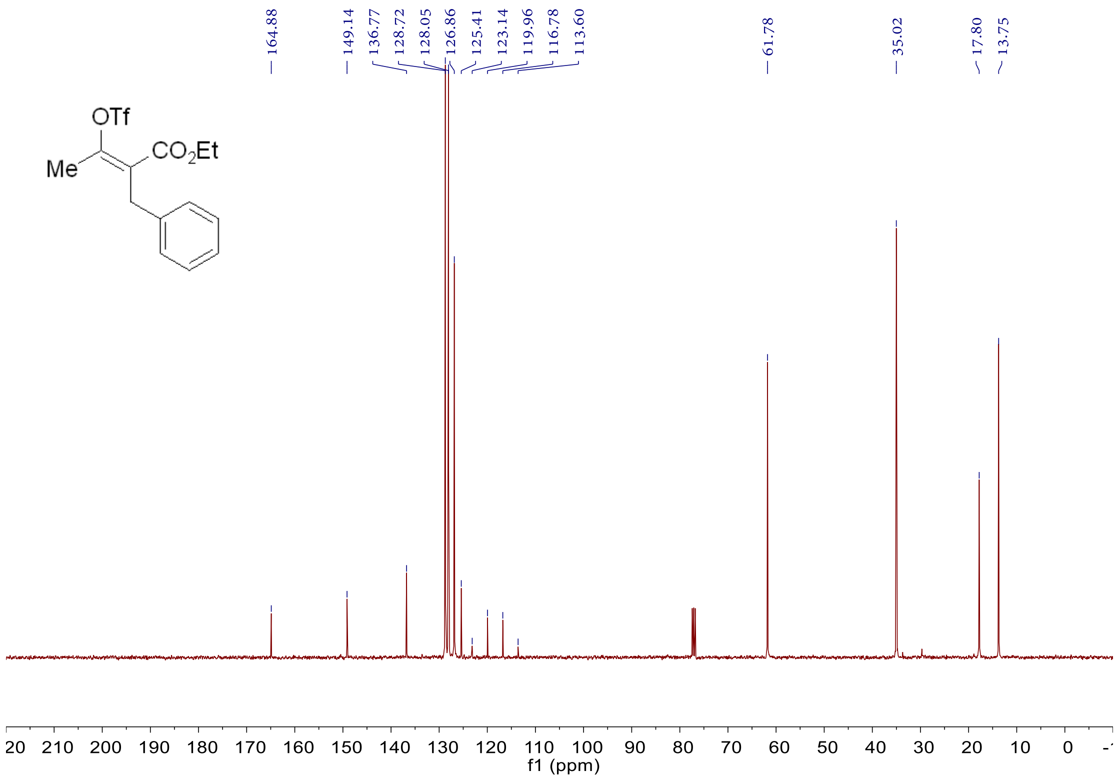

Supplementary Figure 60. ^{13}C NMR spectrum for compound **2o**

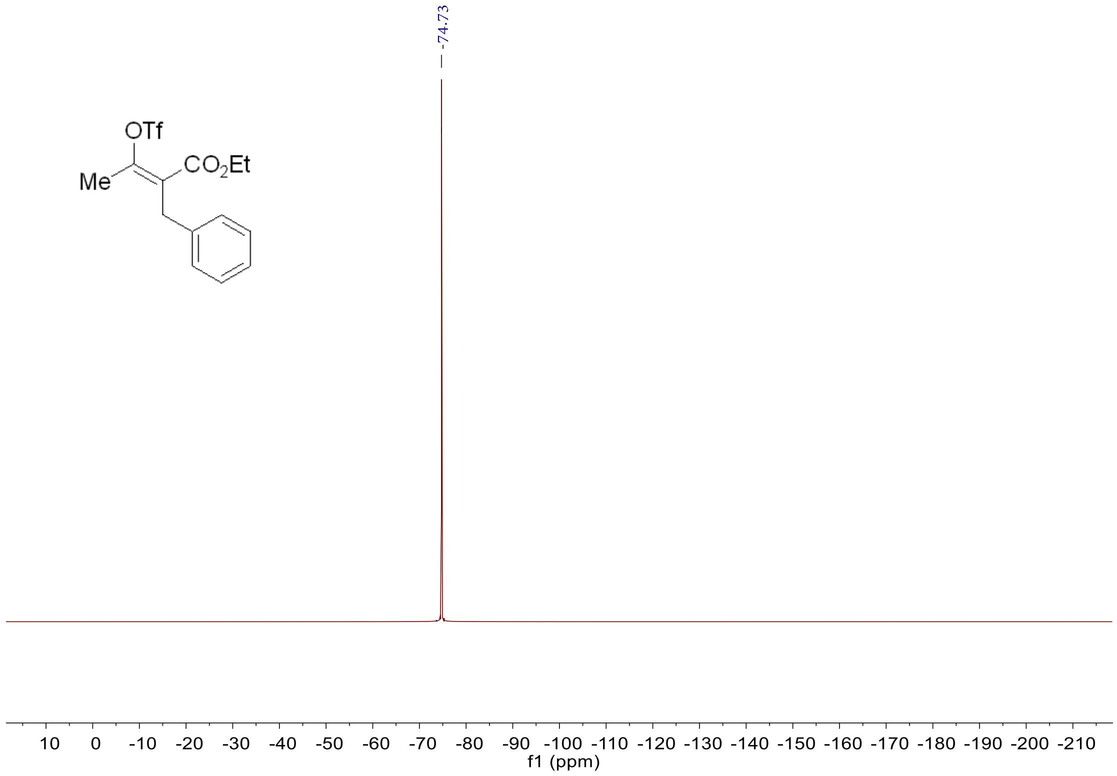

Supplementary Figure 61. ^{19}F NMR spectrum for compound **2o**

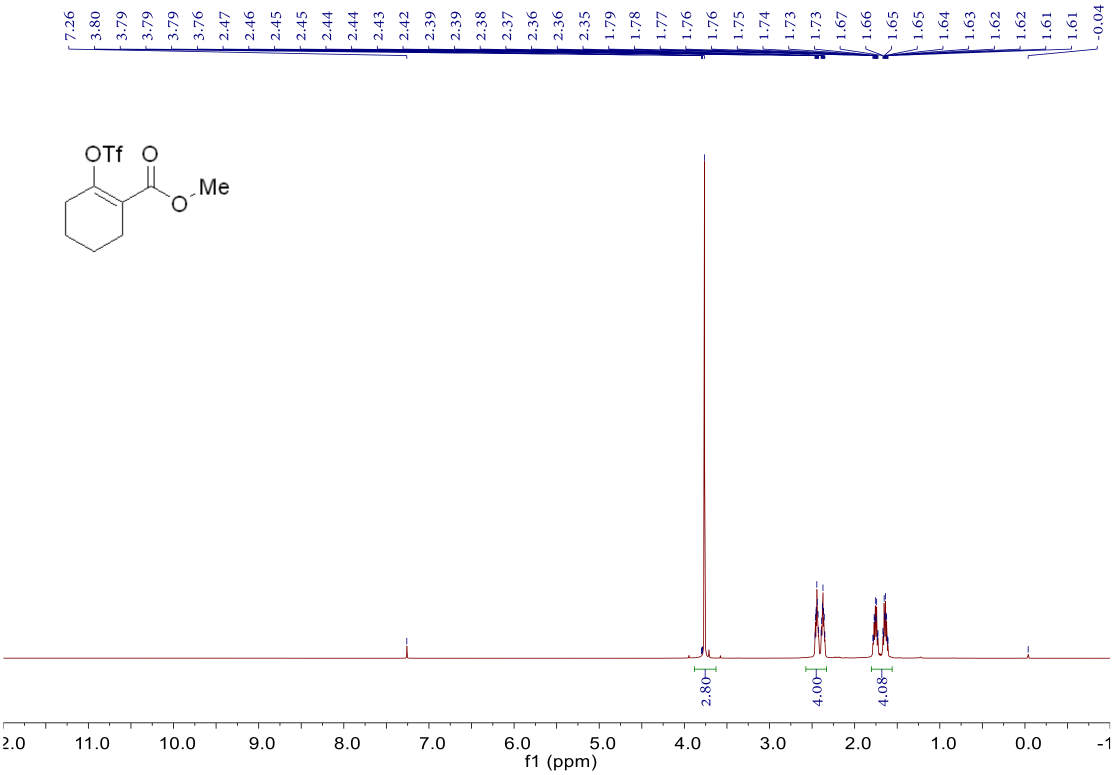

Supplementary Figure 62. ^{1}H NMR spectrum for compound **2p**

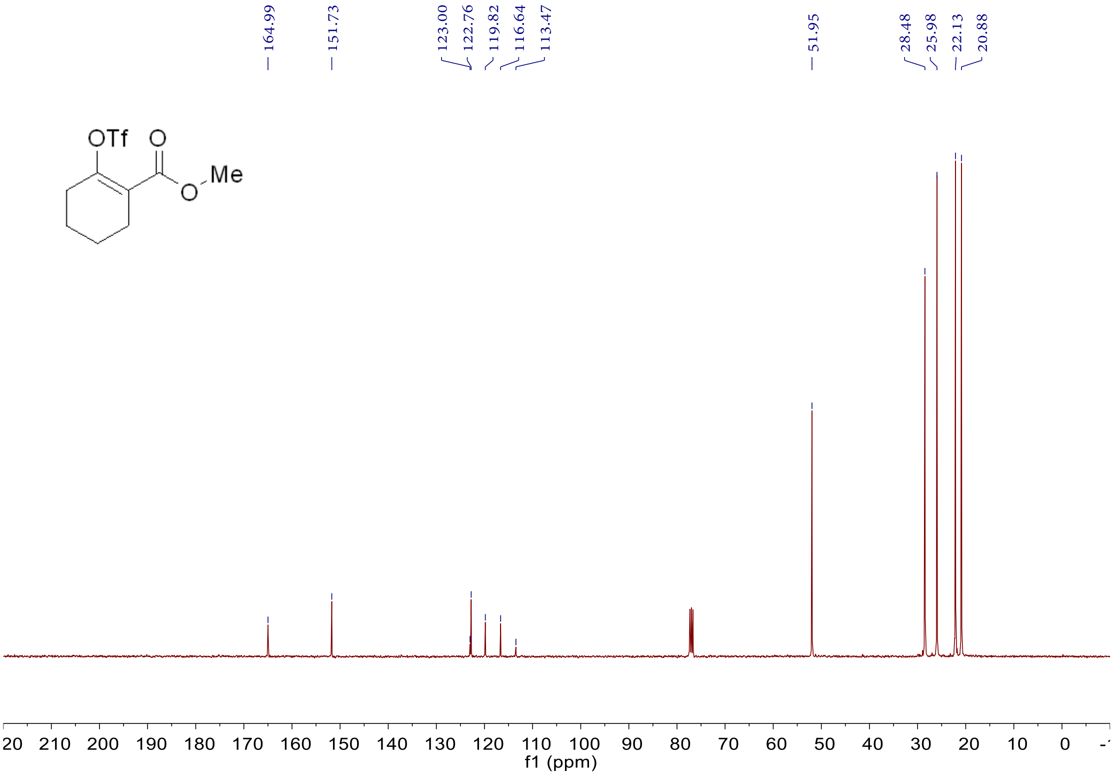

Supplementary Figure 63. ^{13}C NMR spectrum for compound **2p**

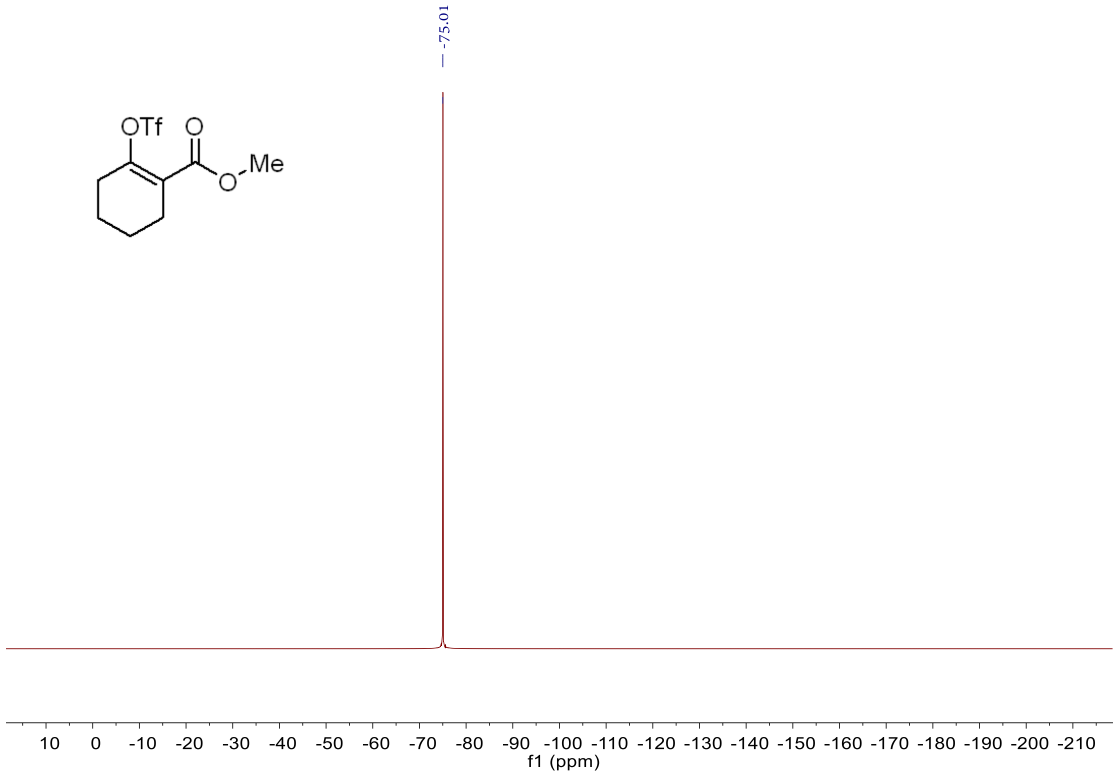

Supplementary Figure 64. ^{19}F NMR spectrum for compound **2p**

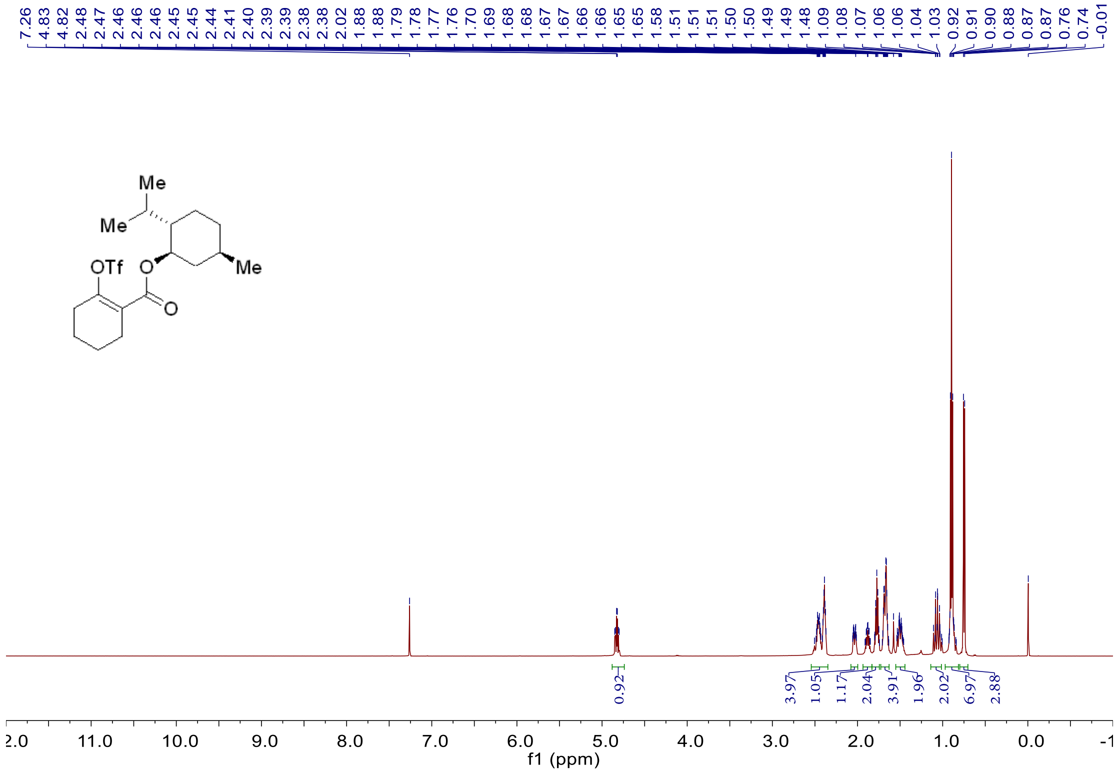

Supplementary Figure 65. ^{1}H NMR spectrum for compound **2q**

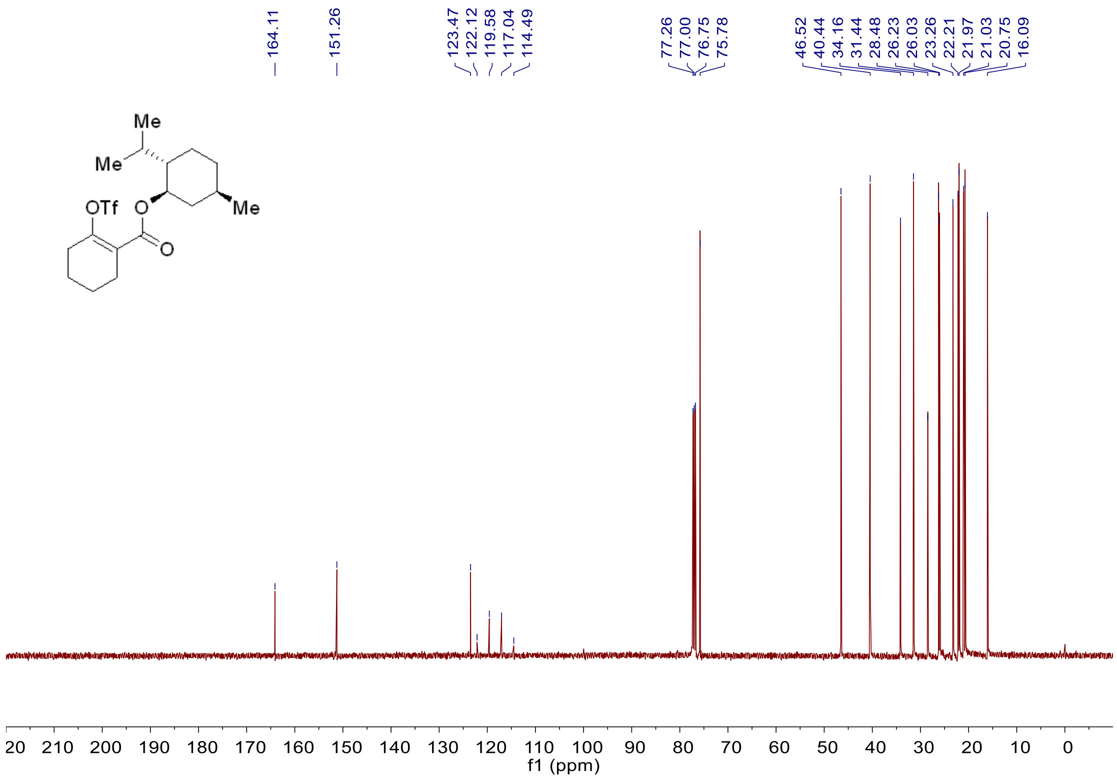

Supplementary Figure 66. ^{13}C NMR spectrum for compound **2q**

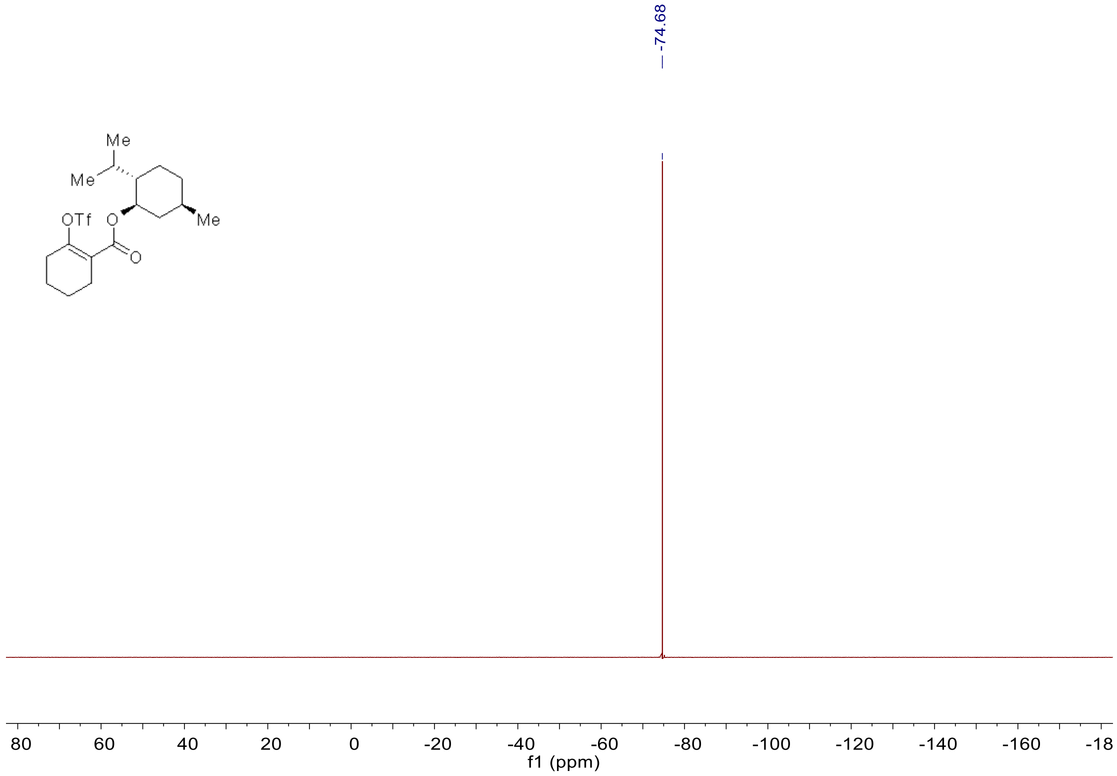

Supplementary Figure 67. ^{19}F NMR spectrum for compound **2q**

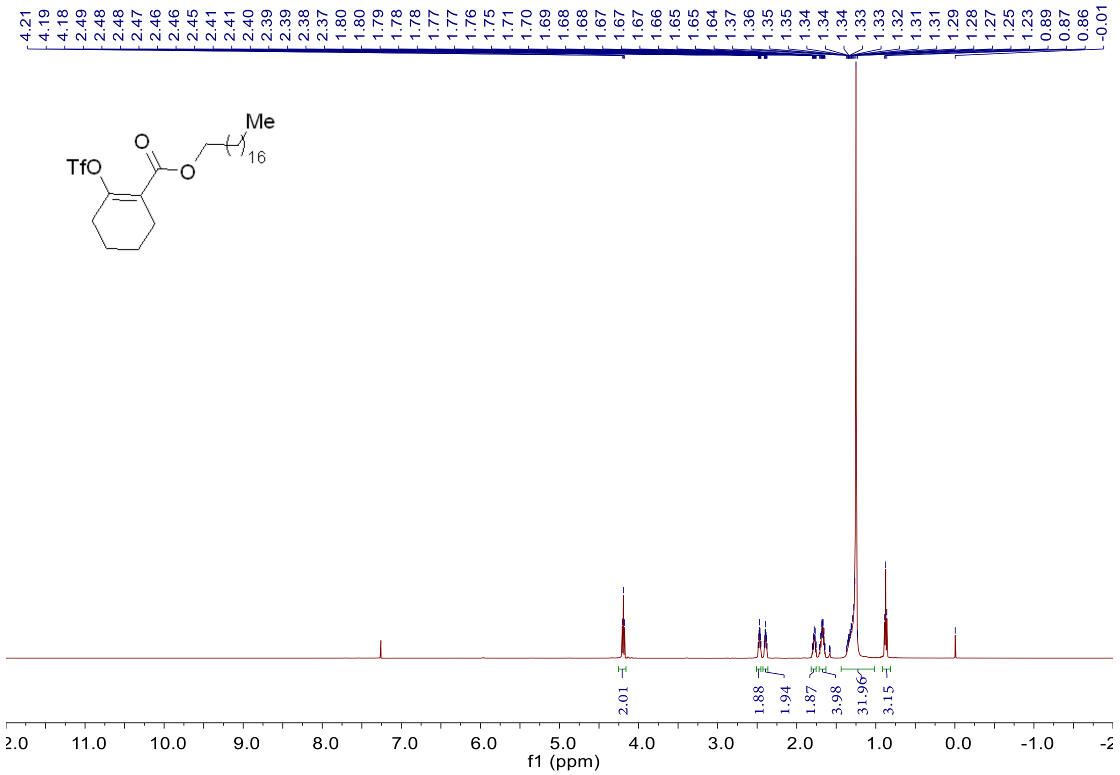

Supplementary Figure 68. ^{1}H NMR spectrum for compound **2r**

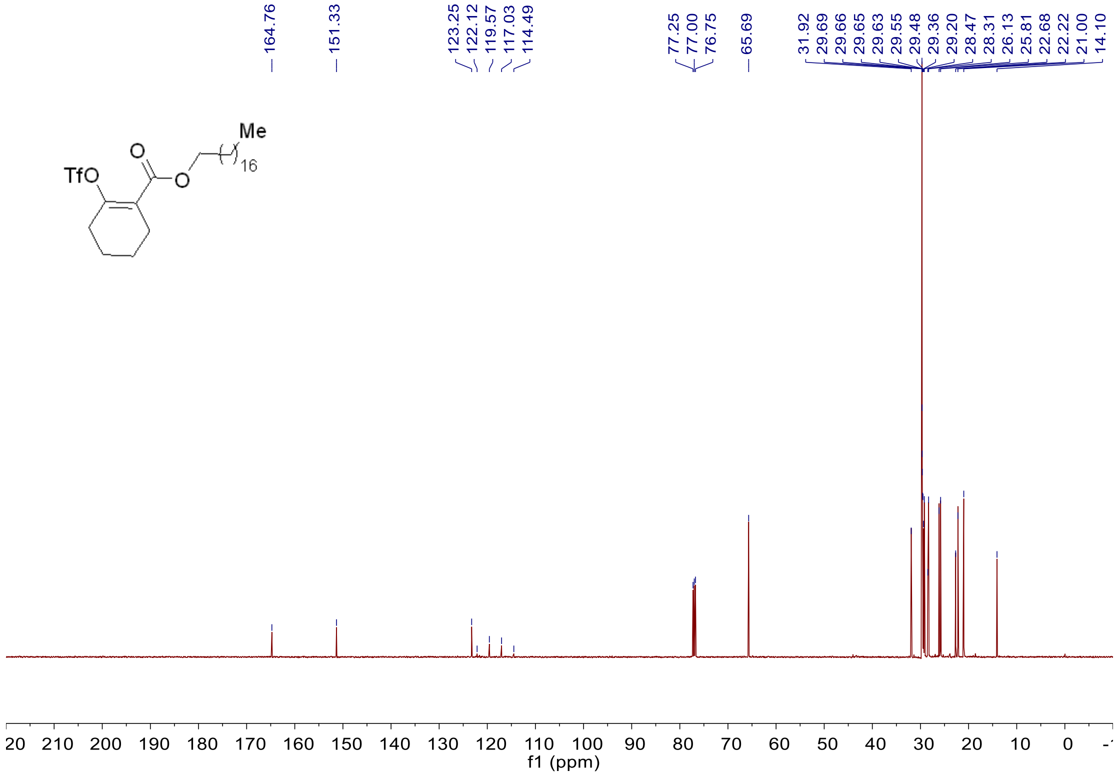

Supplementary Figure 69. ^{13}C NMR spectrum for compound **2r**

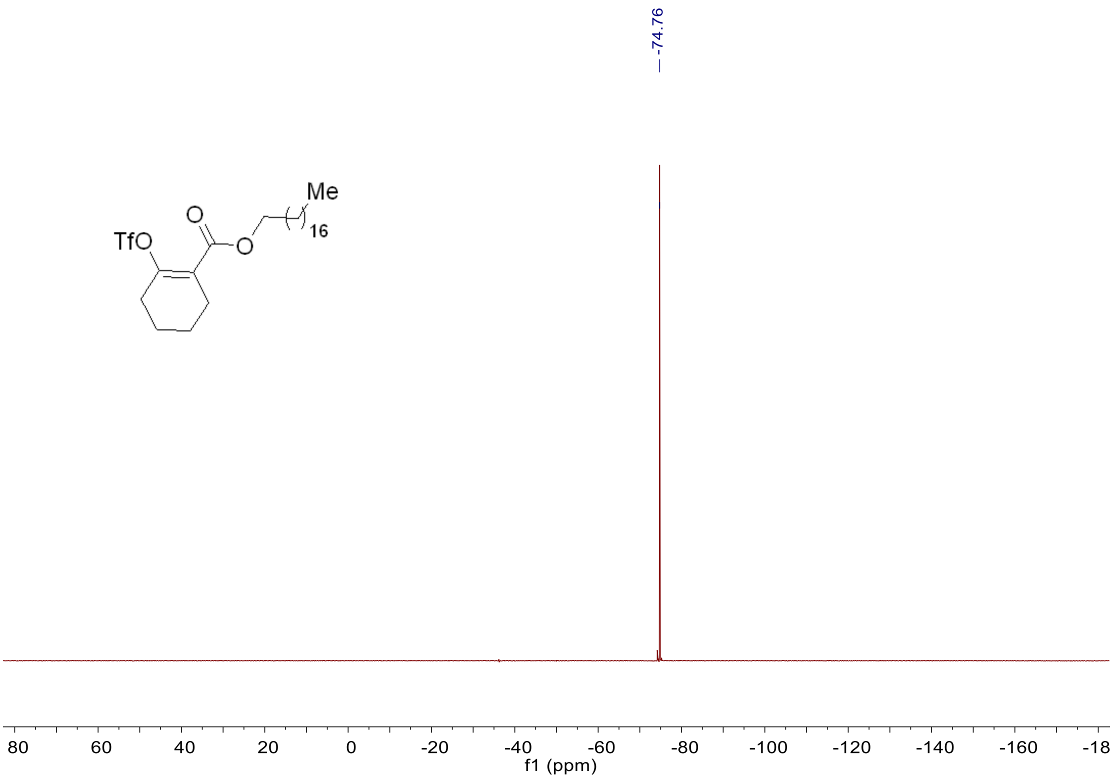

Supplementary Figure 70. ^{19}F NMR spectrum for compound **2r**

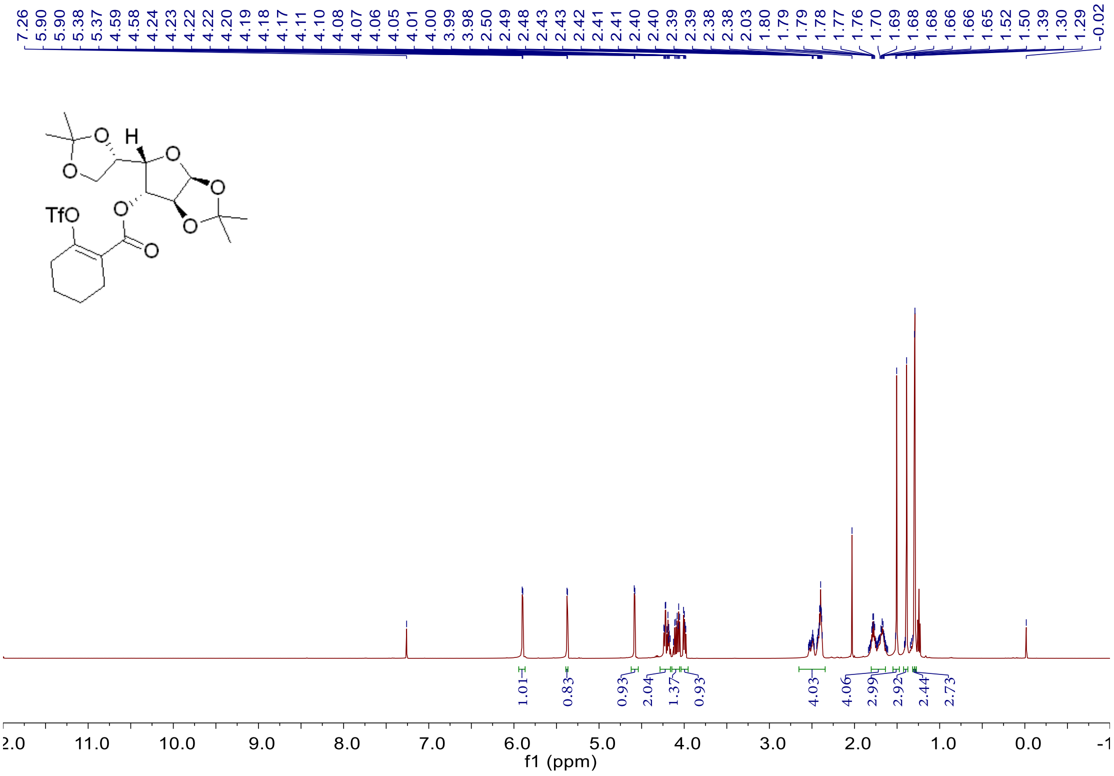

Supplementary Figure 71. ^{1}H NMR spectrum for compound **2s**

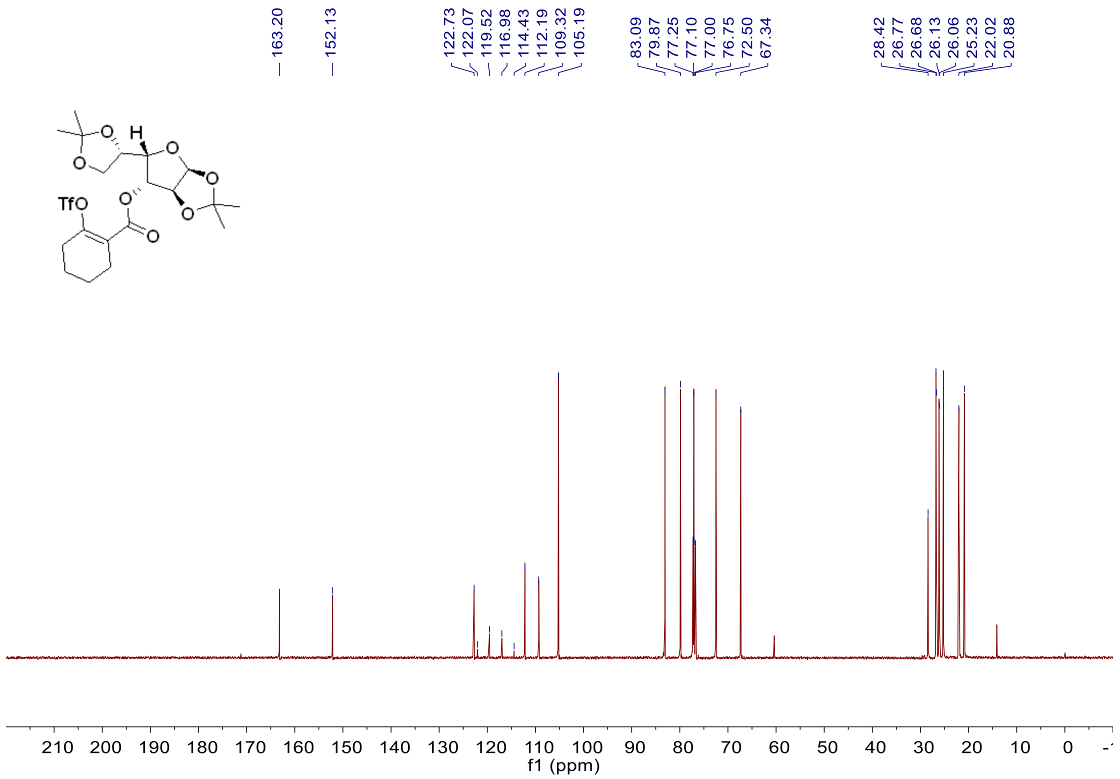

Supplementary Figure 72. ^{13}C NMR spectrum for compound **2s**

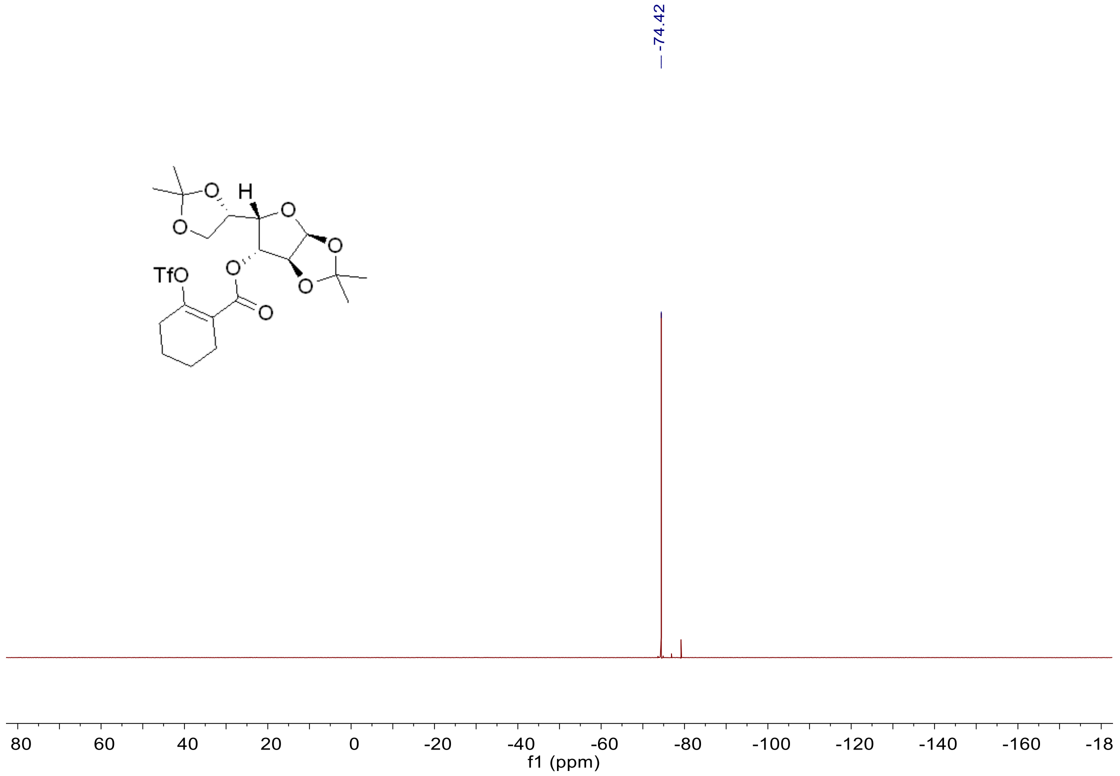

Supplementary Figure 73. ^{19}F NMR spectrum for compound **2s**

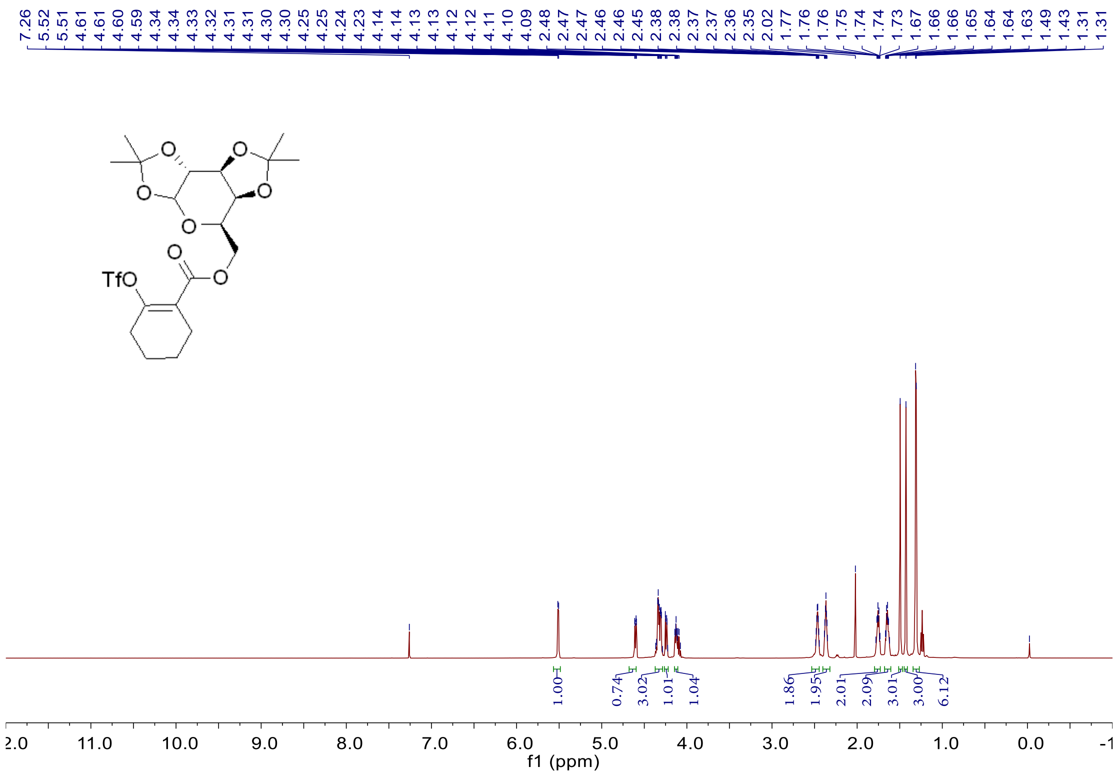

Supplementary Figure 74. ^{1}H NMR spectrum for compound **2t**

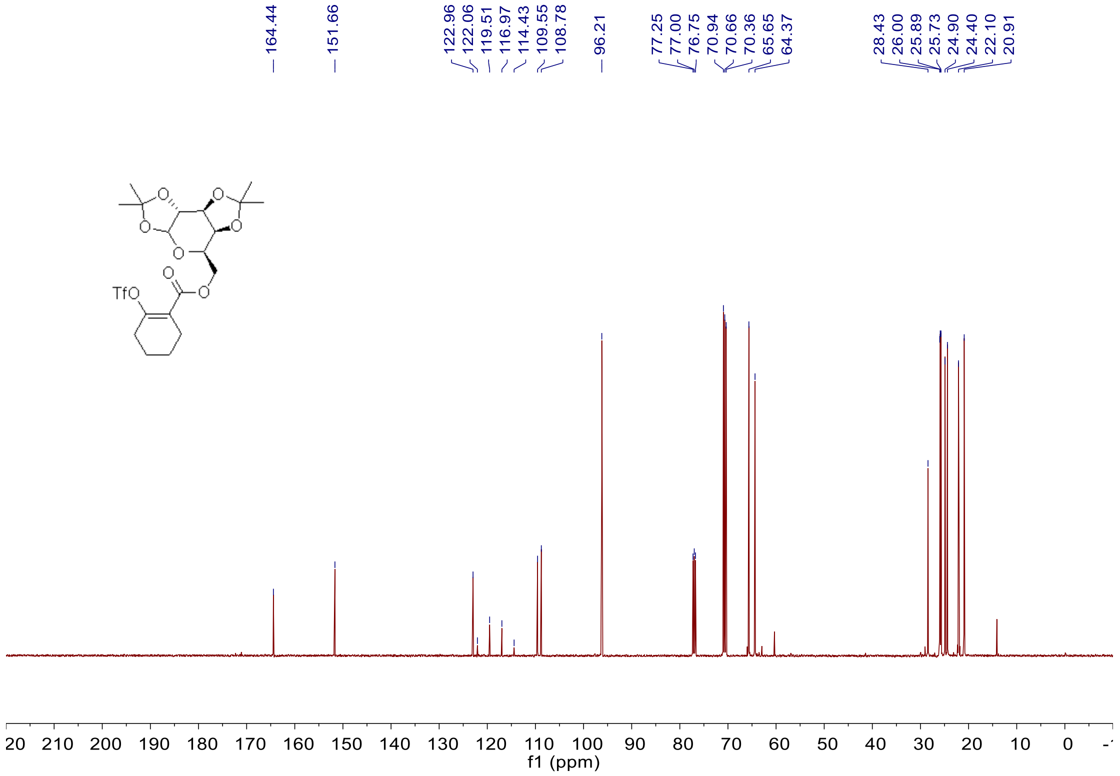

Supplementary Figure 75. ^{13}C NMR spectrum for compound **2t**

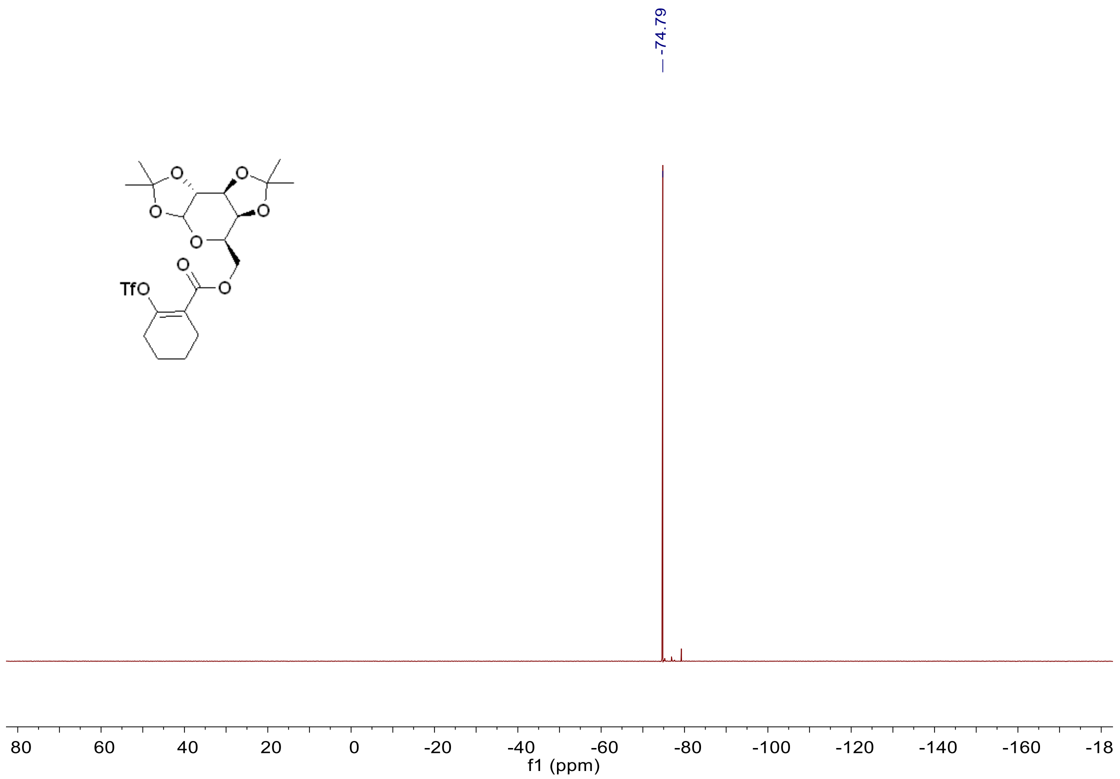

Supplementary Figure 76. ^{19}F NMR spectrum for compound **2t**

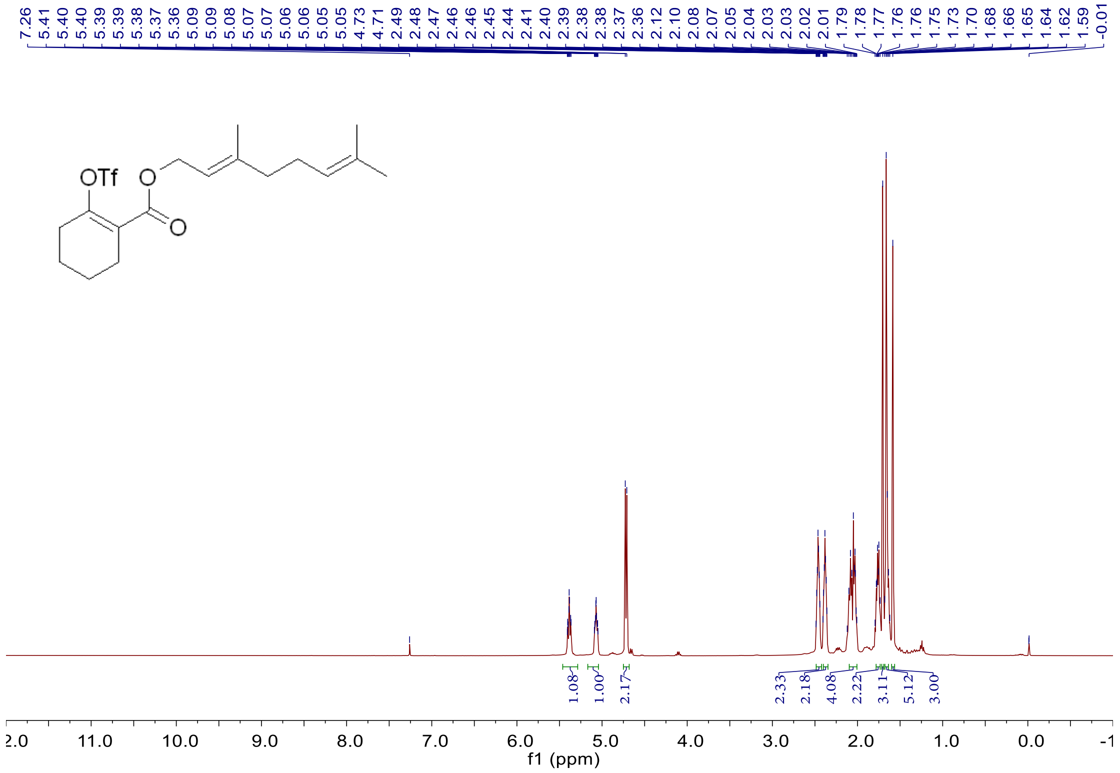

Supplementary Figure 77. ^{1}H NMR spectrum for compound **2u**

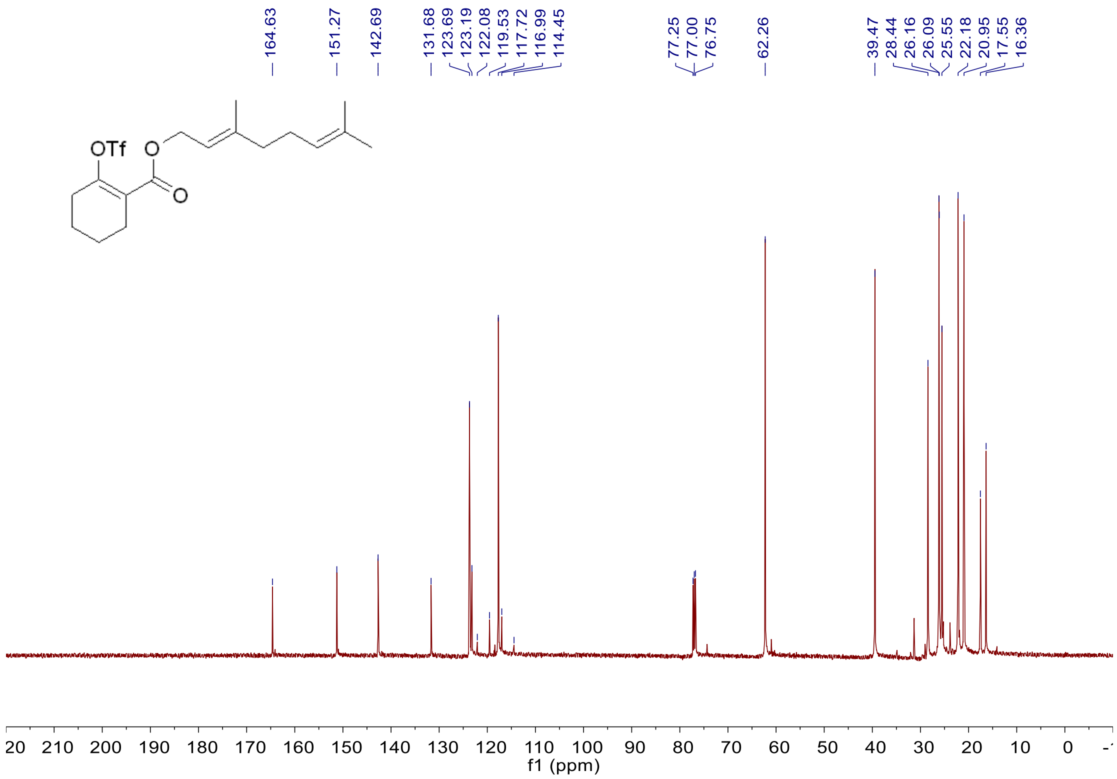

Supplementary Figure 78. ^{13}C NMR spectrum for compound **2u**

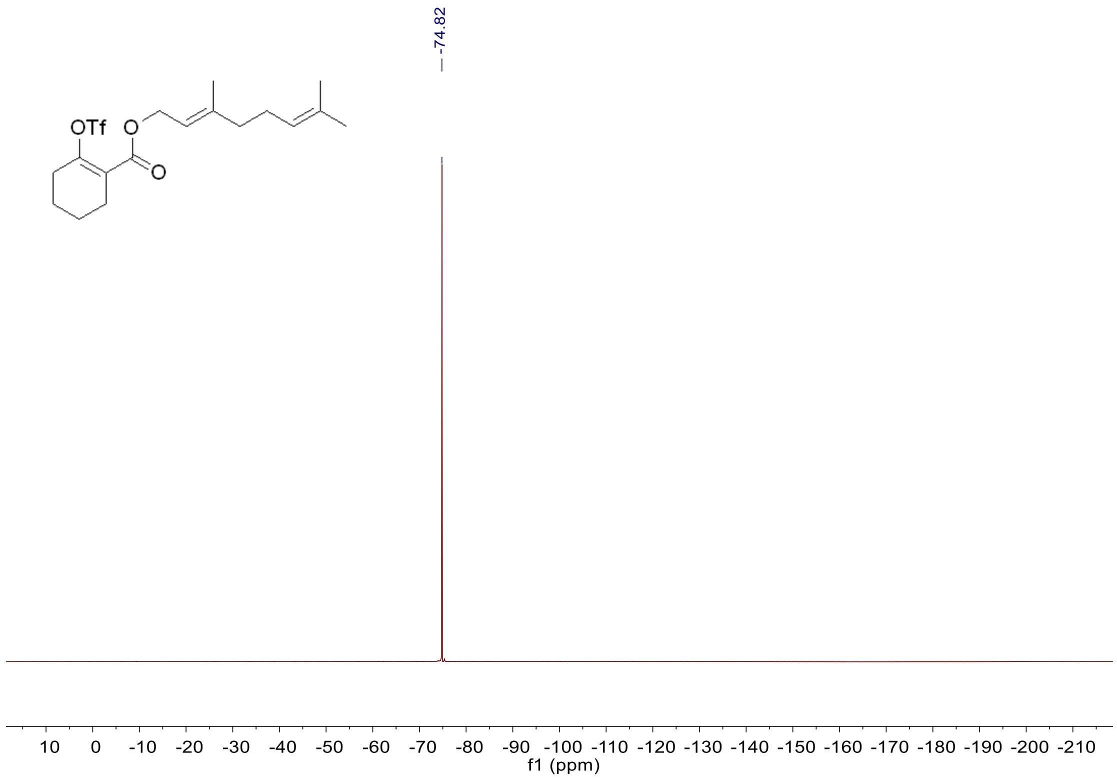

Supplementary Figure 79. ^{19}F NMR spectrum for compound **2u**

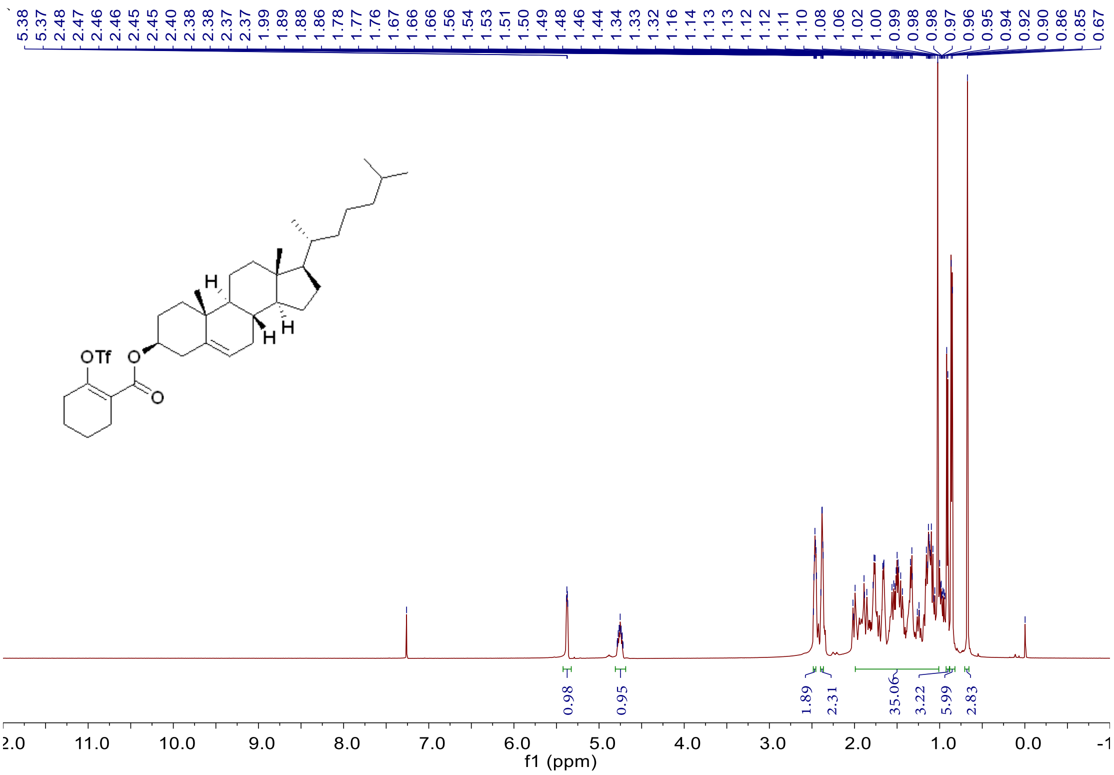

Supplementary Figure 80. ^{1}H NMR spectrum for compound **2v**

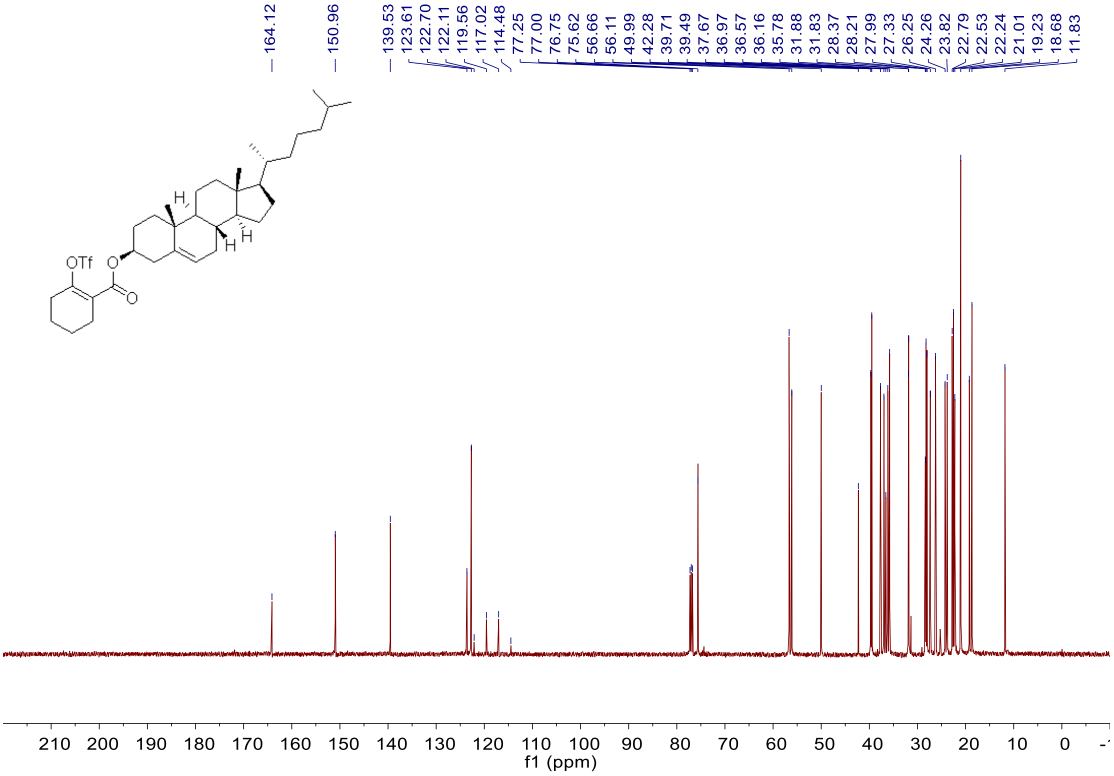

Supplementary Figure 81. ^{13}C NMR spectrum for compound **2v**

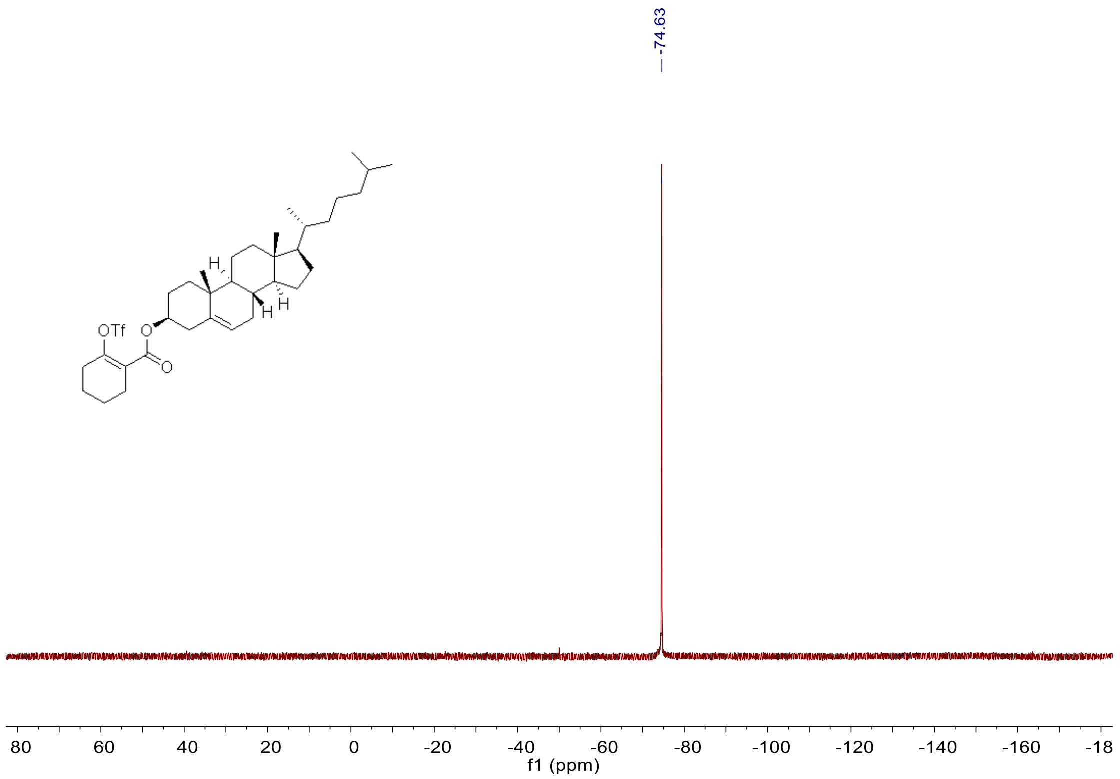

Supplementary Figure 82. ^{19}F NMR spectrum for compound **2v**

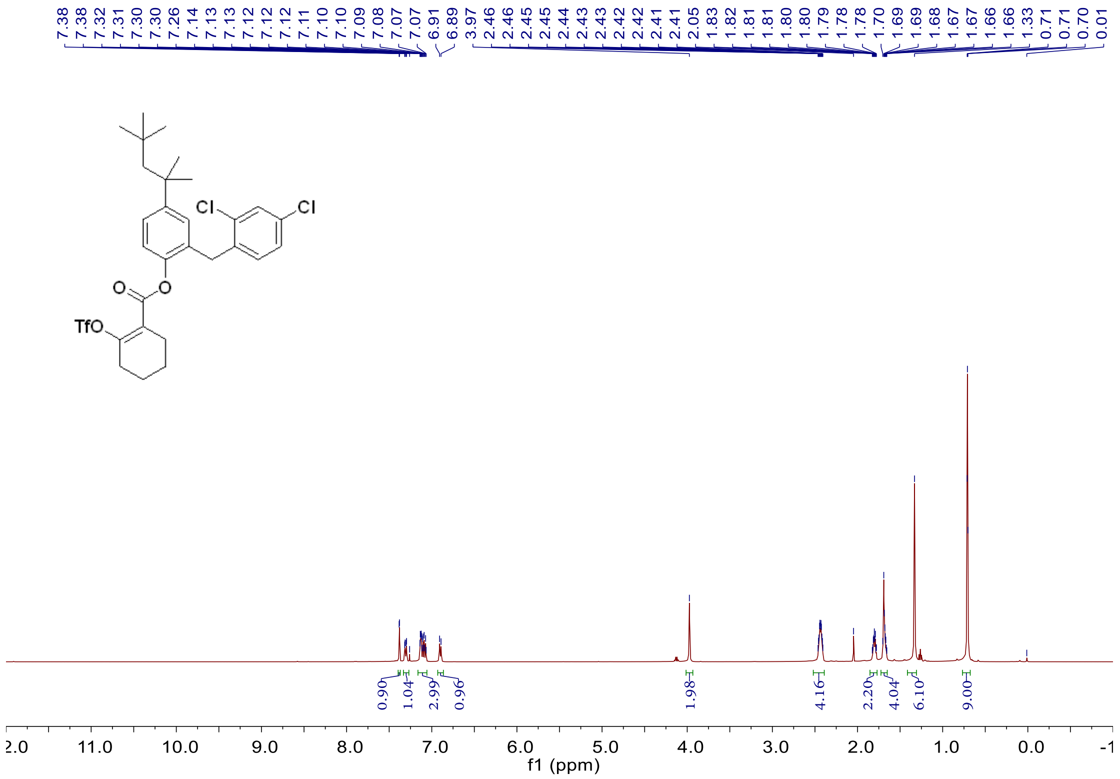

Supplementary Figure 83. ^{1}H NMR spectrum for compound **2w**

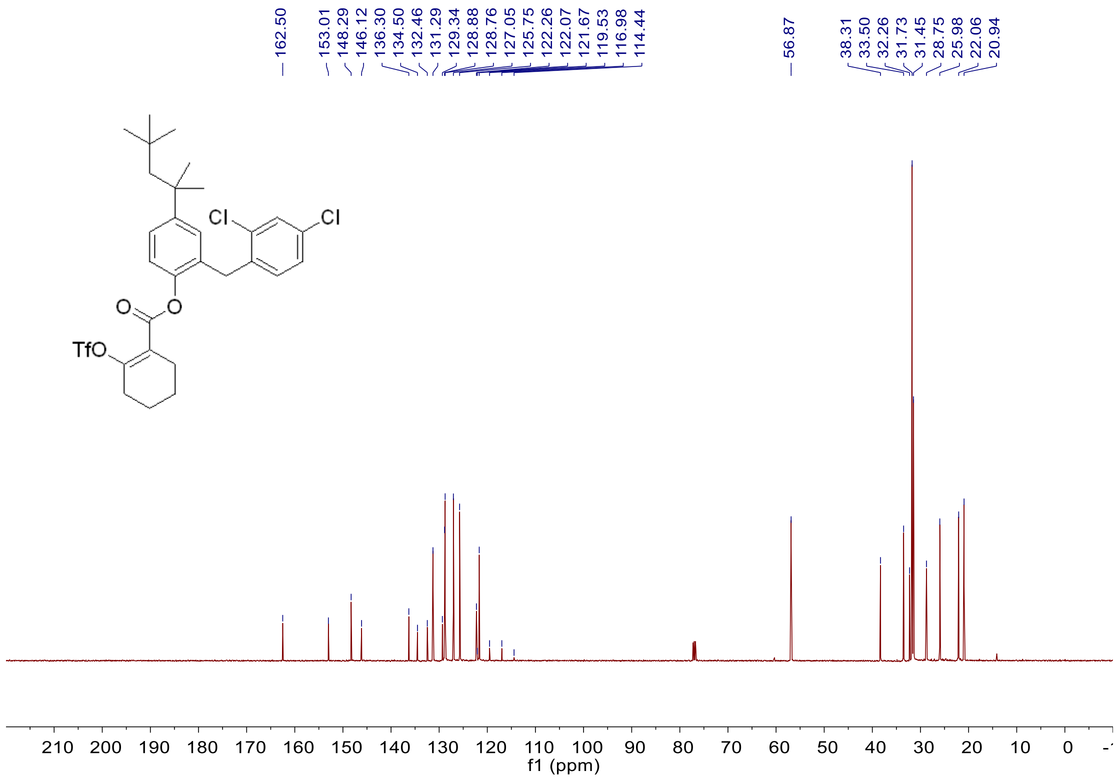

Supplementary Figure 84. ^{13}C NMR spectrum for compound **2w**

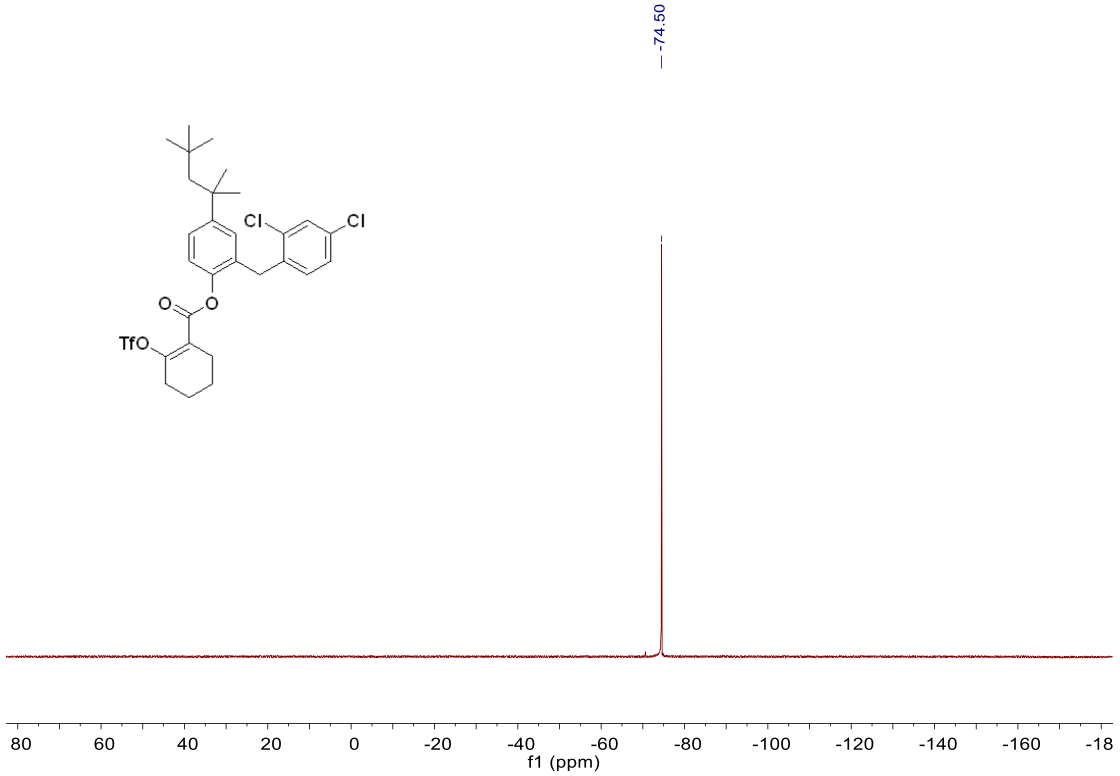

Supplementary Figure 85. ^{19}F NMR spectrum for compound **2w**

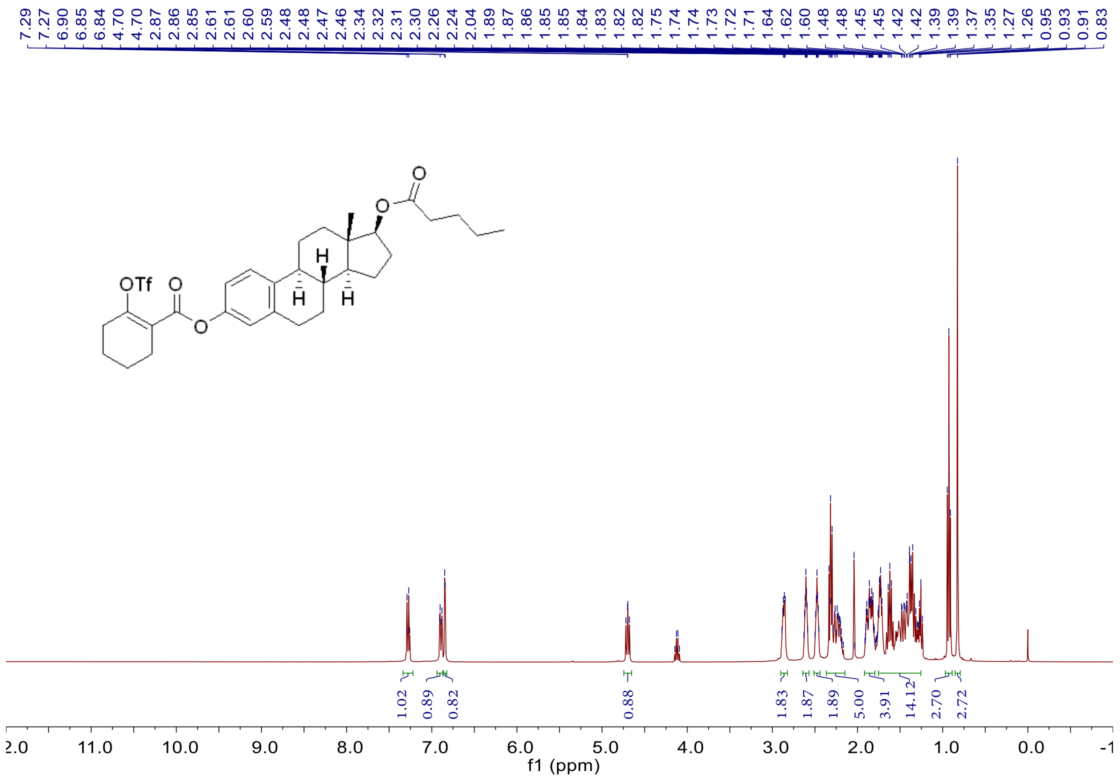

Supplementary Figure 86. ^{1}H NMR spectrum for compound **2x**

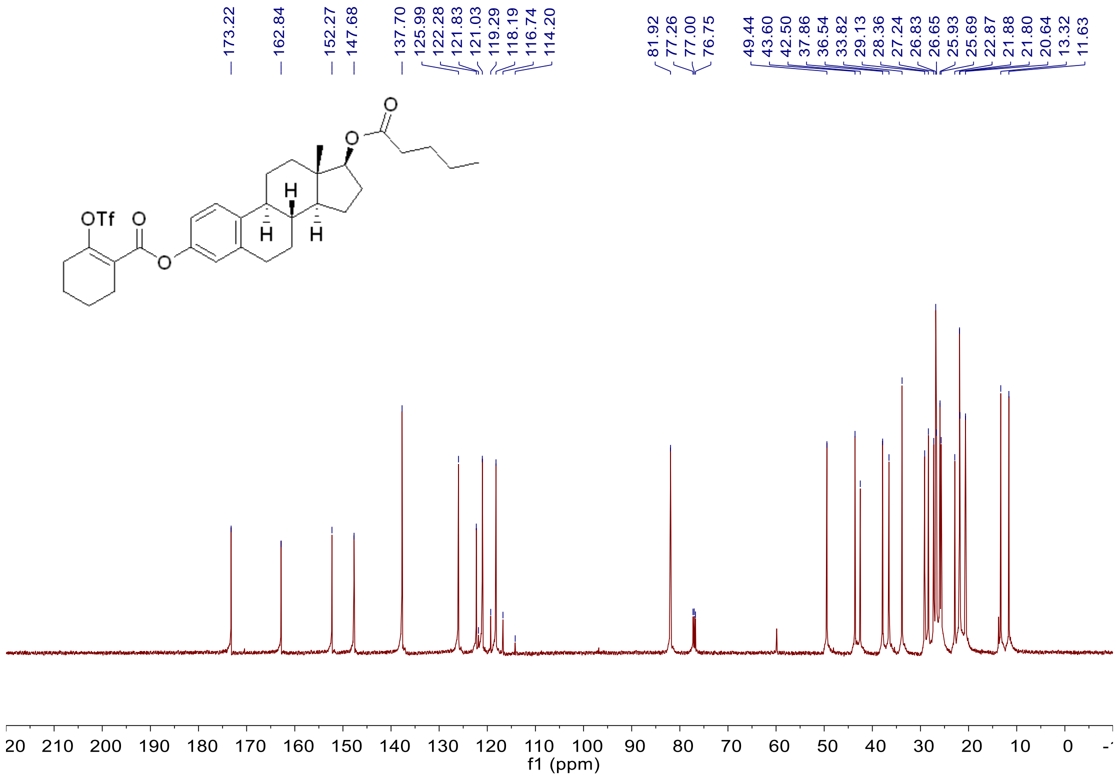

Supplementary Figure 87. ^{13}C NMR spectrum for compound **2x**

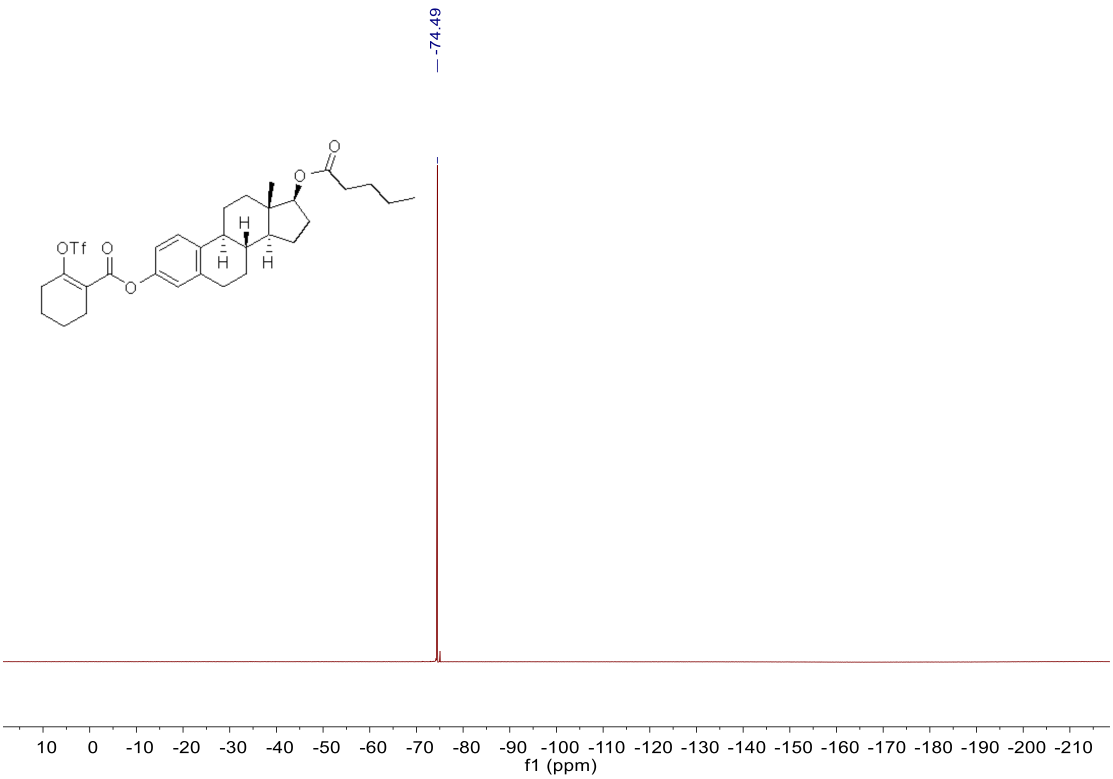

Supplementary Figure 88. ^{19}F NMR spectrum for compound **2x**

2.8.2. NMR spectra of products

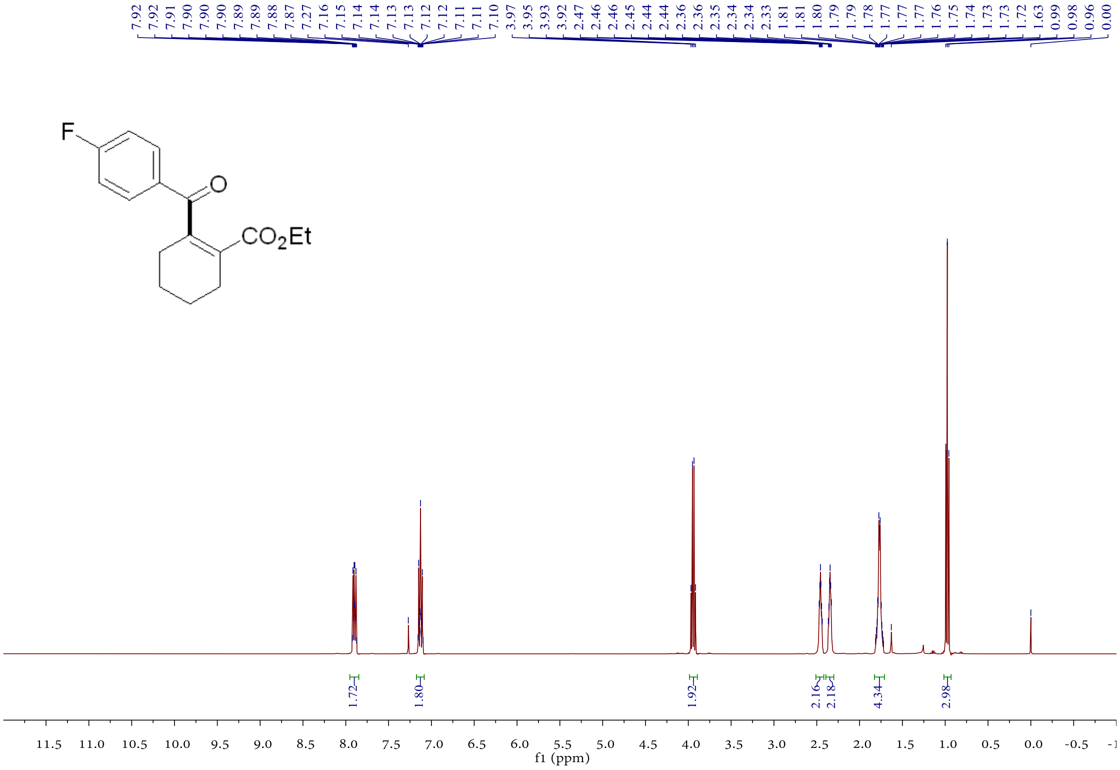

Supplementary Figure 89. ^{1}H NMR spectrum for compound **3a**

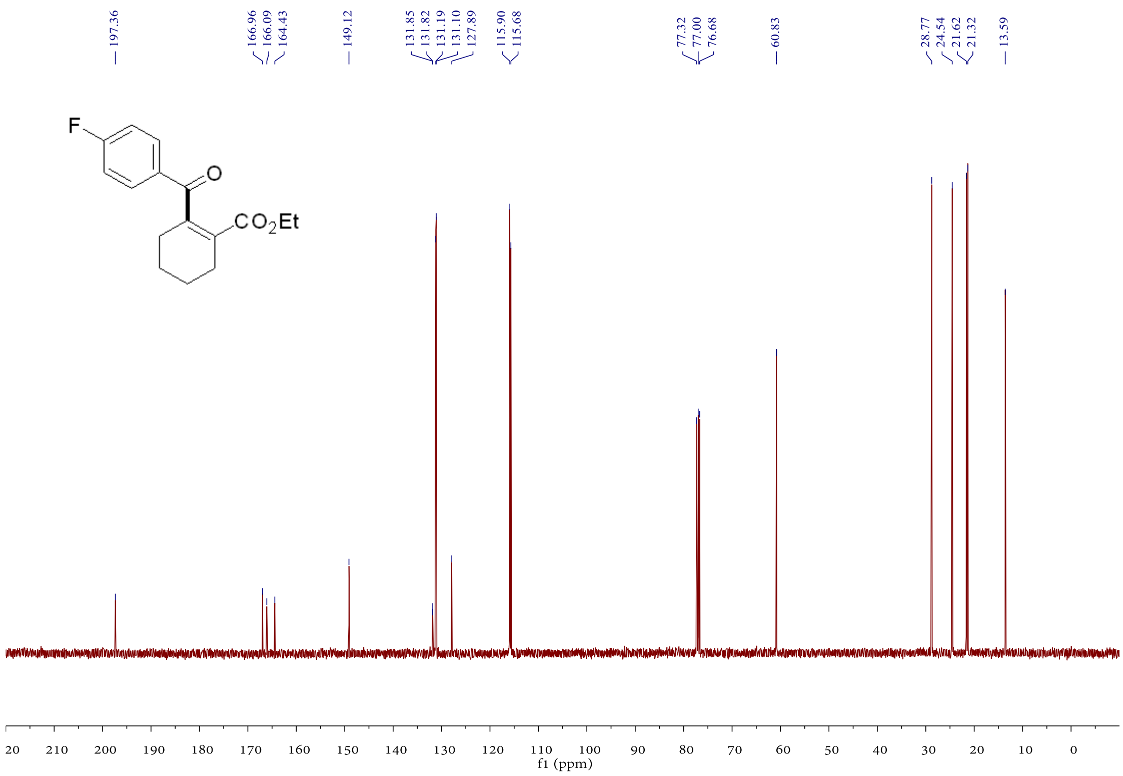

Supplementary Figure 90. ^{13}C NMR spectrum for compound **3a**

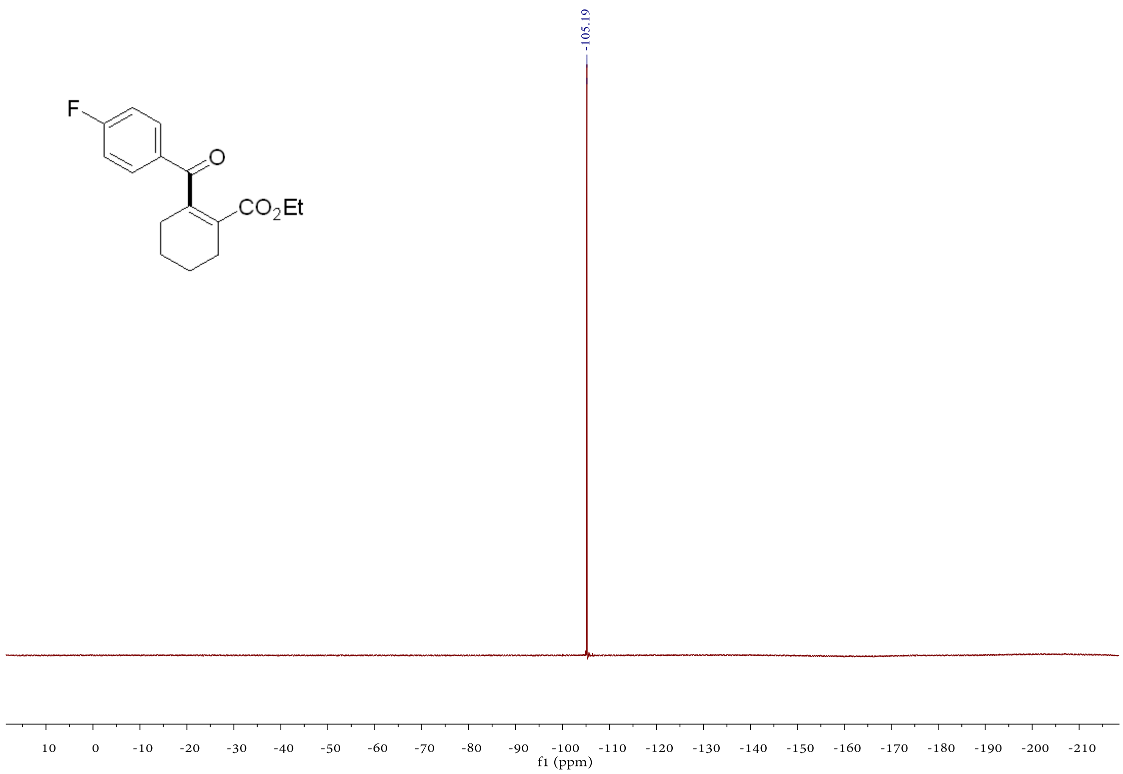

Supplementary Figure 91. ^{19}F NMR spectrum for compound **3a**

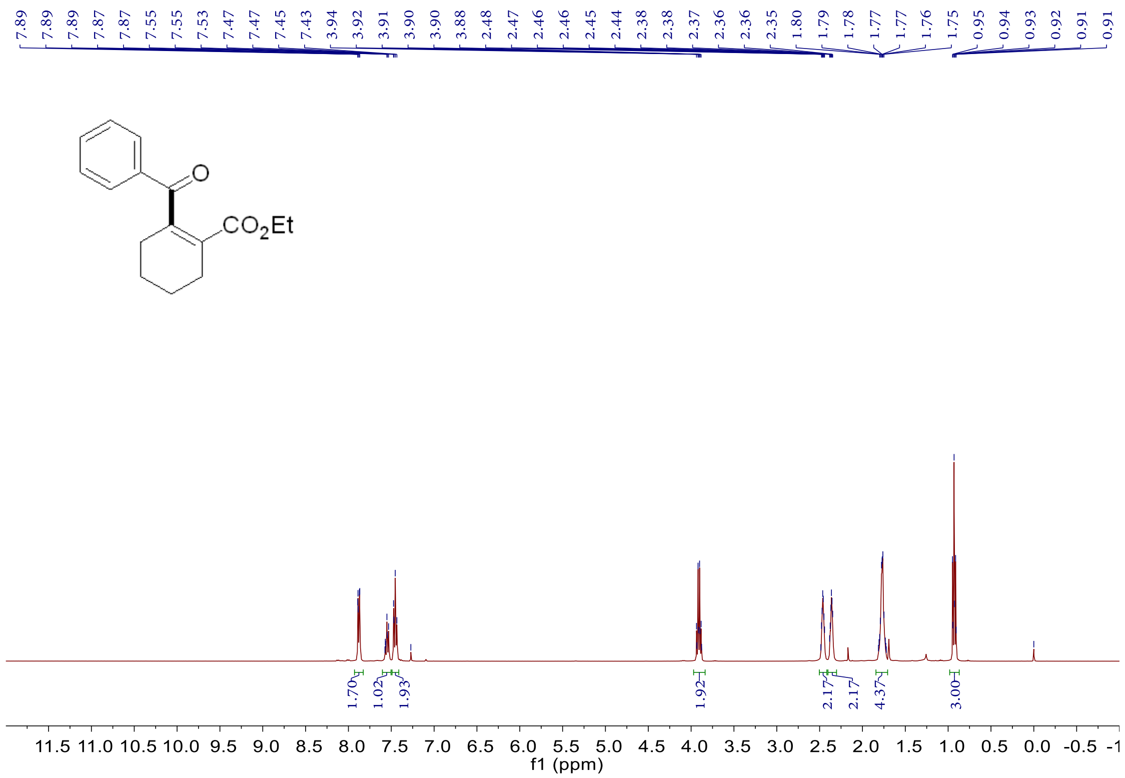

Supplementary Figure 92. ^{1}H NMR spectrum for compound **3b**

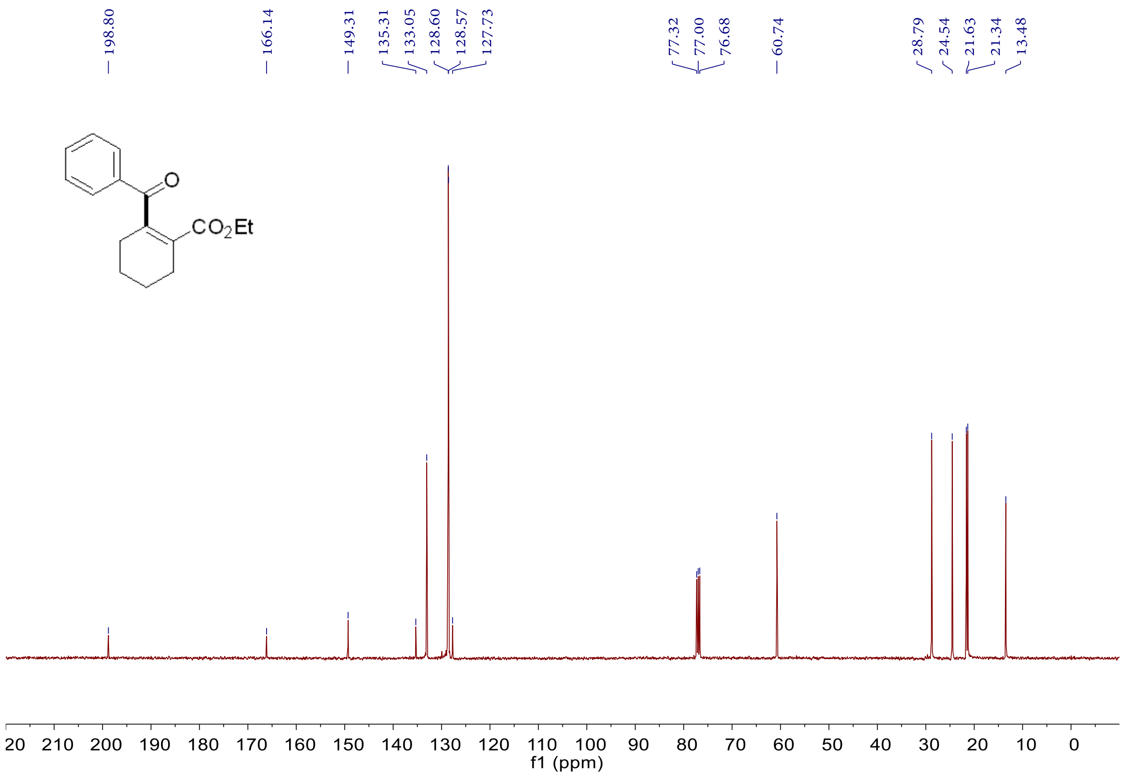

Supplementary Figure 93. ^{13}C NMR spectrum for compound **3b**

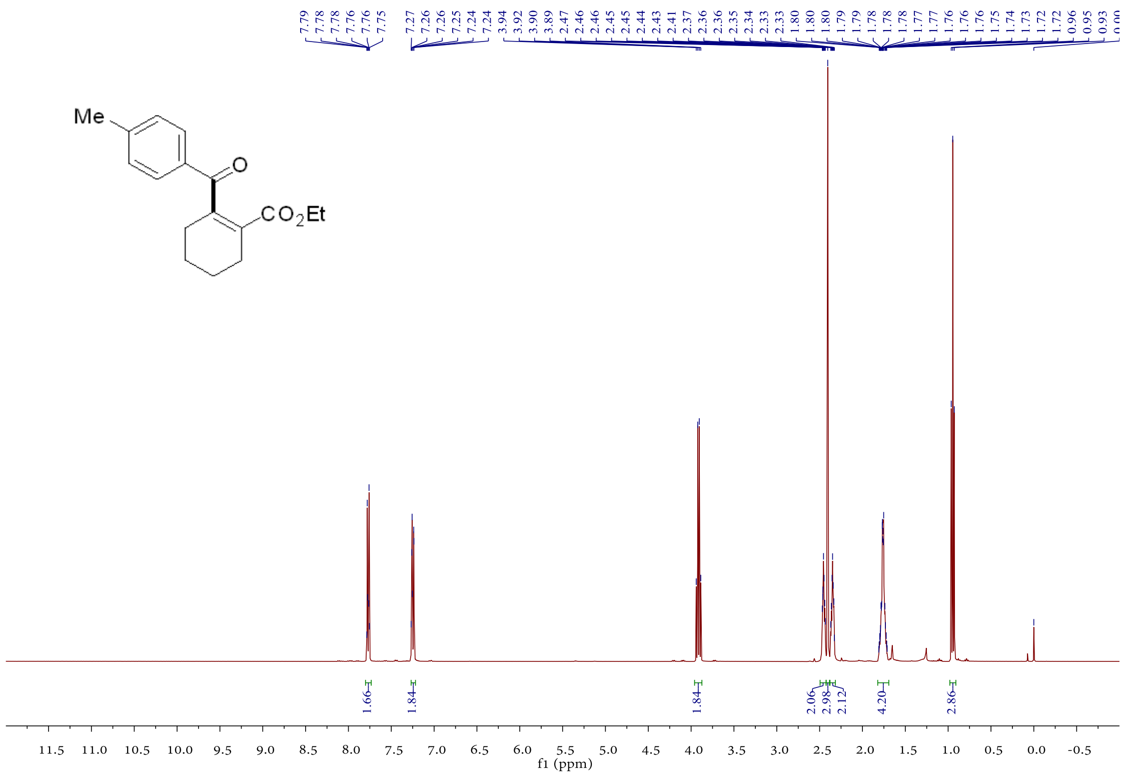

Supplementary Figure 94. ^{1}H NMR spectrum for compound **3c**

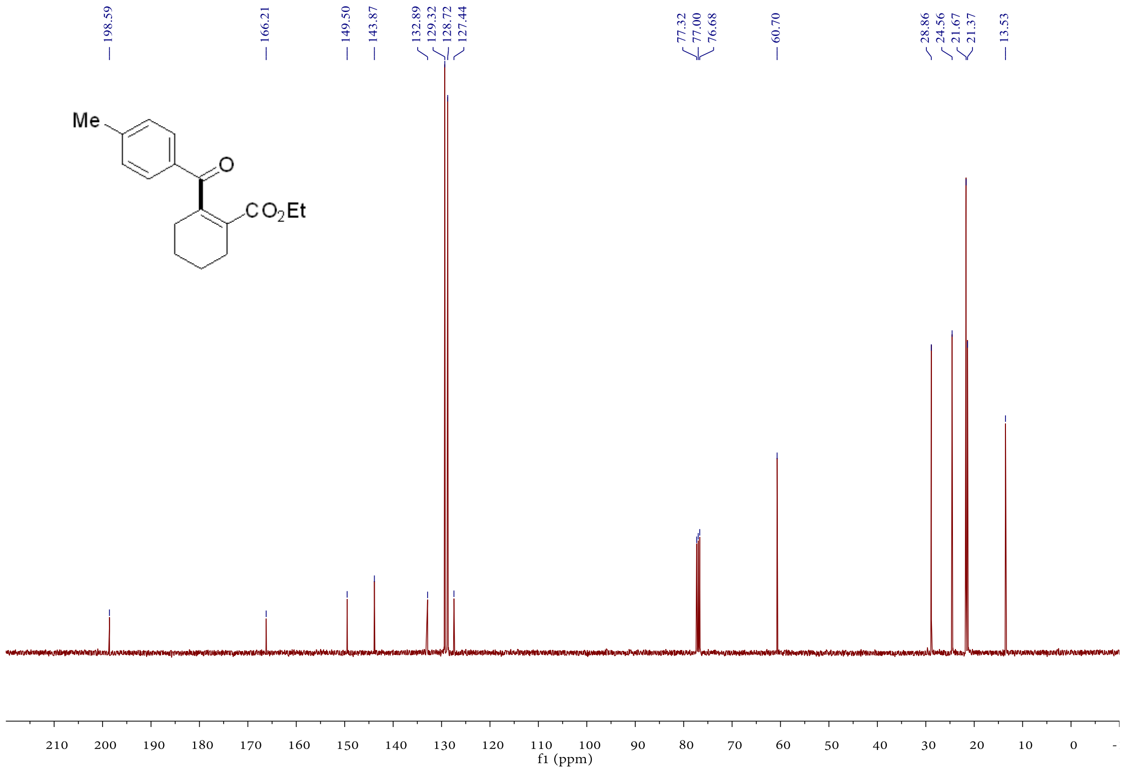

Supplementary Figure 95. ^{13}C NMR spectrum for compound **3c**

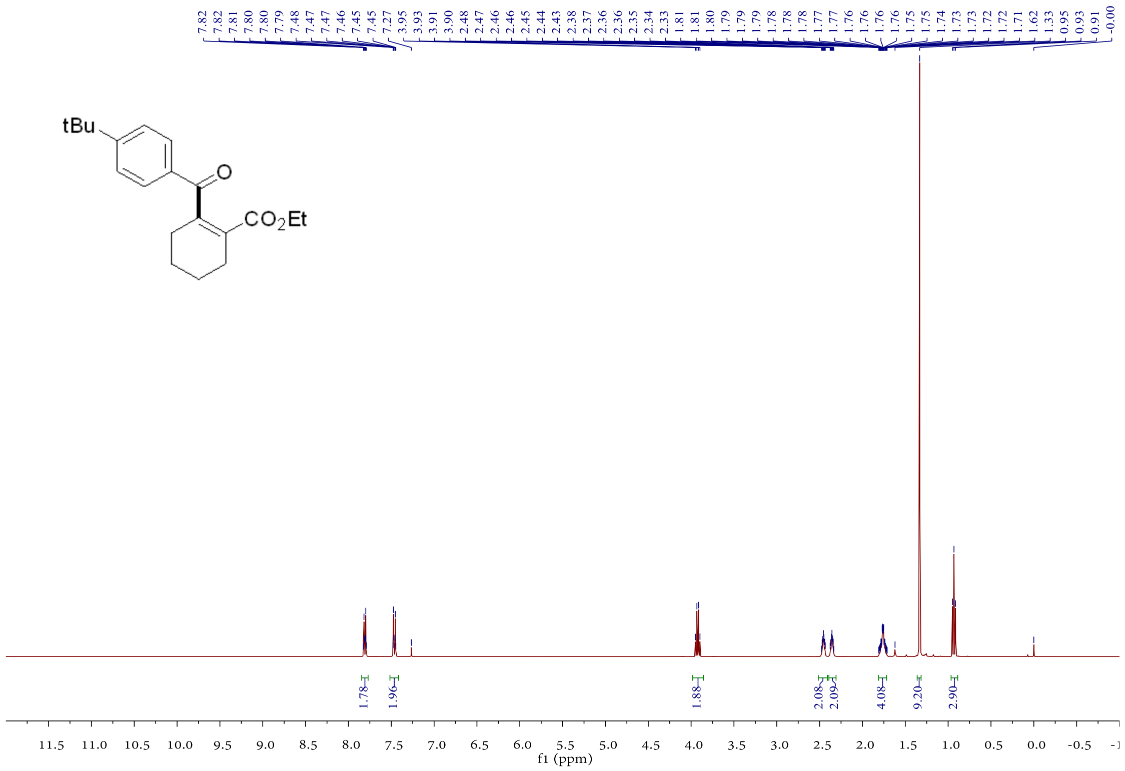

Supplementary Figure 96. ^{1}H NMR spectrum for compound **3d**

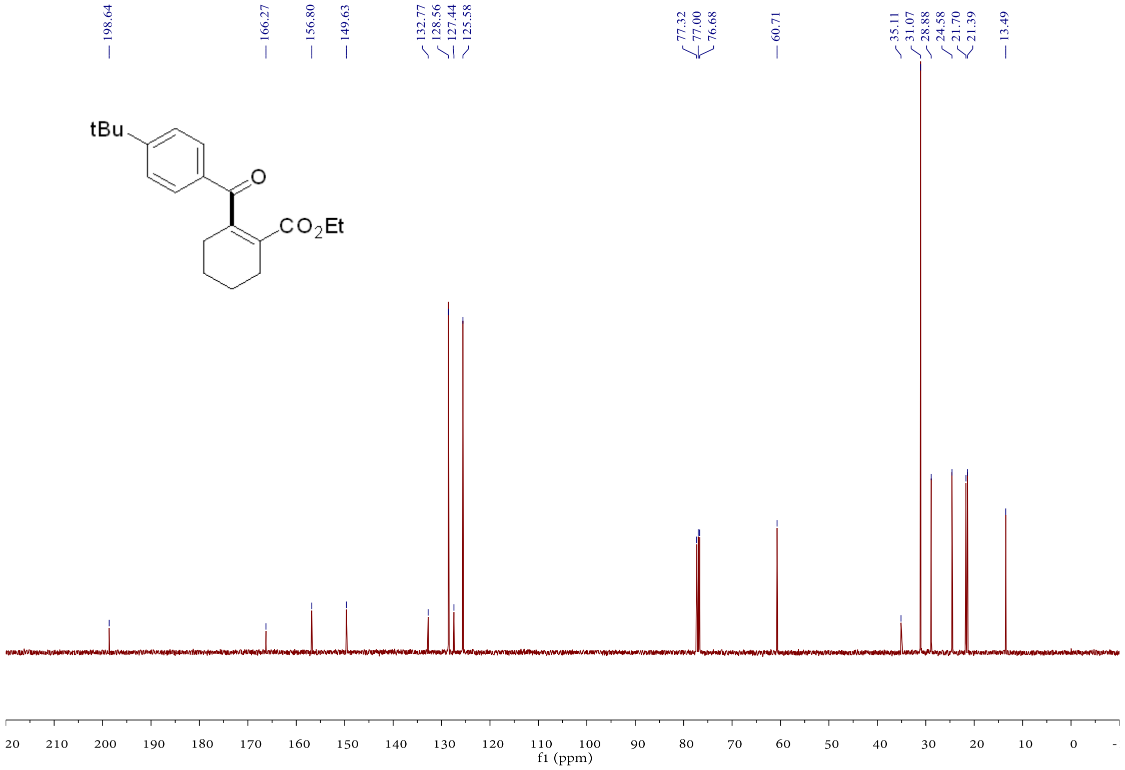

Supplementary Figure 97. ^{13}C NMR spectrum for compound **3d**

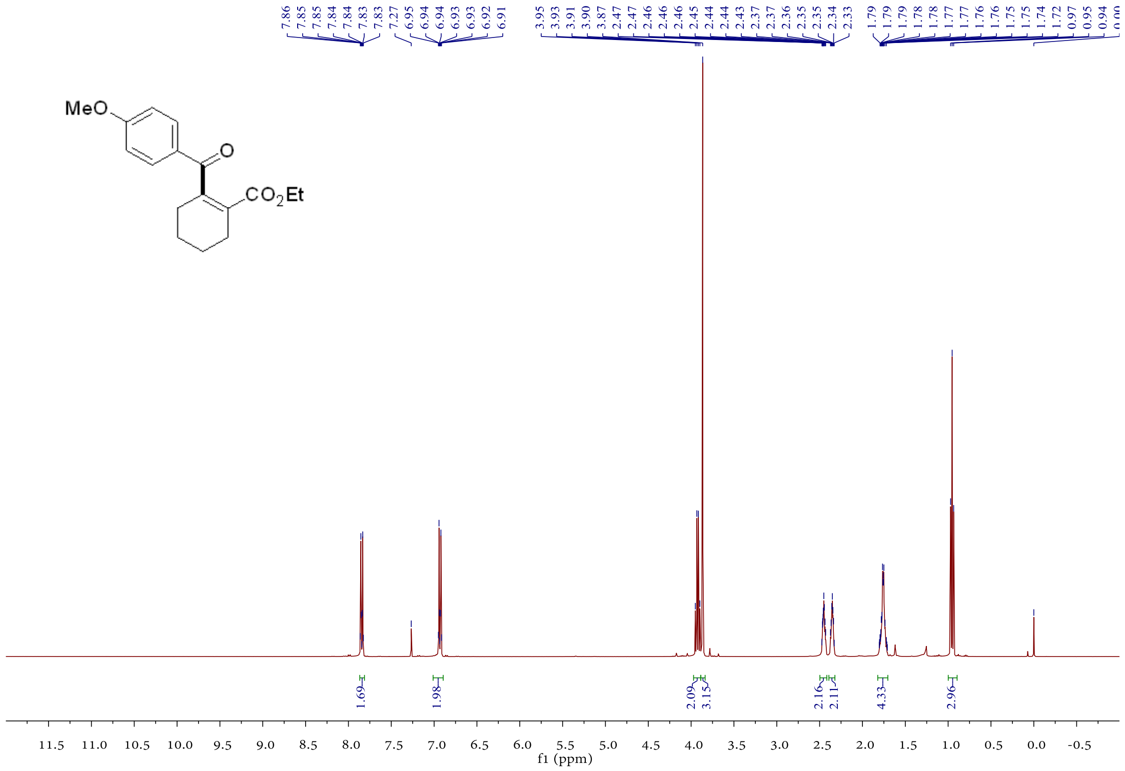

Supplementary Figure 98. ^{1}H NMR spectrum for compound **3e**

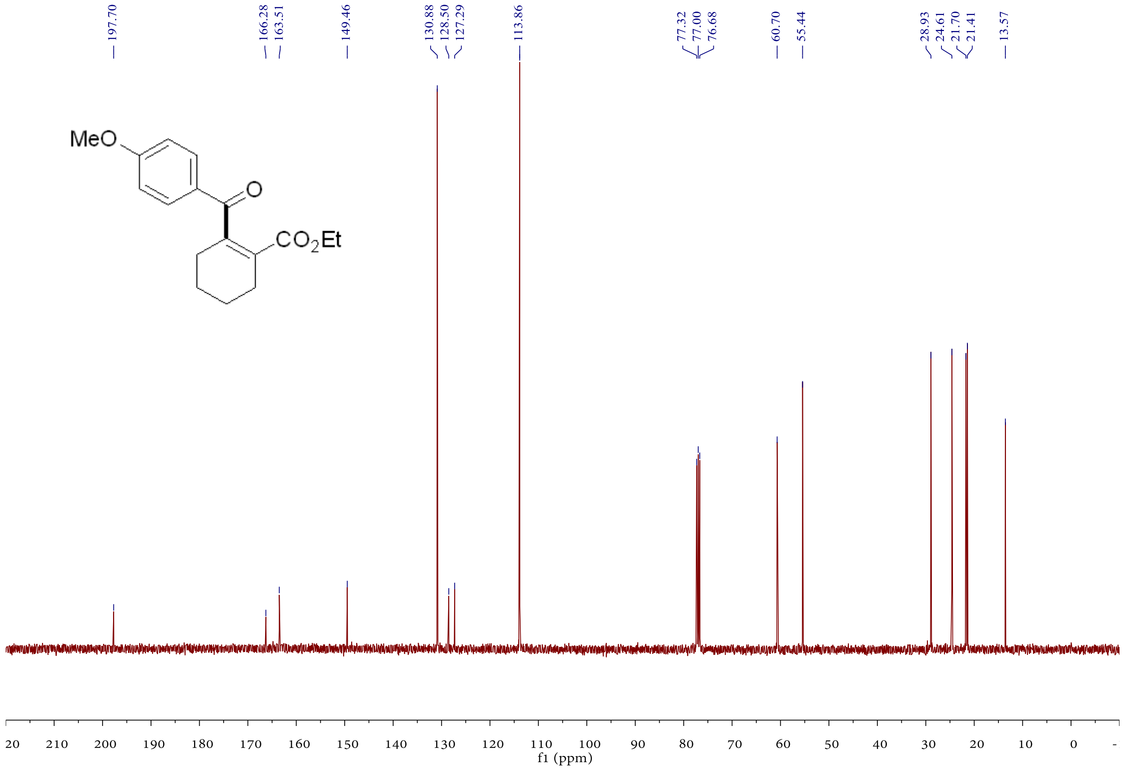

Supplementary Figure 99. ^{13}C NMR spectrum for compound **3e**

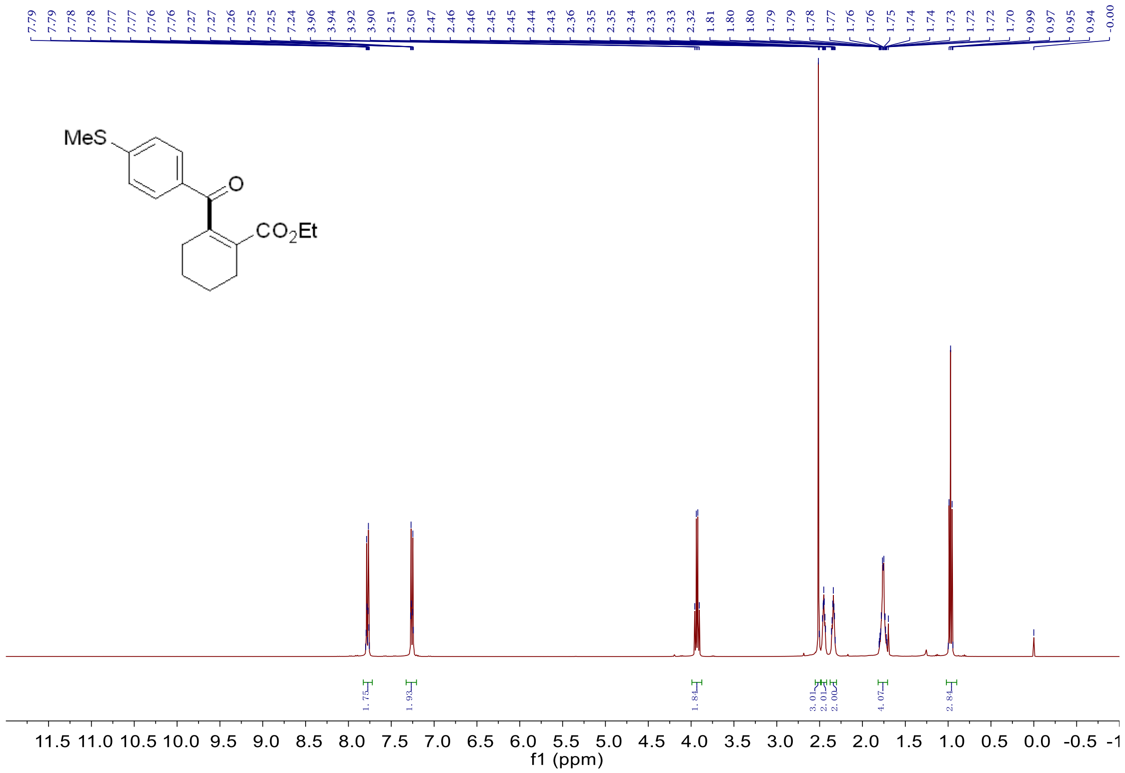

Supplementary Figure 100. ^{1}H NMR spectrum for compound **3f**

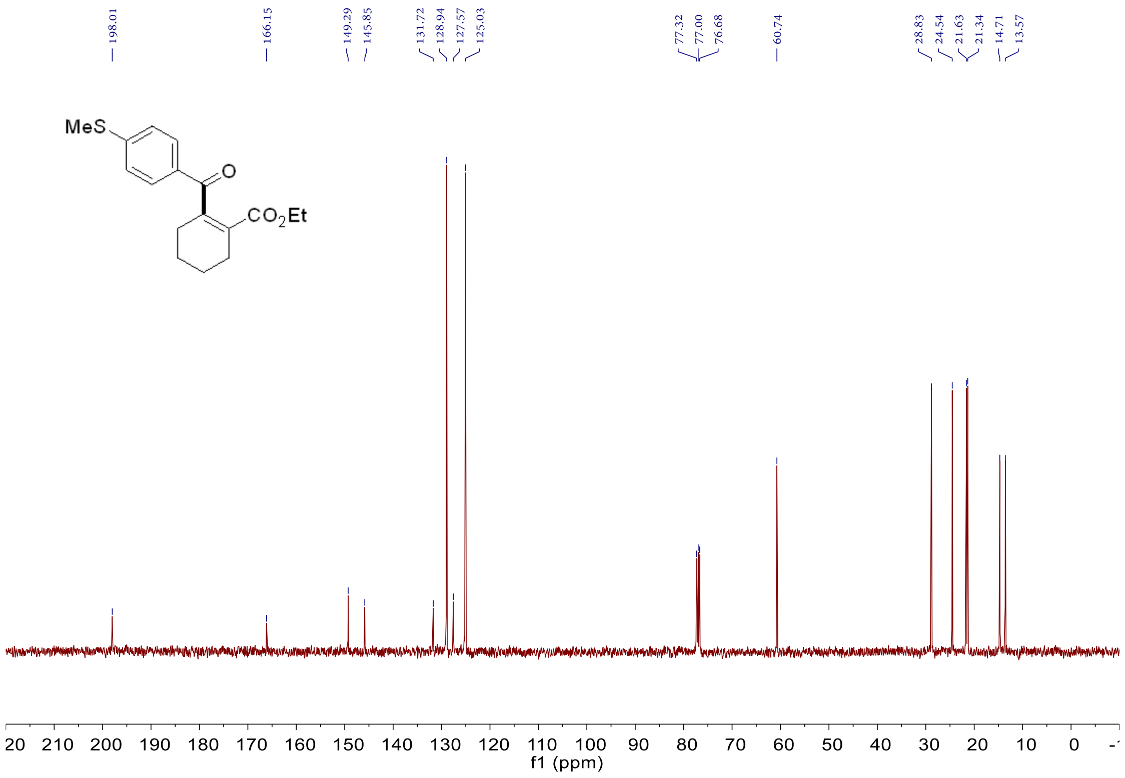

Supplementary Figure 101. ^{13}C NMR spectrum for compound **3f**

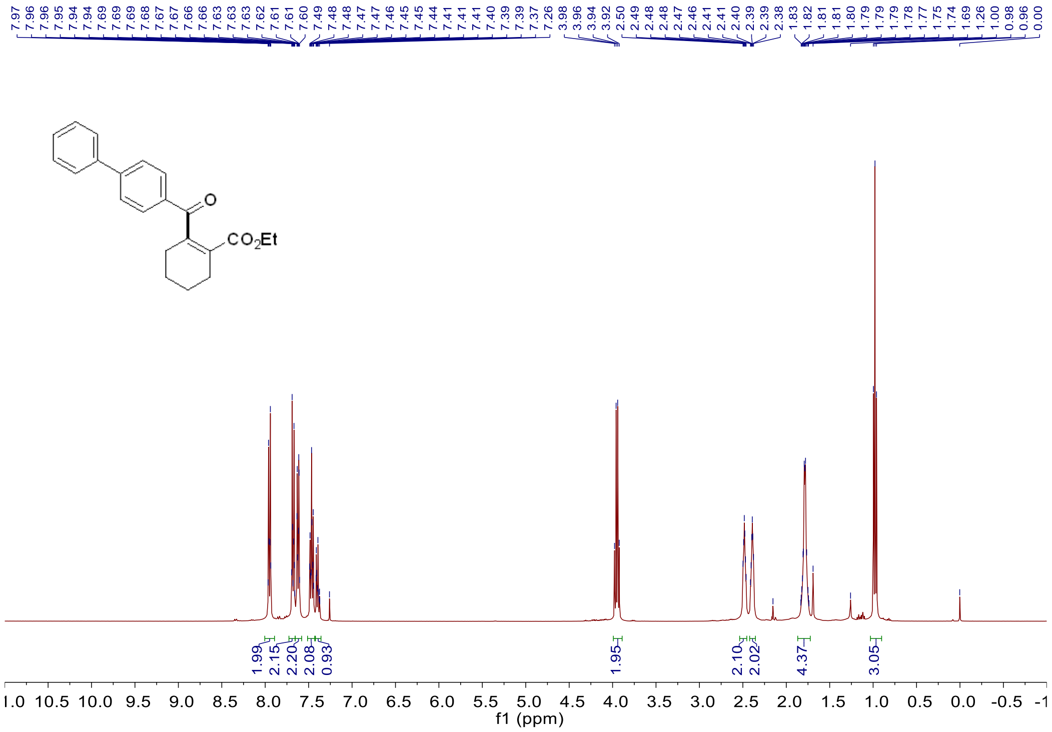

Supplementary Figure 102. ^{1}H NMR spectrum for compound **3g**

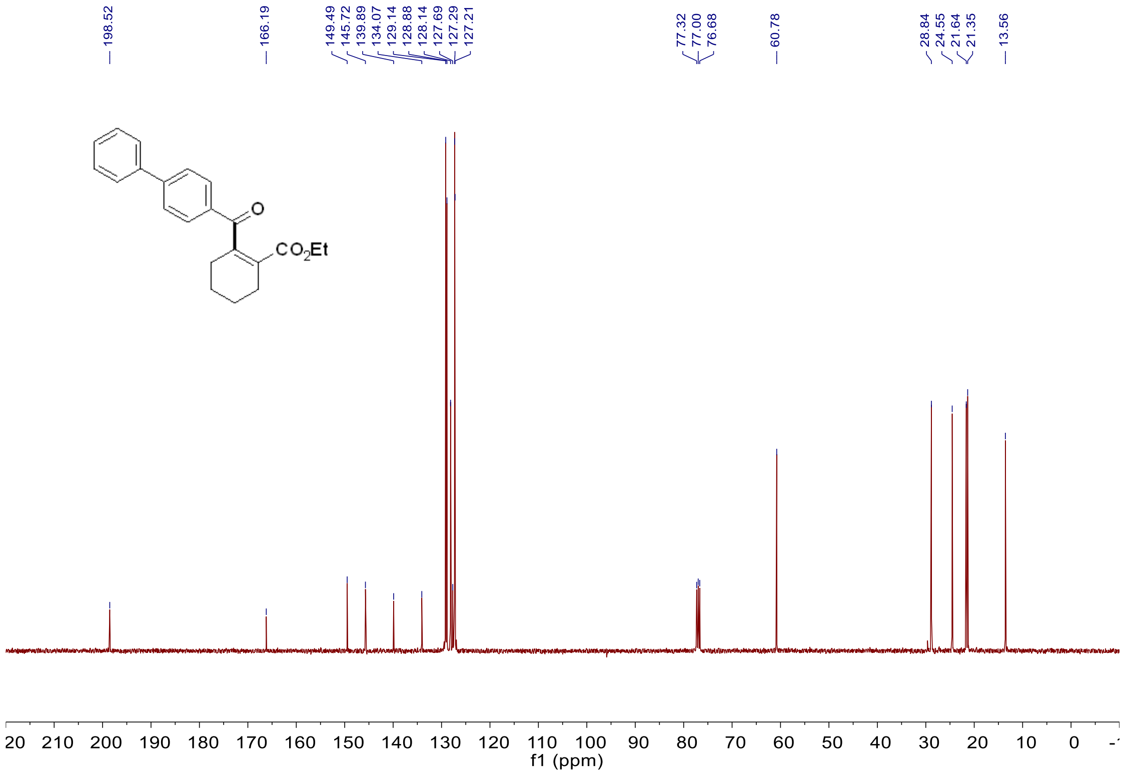

Supplementary Figure 103. ^{13}C NMR spectrum for compound **3g**

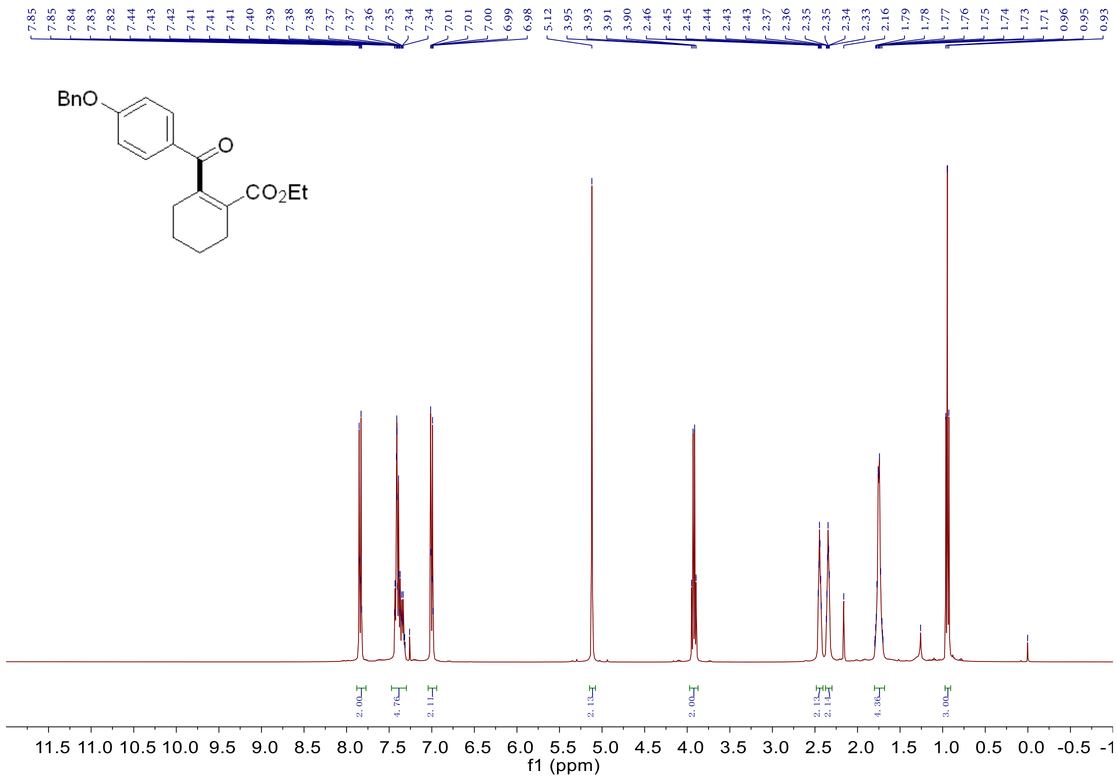

Supplementary Figure 104. ^{1}H NMR spectrum for compound **3h**

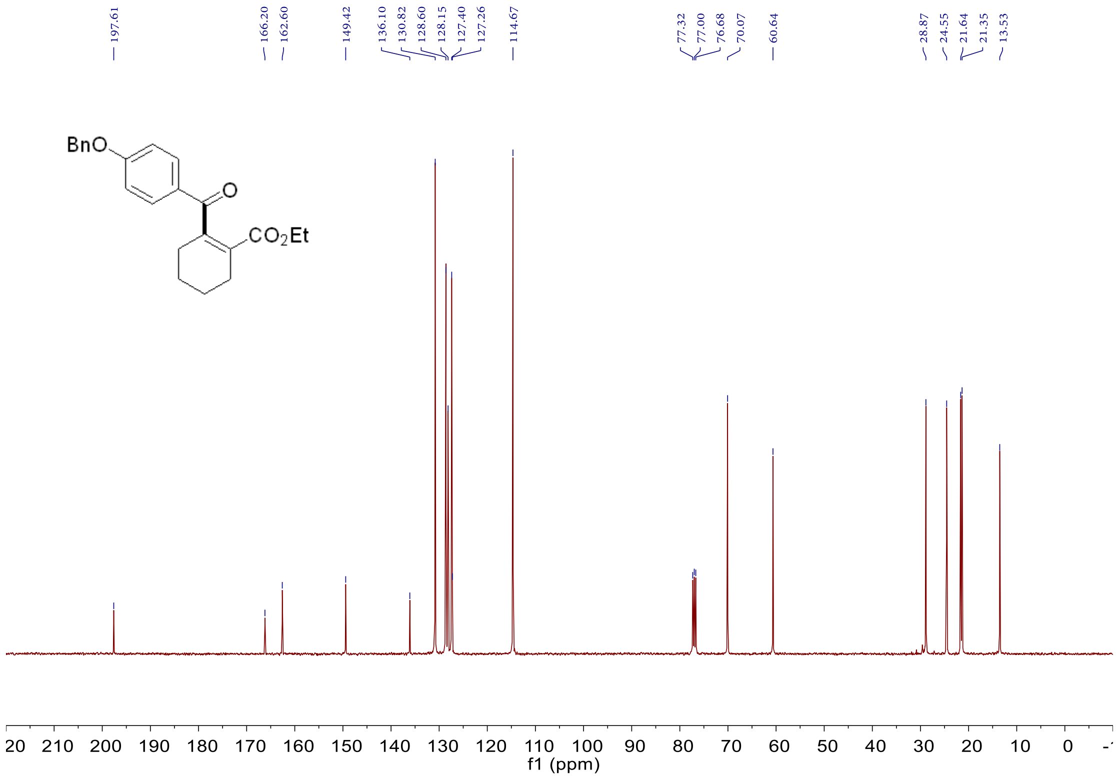

Supplementary Figure 105. ^{13}C NMR spectrum for compound **3h**

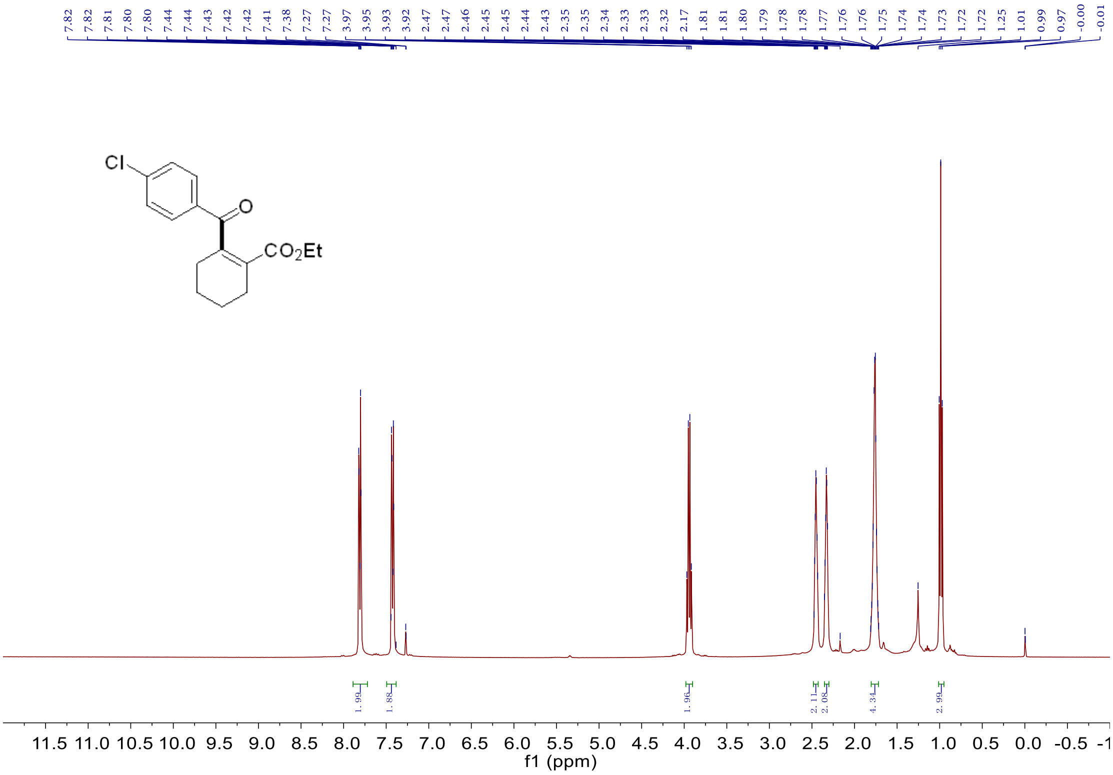

Supplementary Figure 106. ^{1}H NMR spectrum for compound **3i**

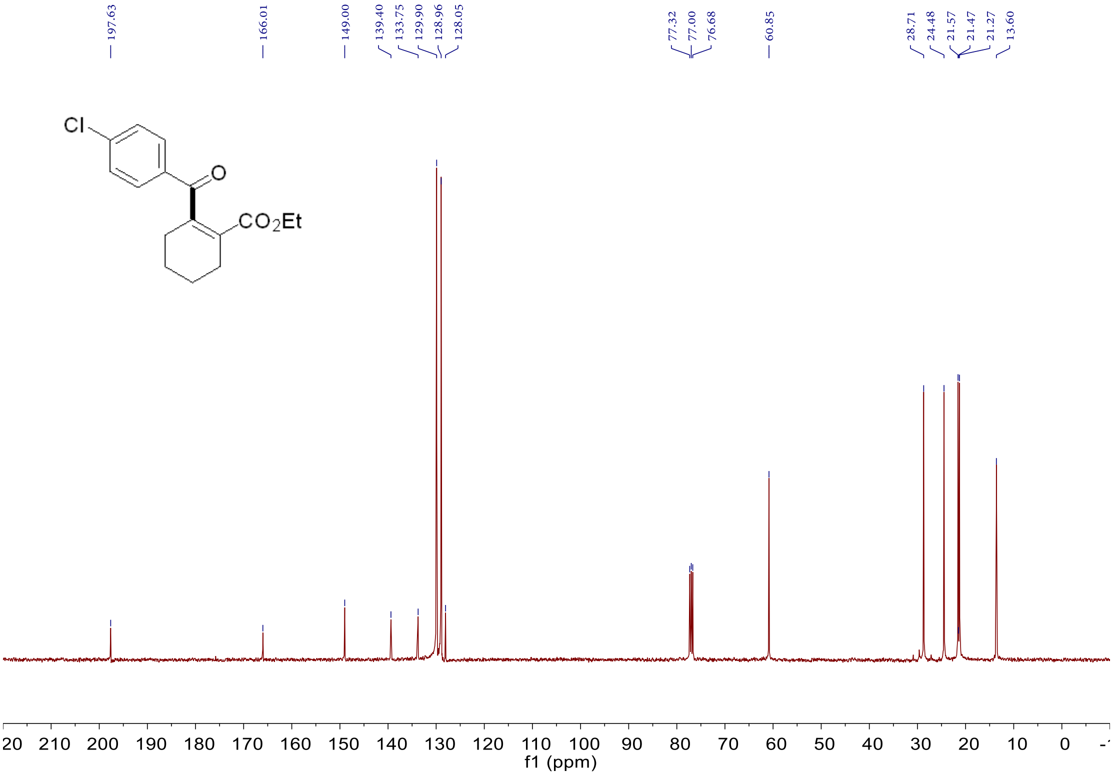

Supplementary Figure 107. ^{13}C NMR spectrum for compound **3i**

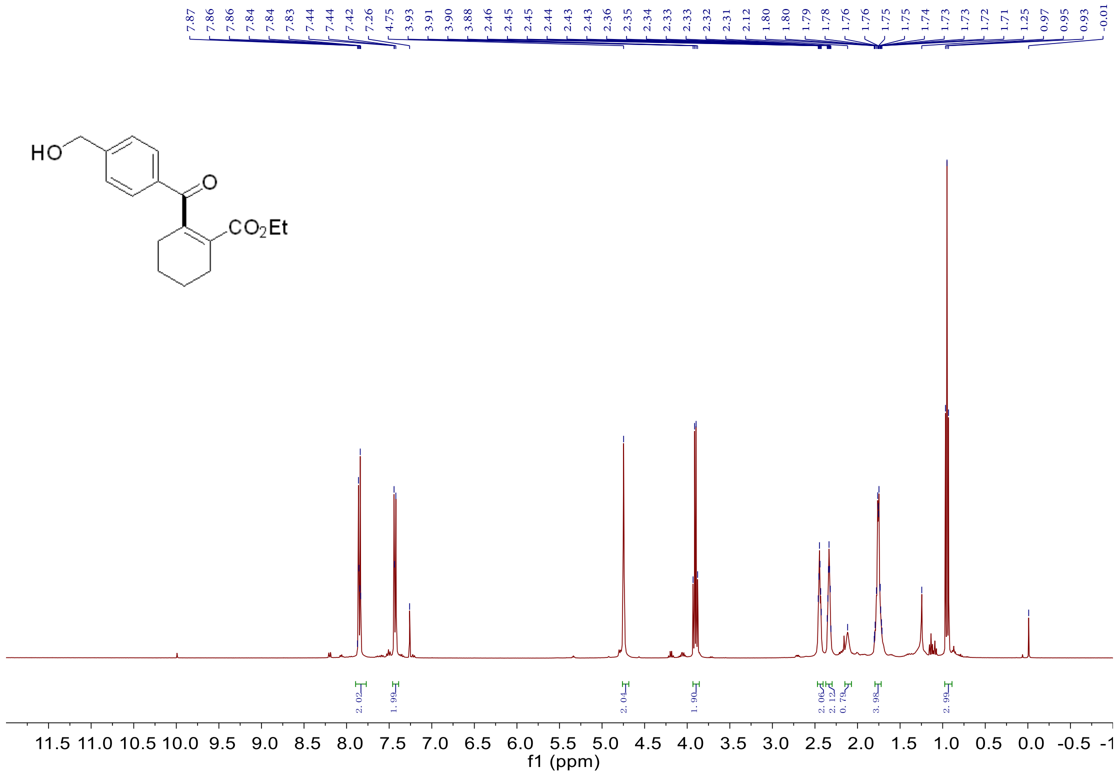

Supplementary Figure 108. ^{1}H NMR spectrum for compound **3j**

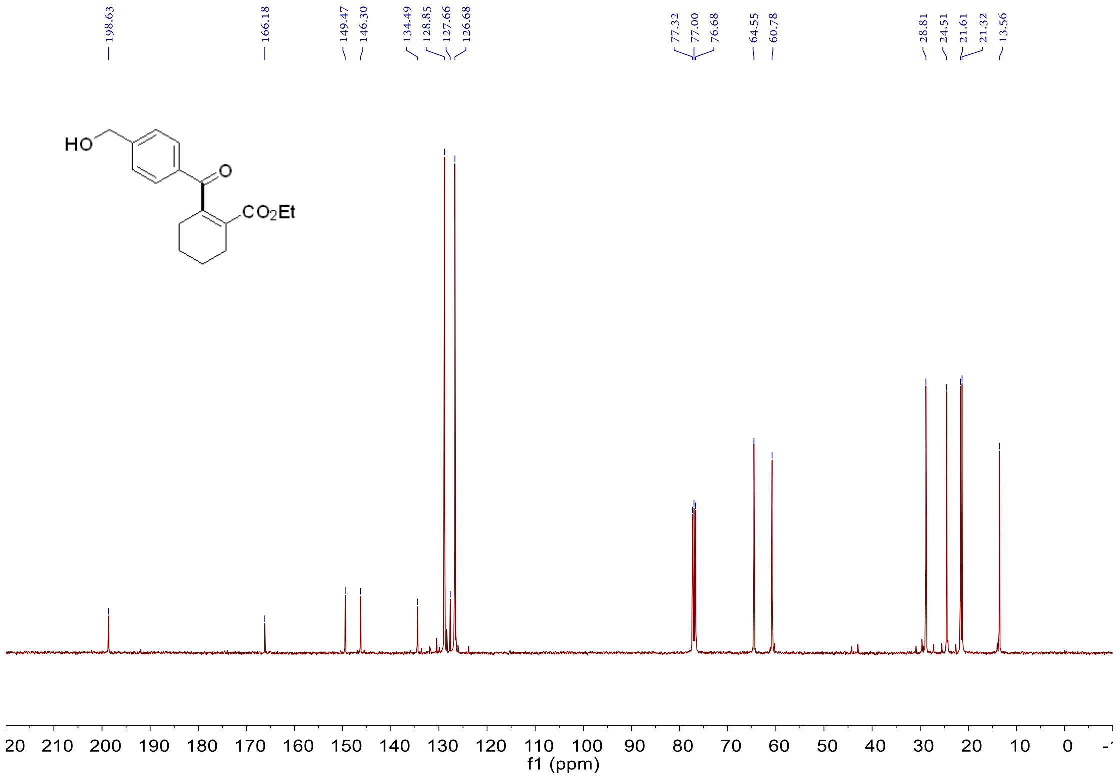

Supplementary Figure 109. ^{13}C NMR spectrum for compound **3j**

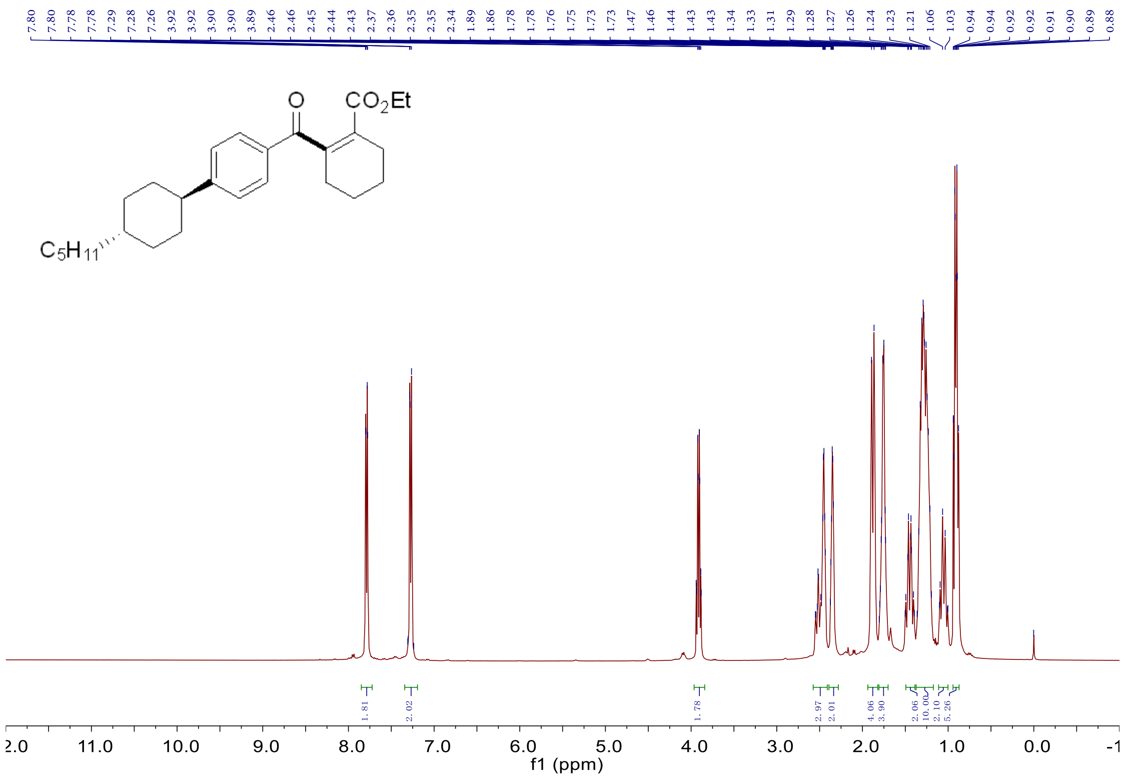

Supplementary Figure 110. ^{1}H NMR spectrum for compound **3k**

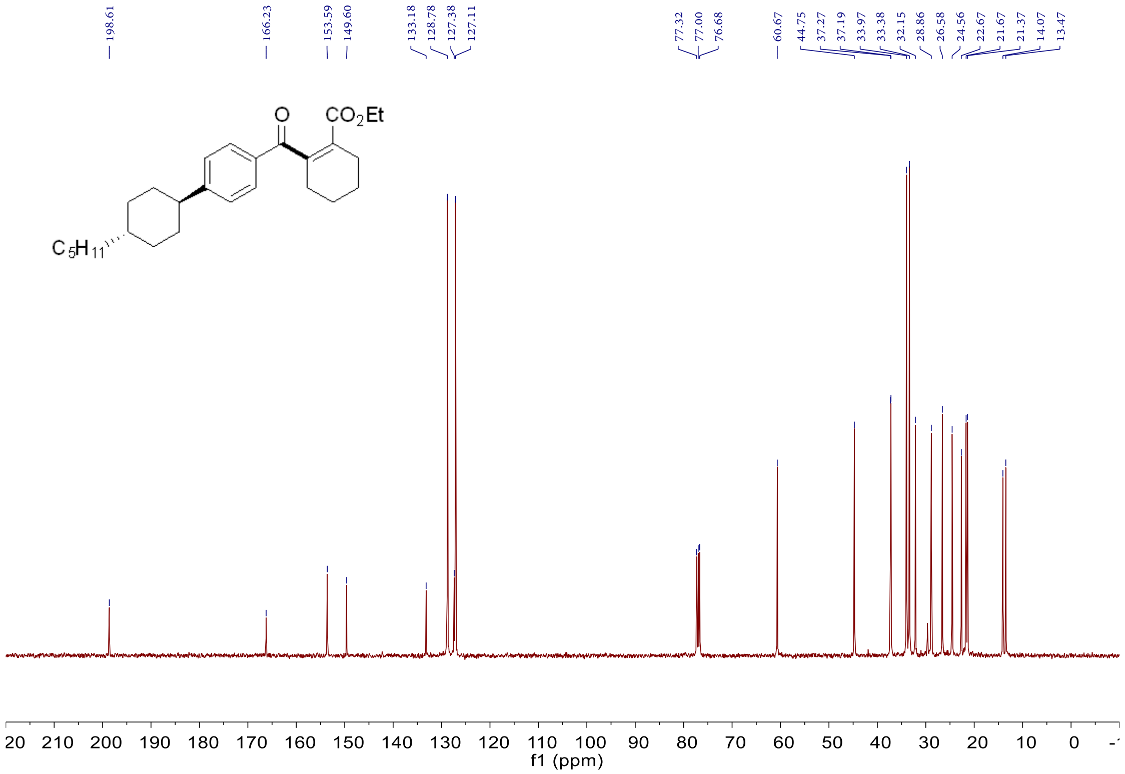

Supplementary Figure 111. ^{13}C NMR spectrum for compound **3k**

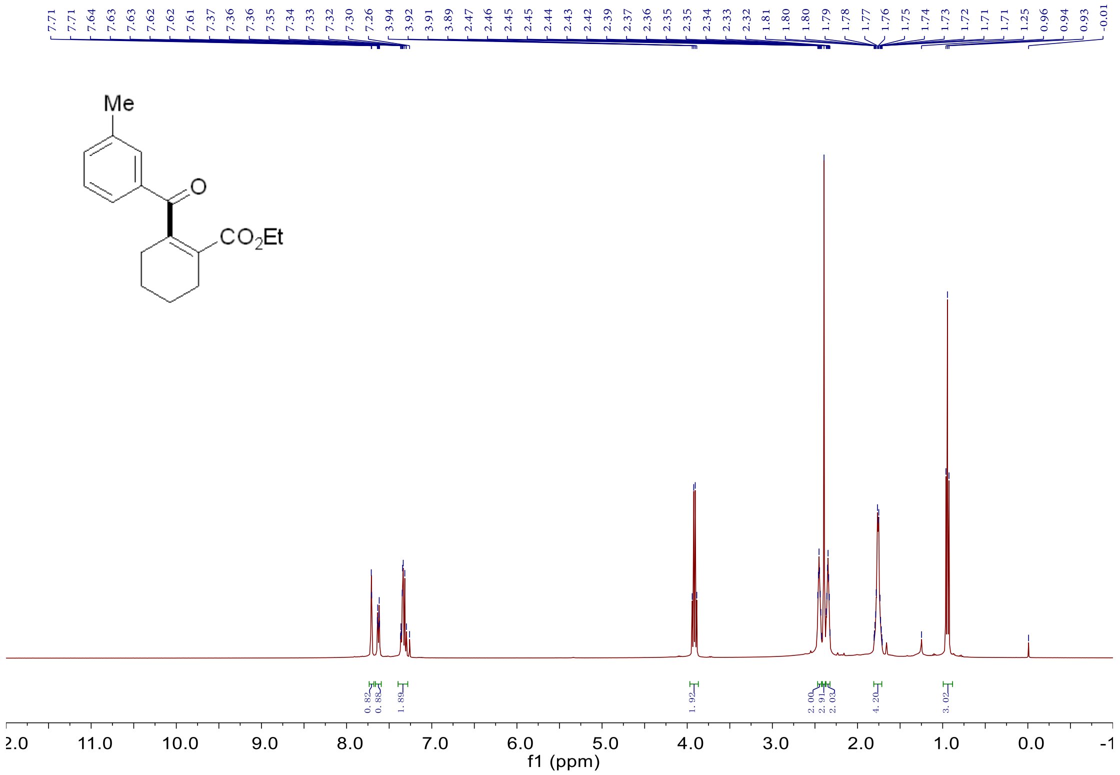

Supplementary Figure 112. ^{1}H NMR spectrum for compound **3l**

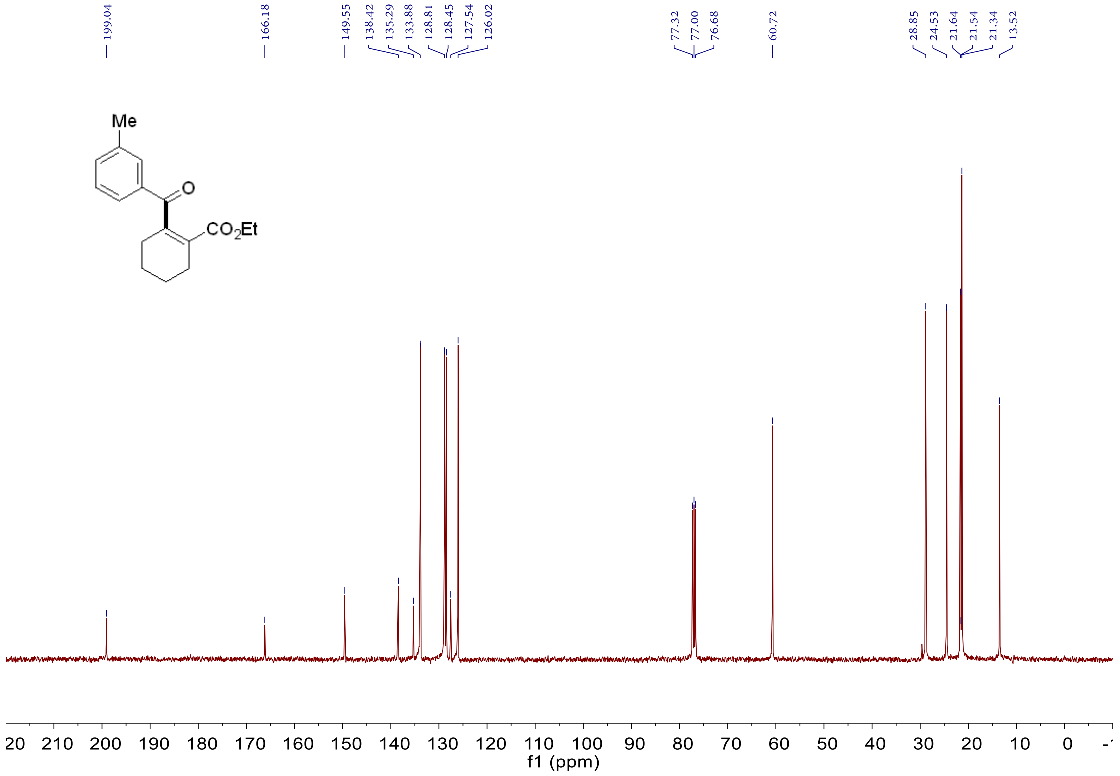

Supplementary Figure 113. ^{13}C NMR spectrum for compound **3l**

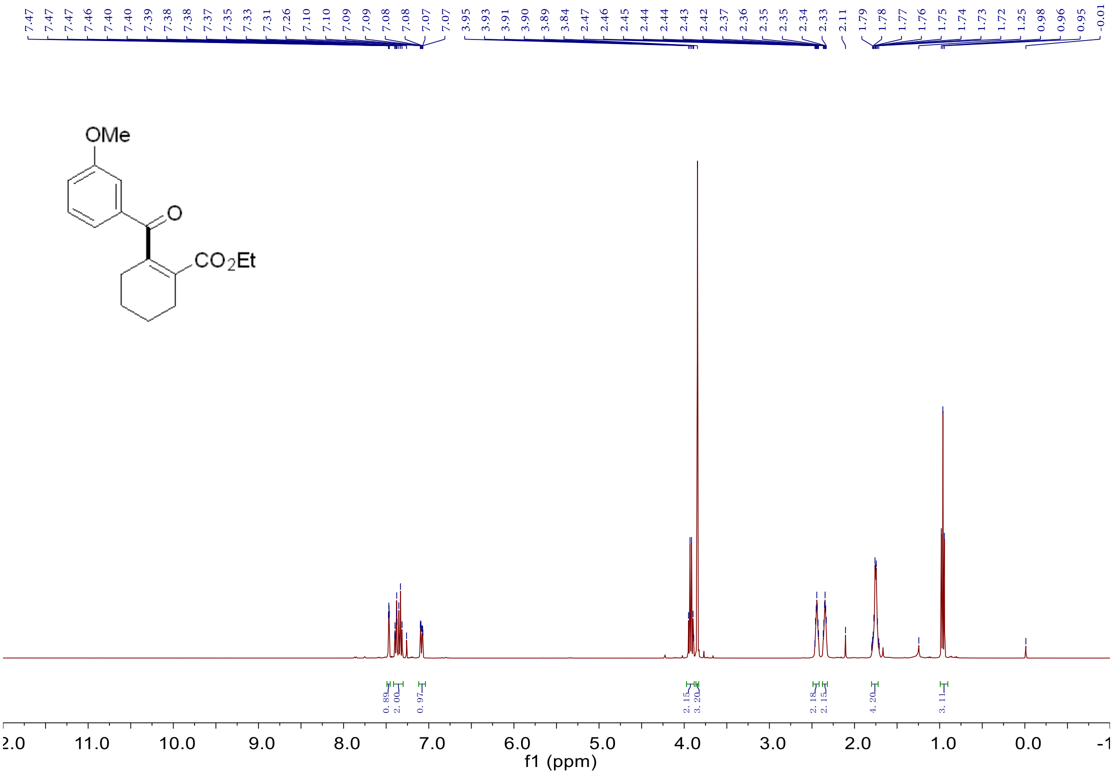

Supplementary Figure 114. ^{1}H NMR spectrum for compound **3m**

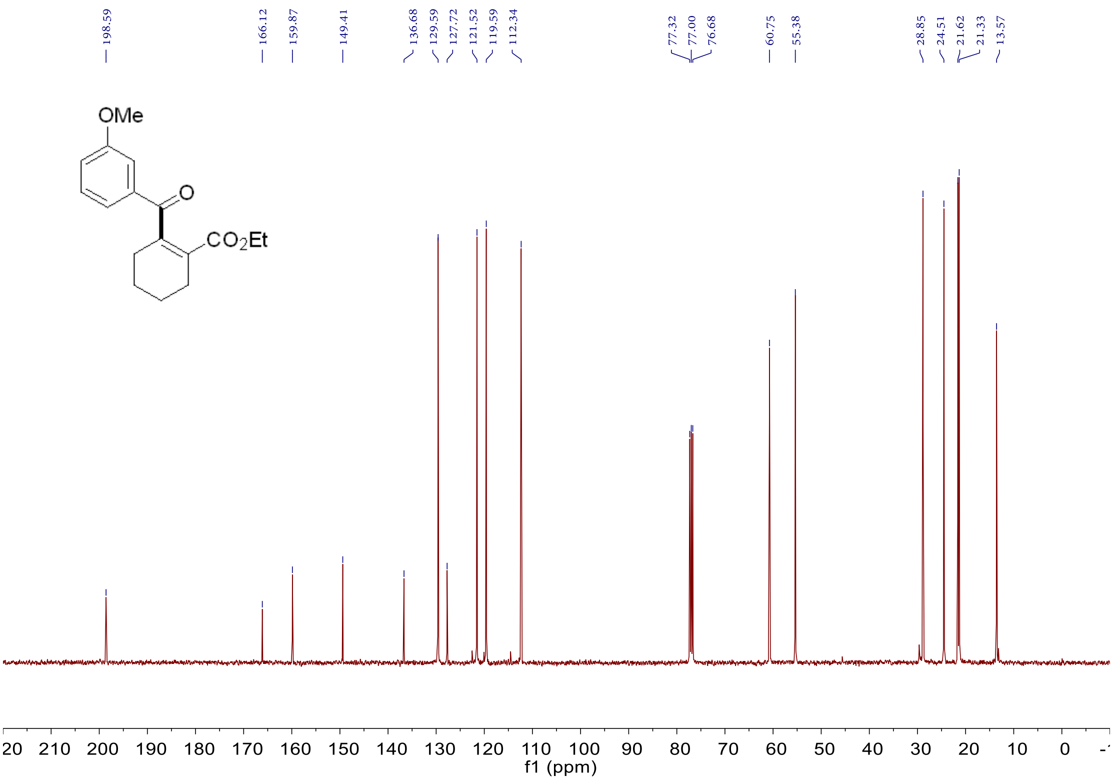

Supplementary Figure 115. ^{13}C NMR spectrum for compound **3m**

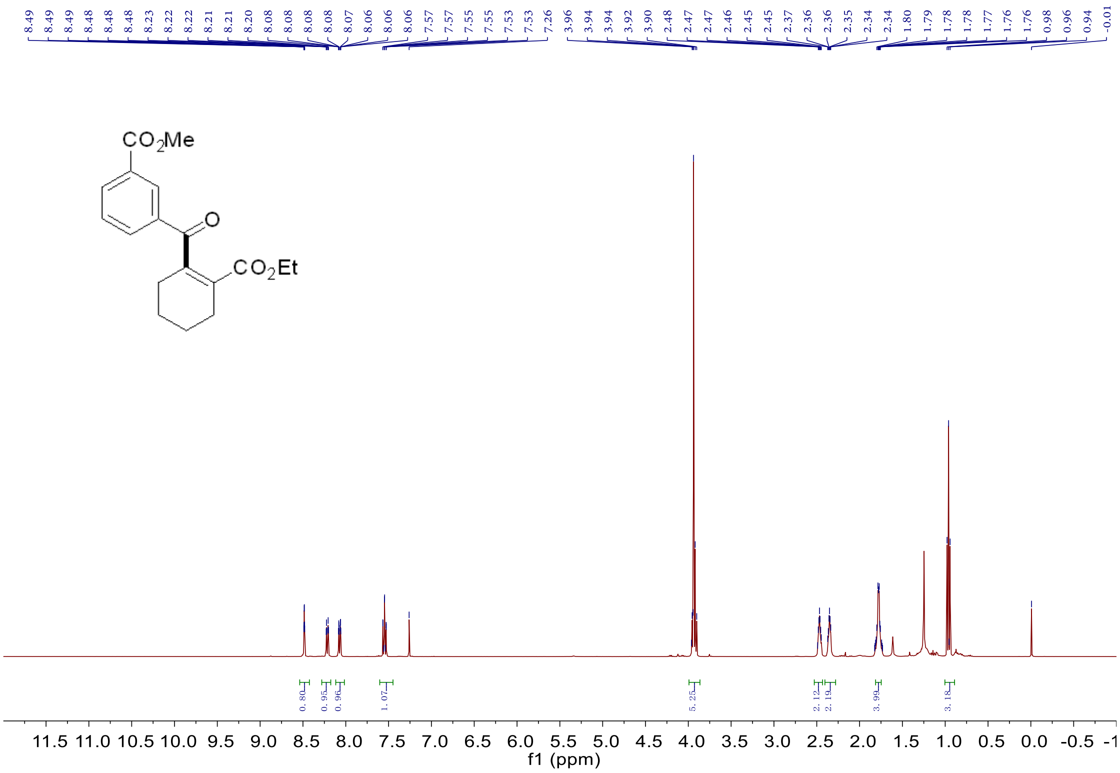

Supplementary Figure 116. ^{1}H NMR spectrum for compound **3n**

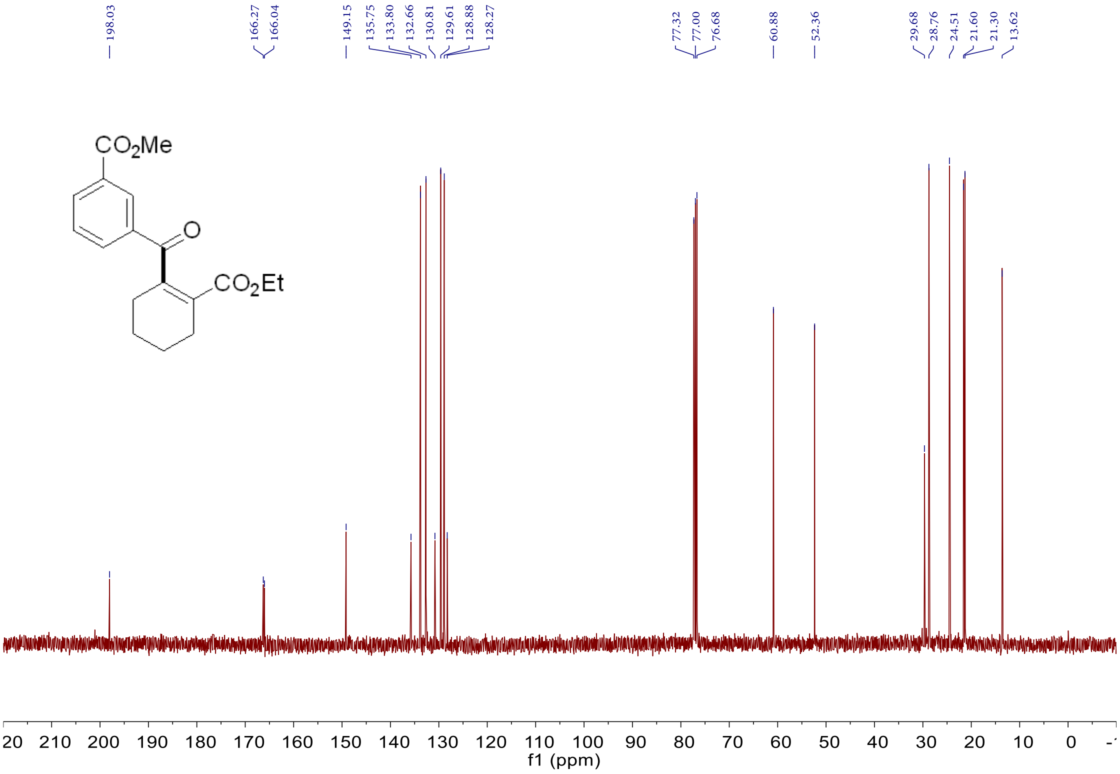

Supplementary Figure 117. ^{13}C NMR spectrum for compound **3n**

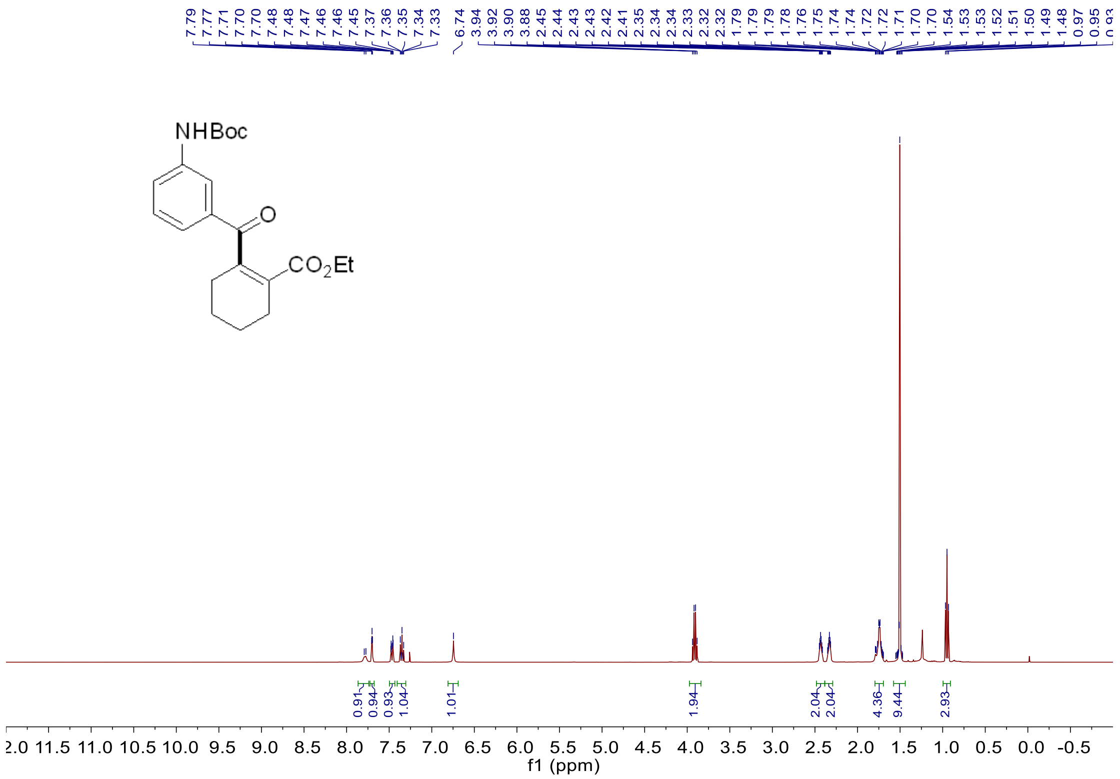

Supplementary Figure 118. ^{1}H NMR spectrum for compound **3o**

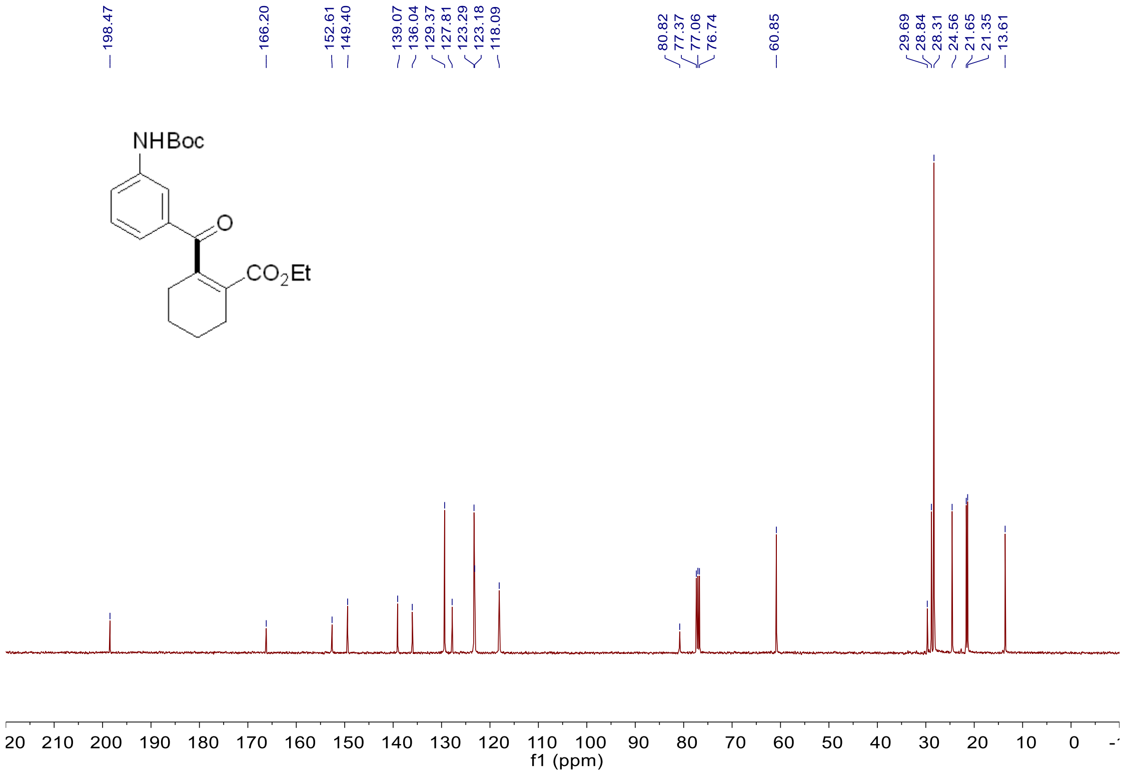

Supplementary Figure 119. ^{13}C NMR spectrum for compound **3o**

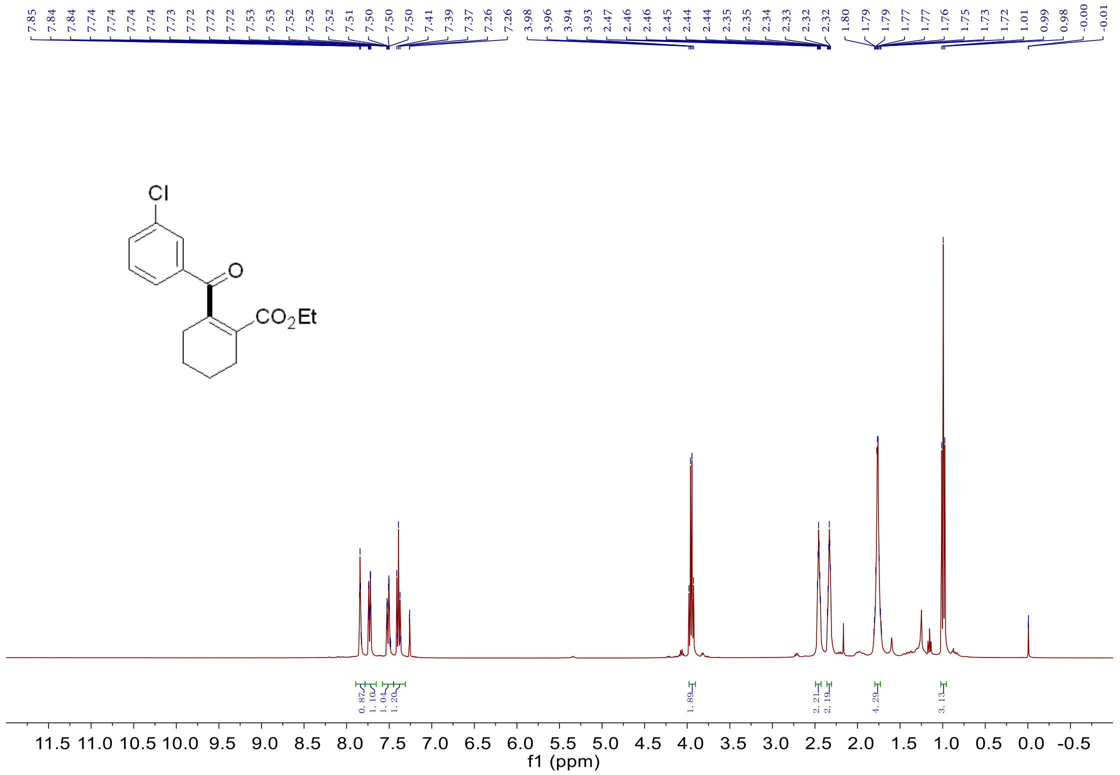

Supplementary Figure 120. ^{1}H NMR spectrum for compound **3p**

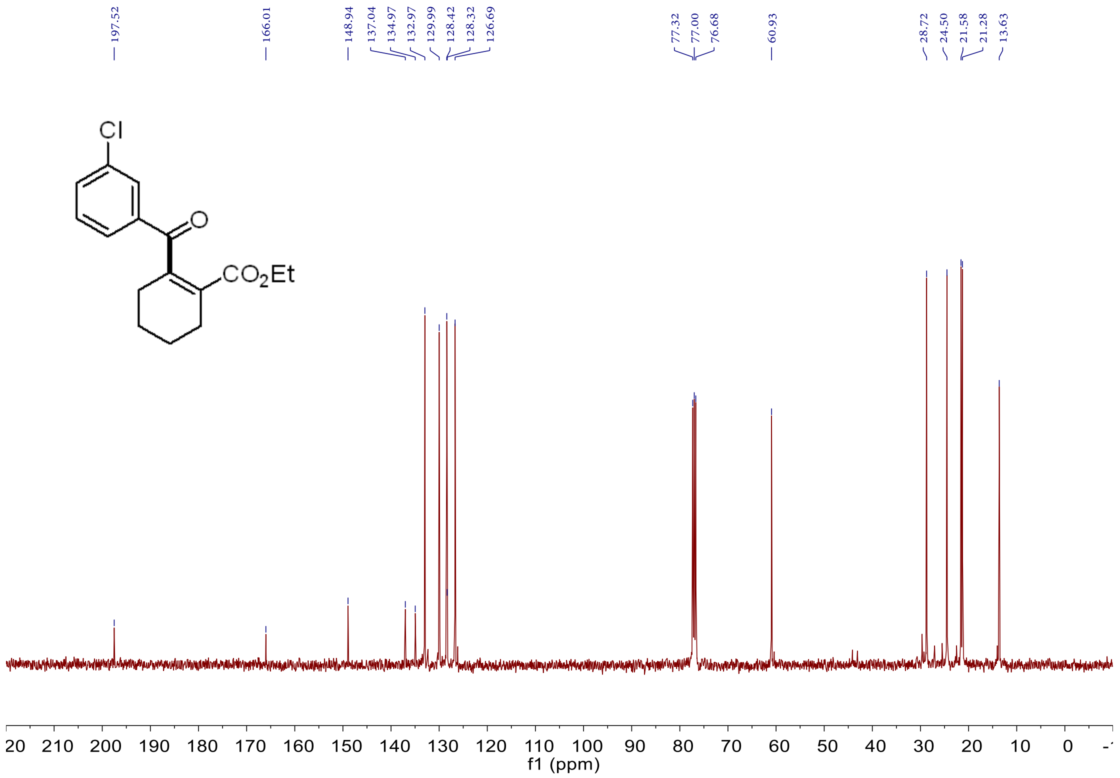

Supplementary Figure 121. ^{13}C NMR spectrum for compound **3p**

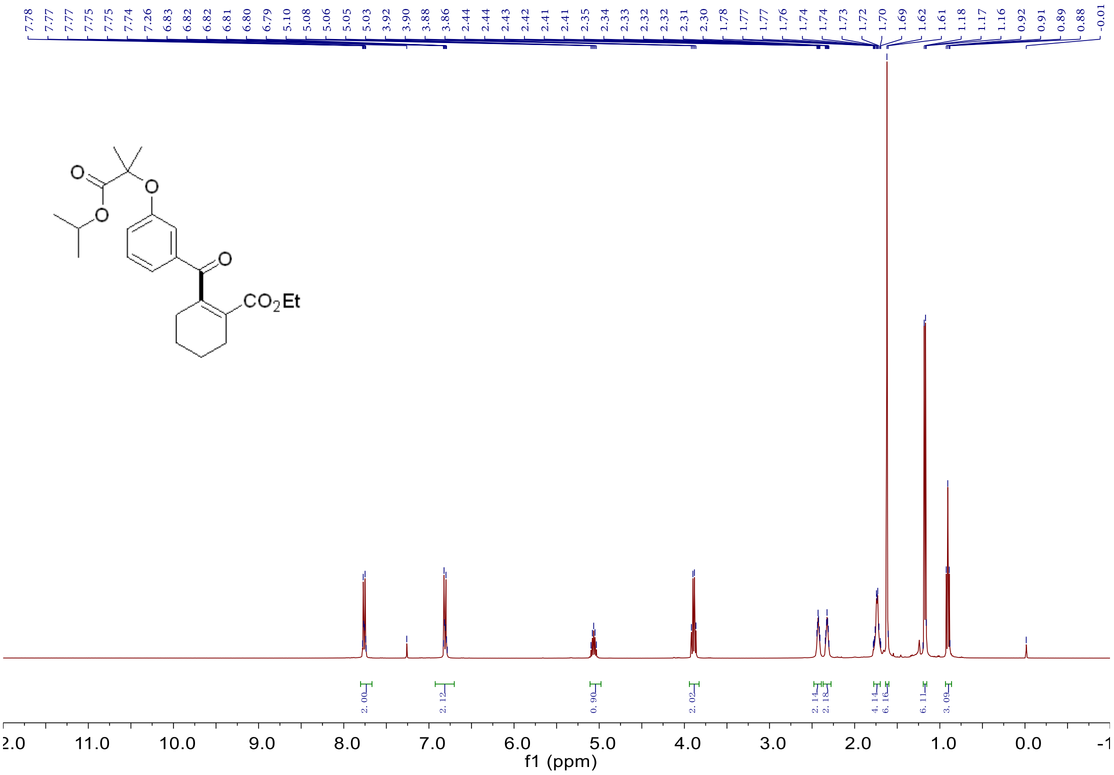

Supplementary Figure 122. ^{1}H NMR spectrum for compound **3q**

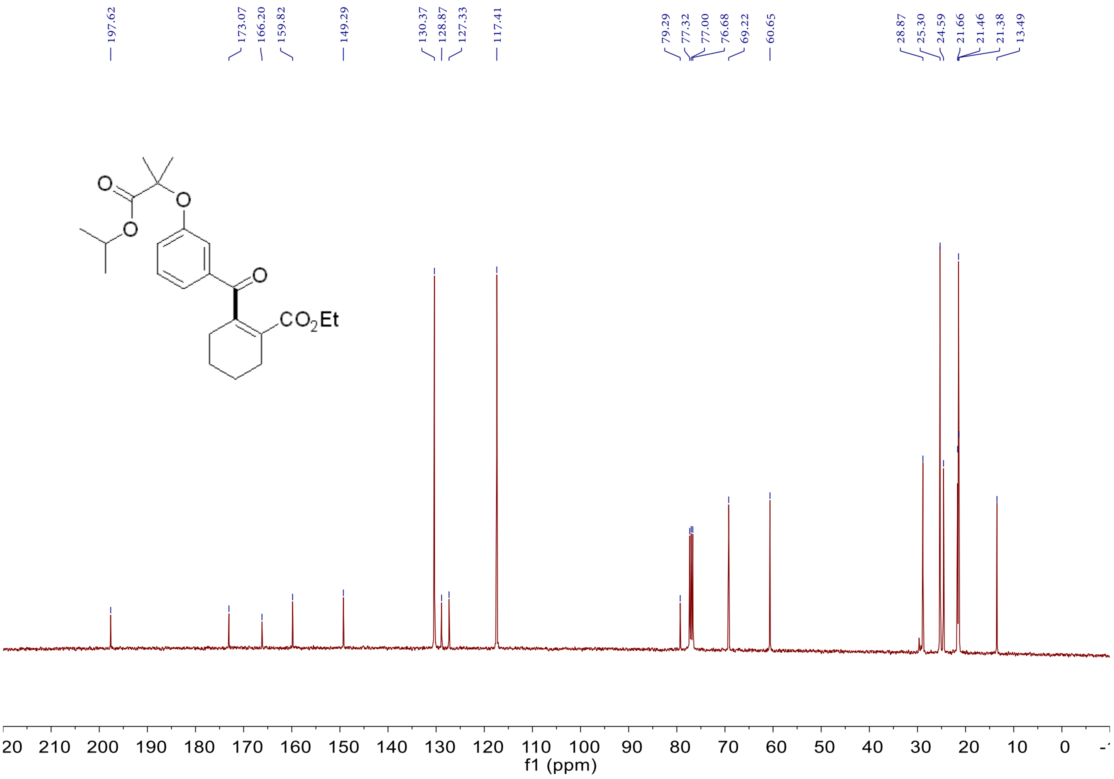

Supplementary Figure 123. ^{13}C NMR spectrum for compound **3q**

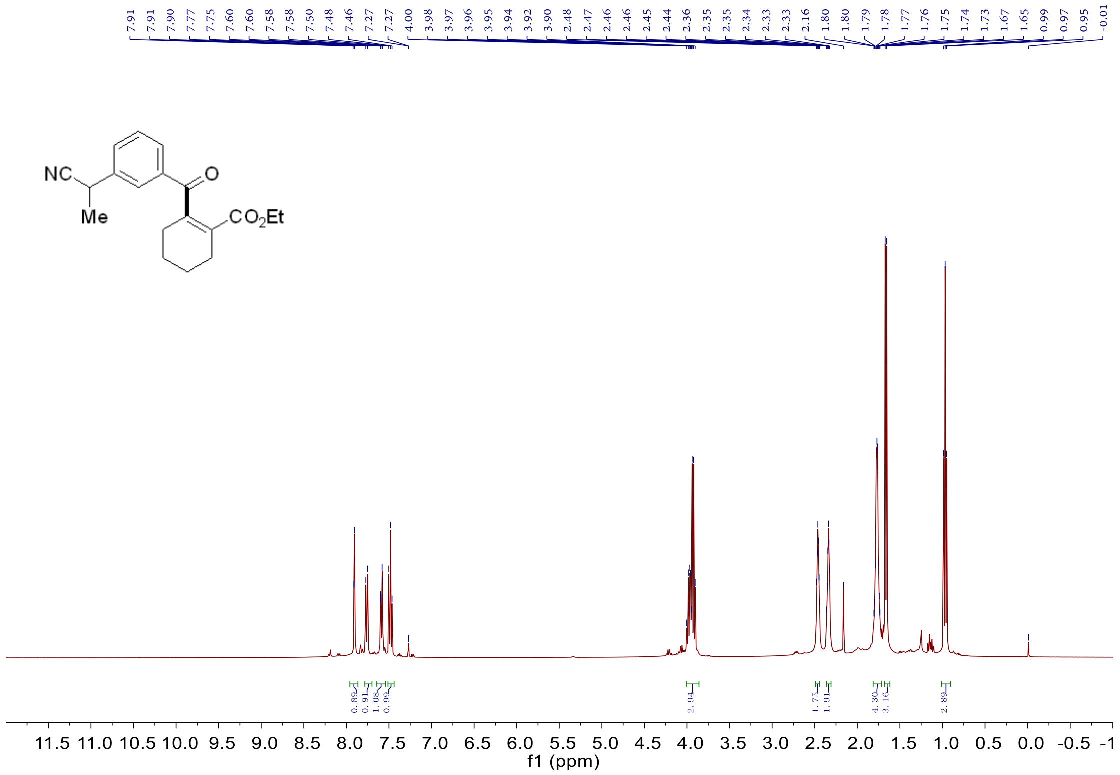

Supplementary Figure 124. ^{1}H NMR spectrum for compound **3r**

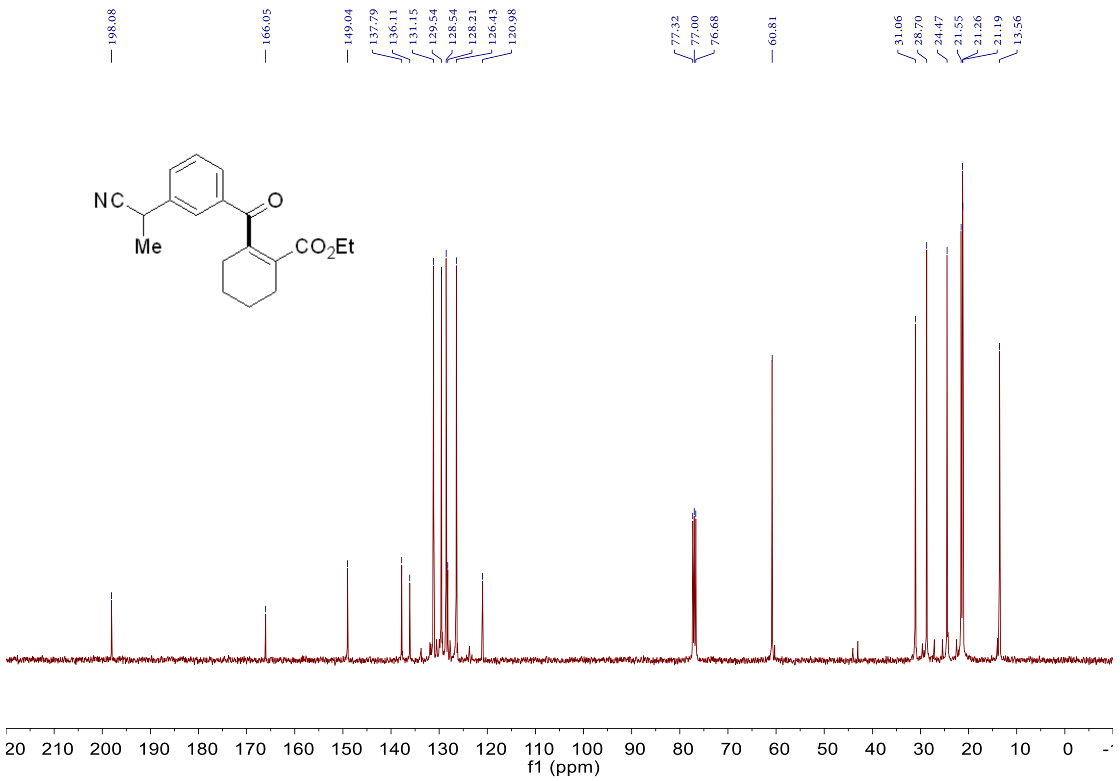

Supplementary Figure 125. ^{13}C NMR spectrum for compound **3r**

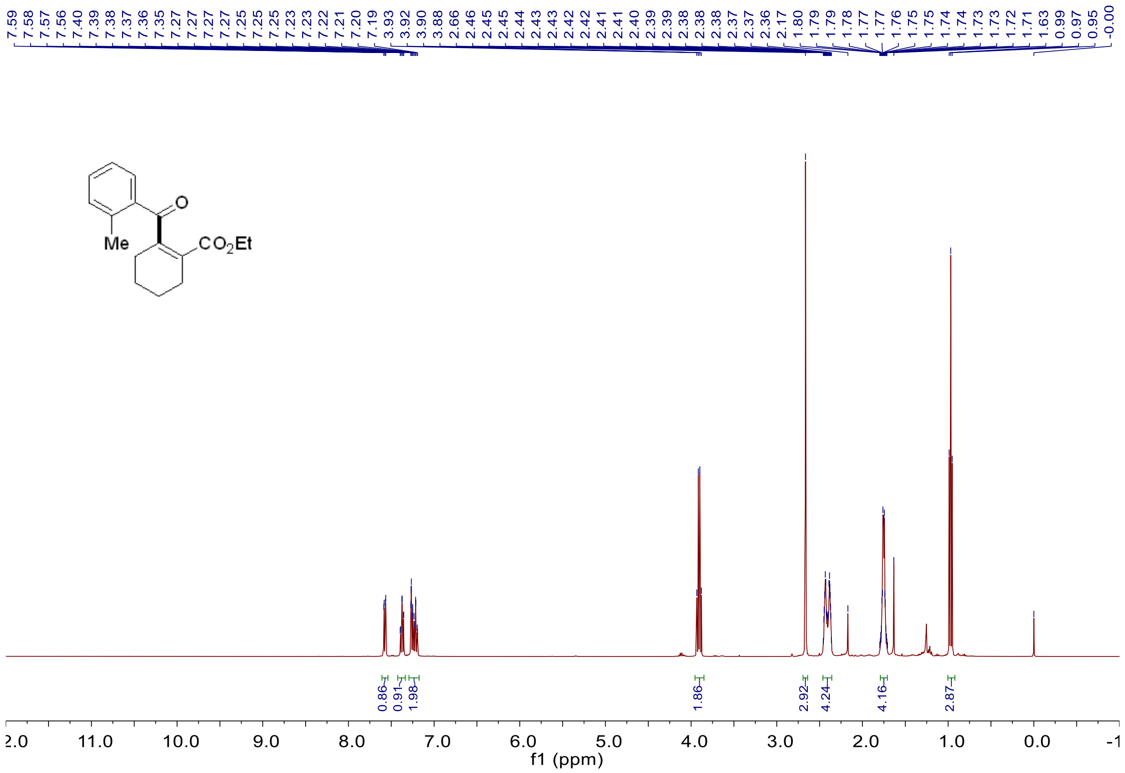

Supplementary Figure 126. ^{1}H NMR spectrum for compound **3s**

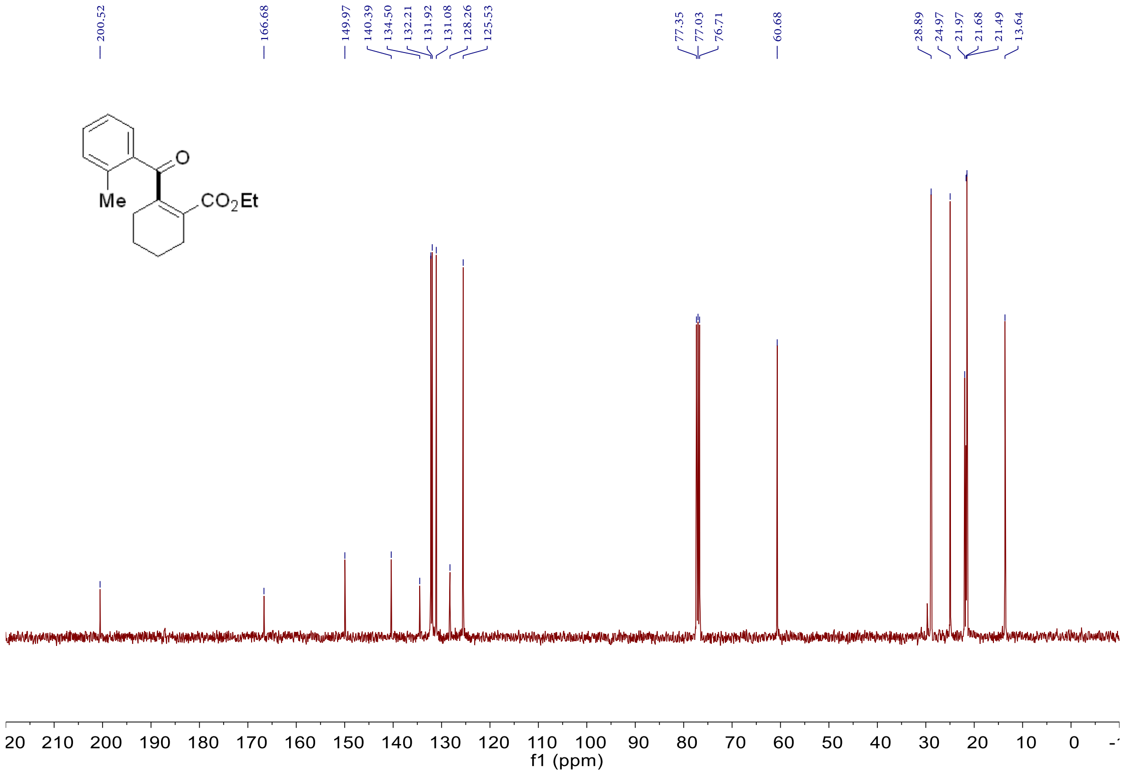

Supplementary Figure 127. ^{13}C NMR spectrum for compound **3s**

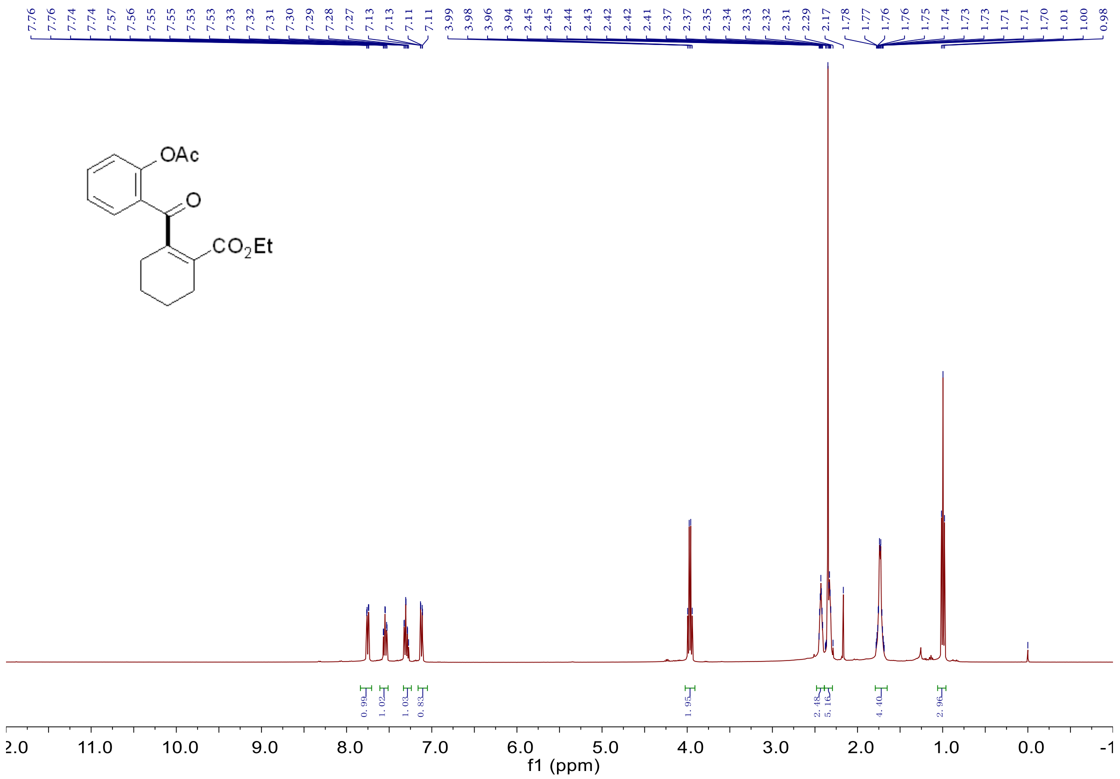

Supplementary Figure 128. ^{1}H NMR spectrum for compound **3t**

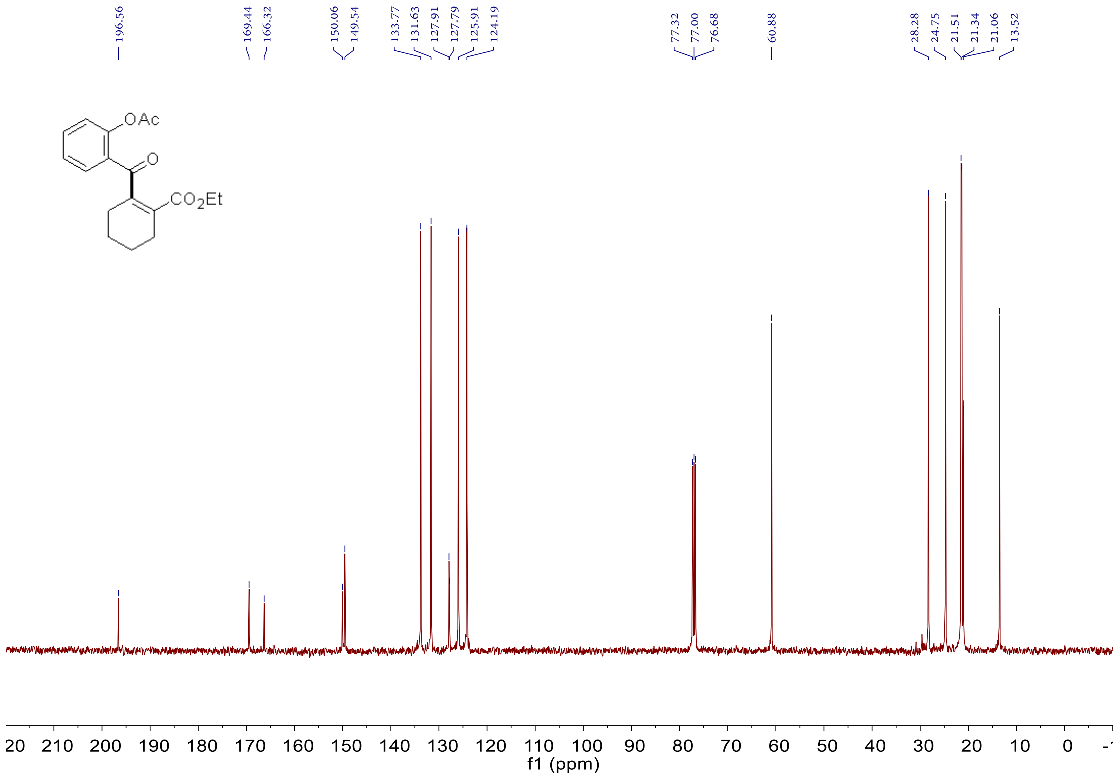

Supplementary Figure 129. ^{13}C NMR spectrum for compound **3t**

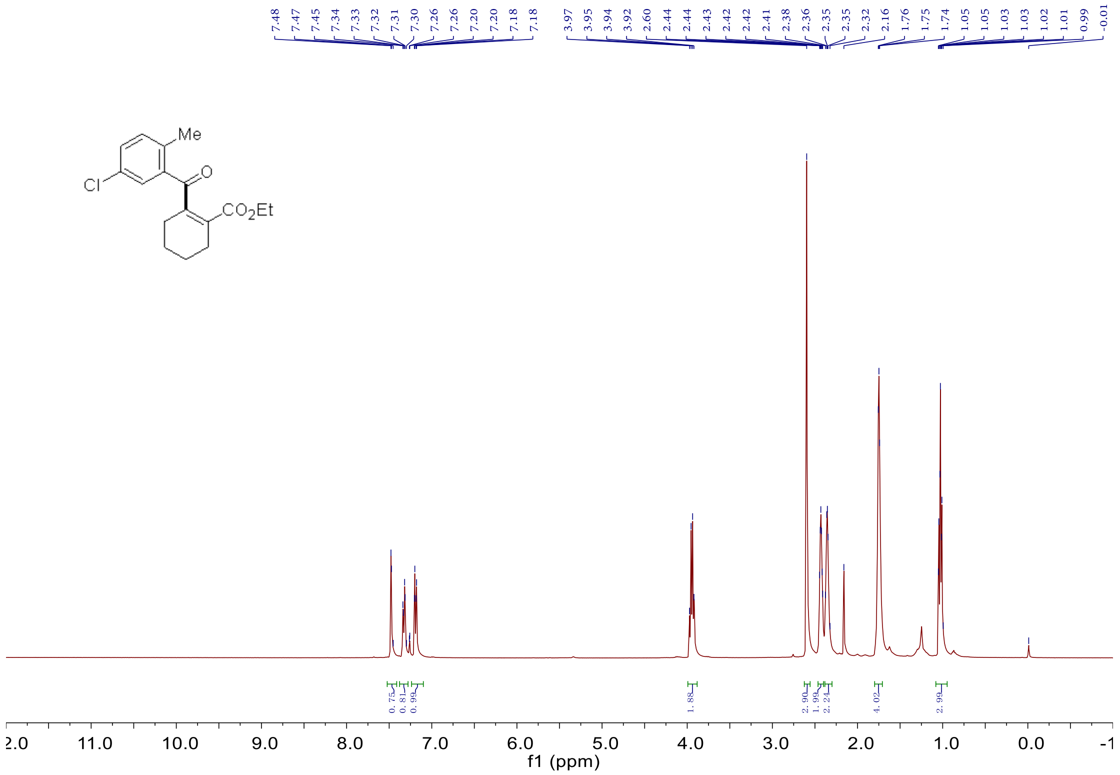

Supplementary Figure 130. ^{1}H NMR spectrum for compound **3u**

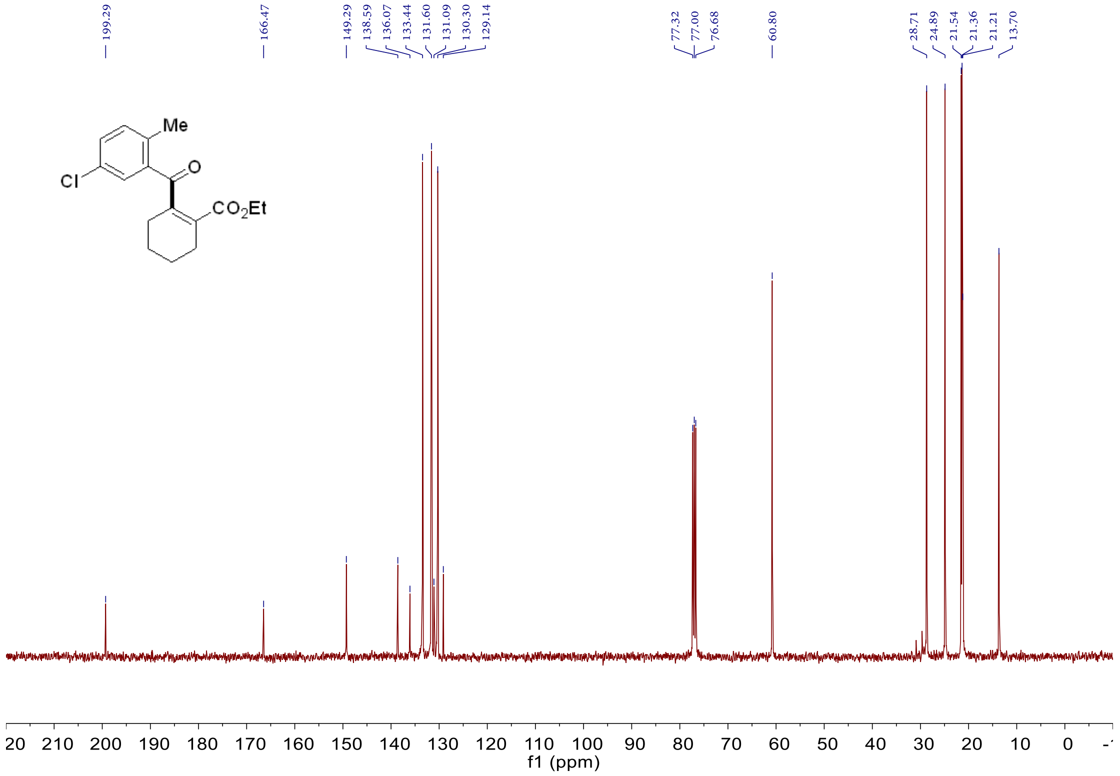

Supplementary Figure 131. ^{13}C NMR spectrum for compound **3u**

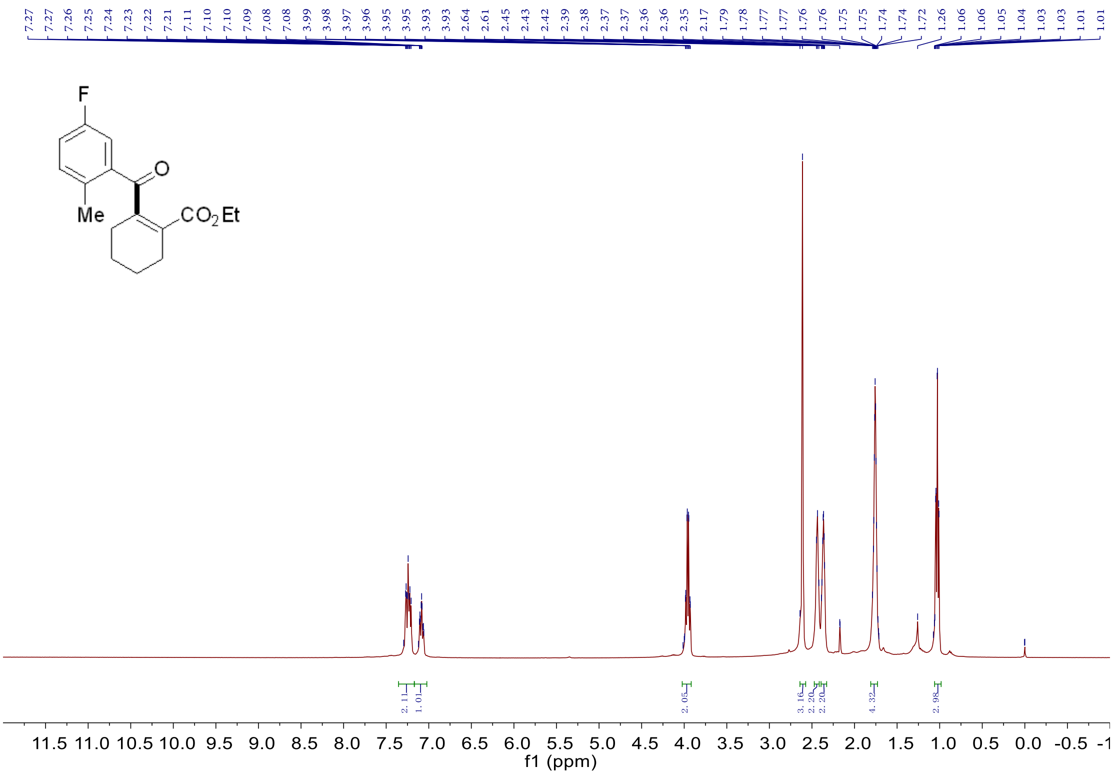

Supplementary Figure 132. ^{1}H NMR spectrum for compound **3v**

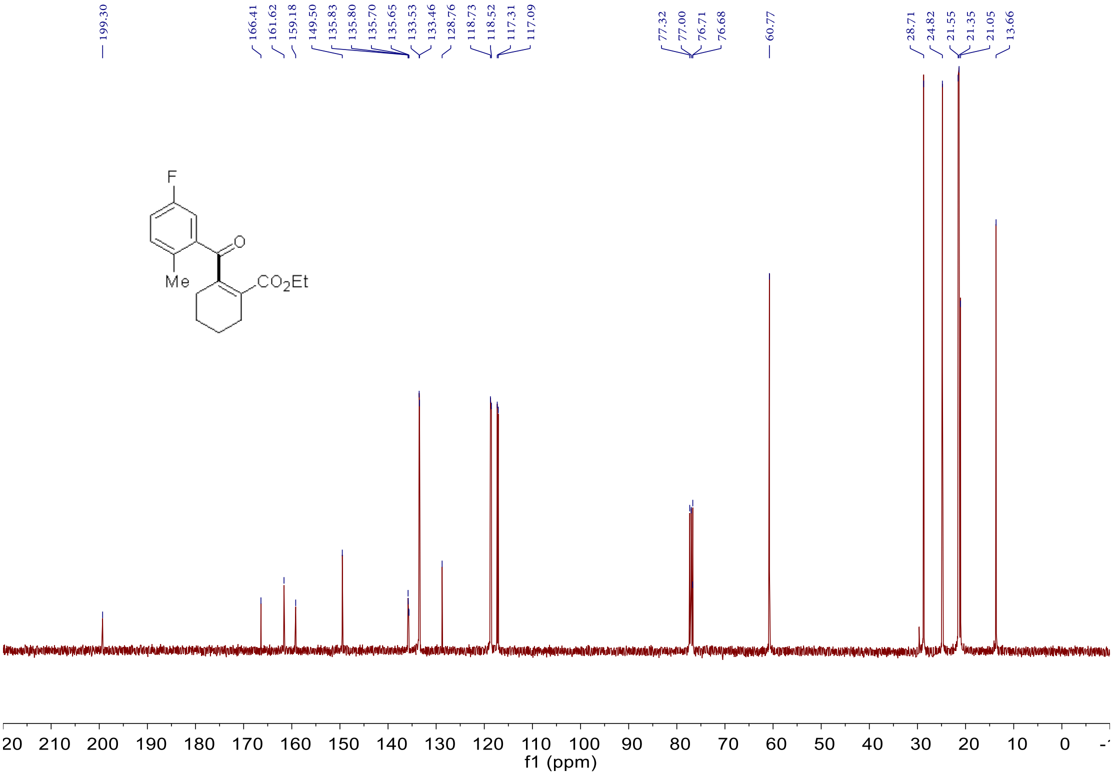

Supplementary Figure 133. ^{13}C NMR spectrum for compound **3v**

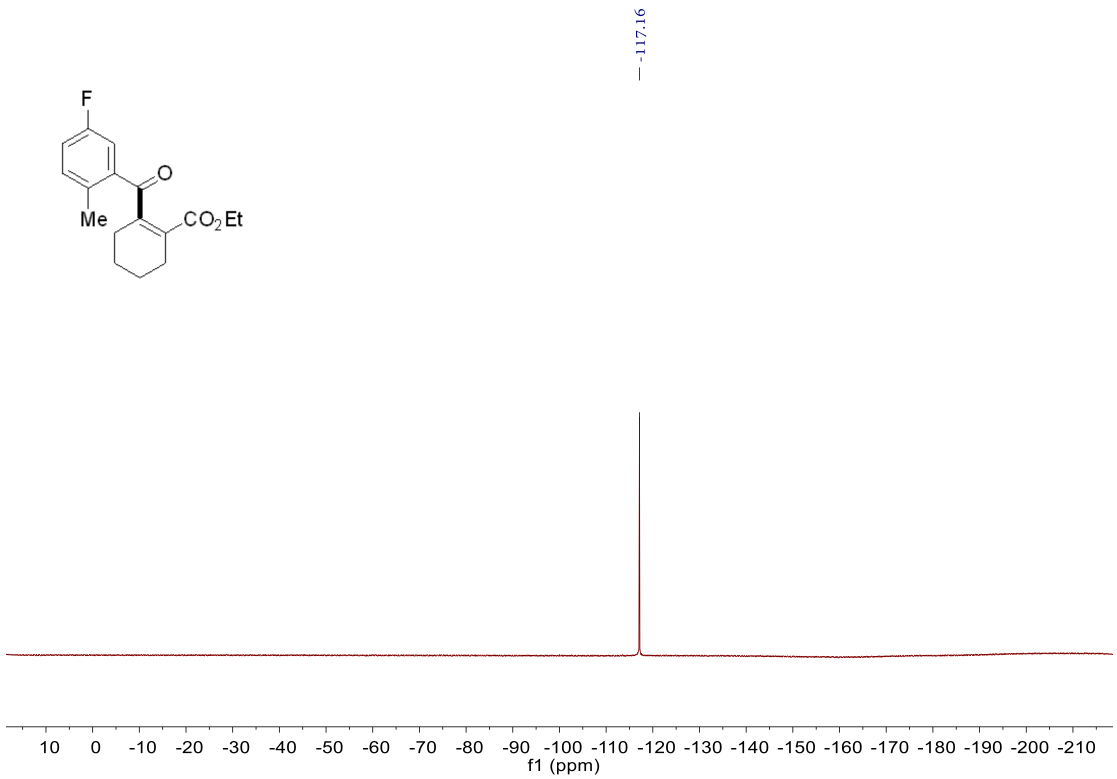

Supplementary Figure 134. ^{19}F NMR spectrum for compound **3v**

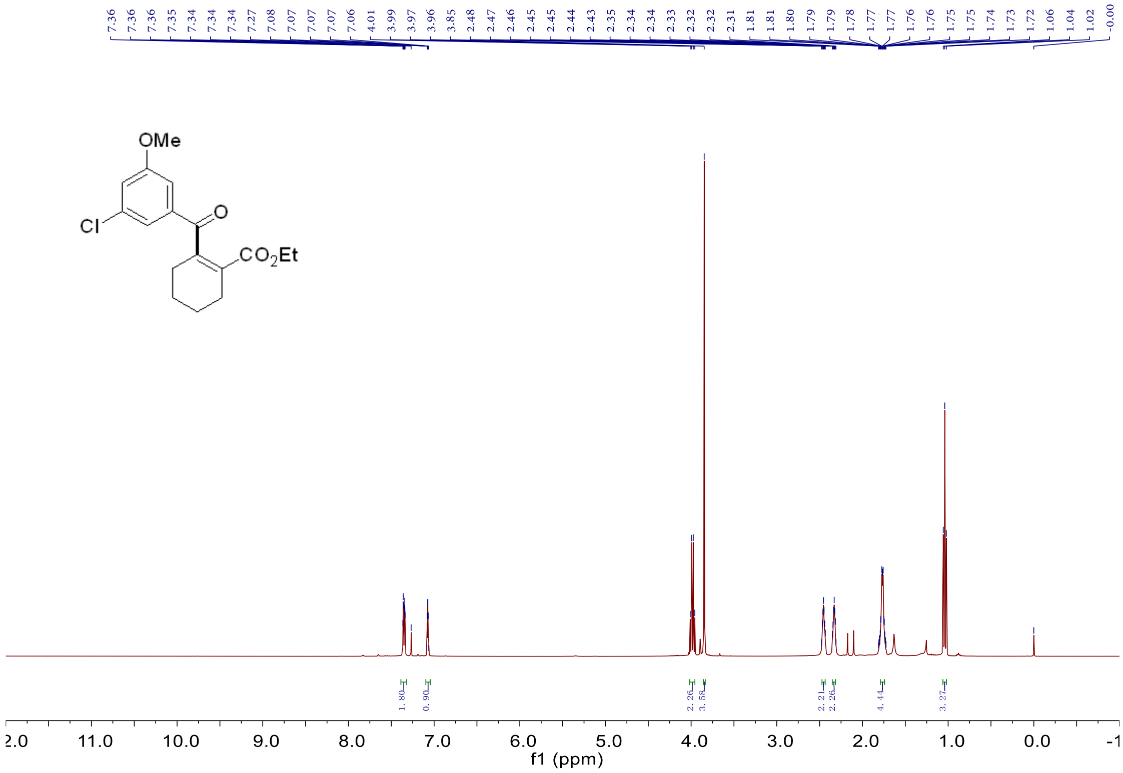

Supplementary Figure 135. ^{1}H NMR spectrum for compound **3w**

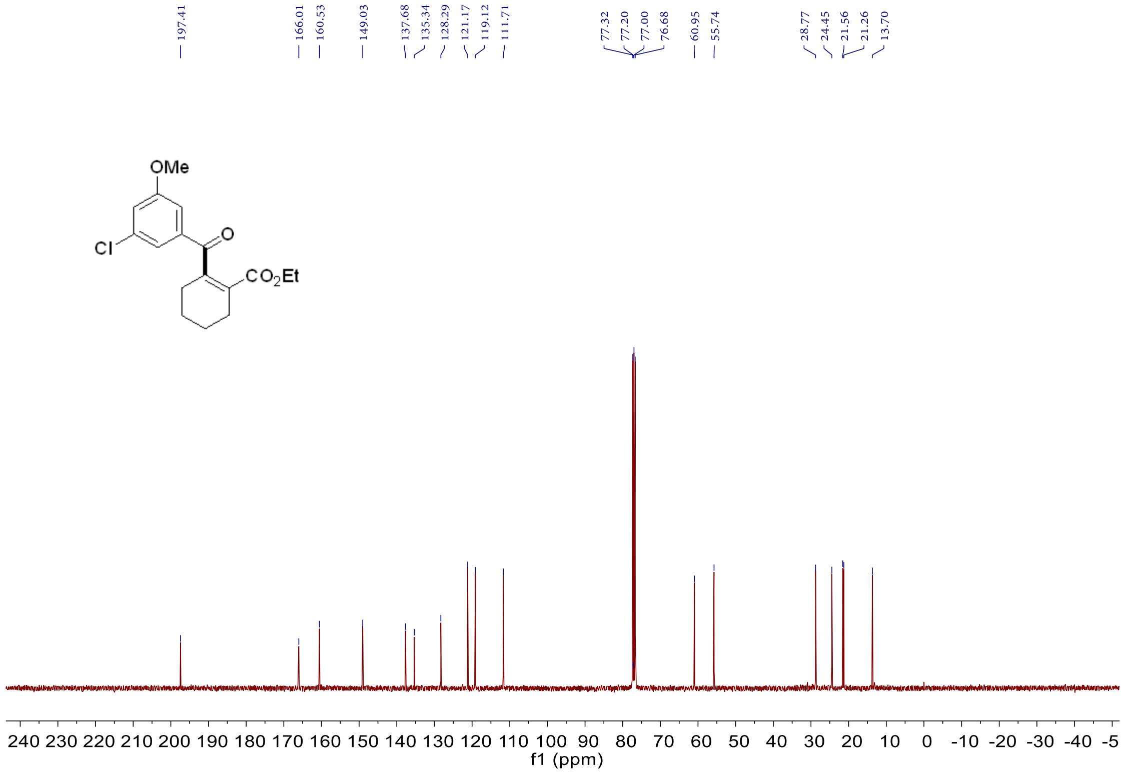

Supplementary Figure 136. ^{13}C NMR spectrum for compound **3w**

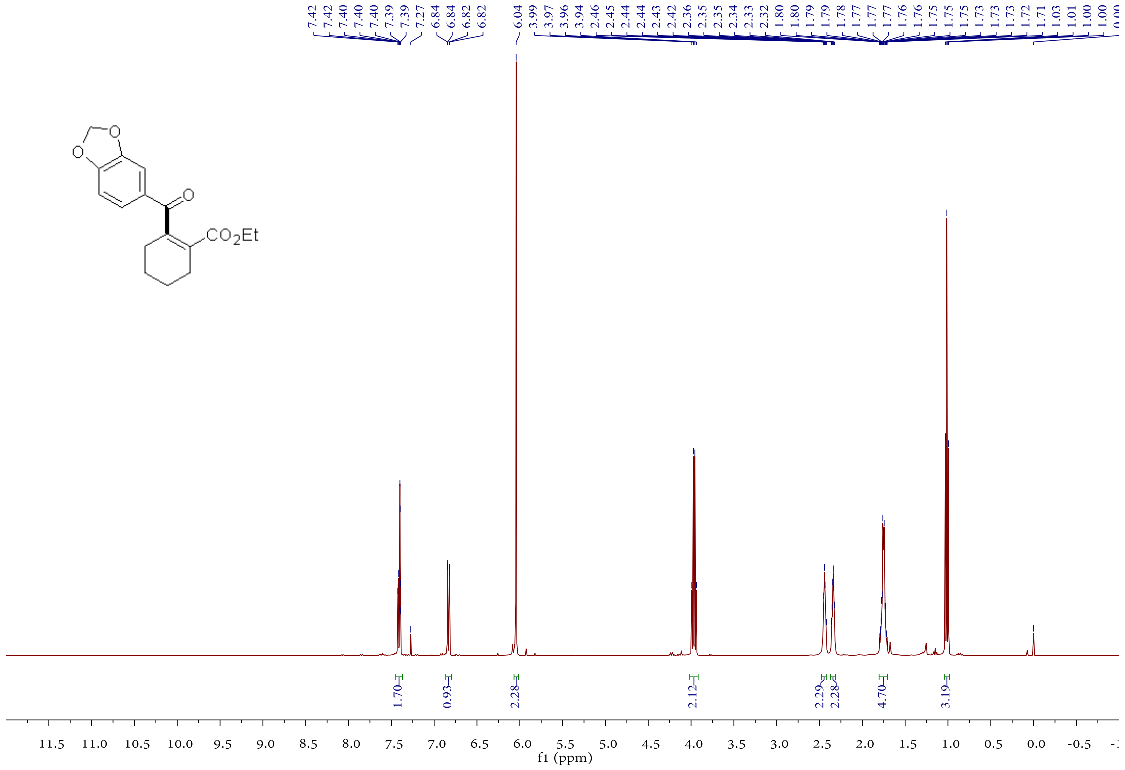

Supplementary Figure 137. ^{1}H NMR spectrum for compound **3x**

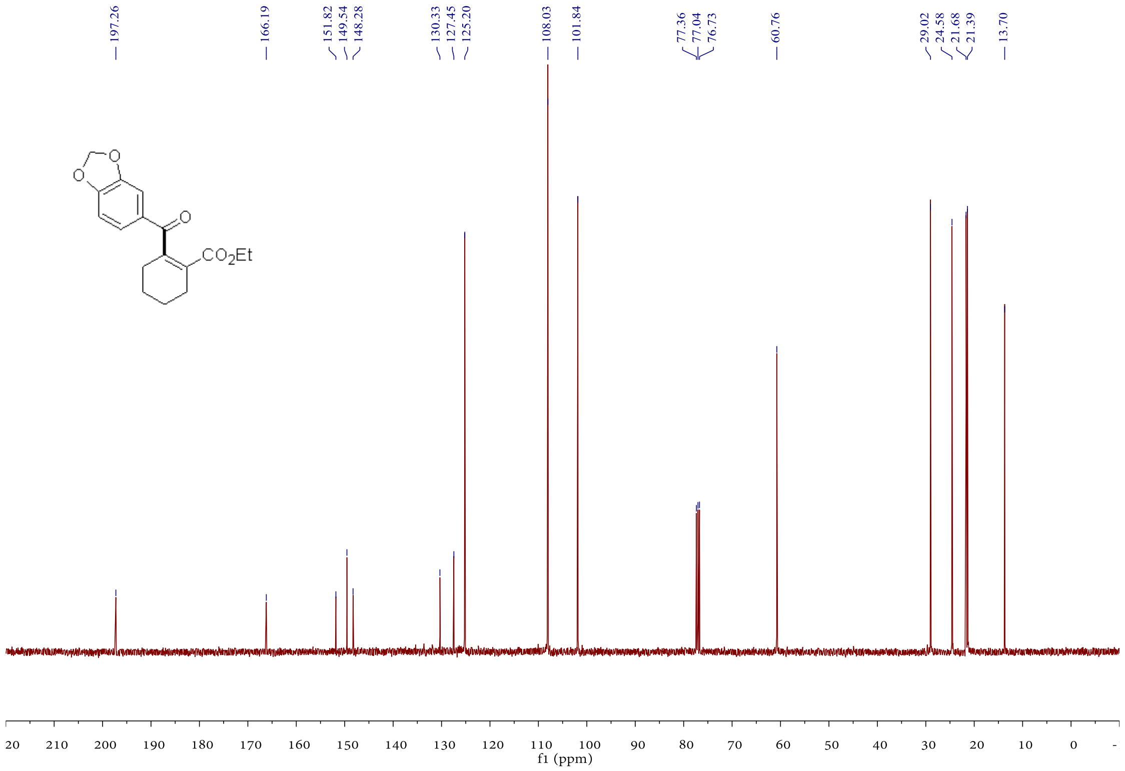

Supplementary Figure 138. ^{13}C NMR spectrum for compound **3x**

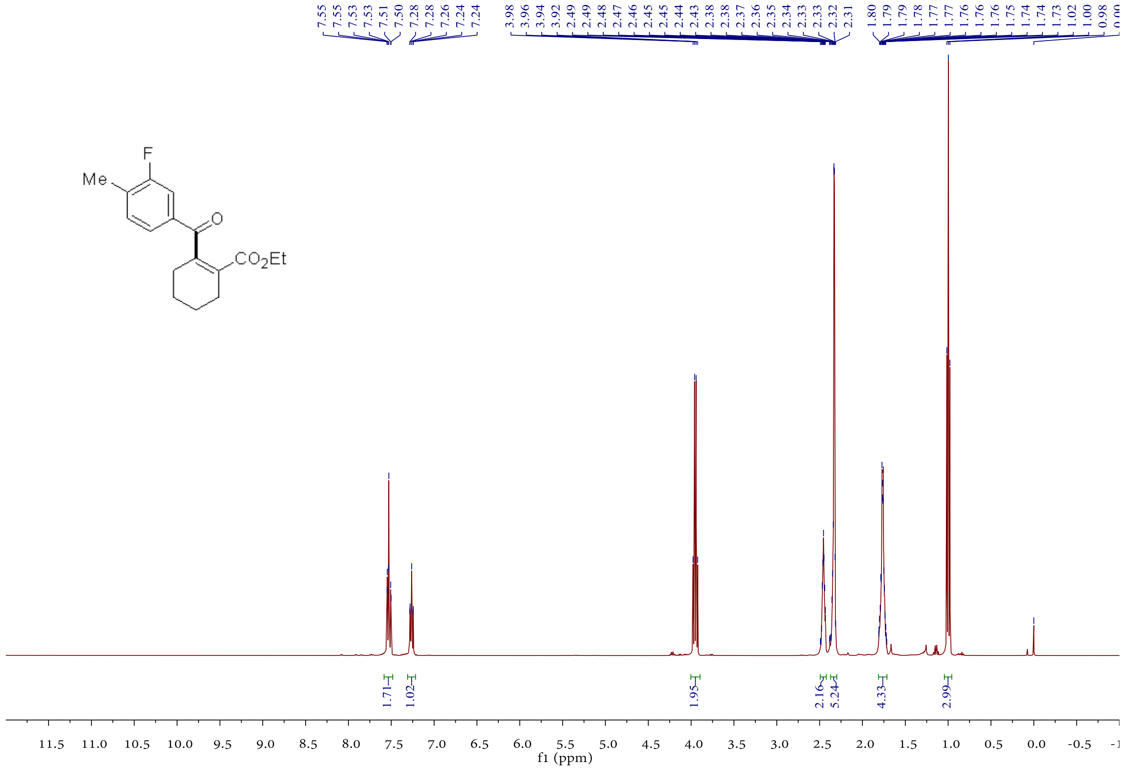

Supplementary Figure 139. ^{1}H NMR spectrum for compound **3y**

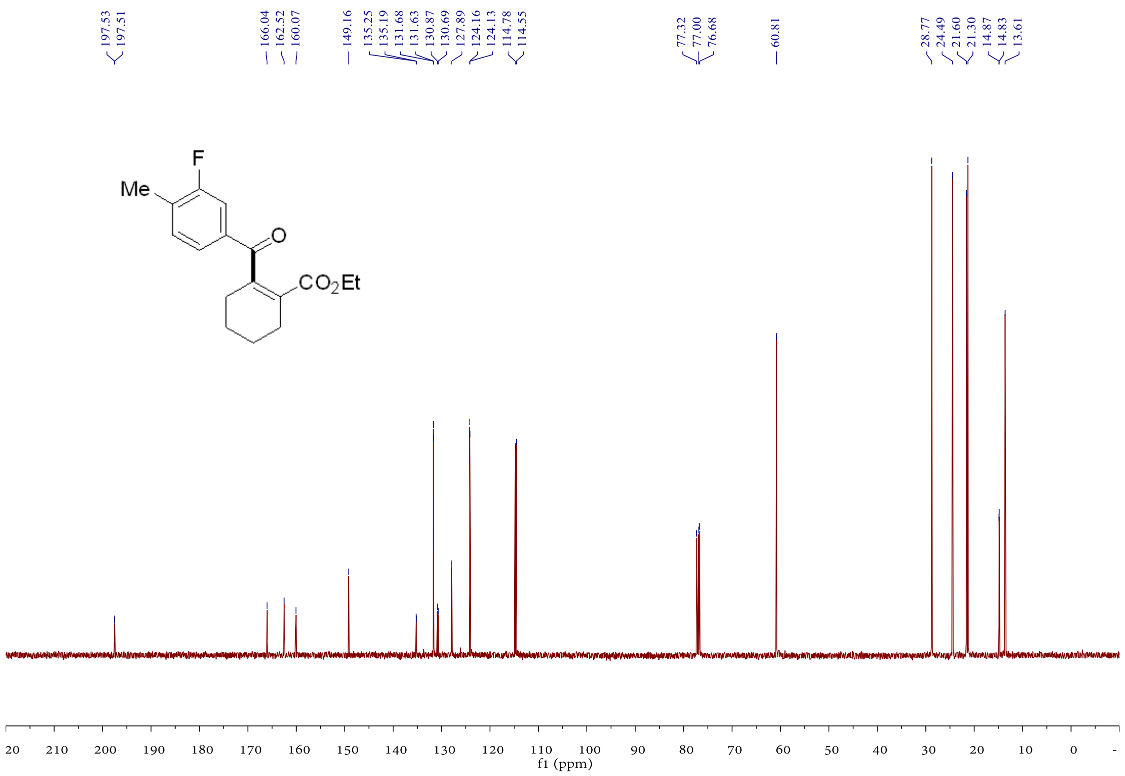

Supplementary Figure 140. ^{13}C NMR spectrum for compound **3y**

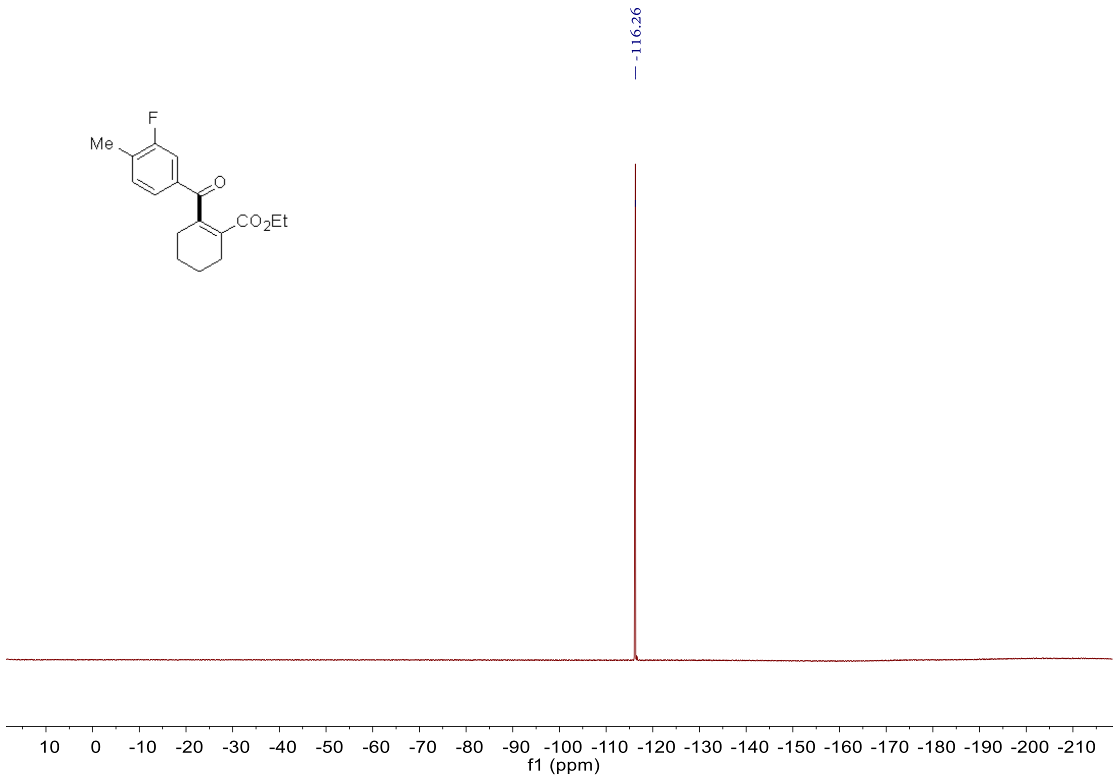

Supplementary Figure 141. ^{19}F NMR spectrum for compound **3y**

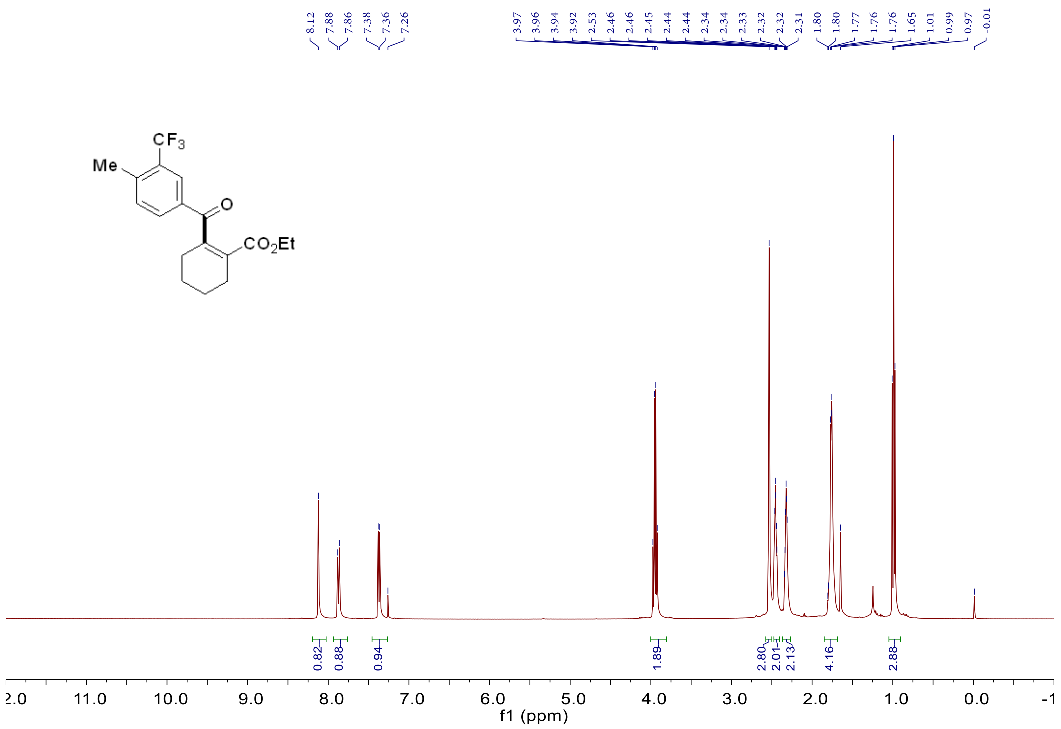

Supplementary Figure 142. ^{1}H NMR spectrum for compound **3z**

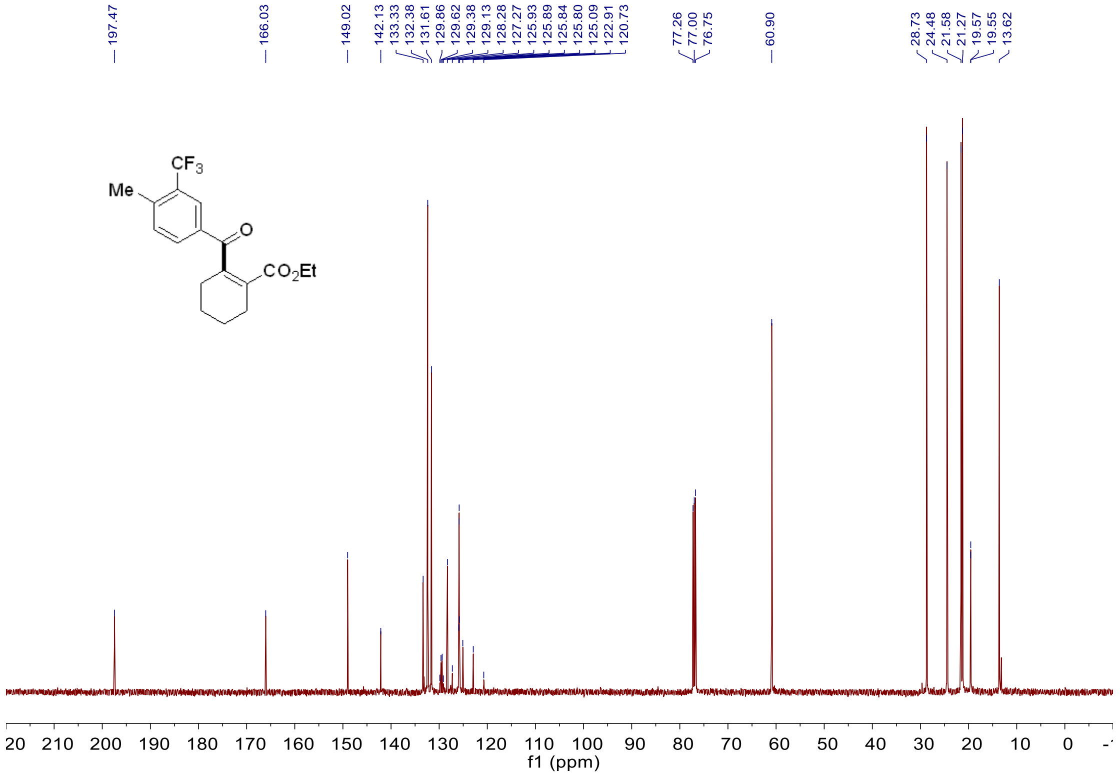

Supplementary Figure 143. ^{13}C NMR spectrum for compound **3z**

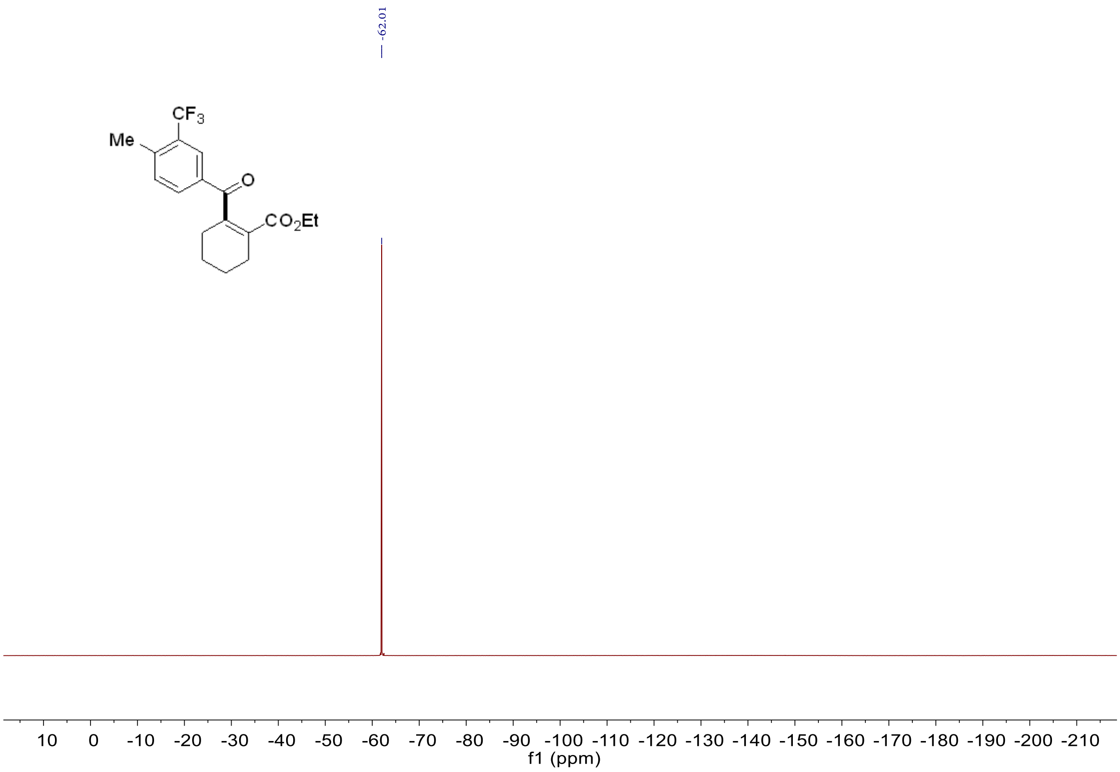

Supplementary Figure 144. ^{19}F NMR spectrum for compound **3z**

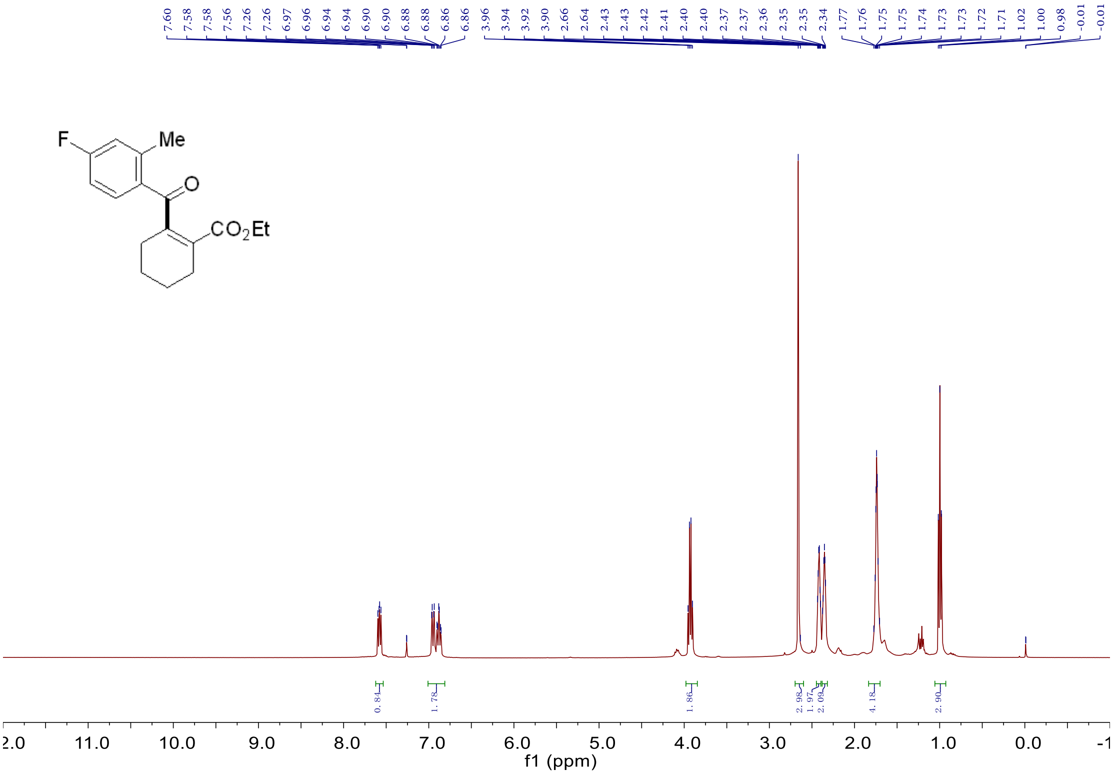

Supplementary Figure 145. ^{1}H NMR spectrum for compound **3aa**

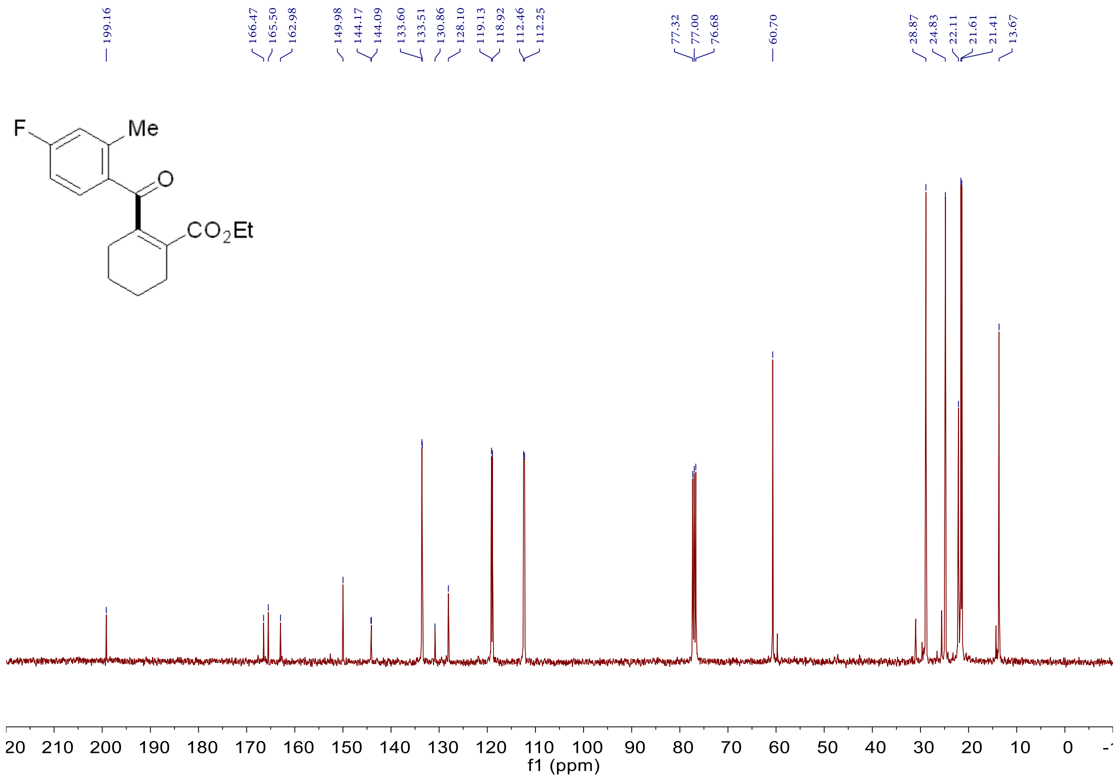

Supplementary Figure 146. ^{13}C NMR spectrum for compound **3aa**

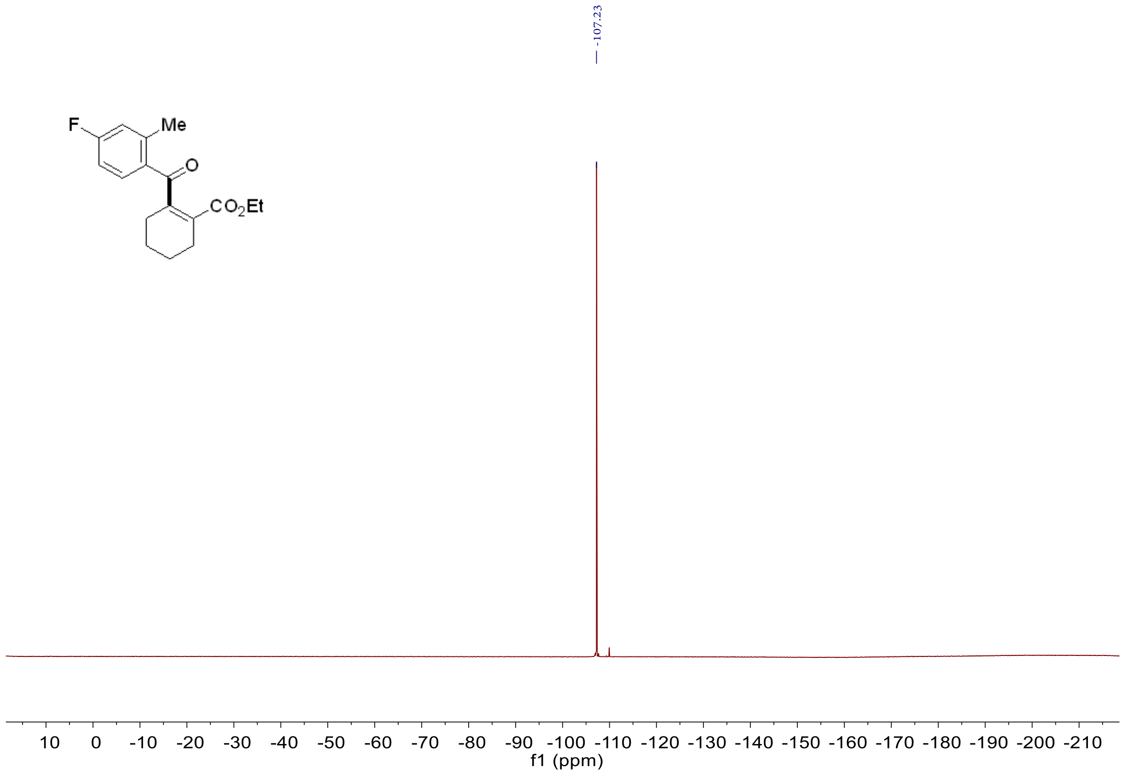

Supplementary Figure 147. ^{19}F NMR spectrum for compound **3aa**

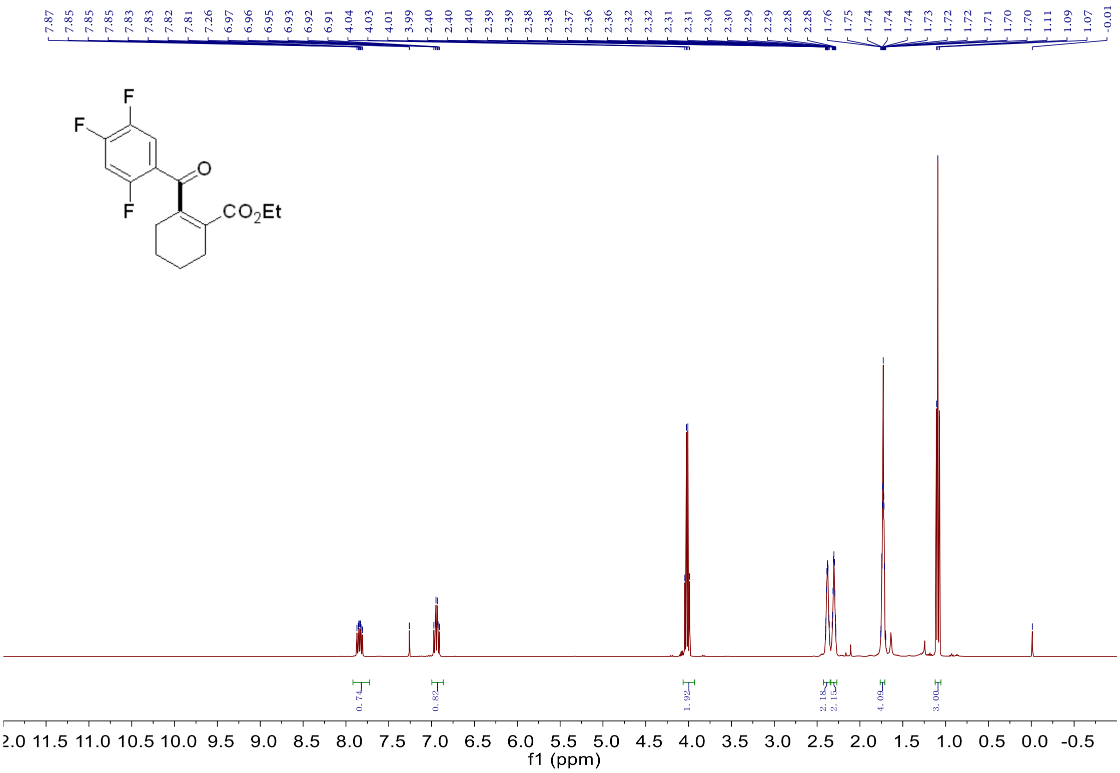

Supplementary Figure 148. ^{1}H NMR spectrum for compound **3bb**

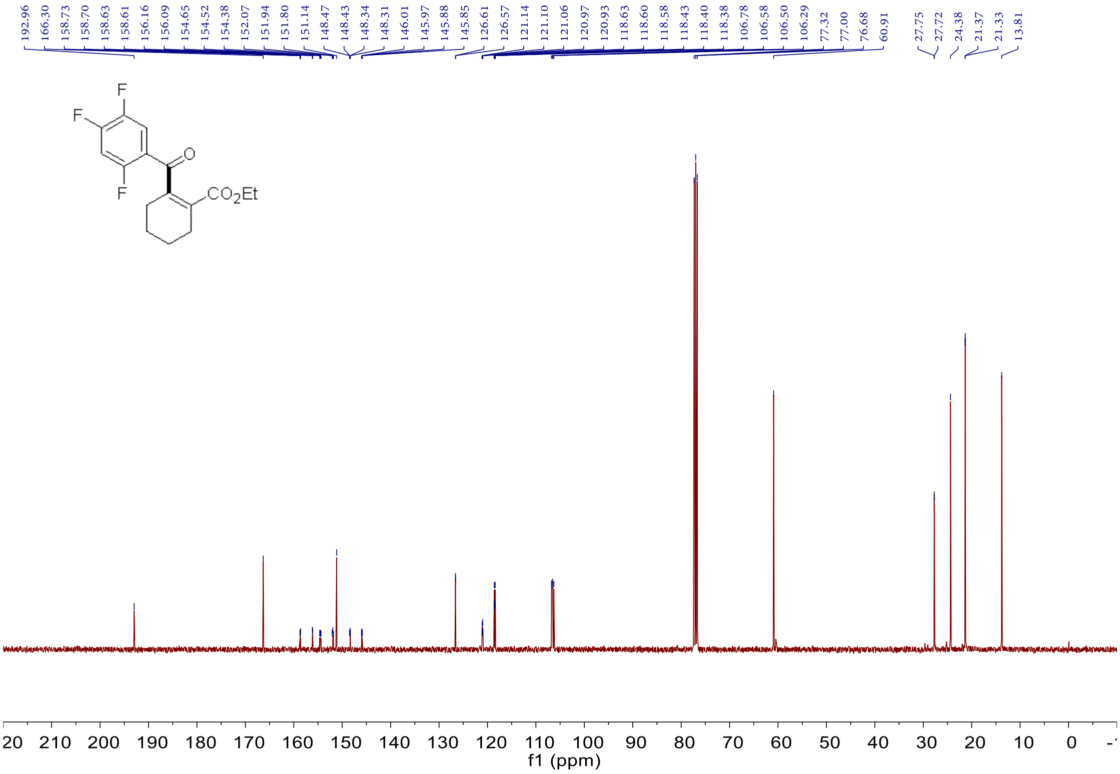

Supplementary Figure 149. ^{13}C NMR spectrum for compound **3bb**

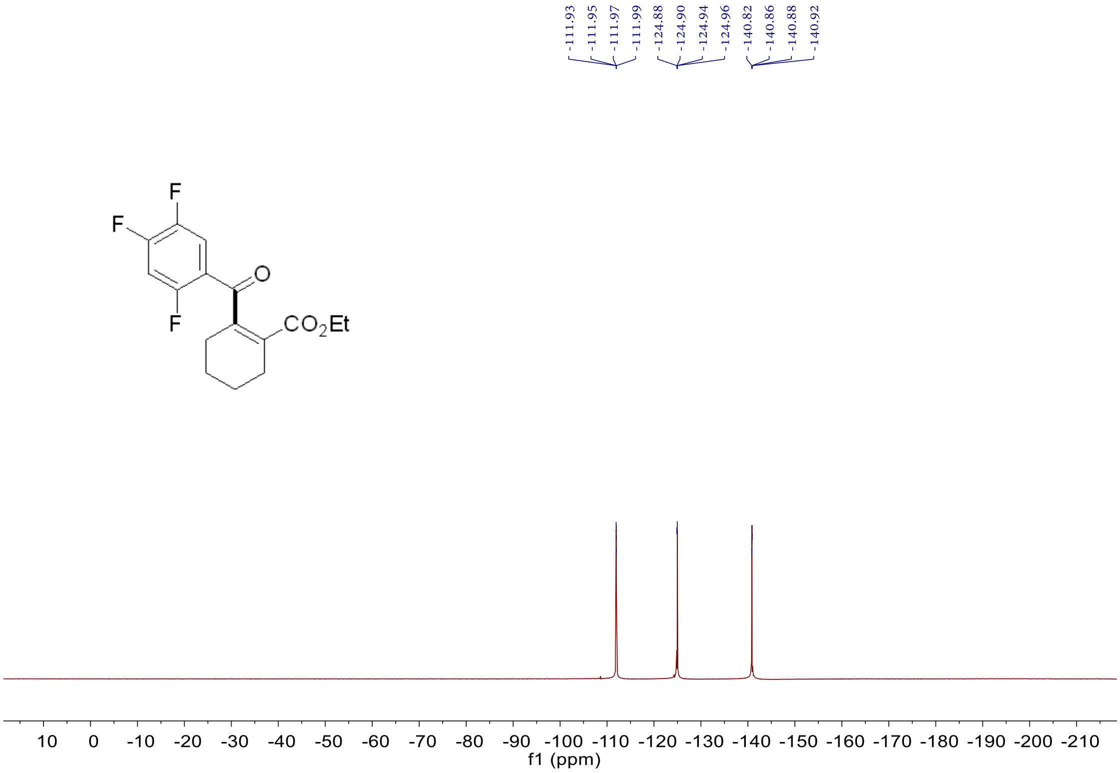

Supplementary Figure 150. ^{19}F NMR spectrum for compound **3bb**

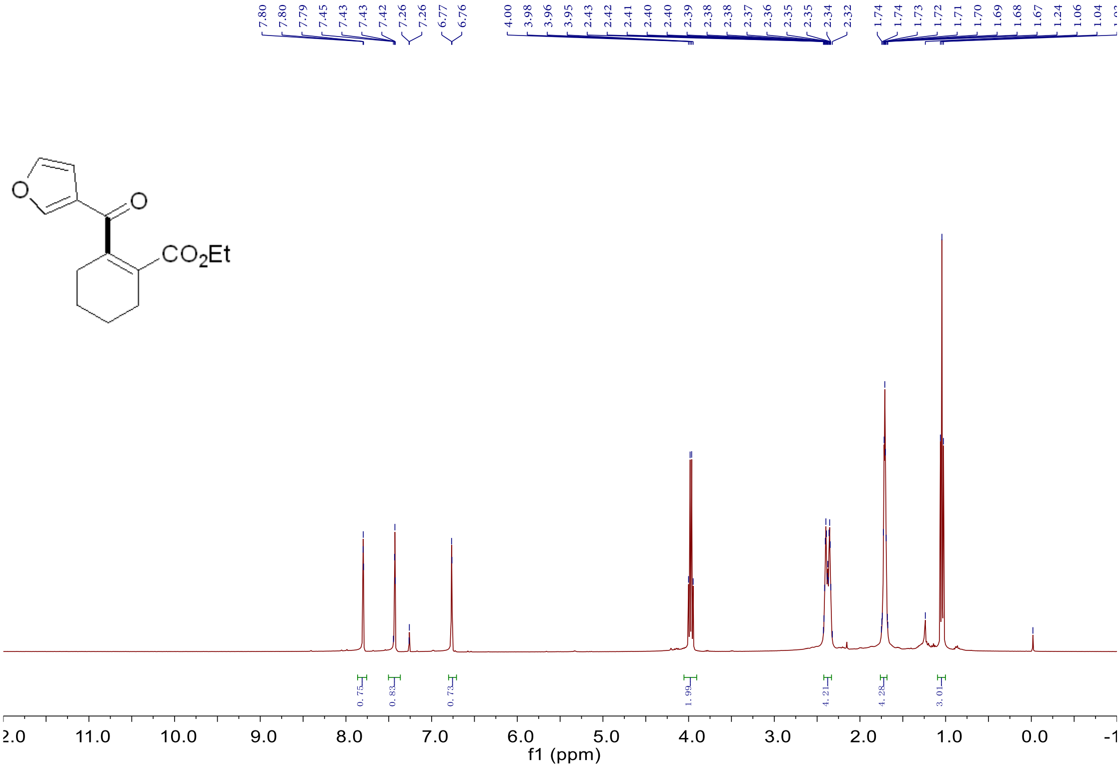

Supplementary Figure 151. ^{1}H NMR spectrum for compound **3cc**

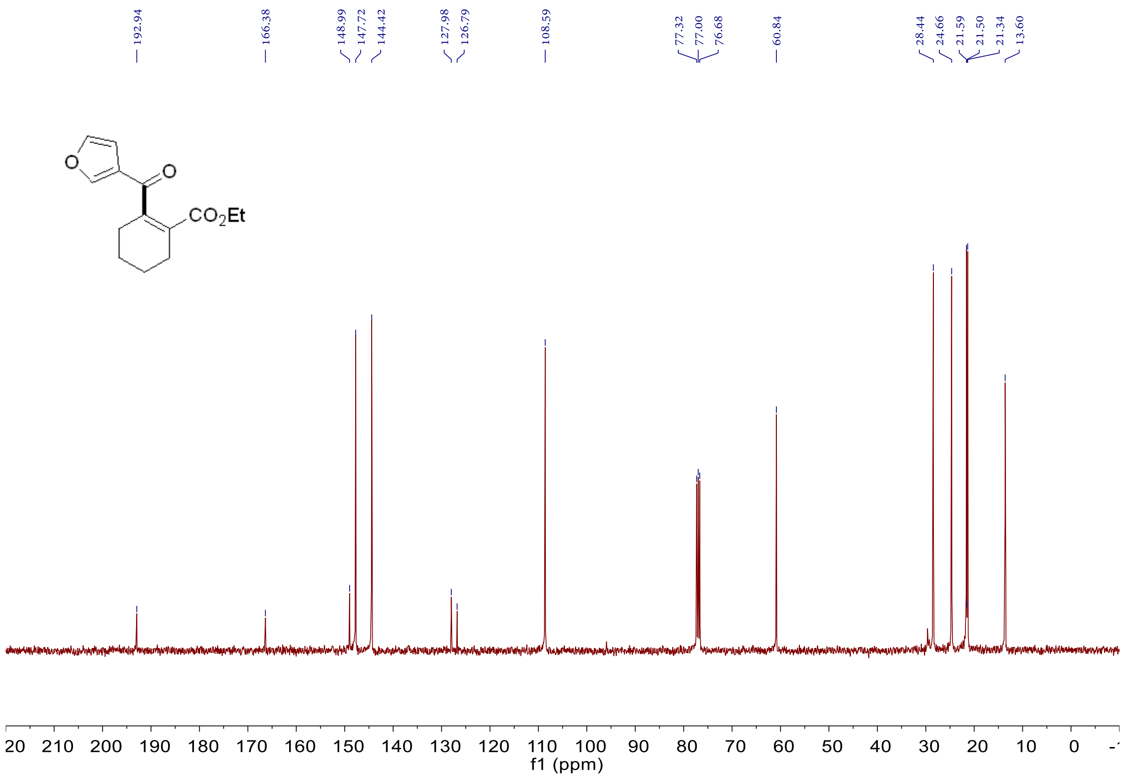

Supplementary Figure 152. ^{13}C NMR spectrum for compound **3cc**

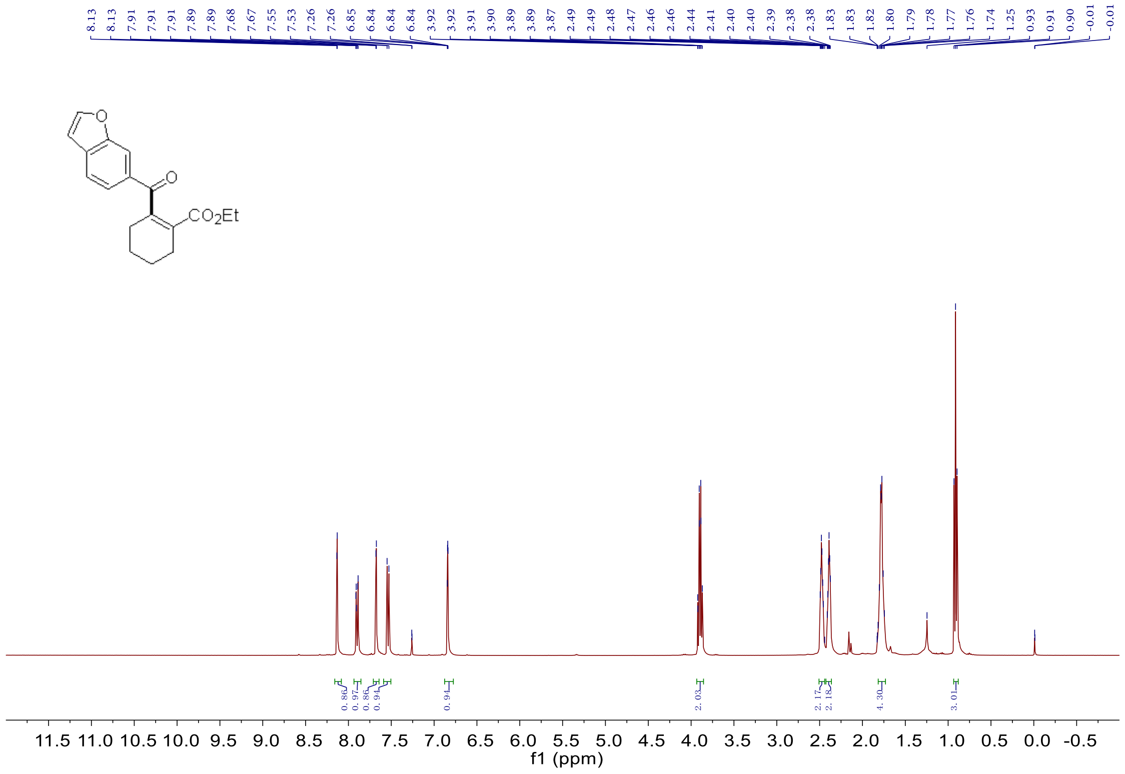

Supplementary Figure 153. ^{1}H NMR spectrum for compound **3dd**

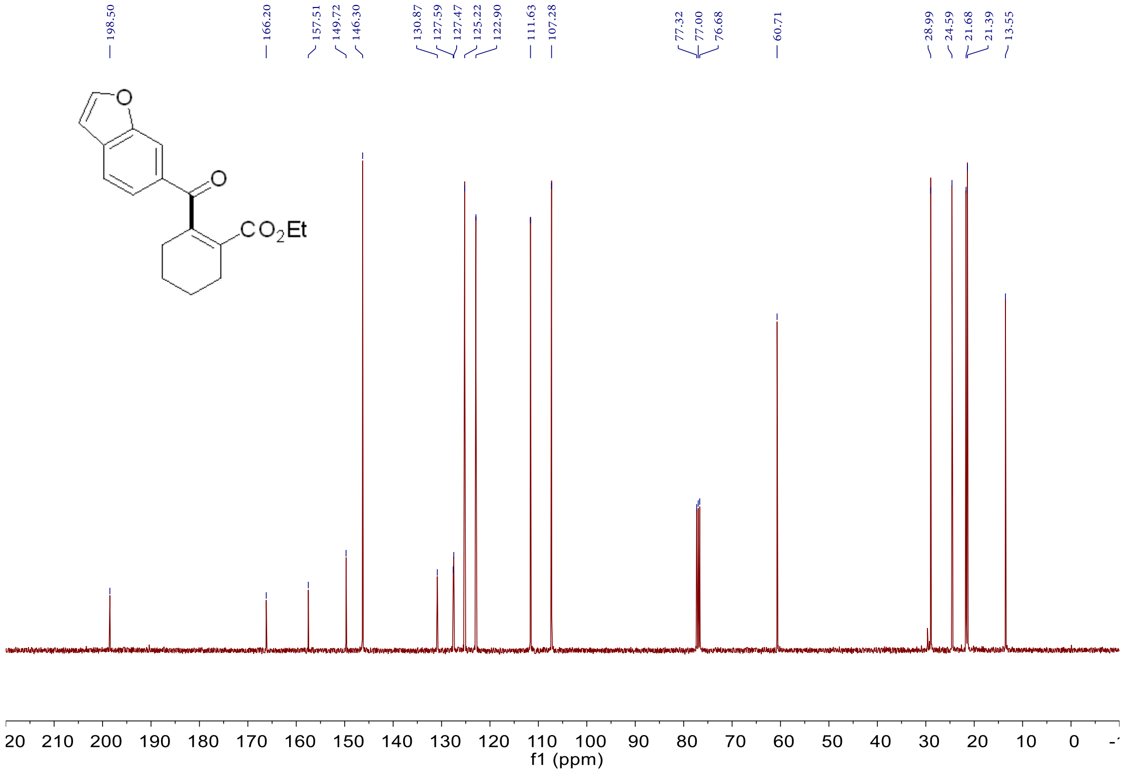

Supplementary Figure 154. ^{13}C NMR spectrum for compound **3dd**

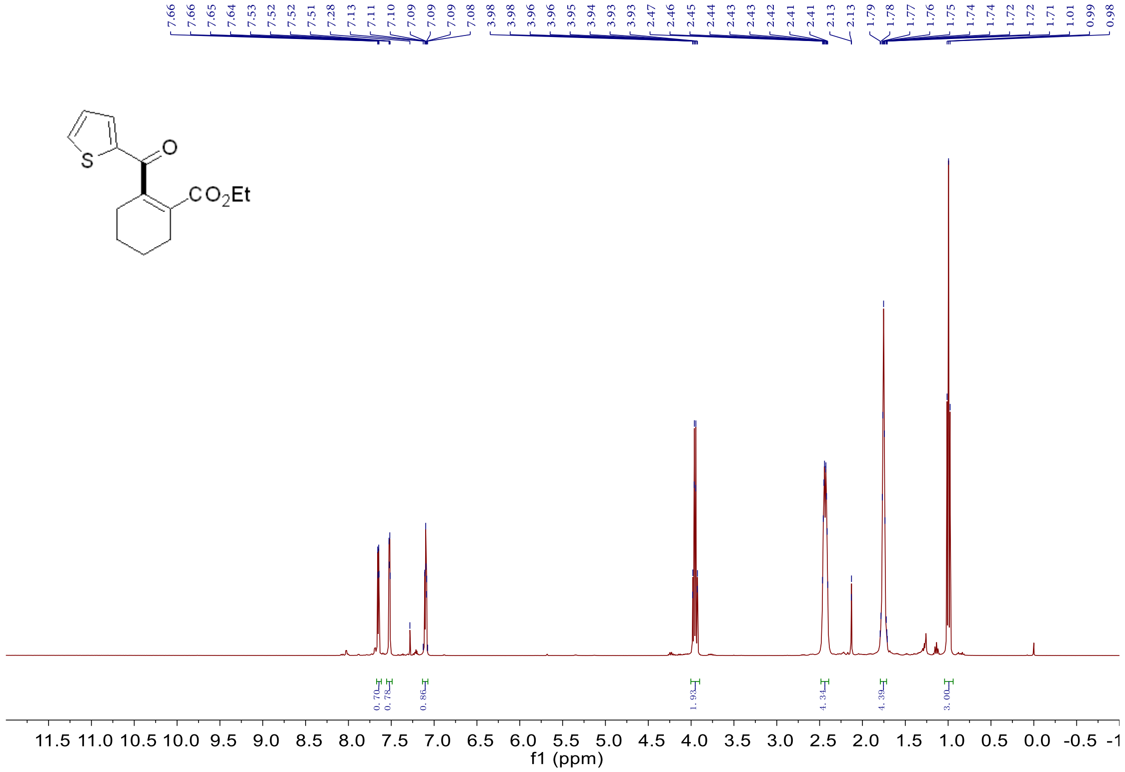

Supplementary Figure 155. ^{1}H NMR spectrum for compound **3ee**

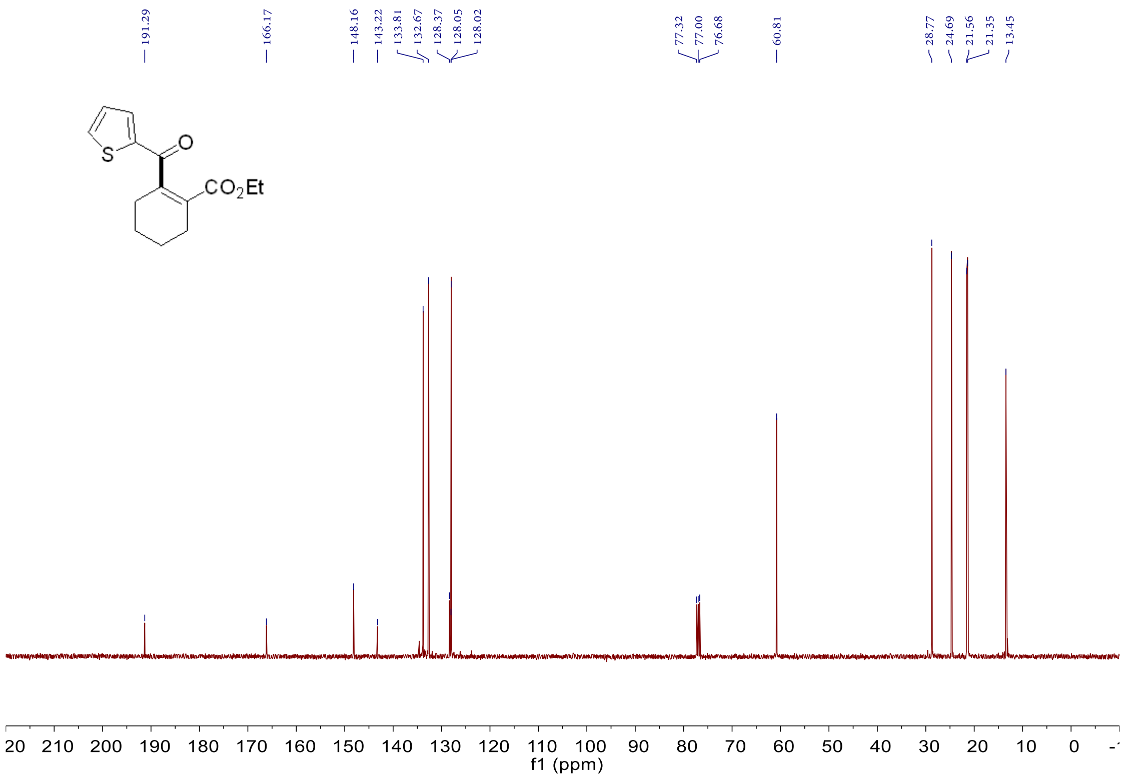

Supplementary Figure 156. ^{13}C NMR spectrum for compound **3ee**

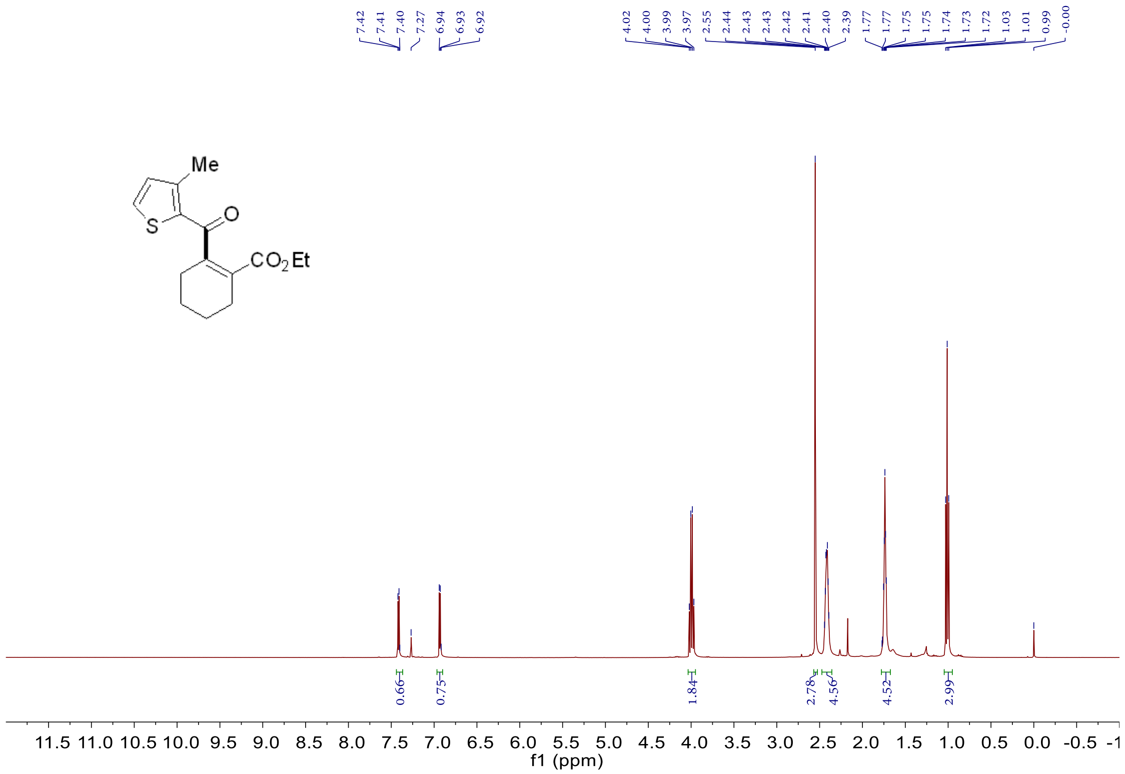

Supplementary Figure 157. ^{1}H NMR spectrum for compound **3ff**

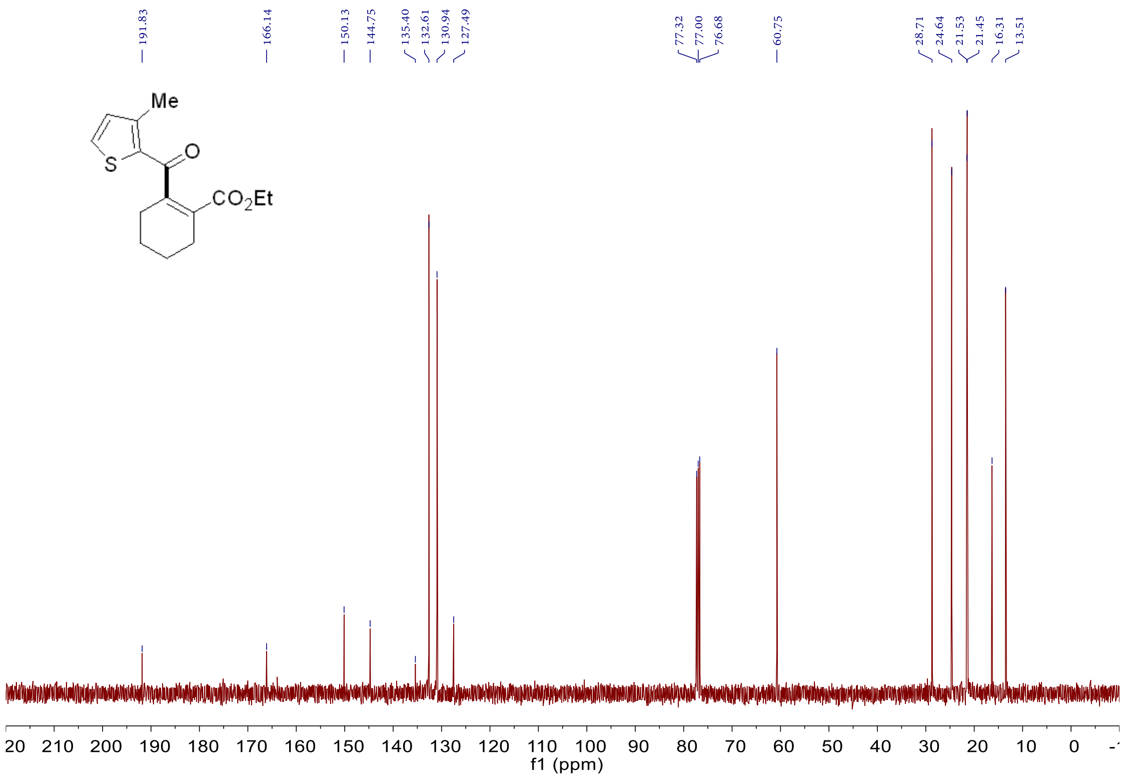

Supplementary Figure 158. ^{13}C NMR spectrum for compound **3ff**

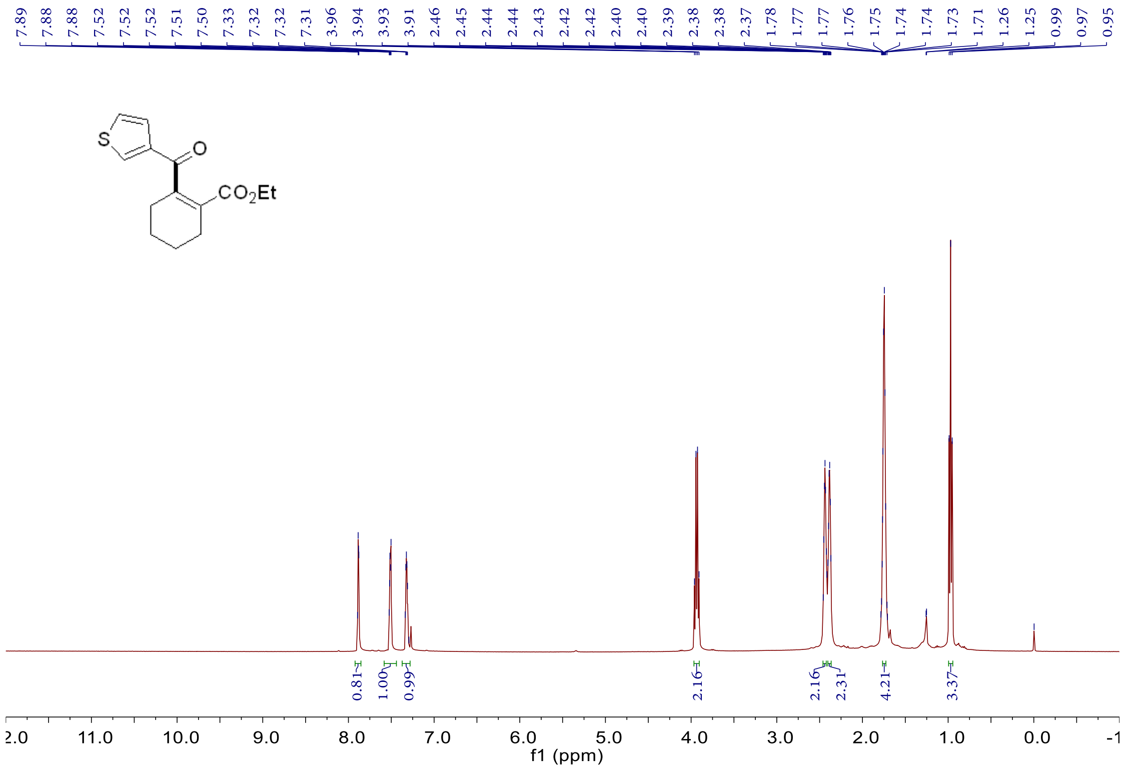

Supplementary Figure 159. ^{1}H NMR spectrum for compound **3gg**

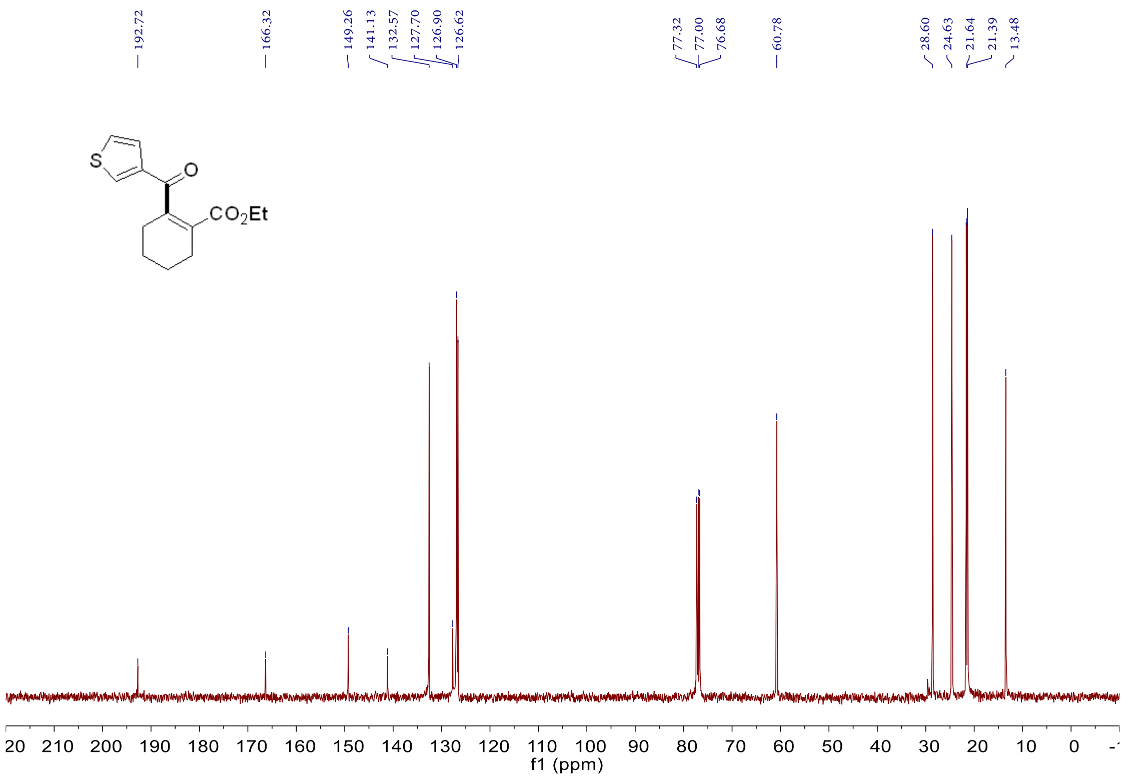

Supplementary Figure 160. ^{13}C NMR spectrum for compound **3gg**

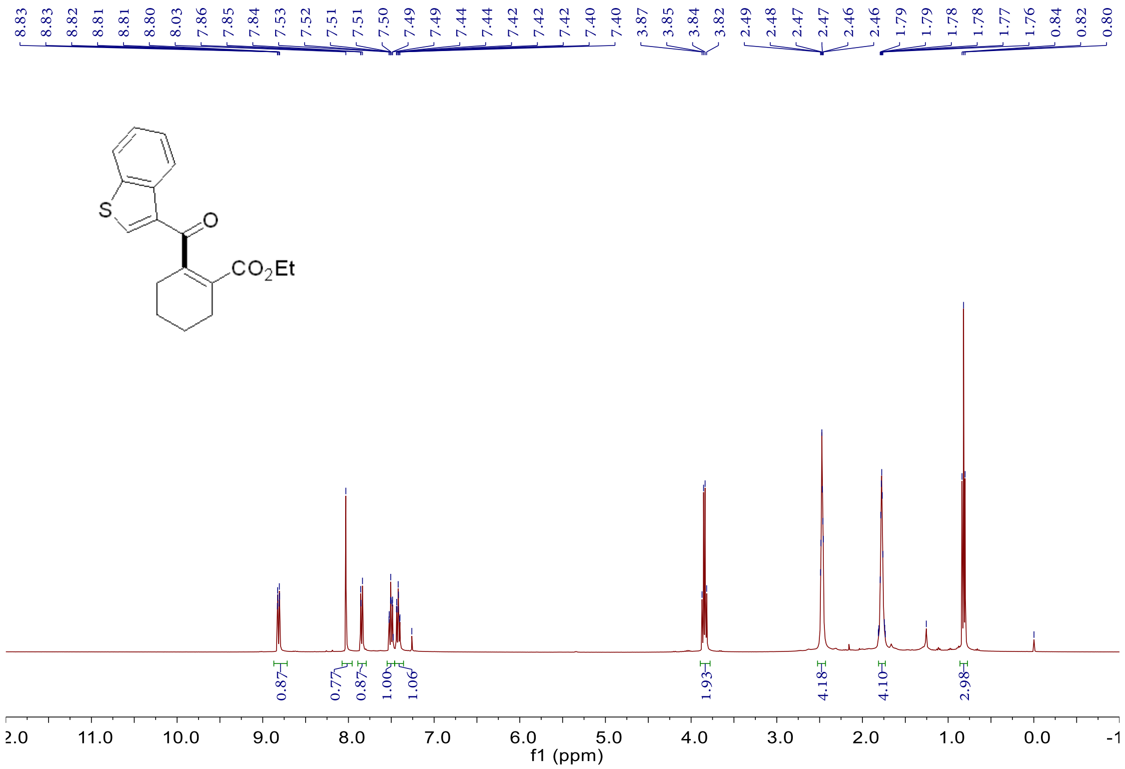

Supplementary Figure 161. ^{1}H NMR spectrum for compound **3hh**

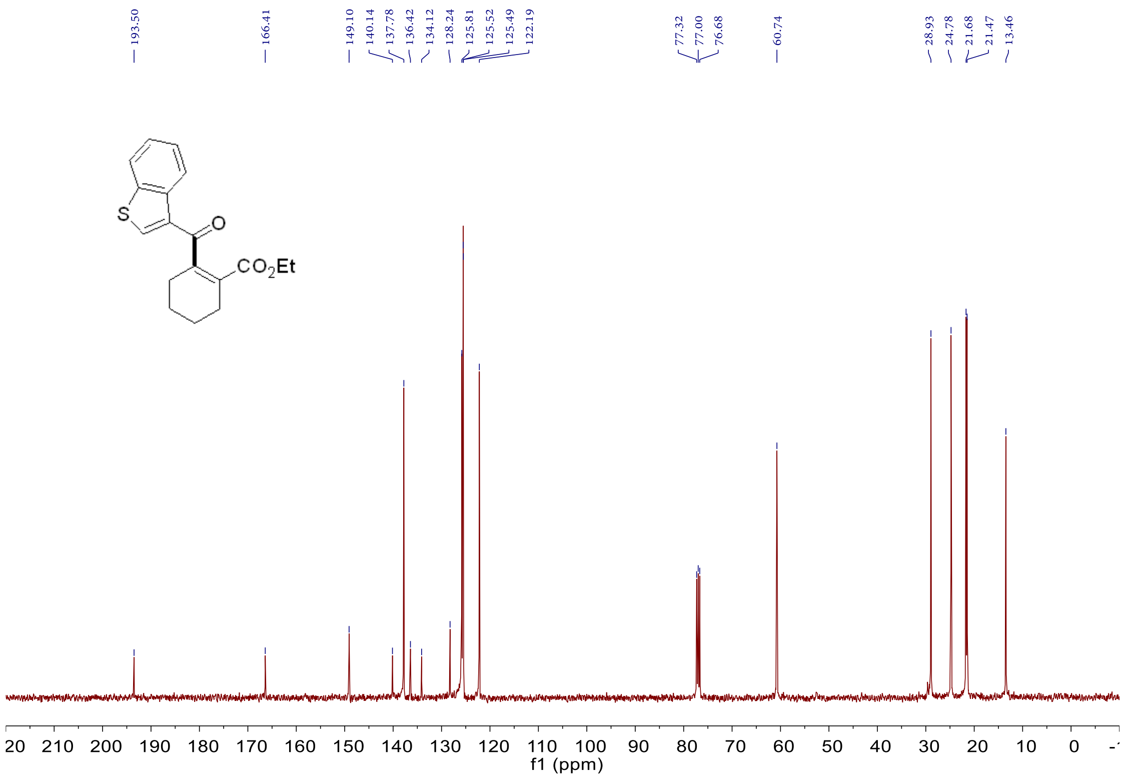

Supplementary Figure 162. ^{13}C NMR spectrum for compound **3hh**

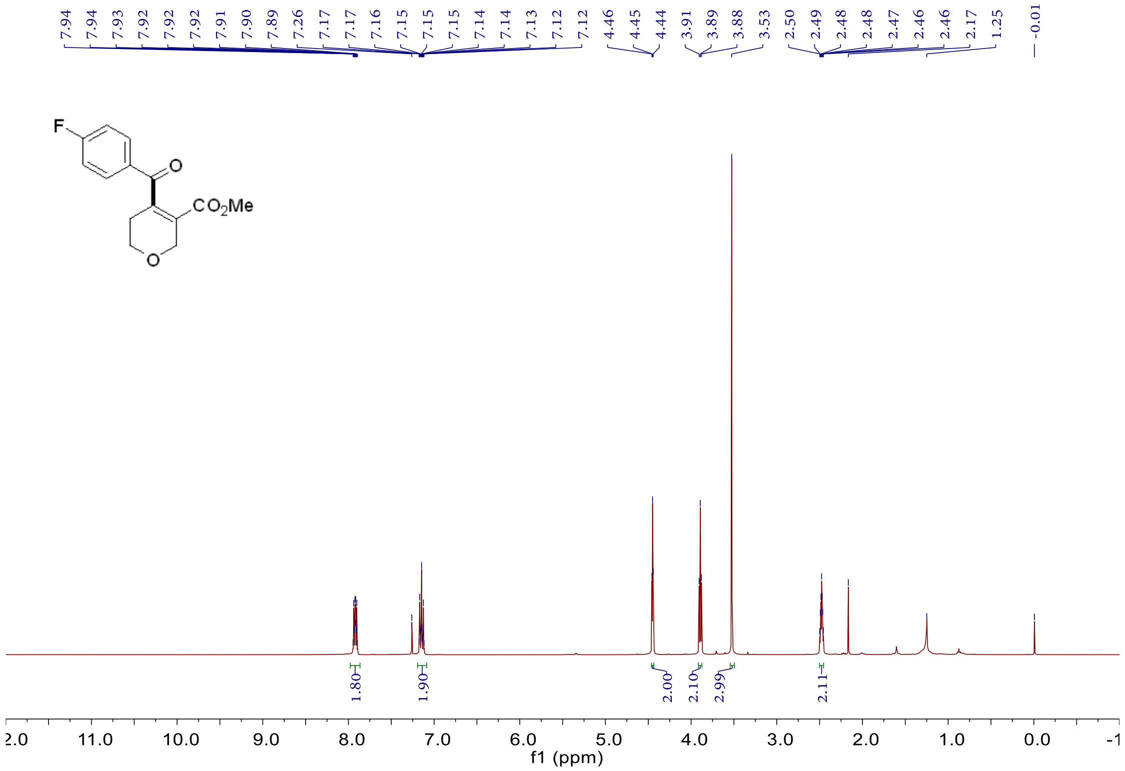

Supplementary Figure 163. ^{1}H NMR spectrum for compound **3ii**

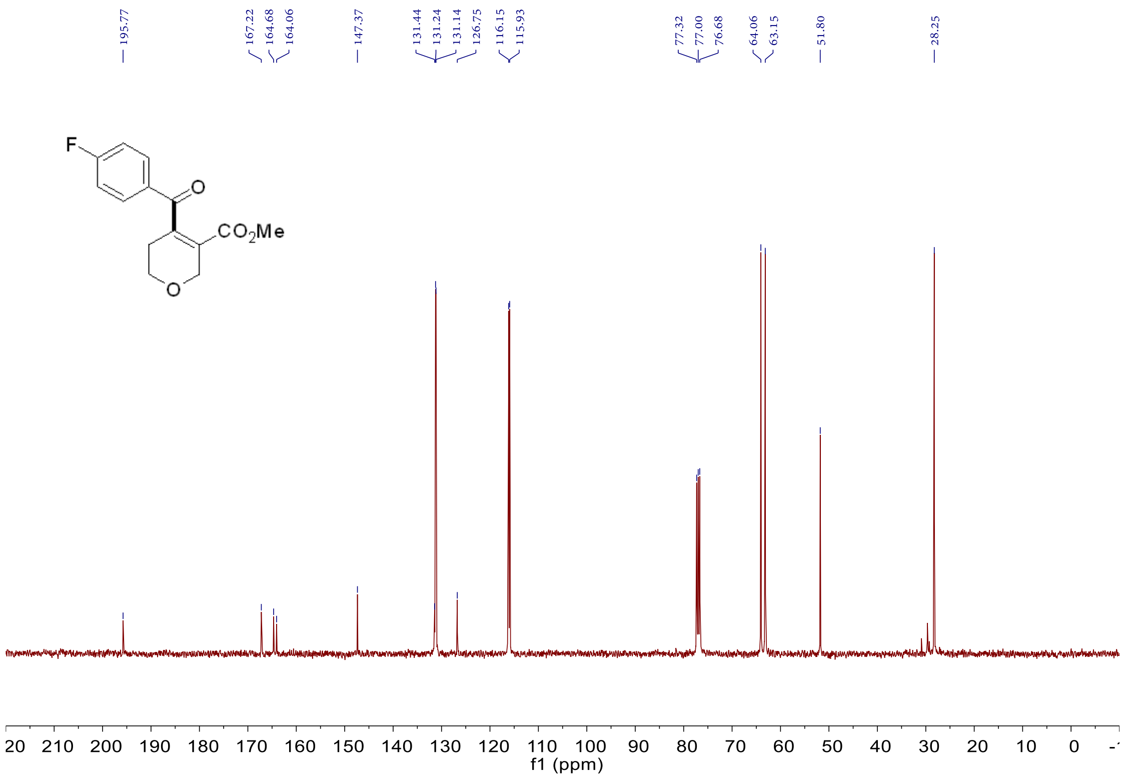

Supplementary Figure 164. ^{13}C NMR spectrum for compound **3ii**

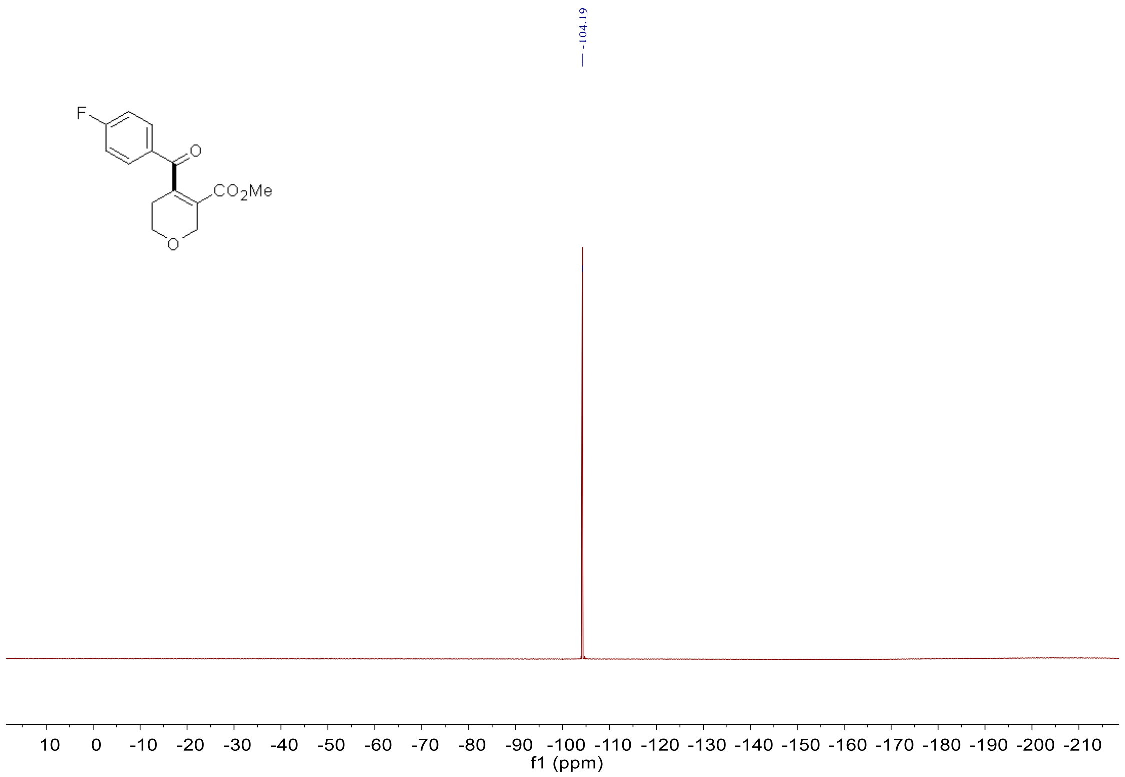

Supplementary Figure 165. ^{19}F NMR spectrum for compound **3ii**

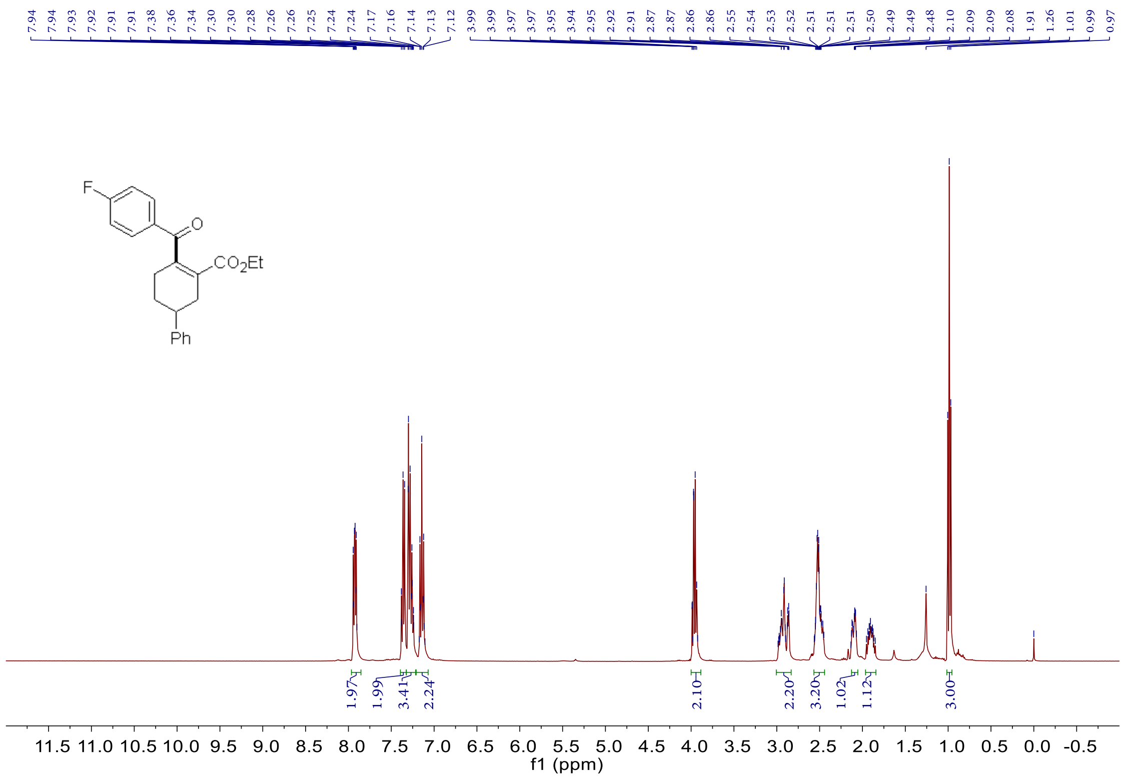

Supplementary Figure 166. ^{1}H NMR spectrum for compound **3jj**

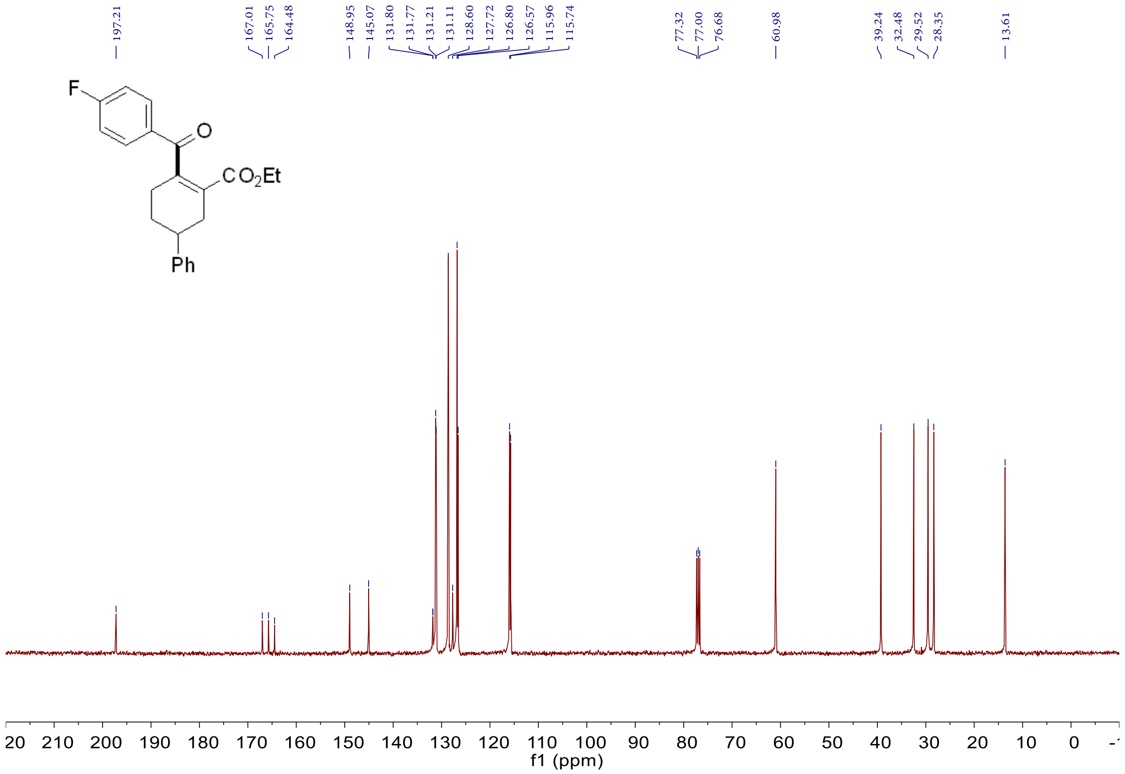

Supplementary Figure 167. ^{13}C NMR spectrum for compound **3jj**

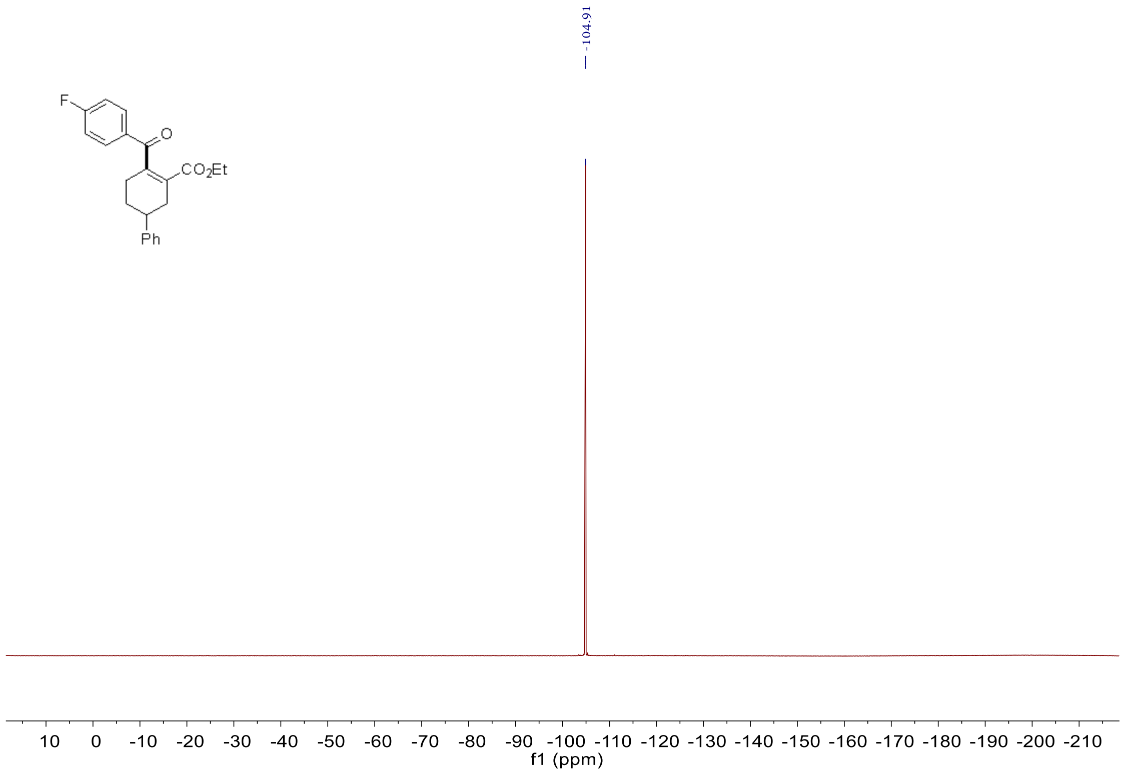

Supplementary Figure 168. ^{19}F NMR spectrum for compound **3jj**

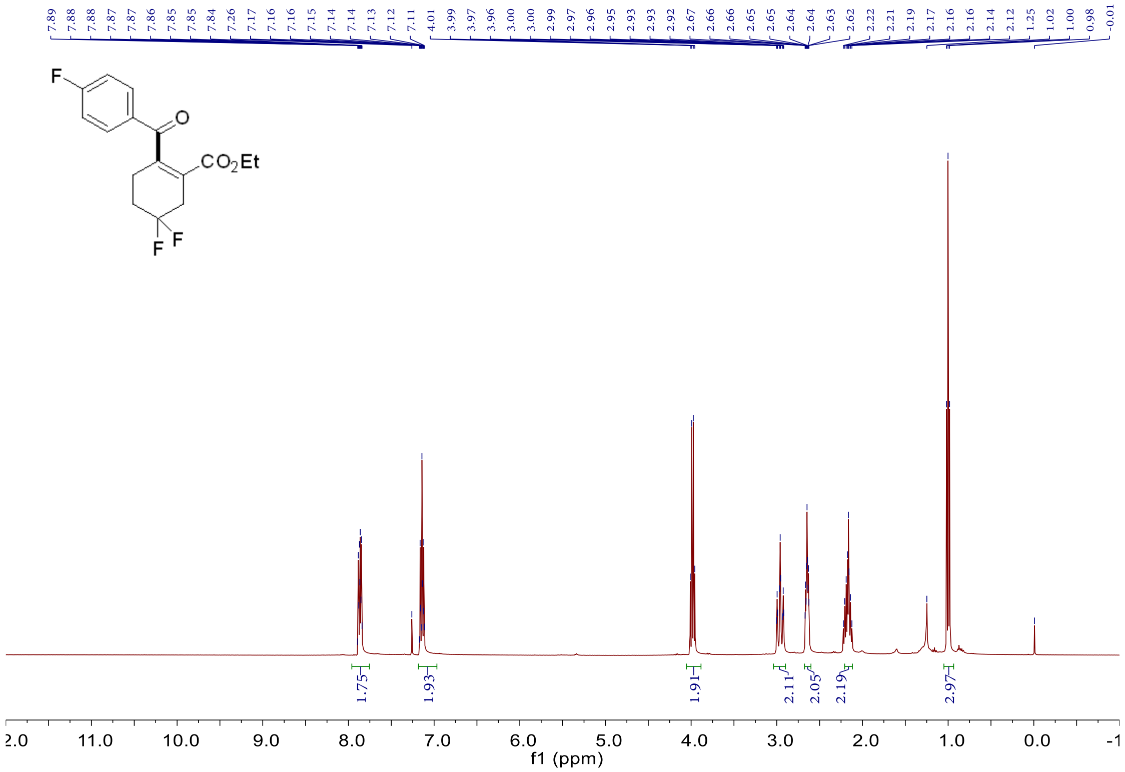

Supplementary Figure 169. ^{1}H NMR spectrum for compound **3kk**

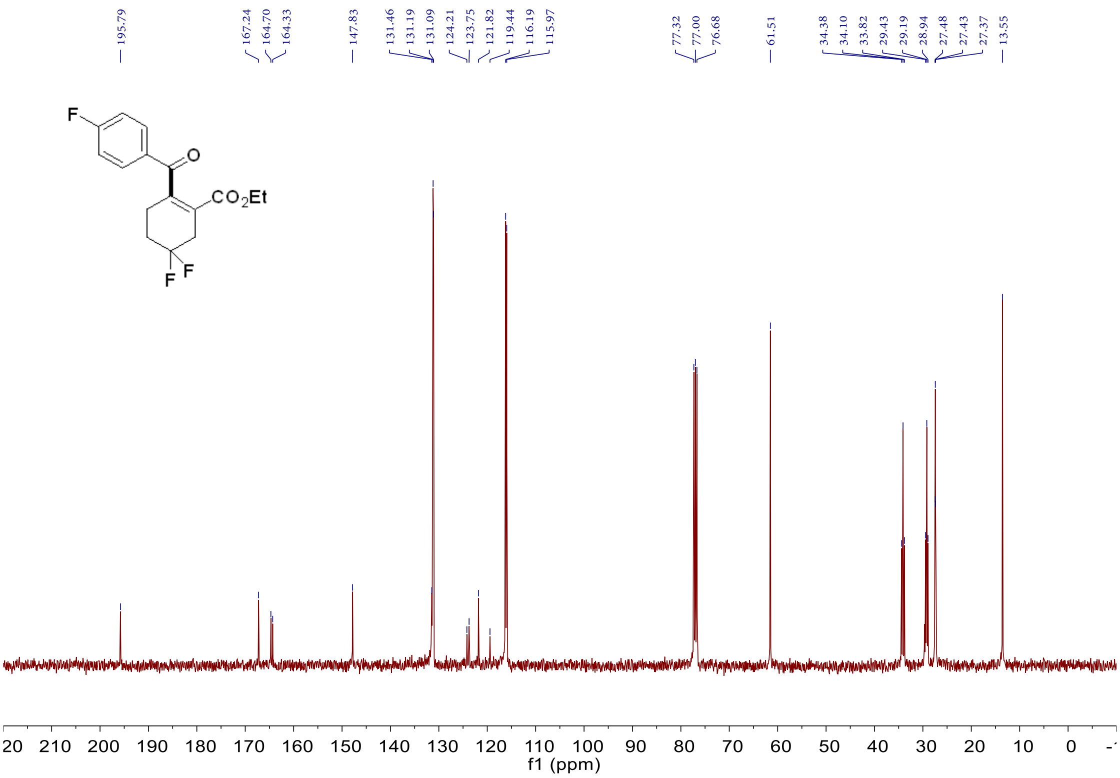

Supplementary Figure 170. ^{13}C NMR spectrum for compound **3kk**

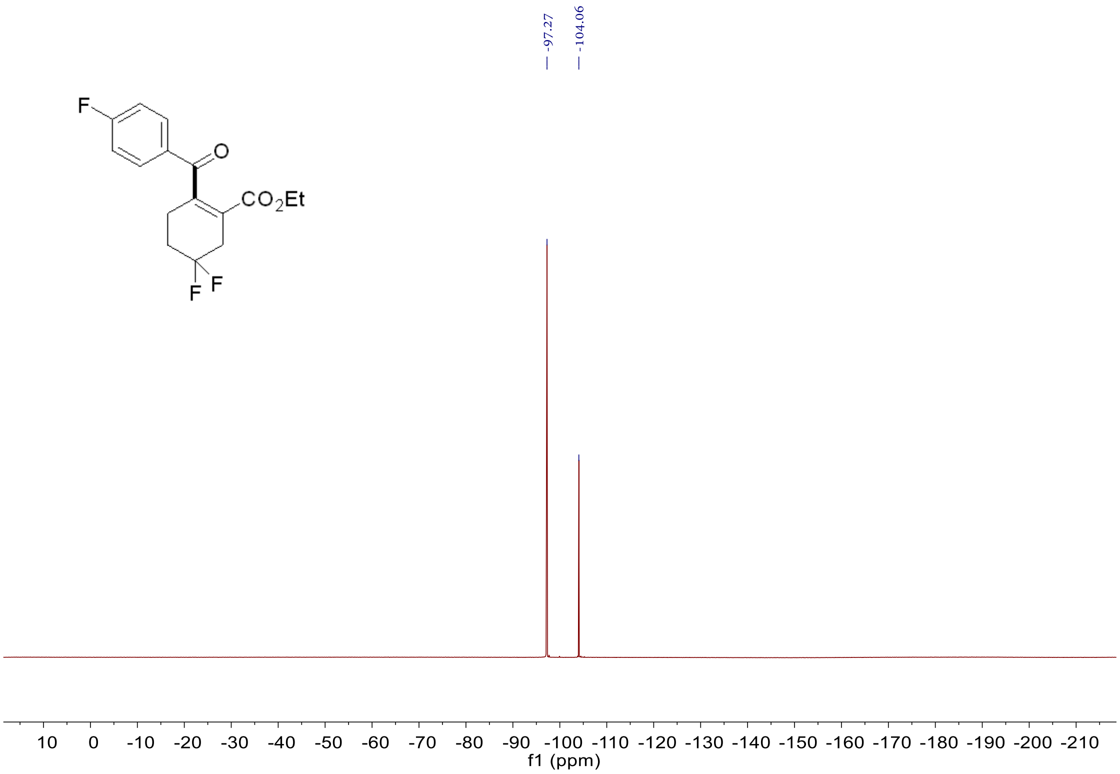

Supplementary Figure 171. ^{19}F NMR spectrum for compound **3kk**

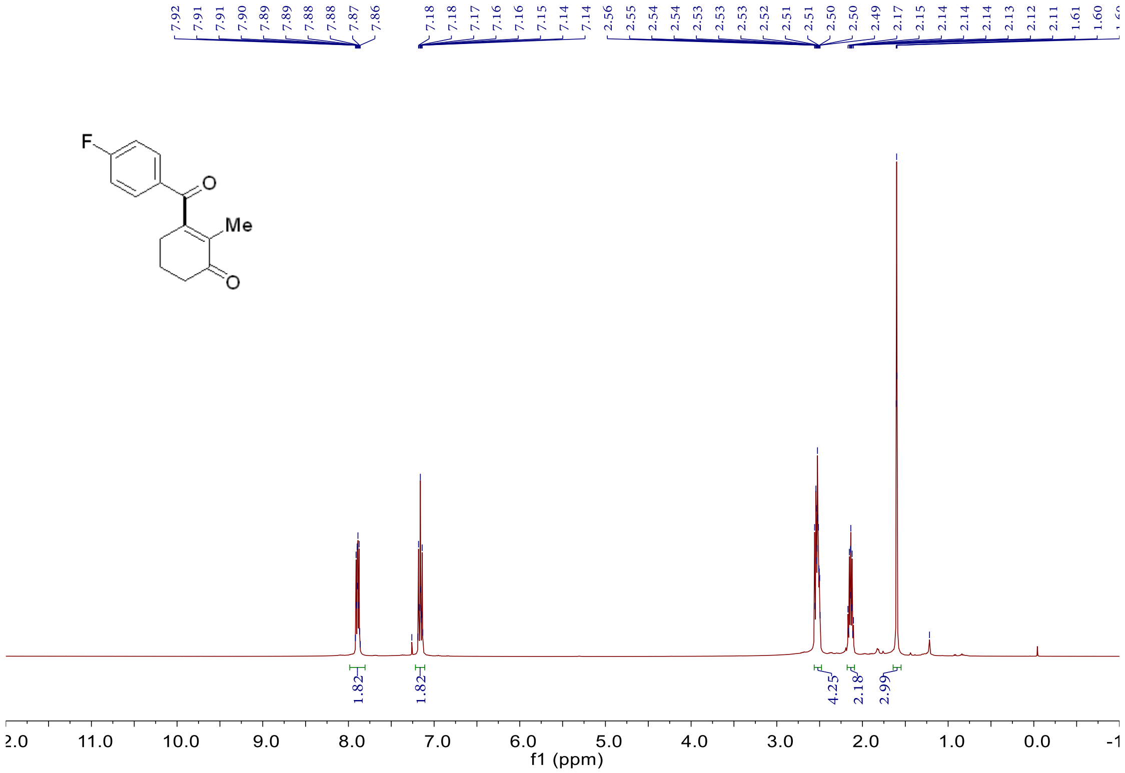

Supplementary Figure 172. ^{1}H NMR spectrum for compound **3ll**

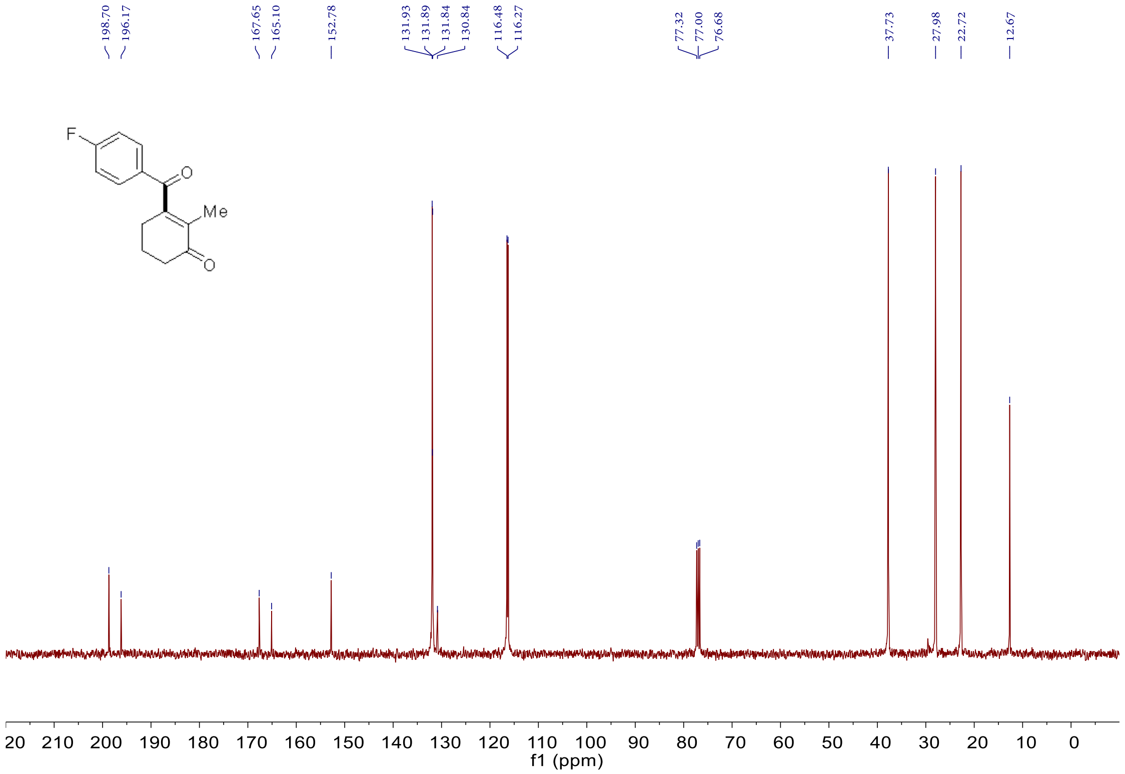

Supplementary Figure 173. ^{13}C NMR spectrum for compound **3ll**

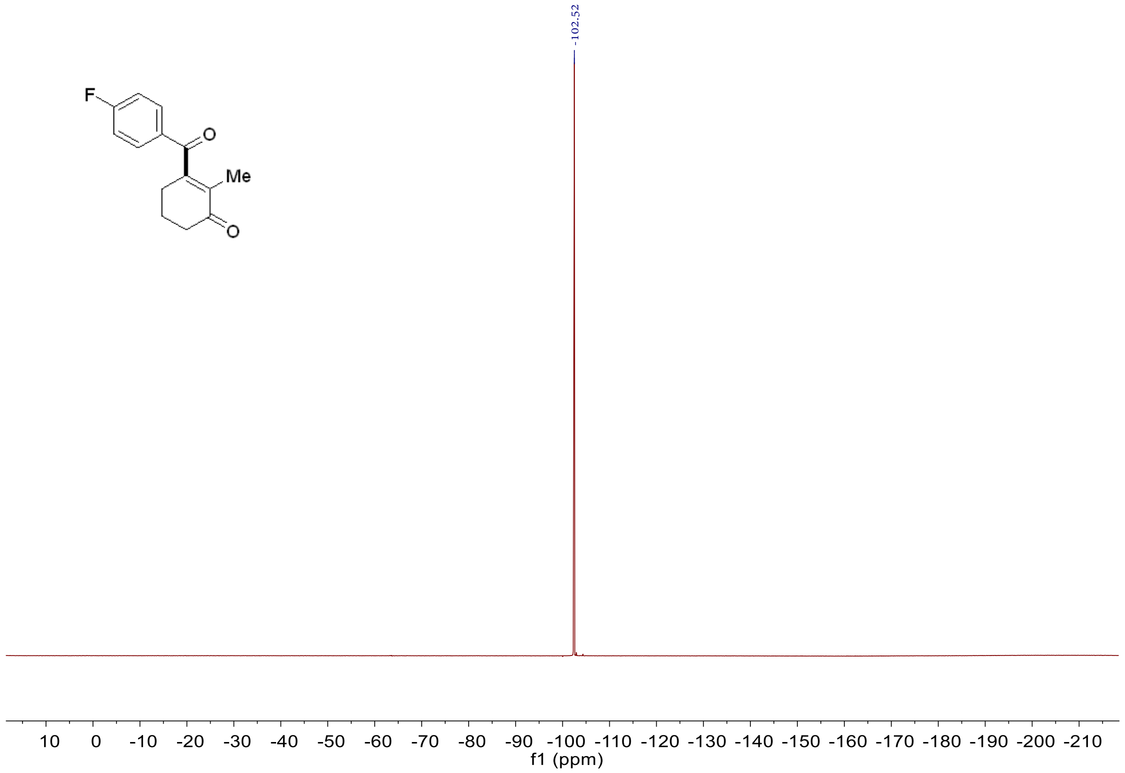

Supplementary Figure 174. ^{19}F NMR spectrum for compound **3ll**

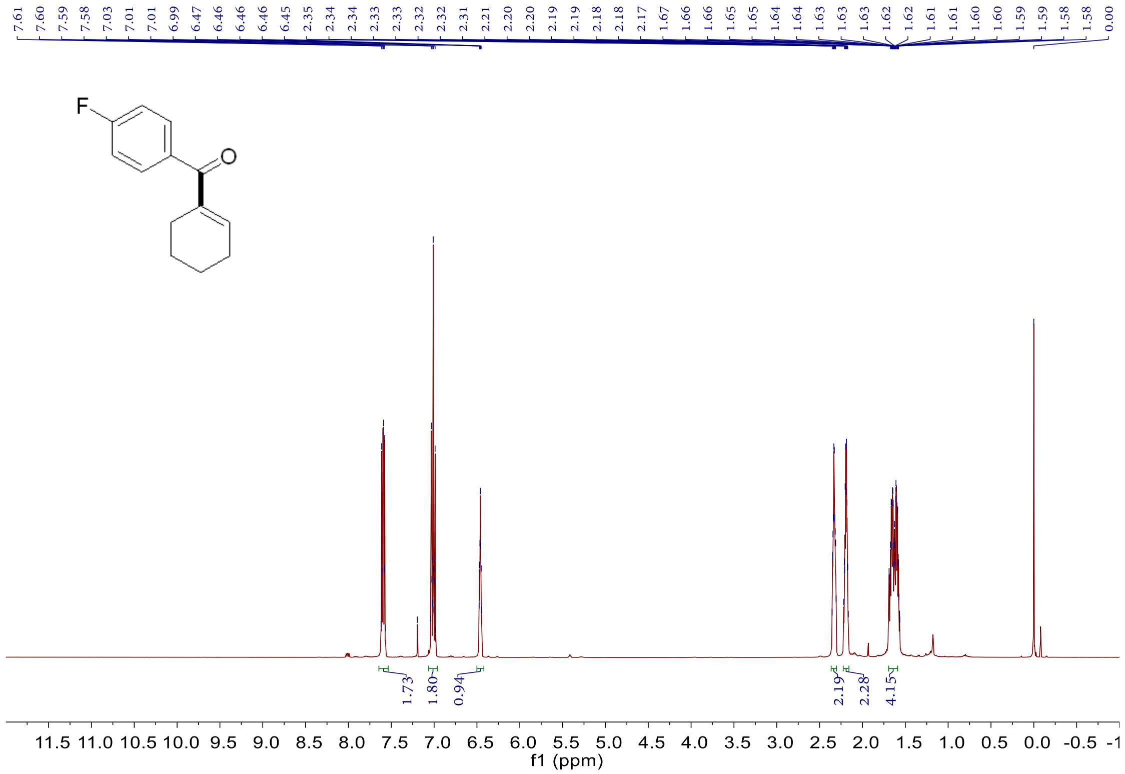

Supplementary Figure 175. ^{1}H NMR spectrum for compound **3mm**

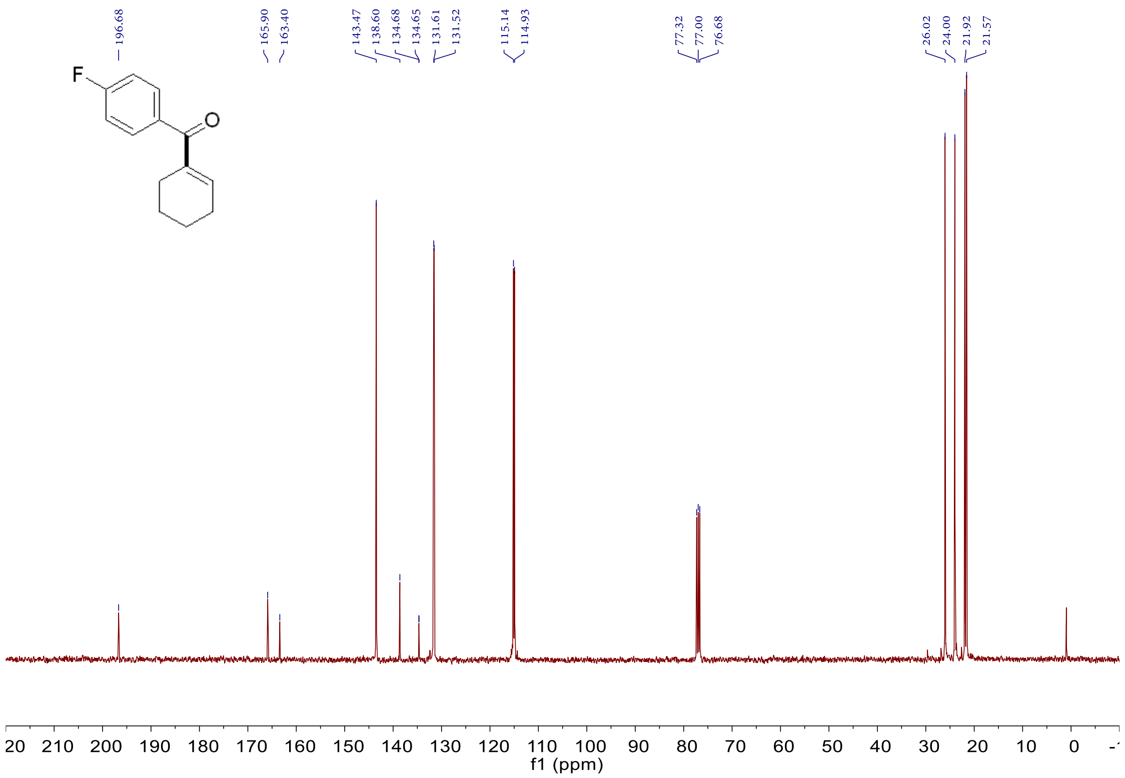

Supplementary Figure 176. ^{13}C NMR spectrum for compound **3mm**

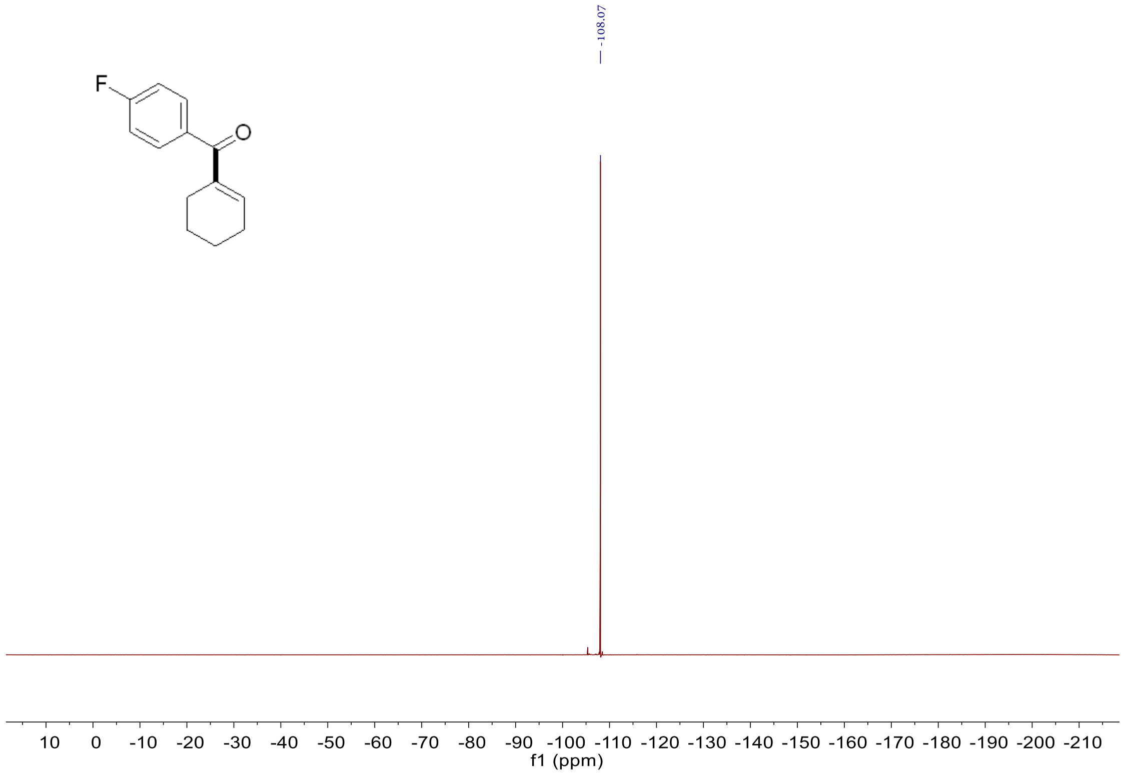

Supplementary Figure 177. ^{19}F NMR spectrum for compound **3mm**

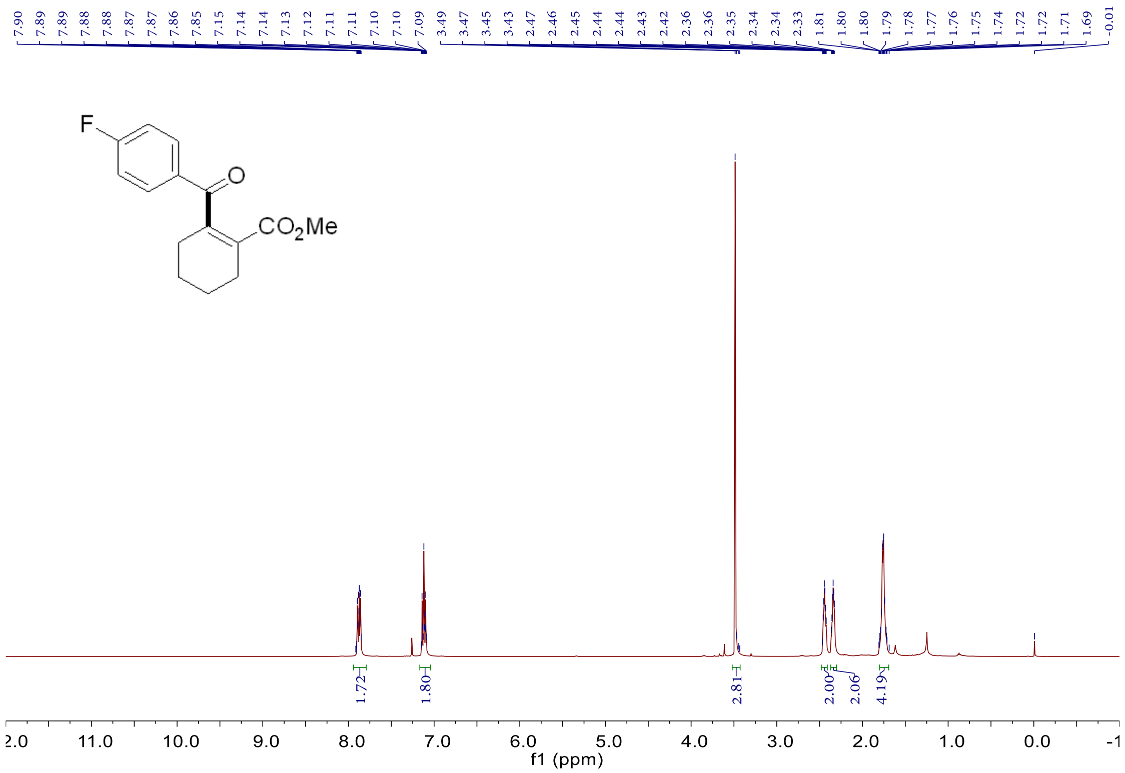

Supplementary Figure 178. ^{1}H NMR spectrum for compound **3nn**

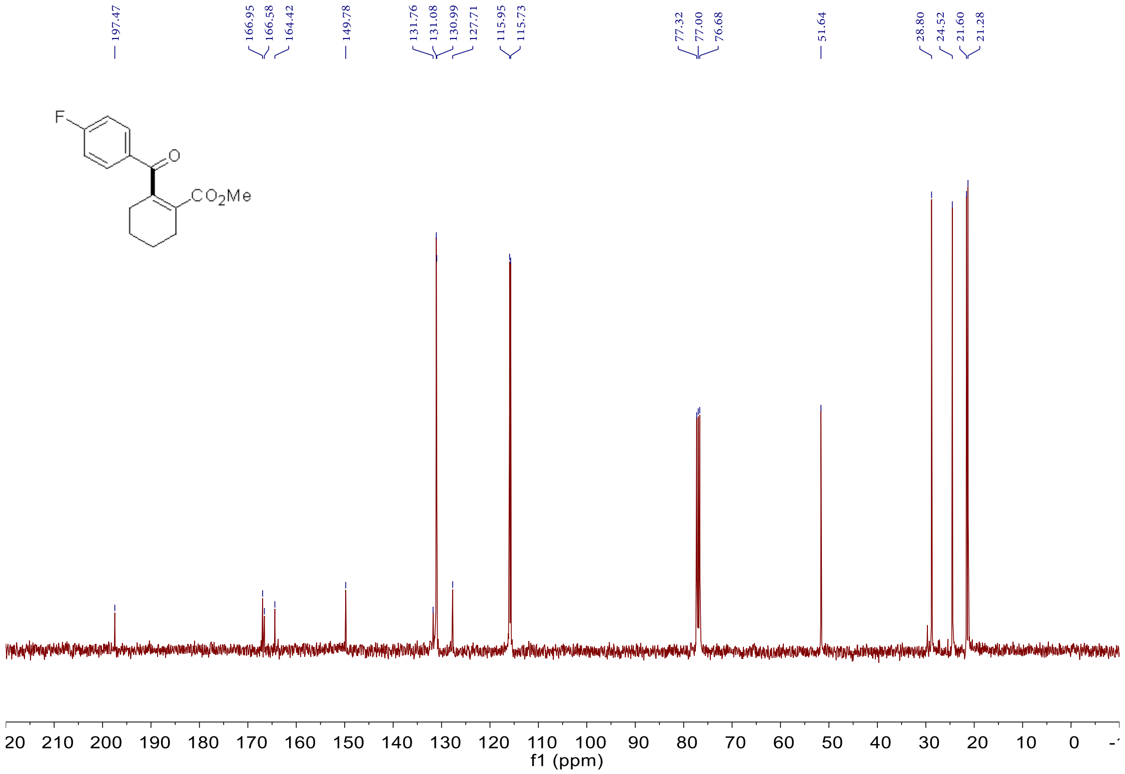

Supplementary Figure 179. ^{13}C NMR spectrum for compound **3nn**

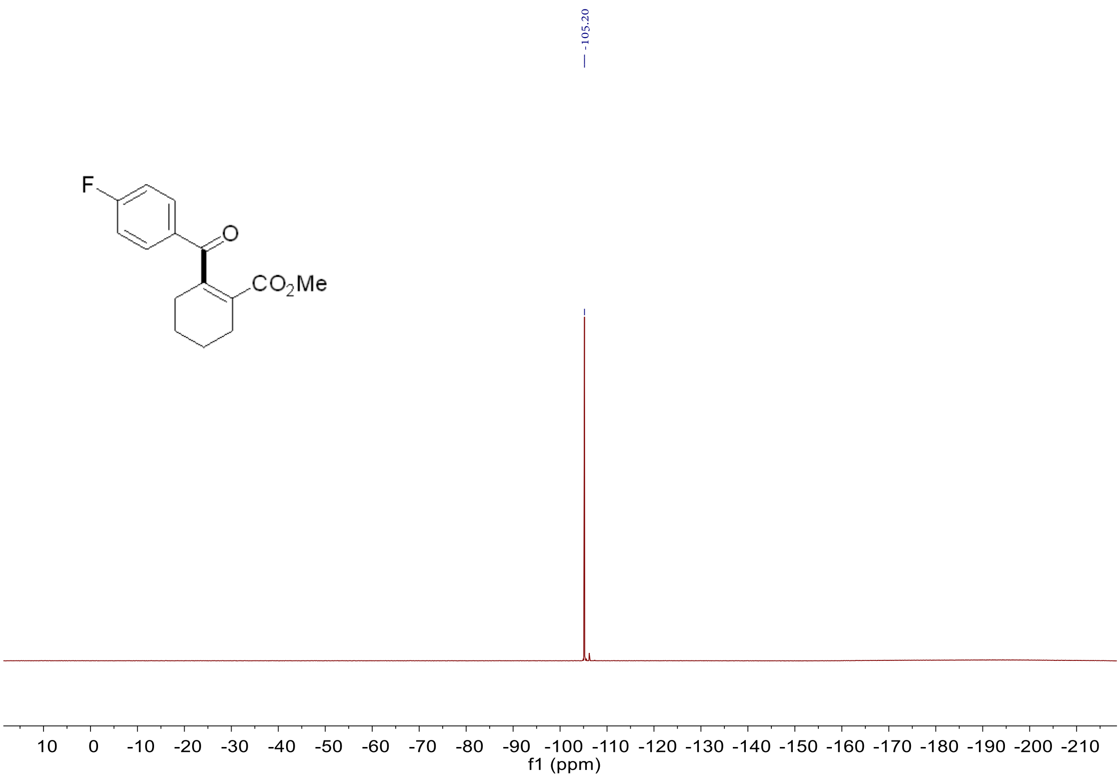

Supplementary Figure 180. ^{19}F NMR spectrum for compound **3nn**

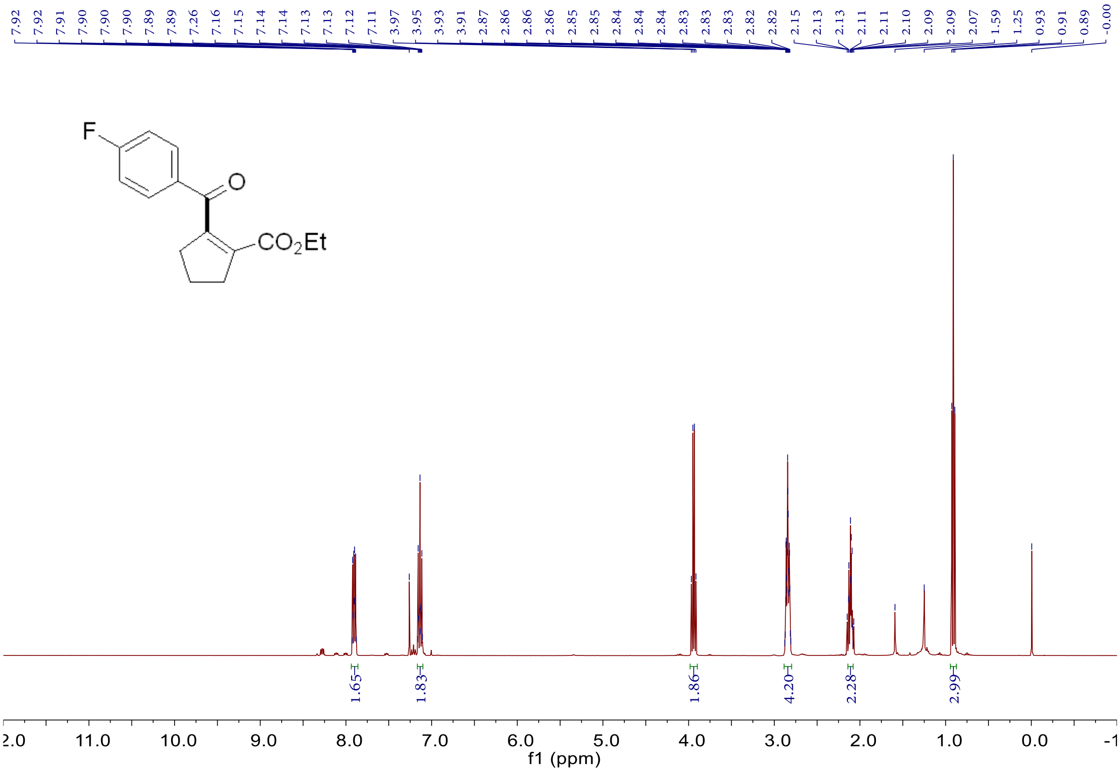

Supplementary Figure 181. ^{1}H NMR spectrum for compound **3oo**

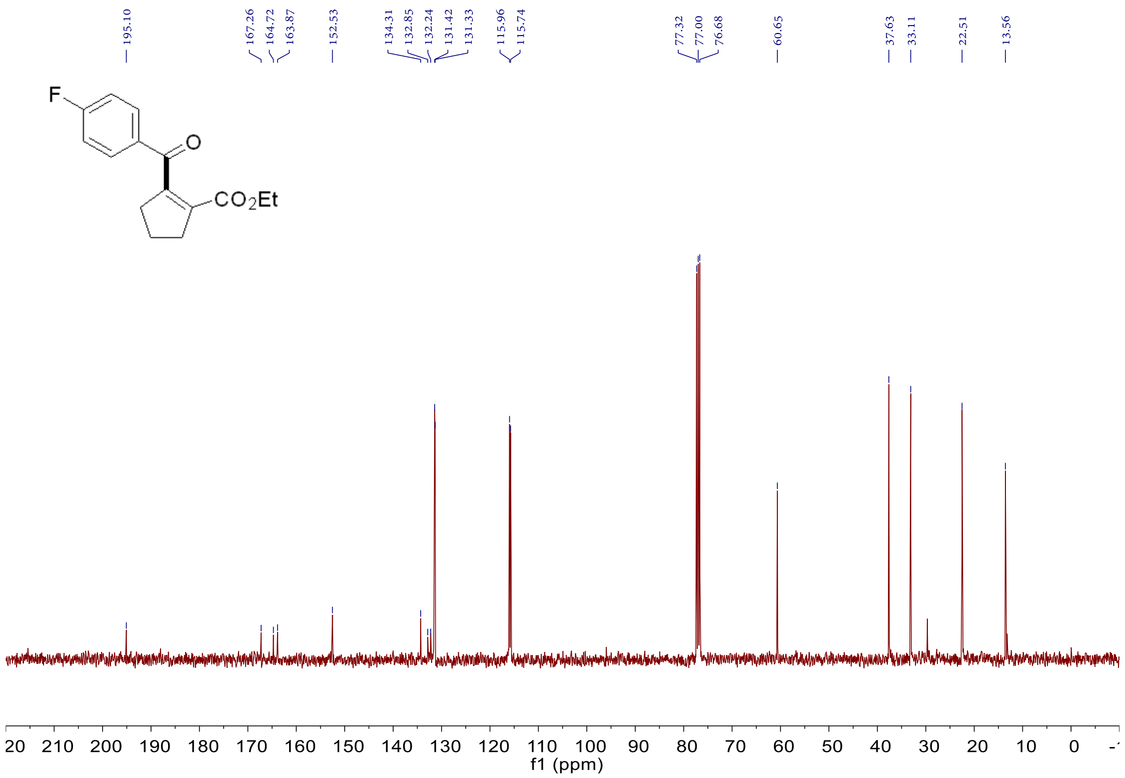

Supplementary Figure 182. ^{13}C NMR spectrum for compound **3oo**

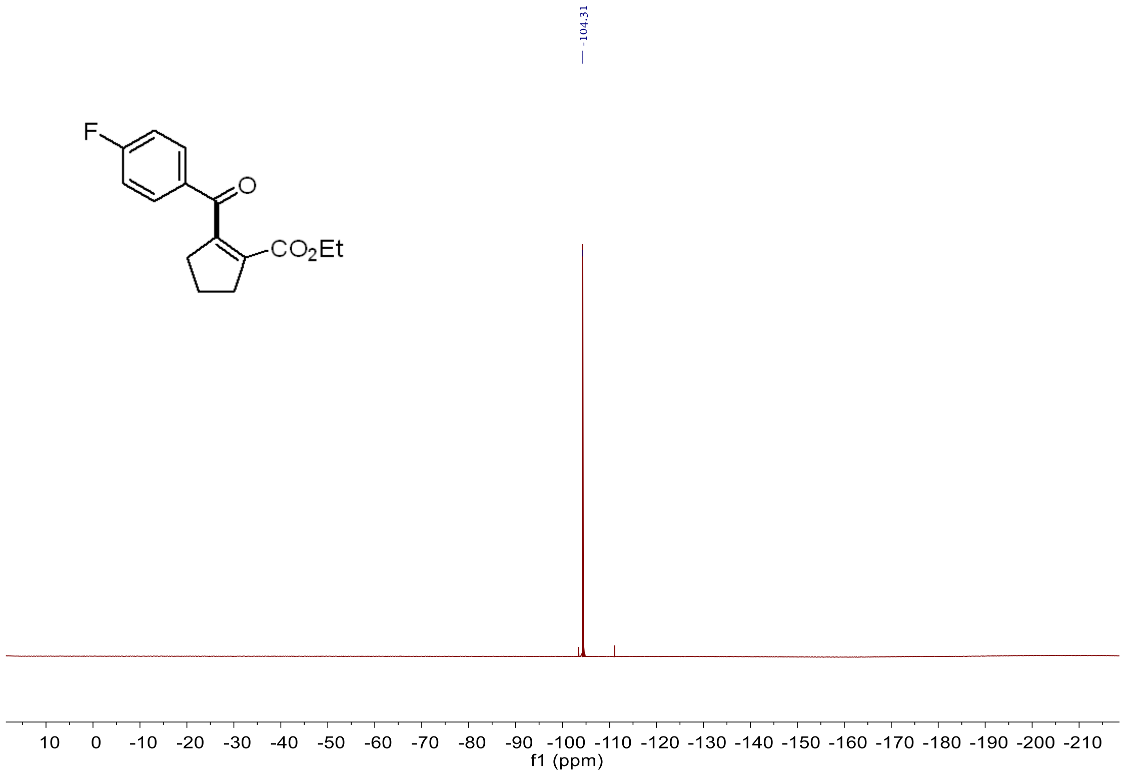

Supplementary Figure 183. ^{19}F NMR spectrum for compound **3oo**

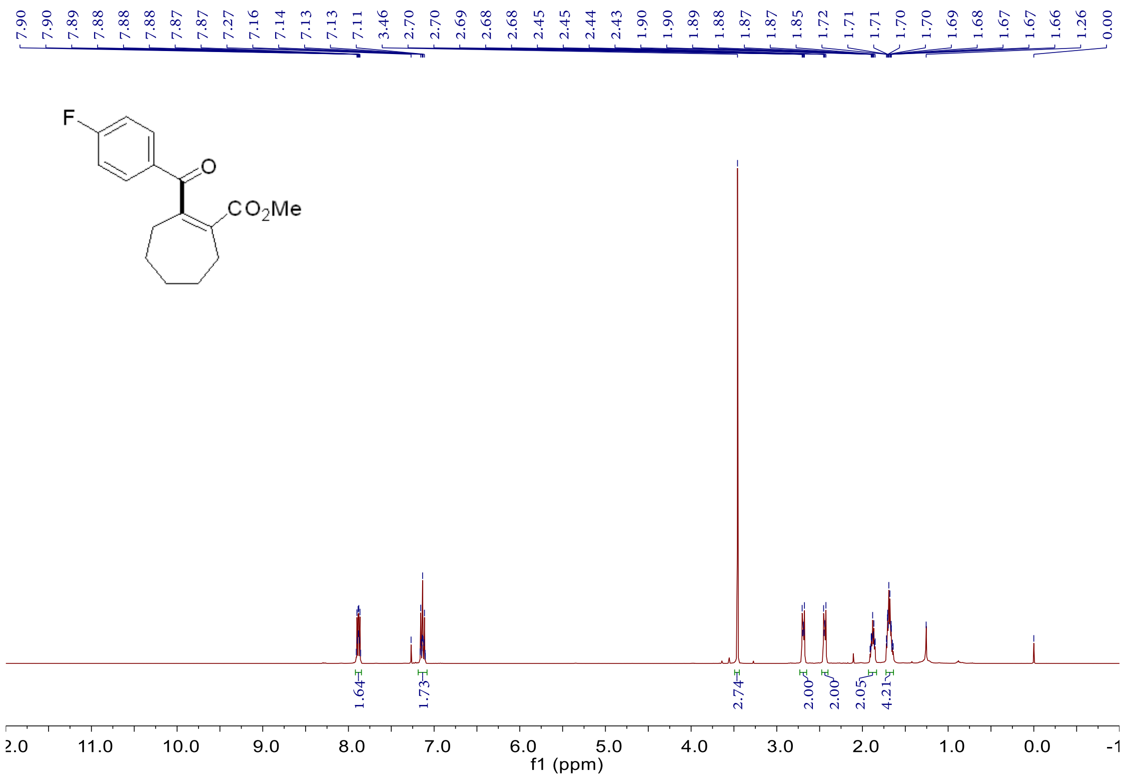

Supplementary Figure 184. ^{1}H NMR spectrum for compound **3pp**

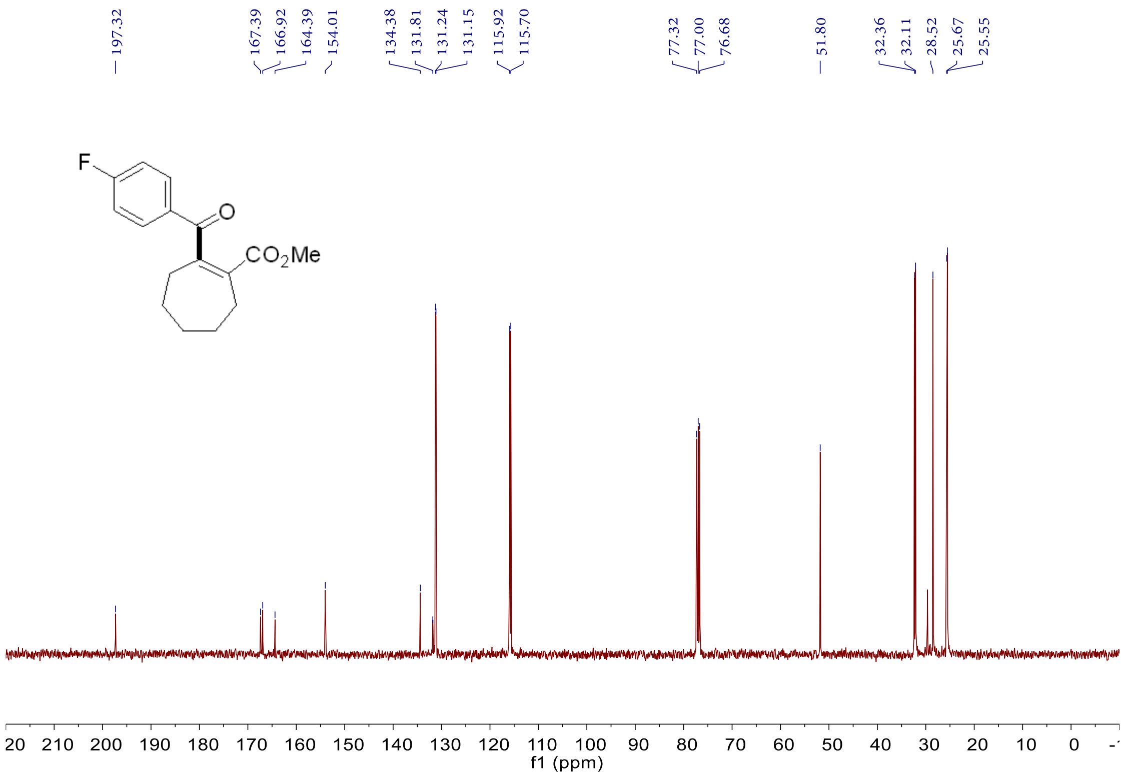

Supplementary Figure 185. ^{13}C NMR spectrum for compound **3pp**

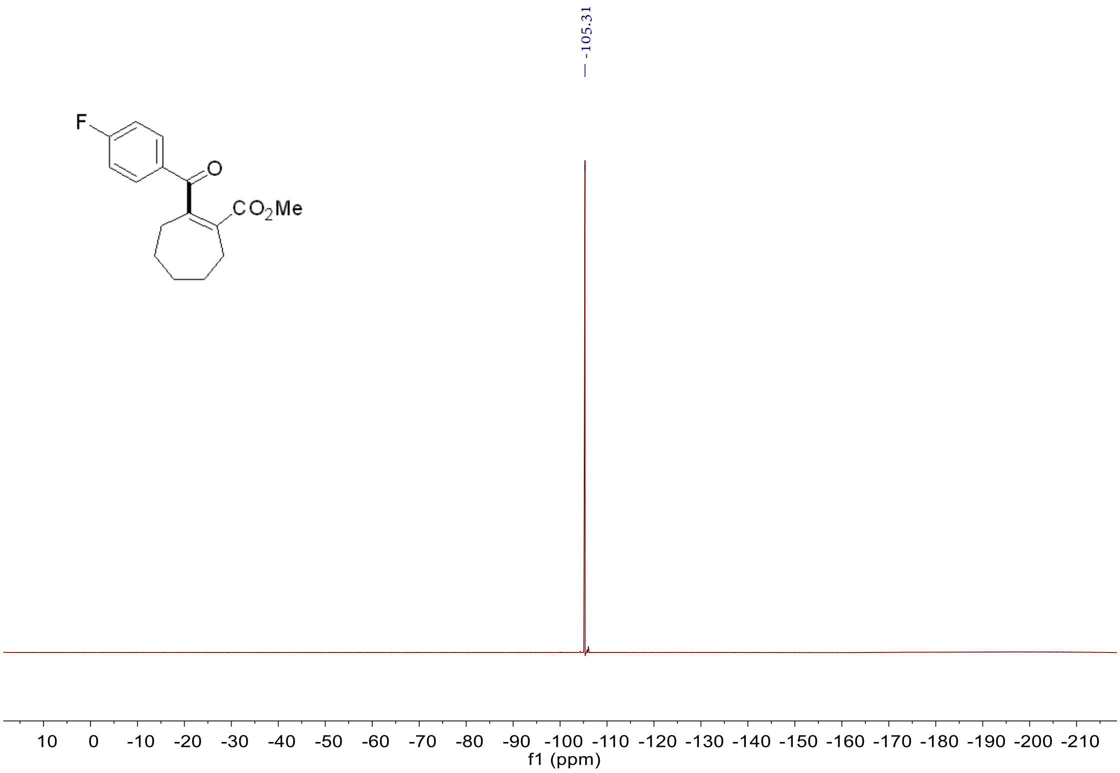

Supplementary Figure 186. ^{19}F NMR spectrum for compound **3pp**

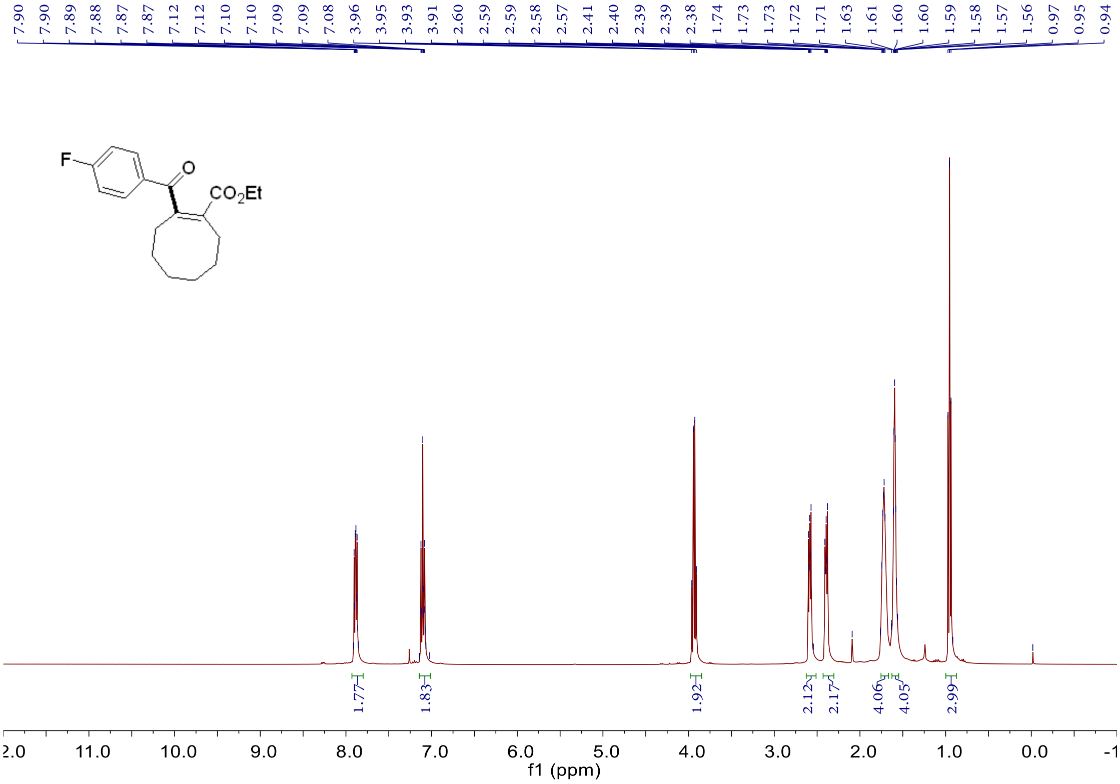

Supplementary Figure 187. ^{1}H NMR spectrum for compound **3qq**

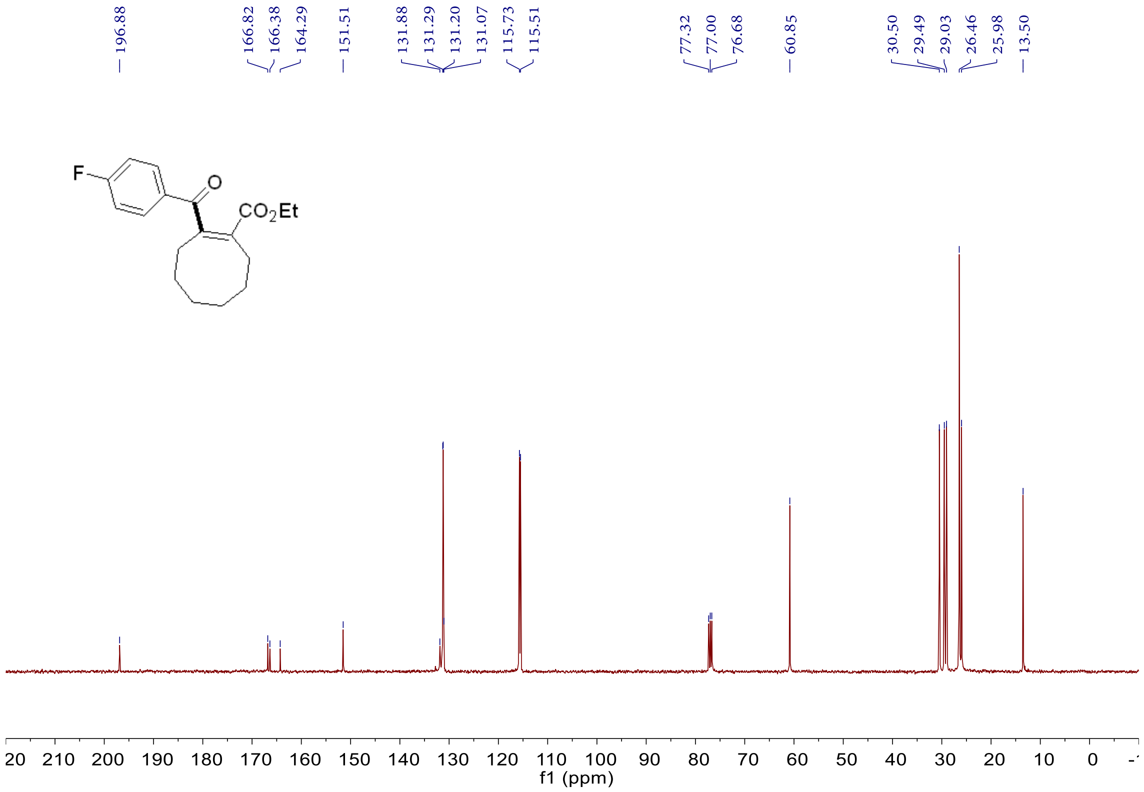

Supplementary Figure 188. ^{13}C NMR spectrum for compound **3qq**

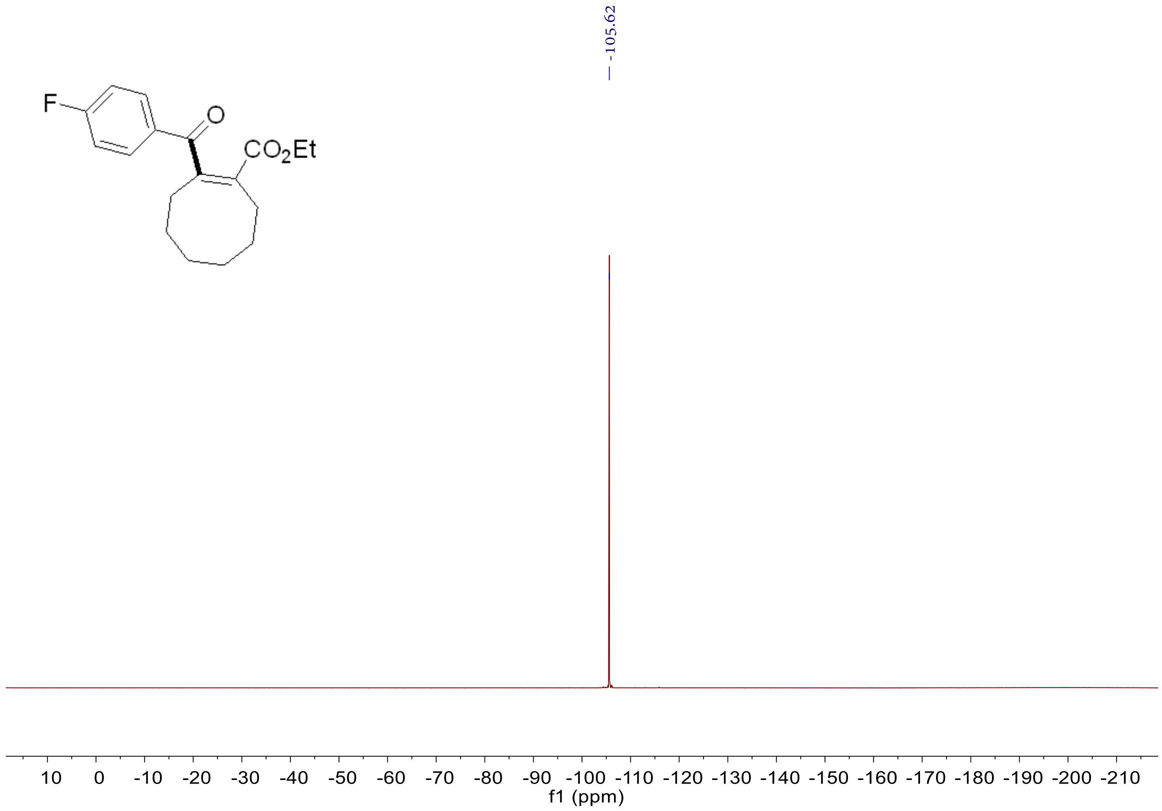

Supplementary Figure 189. ^{19}F NMR spectrum for compound **3qq**

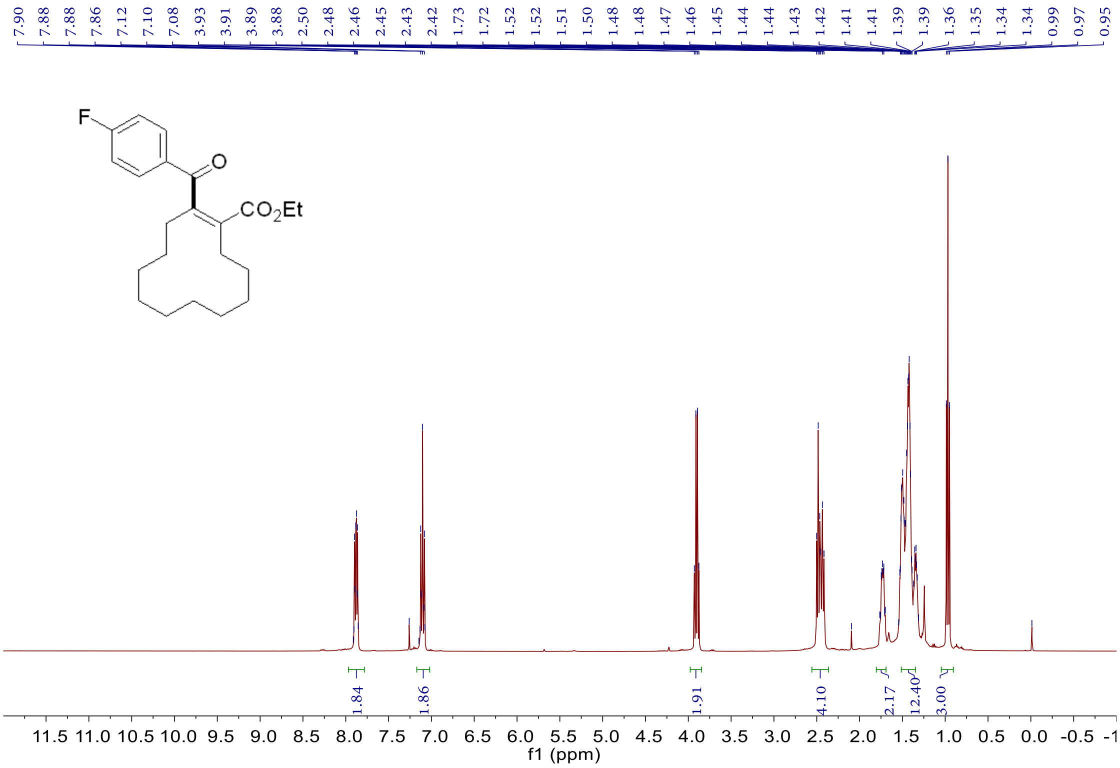

Supplementary Figure 190. ^{1}H NMR spectrum for compound **3rr**

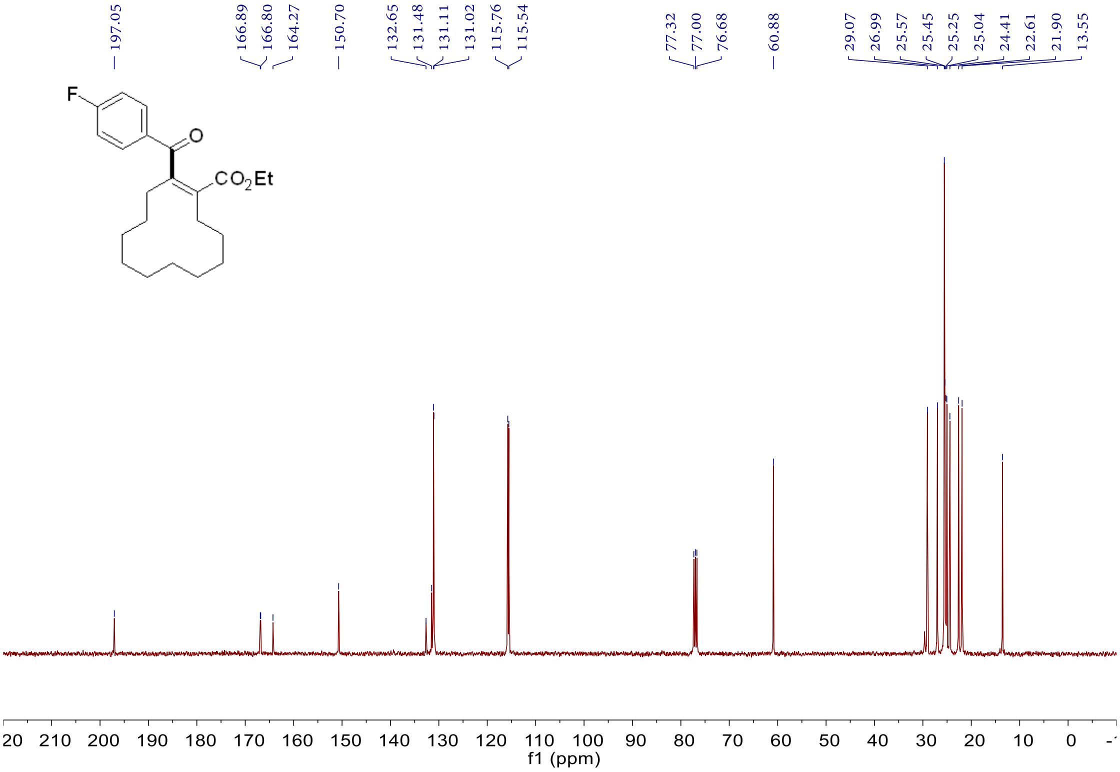

Supplementary Figure 191. ^{13}C NMR spectrum for compound **3rr**

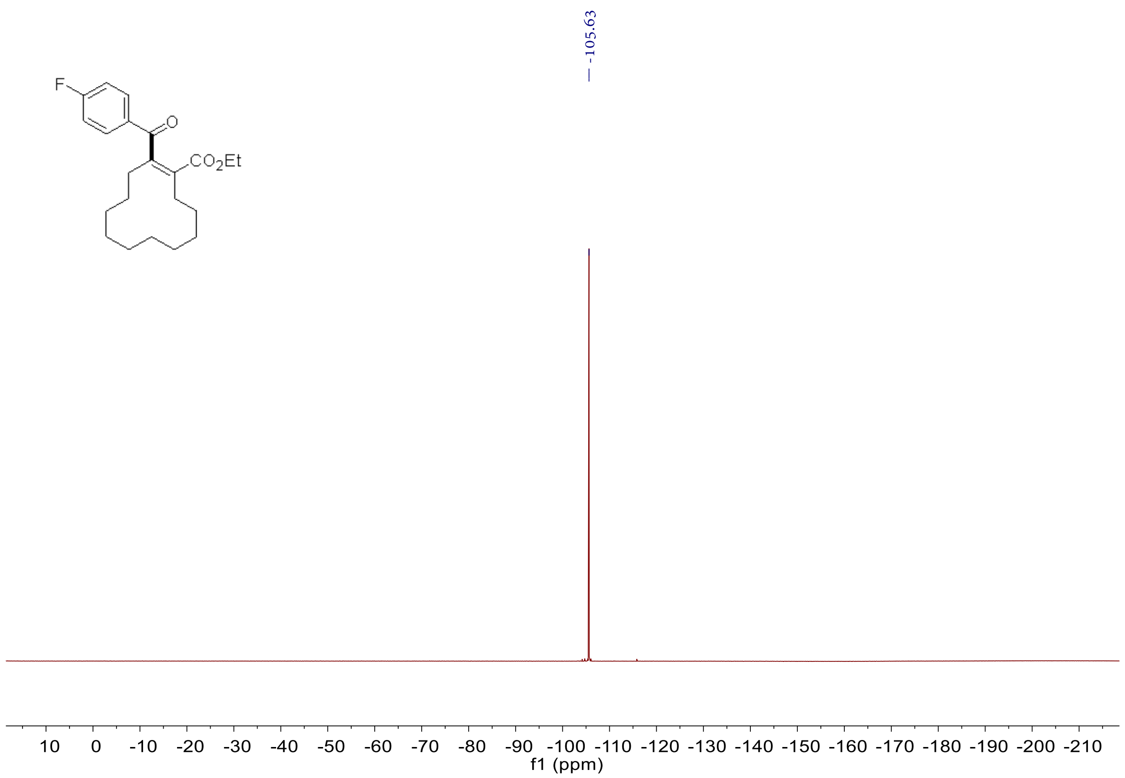

Supplementary Figure 192. ^{19}F NMR spectrum for compound **3rr**

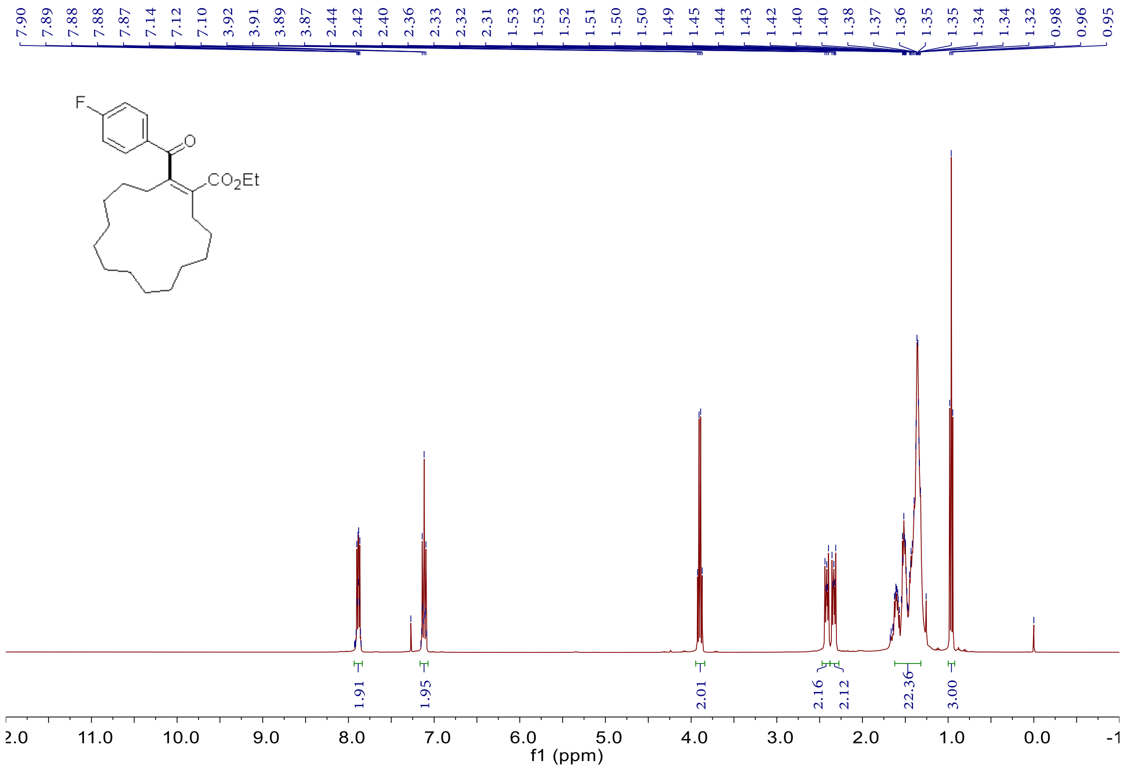

Supplementary Figure 193. ^{1}H NMR spectrum for compound **3ss**

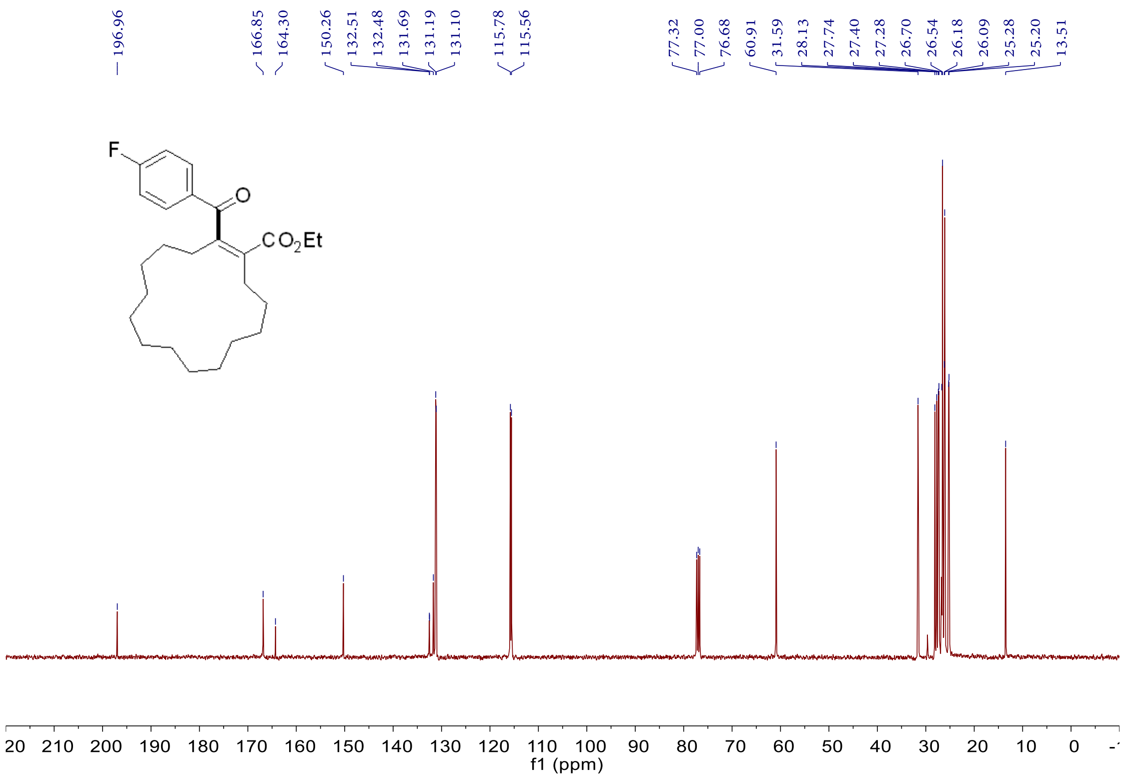

Supplementary Figure 194. ^{13}C NMR spectrum for compound **3ss**

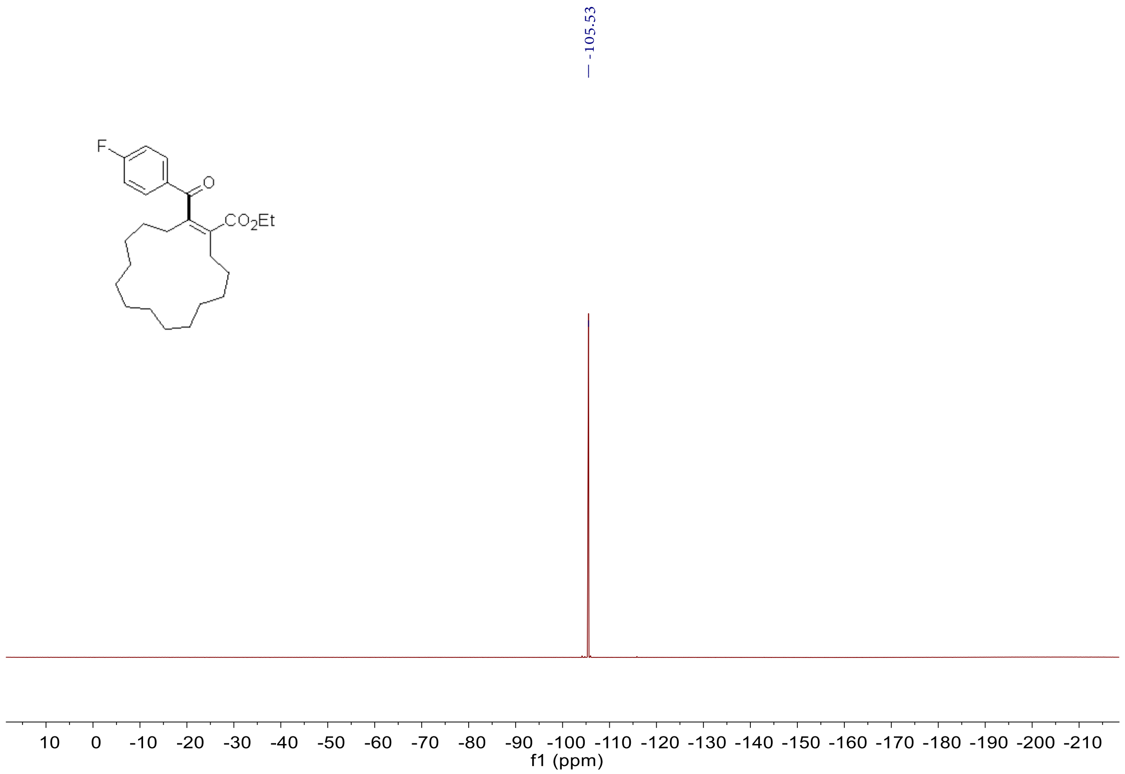

Supplementary Figure 195. ^{19}F NMR spectrum for compound **3ss**

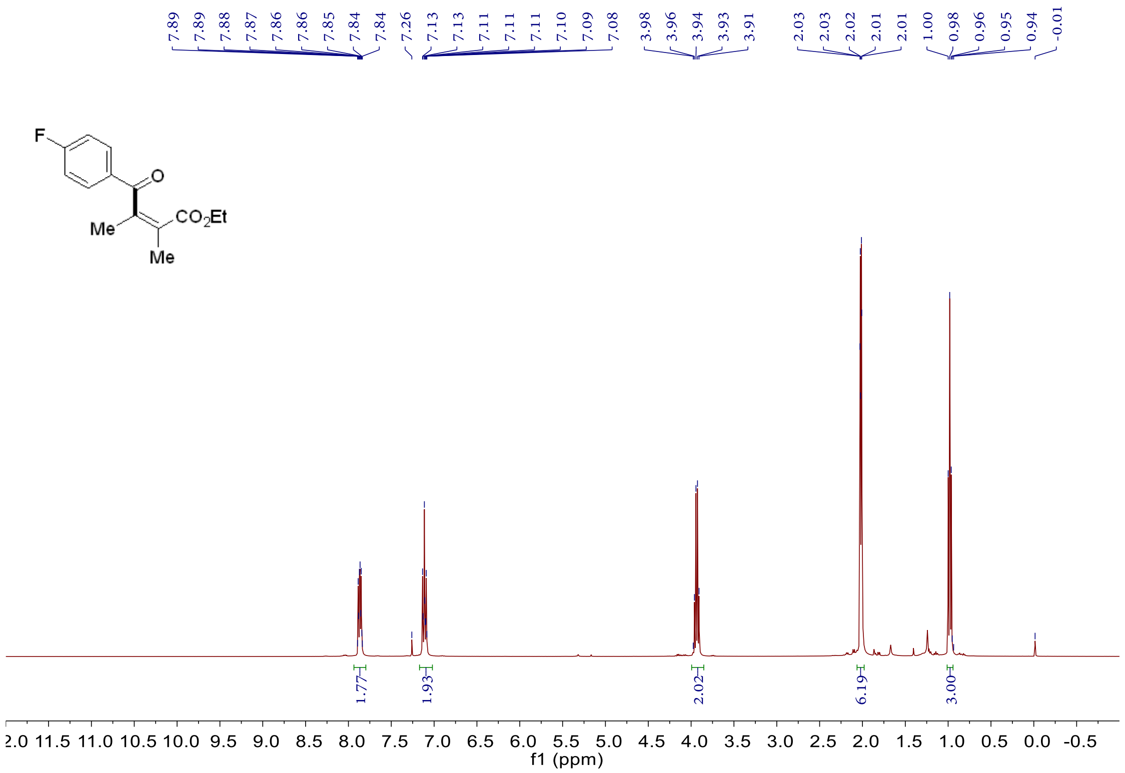

Supplementary Figure 196. ^{1}H NMR spectrum for compound **3tt**

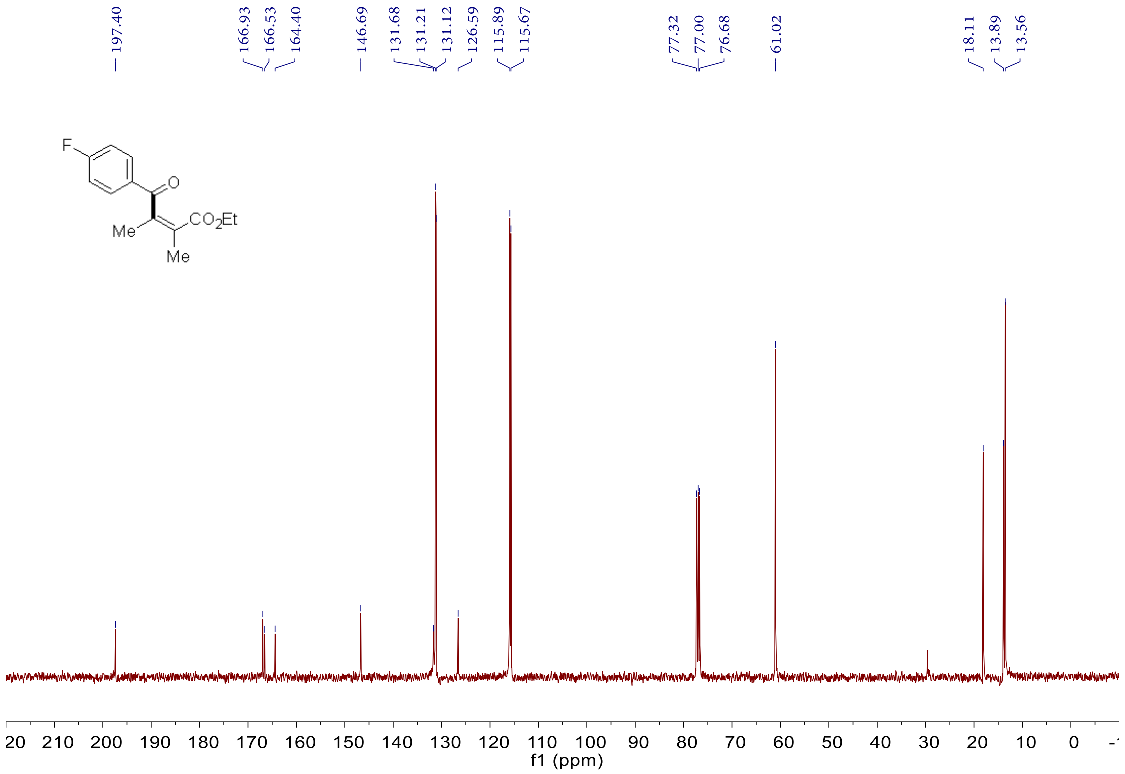

Supplementary Figure 197. ^{13}C NMR spectrum for compound **3tt**

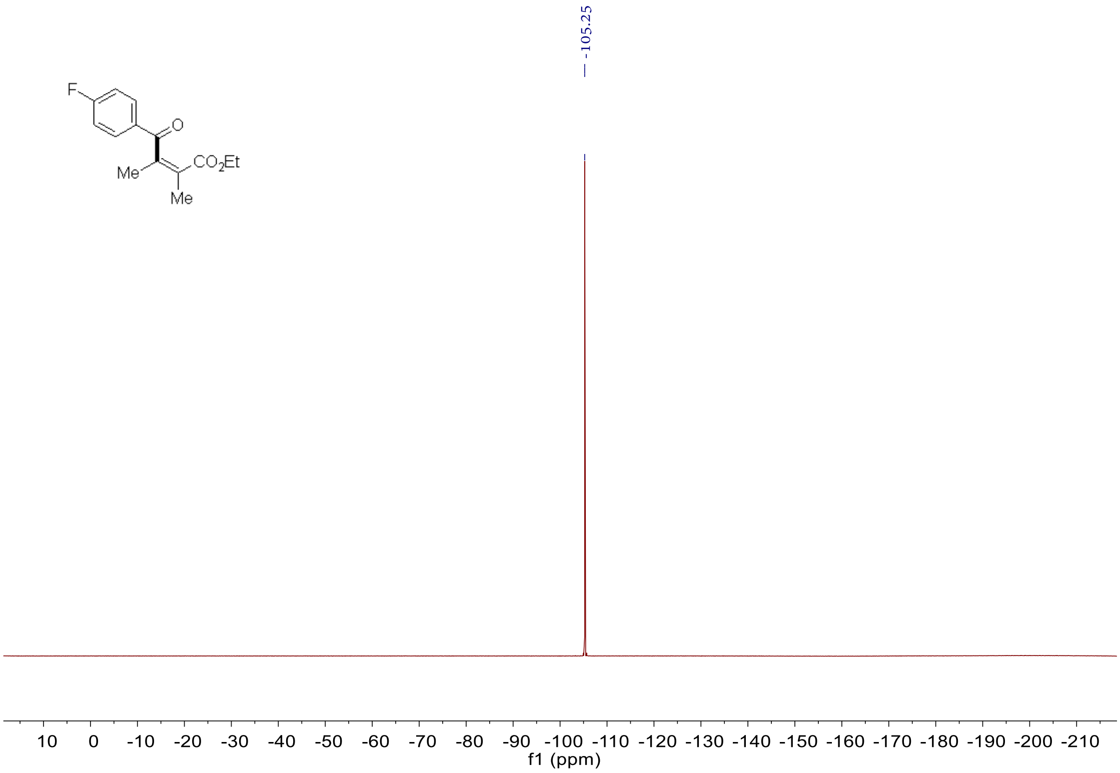

Supplementary Figure 198. ^{19}F NMR spectrum for compound **3tt**

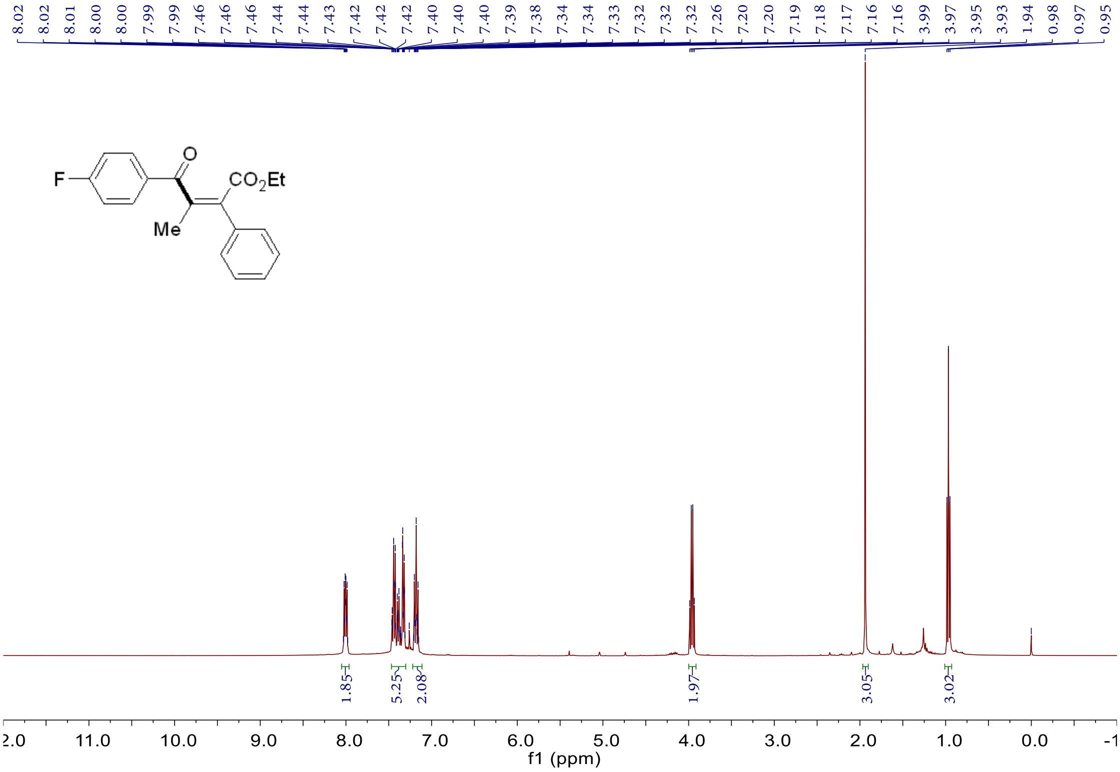

Supplementary Figure 199. ^{1}H NMR spectrum for compound **3uu**

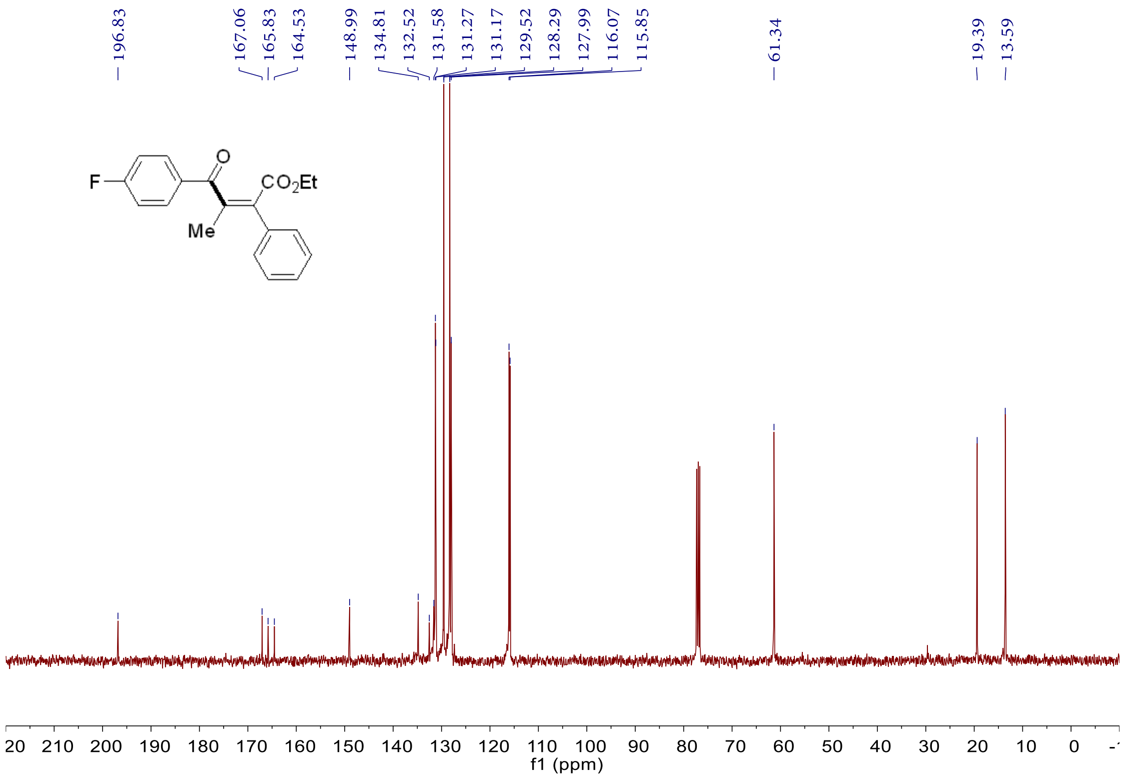

Supplementary Figure 200. ^{13}C NMR spectrum for compound **3uu**

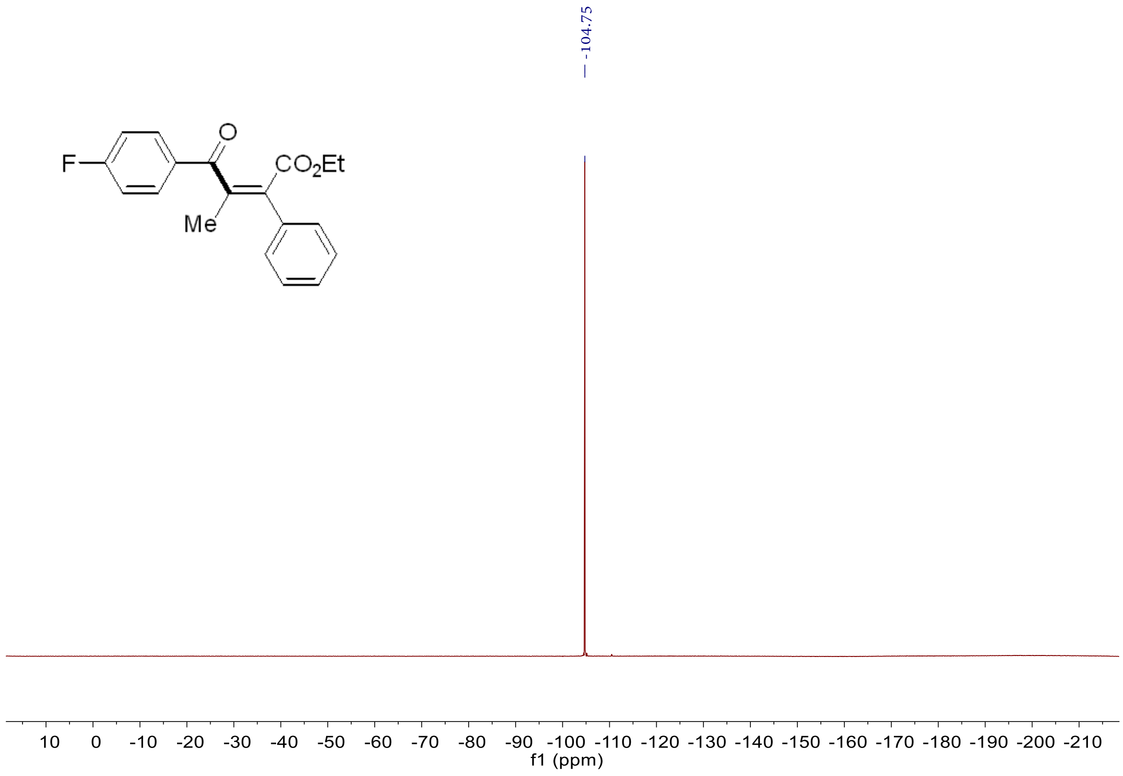

Supplementary Figure 201. ^{19}F NMR spectrum for compound **3uu**

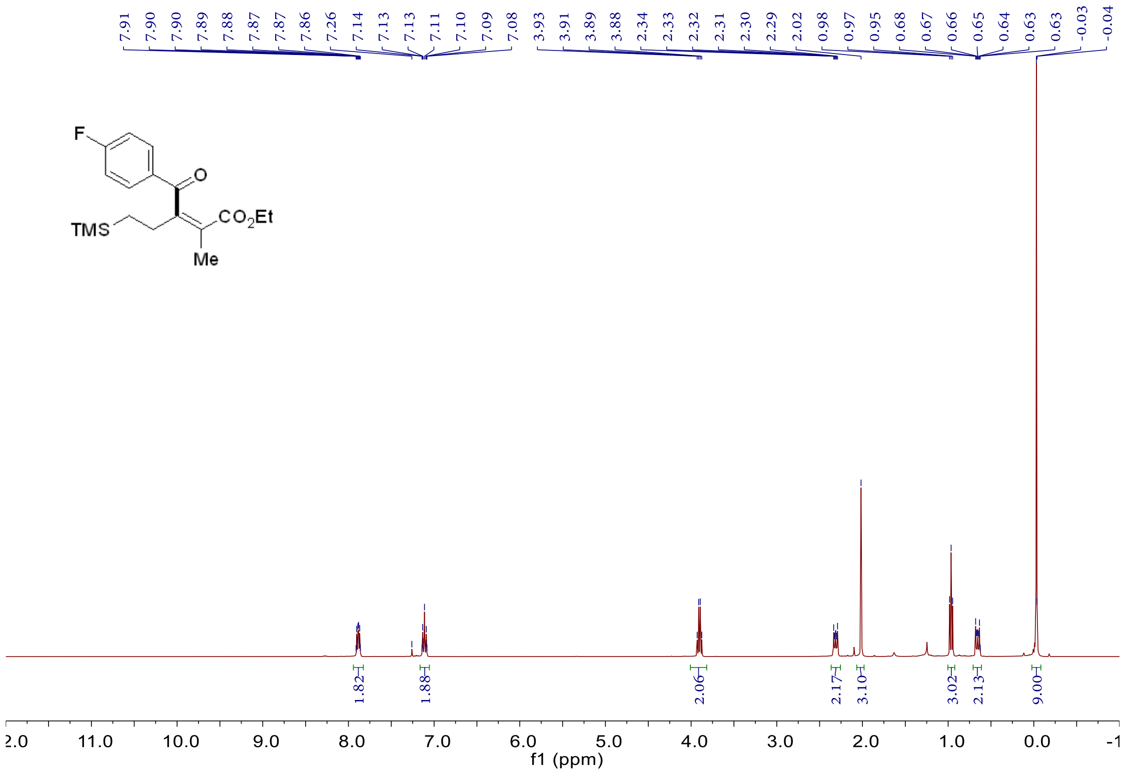

Supplementary Figure 202. ^{1}H NMR spectrum for compound **3vv**

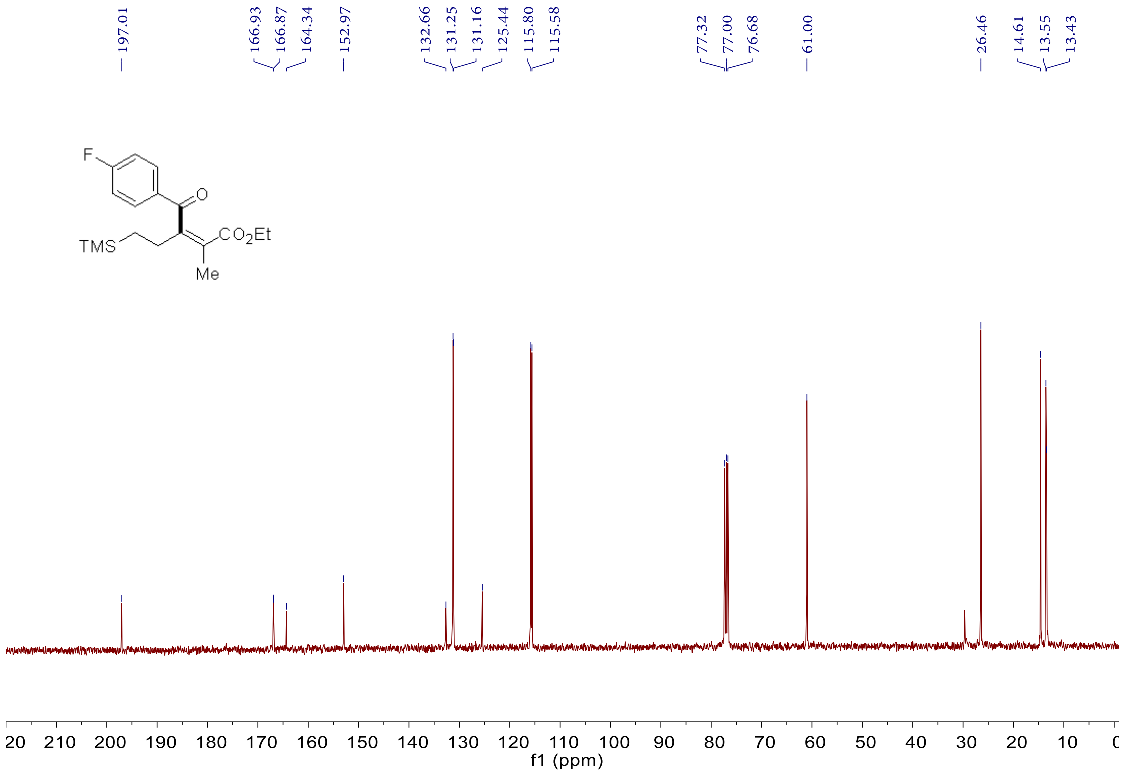

Supplementary Figure 203. ^{13}C NMR spectrum for compound **3vv**

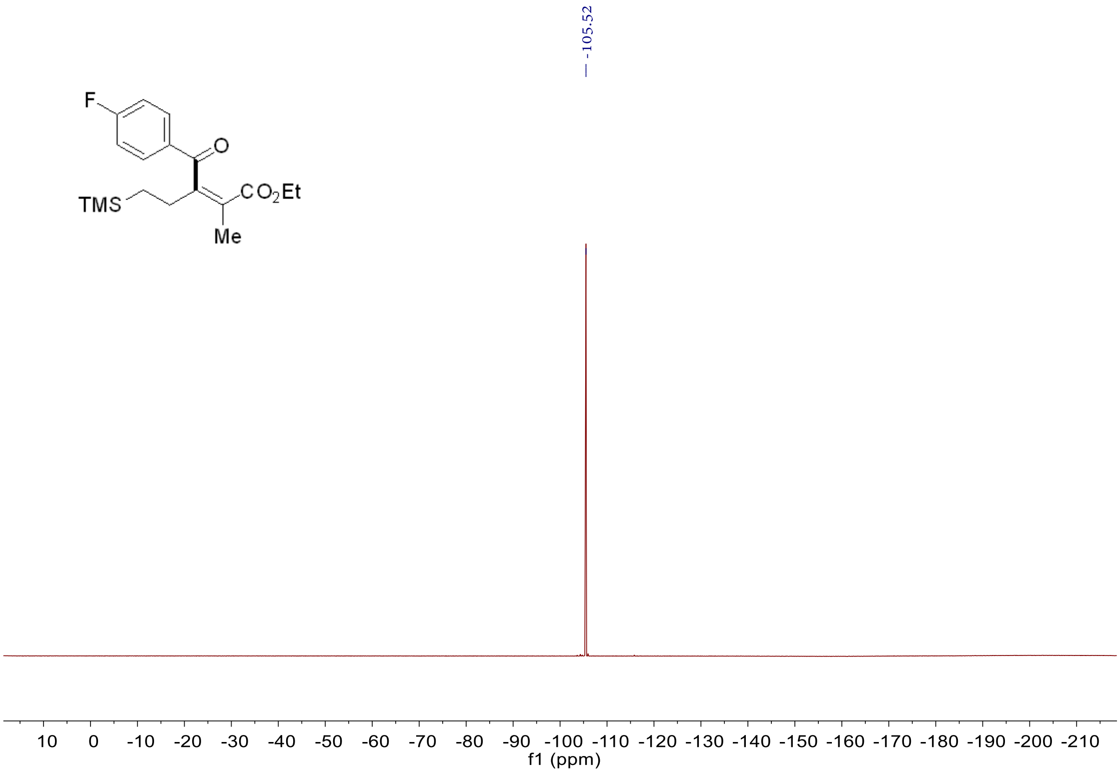

Supplementary Figure 204. ^{19}F NMR spectrum for compound **3vv**

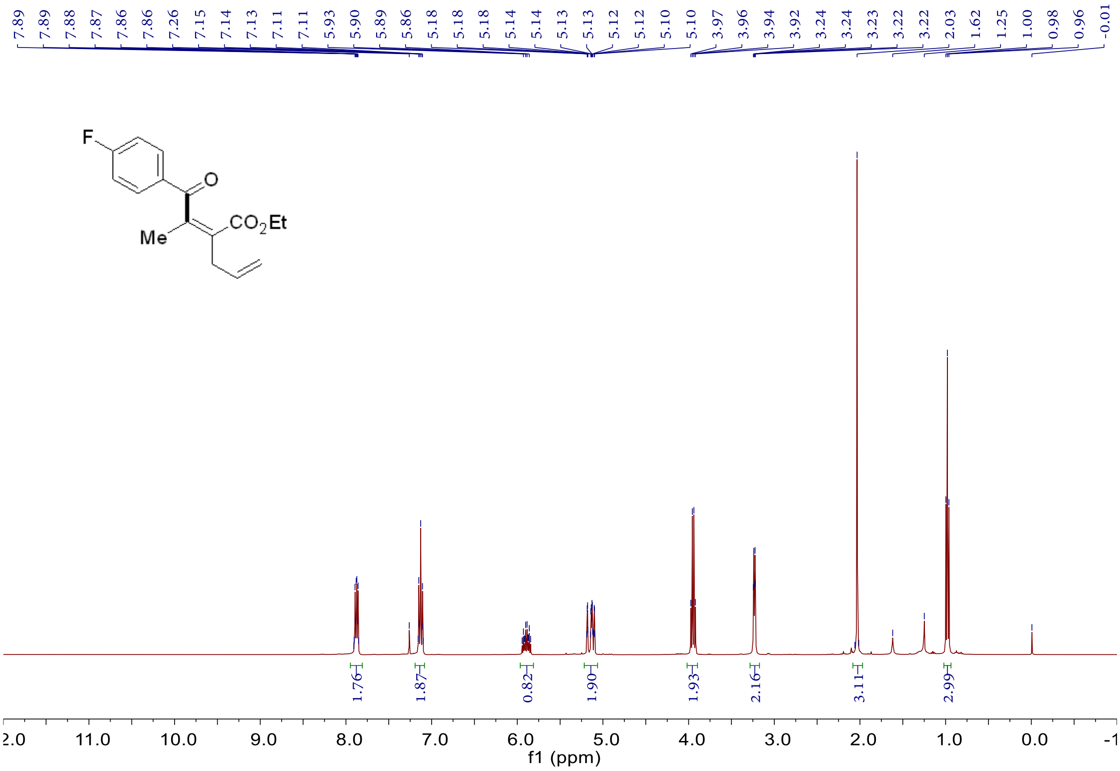

Supplementary Figure 205. ^{1}H NMR spectrum for compound **3ww**

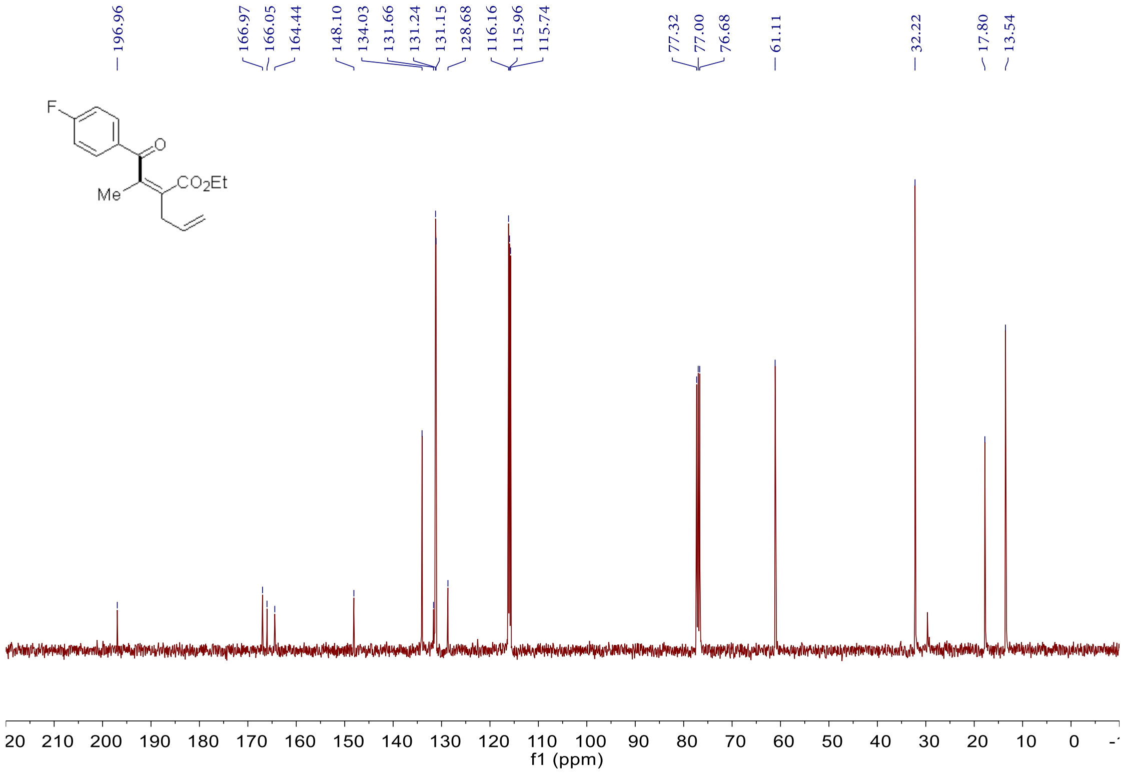

Supplementary Figure 206. ^{13}C NMR spectrum for compound **3ww**

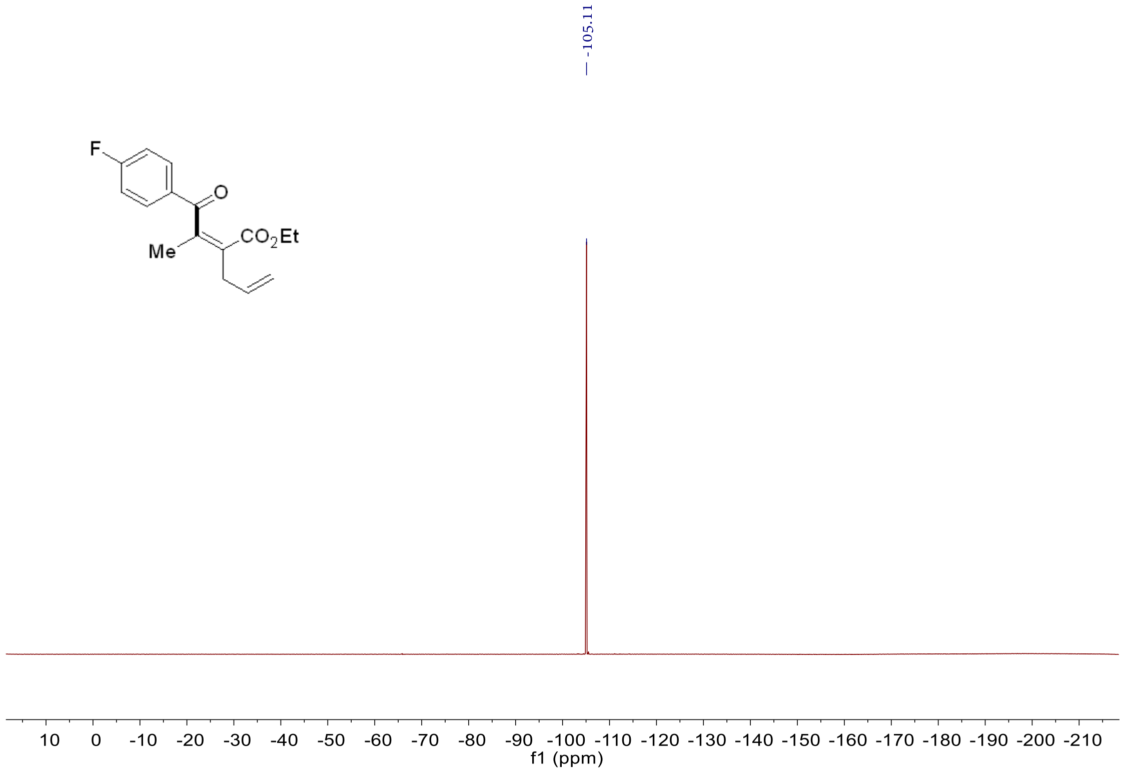

Supplementary Figure 207. ^{19}F NMR spectrum for compound **3ww**

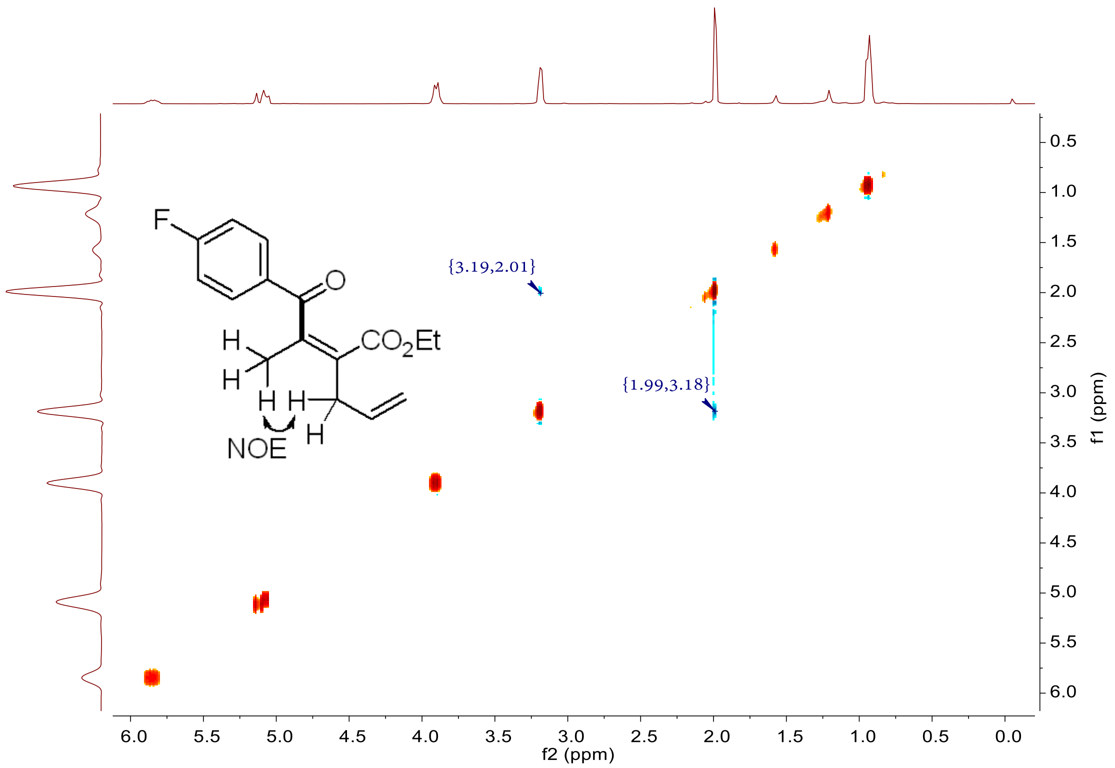

Supplementary Figure 208. NOESY spectrum for compound **3ww**

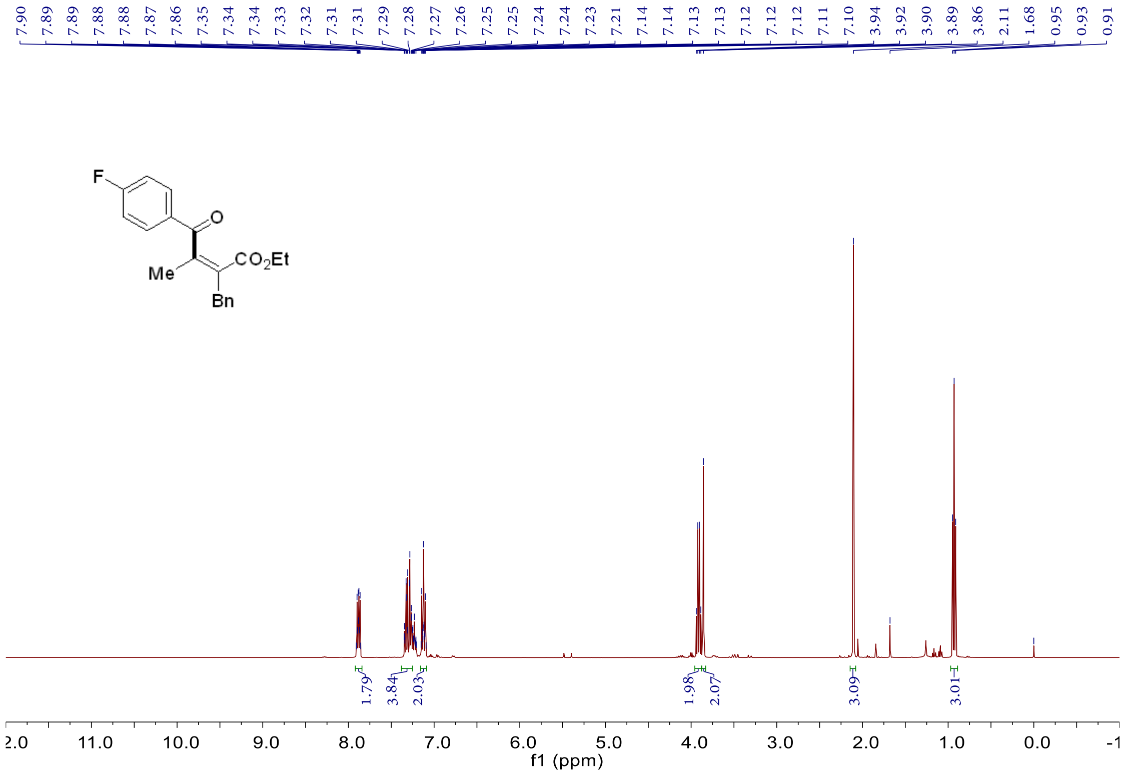

Supplementary Figure 209. ^{1}H NMR spectrum for compound **3xx**

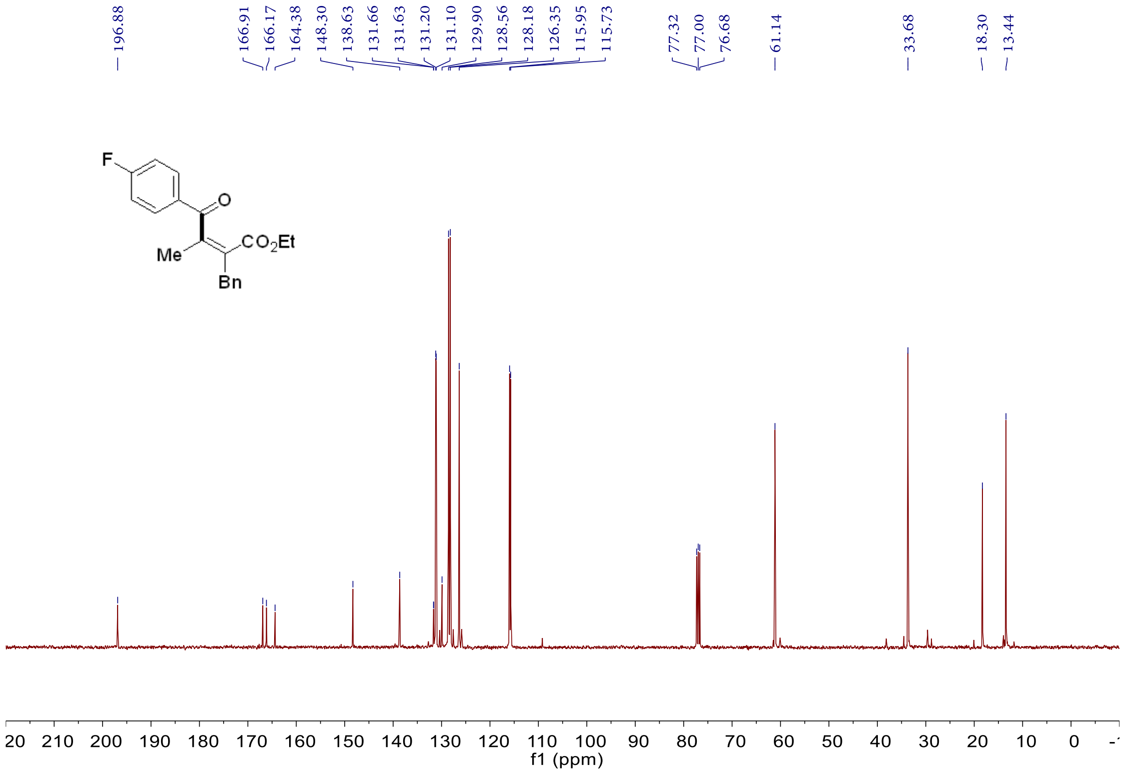

Supplementary Figure 210. ^{13}C NMR spectrum for compound **3xx**

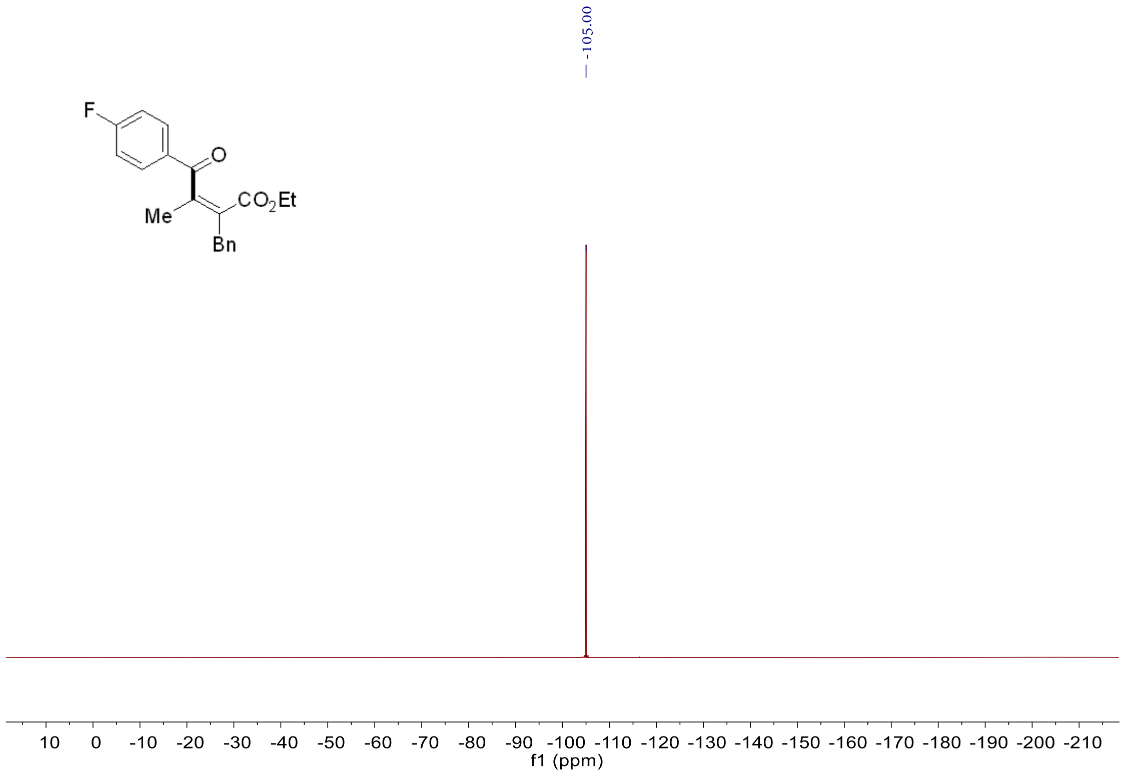

Supplementary Figure 211. ^{19}F NMR spectrum for compound **3xx**

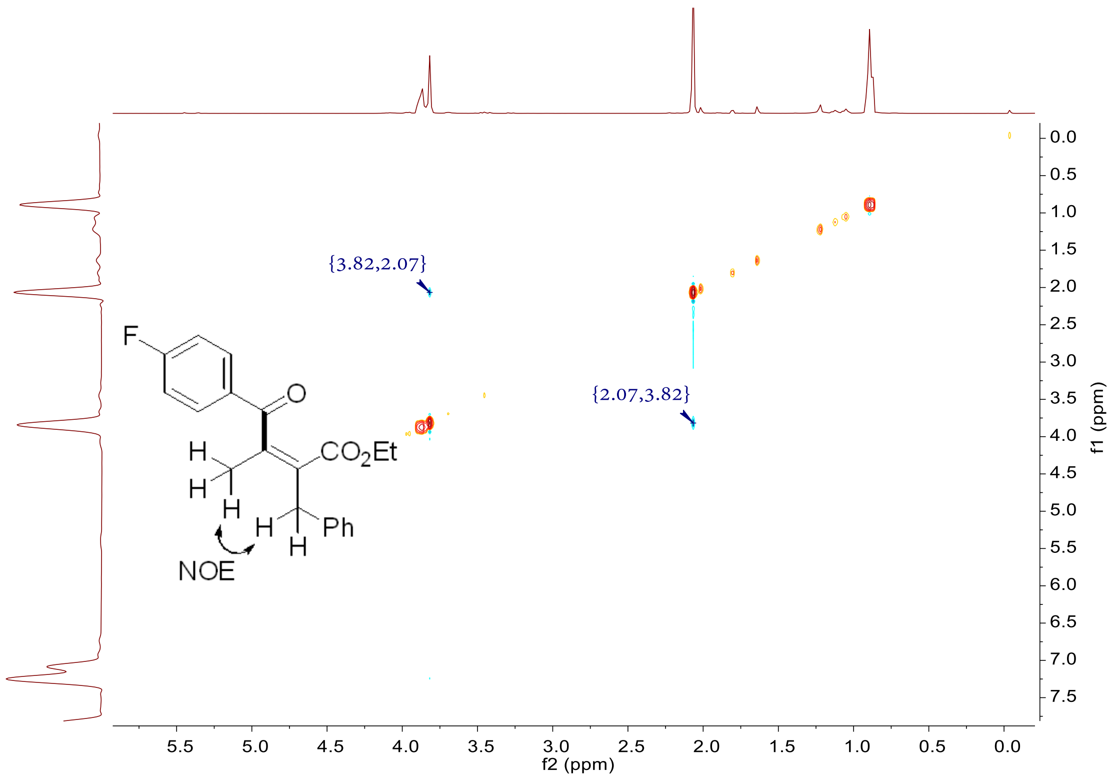

Supplementary Figure 212. NOESY spectrum for compound **3xx**

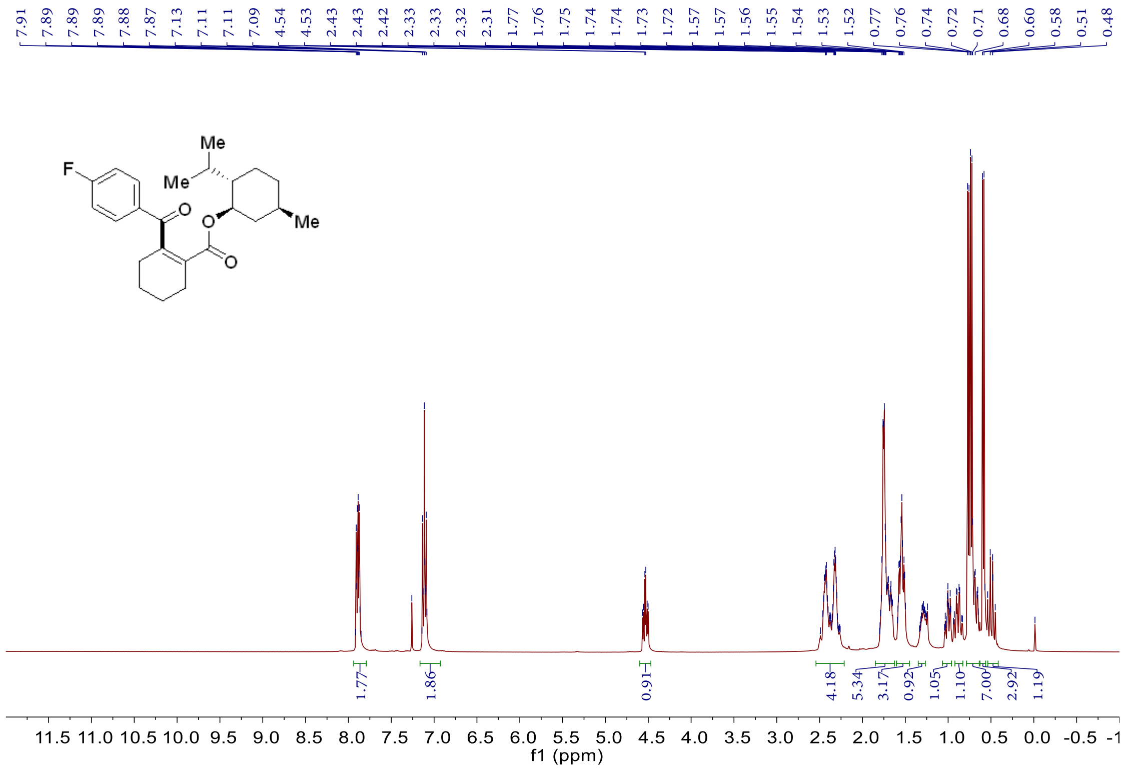

Supplementary Figure 213. ^{1}H NMR spectrum for compound **3yy**

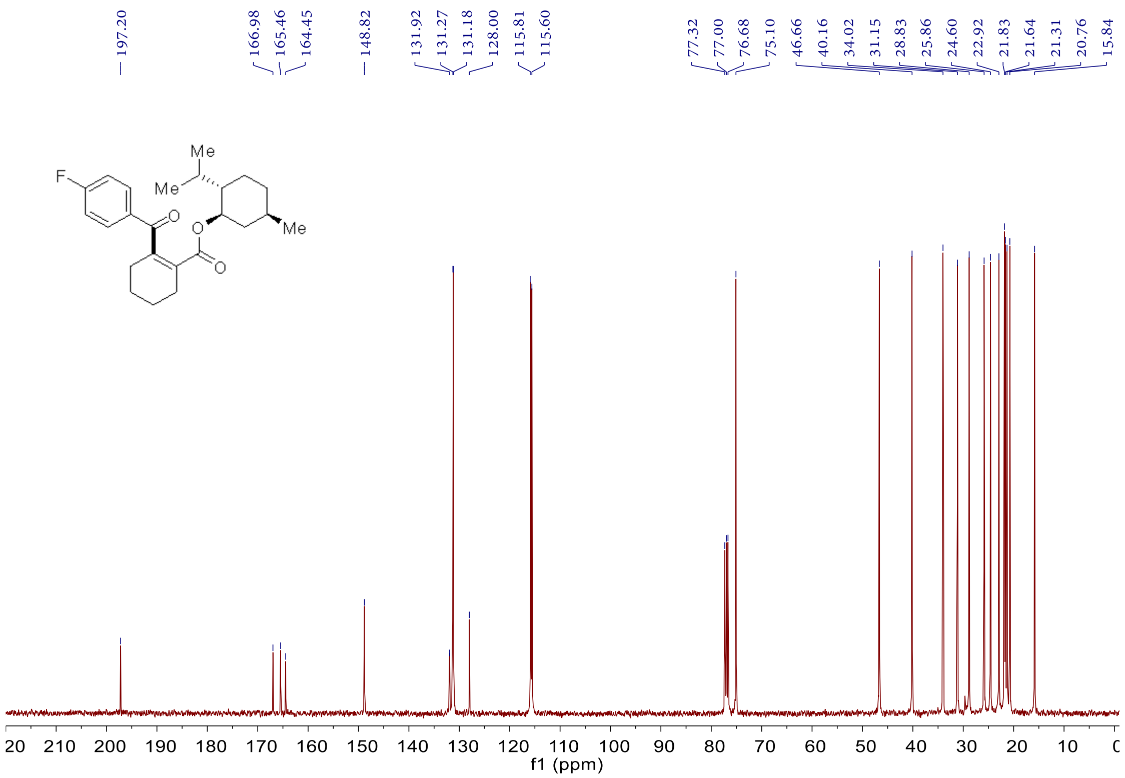

Supplementary Figure 214. ^{13}C NMR spectrum for compound **3yy**

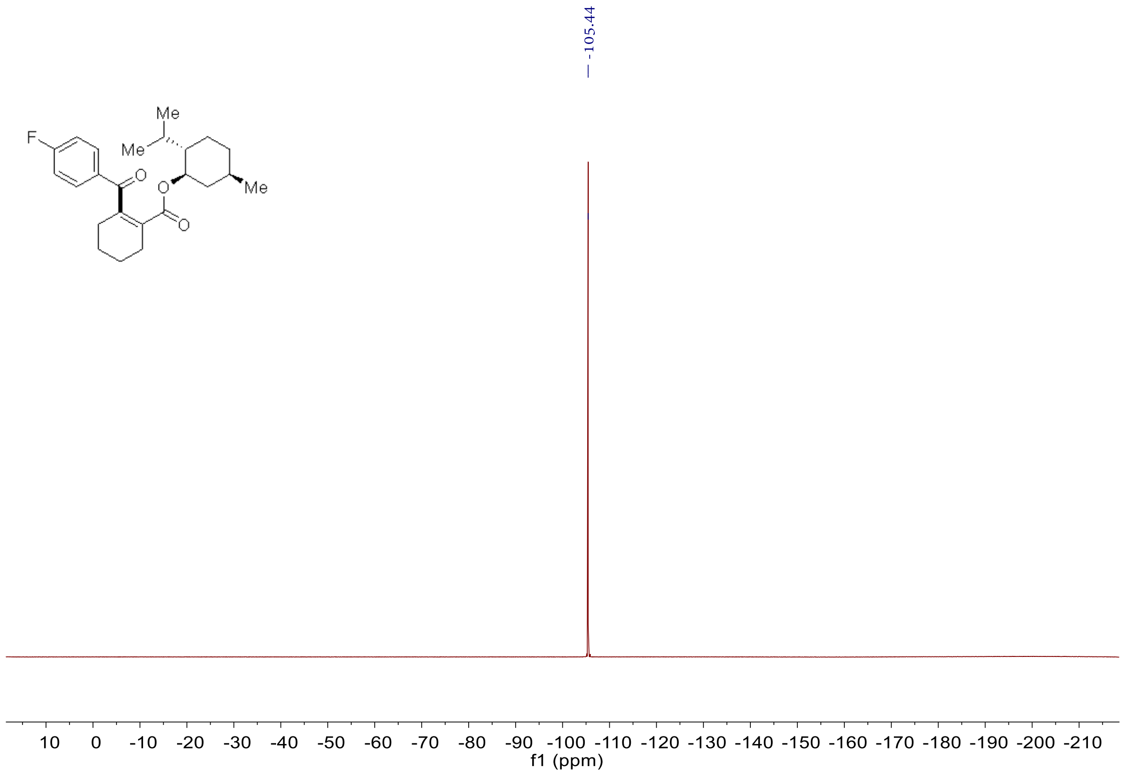

Supplementary Figure 215. ^{19}F NMR spectrum for compound **3yy**

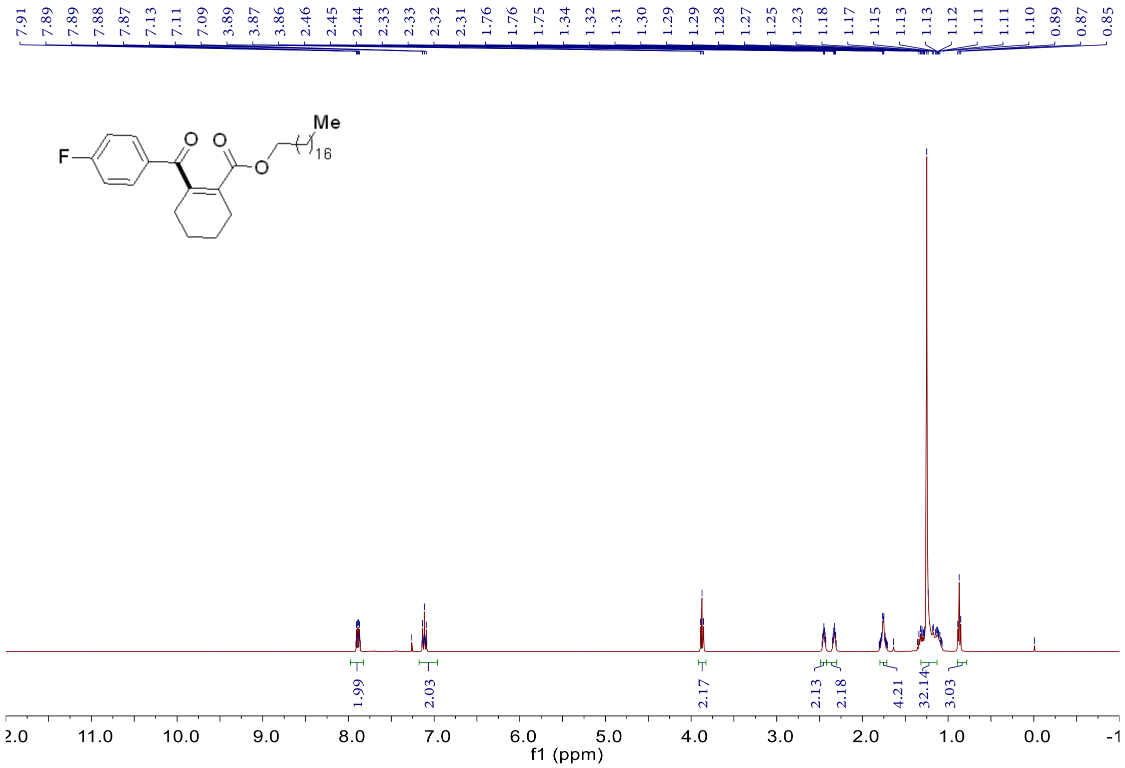

Supplementary Figure 216. ^{1}H NMR spectrum for compound **3zz**

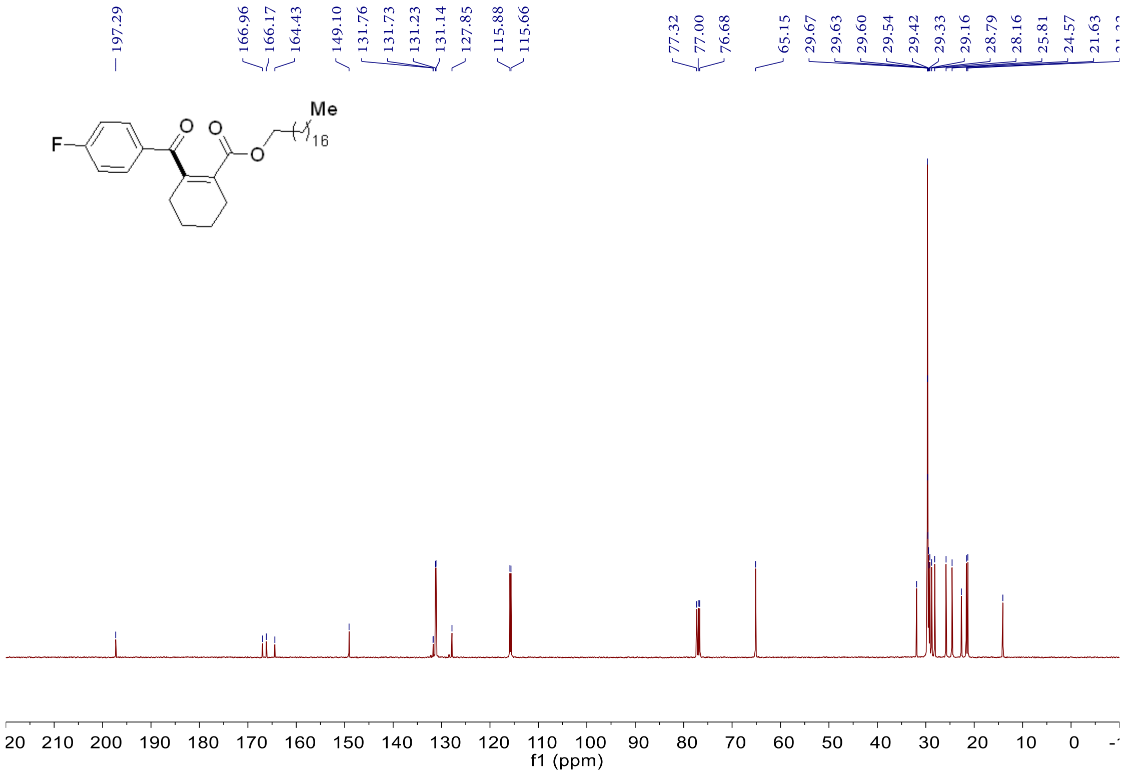

Supplementary Figure 217. ^{13}C NMR spectrum for compound **3zz**

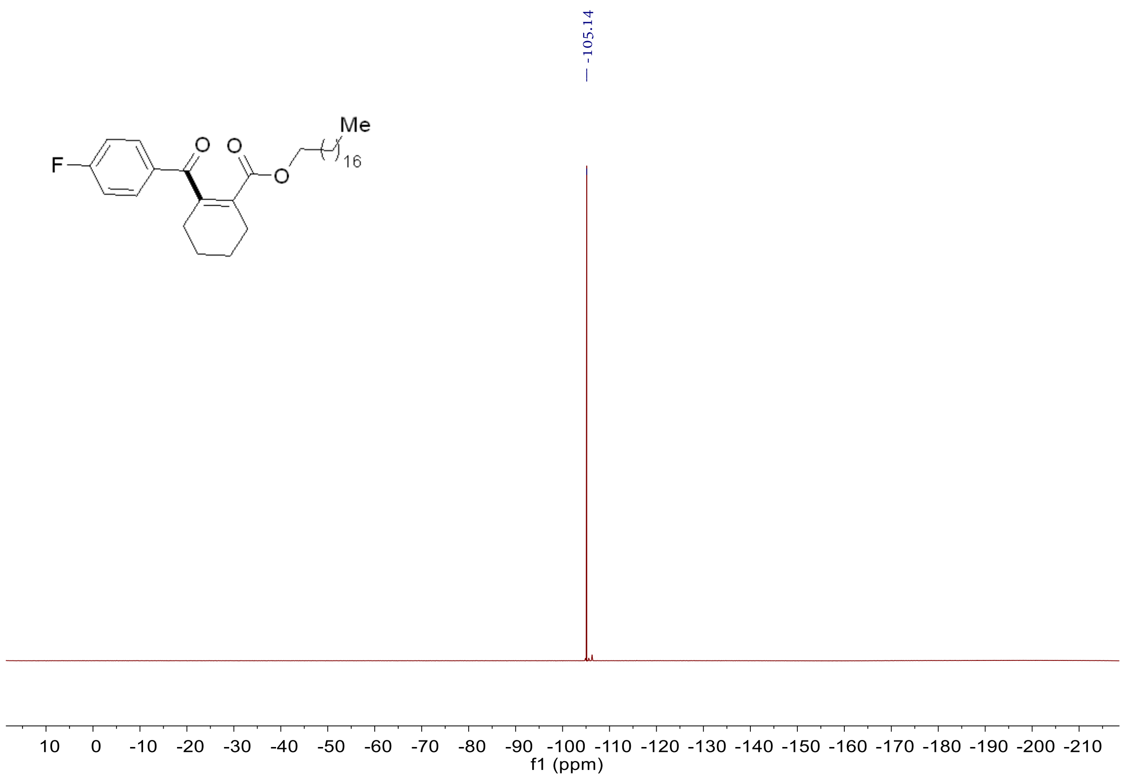

Supplementary Figure 218. ^{19}F NMR spectrum for compound **3zz**

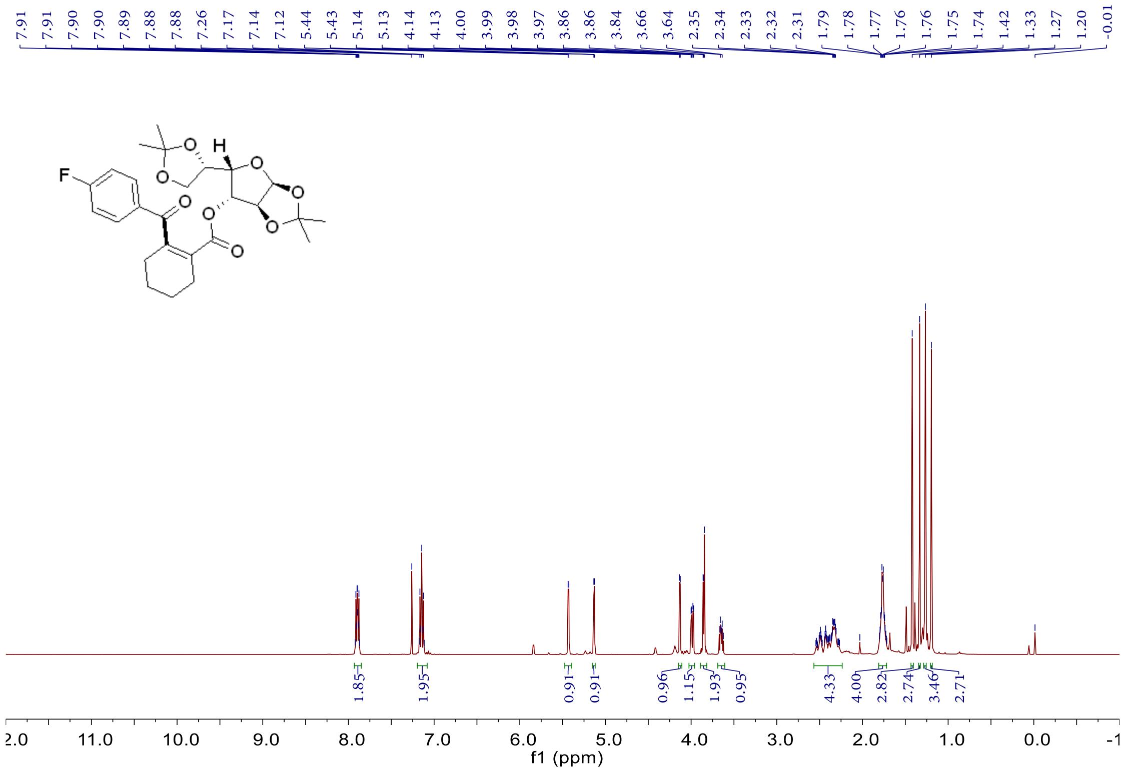

Supplementary Figure 219. ^{1}H NMR spectrum for compound **3AA**

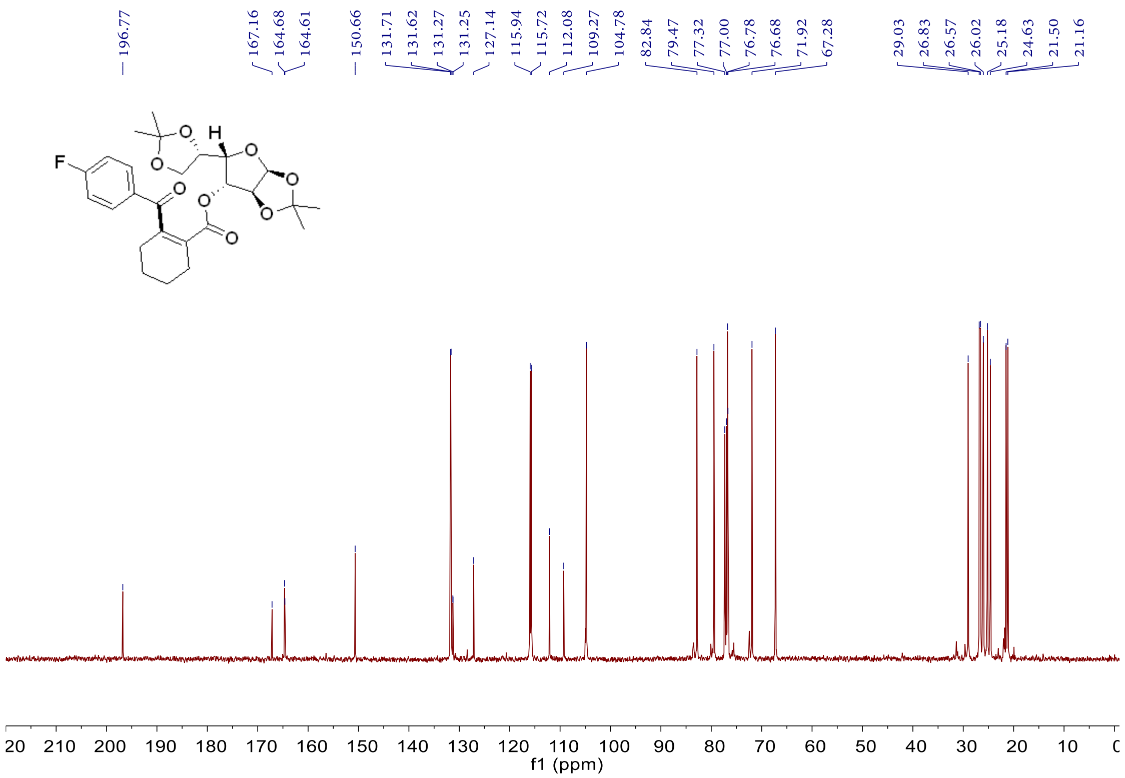

Supplementary Figure 220. ^{13}C NMR spectrum for compound **3AA**

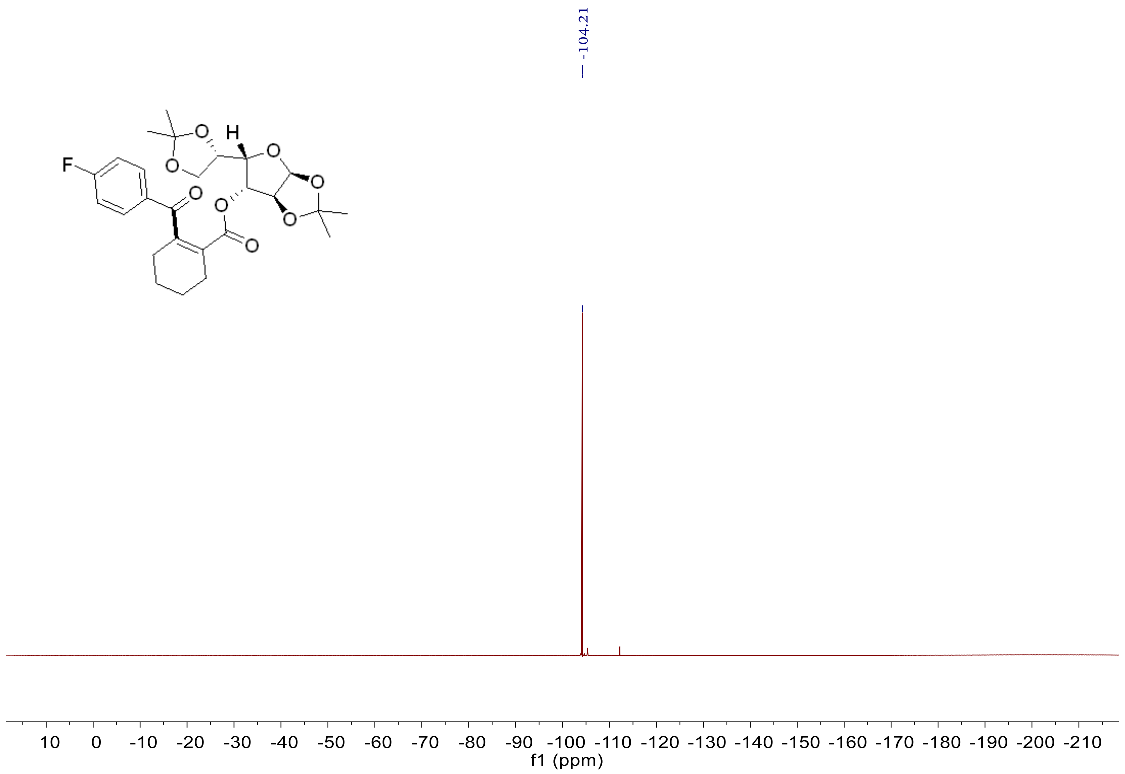

Supplementary Figure 221. ^{19}F NMR spectrum for compound **3AA**

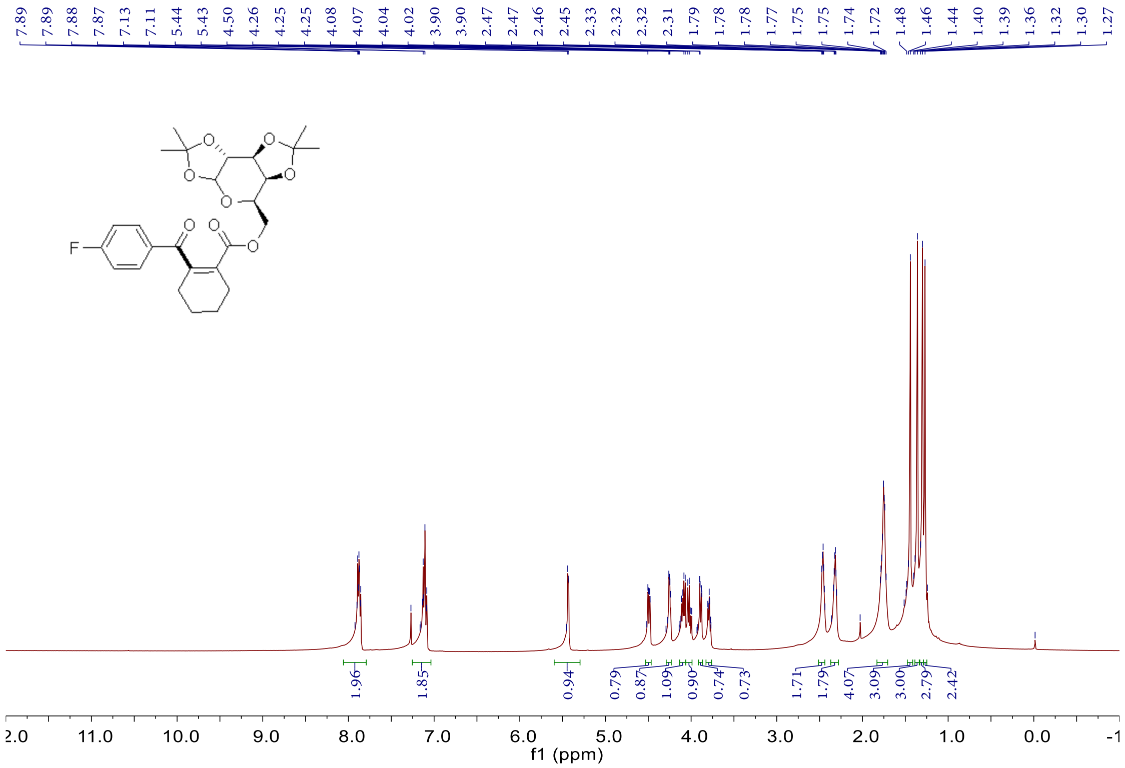

Supplementary Figure 222. ^{1}H NMR spectrum for compound **3BB**

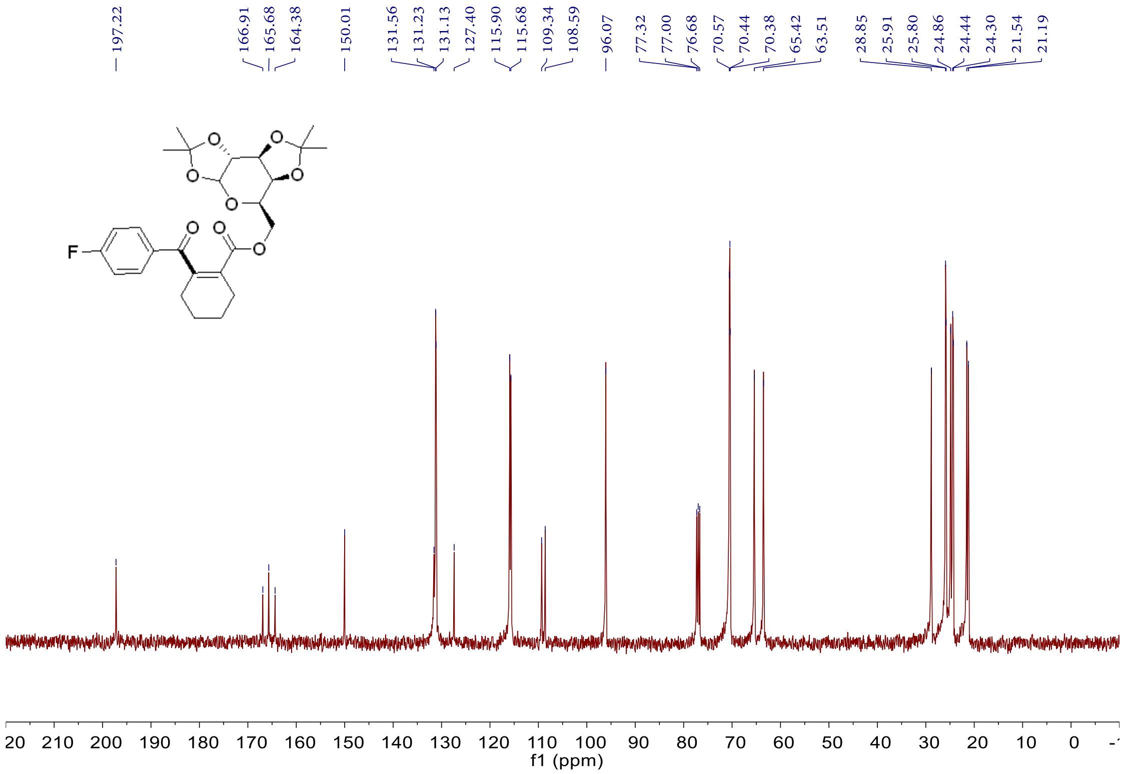

Supplementary Figure 223. ^{13}C NMR spectrum for compound **3BB**

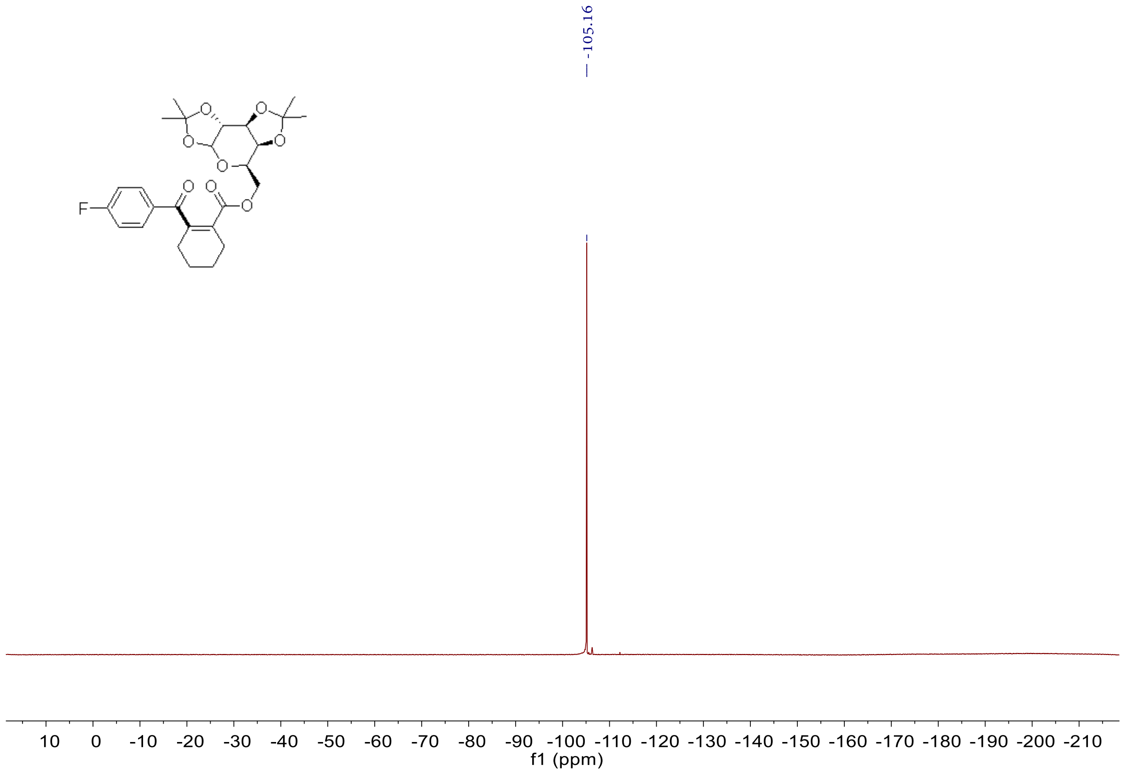

Supplementary Figure 224. ^{19}F NMR spectrum for compound **3BB**

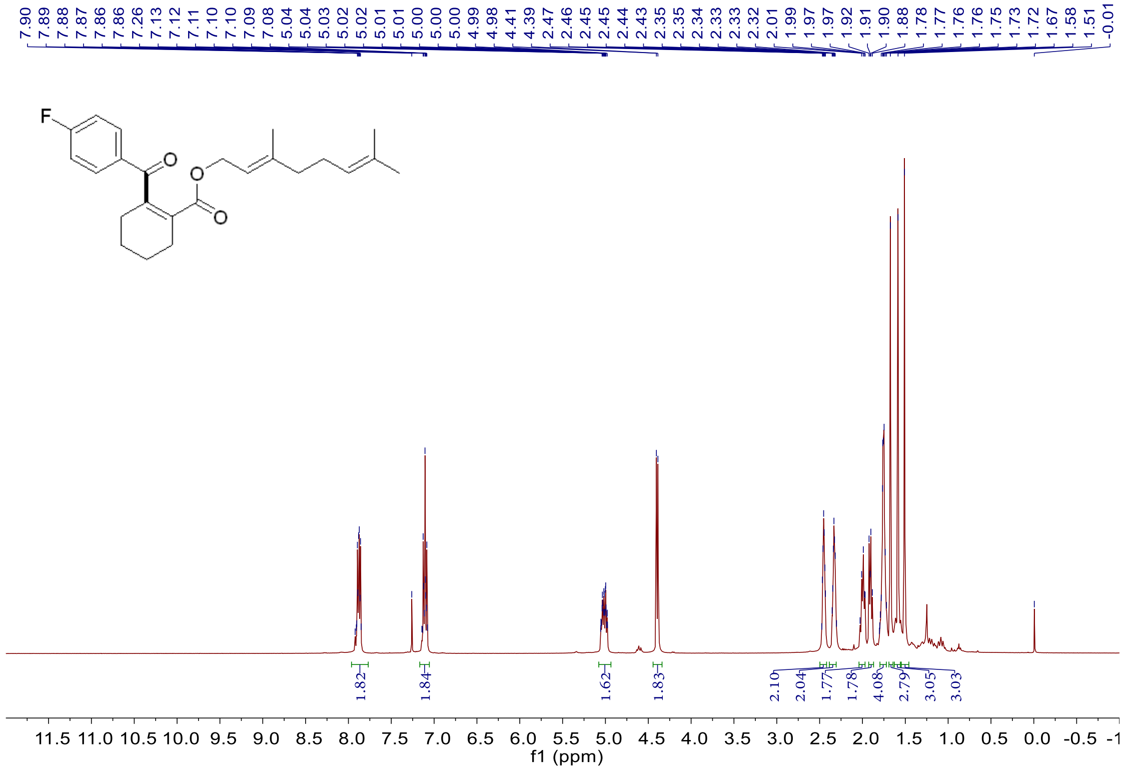

Supplementary Figure 225. ^{1}H NMR spectrum for compound **3DD**

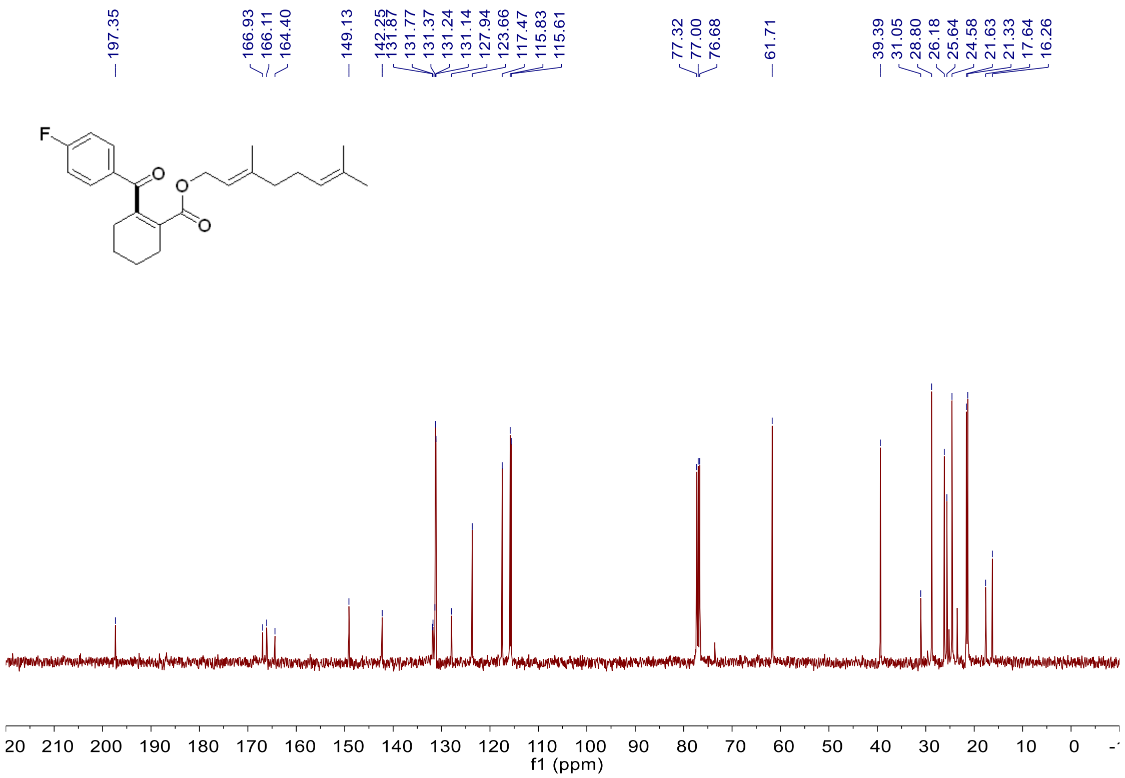

Supplementary Figure 226. ^{12}C NMR spectrum for compound **3DD**

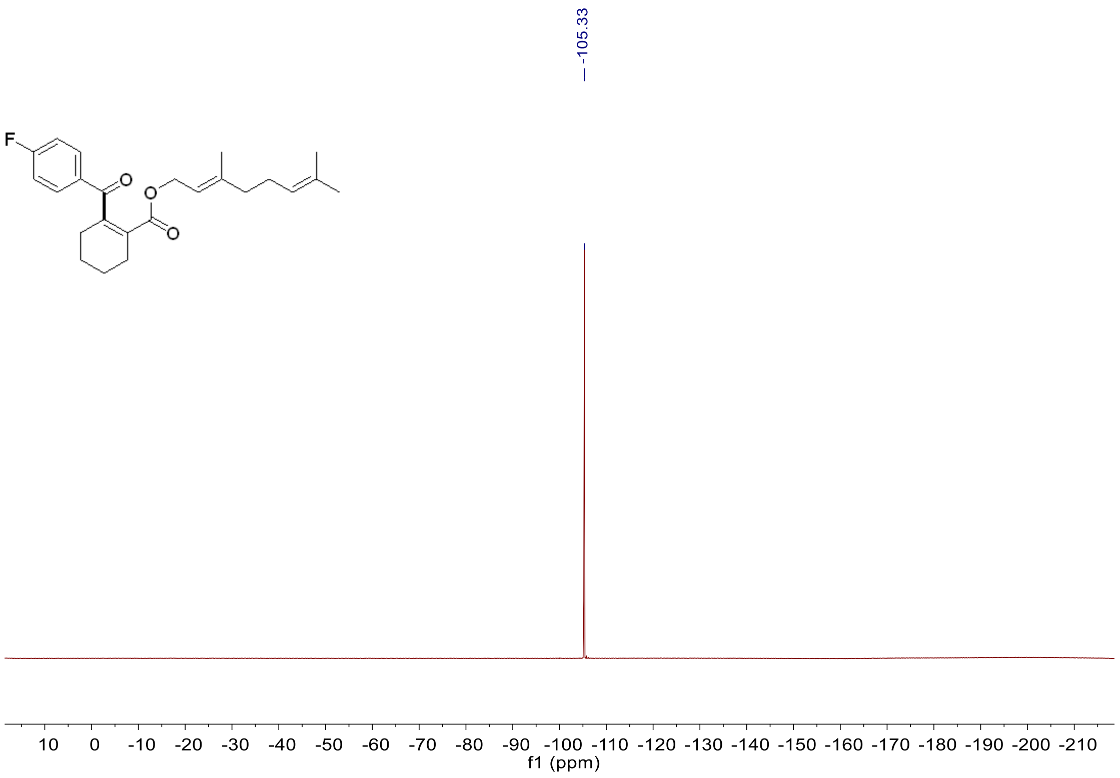

Supplementary Figure 227. ^{19}F NMR spectrum for compound **3DD**

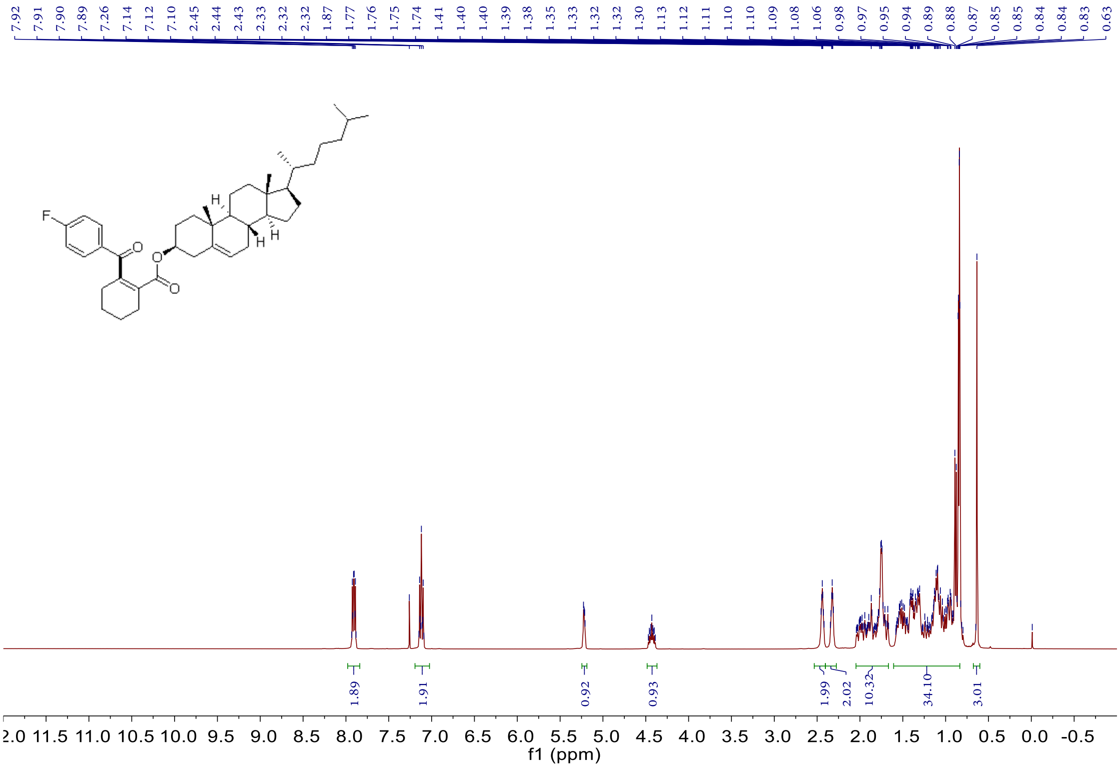

Supplementary Figure 228. ^{1}H NMR spectrum for compound **3CC**

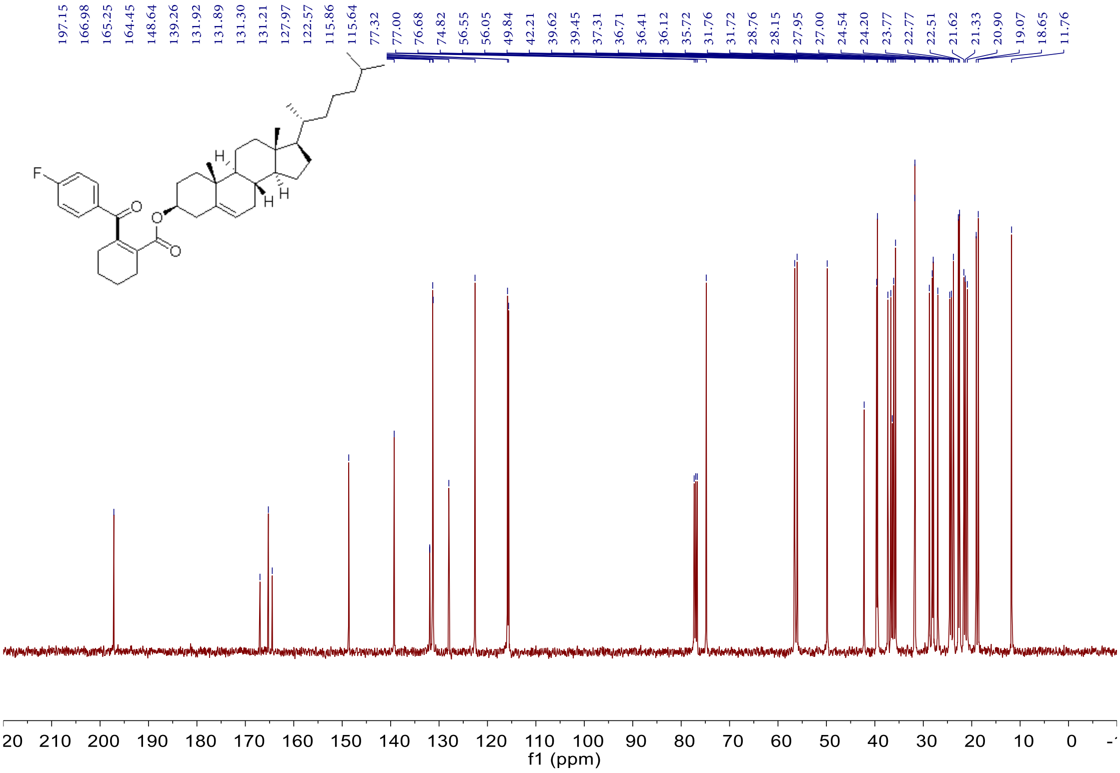

Supplementary Figure 229. ^{13}C NMR spectrum for compound **3CC**

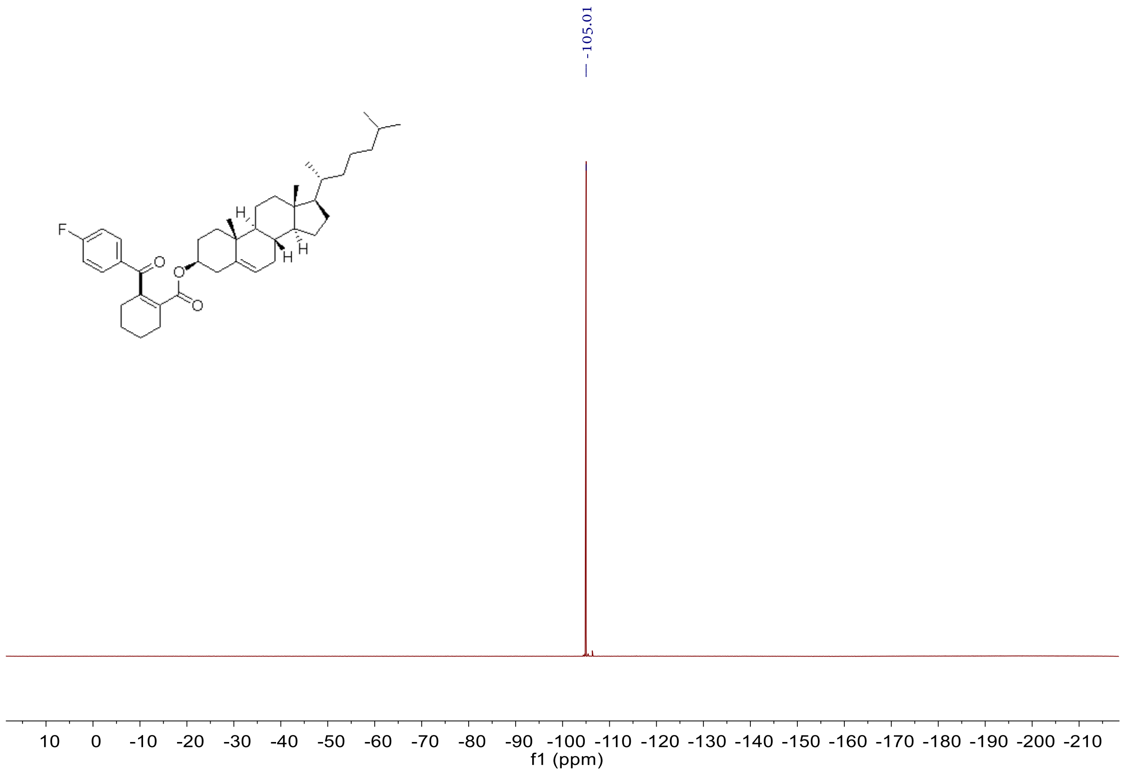

Supplementary Figure 230. ^{19}F NMR spectrum for compound **3CC**

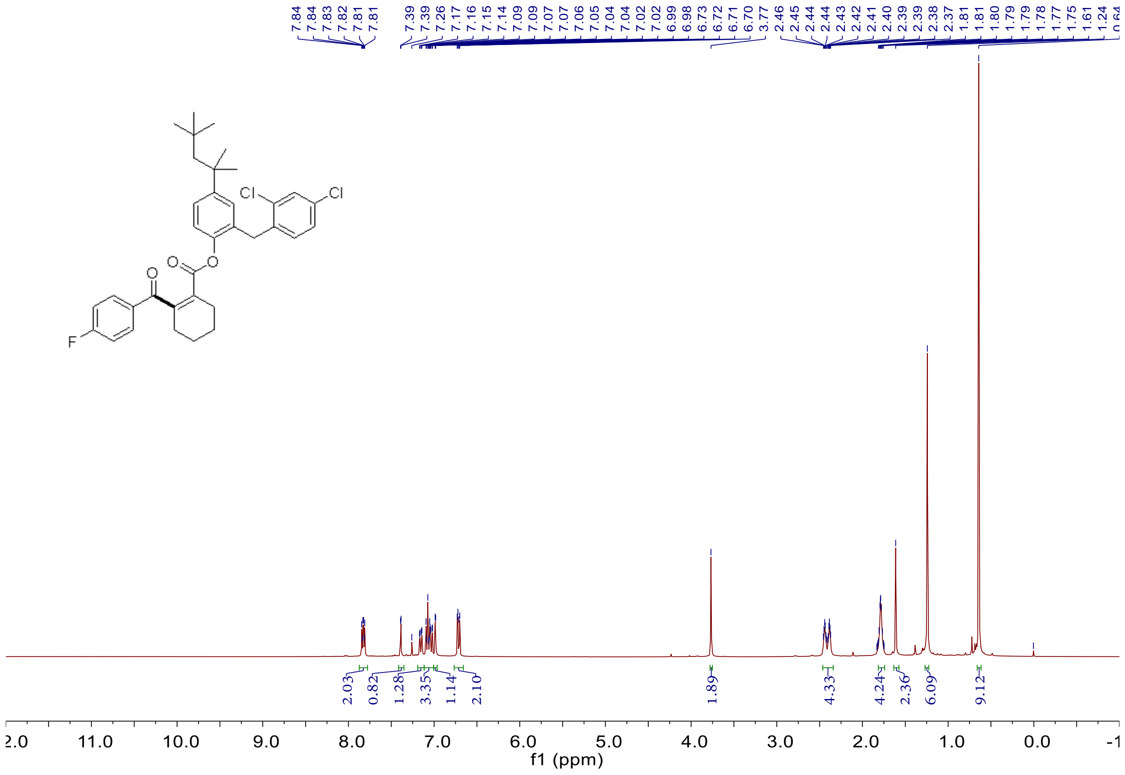

Supplementary Figure 231. ^{1}H NMR spectrum for compound **3EE**

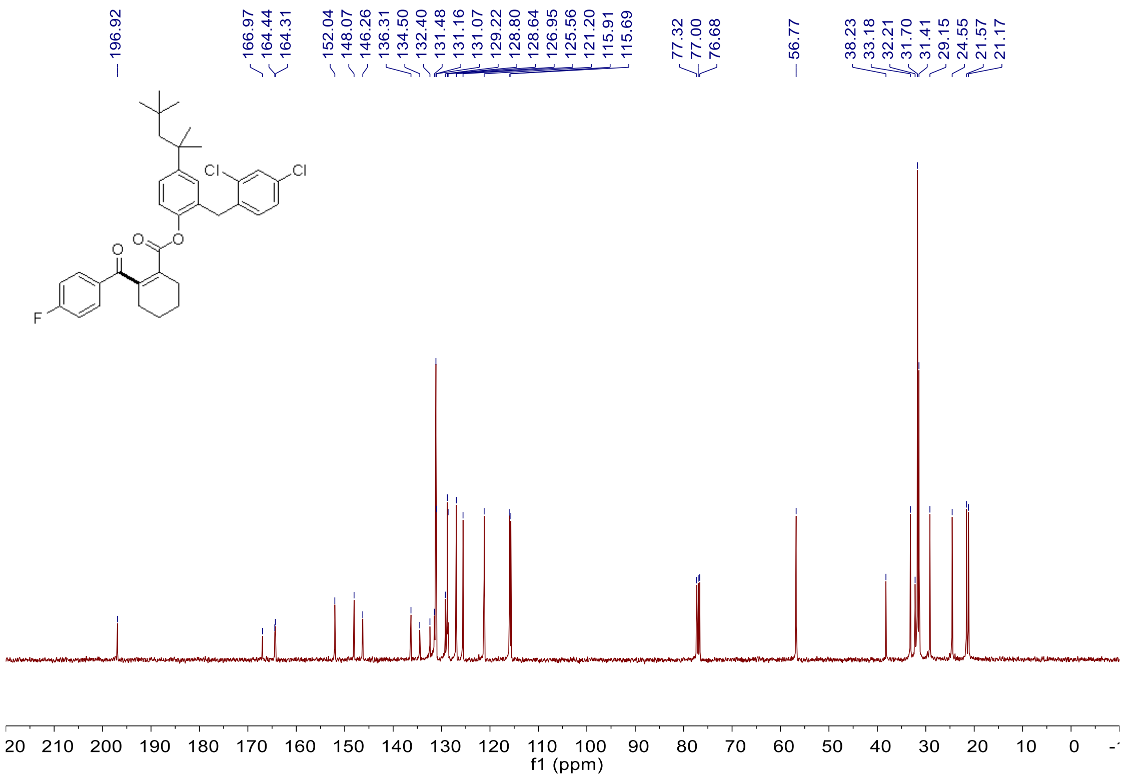

Supplementary Figure 232. ^{13}C NMR spectrum for compound **3EE**

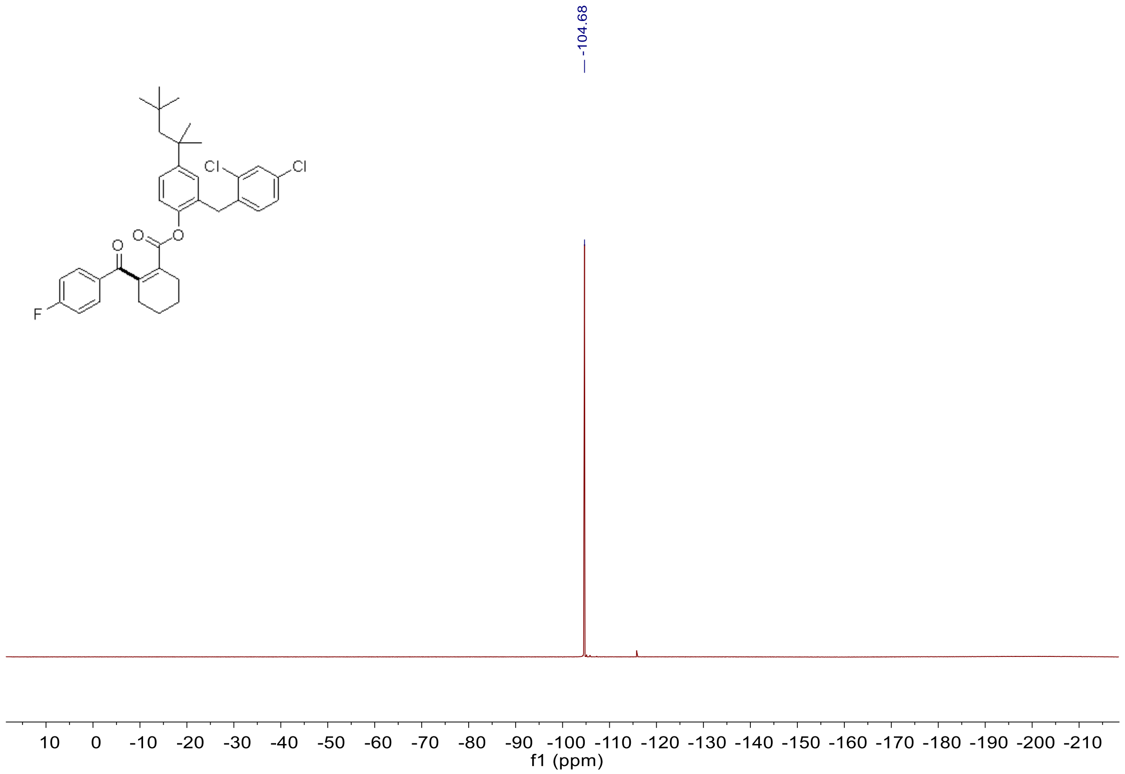

Supplementary Figure 233. ^{19}F NMR spectrum for compound **3EE**

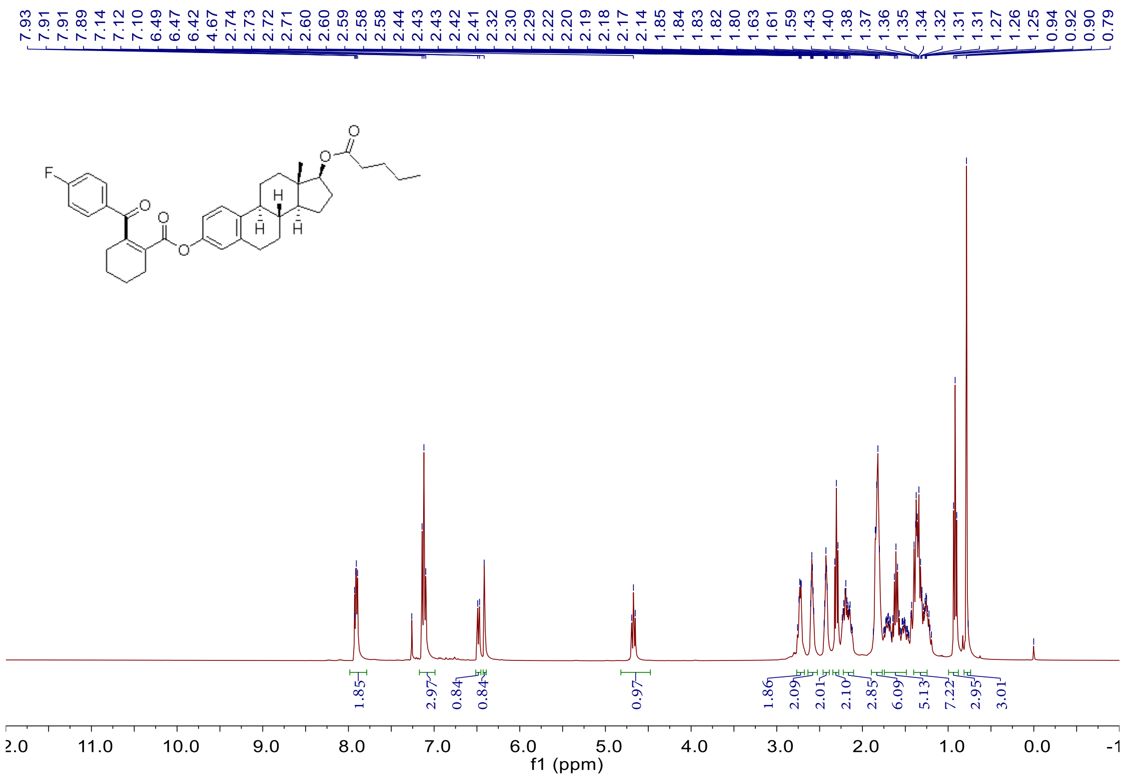

Supplementary Figure 234. ^{1}H NMR spectrum for compound **3FF**

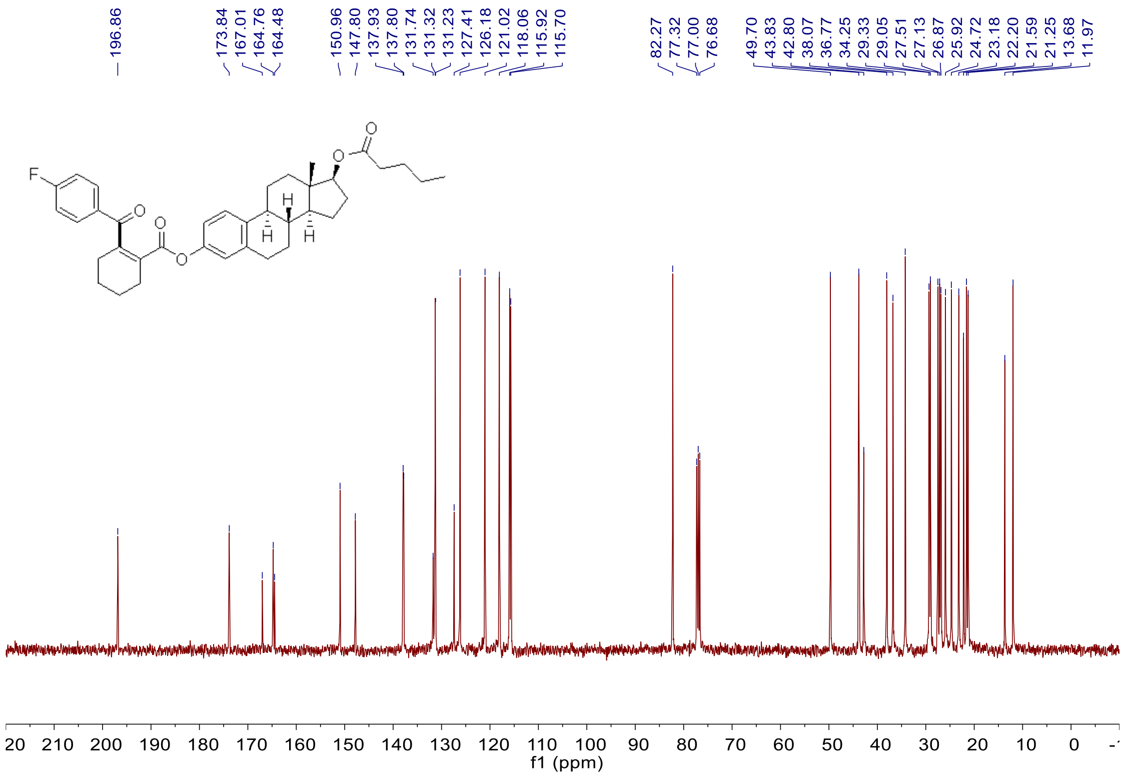

Supplementary Figure 235. ^{13}C NMR spectrum for compound **3FF**

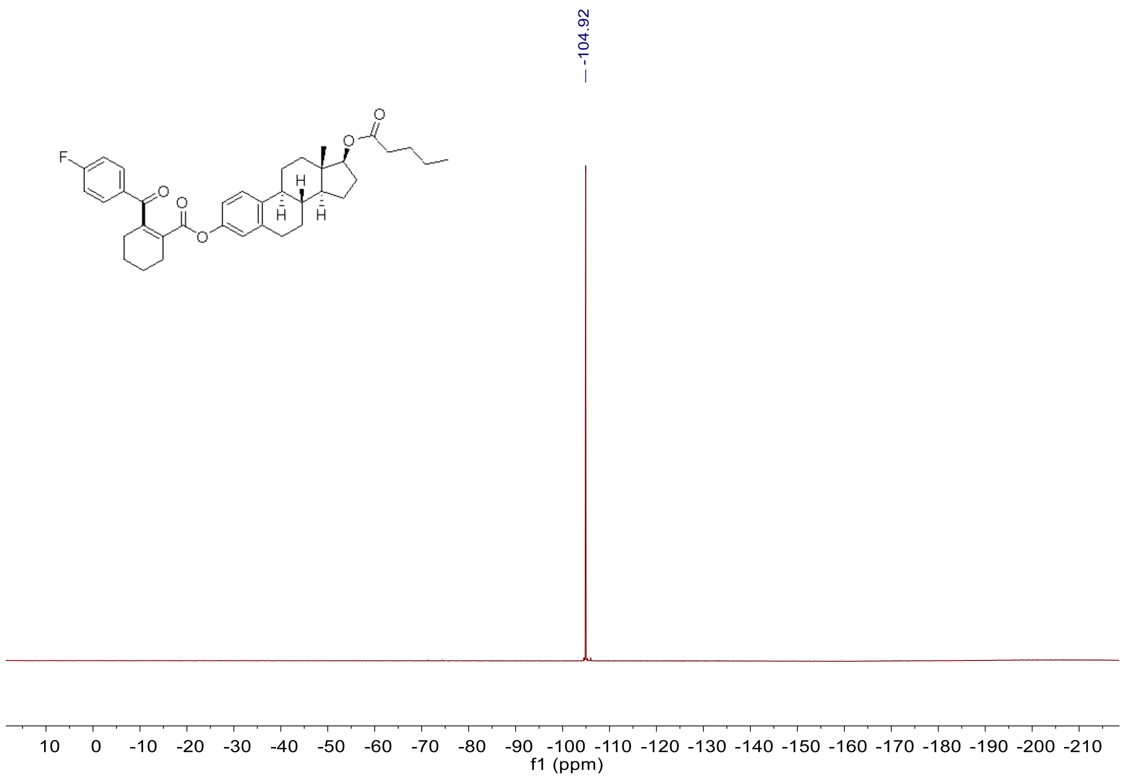

Supplementary Figure 236. ^{19}F NMR spectrum for compound **3FF**

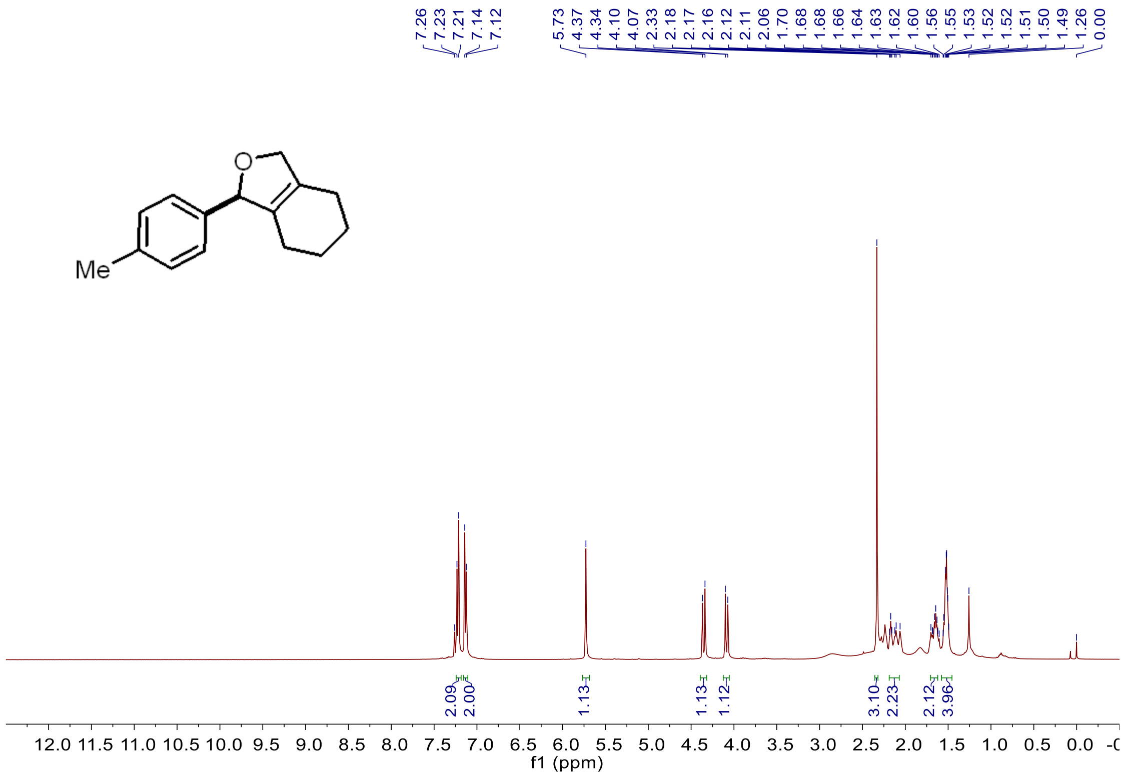

Supplementary Figure 237. ^{1}H NMR spectrum for compound **14**

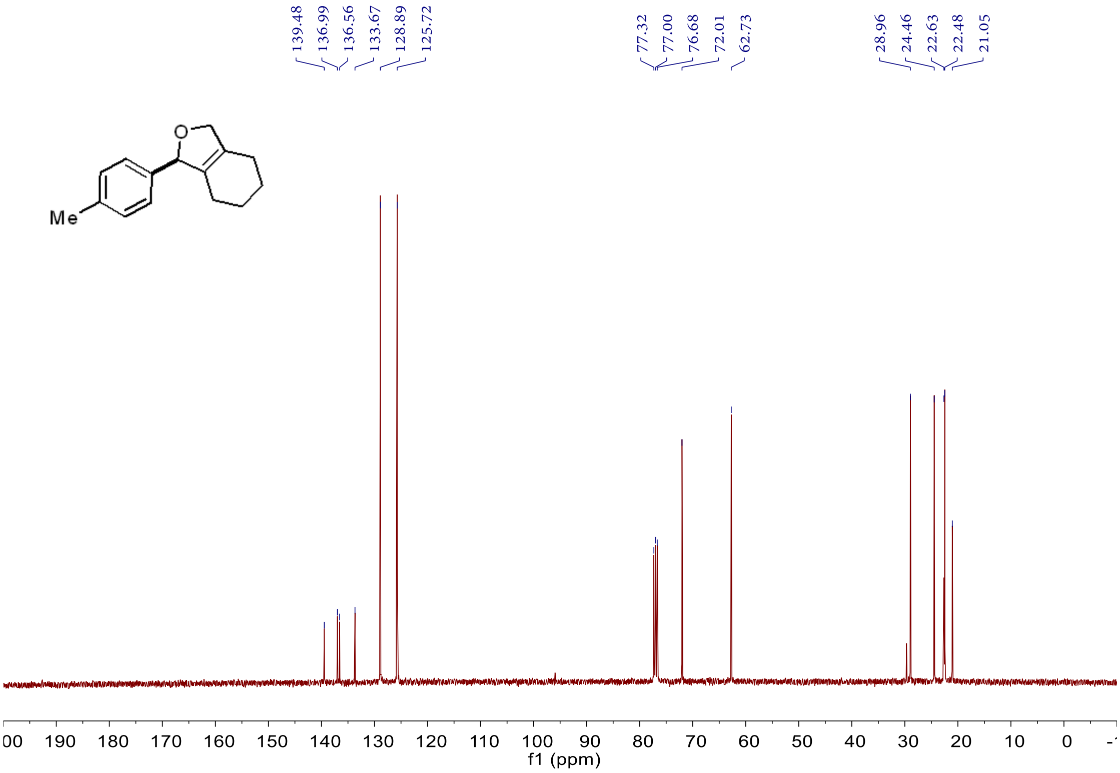

Supplementary Figure 238. ^{13}C NMR spectrum for compound **14**

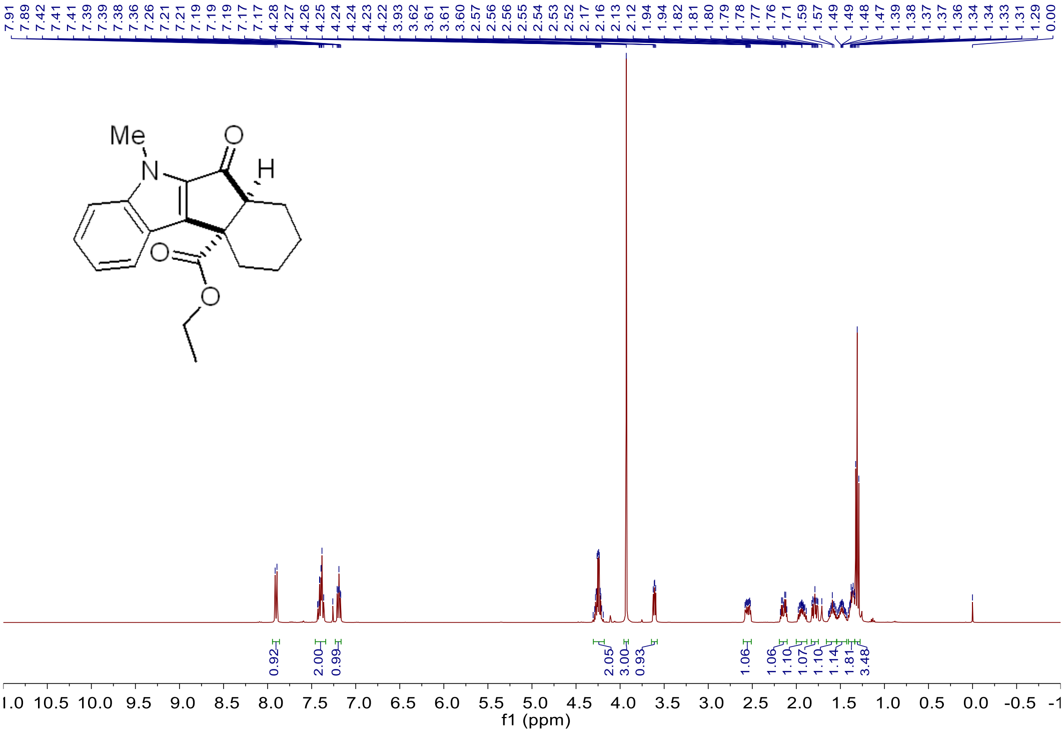

Supplementary Figure 239. ^{1}H NMR spectrum for compound **14**

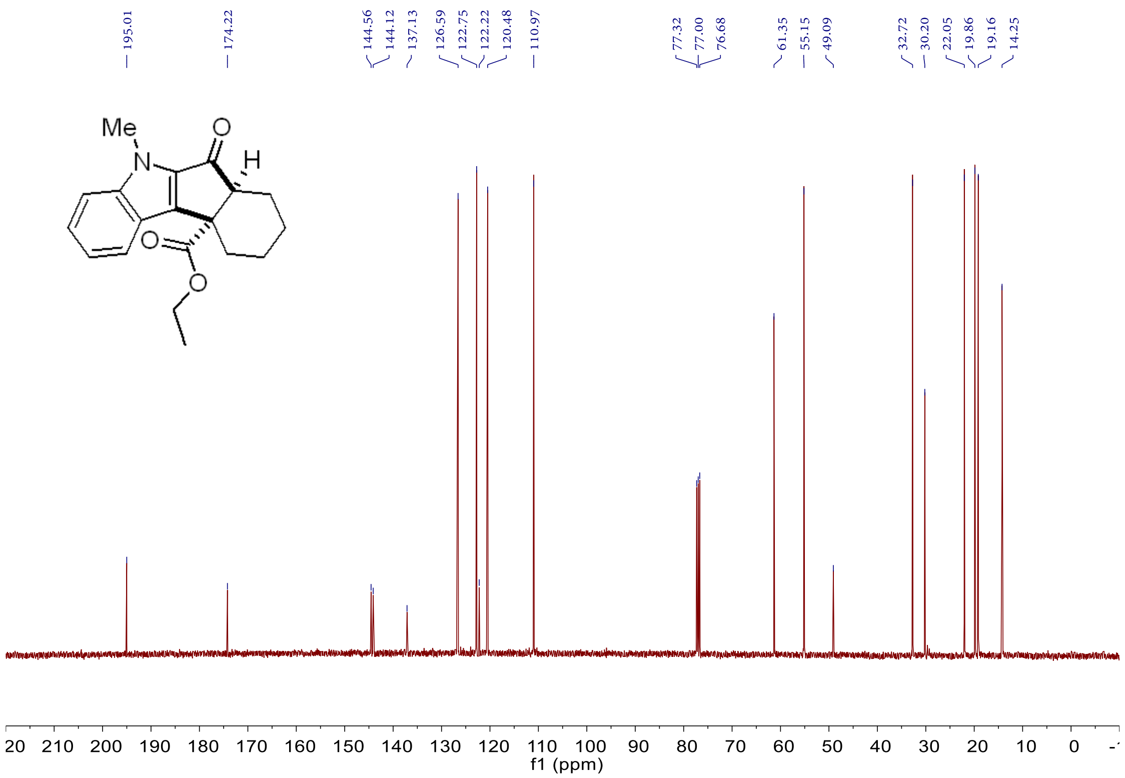

Supplementary Figure 240. ^{13}C NMR spectrum for compound **14**

3. Supplementary References

[1] W. W. Chen, A. Cunillera, D. Chen, S. Lethu, A. López de Moragas, J. Zhu, M. Solà, A. B. Cuenca, A. Shafir, *Angew. Chem., Int. Ed.* **2020**, *59*, 20201-20207.

[2] V. Saini, M. O'Dair, M. S. Sigman, *J. Am. Chem. Soc.* **2015**, *137*, 608-611.

[3] C. Kong, T. G. Driver, *Org. Lett.* **2015**, *17*, 802-805.

[4] D. Babinski, O. Soltani, D. E. Frantz, *Org. Lett.* **2008**, *10*, 2901-2904.

[5] K. Wang, C. Jiang, Z. Zhang, C. Han, X. Wang, Y. Li, K. Chen, J. Zhao, *Chem. Commun.* **2020**, *56*, 12817-12820.

[6] X. Zhao, C. Song, J. D. Rainier, *Org. Lett.* **2019**, *21*, 8611-8614.

[7] N. Su, J. A. Theorell, D. J. Wink, T. G. Driver, *Angew. Chem., Int. Ed.* **2015**, *54*, 12942-12946.

[8] M. Chen, N. Su, T. Deng, D. J. Wink, Y. Zhao, T. G. Driver, *Org. Lett.* **2019**, *21*, 1555-1558.

[9] I. T. Crouch, T. Dreier, D. E. Frantz, *Angew. Chem., Int. Ed.* **2011**, *50*, 6128-6132.

[10] S. Grimme, C. Bannwarth, S. Dohm, A. Hansen, J. Pisarek, P. Pracht, J. Seibert, F. Neese, *Angew. Chem., Int. Ed.* **2017**, *56*, 14763-14769; b) S. Grimme, *J. Chem. Theory Comput.* **2019**, *155*, 2847-2862.

[11] a) S. Grimme, C. Bannwarth, P. Shushkov, *J. Chem. Theory Comput*. **2017**, 13, 1989-2009; b) C. Bannwarth, S. Ehlert and S. Grimme, *J. Chem. Theory Comput*. **2019**, *15*, 1652-1671; c) P. Pracht, E. Caldeweyher, S. Ehlert, S. Grimme, *ChemRxiv*, **2019**, preprint. DOI: 10.26434/chemrxiv.8326202.v1.

[12] M. J. Frisch, *et al.* Gaussian 16 Revision A.03. **2016**.

[13] C. Adamo and V. Barone, *J. Chem. Phys.* **1999**, *110*, 6158-6169.

[14] S. Grimme, S. Ehrlich and L. Goerigk, *J. Comp. Chem.* **2011**, *32*, 1456-1465.

[15] F. Weigend and R. Ahlrichs, *Phys. Chem. Chem. Phys.* **2005**, *7*, 32973-305.

[16] A. V. Marenich, C. J. Cramer, and D. G. Truhlar, *J. Phys. Chem. B* **2009**, *113*, 6378-6396.

[17] S. Dohm, A. Hansen, M. Steinmetz, S. Grimme and M. P. Checinski, *J. Chem. Theory Comput.* **2018**, *14*, 2596–2608.

[18] G. Luchini, J. V. Alegre-Requena, Y. Guan, I. Funes-Ardoiz, R. S. Paton GoodVibes: GoodVibes 3.0.1, **2019**, http://doi.org/10.5281/zenodo.595246.

[19] S. Grimme, *Chem. Eur. J.* **2012**, *18*, 9955-9964.

[20] Y. Li, J. Gomes, S. M. Sharada, A. T. Bell, M. J. Head-Gordon, *Phys. Chem. C* **2015**, *119*, 1840-1850.
